# Proteomic and Biochemical Analyses of the Cotyledon and Root of Flooding-Stressed Soybean Plants

**DOI:** 10.1371/journal.pone.0065301

**Published:** 2013-06-14

**Authors:** Setsuko Komatsu, Takahiro Makino, Hiroshi Yasue

**Affiliations:** 1 National Institute of Crop Science, NARO, Tsukuba, Japan; 2 Graduate School for the Creation of New Photonics Industries, Hamamatsu, Japan; 3 National Institute of Agrobiological Sciences, Tsukuba, Japan; RIKEN Plant Science Center, Japan

## Abstract

**Background:**

Flooding significantly reduces the growth and grain yield of soybean plants. Proteomic and biochemical techniques were used to determine whether the function of cotyledon and root is altered in soybean under flooding stress.

**Results:**

Two-day-old soybean plants were flooded for 2 days, after which the proteins from root and cotyledon were extracted for proteomic analysis. In response to flooding stress, the abundance of 73 and 28 proteins was significantly altered in the root and cotyledon, respectively. The accumulation of only one protein, 70 kDa heat shock protein (HSP70) (Glyma17g08020.1), increased in both organs following flooding. The ratio of protein abundance of HSP70 and biophoton emission in the cotyledon was higher than those detected in the root under flooding stress. Computed tomography and elemental analyses revealed that flooding stress decreases the number of calcium oxalate crystal the cotyledon, indicating calcium ion was elevated in the cotyledon under flooding stress.

**Conclusion:**

These results suggest that calcium might play one role through HSP70 in the cotyledon under flooding stress.

## Introduction

Flooding has a significant negative influence on the productivity of arable farmland, as the vast majority of crops cannot grow under the stress conditions induced by flooding [1]. Most studies of flooding stress have focused on relatively flood-tolerant species such as rice, *Rumex*, and *Echinochloa* [2]. Soybean, however, is sensitive to flooding stress, and its growth and grain yield are significantly reduced by flooding [3], [4]. Hashiguchi et al. [5] showed that soybean seedling root elongation is suppressed after 1 day of flooding stress and is significantly retarded by 2 days of flooding stress. Suppression of root elongation occurred in the root tip region [6], [7] since it contains the root apical meristem, which is important for root system development and also contains the elongation region [8].

Proteomic studies of the total protein complement of the soybean root have indicated that flooding stress alters the abundance of proteins associated with protein transport, protein storage, ATP synthesis, metabolism, and signal transduction pathways [4]. Recent studies using omics techniques [9] have identified numerous flooding-responsive pathways and systems in plants, including hormonal signaling [10], transcriptional control [10], glucose degradation and sucrose accumulation [11], activation of alcohol fermentation [12], the gamma-aminobutyric acid shunt [4], suppression of reactive oxygen species scavenging system [4], suppression of mitochondria [13], ubiquitin/proteasome-mediated proteolysis [6], [14], and the cell wall [15]. Flooding-induced changes in these systems and pathways have been well-documented with regard to the root and hypocotyl of young soybeans; however, there are no reports on the effect of flooding stress on the function of other soybean organs, such as the cotyledon.

Khatoon et al. [16], [17] used gel-based proteomic techniques to examine organ-specific responses in soybean plants flooded for 12 days. In the root, hypocotyl, and leaf, the abundance of 51, 66, and 51 proteins, respectively, changed significantly in response to flooding stress. The abundance of many metabolism-related proteins increased in the root but decreased in both the hypocotyl and leaf. Isoflavone reductase decreased at the protein level in all 3 organs under flooding stress, but expression of isoflavone reductase mRNA was up-regulated in leaf. In addition, flooding stress led to an increase in biophoton emission in all 3 organs. These results suggested that concurrence of the expression of the isoflavone reductase gene at the mRNA and protein levels, together with imbalances in the levels of disease/defense and metabolism-related proteins, might be responsible for the observed impaired growth of root, hypocotyl, and leaf of flooding-stressed soybean seedlings. Such organ-specific analyses have provided many important insights into the flooding response mechanism in soybean.

Biophotons generated during germination of soybean seeds are composed of two spectral components, a UV component, corresponding to what was originally described as “mitogenetic radiation”, and emission in the red and far-red regions [18]. Biophoton emission has been detected in cut potato tubers infected with *Fusarium* [19]. In soybean, biophoton emission has been detected in cadmium-stressed leaf [20] and drought-stressed root [21]. Under flooding stress, biophoton emission has also been detected in leaf and root of soybean [16]; however, the molecular mechanisms underlying biophoton emission are unknown, although biophotons are thought to represent spontaneous. In this study, proteomic and biochemical techniques were used to determine whether flooding stress alters root and cotyledon function in soybean. Biochemical techniques such as biophoton emission analysis, computed tomography, and elemental analysis were used to obtain a more detailed understanding of how the function of cotyledon and root during flooding stress.

## Results

### Flooding-stress-induced Changes in the Abundance of Proteins in the Root and Cotyledon of Soybean Plants

**Figure 1:**
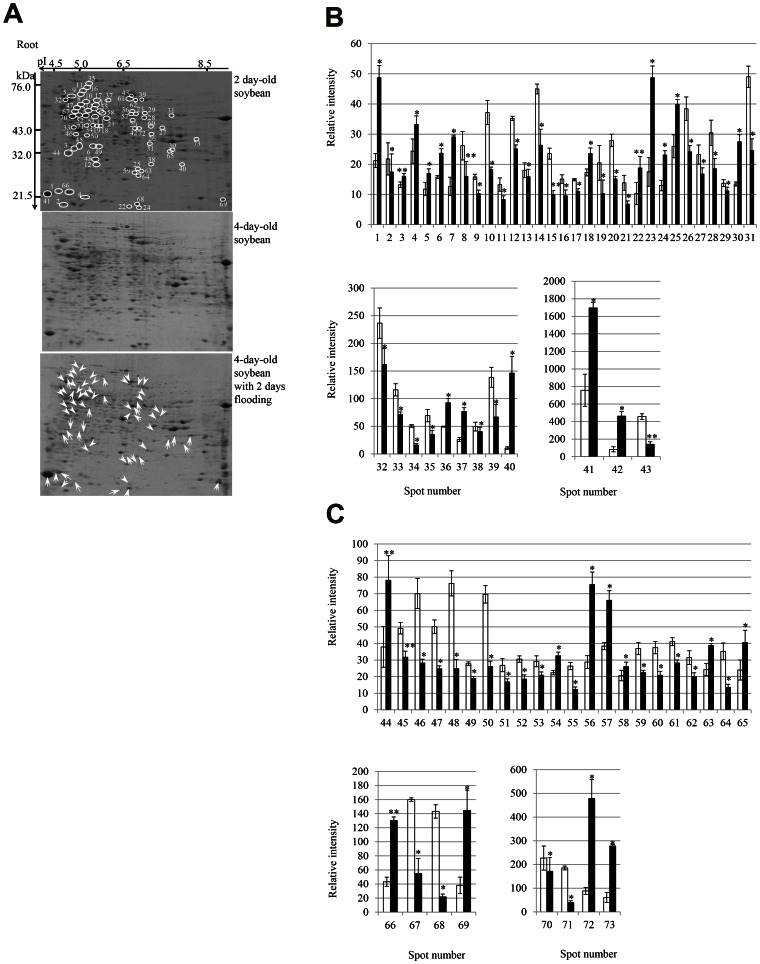
2-DE pattern and relative abundance of proteins in the root of flooding-stressed soybean plants. Two-day-old soybean plants were flooded for 2 days, after which proteins were extracted from the root, separated by 2-DE, and the gels were stained with CBB (A). Open circles denote protein spots showing altered staining intensity. Upward and downward arrows indicate increase or decrease in intensity, respectively. The differential abundance of proteins was determined using PDQuest software and is plotted as the relative intensity (B and C). Results are presented as the mean ± SE of relative protein intensity for gels from 3 biological replicates (Figure S1). Differences were compared using the Student’s *t* test (*P<0.05, **P<0.01). White and black bars show control and treatment, respectively. Spots numbers are the same as shown in panel A.

**Figure 2:**
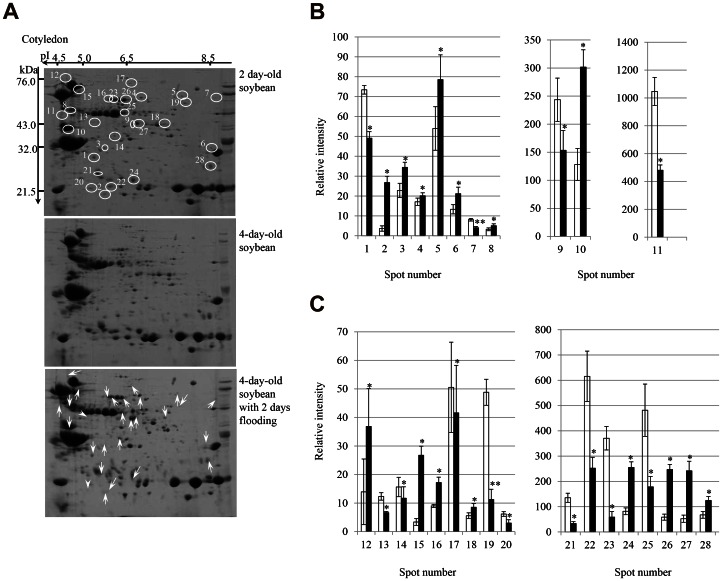
2-DE pattern and relative abundance of proteins in the cotyledon of flooding-stressed soybean plants. Two-day-old soybean plants were flooded for 2 days, after which proteins were extracted from the cotyledon, separated by 2-DE, and the gels were stained with CBB (A). Open circles denote protein spots showing altered staining intensity. Upward and downward arrows indicate increase or decrease in intensity, respectively. The differential abundance of proteins was determined using PDQuest software and is plotted as the relative intensity (B and C). Results are presented as the mean ± SE of relative protein intensity for gels from 3 biological replicates (Figure S2). Differences were compared using the Student’s *t* test (*P<0.05, **P<0.01). White and black bars show control and treatment, respectively. Spots numbers are the same as shown in panel A.

A proteomic approach was used to identify changes in protein abundance in the root and cotyledon during the early stages of growth in flooding-stressed soybean plants. Two-day-old soybean plants were flooded for 2 days. Proteins were extracted from the root and cotyledon, separated using two-dimensional polyacrylamide gel electrophoresis (2-DE), and the gels were stained with Coomassie brilliant blue (CBB) (Figure 1A) (Figure 2A). Three independent experiments were performed as biological replicates (Figure S1) (Figure S2). The relative intensity of protein spots was determined using PDQuest software (Table S1) (Table S2) (Figure 1B, 1C) (Figure 2B, 2C). A total of 615 and 377 protein spots were reproducibly detected on 2-DE gels of the root (Figure 1A and cotyledon (Figure 2A), respectively, of 2-day-old soybeans. Flooding stress resulted in significant changes in the abundance of 73 and 28 proteins in the root (Figure 1C) and cotyledon (Figure 2C), respectively, compared with 2-day-old (Figure 1A) (Figure 2A) and 4-day-old (Figure 1B) (Figure 2B) soybeans. Of the 615 total protein spots comprising the 2-DE pattern of root proteins, the staining intensity of 73 was significantly altered, 27 showing an increase in intensity and 46 showing a decrease in intensity in response to flooding stress (Figure 1). In the cotyledon, the intensity of 28 of 377 protein spots comprising the 2-DE pattern significantly changed in response to flooding stress, and of these 28 spots, 16 had increased in intensity and 12 had decreased (Figure 2).

### Identification of Proteins that Change in Abundance in the Soybean Root and Cotyledon in Response to Flooding Stress

Soybean root and cotyledon proteins that had changed in abundance in response to flooding stress as determined using 2-DE were subsequently identified using nano liquid chromatography (LC)- mass spectrometry (MS)/MS. Peak lists of the identified proteins are provided in Tables (Table S3) (Table S4). A total of 73 and 28 flooding-responsive proteins were identified in the root (Table 1) and cotyledon (Table 2), respectively. A total of 5 root proteins that increased in abundance at least 3-fold in response to flooding stress were identified: decarboxylase isozyme 1 (spot number R37), stem 28 kDa glycoprotein (spot number R40), alcohol dehydrogenase 1F (spot number R42), peptidyl-prolyl cis-trans isomerase 1 (spot number R69), and fructose-bisphosphate aldolase (spot number R73) (Figure 1) (Table 1). A total of 4 root proteins that decreased in abundance at least 3-fold in response to flooding stress were identified: ripening-related protein (spot number R68), S-adenosylmethionine synthetase (spot number R71), and an unknown protein (spot number R72).

**Table 1:** Flooding-responsive proteins identified in soybean root using LC-MS/MS.

					Theoretical					
Spot no	Homologous protein	Species	Protein ID	Accession no	Mr (Da)	pI	Score	MP	Cov(%)	BlastScore
1	Kunitz trypsin protease inhibitor	[Glycine max]	Glyma09g28310.1	ACA23207	22947	5.24	28	2	15	397
2	predicted protein	[Populus trichocarpa]	Glyma03g34820.1	XP_002327377	20260	6.13	26	2	15	212
3	(No significant hits to report)									
4	enolase	[Glycine max]	Glyma03g34830.1	AAS18240	47914	5.49	72	2	13	863
5	Heat shock 70 kDa protein	[Glycine max]	Glyma17g08020.1	P26413	71220	5.28	2152	25	38	1144
6	U2 small nuclear ribonucleoprotein A	[Ricinus communis]	Glyma20g18440.1	XP_002512712	32537	5.16	104	3	18	405
7	UDP-glucose 6-dehydrogenase	[Glycine max]	Glyma08g26520.1	Q96558	53478	5.74	66	2	14	936
8	dihydroxyacid dehydratase	[Glycine max]	Glyma13g27810.1	ACU26534	64644	5.76	314	23	25	1160
9	V- ATPase subnit A	[Glycine max]	Glyma08g23990.1	ABU87506	69020	5.48	281	47	26	1217
10	predicted protein	[Populus trichocarpa]	Glyma09g40690.1	XP_002323696	61059	5.51	1049	104	30	949
11	oligopeptidase A	[Medicago truncatula]	Glyma01g02480.1	ABY48141	88745	6.03	777	84	43	1227
12	Proteasome subnit alpha type-6	[Glycine max]	Glyma06g39710.1	O48551	27481	5.58	1406	107	47	489
13	glutathione reductase	[Vigna unguiculata]	Glyma16g27210.1	ABB89042	54243	5.63	629	79	48	880
14	hypothetical protein	[Vitis vinifera]	Glyma06g48360.1	XP_002272730	67578	5.60	765	89	39	1075
15	isoflavonoid malonyl transferase 2	[Medicago truncatula]	Glyma18g49240.1	ABY91222	56964	5.70	601	75	29	430
16	predicted protein	[Populus trichocarpa]	Glyma05g37670.1	XP_002298026	112704	5.89	69	9	15	1470
17	hypothetical protein	[Vitis vinifera]	Glyma18g04730.1	XP_002270157	82072	6.21	225	15	17	1216
18	UDP-D-apiose/xylose synthetase	[Gossypium hirsutum]	Glyma11g19550.1	ACJ11753	43680	5.83	283	47	27	728
19	hypothetical protein	[Vitis vinifera]	Glyma02g36530.1	XP_002276114	60039	6.69	70	7	16	854
20	isocitrate dehydrogenase [NADP]	[Glycine max]	Glyma02g40820.1	Q06197	46393	5.87	1474	124	40	828
21	UDP-glucose 6-dehydrogenase	[Glycine max]	Glyma08g26520.1	Q96558	53478	5.74	141	8	19	936
22	seed maturation protein PM31	[Glycine max]	Glyma02g08400.1	AAD30865	17907	6.10	57	2	19	323
23	elongation factor 1-gamma	[Glycine max]	Glyma16g00360.1	AAL82617	47785	5.92	117	2	15	619
24	nucleoside diphosphate kinase	[Glycine max]	Glyma03g25820.1	AAN77501	16308	6.30	76	2	12	284
25	cysteine proteinase inhibitor	[Glycine max]	Glyma13g25870.1	BAA19608	27708	6.57	57	5	10	417
26	Transaminase mtnE	[Ricinus communis]	Glyma08g06790.1	XP_002522052	50463	6.67	607	54	32	721
27	acetoacetyl-CoA thiolase	[Medicago sativa]	Glyma14g00760.1	ACX47470	41930	6.16	469	46	39	678
28	hypothetical protein isoform 2	[Vitis vinifera]	Glyma01g06970.1	XP_002269733	53522	6.34	448	44	38	890
29	acetoacetyl-CoA thiolase	[Medicago sativa]	Glyma14g00760.1	ACX47470	41930	6.16	62	2	13	678
30	Gamma-glutamyl hydrolase	[Glycine max]	Glyma13g34290.1	P93164	38282	6.72	3208	212	36	619
31	serine hydroxymethyltransferase 2	[Glycine max]	Glyma05g28490.	ACM45952	52142	6.90	982	67	25	920
32	heat shock protein 70	[Gossypium hirsutum]	Glyma19g35560.1	ACJ11741	71854	5.05	3704	251	37	1135
33	actin isoform PEAc14-1	[Pisum sativum]	Glyma02g10170.1	ADP09679	42036	5.23	682	107	51	754
34	glutamate decarboxylase	[Glycine max]	Glyma18g04940.1	BAF80896	57554	5.59	762	71	33	1011
35	predicted protein	[Populus trichocarpa]	Glyma36750.1	XP_002327728	99030	5.56	999	130	26	1624
36	hypothetical protein	[Vitis vinifera]	Glyma16g32960.1	XP_002267091	48231	6.06	1216	56	40	842
37	decarboxylase isozyme 1	[Glycine max]	Glyma13g30490.1	P51850	64169	5.73	626	97	35	1060
38	Ran3A-1	[Dimocarpus longan]	Glyma04g07350.1	AEM97804	25505	6.38	914	86	53	427
39	copper amino oxidase	[Glycine max]	Glyma17g02260.1	CAE47488	76061	6.21	696	121	32	1284
40	stem 28 kDa glycoprotein	[Glycine max]	Glyma07g01730.1	P15490	29218	8.75	1419	108	51	528
41	(No significant hits to report)									
42	alcohol dehydrogenase-1F	[Phaseolus acutifolius]	Glyma04g41990.1	Caa80691	41638	5.97	2015	183	53	734
43	methionine synthase	[Glycine max]	Glyma16g04240.1	AAQ08403	84401	5.93	2071	243	48	1419
44	hypothetical protein	[Vitis vinifera]	Glyma08g13440.1	XP_002270155	39049	5.12	2842	178	42	417
45	hypothetical protein	[Vitis vinifera]	Glyma13g41960.1	XP_002268097	35546	5.29	3702	207	58	560
46	cytosolic glutamine synthetase GSbeta1	[Glycine max]	Glyma11g33560.1	AAG24873	39138	5.48	262	20	27	718
47	heat shock 70 kDa protein	[Glycine max]	Glyma08g06950.1	Q01899	74981	6.02	3835	187	39	1157
48	lactoylglutathione lyase	[Gossypium hirsutum]	Glyma09g00660.1	ACJ11750	32504	5.76	645	70	31	531
49	glutamate-1-Semialdehyde2,1-aminomutase	[Glycine max]	Glyma04g00420.1	P45621	50239	6.05	168	7	12	874
50	predicted protein	[Populus trichocarpa]	Glyma02g13330.1	XP_002305394	45064	6.10	2165	185	40	685
51	dihydrolipoamide acetyltransferase	[Cucumis melo]	Glyma07g03930.1	ADN33731	59813	8.00	109	10	16	697
52	predicted protein	[Populus trichocarpa]	Glyma09g40690.1	XP_002323696	61059	5.51	2019	157	34	949
53	6-phosphogluconate dehydrogenase	[Glycine max]	Glyma18g51260.1	BAA22812	53903	5.68	699	93	27	927
54	glutathione reductase	[Vigna unguiculata]	Glyma16g27210.1	ABB89042	54243	5.63	1795	168	35	880
55	d-3-phosphoglycerate dehydrogenase	[Ricinus communis]	Glyma10g40750.1	XP_002518687	62859	6.32	41	2	14	878
56	(No significant hits to report)									
57	argininosuccinate synthase,putative	[Ricinus communis]	Glyma05g03190.1	XP_002521168	52641	6.55	16	2	13	774
58	pyruvate decarboxylase 1	[Lotus corniculatus]	Glyma18g43460.1	AAO72533	66371	5.80	273	46	19	1071
59	Chalcone-flavonone isomerase 1A	[Glycine max]	Glyma20g38560.1	Q93XE6	23307	6.23	2359	126	61	394
60	6-phosphogluconate dehydrogenase	[Glycine max]	Glyma08g28230.1	BAA22812	53811	6.11	1433	110	39	899
61	Os05g0553000	[Oryza sativa]	Glyma10g41330.1	NP_001056261	59913	5.80	39	2	15	879
62	predicted protein	[Populus trichocarpa]	Glyma11g03330.1	XP_002329902	65864	6.26	585	52	39	854
63	(No significant hits to report)									
64	quinine oxidoreductase	[Cicer arietinum]	Glyma13g32300.1	CAD31838	21653	6.43	1017	80	47	384
65	ald/keto reductase,putative	[Ricinus communis]	Glyma03g40680.1	XP_002512220	37793	6.26	201	22	33	510
66	Knitz trypsin protease inhibitor	[Glycine max]	Glyma09g28310.1	ACA23207	22947	5.24	405	37	33	397
67	peroxisomal betaine-aldehyde dehydrogenase	[Glycine max]	Glyma06g19820.1	BAG09377	55389	5.23	965	111	35	1015
68	ripening related protein	[Glycine max]	Glyma08g24760.1	AAD50376	17865	5.96	1338	195	79	263
69	peptidyl-prolyl cis-trans isomerase 1	[Glycine max]	Glyma11g10480.1	Q8W171	18441	8.70	1440	71	62	321
70	Os05g0553000	[Oryza sativa]	Glyma10g41330.1	NP_001056261	59913	5.80	3205	160	50	879
71	S-adenosylmethionine synthetase	[Cicer arietinum]	Glyma15g21890.1	ACL14491	43425	5.50	1599	98	51	745
72	(No significant hits to report)									
73	fructose-bisphosphate aldolase	[Glycine max]	Glyma02g38730	O65735	38469	7.12	2591	148	76	634

Spot no shows Spot number. Protein ID shows Phytozome protein ID. Accession no shows NCBI Accession number. MP shows matched peptides. Cov shows sequence coverage.

**Table 2:** Flooding-responsive proteins identified in soybean cotyledon using LC-MS/MS.

					Theoretical					
Spot no	Homologous protein	Species	Protein ID	Accession no	Mr (Da)	pI	Score	MP	Cov (%)	Blast Score
1	sucrose-binding protein	[Glycine max]	Glyma10g03390.1	Q04672	58353	6.08	22	3	17	723
2	(No significant hits to report)									
3	lactoylglutathione lyase	[Gossypium hirsutum]	Glyma09g00660.1	ACJ11750	32504	5.76	393	15	21	531
4	(No significant hits to report)									
5	glycinin G2	[Glycine max]	Glyma03g32020.1	P04405	54927	5.46	202	26	20	799
6	NADPH-protochlorophyllide oxidoreductase	[Vigna radiata]	Glyma06g38160.1	AAF89208	43247	9.12	132	15	19	714
7	(No significant hits to report)	(No significant hits to report)								
8	beta-conglycinin alpha prime subunit	[Glycine max]	Glyma10g39150.1	BAE02726	72469	5.50	105	19	15	893
9	beta-conglycinin alpha subunit	[Glycine max]	Glyma20g28650.1	BAE44299	70549	5.12	82	22	17	783
10	(No significant hits to report)									
11	beta-conglycinin alpha subunit	[Glycine max]	Glyma20g28650.1	BAE44299	70549	5.12	121	33	17	783
12	hypothetical protein	[Vitis vinifera]	Glyma04g35950.1	XP_002281671	91567	5.11	63	6	11	1446
13	predicted protein	[Populus trichocarpa]	Glyma01g05060.1	XP_002323767	48123	5.61	81	2	13	539
14	cytosolic malate dehydrogenase	[Glycine max]	Glyma02g00810.1	AAS18241	35787	5.91	140	7	13	631
15	heat shock 70 kDa protein	[Glycine max]	Glyma17g08020.1	P26413	71220	5.28	130	16	24	1144
16	sucrose-binding protein	[Glycine max]	Glyma10g03390.1	Q04672	58353	6.08	475	38	14	723
17	(No significant hits to report)									
18	(No significant hits to report)									
19	glyoxysomal isocitrate lyase isoform 1	[Glycine max]	Glyma06g45950.1	ABD28288	65144	7.26	30	2	12	1075
20	(No significant hits to report)									
21	ferrous iron transport protein b	[Ralstonia solanacearum]	Glyma02g08980.1	AEG70893	217645	5.32	21	2	10	22.7
22	glycinin G2[Glycine max]	[Glycine max]	Glyma03g32030.1	P04405	54927	5.46	382	23	19	799
23	sucrose-binding protein	[Glycine max]	Glyma10g03390.1	Q04672	58353	6.08	1359	102	36	723
24	glycinin G3	[Glycine max]	Glyma19g34780.1	P11828	54835	5.73	53	9	16	796
25	(No significant hits to report)									
26	sucrose-binding protein	[Glycine max]	Glyma10g03390.1	Q04672	58353	6.08	2101	142	54	723
27	beta-conglycinin alpha subunit	[Glycine max]	Glyma20g28650.1	BAE44299	70549	5.12	584	96	33	783
28	ferrous iron transport protein b	[Ralstonia solanacearum]	Glyma03g42290.1	AEG70893	217201	6.16	15	2	10	22.7
										

Spot no shows Spot number. Protein ID shows Phytozome protein ID. Accession no shows NCBI Accession number. MP shows matched peptides. Cov shows sequence coverage.

In the cotyledon, 7 proteins that increased in abundance at least 2-fold in response to flooding stress were identified: heat shock 70 kDa protein (HSP70) (spot number C15), glycinin G3 (spot number C24), sucrose-binding protein (spot number C26), beta-conglycinin alpha subunit (spot number C27), hypothetical protein (spot number C12), and 2 unknown proteins (spot number C2 and spot number C10). A total of 4 cotyledon proteins that decreased in abundance at least 2-fold in response to flooding stress were identified: glycinin G2 (spot number C22), sucrose-binding protein (spot number C23), beta-conglycinin alpha subunit (spot number C11), and ferrous iron transport protein b (spot number C21) (Figure 2) (Table 2).

### Flooding-stress-induced Changes in the Abundance of Proteins Common to the Root and Cotyledon of Soybean Plants

**Figure 3:**
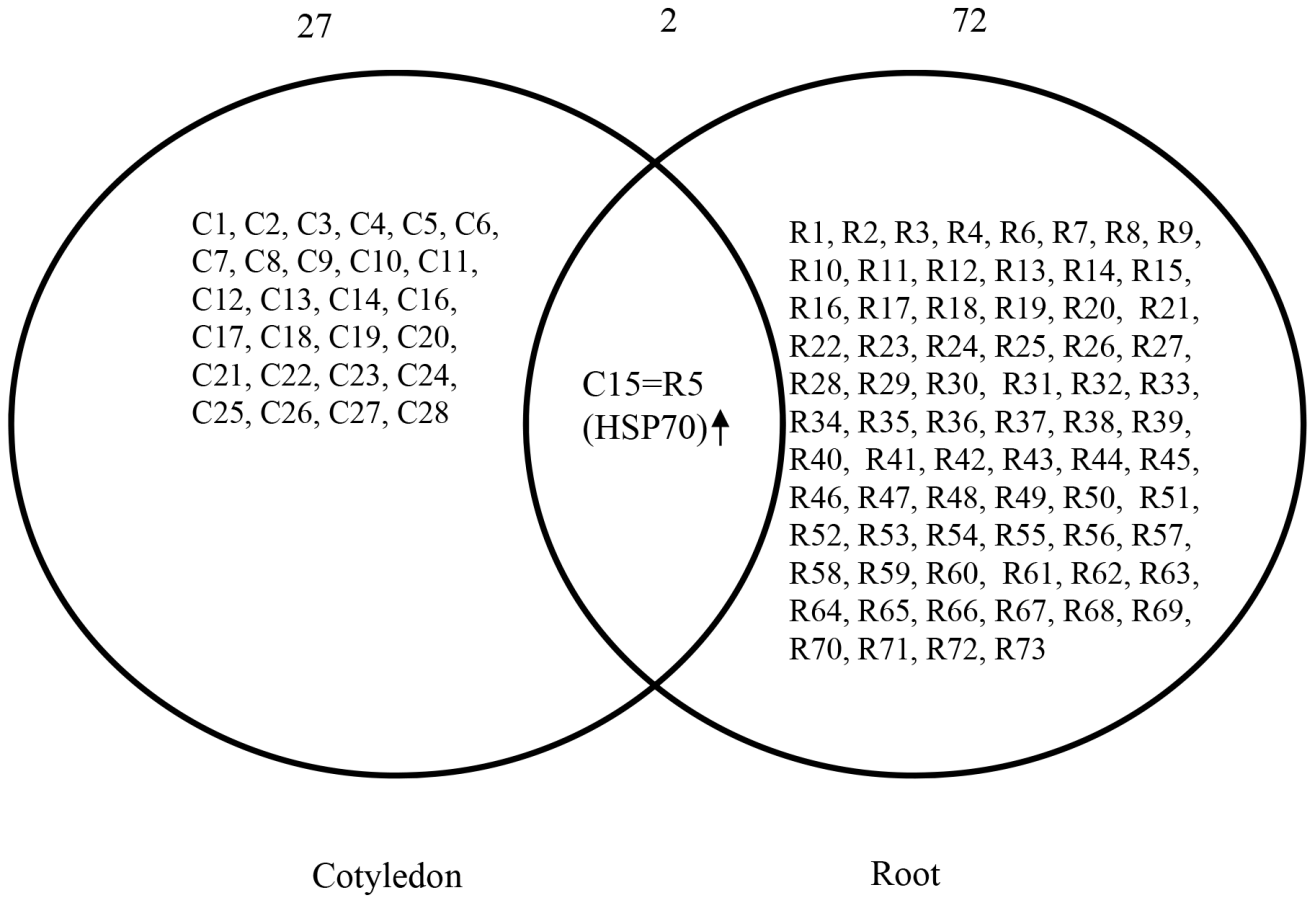
Venn diagram analysis of root and cotyledon proteins of flooding-stressed soybean plants. Diagram shows root and cotyledon proteins that changed in abundance in response to flooding stress. The overlapping area shows the common protein to both root and cotyledon that decreased in abundance in both organs. Numbers at the top of the diagram indicate the number of proteins. Downward arrow indicates a decrease in staining intensity. The numbers within the circles correspond to the proteins listed in Table 1 and Table 2.

Analysis of the 2-DE pattern of root and cotyledon proteins of flooding-stressed soybean plants showed that the abundance of only one protein common to both organs, HSP70 (Glyma17g08020.1) (spot numbers R5 and C15), increased in both the root and cotyledon following flooding stress (Figure 3) (Table S5). The ratio of protein abundance of HSP70 in the cotyledon was higher than that detected in the root under flooding stress. However, HSP70 with accession number Glyma19g35560.1 (spot numbers R32) and Glyma08g06950.1 (spot numbers R47) decreased in root under flooding stress. The remaining proteins showed organ-specific changes in response to flooding, indicating that each organ responds differently to flooding stress.

### Biophoton Emission in the Root and Cotyledon of Soybean Plants under Flooding Stress

**Figure 4:**
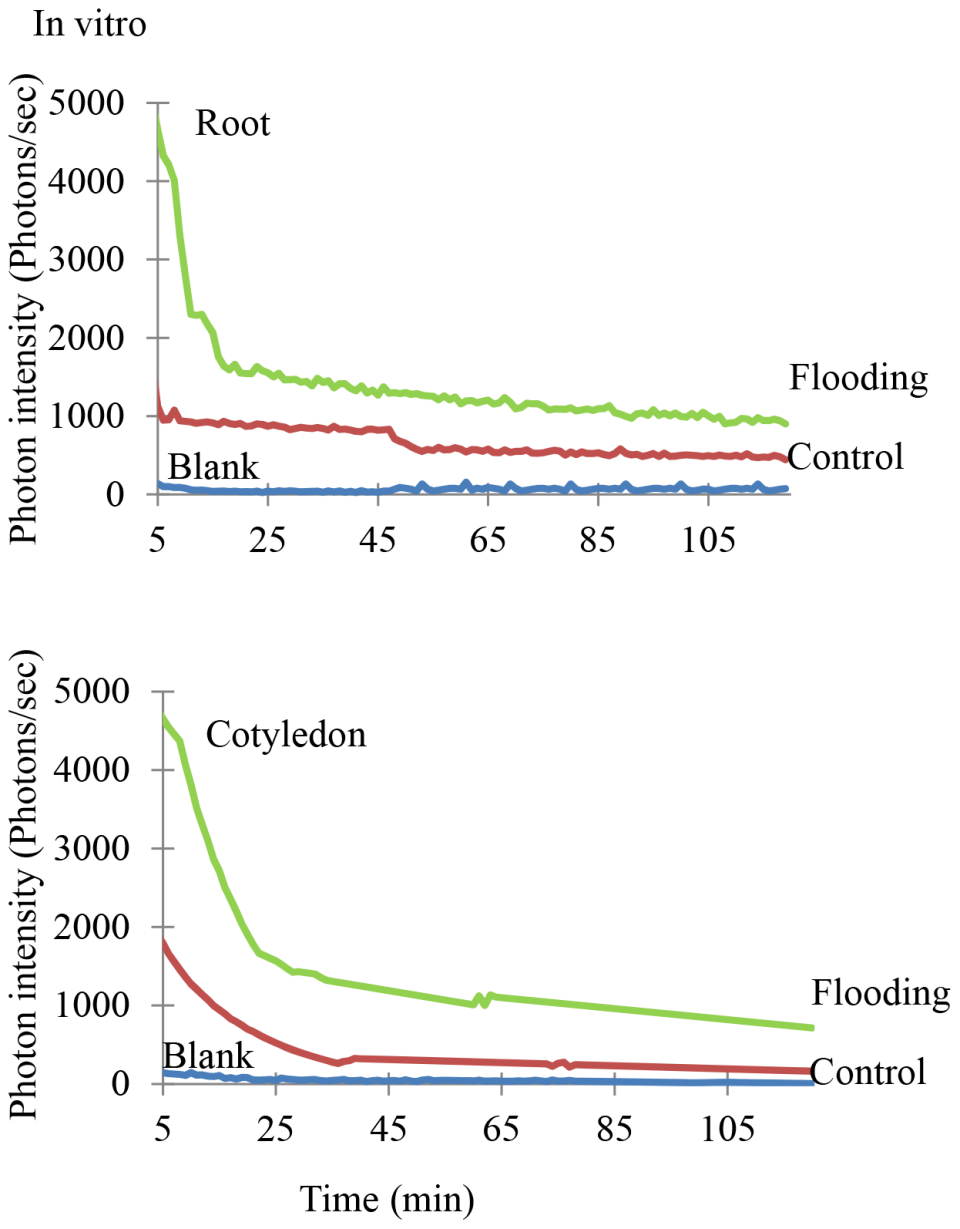
*In vitro* biophoton emission patterns of flooding-stressed soybean plants. Two-day-old soybean plants were flooded for 2 days, after which proteins were extracted from the root and cotyledon and H_2_O_2_ was added. Emission of photons from these extracts was then measured. Three independent biological replicate analyses of the root and cotyledon were performed (Figure S3). Ground photon emission by the empty Petri dish served as a blank (blue). Green lines represent biophoton emission from flooding-stressed soybean plants, while red lines represent biophoton emission from untreated soybean plants.

**Figure 5:**
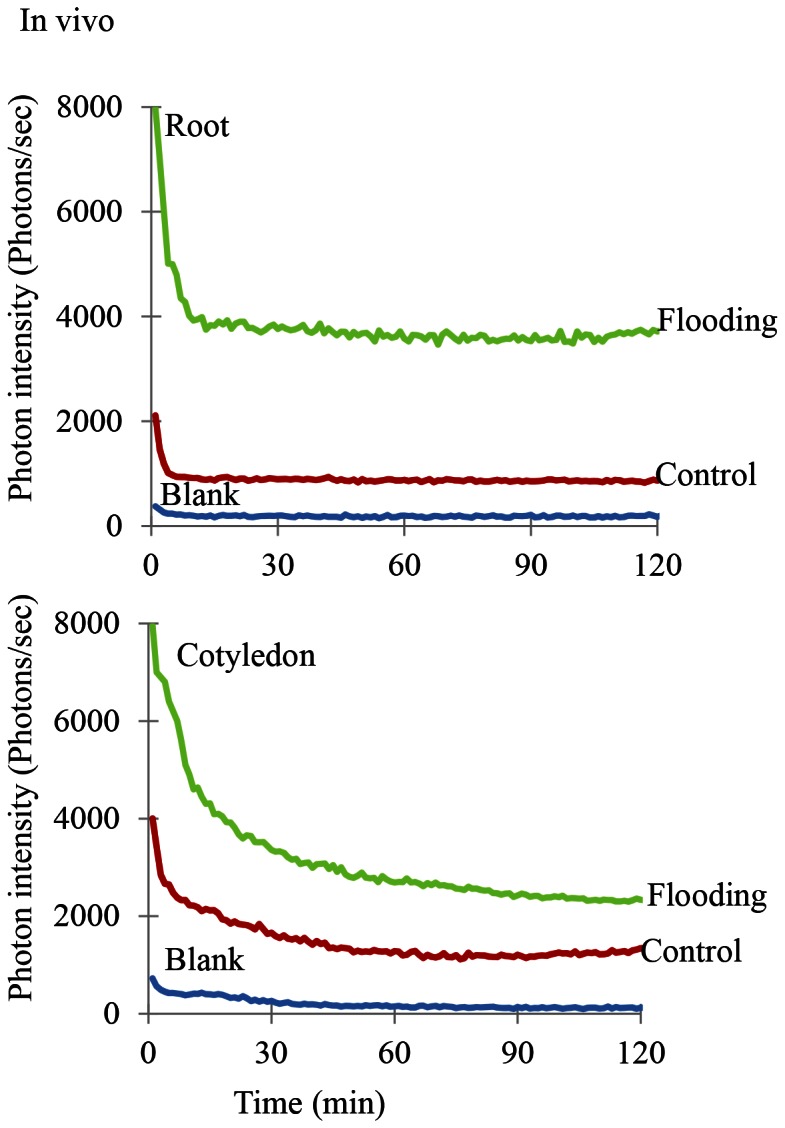
*In vivo* biophoton emission patterns of flooding-stressed soybean plants. Two-day-old soybean plants were flooded for 2 days, after which the root and cotyledon were treated with luminal solution and photon emission was measured. Three independent biological replicate analyses of the root and cotyledon were performed (Figure S4). Ground photon emission by the empty Petri dish served as a blank (blue). Green lines represent biophoton emission from flooding-stressed soybean plants, while red lines represent biophoton emission from untreated soybean plants.

To determine the effect of flooding stress on the physiological state of soybean plants, biophoton emission in the root and cotyledon was measured both *in vitro* and *in vivo* using a photon counter. Two-day-old soybean was flooded for 2 days and the results were compared to measurements of control soybean plants that had not been flooded. *In vitro* measurements showed that the rate of biophoton emission in both the root and cotyledon was higher in flooding-stressed plants than in control untreated plants (Figure 4) (Figure S3). Furthermore, the ratio of biophoton emission of both in vivo and in vitro in the cotyledon was higher than that detected in the root under flooding stress (Figure 4) (Figure 5).

For *in vivo* measurement of biophoton emission, luminal solution was applied to fresh samples which were then transferred to Petri plates and placed on the rotating disk of the photon counter. These analyses confirmed that the intensity of biophoton emission was also higher in both the root and cotyledon of flooding-stressed soybeans (Figure 5) (Figure S4).

### X-Ray Computed Tomography and Scanning Electron Microscopy/Energy Dispersive X-Ray Analysis

**Figure 6:**
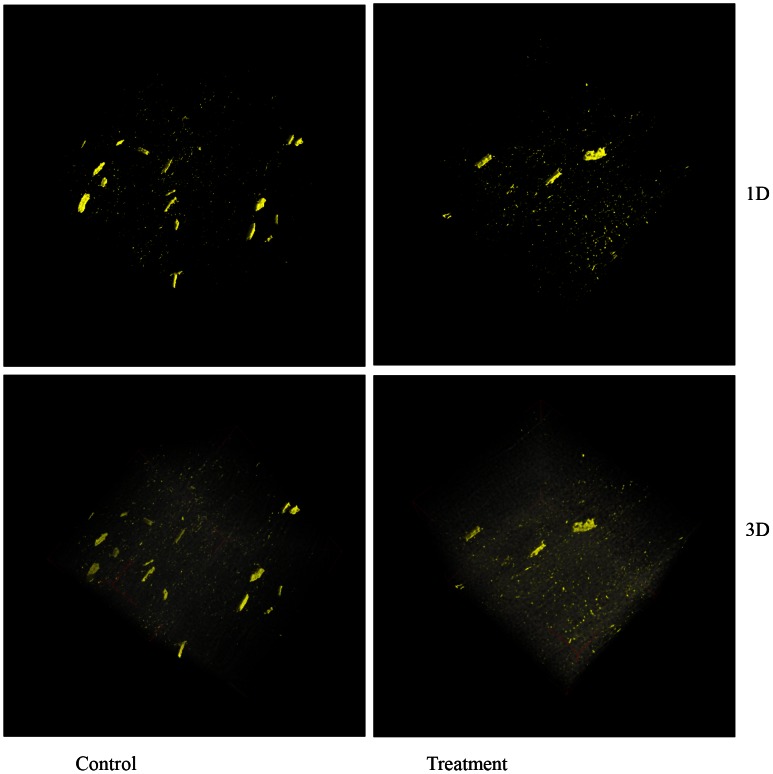
X-ray computed tomography analysis. Two-day-old soybean plants were flooded for 2 days, after which the cotyledon was collected and fixed. Untreated plants were used as control. Digital geometry processing was used to generate a three-dimensional image of the inside of the cotyledon from a large series of two-dimensional X-ray images taken around a single axis of rotation. The “1D” and “3D” show the first-dimension and three-dimension.

Two-day-old soybean plants were flooded for 2 days, and the cotyledon was collected and fixed. Changes in components in the cotyledon of flooding-stressed plants were analyzed using X-ray computed tomography (Figure 6) (Video S1) (Video S2). Flooding stress resulted in a decrease in the number of crystals in the cotyledon (Figure 6).

**Figure 7:**
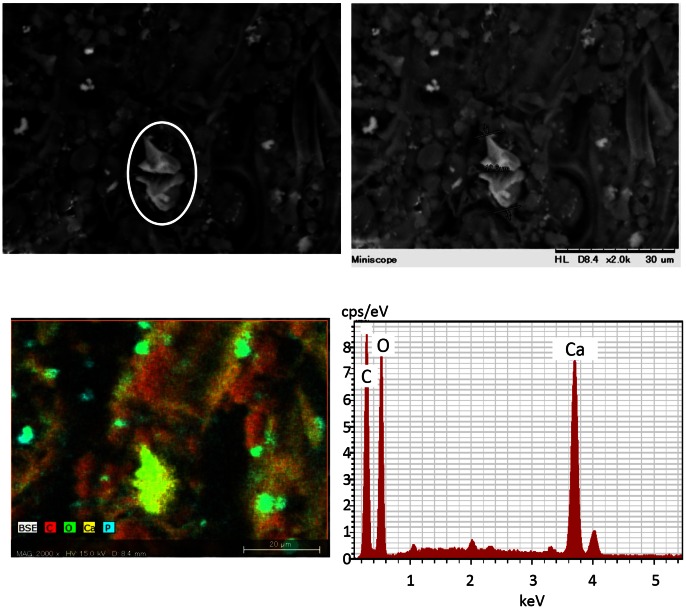
Scanning electron microscopy and energy dispersive X-ray analysis. Two-day-old soybean plants were flooded for 2 days, after which the cotyledon was collected and fixed. Scanning electron micrographs and energy dispersive X-ray spectra are shown.

Analysis of the cotyledon crystals using scanning electron microscopy and energy dispersive X-rays showed that the element of crystal contained the carbon (C), oxygen (O), and calcium (Ca); which indicated that it was composed of calcium oxalate, CaC_2_O_4_ (Figure 7) (Figure S5) (Figure S6). These results indicated that flooding stress leads to a decrease in the number of calcium oxalate crystals in the cotyledon.

## Discussion

Soybean plants are sensitive to flooding stress, and their growth is significantly reduced by flooding [4]. Root elongation of soybean clearly retarded by 2 days of flooding stress [5], [22]. Flooding-associated suppression of root elongation may occur in the root tip because this region contains the root apical meristem and is the site of elongation [8]. The root apical meristem is also important for root system development. Prolonged flooding of soybean seedlings has also been shown to induce the cell death of root tip [6], [7], [14], which is one of the symptoms of flooding injury in soybean seedlings. While a number of reports have described the flood response mechanisms of soybean root [4], there are no reports describing the changes that occur in the cotyledon in response to flooding. In this study, flooding-stress-induced changes in the soybean cotyledon were examined using biological and proteomic technique to obtain a further current understanding of the flooding-stress response in this important agricultural crop.

Changes in components in the cotyledon of soybeans under flooding stress were analyzed using X-ray computed tomography, and flooding stress resulted in a decrease in the number of crystals in the cotyledon (Figure 6). Furthermore, scanning electron microscopy and energy dispersive X-rays showed that it was composed of calcium oxalate (Figure 7). These results indicated that flooding stress leads to a decrease in the number of calcium oxalate crystals in the cotyledon. Calcium oxalate crystals modulate physiological calcium levels within plant tissues [23], [24]. Depending on the calcium requirement of the developing soybean seed, the number and distribution of calcium oxalate crystals change as the seed develops and matures [25]. Chiera and Grabau [26] provided evidence that D-myo-inositol-3-phosphate synthase is associated with oxalate crystal idioblasts. Furthermore, the cell death of cotyledon in flooding-stressed soybean plants has been documented [9].

The proteomic technique was used to examine whether calcium related proteins are involved in soybean under flooding stress. The proteomic analysis of the root and cotyledon showed that only one protein, HSP70, increased in abundance in both organs in response to flooding stress (Figure 3). HSP70 had a weak ATPase activity that was stimulated by interaction with DnaJ proteins [27]. Enhanced ATPase activity was required for stable binding and proper folding of HSP70’s client proteins [28]. HSP70 was involved in many cellular processes, including protein folding, protein translocation across membranes, and regulation of protein degradation. Cha et al. [29] reported that HSP70 bound to calmodulin-2 in the presence of calcium ion via a conserved calmodulin-binding domain. Suri and Dhindsa [30] reported that heat-induced accumulation of HSP70 required a heat-activated MAP kinase. Heat activation of MAP kinase was rapid and transient, and required an influx of calcium ion from the apoplast and the activity of an upstream MEK-related MAPKK. Interestingly, the ratio of protein abundance of HSP70 in the cotyledon was significantly higher than that detected in the root under flooding stress (Figure 1) (Figure 2). These observations and our result indicate that calcium might be one candidate of the factor that operates in soybean cotyledon when responding to the flooding stress.

In addition, biophoton emission was measured because calcium is related to photon emission. The intensity of photon emission was higher in the root and cotyledon of flood-stressed plants than in untreated soybean plants. Furthermore, the ratio of biophoton emission in the cotyledon was higher than that in the root under flooding stress (Figure 4) (Figure 5). Bennett et al. [31] demonstrated that ultra-weak photon emission or biophoton generation was associated with hypersensitive cell death. Biophoton emission required an intact R-signaling network and increased as levels of cytosolic calcium and nitric oxide rise, but elevated levels of reactive oxygen species were not necessary [31]. Subbaiah and Sachs [7] indicated that early rise in cytosolic calcium ion, as well as a quick establishment of ionic homeostasis, may be essential for the induction of adaptive changes at the cellular as well as organism-level. These reports and our result suggest that an increase in calcium level in the cotyledon might stimulate biophoton emission.

A role for calcium oxalate production is as part of a high-capacity mechanism for regulating bulk calcium levels in plant tissues and organs [24]. A decrease in calcium oxalate crystal suggests that calcium ions increase in soybean cotyledon under flooding stress. Decomposition of calcium oxalate crystals produces calcium ions that may be available for HSP70 signal transduction. Since flooding stress leads to a decrease in the amount of calcium oxalate crystals, which means an increase of calcium ion, HSP70 signal transduction might be stimulated. In conclusion, these results suggest that calcium ion supplied from calcium oxalate crystals might play one role for signal transduction through HSP70 in the cotyledon, and that a part of the passing way of flooding stress may induce HSP70-mediated signal transduction.

## Materials and Methods

### Plant Growth and Treatments

Seeds of soybean (*Glycine max* [L.] Merrill cultivar Enrei) were sterilized in a sodium hypochlorite solution and germinated on silica sand. Two-day-old soybean plants were flooded with water for 2 days in a growth chamber illuminated with white fluorescent light (200 µmol m^−2^s^−1^; 12-h light period/day) at 25°C and 70% relative humidity. To examine organ-specific responses, cotyledon and root were collected from the 4-day-old plants after 2 days of flooding. For all experiments, untreated equivalent plants were sampled as controls, and 3 independent experiments were performed as biological replicates.

### Protein Extraction for Two-Dimensional Polyacrylamide Gel Electrophoresis

A portion (500 mg) of fresh sample was ground to powder in liquid nitrogen with a mortar and pestle. The powder was transferred to 10% trichloroacetic acid and 0.07% 2-mercaptoethanol in acetone and the mixture was vortexed. The suspension was sonicated for 5 min and then incubated for 1 h at −20°C. After incubation, the suspension was centrifuged at 9,000 × *g* for 20 min at 4°C. The supernatant was discarded and resulting pellet was washed twice with 0.07% 2-mercaptoethanol in acetone. The resulting pellet was dried using a Speed-Vac concentrator (Savant Instruments, Hicksville, NY, USA) and resuspended by vortexing for 1 h at 25°C in a solution of 8 M urea, 2 M thiourea, 5% CHAPS, and 2 mM tributylphosphine. The resulting suspension was centrifuged at 20,000 × *g* for 20 min at 25°C and the supernatant was collected for two-dimensional polyacrylamide gel electrophoresis (2-DE). Protein concentrations were determined using the Bradford method [32] with bovine serum albumin as the standard.

### Two-Dimensional Polyacrylamide Gel Electrophoresis

Protein samples (500 µg) in a final volume of 180 µL of lysis buffer containing 0.4% ampholytes pH 3–10 (Bio-Lyte, Bio-Rad, Hercules, CA, USA) were loaded into a focusing tray. Immobilized pH gradient strips (3–10 NL, 11 cm, Bio-Rad) were passively rehydrated for 2.5 h and then actively rehydrated for 14 h at 50 V. Isoelectric focusing (IEF) was carried out using a Protean IEF Cell system (Bio-Rad) under the following conditions: 250 V for 15 min with a linear ramp, 8,000 V for 1 h with a linear ramp, and finally 8,000 V for 35,000 V-h with a rapid ramp. After IEF, the strips were incubated for 30 min in equilibration buffer I containing 6 M urea, 2% SDS, 0.375 M Tris-HCl (pH 8.8), 20% glycerol, and 130 mM dithiothreitol. The strips were then incubated for 30 min in equilibration buffer II containing 6 M urea, 2% SDS, 0.375 M Tris-HCl (pH 8.8), 20% glycerol, and 135 mM iodoacetamide. The equilibrated strips were then placed onto 15% SDS-PAGE gels and sealed with 1% low-melting-temperature agarose. Second dimension electrophoresis was performed at a constant current of 30 mA. After electrophoresis, the gels were stained for 1 h with Coomassie brilliant blue (CBB) (Phast Gel™ Blue R, GE Healthcare, Piscataway, NJ, USA) in 35% methanol and 10% acetic acid, and then destained for 12 h in 35% methanol and 10% acetic acid. The images were analyzed as described below.

### Gel Image Analysis

CBB-stained gels were scanned using a high-resolution scanner (GS-800 Calibrated Imaging Densitometer, Bio-Rad). Protein spots were detected and quantified on the basis of relative intensity using PDQuest software (version 8.0.1, Bio-Rad). The intensity of a given protein spot was expressed in terms of its volume, which was defined as the sum of the intensities of all pixels constituting the spot in the image. To compensate for subtle differences in sample loading, gel staining, and gel destaining, the volume of each spot was normalized as a percentage of the total volume of all the spots present in the gel. Manual editing was carried out after automated detection and matching.

Student’s *t* test was used to assess the statistical significance of differences in protein abundance between control and treatment samples. Proteins exhibiting a fold change of more than 2.0 and a *P*-value <0.05 relative to the control were considered to be significantly altered in abundance.

### Peptide Preparation for Mass Spectrometry Analysis

Proteins were identified using mass spectrometry (MS). Protein spots were excised from 2-DE gels and washed with water. Proteins in the excised gel pieces were then reduced by incubating them for 1 h at 60°C in 100 mM NH_4_HCO_3_ buffer containing 10 mM dithiothreitol followed by incubation for 30 min in 100 mM NH_4_HCO_3_ buffer containing 40 mM iodoacetamide. The gel pieces were digested at 37°C overnight in 100 mM NH_4_HCO_3_ containing 1 pM trypsin (Wako, Osaka, Japan). The tryptic peptides were extracted from the gel grains 3 times using 0.1% trifluoroacetic acid in 50% acetonitrile. The procedure described above was performed with DigestPro (Intavis Bioanalytical Instruments AG, Cologne, Germany). The resulting peptide solutions were desalted with C-Tip pipet tips (Nikkyo Technos, Tokyo, Japan) and analyzed by nano-liquid chromatography (LC)-tandem mass spectrometry (LC)-MS/MS.

### Protein Identification by Mass Spectrometry

Peptides in 0.1% formic acid were loaded onto a C18 PepMap trap column (300-µm ID × 5 mm) and eluted using an Ultimate 3000 nanoLC system (Dionex, Germering, Germany). The peptides were separated on a nano-capillary column (NTTC-360/75-3, Nikkyo Technos) with 0.1% formic acid in acetonitrile at a flow rate of 200 nL/min and introduced with a spray voltage of 1.8 kV into a nanospray LTQ XL Orbitrap MS (Thermo Fisher Science, San Jose, CA, USA) operated in data-dependent acquisition mode with the installed XCalibur software. Full-scan mass spectra were acquired over the *m/z* range 150–2,000 with a resolution of 15,000. The 3 most intense ions above the 1,000 threshold were selected for collision-induced fragmentation in the linear ion trap at a normalized collision energy of 35% after accumulation to a target value of 1,000. Dynamic exclusion was employed within 30 s to prevent repetitive selection of peptides.

Acquired MS/MS spectra were converted to individual DTA files using BioWorks software (version 3.3.1) (Thermo Fisher Science). The following parameters were used to create peak lists: parent ions in the mass range with no limitation, 1 grouping of MS/MS scans, and threshold of 100. The resulting peptide mass data were used to search the database using the MASCOT search engine (Matrix Science, London, UK). Soybean genome sequences were downloaded from the soybean genome database [33] (Phytozome, version 6.0, http://www.phytozome.net/soybean) and converted to FASTA format. Carbamidomethylation of cysteines was set as a fixed modification and oxidation of methionine was set as a variable modification. Trypsin was specified as the proteolytic enzyme and 1 missed cleavage was allowed. The search parameters were peptide mass tolerance = 10 ppm, fragment mass tolerance = 0.2 Da, maximum missed cleavages = 1, and peptide charges = +1, +2, and +3. The minimal requirements for accepting protein identifications were as follows: (i) the score, indicating the probability of a true positive identification, must be at least 100, (ii) there must be at least 2 peptide sequence matches above the identity threshold, and (iii) the coverage of the protein sequence by the matching peptides must be at least 7%. Positive matches were BLASTP searched against the NCBI protein database for updated annotation and identification of homologous proteins.

### Measurement of Biophoton Emission

For *in vitro* measurement of biophoton emission, a portion (250 mg) of fresh sample was homogenized with a mortar and pestle in 2.5 mL of 25 mM potassium phosphate buffer (pH 7.8) containing 2% polyvinylpolypyrrolidone, 0.4 mM EDTA, and 1 mM ascorbic acid. After centrifugation at 15,000 × *g* for 20 min at 4°C, the supernatant was collected as protein extract. The solution used for the measurement of biophoton emission consisted of 880 µL of reaction mixture, 100 µL of protein extract, and 20 µL of 0.056% H_2_O_2_. The reaction mixture was composed of 50 mM phosphate buffer (pH 7.8), 0.5 mM sodium ascorbate, and 0.1 mM EDTA. For *in vivo* measurement of biophoton emission, the fresh samples were treated directly with 0.5 mM luminal solution in phosphate buffer.

In order to observe the time-dependent variation in photon emission intensity, a C1230 photon counter (Hamamatsu Photonics, Hamamatsu, Japan) with a built-in high-voltage stabilized direct-current power source was used. The device counts the number of photons detected by a R208 photomultiplier tube (Hamamatsu Photonics) with a bi-alkali photocathode, providing a spectral response from 185–650 nm [19] Biophoton emission was measured in the root, hypocotyl, and leaf at 1-min intervals for 2 h at 25°C. Ground photon emission by the empty Petri dish served as a blank. Each experiment involved 3 independent biological replicates.

### X-Ray Computed Tomography

The cotyledons were excised from soybean plants and fixed in 4% paraformaldehyde (TAAB, Berks, UK) in 0.1 M phosphate buffer (pH 7.4) for overnight at 4°C. The cotyledons thus fixed were immersed in a solution of 100 mM Tris-HCl (pH 8.0) for 60 min at room temperature, followed by washing and dehydration with 100% ethanol. The dehydrated cotyledons were subjected to X-ray computed tomography analysis using a TOHKEN-SkyScan2011 scanner (MARS TOHKEN X-RAY INSPECTION Co. Ltd., Tokyo, Japan). The computed tomography was performed under the following conditions: X-ray emission with 200 µA at 20 kV, resolution of 360 nm/pixel, and slice thickness of 360 *n*m. Photographing of each of the 900 slices was done using a CCD camera with an exposure time of 8 s. Digital geometry processing is used to generate a three-dimensional image of the inside of an object from a large series of two-dimensional X-ray images taken around a single axis of rotation (Tsukuba GeneTech Lab,Tsukuba, Japan).

### Scanning Electron Microscopy and Energy Dispersive X-Ray Analysis

The cotyledons were excised from soybean plants and fixed in 4% paraformaldehyde (TAAB, Berks, UK) in 0.1 M phosphate buffer (pH 7.4) for overnight at 4°C. The cotyledons thus fixed were immersed in a solution of 100 mM Tris-HCl (pH 8.0) for 60 min at room temperature, followed by washing and dehydration with 100% ethanol. Composite membrane morphology was analyzed using a scanning electron microscopy, Hitachi TM3000 equipped with Quantax70 (Hitachi, Tokyo, Japan), operated with an accelerating voltage of 15 kV. Energy dispersive X-ray spectra of composite membranes were obtained with the SEM at 15 kV, and used for elemental analysis of the surface of samples.

## Supporting Information

Figure S1 2-DE patterns in the root proteins of flooding-stressed soybean plants. Two-day-old soybean plants were flooded for 2 days, after which proteins were extracted from the root, separated by 2-DE, and the gels were stained with CBB. Results are presented as the mean ± SE of relative protein intensity for gels from 3 biological replicates. 2-DE patterns were shown 3 biological replicates.
(TIF)

Figure S2 2-DE patterns in the cotyledon proteins of flooding-stressed soybean plants. Two-day-old soybean plants were flooded for 2 days, after which proteins were extracted from the cotyledon, separated by 2-DE, and the gels were stained with CBB. Results are presented as the mean ± SE of relative protein intensity for gels from 3 biological replicates. 2-DE patterns were shown 3 biological replicates.
(TIF)

Figure S3 *In vitro* biophoton emission patterns of flooding-stressed soybean plants. Two-day-old soybean plants were flooded for 2 days, after which proteins were extracted from the root and cotyledon and H_2_O_2_ was added. Emission of photons from these extracts was then measured. R1, R2, and R3 represent 3 independent biological replicate analyses of the root and cotyledon. Ground photon emission by the empty Petri dish served as a blank (blue). Sky blue and purple lines represent biophoton emission from flooded soybean plants, while yellow and pink lines represent biophoton emission from untreated soybean plants.
(TIF)

Figure S4 *In vivo* biophoton emission patterns of flooding-stressed soybean plants. Two-day-old soybean plants were flooded for 2 days, after which the root and cotyledon were treated with luminal solution and photon emission was measured. R1, R2, and R3 represent biophoton emission from 3 independent biological replicate analyses of the root and cotyledon. Ground photon emission by the empty Petri dish served as a blank (blue). Sky blue and purple lines represent biophoton emission from flooded soybean plants, while yellow and pink lines represent biophoton emission from untreated soybean plants.
(TIF)

Figure S5 Scanning electron microscopy and energy dispersive X-ray analysis of Ca, O, and C. Two-day-old soybean plants were flooded for 2 days, after which the cotyledon was collected and fixed. A scanning electron micrograph and energy dispersive X-ray spectra of Ca, O, and C are shown.
(TIF)

Figure S6 Scanning electron microscopy and energy dispersive X-ray analysis of F, K, Na, P, and S. Two-day-old soybean plants were flooded for 2 days, after which the cotyledon was collected and fixed. A scanning electron micrograph and energy dispersive X-ray spectra of F, K, Na, P, and S are shown.
(TIF)

Table S1 The results of Student’s *t* test for each spot in 2-DE pattern of root proteins.
(XLSX)

Table S2 The results of Student’s *t* test for each spot in 2-DE pattern of cotyledon proteins.
(XLSX)

Table S3 Peak lists for proteins identified in the root of flooding-stressed soybean plants.
(DOCX)

Table S4 Peak lists for proteins identified in the cotyledon of flooding-stressed soybean plants.
(DOCX)

Table S5 Common proteins to both cotyledon and root of flooding-stressed soybean plants.
(DOCX)

Video S1 Video detailing X-ray computed tomography analysis of the cotyledon of a 4-day-old soybean plant (control).
(ZIP)

Video S2 Video detailing X-ray computed tomography analysis of the cotyledon of a 4-day-old soybean plant after 2 days of flooding.
(ZIP)
