# Supplementary figures and images for: Proteomic and Biochemical Analyses of the Cotyledon and Root of Flooding-Stressed Soybean Plants
Source: PLoS One. 2013 Jun 14;8(6):e65301. doi: 10.1371/journal.pone.0065301 (PMC3683008; doi:10.1371/journal.pone.0065301)

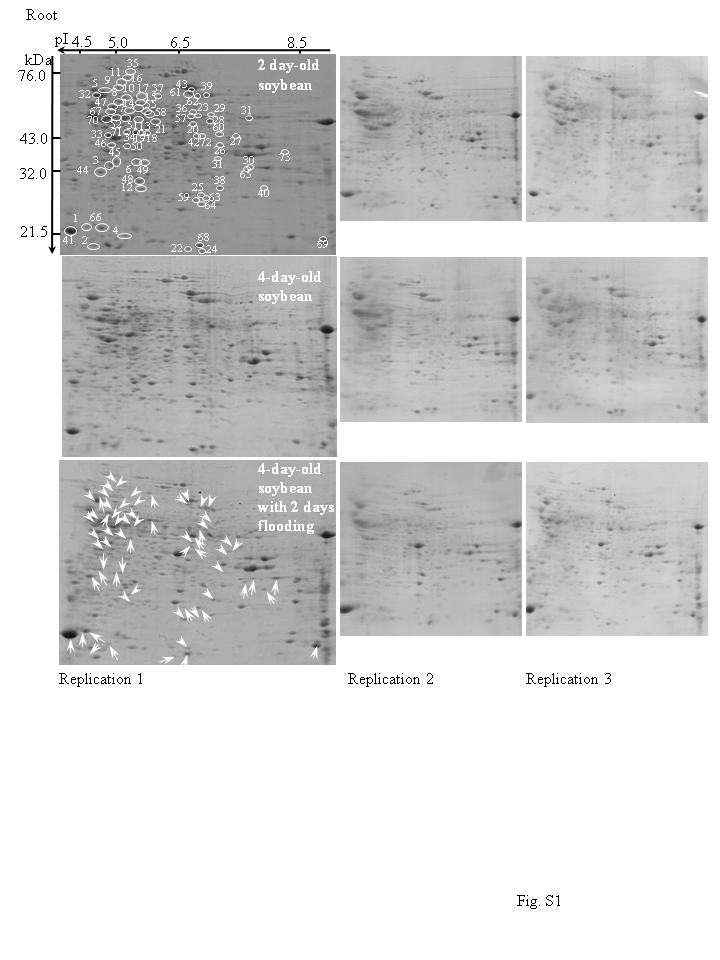

Supplement: Figure S1 — 2-DE patterns in the root proteins of flooding-stressed soybean plants. Two-day-old soybean plants were flooded for 2 days, after which proteins were extracted from the root, separated by 2-DE, and the gels were stained with CBB. Results are presented as the mean ± SE of relative protein intensity for gels from 3 biological replicates. 2-DE patterns were shown 3 biological replicates. (TIF) [file pone.0065301.s001.tif]

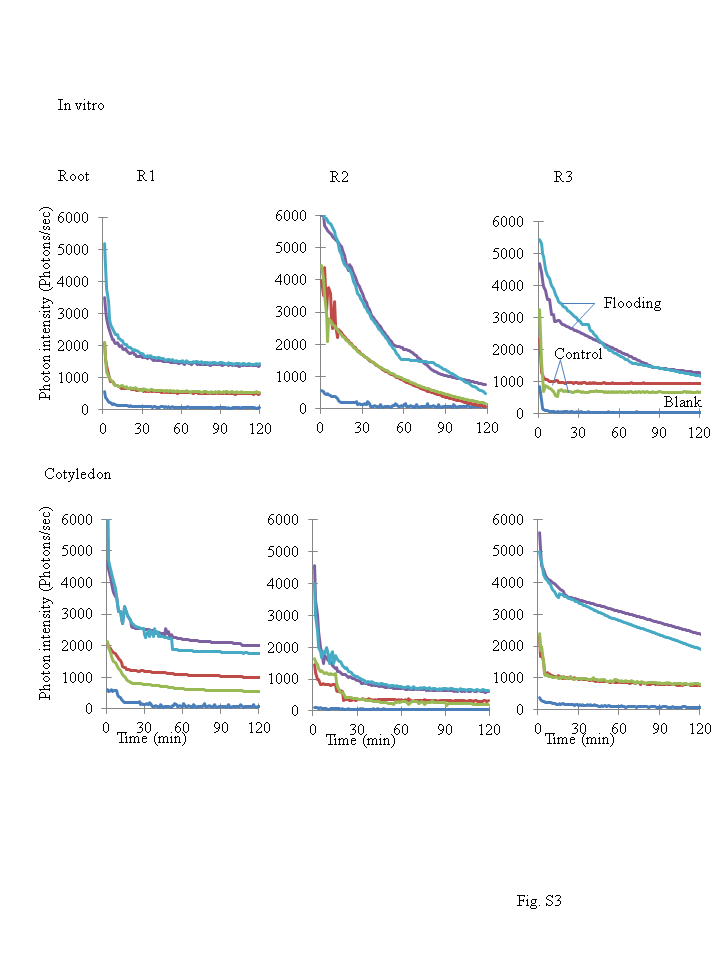

Supplement: Figure S3 — In vitro biophoton emission patterns of flooding-stressed soybean plants. Two-day-old soybean plants were flooded for 2 days, after which proteins were extracted from the root and cotyledon and H2O2 was added. Emission of photons from these extracts was then measured. R1, R2, and R3 represent 3 independent biological replicate analyses of the root and cotyledon. Ground photon emission by the empty Petri dish served as a blank (blue). Sky blue and purple lines represent biophoton emission from flooded soybean plants, while yellow and pink lines represent biophoton emission from untreated soybean plants. (TIF) [file pone.0065301.s003.tif]

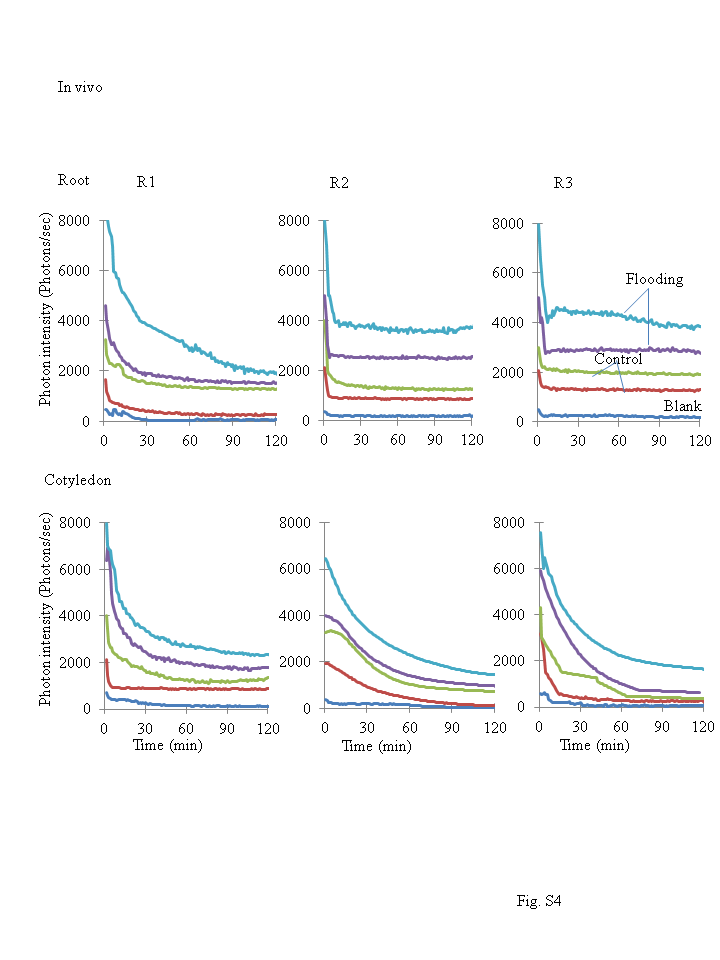

Supplement: Figure S4 — In vivo biophoton emission patterns of flooding-stressed soybean plants. Two-day-old soybean plants were flooded for 2 days, after which the root and cotyledon were treated with luminal solution and photon emission was measured. R1, R2, and R3 represent biophoton emission from 3 independent biological replicate analyses of the root and cotyledon. Ground photon emission by the empty Petri dish served as a blank (blue). Sky blue and purple lines represent biophoton emission from flooded soybean plants, while yellow and pink lines represent biophoton emission from untreated soybean plants. (TIF) [file pone.0065301.s004.tif]

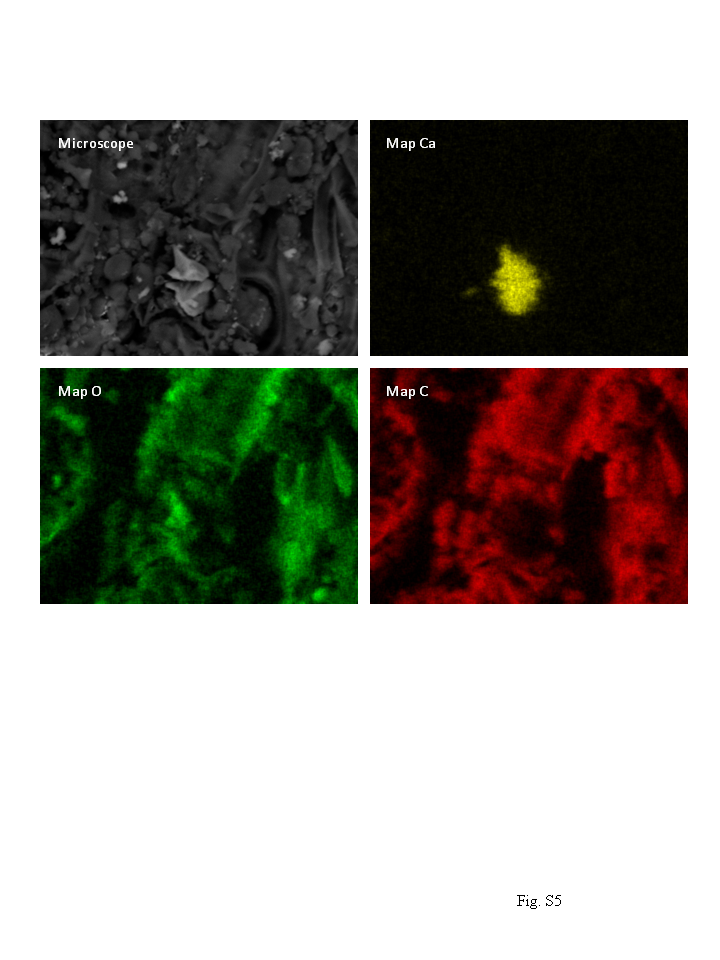

Supplement: Figure S5 — Scanning electron microscopy and energy dispersive X-ray analysis of Ca, O, and C. Two-day-old soybean plants were flooded for 2 days, after which the cotyledon was collected and fixed. A scanning electron micrograph and energy dispersive X-ray spectra of Ca, O, and C are shown. (TIF) [file pone.0065301.s005.tif]

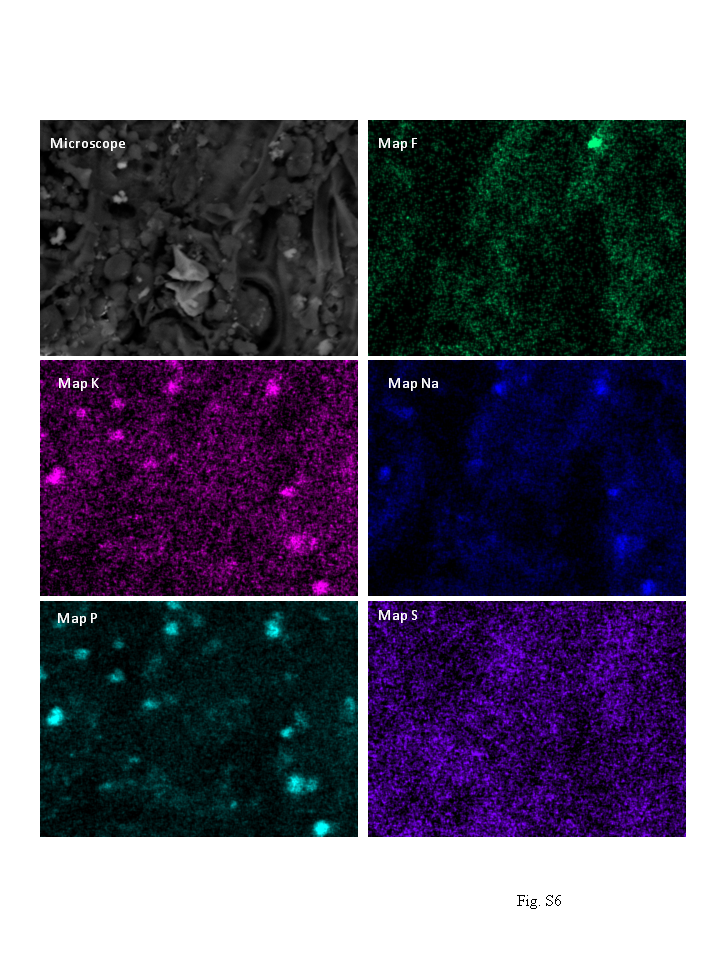

Supplement: Figure S6 — Scanning electron microscopy and energy dispersive X-ray analysis of F, K, Na, P, and S. Two-day-old soybean plants were flooded for 2 days, after which the cotyledon was collected and fixed. A scanning electron micrograph and energy dispersive X-ray spectra of F, K, Na, P, and S are shown. (TIF) [file pone.0065301.s006.tif]
