# Supplementary material for: Proteomic and Biochemical Analyses of the Cotyledon and Root of Flooding-Stressed Soybean Plants
Source: PLoS One. 2013 Jun 14;8(6):e65301. doi: 10.1371/journal.pone.0065301 (PMC3683008; doi:10.1371/journal.pone.0065301)
Supplement: Table S3 — Peak lists for proteins identified in the root of flooding-stressed soybean plants. (DOCX) [file pone.0065301.s009.docx]

| Table S3. Peak list of the identified proteins from root of soybean under flooding stress. | | | | | | | | | | | | | | | |
| --- | --- | --- | --- | --- | --- | --- | --- | --- | --- | --- | --- | --- | --- | --- | --- |
| Spot no. | Homologous protein | Accession no. | Score | | M.P. | Cov. (%) | | Blast score | | Mr (kDa) / pI | | | | Ratio | |
|  |  |  |  |  |  |  |  |  |  | Theo. | | | Exp. |  |  |
| 1a | phosphatidylethanolamine binding protein | Glyma01g30670.1 | 298 | | 8 | 36 | | 246 | | 18.8/4.92 | | |  |  | |
|  | Calc. Mass | Observ. Mass | Da | | ppm | start | | end | | sequence | | | | | |
|  | 1147.587 | 1147.587 | 0 | | 0 | 11 | | 21 | | LVSPAFSNEGK | | | | | |
|  | 1147.587 | 1147.589 | 0 | | 1 | 11 | | 21 | | LVSPAFSNEGK | | | | | |
|  | 1513.825 | 1513.821 | 0 | | -3 | 11 | | 24 | | LVSPAFSNEGKLPR | | | | | |
|  | 1513.825 | 1513.823 | 0 | | -2 | 11 | | 24 | | LVSPAFSNEGKLPR | | | | | |
|  | 1513.825 | 1513.824 | 0 | | -1 | 11 | | 24 | | LVSPAFSNEGKLPR | | | | | |
|  | 1513.825 | 1513.824 | 0 | | -1 | 11 | | 24 | | LVSPAFSNEGKLPR | | | | | |
|  | 1513.825 | 1513.826 | 0 | | 1 | 11 | | 24 | | LVSPAFSNEGKLPR | | | | | |
|  | 1985.026 | 1985.027 | 0 | | 1 | 35 | | 51 | | KNISPPLEWYNLPEGTK | | | | | |
|  | 1985.026 | 1985.027 | 0 | | 1 | 35 | | 51 | | KNISPPLEWYNLPEGTK | | | | | |
|  | 1856.931 | 1856.927 | 0 | | -2 | 36 | | 51 | | NISPPLEWYNLPEGTK | | | | | |
|  | 1856.931 | 1856.93 | 0 | | -1 | 36 | | 51 | | NISPPLEWYNLPEGTK | | | | | |
|  | 1225.565 | 1225.564 | 0 | | -1 | 95 | | 106 | | AAEMGGDYATIK | | | | | |
|  | 1241.56 | 1241.56 | 0 | | 0 | 95 | | 106 | | AAEMGGDYATIK | | | | | |
|  | 2011.916 | 2011.914 | 0 | | -1 | 95 | | 113 | | AAEMGGDYATIKEGNNDLK | | | | | |
|  | 1078.555 | 1078.553 | 0 | | -2 | 133 | | 141 | | LYALDDELK | | | | | |
|  | 1078.555 | 1078.554 | 0 | | -1 | 133 | | 141 | | LYALDDELK | | | | | |
|  | 1078.555 | 1078.554 | 0 | | -1 | 133 | | 141 | | LYALDDELK | | | | | |
|  | 1078.555 | 1078.554 | 0 | | 0 | 133 | | 141 | | LYALDDELK | | | | | |
|  | 1078.555 | 1078.554 | 0 | | 0 | 133 | | 141 | | LYALDDELK | | | | | |
|  | 1078.555 | 1078.555 | 0 | | 0 | 133 | | 141 | | LYALDDELK | | | | | |
|  | 1078.555 | 1078.555 | 0 | | 1 | 133 | | 141 | | LYALDDELK | | | | | |
|  | 1490.798 | 1490.797 | 0 | | -1 | 133 | | 145 | | LYALDDELKLGNK | | | | | |
|  | 1490.798 | 1490.798 | 0 | | 0 | 133 | | 145 | | LYALDDELKLGNK | | | | | |
| Spot no. | Homologous protein | Accession no. | Score | | M.P. | Cov. (%) | | Blast score | | Mr (kDa) / pI | | | | Ratio | |
|  |  |  |  |  |  |  |  |  |  | Theo. | | | Exp. |  |  |
| 1b | Kunitz trypsin protease inhibitor-like precursor | Glyma16g33710.1 | 282 | | 5 | 36 | | 450 | | 24.1/5.17 | | |  |  | |
|  | Calc. Mass | Observ. Mass | Da | | ppm | start | | end | | sequence | | | | | |
|  | 1020.5426 | 1020.542 | 0 | | 0 | 69 | | 76 | | TCPLYVIR | | | | | |
|  | 2099.98 | 2099.977 | 0 | | -1 | 122 | | 141 | | VGSGFWFVSTSGDPNDITSK | | | | | |
|  | 1570.7303 | 1570.728 | 0 | | -2 | 147 | | 159 | | LEGDHAYEIYSFK | | | | | |
|  | 1570.7303 | 1570.729 | 0 | | -1 | 147 | | 159 | | LEGDHAYEIYSFK | | | | | |
|  | 2395.0825 | 2395.079 | 0 | | -1 | 160 | | 182 | | FCPSVPGALCAPVGTFEDADGTK | | | | | |
|  | 1625.7759 | 1625.773 | 0 | | -2 | 183 | | 196 | | VMAVGDDIEPYYVR | | | | | |
|  | 1625.7759 | 1625.784 | 0 | | 5 | 183 | | 196 | | VMAVGDDIEPYYVR | | | | | |
|  | 1641.7709 | 1641.769 | 0 | | -1 | 183 | | 196 | | VMAVGDDIEPYYVR | | | | | |
|  | 1641.7709 | 1641.769 | 0 | | -1 | 183 | | 196 | | VMAVGDDIEPYYVR | | | | | |
| Spot no. | Homologous protein | Accession no. | Score | M.P. | | | Cov. (%) | | Blast score | | Mr (kDa) / pI | | | | Ratio |
|  |  |  |  |  |  |  |  |  |  |  | Theo. | Exp. | | |  |
| 1c | lipoxygease L-4 | Glyma13g42330.1 | 267 | 6 | | | 10 | | 1650 | | 96.8/5.76 |  | | |  |
|  | Calc. Mass | Observ. Mass | Da | ppm | | | start | | end | | sequence | | | | |
|  | 2135.1303 | 2135.131 | 0 | 1 | | | 156 | | 174 | | IFFANNTYLPSETPAPLLK | | | | |
|  | 2061.8803 | 2061.875 | 0 | -2 | | | 197 | | 214 | | IYDYDVYNDLGNPDSGDK | | | | |
|  | 849.5688 | 849.5684 | 0 | 0 | | | 320 | | 327 | | IAPIPVIK | | | | |
|  | 1267.7136 | 1267.711 | 0 | -2 | | | 362 | | 373 | | ETIAGLNPNVIK | | | | |
|  | 1161.6281 | 1161.627 | 0 | -1 | | | 374 | | 383 | | IIEEFPLSSK | | | | |

| Spot no. | Homologous protein | Accession no. | Score | M.P. | Cov. (%) | | Blast score | | Mr (kDa) / pI | | Ratio |
| --- | --- | --- | --- | --- | --- | --- | --- | --- | --- | --- | --- |
|  |  |  |  |  |  |  |  |  | Theo. | Exp. |  |
| 1d | enolase | Glyma03g34830.1 | 198 | 3 | 12 | | 863 | | 48.1/5.49 |  |  |
|  | Calc. Mass | Observ. Mass | Da | ppm | start | | end | | sequence | | |
|  | 1937.8789 | 1937.877 | 0 | -1 | 17 | 34 | | GNPTVEVDLTCSDGTFAR | | | |
|  | 1600.842 | 1600.842 | 0 | 0 | 353 | 367 | | VNQIGSVTESIEAVR | | | |
|  | 2251.122 | 2251.121 | 0 | 0 | 382 | 403 | | SGETEDTFIADLSVGLATGQIK | | | |

| Spot no. | | | Homologous protein | | | | | | | | | | | | | | | | | | Accession no. | | | | | | | | | | | | | | | | | | | | | | | | | | | | | Score | | | | | | | | | | | | | | | | | | | | | | | | | | | | | | | M.P. | | | | | | | | | | | | | | | | | | | | | | | | | | | | | | | | | | Cov. (%) | | | | | | | | | | | | | | | | | | | | | | | | | | | | | | | | | | | | | | | Blast score | | | | | | | | | | | | | | | | | | | | | | | | | | | | | | | | | | | | | | | | | | | | | Mr (kDa) / pI | | | | | | | | | | | | | | | | | | | | | | | | | | | | | | | | | | | | | | | | | | | | | | | | | | | | | | | | | | | | | | | | | | | | | | | | | | | | | | | | | | | | | | | | | | | | | | | | | | | | | | Ratio | | | | | | | | | | | | | | | | | | | | | | | | | | | | | | | | | | | | | | | | | | | | | | | | | | | | | | | | | | | | | | | | | | | | | | |  |  |  |  |  |  |  |  |  |  |  |  |  |  |  |  |  |  |  |  |  |  |  |  |  |  |  |  |  |  |  |  |  |  |  |  |
| --- | --- | --- | --- | --- | --- | --- | --- | --- | --- | --- | --- | --- | --- | --- | --- | --- | --- | --- | --- | --- | --- | --- | --- | --- | --- | --- | --- | --- | --- | --- | --- | --- | --- | --- | --- | --- | --- | --- | --- | --- | --- | --- | --- | --- | --- | --- | --- | --- | --- | --- | --- | --- | --- | --- | --- | --- | --- | --- | --- | --- | --- | --- | --- | --- | --- | --- | --- | --- | --- | --- | --- | --- | --- | --- | --- | --- | --- | --- | --- | --- | --- | --- | --- | --- | --- | --- | --- | --- | --- | --- | --- | --- | --- | --- | --- | --- | --- | --- | --- | --- | --- | --- | --- | --- | --- | --- | --- | --- | --- | --- | --- | --- | --- | --- | --- | --- | --- | --- | --- | --- | --- | --- | --- | --- | --- | --- | --- | --- | --- | --- | --- | --- | --- | --- | --- | --- | --- | --- | --- | --- | --- | --- | --- | --- | --- | --- | --- | --- | --- | --- | --- | --- | --- | --- | --- | --- | --- | --- | --- | --- | --- | --- | --- | --- | --- | --- | --- | --- | --- | --- | --- | --- | --- | --- | --- | --- | --- | --- | --- | --- | --- | --- | --- | --- | --- | --- | --- | --- | --- | --- | --- | --- | --- | --- | --- | --- | --- | --- | --- | --- | --- | --- | --- | --- | --- | --- | --- | --- | --- | --- | --- | --- | --- | --- | --- | --- | --- | --- | --- | --- | --- | --- | --- | --- | --- | --- | --- | --- | --- | --- | --- | --- | --- | --- | --- | --- | --- | --- | --- | --- | --- | --- | --- | --- | --- | --- | --- | --- | --- | --- | --- | --- | --- | --- | --- | --- | --- | --- | --- | --- | --- | --- | --- | --- | --- | --- | --- | --- | --- | --- | --- | --- | --- | --- | --- | --- | --- | --- | --- | --- | --- | --- | --- | --- | --- | --- | --- | --- | --- | --- | --- | --- | --- | --- | --- | --- | --- | --- | --- | --- | --- | --- | --- | --- | --- | --- | --- | --- | --- | --- | --- | --- | --- | --- | --- | --- | --- | --- | --- | --- | --- | --- | --- | --- | --- | --- | --- | --- | --- | --- | --- | --- | --- | --- | --- | --- | --- | --- | --- | --- | --- | --- | --- | --- | --- | --- | --- | --- | --- | --- | --- | --- | --- | --- | --- | --- | --- | --- | --- | --- | --- | --- | --- | --- | --- | --- | --- | --- | --- | --- | --- | --- | --- | --- | --- | --- | --- | --- | --- | --- | --- | --- | --- | --- | --- | --- | --- | --- | --- | --- | --- | --- | --- | --- | --- | --- | --- | --- | --- | --- | --- | --- | --- | --- | --- | --- | --- |
|  |  |  |  |  |  |  |  |  |  |  |  |  |  |  |  |  |  |  |  |  |  |  |  |  |  |  |  |  |  |  |  |  |  |  |  |  |  |  |  |  |  |  |  |  |  |  |  |  |  |  |  |  |  |  |  |  |  |  |  |  |  |  |  |  |  |  |  |  |  |  |  |  |  |  |  |  |  |  |  |  |  |  |  |  |  |  |  |  |  |  |  |  |  |  |  |  |  |  |  |  |  |  |  |  |  |  |  |  |  |  |  |  |  |  |  |  |  |  |  |  |  |  |  |  |  |  |  |  |  |  |  |  |  |  |  |  |  |  |  |  |  |  |  |  |  |  |  |  |  |  |  |  |  |  |  |  |  |  |  |  |  |  |  |  |  |  |  |  |  |  |  |  |  |  |  |  |  |  |  |  |  |  |  |  |  |  |  |  |  |  |  |  |  |  |  |  |  |  | Theo. | | | | | | | | | | | | | | | | | | | | | | | | | | | | | | | | | | | | | | | | | Exp. | | | | | | | | | | | | | | | | | | | | | | | | | | | | | | | | | | | | | | | | | | | | | | | | | | | | | | | | | | | | |  |  |  |  |  |  |  |  |  |  |  |  |  |  |  |  |  |  |  |  |  |  |  |  |  |  |  |  |  |  |  |  |  |  |  |  |  |  |  |  |  |  |  |  |  |  |  |  |  |  |  |  |  |  |  |  |  |  |  |  |  |  |  |  |  |  |  |  |  |  |  |  |  |  |  |  |  |  |  |  |  |  |  |  |  |  |  |  |  |  |  |  |  |  |  |  |  |  |  |  |  |  |  |  |  |  |  |
| 1e | | | uncharacterized protein LOC100305855 precursor | | | | | | | | | | | | | | | | | | Glyma01g10900.1 | | | | | | | | | | | | | | | | | | | | | | | | | | | | | 139 | | | | | | | | | | | | | | | | | | | | | | | | | | | | | | | 2 | | | | | | | | | | | | | | | | | | | | | | | | | | | | | | | | | | 10 | | | | | | | | | | | | | | | | | | | | | | | | | | | | | | | | | | | | | | | 385 | | | | | | | | | | | | | | | | | | | | | | | | | | | | | | | | | | | | | | | | | | | | | 22.9/4.85 | | | | | | | | | | | | | | | | | | | | | | | | | | | | | | | | | | | | | | | | |  | | | | | | | | | | | | | | | | | | | | | | | | | | | | | | | | | | | | | | | | | | | | | | | | | | | | | | | | | | | | |  | | | | | | | | | | | | | | | | | | | | | | | | | | | | | | | | | | | | | | | | | | | | | | | | | | | | | | | | | | | | | | | | | | | | | | |  |  |  |  |  |  |  |  |  |  |  |  |  |  |  |  |  |  |  |  |  |  |  |  |  |  |  |  |  |  |  |  |  |  |  |  |
|  | | | Calc. Mass | | | | | | | | | | | | | | | | | | Observ. Mass | | | | | | | | | | | | | | | | | | | | | | | | | | | | | Da | | | | | | | | | | | | | | | | | | | | | | | | | | | | | | | ppm | | | | | | | | | | | | | | | | | | | | | | | | | | | | | | | | | | start | | | | | | | | | | | | | | | | | | | | | | | | | | | | | | | | | | | | | | | end | | | | | | | | | | | | | | | | | | | | | | | | | | | | | | | | | | | | | | | | | | | | | sequence | | | | | | | | | | | | | | | | | | | | | | | | | | | | | | | | | | | | | | | | | | | | | | | | | | | | | | | | | | | | | | | | | | | | | | | | | | | | | | | | | | | | | | | | | | | | | | | | | | | | | | | | | | | | | | | | | | | | | | | | | | | | | | | | | | | | | | | | | | | | | | | | | | | | | | | | | | | | | | | | | | | | | | | | | | | | |  |  |  |  |  |  |  |  |  |  |  |  |  |  |  |  |  |  |  |  |  |  |  |  |  |  |  |  |  |  |  |  |  |  |  |  |
|  | | | 1447.6766 | | | | | | | | | | | | | | | | | | 1447.676 | | | | | | | | | | | | | | | | | | | | | | | | | | | | | 0 | | | | | | | | | | | | | | | | | | | | | | | | | | | | | | | 0 | | | | | | | | | | | | | | | | | | | | | | | | | | | | | | | | | | 156 | | | | | | | | | | | | | | | | | | | | | | | | | | | | | | | | | | | | | | | 167 | | | | | | | | | | | | | | | | | | | | | | | | | | | | | | | | | | | | | | | | | | | | | LVFCPQQAEDNK | | | | | | | | | | | | | | | | | | | | | | | | | | | | | | | | | | | | | | | | | | | | | | | | | | | | | | | | | | | | | | | | | | | | | | | | | | | | | | | | | | | | | | | | | | | | | | | | | | | | | | | | | | | | | | | | | | | | | | | | | | | | | | | | | | | | | | | | | | | | | | | | | | | | | | | | | | | | | | | | | | | | | | | | | | | | |  |  |  |  |  |  |  |  |  |  |  |  |  |  |  |  |  |  |  |  |  |  |  |  |  |  |  |  |  |  |  |  |  |  |  |  |
|  | | | 1199.7026 | | | | | | | | | | | | | | | | | | 1199.702 | | | | | | | | | | | | | | | | | | | | | | | | | | | | | 0 | | | | | | | | | | | | | | | | | | | | | | | | | | | | | | | -1 | | | | | | | | | | | | | | | | | | | | | | | | | | | | | | | | | | 188 | | | | | | | | | | | | | | | | | | | | | | | | | | | | | | | | | | | | | | | 197 | | | | | | | | | | | | | | | | | | | | | | | | | | | | | | | | | | | | | | | | | | | | | NKPLVVQFQK | | | | | | | | | | | | | | | | | | | | | | | | | | | | | | | | | | | | | | | | | | | | | | | | | | | | | | | | | | | | | | | | | | | | | | | | | | | | | | | | | | | | | | | | | | | | | | | | | | | | | | | | | | | | | | | | | | | | | | | | | | | | | | | | | | | | | | | | | | | | | | | | | | | | | | | | | | | | | | | | | | | | | | | | | | | | |  |  |  |  |  |  |  |  |  |  |  |  |  |  |  |  |  |  |  |  |  |  |  |  |  |  |  |  |  |  |  |  |  |  |  |  |
|  | | | 1199.7026 | | | | | | | | | | | | | | | | | | 1199.702 | | | | | | | | | | | | | | | | | | | | | | | | | | | | | 0 | | | | | | | | | | | | | | | | | | | | | | | | | | | | | | | -1 | | | | | | | | | | | | | | | | | | | | | | | | | | | | | | | | | | 188 | | | | | | | | | | | | | | | | | | | | | | | | | | | | | | | | | | | | | | | 197 | | | | | | | | | | | | | | | | | | | | | | | | | | | | | | | | | | | | | | | | | | | | | NKPLVVQFQK | | | | | | | | | | | | | | | | | | | | | | | | | | | | | | | | | | | | | | | | | | | | | | | | | | | | | | | | | | | | | | | | | | | | | | | | | | | | | | | | | | | | | | | | | | | | | | | | | | | | | | | | | | | | | | | | | | | | | | | | | | | | | | | | | | | | | | | | | | | | | | | | | | | | | | | | | | | | | | | | | | | | | | | | | | | | |  |  |  |  |  |  |  |  |  |  |  |  |  |  |  |  |  |  |  |  |  |  |  |  |  |  |  |  |  |  |  |  |  |  |  |  |
|  | | | 1199.7026 | | | | | | | | | | | | | | | | | | 1199.702 | | | | | | | | | | | | | | | | | | | | | | | | | | | | | 0 | | | | | | | | | | | | | | | | | | | | | | | | | | | | | | | 0 | | | | | | | | | | | | | | | | | | | | | | | | | | | | | | | | | | 188 | | | | | | | | | | | | | | | | | | | | | | | | | | | | | | | | | | | | | | | 197 | | | | | | | | | | | | | | | | | | | | | | | | | | | | | | | | | | | | | | | | | | | | | NKPLVVQFQK | | | | | | | | | | | | | | | | | | | | | | | | | | | | | | | | | | | | | | | | | | | | | | | | | | | | | | | | | | | | | | | | | | | | | | | | | | | | | | | | | | | | | | | | | | | | | | | | | | | | | | | | | | | | | | | | | | | | | | | | | | | | | | | | | | | | | | | | | | | | | | | | | | | | | | | | | | | | | | | | | | | | | | | | | | | | |  |  |  |  |  |  |  |  |  |  |  |  |  |  |  |  |  |  |  |  |  |  |  |  |  |  |  |  |  |  |  |  |  |  |  |  |
|  | | | 1199.7026 | | | | | | | | | | | | | | | | | | 1199.702 | | | | | | | | | | | | | | | | | | | | | | | | | | | | | 0 | | | | | | | | | | | | | | | | | | | | | | | | | | | | | | | 0 | | | | | | | | | | | | | | | | | | | | | | | | | | | | | | | | | | 188 | | | | | | | | | | | | | | | | | | | | | | | | | | | | | | | | | | | | | | | 197 | | | | | | | | | | | | | | | | | | | | | | | | | | | | | | | | | | | | | | | | | | | | | NKPLVVQFQK | | | | | | | | | | | | | | | | | | | | | | | | | | | | | | | | | | | | | | | | | | | | | | | | | | | | | | | | | | | | | | | | | | | | | | | | | | | | | | | | | | | | | | | | | | | | | | | | | | | | | | | | | | | | | | | | | | | | | | | | | | | | | | | | | | | | | | | | | | | | | | | | | | | | | | | | | | | | | | | | | | | | | | | | | | | | |  |  |  |  |  |  |  |  |  |  |  |  |  |  |  |  |  |  |  |  |  |  |  |  |  |  |  |  |  |  |  |  |  |  |  |  |
|  | | | 1199.7026 | | | | | | | | | | | | | | | | | | 1199.703 | | | | | | | | | | | | | | | | | | | | | | | | | | | | | 0 | | | | | | | | | | | | | | | | | | | | | | | | | | | | | | | 0 | | | | | | | | | | | | | | | | | | | | | | | | | | | | | | | | | | 188 | | | | | | | | | | | | | | | | | | | | | | | | | | | | | | | | | | | | | | | 197 | | | | | | | | | | | | | | | | | | | | | | | | | | | | | | | | | | | | | | | | | | | | | NKPLVVQFQK | | | | | | | | | | | | | | | | | | | | | | | | | | | | | | | | | | | | | | | | | | | | | | | | | | | | | | | | | | | | | | | | | | | | | | | | | | | | | | | | | | | | | | | | | | | | | | | | | | | | | | | | | | | | | | | | | | | | | | | | | | | | | | | | | | | | | | | | | | | | | | | | | | | | | | | | | | | | | | | | | | | | | | | | | | | | |  |  |  |  |  |  |  |  |  |  |  |  |  |  |  |  |  |  |  |  |  |  |  |  |  |  |  |  |  |  |  |  |  |  |  |  |
|  | | | 1199.7026 | | | | | | | | | | | | | | | | | | 1199.703 | | | | | | | | | | | | | | | | | | | | | | | | | | | | | 0 | | | | | | | | | | | | | | | | | | | | | | | | | | | | | | | 0 | | | | | | | | | | | | | | | | | | | | | | | | | | | | | | | | | | 188 | | | | | | | | | | | | | | | | | | | | | | | | | | | | | | | | | | | | | | | 197 | | | | | | | | | | | | | | | | | | | | | | | | | | | | | | | | | | | | | | | | | | | | | NKPLVVQFQK | | | | | | | | | | | | | | | | | | | | | | | | | | | | | | | | | | | | | | | | | | | | | | | | | | | | | | | | | | | | | | | | | | | | | | | | | | | | | | | | | | | | | | | | | | | | | | | | | | | | | | | | | | | | | | | | | | | | | | | | | | | | | | | | | | | | | | | | | | | | | | | | | | | | | | | | | | | | | | | | | | | | | | | | | | | | |  |  |  |  |  |  |  |  |  |  |  |  |  |  |  |  |  |  |  |  |  |  |  |  |  |  |  |  |  |  |  |  |  |  |  |  |
|  | | | 1199.7026 | | | | | | | | | | | | | | | | | | 1199.703 | | | | | | | | | | | | | | | | | | | | | | | | | | | | | 0 | | | | | | | | | | | | | | | | | | | | | | | | | | | | | | | 0 | | | | | | | | | | | | | | | | | | | | | | | | | | | | | | | | | | 188 | | | | | | | | | | | | | | | | | | | | | | | | | | | | | | | | | | | | | | | 197 | | | | | | | | | | | | | | | | | | | | | | | | | | | | | | | | | | | | | | | | | | | | | NKPLVVQFQK | | | | | | | | | | | | | | | | | | | | | | | | | | | | | | | | | | | | | | | | | | | | | | | | | | | | | | | | | | | | | | | | | | | | | | | | | | | | | | | | | | | | | | | | | | | | | | | | | | | | | | | | | | | | | | | | | | | | | | | | | | | | | | | | | | | | | | | | | | | | | | | | | | | | | | | | | | | | | | | | | | | | | | | | | | | | |  |  |  |  |  |  |  |  |  |  |  |  |  |  |  |  |  |  |  |  |  |  |  |  |  |  |  |  |  |  |  |  |  |  |  |  |
|  | | | 1199.7026 | | | | | | | | | | | | | | | | | | 1199.703 | | | | | | | | | | | | | | | | | | | | | | | | | | | | | 0 | | | | | | | | | | | | | | | | | | | | | | | | | | | | | | | 0 | | | | | | | | | | | | | | | | | | | | | | | | | | | | | | | | | | 188 | | | | | | | | | | | | | | | | | | | | | | | | | | | | | | | | | | | | | | | 197 | | | | | | | | | | | | | | | | | | | | | | | | | | | | | | | | | | | | | | | | | | | | | NKPLVVQFQK | | | | | | | | | | | | | | | | | | | | | | | | | | | | | | | | | | | | | | | | | | | | | | | | | | | | | | | | | | | | | | | | | | | | | | | | | | | | | | | | | | | | | | | | | | | | | | | | | | | | | | | | | | | | | | | | | | | | | | | | | | | | | | | | | | | | | | | | | | | | | | | | | | | | | | | | | | | | | | | | | | | | | | | | | | | | |  |  |  |  |  |  |  |  |  |  |  |  |  |  |  |  |  |  |  |  |  |  |  |  |  |  |  |  |  |  |  |  |  |  |  |  |
|  | | | 1199.7026 | | | | | | | | | | | | | | | | | | 1199.703 | | | | | | | | | | | | | | | | | | | | | | | | | | | | | 0 | | | | | | | | | | | | | | | | | | | | | | | | | | | | | | | 1 | | | | | | | | | | | | | | | | | | | | | | | | | | | | | | | | | | 188 | | | | | | | | | | | | | | | | | | | | | | | | | | | | | | | | | | | | | | | 197 | | | | | | | | | | | | | | | | | | | | | | | | | | | | | | | | | | | | | | | | | | | | | NKPLVVQFQK | | | | | | | | | | | | | | | | | | | | | | | | | | | | | | | | | | | | | | | | | | | | | | | | | | | | | | | | | | | | | | | | | | | | | | | | | | | | | | | | | | | | | | | | | | | | | | | | | | | | | | | | | | | | | | | | | | | | | | | | | | | | | | | | | | | | | | | | | | | | | | | | | | | | | | | | | | | | | | | | | | | | | | | | | | | | |  |  |  |  |  |  |  |  |  |  |  |  |  |  |  |  |  |  |  |  |  |  |  |  |  |  |  |  |  |  |  |  |  |  |  |  |
| Spot no. | | | Homologous protein | | | | | | | | | | | | | | | | | | Accession no. | | | | | | | | | | | | | | | | | | | | | | | | | | | | | Score | | | | | | | | | | | | | | | | | | | | | | | | | | | | | | | M.P. | | | | | | | | | | | | | | | | | | | | | | | | | | | | | | | | | | Cov. (%) | | | | | | | | | | | | | | | | | | | | | | | | | | | | | | | | | | | | | | | Blast score | | | | | | | | | | | | | | | | | | | | | | | | | | | | | | | | | | | | | | | | | | | | | Mr (kDa) / pI | | | | | | | | | | | | | | | | | | | | | | | | | | | | | | | | | | | | | | | | | | | | | | | | | | | | | | | | | | | | | | | | | | | | | | | | | | | | | | | | | | | | | | | | | | | | | | | | | | | | | | | | | | | | | | | | | | Ratio | | | | | | | | | | | | | | | | | | | | | | | | | | | | | | | | | | | | | | | | | | | | | | | | | | | | | | | | | | | | | | | | | | |  |  |  |  |  |  |  |  |  |  |  |  |  |  |  |  |  |  |  |  |  |  |  |  |  |  |  |  |
|  |  |  |  |  |  |  |  |  |  |  |  |  |  |  |  |  |  |  |  |  |  |  |  |  |  |  |  |  |  |  |  |  |  |  |  |  |  |  |  |  |  |  |  |  |  |  |  |  |  |  |  |  |  |  |  |  |  |  |  |  |  |  |  |  |  |  |  |  |  |  |  |  |  |  |  |  |  |  |  |  |  |  |  |  |  |  |  |  |  |  |  |  |  |  |  |  |  |  |  |  |  |  |  |  |  |  |  |  |  |  |  |  |  |  |  |  |  |  |  |  |  |  |  |  |  |  |  |  |  |  |  |  |  |  |  |  |  |  |  |  |  |  |  |  |  |  |  |  |  |  |  |  |  |  |  |  |  |  |  |  |  |  |  |  |  |  |  |  |  |  |  |  |  |  |  |  |  |  |  |  |  |  |  |  |  |  |  |  |  |  |  |  |  |  |  |  |  |  | Theo. | | | | | | | | | | | | | | | | | | | | | | | | | | | | | | | | | | | | | | | | | | | | | | | | | | | | | | | | | | | | | | | | | | | | | | | | | | | Exp. | | | | | | | | | | | | | | | | | | | | | | | | | | | | | | | | | | | | | | |  |  |  |  |  |  |  |  |  |  |  |  |  |  |  |  |  |  |  |  |  |  |  |  |  |  |  |  |  |  |  |  |  |  |  |  |  |  |  |  |  |  |  |  |  |  |  |  |  |  |  |  |  |  |  |  |  |  |  |  |  |  |  |  |  |  |  |  |  |  |  |  |  |  |  |  |  |  |  |  |  |  |  |  |  |  |  |  |  |  |  |  |  |  |  |
| 1f | | | uncharacterized protein LOC100527911 | | | | | | | | | | | | | | | | | | Glyma02g11540.1 | | | | | | | | | | | | | | | | | | | | | | | | | | | | | 129 | | | | | | | | | | | | | | | | | | | | | | | | | | | | | | | 7 | | | | | | | | | | | | | | | | | | | | | | | | | | | | | | | | | | 37 | | | | | | | | | | | | | | | | | | | | | | | | | | | | | | | | | | | | | | | 276 | | | | | | | | | | | | | | | | | | | | | | | | | | | | | | | | | | | | | | | | | | | | | 18.1/10.21 | | | | | | | | | | | | | | | | | | | | | | | | | | | | | | | | | | | | | | | | | | | | | | | | | | | | | | | | | | | | | | | | | | | | | | | | | | |  | | | | | | | | | | | | | | | | | | | | | | | | | | | | | | | | | | | | | | |  | | | | | | | | | | | | | | | | | | | | | | | | | | | | | | | | | | | | | | | | | | | | | | | | | | | | | | | | | | | | | | | | | | |  |  |  |  |  |  |  |  |  |  |  |  |  |  |  |  |  |  |  |  |  |  |  |  |  |  |  |  |
|  | | | Calc. Mass | | | | | | | | | | | | | | | | | | Observ. Mass | | | | | | | | | | | | | | | | | | | | | | | | | | | | | Da | | | | | | | | | | | | | | | | | | | | | | | | | | | | | | | ppm | | | | | | | | | | | | | | | | | | | | | | | | | | | | | | | | | | start | | | | | | | | | | | | | | | | | | | | | | | | | | | | | | | | | | | | | | | end | | | | | | | | | | | | | | | | | | | | | | | | | | | | | | | | | | | | | | | | | | | | | sequence | | | | | | | | | | | | | | | | | | | | | | | | | | | | | | | | | | | | | | | | | | | | | | | | | | | | | | | | | | | | | | | | | | | | | | | | | | | | | | | | | | | | | | | | | | | | | | | | | | | | | | | | | | | | | | | | | | | | | | | | | | | | | | | | | | | | | | | | | | | | | | | | | | | | | | | | | | | | | | | | | | | | | | | | | | | | |  |  |  |  |  |  |  |  |  |  |  |  |  |  |  |  |  |  |  |  |  |  |  |  |  |  |  |  |  |  |  |  |  |  |  |  |
|  | | | 1011.5059 | | | | | | | | | | | | | | | | | | 1011.507 | | | | | | | | | | | | | | | | | | | | | | | | | | | | | 0 | | | | | | | | | | | | | | | | | | | | | | | | | | | | | | | 1 | | | | | | | | | | | | | | | | | | | | | | | | | | | | | | | | | | 28 | | | | | | | | | | | | | | | | | | | | | | | | | | | | | | | | | | | | | | | 36 | | | | | | | | | | | | | | | | | | | | | | | | | | | | | | | | | | | | | | | | | | | | | TAVAVTYCK | | | | | | | | | | | | | | | | | | | | | | | | | | | | | | | | | | | | | | | | | | | | | | | | | | | | | | | | | | | | | | | | | | | | | | | | | | | | | | | | | | | | | | | | | | | | | | | | | | | | | | | | | | | | | | | | | | | | | | | | | | | | | | | | | | | | | | | | | | | | | | | | | | | | | | | | | | | | | | | | | | | | | | | | | | | | |  |  |  |  |  |  |  |  |  |  |  |  |  |  |  |  |  |  |  |  |  |  |  |  |  |  |  |  |  |  |  |  |  |  |  |  |
|  | | | 1750.9287 | | | | | | | | | | | | | | | | | | 1750.927 | | | | | | | | | | | | | | | | | | | | | | | | | | | | | 0 | | | | | | | | | | | | | | | | | | | | | | | | | | | | | | | -1 | | | | | | | | | | | | | | | | | | | | | | | | | | | | | | | | | | 44 | | | | | | | | | | | | | | | | | | | | | | | | | | | | | | | | | | | | | | | 58 | | | | | | | | | | | | | | | | | | | | | | | | | | | | | | | | | | | | | | | | | | | | | INGCPIELVEPEILR | | | | | | | | | | | | | | | | | | | | | | | | | | | | | | | | | | | | | | | | | | | | | | | | | | | | | | | | | | | | | | | | | | | | | | | | | | | | | | | | | | | | | | | | | | | | | | | | | | | | | | | | | | | | | | | | | | | | | | | | | | | | | | | | | | | | | | | | | | | | | | | | | | | | | | | | | | | | | | | | | | | | | | | | | | | | |  |  |  |  |  |  |  |  |  |  |  |  |  |  |  |  |  |  |  |  |  |  |  |  |  |  |  |  |  |  |  |  |  |  |  |  |
|  | | | 1750.9287 | | | | | | | | | | | | | | | | | | 1750.929 | | | | | | | | | | | | | | | | | | | | | | | | | | | | | 0 | | | | | | | | | | | | | | | | | | | | | | | | | | | | | | | 0 | | | | | | | | | | | | | | | | | | | | | | | | | | | | | | | | | | 44 | | | | | | | | | | | | | | | | | | | | | | | | | | | | | | | | | | | | | | | 58 | | | | | | | | | | | | | | | | | | | | | | | | | | | | | | | | | | | | | | | | | | | | | INGCPIELVEPEILR | | | | | | | | | | | | | | | | | | | | | | | | | | | | | | | | | | | | | | | | | | | | | | | | | | | | | | | | | | | | | | | | | | | | | | | | | | | | | | | | | | | | | | | | | | | | | | | | | | | | | | | | | | | | | | | | | | | | | | | | | | | | | | | | | | | | | | | | | | | | | | | | | | | | | | | | | | | | | | | | | | | | | | | | | | | | |  |  |  |  |  |  |  |  |  |  |  |  |  |  |  |  |  |  |  |  |  |  |  |  |  |  |  |  |  |  |  |  |  |  |  |  |
|  | | | 1750.9287 | | | | | | | | | | | | | | | | | | 1750.932 | | | | | | | | | | | | | | | | | | | | | | | | | | | | | 0 | | | | | | | | | | | | | | | | | | | | | | | | | | | | | | | 2 | | | | | | | | | | | | | | | | | | | | | | | | | | | | | | | | | | 44 | | | | | | | | | | | | | | | | | | | | | | | | | | | | | | | | | | | | | | | 58 | | | | | | | | | | | | | | | | | | | | | | | | | | | | | | | | | | | | | | | | | | | | | INGCPIELVEPEILR | | | | | | | | | | | | | | | | | | | | | | | | | | | | | | | | | | | | | | | | | | | | | | | | | | | | | | | | | | | | | | | | | | | | | | | | | | | | | | | | | | | | | | | | | | | | | | | | | | | | | | | | | | | | | | | | | | | | | | | | | | | | | | | | | | | | | | | | | | | | | | | | | | | | | | | | | | | | | | | | | | | | | | | | | | | | |  |  |  |  |  |  |  |  |  |  |  |  |  |  |  |  |  |  |  |  |  |  |  |  |  |  |  |  |  |  |  |  |  |  |  |  |
|  | | | 1374.8275 | | | | | | | | | | | | | | | | | | 1374.823 | | | | | | | | | | | | | | | | | | | | | | | | | | | | | 0 | | | | | | | | | | | | | | | | | | | | | | | | | | | | | | | -4 | | | | | | | | | | | | | | | | | | | | | | | | | | | | | | | | | | 59 | | | | | | | | | | | | | | | | | | | | | | | | | | | | | | | | | | | | | | | 70 | | | | | | | | | | | | | | | | | | | | | | | | | | | | | | | | | | | | | | | | | | | | | FKAFEPILLLGK | | | | | | | | | | | | | | | | | | | | | | | | | | | | | | | | | | | | | | | | | | | | | | | | | | | | | | | | | | | | | | | | | | | | | | | | | | | | | | | | | | | | | | | | | | | | | | | | | | | | | | | | | | | | | | | | | | | | | | | | | | | | | | | | | | | | | | | | | | | | | | | | | | | | | | | | | | | | | | | | | | | | | | | | | | | | |  |  |  |  |  |  |  |  |  |  |  |  |  |  |  |  |  |  |  |  |  |  |  |  |  |  |  |  |  |  |  |  |  |  |  |  |
|  | | | 1099.6641 | | | | | | | | | | | | | | | | | | 1099.663 | | | | | | | | | | | | | | | | | | | | | | | | | | | | | 0 | | | | | | | | | | | | | | | | | | | | | | | | | | | | | | | -1 | | | | | | | | | | | | | | | | | | | | | | | | | | | | | | | | | | 61 | | | | | | | | | | | | | | | | | | | | | | | | | | | | | | | | | | | | | | | 70 | | | | | | | | | | | | | | | | | | | | | | | | | | | | | | | | | | | | | | | | | | | | | AFEPILLLGK | | | | | | | | | | | | | | | | | | | | | | | | | | | | | | | | | | | | | | | | | | | | | | | | | | | | | | | | | | | | | | | | | | | | | | | | | | | | | | | | | | | | | | | | | | | | | | | | | | | | | | | | | | | | | | | | | | | | | | | | | | | | | | | | | | | | | | | | | | | | | | | | | | | | | | | | | | | | | | | | | | | | | | | | | | | | |  |  |  |  |  |  |  |  |  |  |  |  |  |  |  |  |  |  |  |  |  |  |  |  |  |  |  |  |  |  |  |  |  |  |  |  |
|  | | | 1099.6641 | | | | | | | | | | | | | | | | | | 1099.664 | | | | | | | | | | | | | | | | | | | | | | | | | | | | | 0 | | | | | | | | | | | | | | | | | | | | | | | | | | | | | | | 0 | | | | | | | | | | | | | | | | | | | | | | | | | | | | | | | | | | 61 | | | | | | | | | | | | | | | | | | | | | | | | | | | | | | | | | | | | | | | 70 | | | | | | | | | | | | | | | | | | | | | | | | | | | | | | | | | | | | | | | | | | | | | AFEPILLLGK | | | | | | | | | | | | | | | | | | | | | | | | | | | | | | | | | | | | | | | | | | | | | | | | | | | | | | | | | | | | | | | | | | | | | | | | | | | | | | | | | | | | | | | | | | | | | | | | | | | | | | | | | | | | | | | | | | | | | | | | | | | | | | | | | | | | | | | | | | | | | | | | | | | | | | | | | | | | | | | | | | | | | | | | | | | | |  |  |  |  |  |  |  |  |  |  |  |  |  |  |  |  |  |  |  |  |  |  |  |  |  |  |  |  |  |  |  |  |  |  |  |  |
|  | | | 1099.6641 | | | | | | | | | | | | | | | | | | 1099.665 | | | | | | | | | | | | | | | | | | | | | | | | | | | | | 0 | | | | | | | | | | | | | | | | | | | | | | | | | | | | | | | 1 | | | | | | | | | | | | | | | | | | | | | | | | | | | | | | | | | | 61 | | | | | | | | | | | | | | | | | | | | | | | | | | | | | | | | | | | | | | | 70 | | | | | | | | | | | | | | | | | | | | | | | | | | | | | | | | | | | | | | | | | | | | | AFEPILLLGK | | | | | | | | | | | | | | | | | | | | | | | | | | | | | | | | | | | | | | | | | | | | | | | | | | | | | | | | | | | | | | | | | | | | | | | | | | | | | | | | | | | | | | | | | | | | | | | | | | | | | | | | | | | | | | | | | | | | | | | | | | | | | | | | | | | | | | | | | | | | | | | | | | | | | | | | | | | | | | | | | | | | | | | | | | | | |  |  |  |  |  |  |  |  |  |  |  |  |  |  |  |  |  |  |  |  |  |  |  |  |  |  |  |  |  |  |  |  |  |  |  |  |
|  | | | 1342.7972 | | | | | | | | | | | | | | | | | | 1342.797 | | | | | | | | | | | | | | | | | | | | | | | | | | | | | 0 | | | | | | | | | | | | | | | | | | | | | | | | | | | | | | | 0 | | | | | | | | | | | | | | | | | | | | | | | | | | | | | | | | | | 61 | | | | | | | | | | | | | | | | | | | | | | | | | | | | | | | | | | | | | | | 72 | | | | | | | | | | | | | | | | | | | | | | | | | | | | | | | | | | | | | | | | | | | | | AFEPILLLGKSR | | | | | | | | | | | | | | | | | | | | | | | | | | | | | | | | | | | | | | | | | | | | | | | | | | | | | | | | | | | | | | | | | | | | | | | | | | | | | | | | | | | | | | | | | | | | | | | | | | | | | | | | | | | | | | | | | | | | | | | | | | | | | | | | | | | | | | | | | | | | | | | | | | | | | | | | | | | | | | | | | | | | | | | | | | | | |  |  |  |  |  |  |  |  |  |  |  |  |  |  |  |  |  |  |  |  |  |  |  |  |  |  |  |  |  |  |  |  |  |  |  |  |
|  | | | 1485.8052 | | | | | | | | | | | | | | | | | | 1485.805 | | | | | | | | | | | | | | | | | | | | | | | | | | | | | 0 | | | | | | | | | | | | | | | | | | | | | | | | | | | | | | | 0 | | | | | | | | | | | | | | | | | | | | | | | | | | | | | | | | | | 82 | | | | | | | | | | | | | | | | | | | | | | | | | | | | | | | | | | | | | | | 95 | | | | | | | | | | | | | | | | | | | | | | | | | | | | | | | | | | | | | | | | | | | | | VKGGGHTSQIYAIR | | | | | | | | | | | | | | | | | | | | | | | | | | | | | | | | | | | | | | | | | | | | | | | | | | | | | | | | | | | | | | | | | | | | | | | | | | | | | | | | | | | | | | | | | | | | | | | | | | | | | | | | | | | | | | | | | | | | | | | | | | | | | | | | | | | | | | | | | | | | | | | | | | | | | | | | | | | | | | | | | | | | | | | | | | | | |  |  |  |  |  |  |  |  |  |  |  |  |  |  |  |  |  |  |  |  |  |  |  |  |  |  |  |  |  |  |  |  |  |  |  |  |
|  | | | 1485.8052 | | | | | | | | | | | | | | | | | | 1485.807 | | | | | | | | | | | | | | | | | | | | | | | | | | | | | 0 | | | | | | | | | | | | | | | | | | | | | | | | | | | | | | | 1 | | | | | | | | | | | | | | | | | | | | | | | | | | | | | | | | | | 82 | | | | | | | | | | | | | | | | | | | | | | | | | | | | | | | | | | | | | | | 95 | | | | | | | | | | | | | | | | | | | | | | | | | | | | | | | | | | | | | | | | | | | | | VKGGGHTSQIYAIR | | | | | | | | | | | | | | | | | | | | | | | | | | | | | | | | | | | | | | | | | | | | | | | | | | | | | | | | | | | | | | | | | | | | | | | | | | | | | | | | | | | | | | | | | | | | | | | | | | | | | | | | | | | | | | | | | | | | | | | | | | | | | | | | | | | | | | | | | | | | | | | | | | | | | | | | | | | | | | | | | | | | | | | | | | | | |  |  |  |  |  |  |  |  |  |  |  |  |  |  |  |  |  |  |  |  |  |  |  |  |  |  |  |  |  |  |  |  |  |  |  |  |
|  | | | 938.5225 | | | | | | | | | | | | | | | | | | 938.5218 | | | | | | | | | | | | | | | | | | | | | | | | | | | | | 0 | | | | | | | | | | | | | | | | | | | | | | | | | | | | | | | -1 | | | | | | | | | | | | | | | | | | | | | | | | | | | | | | | | | | 101 | | | | | | | | | | | | | | | | | | | | | | | | | | | | | | | | | | | | | | | 108 | | | | | | | | | | | | | | | | | | | | | | | | | | | | | | | | | | | | | | | | | | | | | ALVAFYQK | | | | | | | | | | | | | | | | | | | | | | | | | | | | | | | | | | | | | | | | | | | | | | | | | | | | | | | | | | | | | | | | | | | | | | | | | | | | | | | | | | | | | | | | | | | | | | | | | | | | | | | | | | | | | | | | | | | | | | | | | | | | | | | | | | | | | | | | | | | | | | | | | | | | | | | | | | | | | | | | | | | | | | | | | | | | |  |  |  |  |  |  |  |  |  |  |  |  |  |  |  |  |  |  |  |  |  |  |  |  |  |  |  |  |  |  |  |  |  |  |  |  |
|  | | | 938.5225 | | | | | | | | | | | | | | | | | | 938.5221 | | | | | | | | | | | | | | | | | | | | | | | | | | | | | 0 | | | | | | | | | | | | | | | | | | | | | | | | | | | | | | | 0 | | | | | | | | | | | | | | | | | | | | | | | | | | | | | | | | | | 101 | | | | | | | | | | | | | | | | | | | | | | | | | | | | | | | | | | | | | | | 108 | | | | | | | | | | | | | | | | | | | | | | | | | | | | | | | | | | | | | | | | | | | | | ALVAFYQK | | | | | | | | | | | | | | | | | | | | | | | | | | | | | | | | | | | | | | | | | | | | | | | | | | | | | | | | | | | | | | | | | | | | | | | | | | | | | | | | | | | | | | | | | | | | | | | | | | | | | | | | | | | | | | | | | | | | | | | | | | | | | | | | | | | | | | | | | | | | | | | | | | | | | | | | | | | | | | | | | | | | | | | | | | | | |  |  |  |  |  |  |  |  |  |  |  |  |  |  |  |  |  |  |  |  |  |  |  |  |  |  |  |  |  |  |  |  |  |  |  |  |
|  | | | 938.5225 | | | | | | | | | | | | | | | | | | 938.5226 | | | | | | | | | | | | | | | | | | | | | | | | | | | | | 0 | | | | | | | | | | | | | | | | | | | | | | | | | | | | | | | 0 | | | | | | | | | | | | | | | | | | | | | | | | | | | | | | | | | | 101 | | | | | | | | | | | | | | | | | | | | | | | | | | | | | | | | | | | | | | | 108 | | | | | | | | | | | | | | | | | | | | | | | | | | | | | | | | | | | | | | | | | | | | | ALVAFYQK | | | | | | | | | | | | | | | | | | | | | | | | | | | | | | | | | | | | | | | | | | | | | | | | | | | | | | | | | | | | | | | | | | | | | | | | | | | | | | | | | | | | | | | | | | | | | | | | | | | | | | | | | | | | | | | | | | | | | | | | | | | | | | | | | | | | | | | | | | | | | | | | | | | | | | | | | | | | | | | | | | | | | | | | | | | | |  |  |  |  |  |  |  |  |  |  |  |  |  |  |  |  |  |  |  |  |  |  |  |  |  |  |  |  |  |  |  |  |  |  |  |  |
| Spot no. | | | Homologous protein | | | | | | | | | | | | | | | | | | Accession no. | | | | | | | | | | | | | | | | | | | | | | | | | | | | | Score | | | | | | | | | | | | | | | | | | | | | | | | | | | | | | | M.P. | | | | | | | | | | | | | | | | | | | | | | | | | | | | | | | | | | Cov. (%) | | | | | | | | | | | | | | | | | | | | | | | | | | | | | | | | | | | | | | | Blast score | | | | | | | | | | | | | | | | | | | | | | | | | | | | | | | | | | | | | | | | | | | | | Mr (kDa) / pI | | | | | | | | | | | | | | | | | | | | | | | | | | | | | | | | | | | | | | | | | | | | | | | | | | | | | | | | | | | | | | | | | | | | | | | | | | | | | | | | | | | | | | | | | | | | | | | | | | | | | | | Ratio | | | | | | | | | | | | | | | | | | | | | | | | | | | | | | | | | | | | | | | | | | | | | | | | | | | | | | | | | | | | | | | | | | | | | | | | | | | | | |  |  |  |  |  |  |  |  |  |  |  |  |  |  |  |  |  |  |  |  |  |  |  |  |  |  |  |  |
|  |  |  |  |  |  |  |  |  |  |  |  |  |  |  |  |  |  |  |  |  |  |  |  |  |  |  |  |  |  |  |  |  |  |  |  |  |  |  |  |  |  |  |  |  |  |  |  |  |  |  |  |  |  |  |  |  |  |  |  |  |  |  |  |  |  |  |  |  |  |  |  |  |  |  |  |  |  |  |  |  |  |  |  |  |  |  |  |  |  |  |  |  |  |  |  |  |  |  |  |  |  |  |  |  |  |  |  |  |  |  |  |  |  |  |  |  |  |  |  |  |  |  |  |  |  |  |  |  |  |  |  |  |  |  |  |  |  |  |  |  |  |  |  |  |  |  |  |  |  |  |  |  |  |  |  |  |  |  |  |  |  |  |  |  |  |  |  |  |  |  |  |  |  |  |  |  |  |  |  |  |  |  |  |  |  |  |  |  |  |  |  |  |  |  |  |  |  |  | Theo. | | | | | | | | | | | | | | | | | | | | | | | | | | | | | | | | | | | | | | | | | | | Exp. | | | | | | | | | | | | | | | | | | | | | | | | | | | | | | | | | | | | | | | | | | | | | | | | | | | | | | | | | | | |  |  |  |  |  |  |  |  |  |  |  |  |  |  |  |  |  |  |  |  |  |  |  |  |  |  |  |  |  |  |  |  |  |  |  |  |  |  |  |  |  |  |  |  |  |  |  |  |  |  |  |  |  |  |  |  |  |  |  |  |  |  |  |  |  |  |  |  |  |  |  |  |  |  |  |  |  |  |  |  |  |  |  |  |  |  |  |  |  |  |  |  |  |  |  |  |  |  |  |  |  |  |  |  |  |  |
| 1g | | | glyceraldehyde-3-phosphate dehydrogenase | | | | | | | | | | | | | | | | | | Glyma04g36860.1 | | | | | | | | | | | | | | | | | | | | | | | | | | | | | 120 | | | | | | | | | | | | | | | | | | | | | | | | | | | | | | | 2 | | | | | | | | | | | | | | | | | | | | | | | | | | | | | | | | | | 13 | | | | | | | | | | | | | | | | | | | | | | | | | | | | | | | | | | | | | | | 637 | | | | | | | | | | | | | | | | | | | | | | | | | | | | | | | | | | | | | | | | | | | | | 37.1/6.72 | | | | | | | | | | | | | | | | | | | | | | | | | | | | | | | | | | | | | | | | | | |  | | | | | | | | | | | | | | | | | | | | | | | | | | | | | | | | | | | | | | | | | | | | | | | | | | | | | | | | | | | |  | | | | | | | | | | | | | | | | | | | | | | | | | | | | | | | | | | | | | | | | | | | | | | | | | | | | | | | | | | | | | | | | | | | | | | | | | | | | | |  |  |  |  |  |  |  |  |  |  |  |  |  |  |  |  |  |  |  |  |  |  |  |  |  |  |  |  |
|  | | | Calc. Mass | | | | | | | | | | | | | | | | | | Observ. Mass | | | | | | | | | | | | | | | | | | | | | | | | | | | | | Da | | | | | | | | | | | | | | | | | | | | | | | | | | | | | | | ppm | | | | | | | | | | | | | | | | | | | | | | | | | | | | | | | | | | start | | | | | | | | | | | | | | | | | | | | | | | | | | | | | | | | | | | | | | | end | | | | | | | | | | | | | | | | | | | | | | | | | | | | | | | | | | | | | | | | | | | | | sequence | | | | | | | | | | | | | | | | | | | | | | | | | | | | | | | | | | | | | | | | | | | | | | | | | | | | | | | | | | | | | | | | | | | | | | | | | | | | | | | | | | | | | | | | | | | | | | | | | | | | | | | | | | | | | | | | | | | | | | | | | | | | | | | | | | | | | | | | | | | | | | | | | | | | | | | | | | | | | | | | | | | | | | | | | | | | | | | | | | | | |  |  |  |  |  |  |  |  |  |  |  |  |  |  |  |  |  |  |  |  |  |  |  |  |  |  |  |  |
|  | | | 2904.4029 | | | | | | | | | | | | | | | | | | 2904.403 | | | | | | | | | | | | | | | | | | | | | | | | | | | | | 0 | | | | | | | | | | | | | | | | | | | | | | | | | | | | | | | 0 | | | | | | | | | | | | | | | | | | | | | | | | | | | | | | | | | | 83 | | | | | | | | | | | | | | | | | | | | | | | | | | | | | | | | | | | | | | | 109 | | | | | | | | | | | | | | | | | | | | | | | | | | | | | | | | | | | | | | | | | | | | | NPEEIPWGSTGADIIVESTGVFTDKDK | | | | | | | | | | | | | | | | | | | | | | | | | | | | | | | | | | | | | | | | | | | | | | | | | | | | | | | | | | | | | | | | | | | | | | | | | | | | | | | | | | | | | | | | | | | | | | | | | | | | | | | | | | | | | | | | | | | | | | | | | | | | | | | | | | | | | | | | | | | | | | | | | | | | | | | | | | | | | | | | | | | | | | | | | | | | | | | | | | | | |  |  |  |  |  |  |  |  |  |  |  |  |  |  |  |  |  |  |  |  |  |  |  |  |  |  |  |  |
|  | | | 2143.991 | | | | | | | | | | | | | | | | | | 2143.985 | | | | | | | | | | | | | | | | | | | | | | | | | | | | | 0 | | | | | | | | | | | | | | | | | | | | | | | | | | | | | | | -3 | | | | | | | | | | | | | | | | | | | | | | | | | | | | | | | | | | 274 | | | | | | | | | | | | | | | | | | | | | | | | | | | | | | | | | | | | | | | 293 | | | | | | | | | | | | | | | | | | | | | | | | | | | | | | | | | | | | | | | | | | | | | GILGYTEDDVVSTDFVGDSR | | | | | | | | | | | | | | | | | | | | | | | | | | | | | | | | | | | | | | | | | | | | | | | | | | | | | | | | | | | | | | | | | | | | | | | | | | | | | | | | | | | | | | | | | | | | | | | | | | | | | | | | | | | | | | | | | | | | | | | | | | | | | | | | | | | | | | | | | | | | | | | | | | | | | | | | | | | | | | | | | | | | | | | | | | | | | | | | | | | | |  |  |  |  |  |  |  |  |  |  |  |  |  |  |  |  |  |  |  |  |  |  |  |  |  |  |  |  |
| Spot no. | | | Homologous protein | | | | | | | | | | | | | | | | | | Accession no. | | | | | | | | | | | | | | | | | | | | | | | | | | | | | Score | | | | | | | | | | | | | | | | | | | | | | | | | | | | | | | | | | | M.P. | | | | | | | | | | | | | | | | | | | | | | | | | | | | | | | | | | | Cov. (%) | | | | | | | | | | | | | | | | | | | | | | | | | | | | | | | | | | | | | Blast score | | | | | | | | | | | | | | | | | | | | | | | | | | | | | | | | | | | | | | | | | | | | | Mr (kDa) / pI | | | | | | | | | | | | | | | | | | | | | | | | | | | | | | | | | | | | | | | | | | | | | | | | | | | | | | | | | | | | | | | | | | | | | | | | | | | | | | | | | | | | | | | | | | | | | | | | | | | | | | | | | | | | | | | | | | | | | | | | | | | | | | | | | | Ratio | | | | | | | | | | | | | | | | | | | | | | | | | | | | | | | | | | | | | | | |  |  |  |  |  |  |  |  |  |  |  |  |  |  |  |  |  |  |  |  |  |  |  |  |  |  |  |  |  |  |  |  |  |  |  |  |
|  |  |  |  |  |  |  |  |  |  |  |  |  |  |  |  |  |  |  |  |  |  |  |  |  |  |  |  |  |  |  |  |  |  |  |  |  |  |  |  |  |  |  |  |  |  |  |  |  |  |  |  |  |  |  |  |  |  |  |  |  |  |  |  |  |  |  |  |  |  |  |  |  |  |  |  |  |  |  |  |  |  |  |  |  |  |  |  |  |  |  |  |  |  |  |  |  |  |  |  |  |  |  |  |  |  |  |  |  |  |  |  |  |  |  |  |  |  |  |  |  |  |  |  |  |  |  |  |  |  |  |  |  |  |  |  |  |  |  |  |  |  |  |  |  |  |  |  |  |  |  |  |  |  |  |  |  |  |  |  |  |  |  |  |  |  |  |  |  |  |  |  |  |  |  |  |  |  |  |  |  |  |  |  |  |  |  |  |  |  |  |  |  |  |  |  |  |  |  |  |  |  | Theo. | | | | | | | | | | | | | | | | | | | | | | | | | | | | | | | | | | | | | | | | Exp. | | | | | | | | | | | | | | | | | | | | | | | | | | | | | | | | | | | | | | | | | | | | | | | | | | | | | | | | | | | | | | | | | | | | | | | | | | | | | | | | | | | | | | | | | |  |  |  |  |  |  |  |  |  |  |  |  |  |  |  |  |  |  |  |  |  |  |  |  |  |  |  |  |  |  |  |  |  |  |  |  |  |  |  |  |  |  |  |  |  |  |  |  |  |  |  |  |  |  |  |  |  |  |  |  |  |  |  |  |  |  |  |  |  |  |  |  |  |  |  |  |
| 1h | | | stem 31 kDa glycoprotein | | | | | | | | | | | | | | | | | | Glyma08g21410.1 | | | | | | | | | | | | | | | | | | | | | | | | | | | | | 100 | | | | | | | | | | | | | | | | | | | | | | | | | | | | | | | | | | | 1 | | | | | | | | | | | | | | | | | | | | | | | | | | | | | | | | | | | 5 | | | | | | | | | | | | | | | | | | | | | | | | | | | | | | | | | | | | | 500 | | | | | | | | | | | | | | | | | | | | | | | | | | | | | | | | | | | | | | | | | | | | | 29.7/6.71 | | | | | | | | | | | | | | | | | | | | | | | | | | | | | | | | | | | | | | | |  | | | | | | | | | | | | | | | | | | | | | | | | | | | | | | | | | | | | | | | | | | | | | | | | | | | | | | | | | | | | | | | | | | | | | | | | | | | | | | | | | | | | | | | | | |  | | | | | | | | | | | | | | | | | | | | | | | | | | | | | | | | | | | | | | | |  |  |  |  |  |  |  |  |  |  |  |  |  |  |  |  |  |  |  |  |  |  |  |  |  |  |  |  |  |  |  |  |  |  |  |  |
|  | | | Calc. Mass | | | | | | | | | | | | | | | | | | Observ. Mass | | | | | | | | | | | | | | | | | | | | | | | | | | | | | Da | | | | | | | | | | | | | | | | | | | | | | | | | | | | | | | | | | | ppm | | | | | | | | | | | | | | | | | | | | | | | | | | | | | | | | | | | start | | | | | | | | | | | | | | | | | | | | | | | | | | | | | | | | | | | | | end | | | | | | | | | | | | | | | | | | | | | | | | | | | | | | | | | | | | | | | | | | | | | sequence | | | | | | | | | | | | | | | | | | | | | | | | | | | | | | | | | | | | | | | | | | | | | | | | | | | | | | | | | | | | | | | | | | | | | | | | | | | | | | | | | | | | | | | | | | | | | | | | | | | | | | | | | | | | | | | | | | | | | | | | | | | | | | | | | | | | | | | | | | | | | | | | | | | | | | | | | | | | | | | | | | | | | | | | | |  |  |  |  |  |  |  |  |  |  |  |  |  |  |  |  |  |  |  |  |  |  |  |  |  |  |  |  |  |  |  |  |  |  |  |  |
|  | | | 1559.7368 | | | | | | | | | | | | | | | | | | 1559.736 | | | | | | | | | | | | | | | | | | | | | | | | | | | | | 0 | | | | | | | | | | | | | | | | | | | | | | | | | | | | | | | | | | | -1 | | | | | | | | | | | | | | | | | | | | | | | | | | | | | | | | | | | 84 | | | | | | | | | | | | | | | | | | | | | | | | | | | | | | | | | | | | | 96 | | | | | | | | | | | | | | | | | | | | | | | | | | | | | | | | | | | | | | | | | | | | | TVNQQAFFYASER | | | | | | | | | | | | | | | | | | | | | | | | | | | | | | | | | | | | | | | | | | | | | | | | | | | | | | | | | | | | | | | | | | | | | | | | | | | | | | | | | | | | | | | | | | | | | | | | | | | | | | | | | | | | | | | | | | | | | | | | | | | | | | | | | | | | | | | | | | | | | | | | | | | | | | | | | | | | | | | | | | | | | | | | | |  |  |  |  |  |  |  |  |  |  |  |  |  |  |  |  |  |  |  |  |  |  |  |  |  |  |  |  |  |  |  |  |  |  |  |  |
|  | | | 1559.7368 | | | | | | | | | | | | | | | | | | 1559.736 | | | | | | | | | | | | | | | | | | | | | | | | | | | | | 0 | | | | | | | | | | | | | | | | | | | | | | | | | | | | | | | | | | | -1 | | | | | | | | | | | | | | | | | | | | | | | | | | | | | | | | | | | 84 | | | | | | | | | | | | | | | | | | | | | | | | | | | | | | | | | | | | | 96 | | | | | | | | | | | | | | | | | | | | | | | | | | | | | | | | | | | | | | | | | | | | | TVNQQAFFYASER | | | | | | | | | | | | | | | | | | | | | | | | | | | | | | | | | | | | | | | | | | | | | | | | | | | | | | | | | | | | | | | | | | | | | | | | | | | | | | | | | | | | | | | | | | | | | | | | | | | | | | | | | | | | | | | | | | | | | | | | | | | | | | | | | | | | | | | | | | | | | | | | | | | | | | | | | | | | | | | | | | | | | | | | | |  |  |  |  |  |  |  |  |  |  |  |  |  |  |  |  |  |  |  |  |  |  |  |  |  |  |  |  |  |  |  |  |  |  |  |  |
| Spot no. | | | Homologous protein | | | | | | | | | | | | | | | | | | Accession no. | | | | | | | | | | | | | | | | | | | | | | | | | | | | | Score | | | | | | | | | | | | | | | | | | | | | | | | | | | | | | | | | | | M.P. | | | | | | | | | | | | | | | | | | | | | | | | | | | | | | | | | | | Cov. (%) | | | | | | | | | | | | | | | | | | | | | | | | | | | | | | | | | | | | | Blast score | | | | | | | | | | | | | | | | | | | | | | | | | | | | | | | | | | | | | | | | | | | | | Mr (kDa) / pI | | | | | | | | | | | | | | | | | | | | | | | | | | | | | | | | | | | | | | | | | | | | | | | | | | | | | | | | | | | | | | | | | | | | | | | | | | | | | | | | | | | | | | | | | | | | | | | | | | | | | | | | | | | | | | | | | | | | | | | | | | | | | | | | | | | | Ratio | | | | | | | | | | | | | | | | | | | | | | | | | | | | | | | | | | | | | |  |  |  |  |  |  |  |  |  |  |  |  |  |  |  |  |  |  |  |  |  |  |  |  |  |  |  |  |  |  |  |  |  |  |  |  |
|  |  |  |  |  |  |  |  |  |  |  |  |  |  |  |  |  |  |  |  |  |  |  |  |  |  |  |  |  |  |  |  |  |  |  |  |  |  |  |  |  |  |  |  |  |  |  |  |  |  |  |  |  |  |  |  |  |  |  |  |  |  |  |  |  |  |  |  |  |  |  |  |  |  |  |  |  |  |  |  |  |  |  |  |  |  |  |  |  |  |  |  |  |  |  |  |  |  |  |  |  |  |  |  |  |  |  |  |  |  |  |  |  |  |  |  |  |  |  |  |  |  |  |  |  |  |  |  |  |  |  |  |  |  |  |  |  |  |  |  |  |  |  |  |  |  |  |  |  |  |  |  |  |  |  |  |  |  |  |  |  |  |  |  |  |  |  |  |  |  |  |  |  |  |  |  |  |  |  |  |  |  |  |  |  |  |  |  |  |  |  |  |  |  |  |  |  |  |  |  |  |  | Theo. | | | | | | | | | | | | | | | | | | | | | | | | | | | | | | | | | | | | | | | | | | | | | | Exp. | | | | | | | | | | | | | | | | | | | | | | | | | | | | | | | | | | | | | | | | | | | | | | | | | | | | | | | | | | | | | | | | | | | | | | | | | | | | | | | | | | | | | |  |  |  |  |  |  |  |  |  |  |  |  |  |  |  |  |  |  |  |  |  |  |  |  |  |  |  |  |  |  |  |  |  |  |  |  |  |  |  |  |  |  |  |  |  |  |  |  |  |  |  |  |  |  |  |  |  |  |  |  |  |  |  |  |  |  |  |  |  |  |  |  |  |  |
| 2a | | | uncharacterized protein LOC100527731 | | | | | | | | | | | | | | | | | | Glyma07g37270.1 | | | | | | | | | | | | | | | | | | | | | | | | | | | | | 212 | | | | | | | | | | | | | | | | | | | | | | | | | | | | | | | | | | | 6 | | | | | | | | | | | | | | | | | | | | | | | | | | | | | | | | | | | 40 | | | | | | | | | | | | | | | | | | | | | | | | | | | | | | | | | | | | | 317 | | | | | | | | | | | | | | | | | | | | | | | | | | | | | | | | | | | | | | | | | | | | | 17.0/4.72 | | | | | | | | | | | | | | | | | | | | | | | | | | | | | | | | | | | | | | | | | | | | | |  | | | | | | | | | | | | | | | | | | | | | | | | | | | | | | | | | | | | | | | | | | | | | | | | | | | | | | | | | | | | | | | | | | | | | | | | | | | | | | | | | | | | | |  | | | | | | | | | | | | | | | | | | | | | | | | | | | | | | | | | | | | | |  |  |  |  |  |  |  |  |  |  |  |  |  |  |  |  |  |  |  |  |  |  |  |  |  |  |  |  |  |  |  |  |  |  |  |  |
|  | | | Calc. Mass | | | | | | | | | | | | | | | | | | Observ. Mass | | | | | | | | | | | | | | | | | | | | | | | | | | | | | Da | | | | | | | | | | | | | | | | | | | | | | | | | | | | | | | | | | | ppm | | | | | | | | | | | | | | | | | | | | | | | | | | | | | | | | | | | start | | | | | | | | | | | | | | | | | | | | | | | | | | | | | | | | | | | | | end | | | | | | | | | | | | | | | | | | | | | | | | | | | | | | | | | | | | | | | | | | | | | sequence | | | | | | | | | | | | | | | | | | | | | | | | | | | | | | | | | | | | | | | | | | | | | | | | | | | | | | | | | | | | | | | | | | | | | | | | | | | | | | | | | | | | | | | | | | | | | | | | | | | | | | | | | | | | | | | | | | | | | | | | | | | | | | | | | | | | | | | | | | | | | | | | | | | | | | | | | | | | | | | | | | | | | | | | | |  |  |  |  |  |  |  |  |  |  |  |  |  |  |  |  |  |  |  |  |  |  |  |  |  |  |  |  |  |  |  |  |  |  |  |  |
|  | | | 2172.0627 | | | | | | | | | | | | | | | | | | 2172.063 | | | | | | | | | | | | | | | | | | | | | | | | | | | | | 0 | | | | | | | | | | | | | | | | | | | | | | | | | | | | | | | | | | | 0 | | | | | | | | | | | | | | | | | | | | | | | | | | | | | | | | | | | 2 | | | | | | | | | | | | | | | | | | | | | | | | | | | | | | | | | | | | | 21 | | | | | | | | | | | | | | | | | | | | | | | | | | | | | | | | | | | | | | | | | | | | | GVFTFEDETTSPVAPATLYK | | | | | | | | | | | | | | | | | | | | | | | | | | | | | | | | | | | | | | | | | | | | | | | | | | | | | | | | | | | | | | | | | | | | | | | | | | | | | | | | | | | | | | | | | | | | | | | | | | | | | | | | | | | | | | | | | | | | | | | | | | | | | | | | | | | | | | | | | | | | | | | | | | | | | | | | | | | | | | | | | | | | | | | | | |  |  |  |  |  |  |  |  |  |  |  |  |  |  |  |  |  |  |  |  |  |  |  |  |  |  |  |  |  |  |  |  |  |  |  |  |
|  | | | 1254.682 | | | | | | | | | | | | | | | | | | 1254.682 | | | | | | | | | | | | | | | | | | | | | | | | | | | | | 0 | | | | | | | | | | | | | | | | | | | | | | | | | | | | | | | | | | | 0 | | | | | | | | | | | | | | | | | | | | | | | | | | | | | | | | | | | 22 | | | | | | | | | | | | | | | | | | | | | | | | | | | | | | | | | | | | | 33 | | | | | | | | | | | | | | | | | | | | | | | | | | | | | | | | | | | | | | | | | | | | | ALVTDADNVIPK | | | | | | | | | | | | | | | | | | | | | | | | | | | | | | | | | | | | | | | | | | | | | | | | | | | | | | | | | | | | | | | | | | | | | | | | | | | | | | | | | | | | | | | | | | | | | | | | | | | | | | | | | | | | | | | | | | | | | | | | | | | | | | | | | | | | | | | | | | | | | | | | | | | | | | | | | | | | | | | | | | | | | | | | | |  |  |  |  |  |  |  |  |  |  |  |  |  |  |  |  |  |  |  |  |  |  |  |  |  |  |  |  |  |  |  |  |  |  |  |  |
|  | | | 1254.682 | | | | | | | | | | | | | | | | | | 1254.682 | | | | | | | | | | | | | | | | | | | | | | | | | | | | | 0 | | | | | | | | | | | | | | | | | | | | | | | | | | | | | | | | | | | 0 | | | | | | | | | | | | | | | | | | | | | | | | | | | | | | | | | | | 22 | | | | | | | | | | | | | | | | | | | | | | | | | | | | | | | | | | | | | 33 | | | | | | | | | | | | | | | | | | | | | | | | | | | | | | | | | | | | | | | | | | | | | ALVTDADNVIPK | | | | | | | | | | | | | | | | | | | | | | | | | | | | | | | | | | | | | | | | | | | | | | | | | | | | | | | | | | | | | | | | | | | | | | | | | | | | | | | | | | | | | | | | | | | | | | | | | | | | | | | | | | | | | | | | | | | | | | | | | | | | | | | | | | | | | | | | | | | | | | | | | | | | | | | | | | | | | | | | | | | | | | | | | |  |  |  |  |  |  |  |  |  |  |  |  |  |  |  |  |  |  |  |  |  |  |  |  |  |  |  |  |  |  |  |  |  |  |  |  |
|  | | | 1254.682 | | | | | | | | | | | | | | | | | | 1254.682 | | | | | | | | | | | | | | | | | | | | | | | | | | | | | 0 | | | | | | | | | | | | | | | | | | | | | | | | | | | | | | | | | | | 0 | | | | | | | | | | | | | | | | | | | | | | | | | | | | | | | | | | | 22 | | | | | | | | | | | | | | | | | | | | | | | | | | | | | | | | | | | | | 33 | | | | | | | | | | | | | | | | | | | | | | | | | | | | | | | | | | | | | | | | | | | | | ALVTDADNVIPK | | | | | | | | | | | | | | | | | | | | | | | | | | | | | | | | | | | | | | | | | | | | | | | | | | | | | | | | | | | | | | | | | | | | | | | | | | | | | | | | | | | | | | | | | | | | | | | | | | | | | | | | | | | | | | | | | | | | | | | | | | | | | | | | | | | | | | | | | | | | | | | | | | | | | | | | | | | | | | | | | | | | | | | | | |  |  |  |  |  |  |  |  |  |  |  |  |  |  |  |  |  |  |  |  |  |  |  |  |  |  |  |  |  |  |  |  |  |  |  |  |
|  | | | 1914.0211 | | | | | | | | | | | | | | | | | | 1914.021 | | | | | | | | | | | | | | | | | | | | | | | | | | | | | 0 | | | | | | | | | | | | | | | | | | | | | | | | | | | | | | | | | | | 0 | | | | | | | | | | | | | | | | | | | | | | | | | | | | | | | | | | | 22 | | | | | | | | | | | | | | | | | | | | | | | | | | | | | | | | | | | | | 39 | | | | | | | | | | | | | | | | | | | | | | | | | | | | | | | | | | | | | | | | | | | | | ALVTDADNVIPKAVDAFR | | | | | | | | | | | | | | | | | | | | | | | | | | | | | | | | | | | | | | | | | | | | | | | | | | | | | | | | | | | | | | | | | | | | | | | | | | | | | | | | | | | | | | | | | | | | | | | | | | | | | | | | | | | | | | | | | | | | | | | | | | | | | | | | | | | | | | | | | | | | | | | | | | | | | | | | | | | | | | | | | | | | | | | | | |  |  |  |  |  |  |  |  |  |  |  |  |  |  |  |  |  |  |  |  |  |  |  |  |  |  |  |  |  |  |  |  |  |  |  |  |
|  | | | 1279.666 | | | | | | | | | | | | | | | | | | 1279.666 | | | | | | | | | | | | | | | | | | | | | | | | | | | | | 0 | | | | | | | | | | | | | | | | | | | | | | | | | | | | | | | | | | | 0 | | | | | | | | | | | | | | | | | | | | | | | | | | | | | | | | | | | 55 | | | | | | | | | | | | | | | | | | | | | | | | | | | | | | | | | | | | | 65 | | | | | | | | | | | | | | | | | | | | | | | | | | | | | | | | | | | | | | | | | | | | | KITFLEDGETK | | | | | | | | | | | | | | | | | | | | | | | | | | | | | | | | | | | | | | | | | | | | | | | | | | | | | | | | | | | | | | | | | | | | | | | | | | | | | | | | | | | | | | | | | | | | | | | | | | | | | | | | | | | | | | | | | | | | | | | | | | | | | | | | | | | | | | | | | | | | | | | | | | | | | | | | | | | | | | | | | | | | | | | | | |  |  |  |  |  |  |  |  |  |  |  |  |  |  |  |  |  |  |  |  |  |  |  |  |  |  |  |  |  |  |  |  |  |  |  |  |
|  | | | 1279.666 | | | | | | | | | | | | | | | | | | 1279.668 | | | | | | | | | | | | | | | | | | | | | | | | | | | | | 0 | | | | | | | | | | | | | | | | | | | | | | | | | | | | | | | | | | | 1 | | | | | | | | | | | | | | | | | | | | | | | | | | | | | | | | | | | 55 | | | | | | | | | | | | | | | | | | | | | | | | | | | | | | | | | | | | | 65 | | | | | | | | | | | | | | | | | | | | | | | | | | | | | | | | | | | | | | | | | | | | | KITFLEDGETK | | | | | | | | | | | | | | | | | | | | | | | | | | | | | | | | | | | | | | | | | | | | | | | | | | | | | | | | | | | | | | | | | | | | | | | | | | | | | | | | | | | | | | | | | | | | | | | | | | | | | | | | | | | | | | | | | | | | | | | | | | | | | | | | | | | | | | | | | | | | | | | | | | | | | | | | | | | | | | | | | | | | | | | | | |  |  |  |  |  |  |  |  |  |  |  |  |  |  |  |  |  |  |  |  |  |  |  |  |  |  |  |  |  |  |  |  |  |  |  |  |
|  | | | 1151.571 | | | | | | | | | | | | | | | | | | 1151.57 | | | | | | | | | | | | | | | | | | | | | | | | | | | | | 0 | | | | | | | | | | | | | | | | | | | | | | | | | | | | | | | | | | | -1 | | | | | | | | | | | | | | | | | | | | | | | | | | | | | | | | | | | 56 | | | | | | | | | | | | | | | | | | | | | | | | | | | | | | | | | | | | | 65 | | | | | | | | | | | | | | | | | | | | | | | | | | | | | | | | | | | | | | | | | | | | | ITFLEDGETK | | | | | | | | | | | | | | | | | | | | | | | | | | | | | | | | | | | | | | | | | | | | | | | | | | | | | | | | | | | | | | | | | | | | | | | | | | | | | | | | | | | | | | | | | | | | | | | | | | | | | | | | | | | | | | | | | | | | | | | | | | | | | | | | | | | | | | | | | | | | | | | | | | | | | | | | | | | | | | | | | | | | | | | | | |  |  |  |  |  |  |  |  |  |  |  |  |  |  |  |  |  |  |  |  |  |  |  |  |  |  |  |  |  |  |  |  |  |  |  |  |
|  | | | 1413.794 | | | | | | | | | | | | | | | | | | 1413.794 | | | | | | | | | | | | | | | | | | | | | | | | | | | | | 0 | | | | | | | | | | | | | | | | | | | | | | | | | | | | | | | | | | | 0 | | | | | | | | | | | | | | | | | | | | | | | | | | | | | | | | | | | 104 | | | | | | | | | | | | | | | | | | | | | | | | | | | | | | | | | | | | | 119 | | | | | | | | | | | | | | | | | | | | | | | | | | | | | | | | | | | | | | | | | | | | | LAAGANGGSAGKLTVK | | | | | | | | | | | | | | | | | | | | | | | | | | | | | | | | | | | | | | | | | | | | | | | | | | | | | | | | | | | | | | | | | | | | | | | | | | | | | | | | | | | | | | | | | | | | | | | | | | | | | | | | | | | | | | | | | | | | | | | | | | | | | | | | | | | | | | | | | | | | | | | | | | | | | | | | | | | | | | | | | | | | | | | | | |  |  |  |  |  |  |  |  |  |  |  |  |  |  |  |  |  |  |  |  |  |  |  |  |  |  |  |  |  |  |  |  |  |  |  |  |
| Spot no. | | | | | | | Homologous protein | | | | | | | | | | | | | | | | | | Accession no. | | | | | | | | | | | | | | | | | | | | | | | | | | | | | | | | Score | | | | | | | | | | | | | | | | | | | | | | | | | | | | | | M.P. | | | | | | | | | | | | | | | | | | | | | | | | | | | | | | | | | | | Cov. (%) | | | | | | | | | | | | | | | | | | | | | | | | | | | | | | | | | | | | Blast score | | | | | | | | | | | | | | | | | | | | | | | | | | | | | | | | | | | | | | | | | | | | Mr (kDa) / pI | | | | | | | | | | | | | | | | | | | | | | | | | | | | | | | | | | | | | | | | | | | | | | | | | | | | | | | | | | | | | | | | | | | | | | | | | | | | | | | | | | | | | | | | | | | | | | | | | | | | | | | | | | | | | | | | | | | | Ratio | | | | | | | | | | | | | | | | | | | | | | | | | | | | | | | | | | | | | | | | | | | | | | | | | | | | | | | | | | | | | |  |  |  |  |  |  |  |  |  |  |  |  |  |  |  |  |  |  |  |  |  |  |  |  |  |  |  |  |
|  |  |  |  |  |  |  |  |  |  |  |  |  |  |  |  |  |  |  |  |  |  |  |  |  |  |  |  |  |  |  |  |  |  |  |  |  |  |  |  |  |  |  |  |  |  |  |  |  |  |  |  |  |  |  |  |  |  |  |  |  |  |  |  |  |  |  |  |  |  |  |  |  |  |  |  |  |  |  |  |  |  |  |  |  |  |  |  |  |  |  |  |  |  |  |  |  |  |  |  |  |  |  |  |  |  |  |  |  |  |  |  |  |  |  |  |  |  |  |  |  |  |  |  |  |  |  |  |  |  |  |  |  |  |  |  |  |  |  |  |  |  |  |  |  |  |  |  |  |  |  |  |  |  |  |  |  |  |  |  |  |  |  |  |  |  |  |  |  |  |  |  |  |  |  |  |  |  |  |  |  |  |  |  |  |  |  |  |  |  |  |  |  |  |  |  |  |  |  |  |  |  | Theo. | | | | | | | | | | | | | | | | | | | | | | | | | | | | | | | | | | | | | | | | | | Exp. | | | | | | | | | | | | | | | | | | | | | | | | | | | | | | | | | | | | | | | | | | | | | | | | | | | | | | | | | | | | | | | | | | | | | | | | | |  |  |  |  |  |  |  |  |  |  |  |  |  |  |  |  |  |  |  |  |  |  |  |  |  |  |  |  |  |  |  |  |  |  |  |  |  |  |  |  |  |  |  |  |  |  |  |  |  |  |  |  |  |  |  |  |  |  |  |  |  |  |  |  |  |  |  |  |  |  |  |  |  |  |  |  |  |  |  |  |  |  |  |  |  |  |  |  |  |  |
| 2b | | | | | | | Phosphoglycerate kinase | | | | | | | | | | | | | | | | | | Glyma08g17600.1 | | | | | | | | | | | | | | | | | | | | | | | | | | | | | | | | 156 | | | | | | | | | | | | | | | | | | | | | | | | | | | | | | 3 | | | | | | | | | | | | | | | | | | | | | | | | | | | | | | | | | | | 14 | | | | | | | | | | | | | | | | | | | | | | | | | | | | | | | | | | | | 735 | | | | | | | | | | | | | | | | | | | | | | | | | | | | | | | | | | | | | | | | | | | | 42.6/6.28 | | | | | | | | | | | | | | | | | | | | | | | | | | | | | | | | | | | | | | | | | |  | | | | | | | | | | | | | | | | | | | | | | | | | | | | | | | | | | | | | | | | | | | | | | | | | | | | | | | | | | | | | | | | | | | | | | | | | |  | | | | | | | | | | | | | | | | | | | | | | | | | | | | | | | | | | | | | | | | | | | | | | | | | | | | | | | | | | | | | |  |  |  |  |  |  |  |  |  |  |  |  |  |  |  |  |  |  |  |  |  |  |  |  |  |  |  |  |
|  | | | | | | | Calc. Mass | | | | | | | | | | | | | | | | | | Observ. Mass | | | | | | | | | | | | | | | | | | | | | | | | | | | | | | | | Da | | | | | | | | | | | | | | | | | | | | | | | | | | | | | | ppm | | | | | | | | | | | | | | | | | | | | | | | | | | | | | | | | | | | start | | | | | | | | | | | | | | | | | | | | | | | | | | | | | | | | | | | | end | | | | | | | | | | | | | | | | | | | | | | | | | | | | | | | | | | | | | | | | | | | | sequence | | | | | | | | | | | | | | | | | | | | | | | | | | | | | | | | | | | | | | | | | | | | | | | | | | | | | | | | | | | | | | | | | | | | | | | | | | | | | | | | | | | | | | | | | | | | | | | | | | | | | | | | | | | | | | | | | | | | | | | | | | | | | | | | | | | | | | | | | | | | | | | | | | | | | | | | | | | | | | | | | | | | | | | | | | | | | | | | | |  |  |  |  |  |  |  |  |  |  |  |  |  |  |  |  |  |  |  |  |  |  |  |  |  |  |  |  |
|  | | | | | | | 2040.9964 | | | | | | | | | | | | | | | | | | 2040.989 | | | | | | | | | | | | | | | | | | | | | | | | | | | | | | | | 0 | | | | | | | | | | | | | | | | | | | | | | | | | | | | | | -3 | | | | | | | | | | | | | | | | | | | | | | | | | | | | | | | | | | | 24 | | | | | | | | | | | | | | | | | | | | | | | | | | | | | | | | | | | | 41 | | | | | | | | | | | | | | | | | | | | | | | | | | | | | | | | | | | | | | | | | | | | VDLNVPLDDNLNITDDTR | | | | | | | | | | | | | | | | | | | | | | | | | | | | | | | | | | | | | | | | | | | | | | | | | | | | | | | | | | | | | | | | | | | | | | | | | | | | | | | | | | | | | | | | | | | | | | | | | | | | | | | | | | | | | | | | | | | | | | | | | | | | | | | | | | | | | | | | | | | | | | | | | | | | | | | | | | | | | | | | | | | | | | | | | | | | | | | | | |  |  |  |  |  |  |  |  |  |  |  |  |  |  |  |  |  |  |  |  |  |  |  |  |  |  |  |  |
|  | | | | | | | 2637.3749 | | | | | | | | | | | | | | | | | | 2637.38 | | | | | | | | | | | | | | | | | | | | | | | | | | | | | | | | 0 | | | | | | | | | | | | | | | | | | | | | | | | | | | | | | 2 | | | | | | | | | | | | | | | | | | | | | | | | | | | | | | | | | | | 233 | | | | | | | | | | | | | | | | | | | | | | | | | | | | | | | | | | | | 257 | | | | | | | | | | | | | | | | | | | | | | | | | | | | | | | | | | | | | | | | | | | | AQGYSVGSSLVEEDKLSLATTLLEK | | | | | | | | | | | | | | | | | | | | | | | | | | | | | | | | | | | | | | | | | | | | | | | | | | | | | | | | | | | | | | | | | | | | | | | | | | | | | | | | | | | | | | | | | | | | | | | | | | | | | | | | | | | | | | | | | | | | | | | | | | | | | | | | | | | | | | | | | | | | | | | | | | | | | | | | | | | | | | | | | | | | | | | | | | | | | | | | | |  |  |  |  |  |  |  |  |  |  |  |  |  |  |  |  |  |  |  |  |  |  |  |  |  |  |  |  |
|  | | | | | | | 1572.8359 | | | | | | | | | | | | | | | | | | 1572.833 | | | | | | | | | | | | | | | | | | | | | | | | | | | | | | | | 0 | | | | | | | | | | | | | | | | | | | | | | | | | | | | | | -2 | | | | | | | | | | | | | | | | | | | | | | | | | | | | | | | | | | | 350 | | | | | | | | | | | | | | | | | | | | | | | | | | | | | | | | | | | | 366 | | | | | | | | | | | | | | | | | | | | | | | | | | | | | | | | | | | | | | | | | | | | GVTTIIGGGDSVAAVEK | | | | | | | | | | | | | | | | | | | | | | | | | | | | | | | | | | | | | | | | | | | | | | | | | | | | | | | | | | | | | | | | | | | | | | | | | | | | | | | | | | | | | | | | | | | | | | | | | | | | | | | | | | | | | | | | | | | | | | | | | | | | | | | | | | | | | | | | | | | | | | | | | | | | | | | | | | | | | | | | | | | | | | | | | | | | | | | | | |  |  |  |  |  |  |  |  |  |  |  |  |  |  |  |  |  |  |  |  |  |  |  |  |  |  |  |  |
| Spot no. | | | | | | | Homologous protein | | | | | | | | | | | | | | | | | | Accession no. | | | | | | | | | | | | | | | | | | | | | | | | | | | | | | | | Score | | | | | | | | | | | | | | | | | | | | | | | | | | | | | | M.P. | | | | | | | | | | | | | | | | | | | | | | | | | | | | | | | | | | | Cov. (%) | | | | | | | | | | | | | | | | | | | | | | | | | | | | | | | | | | | | Blast score | | | | | | | | | | | | | | | | | | | | | | | | | | | | | | | | | | | | | | | | | | | | Mr (kDa) / pI | | | | | | | | | | | | | | | | | | | | | | | | | | | | | | | | | | | | | | | | | | | | | | | | | | | | | | | | | | | | | | | | | | | | | | | | | | | | | | | | | | | | | | | | | | | | | | | | | | | | | | | | | | | | | | | | | | | | | | | | | | | | | | | | | | | | Ratio | | | | | | | | | | | | | | | | | | | | | | | | | | | | | | | | | | | | | | | | | | | | | |  |  |  |  |  |  |  |  |  |  |  |  |  |  |  |  |  |  |  |  |  |  |  |  |  |  |  |  |
|  |  |  |  |  |  |  |  |  |  |  |  |  |  |  |  |  |  |  |  |  |  |  |  |  |  |  |  |  |  |  |  |  |  |  |  |  |  |  |  |  |  |  |  |  |  |  |  |  |  |  |  |  |  |  |  |  |  |  |  |  |  |  |  |  |  |  |  |  |  |  |  |  |  |  |  |  |  |  |  |  |  |  |  |  |  |  |  |  |  |  |  |  |  |  |  |  |  |  |  |  |  |  |  |  |  |  |  |  |  |  |  |  |  |  |  |  |  |  |  |  |  |  |  |  |  |  |  |  |  |  |  |  |  |  |  |  |  |  |  |  |  |  |  |  |  |  |  |  |  |  |  |  |  |  |  |  |  |  |  |  |  |  |  |  |  |  |  |  |  |  |  |  |  |  |  |  |  |  |  |  |  |  |  |  |  |  |  |  |  |  |  |  |  |  |  |  |  |  |  |  |  | Theo. | | | | | | | | | | | | | | | | | | | | | | | | | | | | | | | | | | | | | | | | Exp. | | | | | | | | | | | | | | | | | | | | | | | | | | | | | | | | | | | | | | | | | | | | | | | | | | | | | | | | | | | | | | | | | | | | | | | | | | | | | | | | | | | | | | | | | | | |  |  |  |  |  |  |  |  |  |  |  |  |  |  |  |  |  |  |  |  |  |  |  |  |  |  |  |  |  |  |  |  |  |  |  |  |  |  |  |  |  |  |  |  |  |  |  |  |  |  |  |  |  |  |  |  |  |  |  |  |  |  |  |  |  |  |  |  |  |  |  |  |  |  |
| 2c | | | | | | | enolase | | | | | | | | | | | | | | | | | | Glyma03g34830.1 | | | | | | | | | | | | | | | | | | | | | | | | | | | | | | | | 153 | | | | | | | | | | | | | | | | | | | | | | | | | | | | | | 1 | | | | | | | | | | | | | | | | | | | | | | | | | | | | | | | | | | | 4 | | | | | | | | | | | | | | | | | | | | | | | | | | | | | | | | | | | | 863 | | | | | | | | | | | | | | | | | | | | | | | | | | | | | | | | | | | | | | | | | | | | 48.1/5.49 | | | | | | | | | | | | | | | | | | | | | | | | | | | | | | | | | | | | | | | |  | | | | | | | | | | | | | | | | | | | | | | | | | | | | | | | | | | | | | | | | | | | | | | | | | | | | | | | | | | | | | | | | | | | | | | | | | | | | | | | | | | | | | | | | | | | |  | | | | | | | | | | | | | | | | | | | | | | | | | | | | | | | | | | | | | | | | | | | | | |  |  |  |  |  |  |  |  |  |  |  |  |  |  |  |  |  |  |  |  |  |  |  |  |  |  |  |  |
|  | | | | | | | Calc. Mass | | | | | | | | | | | | | | | | | | Observ. Mass | | | | | | | | | | | | | | | | | | | | | | | | | | | | | | | | Da | | | | | | | | | | | | | | | | | | | | | | | | | | | | | | ppm | | | | | | | | | | | | | | | | | | | | | | | | | | | | | | | | | | | start | | | | | | | | | | | | | | | | | | | | | | | | | | | | | | | | | | | | end | | | | | | | | | | | | | | | | | | | | | | | | | | | | | | | | | | | | | | | | | | | | sequence | | | | | | | | | | | | | | | | | | | | | | | | | | | | | | | | | | | | | | | | | | | | | | | | | | | | | | | | | | | | | | | | | | | | | | | | | | | | | | | | | | | | | | | | | | | | | | | | | | | | | | | | | | | | | | | | | | | | | | | | | | | | | | | | | | | | | | | | | | | | | | | | | | | | | | | | | | | | | | | | | | | | | | | | | | | | | | | | | |  |  |  |  |  |  |  |  |  |  |  |  |  |  |  |  |  |  |  |  |  |  |  |  |  |  |  |  |
|  | | | | | | | 2251.122 | | | | | | | | | | | | | | | | | | 2251.119 | | | | | | | | | | | | | | | | | | | | | | | | | | | | | | | | 0 | | | | | | | | | | | | | | | | | | | | | | | | | | | | | | -1 | | | | | | | | | | | | | | | | | | | | | | | | | | | | | | | | | | | 382 | | | | | | | | | | | | | | | | | | | | | | | | | | | | | | | | | | | | 403 | | | | | | | | | | | | | | | | | | | | | | | | | | | | | | | | | | | | | | | | | | | | SGETEDTFIADLSVGLATGQIK | | | | | | | | | | | | | | | | | | | | | | | | | | | | | | | | | | | | | | | | | | | | | | | | | | | | | | | | | | | | | | | | | | | | | | | | | | | | | | | | | | | | | | | | | | | | | | | | | | | | | | | | | | | | | | | | | | | | | | | | | | | | | | | | | | | | | | | | | | | | | | | | | | | | | | | | | | | | | | | | | | | | | | | | | | | | | | | | | |  |  |  |  |  |  |  |  |  |  |  |  |  |  |  |  |  |  |  |  |  |  |  |  |  |  |  |  |
| Spot no. | | | | | | | Homologous protein | | | | | | | | | | | | | | | | | | | | | | | | | Accession no. | | | | | | | | | | | | | | | | | | | | | | | | | | | | | | | | Score | | | | | | | | | | | | | | | | | | | | | | | | | | | | | M.P. | | | | | | | | | | | | | | | | | | | | | | | | | | | | | | | | | | | | | Cov. (%) | | | | | | | | | | | | | | | | | | | | | | | | | | | | | | | | | | | | | | | Blast score | | | | | | | | | | | | | | | | | | | | | | | | | | | | | | | | | | | | | | | | | | Mr (kDa) / pI | | | | | | | | | | | | | | | | | | | | | | | | | | | | | | | | | | | | | | | | | | | | | | | | | | | | | | | | | | | | | | | | | | | | | | | | | | | | | | | | | | | | | | | | | | | | | | | | | | | | | | | | | | | | | | | | | | | | | | | | | | | | | | Ratio | | | | | | | | | | | | | | | | | | | | | | | | | | | | | | | | | | | | | | | | | | |  |  |  |  |  |  |  |  |  |  |  |  |  |  |  |  |  |  |  |  |  |  |  |  |  |  |  |  |
|  |  |  |  |  |  |  |  |  |  |  |  |  |  |  |  |  |  |  |  |  |  |  |  |  |  |  |  |  |  |  |  |  |  |  |  |  |  |  |  |  |  |  |  |  |  |  |  |  |  |  |  |  |  |  |  |  |  |  |  |  |  |  |  |  |  |  |  |  |  |  |  |  |  |  |  |  |  |  |  |  |  |  |  |  |  |  |  |  |  |  |  |  |  |  |  |  |  |  |  |  |  |  |  |  |  |  |  |  |  |  |  |  |  |  |  |  |  |  |  |  |  |  |  |  |  |  |  |  |  |  |  |  |  |  |  |  |  |  |  |  |  |  |  |  |  |  |  |  |  |  |  |  |  |  |  |  |  |  |  |  |  |  |  |  |  |  |  |  |  |  |  |  |  |  |  |  |  |  |  |  |  |  |  |  |  |  |  |  |  |  |  |  |  |  |  |  |  |  |  |  |  |  |  |  |  |  |  |  |  |  | Theo. | | | | | | | | | | | | | | | | | | | | | | | | | | | | | | | | | | | | | | | | | | | | | | | | | | | | | | | | | | | | | | | Exp. | | | | | | | | | | | | | | | | | | | | | | | | | | | | | | | | | | | | | | | | | | | | | | | | | | | | | | | | | | | | | | |  |  |  |  |  |  |  |  |  |  |  |  |  |  |  |  |  |  |  |  |  |  |  |  |  |  |  |  |  |  |  |  |  |  |  |  |  |  |  |  |  |  |  |  |  |  |  |  |  |  |  |  |  |  |  |  |  |  |  |  |  |  |  |  |  |  |  |  |  |  |  |
| 2d | | | | | | | uncharacterized protein LOC100499665 | | | | | | | | | | | | | | | | | | | | | | | | | Glyma03g40110.1 | | | | | | | | | | | | | | | | | | | | | | | | | | | | | | | | 147 | | | | | | | | | | | | | | | | | | | | | | | | | | | | | 3 | | | | | | | | | | | | | | | | | | | | | | | | | | | | | | | | | | | | | 22 | | | | | | | | | | | | | | | | | | | | | | | | | | | | | | | | | | | | | | | 243 | | | | | | | | | | | | | | | | | | | | | | | | | | | | | | | | | | | | | | | | | | 16.6/10.87 | | | | | | | | | | | | | | | | | | | | | | | | | | | | | | | | | | | | | | | | | | | | | | | | | | | | | | | | | | | | | | |  | | | | | | | | | | | | | | | | | | | | | | | | | | | | | | | | | | | | | | | | | | | | | | | | | | | | | | | | | | | | | | |  | | | | | | | | | | | | | | | | | | | | | | | | | | | | | | | | | | | | | | | | | | |  |  |  |  |  |  |  |  |  |  |  |  |  |  |  |  |  |  |  |  |  |  |  |  |  |  |  |  |
|  | | | | | | | Calc. Mass | | | | | | | | | | | | | | | | | | | | | | | | | Observ. Mass | | | | | | | | | | | | | | | | | | | | | | | | | | | | | | | | Da | | | | | | | | | | | | | | | | | | | | | | | | | | | | | ppm | | | | | | | | | | | | | | | | | | | | | | | | | | | | | | | | | | | | | start | | | | | | | | | | | | | | | | | | | | | | | | | | | | | | | | | | | | | | | end | | | | | | | | | | | | | | | | | | | | | | | | | | | | | | | | | | | | | | | | | | sequence | | | | | | | | | | | | | | | | | | | | | | | | | | | | | | | | | | | | | | | | | | | | | | | | | | | | | | | | | | | | | | | | | | | | | | | | | | | | | | | | | | | | | | | | | | | | | | | | | | | | | | | | | | | | | | | | | | | | | | | | | | | | | | | | | | | | | | | | | | | | | | | | | | | | | | | | | | | | | | | | | | | | | | | | |  |  |  |  |  |  |  |  |  |  |  |  |  |  |  |  |  |  |  |  |  |  |  |  |  |  |  |  |
|  | | | | | | | 1880.8574 | | | | | | | | | | | | | | | | | | | | | | | | | 1880.853 | | | | | | | | | | | | | | | | | | | | | | | | | | | | | | | | 0 | | | | | | | | | | | | | | | | | | | | | | | | | | | | | -2 | | | | | | | | | | | | | | | | | | | | | | | | | | | | | | | | | | | | | 66 | | | | | | | | | | | | | | | | | | | | | | | | | | | | | | | | | | | | | | | 83 | | | | | | | | | | | | | | | | | | | | | | | | | | | | | | | | | | | | | | | | | | DESSPYAAMLAAQDVAAR | | | | | | | | | | | | | | | | | | | | | | | | | | | | | | | | | | | | | | | | | | | | | | | | | | | | | | | | | | | | | | | | | | | | | | | | | | | | | | | | | | | | | | | | | | | | | | | | | | | | | | | | | | | | | | | | | | | | | | | | | | | | | | | | | | | | | | | | | | | | | | | | | | | | | | | | | | | | | | | | | | | | | | | | |  |  |  |  |  |  |  |  |  |  |  |  |  |  |  |  |  |  |  |  |  |  |  |  |  |  |  |  |
|  | | | | | | | 1880.8574 | | | | | | | | | | | | | | | | | | | | | | | | | 1880.856 | | | | | | | | | | | | | | | | | | | | | | | | | | | | | | | | 0 | | | | | | | | | | | | | | | | | | | | | | | | | | | | | -1 | | | | | | | | | | | | | | | | | | | | | | | | | | | | | | | | | | | | | 66 | | | | | | | | | | | | | | | | | | | | | | | | | | | | | | | | | | | | | | | 83 | | | | | | | | | | | | | | | | | | | | | | | | | | | | | | | | | | | | | | | | | | DESSPYAAMLAAQDVAAR | | | | | | | | | | | | | | | | | | | | | | | | | | | | | | | | | | | | | | | | | | | | | | | | | | | | | | | | | | | | | | | | | | | | | | | | | | | | | | | | | | | | | | | | | | | | | | | | | | | | | | | | | | | | | | | | | | | | | | | | | | | | | | | | | | | | | | | | | | | | | | | | | | | | | | | | | | | | | | | | | | | | | | | | |  |  |  |  |  |  |  |  |  |  |  |  |  |  |  |  |  |  |  |  |  |  |  |  |  |  |  |  |
|  | | | | | | | 1754.9163 | | | | | | | | | | | | | | | | | | | | | | | | | 1754.916 | | | | | | | | | | | | | | | | | | | | | | | | | | | | | | | | 0 | | | | | | | | | | | | | | | | | | | | | | | | | | | | | 0 | | | | | | | | | | | | | | | | | | | | | | | | | | | | | | | | | | | | | 125 | | | | | | | | | | | | | | | | | | | | | | | | | | | | | | | | | | | | | | | 140 | | | | | | | | | | | | | | | | | | | | | | | | | | | | | | | | | | | | | | | | | | IGRIEDVTPIPSDSTR | | | | | | | | | | | | | | | | | | | | | | | | | | | | | | | | | | | | | | | | | | | | | | | | | | | | | | | | | | | | | | | | | | | | | | | | | | | | | | | | | | | | | | | | | | | | | | | | | | | | | | | | | | | | | | | | | | | | | | | | | | | | | | | | | | | | | | | | | | | | | | | | | | | | | | | | | | | | | | | | | | | | | | | | |  |  |  |  |  |  |  |  |  |  |  |  |  |  |  |  |  |  |  |  |  |  |  |  |  |  |  |  |
|  | | | | | | | 1428.7096 | | | | | | | | | | | | | | | | | | | | | | | | | 1428.704 | | | | | | | | | | | | | | | | | | | | | | | | | | | | | | | | 0 | | | | | | | | | | | | | | | | | | | | | | | | | | | | | -4 | | | | | | | | | | | | | | | | | | | | | | | | | | | | | | | | | | | | | 128 | | | | | | | | | | | | | | | | | | | | | | | | | | | | | | | | | | | | | | | 140 | | | | | | | | | | | | | | | | | | | | | | | | | | | | | | | | | | | | | | | | | | IEDVTPIPSDSTR | | | | | | | | | | | | | | | | | | | | | | | | | | | | | | | | | | | | | | | | | | | | | | | | | | | | | | | | | | | | | | | | | | | | | | | | | | | | | | | | | | | | | | | | | | | | | | | | | | | | | | | | | | | | | | | | | | | | | | | | | | | | | | | | | | | | | | | | | | | | | | | | | | | | | | | | | | | | | | | | | | | | | | | | |  |  |  |  |  |  |  |  |  |  |  |  |  |  |  |  |  |  |  |  |  |  |  |  |  |  |  |  |
|  | | | | | | | 1428.7096 | | | | | | | | | | | | | | | | | | | | | | | | | 1428.707 | | | | | | | | | | | | | | | | | | | | | | | | | | | | | | | | 0 | | | | | | | | | | | | | | | | | | | | | | | | | | | | | -2 | | | | | | | | | | | | | | | | | | | | | | | | | | | | | | | | | | | | | 128 | | | | | | | | | | | | | | | | | | | | | | | | | | | | | | | | | | | | | | | 140 | | | | | | | | | | | | | | | | | | | | | | | | | | | | | | | | | | | | | | | | | | IEDVTPIPSDSTR | | | | | | | | | | | | | | | | | | | | | | | | | | | | | | | | | | | | | | | | | | | | | | | | | | | | | | | | | | | | | | | | | | | | | | | | | | | | | | | | | | | | | | | | | | | | | | | | | | | | | | | | | | | | | | | | | | | | | | | | | | | | | | | | | | | | | | | | | | | | | | | | | | | | | | | | | | | | | | | | | | | | | | | | |  |  |  |  |  |  |  |  |  |  |  |  |  |  |  |  |  |  |  |  |  |  |  |  |  |  |  |  |
| Spot no. | | | | | | | Homologous protein | | | | | | | | | | | | | | | | | | | | | | | | | Accession no. | | | | | | | | | | | | | | | | | | | | | | | | | | | | | | | | Score | | | | | | | | | | | | | | | | | | | | | | | | | | | | | M.P. | | | | | | | | | | | | | | | | | | | | | | | | | | | | | | | | | | | | | Cov. (%) | | | | | | | | | | | | | | | | | | | | | | | | | | | | | | | | | | | | | | | Blast score | | | | | | | | | | | | | | | | | | | | | | | | | | | | | | | | | | | | | | | | | | Mr (kDa) / pI | | | | | | | | | | | | | | | | | | | | | | | | | | | | | | | | | | | | | | | | | | | | | | | | | | | | | | | | | | | | | | | | | | | | | | | | | | | | | | | | | | | | | | | | | | | | | | | | | | | | | | Ratio | | | | | | | | | | | | | | | | | | | | | | | | | | | | | | | | | | | | | | | | | | | | | | | | | | | | | | | | | | | | | | | | | | |  |  |  |  |  |  |  |  |  |  |  |  |  |  |  |  |  |  |  |  |  |  |  |  |  |  |  |  |
|  |  |  |  |  |  |  |  |  |  |  |  |  |  |  |  |  |  |  |  |  |  |  |  |  |  |  |  |  |  |  |  |  |  |  |  |  |  |  |  |  |  |  |  |  |  |  |  |  |  |  |  |  |  |  |  |  |  |  |  |  |  |  |  |  |  |  |  |  |  |  |  |  |  |  |  |  |  |  |  |  |  |  |  |  |  |  |  |  |  |  |  |  |  |  |  |  |  |  |  |  |  |  |  |  |  |  |  |  |  |  |  |  |  |  |  |  |  |  |  |  |  |  |  |  |  |  |  |  |  |  |  |  |  |  |  |  |  |  |  |  |  |  |  |  |  |  |  |  |  |  |  |  |  |  |  |  |  |  |  |  |  |  |  |  |  |  |  |  |  |  |  |  |  |  |  |  |  |  |  |  |  |  |  |  |  |  |  |  |  |  |  |  |  |  |  |  |  |  |  |  |  |  |  |  |  |  |  |  |  |  | Theo. | | | | | | | | | | | | | | | | | | | | | | | | | | | | | | | | | | | | | | | | | | | | | | | | | | | | | | | | | | | | | | | Exp. | | | | | | | | | | | | | | | | | | | | | | | | | | | | | | | | | | | | | | |  |  |  |  |  |  |  |  |  |  |  |  |  |  |  |  |  |  |  |  |  |  |  |  |  |  |  |  |  |  |  |  |  |  |  |  |  |  |  |  |  |  |  |  |  |  |  |  |  |  |  |  |  |  |  |  |  |  |  |  |  |  |  |  |  |  |  |  |  |  |  |  |  |  |  |  |  |  |  |  |  |  |  |  |  |  |  |  |  |  |  |  |  |  |  |
| 3a | | | | | | | unknown | | | | | | | | | | | | | | | | | | | | | | | | | Glyma09g29030.1 | | | | | | | | | | | | | | | | | | | | | | | | | | | | | | | | 765 | | | | | | | | | | | | | | | | | | | | | | | | | | | | | 10 | | | | | | | | | | | | | | | | | | | | | | | | | | | | | | | | | | | | | 33 | | | | | | | | | | | | | | | | | | | | | | | | | | | | | | | | | | | | | | | 468 | | | | | | | | | | | | | | | | | | | | | | | | | | | | | | | | | | | | | | | | | | 31.4/5.07 | | | | | | | | | | | | | | | | | | | | | | | | | | | | | | | | | | | | | | | | | | | | | | | | | | | | | | | | | | | | | | |  | | | | | | | | | | | | | | | | | | | | | | | | | | | | | | | | | | | | | | |  | | | | | | | | | | | | | | | | | | | | | | | | | | | | | | | | | | | | | | | | | | | | | | | | | | | | | | | | | | | | | | | | | | |  |  |  |  |  |  |  |  |  |  |  |  |  |  |  |  |  |  |  |  |  |  |  |  |  |  |  |  |
|  | | | | | | | Calc. Mass | | | | | | | | | | | | | | | | | | | | | | | | | Observ. Mass | | | | | | | | | | | | | | | | | | | | | | | | | | | | | | | | Da | | | | | | | | | | | | | | | | | | | | | | | | | | | | | ppm | | | | | | | | | | | | | | | | | | | | | | | | | | | | | | | | | | | | | start | | | | | | | | | | | | | | | | | | | | | | | | | | | | | | | | | | | | | | | end | | | | | | | | | | | | | | | | | | | | | | | | | | | | | | | | | | | | | | | | | | sequence | | | | | | | | | | | | | | | | | | | | | | | | | | | | | | | | | | | | | | | | | | | | | | | | | | | | | | | | | | | | | | | | | | | | | | | | | | | | | | | | | | | | | | | | | | | | | | | | | | | | | | | | | | | | | | | | | | | | | | | | | | | | | | | | | | | | | | | | | | | | | | | | | | | | | | | | | | | | | | | | | | | | | | | | |  |  |  |  |  |  |  |  |  |  |  |  |  |  |  |  |  |  |  |  |  |  |  |  |  |  |  |  |
|  | | | | | | | 1709.7645 | | | | | | | | | | | | | | | | | | | | | | | | | 1709.761 | | | | | | | | | | | | | | | | | | | | | | | | | | | | | | | | 0 | | | | | | | | | | | | | | | | | | | | | | | | | | | | | -2 | | | | | | | | | | | | | | | | | | | | | | | | | | | | | | | | | | | | | 4 | | | | | | | | | | | | | | | | | | | | | | | | | | | | | | | | | | | | | | | 18 | | | | | | | | | | | | | | | | | | | | | | | | | | | | | | | | | | | | | | | | | | NQYDTDVTTWSPAGR | | | | | | | | | | | | | | | | | | | | | | | | | | | | | | | | | | | | | | | | | | | | | | | | | | | | | | | | | | | | | | | | | | | | | | | | | | | | | | | | | | | | | | | | | | | | | | | | | | | | | | | | | | | | | | | | | | | | | | | | | | | | | | | | | | | | | | | | | | | | | | | | | | | | | | | | | | | | | | | | | | | | | | | | |  |  |  |  |  |  |  |  |  |  |  |  |  |  |  |  |  |  |  |  |  |  |  |  |  |  |  |  |
|  | | | | | | | 1709.7645 | | | | | | | | | | | | | | | | | | | | | | | | | 1709.762 | | | | | | | | | | | | | | | | | | | | | | | | | | | | | | | | 0 | | | | | | | | | | | | | | | | | | | | | | | | | | | | | -1 | | | | | | | | | | | | | | | | | | | | | | | | | | | | | | | | | | | | | 4 | | | | | | | | | | | | | | | | | | | | | | | | | | | | | | | | | | | | | | | 18 | | | | | | | | | | | | | | | | | | | | | | | | | | | | | | | | | | | | | | | | | | NQYDTDVTTWSPAGR | | | | | | | | | | | | | | | | | | | | | | | | | | | | | | | | | | | | | | | | | | | | | | | | | | | | | | | | | | | | | | | | | | | | | | | | | | | | | | | | | | | | | | | | | | | | | | | | | | | | | | | | | | | | | | | | | | | | | | | | | | | | | | | | | | | | | | | | | | | | | | | | | | | | | | | | | | | | | | | | | | | | | | | | |  |  |  |  |  |  |  |  |  |  |  |  |  |  |  |  |  |  |  |  |  |  |  |  |  |  |  |  |
|  | | | | | | | 1709.7645 | | | | | | | | | | | | | | | | | | | | | | | | | 1709.763 | | | | | | | | | | | | | | | | | | | | | | | | | | | | | | | | 0 | | | | | | | | | | | | | | | | | | | | | | | | | | | | | -1 | | | | | | | | | | | | | | | | | | | | | | | | | | | | | | | | | | | | | 4 | | | | | | | | | | | | | | | | | | | | | | | | | | | | | | | | | | | | | | | 18 | | | | | | | | | | | | | | | | | | | | | | | | | | | | | | | | | | | | | | | | | | NQYDTDVTTWSPAGR | | | | | | | | | | | | | | | | | | | | | | | | | | | | | | | | | | | | | | | | | | | | | | | | | | | | | | | | | | | | | | | | | | | | | | | | | | | | | | | | | | | | | | | | | | | | | | | | | | | | | | | | | | | | | | | | | | | | | | | | | | | | | | | | | | | | | | | | | | | | | | | | | | | | | | | | | | | | | | | | | | | | | | | | |  |  |  |  |  |  |  |  |  |  |  |  |  |  |  |  |  |  |  |  |  |  |  |  |  |  |  |  |
|  | | | | | | | 1709.7645 | | | | | | | | | | | | | | | | | | | | | | | | | 1709.763 | | | | | | | | | | | | | | | | | | | | | | | | | | | | | | | | 0 | | | | | | | | | | | | | | | | | | | | | | | | | | | | | -1 | | | | | | | | | | | | | | | | | | | | | | | | | | | | | | | | | | | | | 4 | | | | | | | | | | | | | | | | | | | | | | | | | | | | | | | | | | | | | | | 18 | | | | | | | | | | | | | | | | | | | | | | | | | | | | | | | | | | | | | | | | | | NQYDTDVTTWSPAGR | | | | | | | | | | | | | | | | | | | | | | | | | | | | | | | | | | | | | | | | | | | | | | | | | | | | | | | | | | | | | | | | | | | | | | | | | | | | | | | | | | | | | | | | | | | | | | | | | | | | | | | | | | | | | | | | | | | | | | | | | | | | | | | | | | | | | | | | | | | | | | | | | | | | | | | | | | | | | | | | | | | | | | | | |  |  |  |  |  |  |  |  |  |  |  |  |  |  |  |  |  |  |  |  |  |  |  |  |  |  |  |  |
|  | | | | | | | 1709.7645 | | | | | | | | | | | | | | | | | | | | | | | | | 1709.763 | | | | | | | | | | | | | | | | | | | | | | | | | | | | | | | | 0 | | | | | | | | | | | | | | | | | | | | | | | | | | | | | -1 | | | | | | | | | | | | | | | | | | | | | | | | | | | | | | | | | | | | | 4 | | | | | | | | | | | | | | | | | | | | | | | | | | | | | | | | | | | | | | | 18 | | | | | | | | | | | | | | | | | | | | | | | | | | | | | | | | | | | | | | | | | | NQYDTDVTTWSPAGR | | | | | | | | | | | | | | | | | | | | | | | | | | | | | | | | | | | | | | | | | | | | | | | | | | | | | | | | | | | | | | | | | | | | | | | | | | | | | | | | | | | | | | | | | | | | | | | | | | | | | | | | | | | | | | | | | | | | | | | | | | | | | | | | | | | | | | | | | | | | | | | | | | | | | | | | | | | | | | | | | | | | | | | | |  |  |  |  |  |  |  |  |  |  |  |  |  |  |  |  |  |  |  |  |  |  |  |  |  |  |  |  |
|  | | | | | | | 1709.7645 | | | | | | | | | | | | | | | | | | | | | | | | | 1709.763 | | | | | | | | | | | | | | | | | | | | | | | | | | | | | | | | 0 | | | | | | | | | | | | | | | | | | | | | | | | | | | | | -1 | | | | | | | | | | | | | | | | | | | | | | | | | | | | | | | | | | | | | 4 | | | | | | | | | | | | | | | | | | | | | | | | | | | | | | | | | | | | | | | 18 | | | | | | | | | | | | | | | | | | | | | | | | | | | | | | | | | | | | | | | | | | NQYDTDVTTWSPAGR | | | | | | | | | | | | | | | | | | | | | | | | | | | | | | | | | | | | | | | | | | | | | | | | | | | | | | | | | | | | | | | | | | | | | | | | | | | | | | | | | | | | | | | | | | | | | | | | | | | | | | | | | | | | | | | | | | | | | | | | | | | | | | | | | | | | | | | | | | | | | | | | | | | | | | | | | | | | | | | | | | | | | | | | |  |  |  |  |  |  |  |  |  |  |  |  |  |  |  |  |  |  |  |  |  |  |  |  |  |  |  |  |
|  | | | | | | | 1709.7645 | | | | | | | | | | | | | | | | | | | | | | | | | 1709.763 | | | | | | | | | | | | | | | | | | | | | | | | | | | | | | | | 0 | | | | | | | | | | | | | | | | | | | | | | | | | | | | | -1 | | | | | | | | | | | | | | | | | | | | | | | | | | | | | | | | | | | | | 4 | | | | | | | | | | | | | | | | | | | | | | | | | | | | | | | | | | | | | | | 18 | | | | | | | | | | | | | | | | | | | | | | | | | | | | | | | | | | | | | | | | | | NQYDTDVTTWSPAGR | | | | | | | | | | | | | | | | | | | | | | | | | | | | | | | | | | | | | | | | | | | | | | | | | | | | | | | | | | | | | | | | | | | | | | | | | | | | | | | | | | | | | | | | | | | | | | | | | | | | | | | | | | | | | | | | | | | | | | | | | | | | | | | | | | | | | | | | | | | | | | | | | | | | | | | | | | | | | | | | | | | | | | | | |  |  |  |  |  |  |  |  |  |  |  |  |  |  |  |  |  |  |  |  |  |  |  |  |  |  |  |  |
|  | | | | | | | 1426.7166 | | | | | | | | | | | | | | | | | | | | | | | | | 1426.716 | | | | | | | | | | | | | | | | | | | | | | | | | | | | | | | | 0 | | | | | | | | | | | | | | | | | | | | | | | | | | | | | 0 | | | | | | | | | | | | | | | | | | | | | | | | | | | | | | | | | | | | | 19 | | | | | | | | | | | | | | | | | | | | | | | | | | | | | | | | | | | | | | | 30 | | | | | | | | | | | | | | | | | | | | | | | | | | | | | | | | | | | | | | | | | | LFQVEYAMEAVK | | | | | | | | | | | | | | | | | | | | | | | | | | | | | | | | | | | | | | | | | | | | | | | | | | | | | | | | | | | | | | | | | | | | | | | | | | | | | | | | | | | | | | | | | | | | | | | | | | | | | | | | | | | | | | | | | | | | | | | | | | | | | | | | | | | | | | | | | | | | | | | | | | | | | | | | | | | | | | | | | | | | | | | | |  |  |  |  |  |  |  |  |  |  |  |  |  |  |  |  |  |  |  |  |  |  |  |  |  |  |  |  |
|  | | | | | | | 1442.7115 | | | | | | | | | | | | | | | | | | | | | | | | | 1442.711 | | | | | | | | | | | | | | | | | | | | | | | | | | | | | | | | 0 | | | | | | | | | | | | | | | | | | | | | | | | | | | | | -1 | | | | | | | | | | | | | | | | | | | | | | | | | | | | | | | | | | | | | 19 | | | | | | | | | | | | | | | | | | | | | | | | | | | | | | | | | | | | | | | 30 | | | | | | | | | | | | | | | | | | | | | | | | | | | | | | | | | | | | | | | | | | LFQVEYAMEAVK | | | | | | | | | | | | | | | | | | | | | | | | | | | | | | | | | | | | | | | | | | | | | | | | | | | | | | | | | | | | | | | | | | | | | | | | | | | | | | | | | | | | | | | | | | | | | | | | | | | | | | | | | | | | | | | | | | | | | | | | | | | | | | | | | | | | | | | | | | | | | | | | | | | | | | | | | | | | | | | | | | | | | | | | |  |  |  |  |  |  |  |  |  |  |  |  |  |  |  |  |  |  |  |  |  |  |  |  |  |  |  |  |
|  | | | | | | | 1442.7115 | | | | | | | | | | | | | | | | | | | | | | | | | 1442.711 | | | | | | | | | | | | | | | | | | | | | | | | | | | | | | | | 0 | | | | | | | | | | | | | | | | | | | | | | | | | | | | | -1 | | | | | | | | | | | | | | | | | | | | | | | | | | | | | | | | | | | | | 19 | | | | | | | | | | | | | | | | | | | | | | | | | | | | | | | | | | | | | | | 30 | | | | | | | | | | | | | | | | | | | | | | | | | | | | | | | | | | | | | | | | | | LFQVEYAMEAVK | | | | | | | | | | | | | | | | | | | | | | | | | | | | | | | | | | | | | | | | | | | | | | | | | | | | | | | | | | | | | | | | | | | | | | | | | | | | | | | | | | | | | | | | | | | | | | | | | | | | | | | | | | | | | | | | | | | | | | | | | | | | | | | | | | | | | | | | | | | | | | | | | | | | | | | | | | | | | | | | | | | | | | | | |  |  |  |  |  |  |  |  |  |  |  |  |  |  |  |  |  |  |  |  |  |  |  |  |  |  |  |  |
|  | | | | | | | 1442.7115 | | | | | | | | | | | | | | | | | | | | | | | | | 1442.711 | | | | | | | | | | | | | | | | | | | | | | | | | | | | | | | | 0 | | | | | | | | | | | | | | | | | | | | | | | | | | | | | 0 | | | | | | | | | | | | | | | | | | | | | | | | | | | | | | | | | | | | | 19 | | | | | | | | | | | | | | | | | | | | | | | | | | | | | | | | | | | | | | | 30 | | | | | | | | | | | | | | | | | | | | | | | | | | | | | | | | | | | | | | | | | | LFQVEYAMEAVK | | | | | | | | | | | | | | | | | | | | | | | | | | | | | | | | | | | | | | | | | | | | | | | | | | | | | | | | | | | | | | | | | | | | | | | | | | | | | | | | | | | | | | | | | | | | | | | | | | | | | | | | | | | | | | | | | | | | | | | | | | | | | | | | | | | | | | | | | | | | | | | | | | | | | | | | | | | | | | | | | | | | | | | | |  |  |  |  |  |  |  |  |  |  |  |  |  |  |  |  |  |  |  |  |  |  |  |  |  |  |  |  |
|  | | | | | | | 1442.7115 | | | | | | | | | | | | | | | | | | | | | | | | | 1442.711 | | | | | | | | | | | | | | | | | | | | | | | | | | | | | | | | 0 | | | | | | | | | | | | | | | | | | | | | | | | | | | | | 0 | | | | | | | | | | | | | | | | | | | | | | | | | | | | | | | | | | | | | 19 | | | | | | | | | | | | | | | | | | | | | | | | | | | | | | | | | | | | | | | 30 | | | | | | | | | | | | | | | | | | | | | | | | | | | | | | | | | | | | | | | | | | LFQVEYAMEAVK | | | | | | | | | | | | | | | | | | | | | | | | | | | | | | | | | | | | | | | | | | | | | | | | | | | | | | | | | | | | | | | | | | | | | | | | | | | | | | | | | | | | | | | | | | | | | | | | | | | | | | | | | | | | | | | | | | | | | | | | | | | | | | | | | | | | | | | | | | | | | | | | | | | | | | | | | | | | | | | | | | | | | | | | |  |  |  |  |  |  |  |  |  |  |  |  |  |  |  |  |  |  |  |  |  |  |  |  |  |  |  |  |
|  | | | | | | | 871.4875 | | | | | | | | | | | | | | | | | | | | | | | | | 871.4876 | | | | | | | | | | | | | | | | | | | | | | | | | | | | | | | | 0 | | | | | | | | | | | | | | | | | | | | | | | | | | | | | 0 | | | | | | | | | | | | | | | | | | | | | | | | | | | | | | | | | | | | | 31 | | | | | | | | | | | | | | | | | | | | | | | | | | | | | | | | | | | | | | | 39 | | | | | | | | | | | | | | | | | | | | | | | | | | | | | | | | | | | | | | | | | | QGSAAIGLR | | | | | | | | | | | | | | | | | | | | | | | | | | | | | | | | | | | | | | | | | | | | | | | | | | | | | | | | | | | | | | | | | | | | | | | | | | | | | | | | | | | | | | | | | | | | | | | | | | | | | | | | | | | | | | | | | | | | | | | | | | | | | | | | | | | | | | | | | | | | | | | | | | | | | | | | | | | | | | | | | | | | | | | | |  |  |  |  |  |  |  |  |  |  |  |  |  |  |  |  |  |  |  |  |  |  |  |  |  |  |  |  |
|  | | | | | | | 1354.7391 | | | | | | | | | | | | | | | | | | | | | | | | | 1354.738 | | | | | | | | | | | | | | | | | | | | | | | | | | | | | | | | 0 | | | | | | | | | | | | | | | | | | | | | | | | | | | | | -1 | | | | | | | | | | | | | | | | | | | | | | | | | | | | | | | | | | | | | 40 | | | | | | | | | | | | | | | | | | | | | | | | | | | | | | | | | | | | | | | 51 | | | | | | | | | | | | | | | | | | | | | | | | | | | | | | | | | | | | | | | | | | SKTHVVLACVNK | | | | | | | | | | | | | | | | | | | | | | | | | | | | | | | | | | | | | | | | | | | | | | | | | | | | | | | | | | | | | | | | | | | | | | | | | | | | | | | | | | | | | | | | | | | | | | | | | | | | | | | | | | | | | | | | | | | | | | | | | | | | | | | | | | | | | | | | | | | | | | | | | | | | | | | | | | | | | | | | | | | | | | | | |  |  |  |  |  |  |  |  |  |  |  |  |  |  |  |  |  |  |  |  |  |  |  |  |  |  |  |  |
|  | | | | | | | 1354.7391 | | | | | | | | | | | | | | | | | | | | | | | | | 1354.738 | | | | | | | | | | | | | | | | | | | | | | | | | | | | | | | | 0 | | | | | | | | | | | | | | | | | | | | | | | | | | | | | -1 | | | | | | | | | | | | | | | | | | | | | | | | | | | | | | | | | | | | | 40 | | | | | | | | | | | | | | | | | | | | | | | | | | | | | | | | | | | | | | | 51 | | | | | | | | | | | | | | | | | | | | | | | | | | | | | | | | | | | | | | | | | | SKTHVVLACVNK | | | | | | | | | | | | | | | | | | | | | | | | | | | | | | | | | | | | | | | | | | | | | | | | | | | | | | | | | | | | | | | | | | | | | | | | | | | | | | | | | | | | | | | | | | | | | | | | | | | | | | | | | | | | | | | | | | | | | | | | | | | | | | | | | | | | | | | | | | | | | | | | | | | | | | | | | | | | | | | | | | | | | | | | |  |  |  |  |  |  |  |  |  |  |  |  |  |  |  |  |  |  |  |  |  |  |  |  |  |  |  |  |
|  | | | | | | | 1354.7391 | | | | | | | | | | | | | | | | | | | | | | | | | 1354.738 | | | | | | | | | | | | | | | | | | | | | | | | | | | | | | | | 0 | | | | | | | | | | | | | | | | | | | | | | | | | | | | | -1 | | | | | | | | | | | | | | | | | | | | | | | | | | | | | | | | | | | | | 40 | | | | | | | | | | | | | | | | | | | | | | | | | | | | | | | | | | | | | | | 51 | | | | | | | | | | | | | | | | | | | | | | | | | | | | | | | | | | | | | | | | | | SKTHVVLACVNK | | | | | | | | | | | | | | | | | | | | | | | | | | | | | | | | | | | | | | | | | | | | | | | | | | | | | | | | | | | | | | | | | | | | | | | | | | | | | | | | | | | | | | | | | | | | | | | | | | | | | | | | | | | | | | | | | | | | | | | | | | | | | | | | | | | | | | | | | | | | | | | | | | | | | | | | | | | | | | | | | | | | | | | | |  |  |  |  |  |  |  |  |  |  |  |  |  |  |  |  |  |  |  |  |  |  |  |  |  |  |  |  |
|  | | | | | | | 1354.7391 | | | | | | | | | | | | | | | | | | | | | | | | | 1354.738 | | | | | | | | | | | | | | | | | | | | | | | | | | | | | | | | 0 | | | | | | | | | | | | | | | | | | | | | | | | | | | | | -1 | | | | | | | | | | | | | | | | | | | | | | | | | | | | | | | | | | | | | 40 | | | | | | | | | | | | | | | | | | | | | | | | | | | | | | | | | | | | | | | 51 | | | | | | | | | | | | | | | | | | | | | | | | | | | | | | | | | | | | | | | | | | SKTHVVLACVNK | | | | | | | | | | | | | | | | | | | | | | | | | | | | | | | | | | | | | | | | | | | | | | | | | | | | | | | | | | | | | | | | | | | | | | | | | | | | | | | | | | | | | | | | | | | | | | | | | | | | | | | | | | | | | | | | | | | | | | | | | | | | | | | | | | | | | | | | | | | | | | | | | | | | | | | | | | | | | | | | | | | | | | | | |  |  |  |  |  |  |  |  |  |  |  |  |  |  |  |  |  |  |  |  |  |  |  |  |  |  |  |  |
|  | | | | | | | 1354.7391 | | | | | | | | | | | | | | | | | | | | | | | | | 1354.739 | | | | | | | | | | | | | | | | | | | | | | | | | | | | | | | | 0 | | | | | | | | | | | | | | | | | | | | | | | | | | | | | 0 | | | | | | | | | | | | | | | | | | | | | | | | | | | | | | | | | | | | | 40 | | | | | | | | | | | | | | | | | | | | | | | | | | | | | | | | | | | | | | | 51 | | | | | | | | | | | | | | | | | | | | | | | | | | | | | | | | | | | | | | | | | | SKTHVVLACVNK | | | | | | | | | | | | | | | | | | | | | | | | | | | | | | | | | | | | | | | | | | | | | | | | | | | | | | | | | | | | | | | | | | | | | | | | | | | | | | | | | | | | | | | | | | | | | | | | | | | | | | | | | | | | | | | | | | | | | | | | | | | | | | | | | | | | | | | | | | | | | | | | | | | | | | | | | | | | | | | | | | | | | | | | |  |  |  |  |  |  |  |  |  |  |  |  |  |  |  |  |  |  |  |  |  |  |  |  |  |  |  |  |
|  | | | | | | | 1354.7391 | | | | | | | | | | | | | | | | | | | | | | | | | 1354.739 | | | | | | | | | | | | | | | | | | | | | | | | | | | | | | | | 0 | | | | | | | | | | | | | | | | | | | | | | | | | | | | | 0 | | | | | | | | | | | | | | | | | | | | | | | | | | | | | | | | | | | | | 40 | | | | | | | | | | | | | | | | | | | | | | | | | | | | | | | | | | | | | | | 51 | | | | | | | | | | | | | | | | | | | | | | | | | | | | | | | | | | | | | | | | | | SKTHVVLACVNK | | | | | | | | | | | | | | | | | | | | | | | | | | | | | | | | | | | | | | | | | | | | | | | | | | | | | | | | | | | | | | | | | | | | | | | | | | | | | | | | | | | | | | | | | | | | | | | | | | | | | | | | | | | | | | | | | | | | | | | | | | | | | | | | | | | | | | | | | | | | | | | | | | | | | | | | | | | | | | | | | | | | | | | | |  |  |  |  |  |  |  |  |  |  |  |  |  |  |  |  |  |  |  |  |  |  |  |  |  |  |  |  |
|  | | | | | | | 1354.7391 | | | | | | | | | | | | | | | | | | | | | | | | | 1354.739 | | | | | | | | | | | | | | | | | | | | | | | | | | | | | | | | 0 | | | | | | | | | | | | | | | | | | | | | | | | | | | | | 0 | | | | | | | | | | | | | | | | | | | | | | | | | | | | | | | | | | | | | 40 | | | | | | | | | | | | | | | | | | | | | | | | | | | | | | | | | | | | | | | 51 | | | | | | | | | | | | | | | | | | | | | | | | | | | | | | | | | | | | | | | | | | SKTHVVLACVNK | | | | | | | | | | | | | | | | | | | | | | | | | | | | | | | | | | | | | | | | | | | | | | | | | | | | | | | | | | | | | | | | | | | | | | | | | | | | | | | | | | | | | | | | | | | | | | | | | | | | | | | | | | | | | | | | | | | | | | | | | | | | | | | | | | | | | | | | | | | | | | | | | | | | | | | | | | | | | | | | | | | | | | | | |  |  |  |  |  |  |  |  |  |  |  |  |  |  |  |  |  |  |  |  |  |  |  |  |  |  |  |  |
|  | | | | | | | 1354.7391 | | | | | | | | | | | | | | | | | | | | | | | | | 1354.74 | | | | | | | | | | | | | | | | | | | | | | | | | | | | | | | | 0 | | | | | | | | | | | | | | | | | | | | | | | | | | | | | 0 | | | | | | | | | | | | | | | | | | | | | | | | | | | | | | | | | | | | | 40 | | | | | | | | | | | | | | | | | | | | | | | | | | | | | | | | | | | | | | | 51 | | | | | | | | | | | | | | | | | | | | | | | | | | | | | | | | | | | | | | | | | | SKTHVVLACVNK | | | | | | | | | | | | | | | | | | | | | | | | | | | | | | | | | | | | | | | | | | | | | | | | | | | | | | | | | | | | | | | | | | | | | | | | | | | | | | | | | | | | | | | | | | | | | | | | | | | | | | | | | | | | | | | | | | | | | | | | | | | | | | | | | | | | | | | | | | | | | | | | | | | | | | | | | | | | | | | | | | | | | | | | |  |  |  |  |  |  |  |  |  |  |  |  |  |  |  |  |  |  |  |  |  |  |  |  |  |  |  |  |
|  | | | | | | | 1354.7391 | | | | | | | | | | | | | | | | | | | | | | | | | 1354.741 | | | | | | | | | | | | | | | | | | | | | | | | | | | | | | | | 0 | | | | | | | | | | | | | | | | | | | | | | | | | | | | | 1 | | | | | | | | | | | | | | | | | | | | | | | | | | | | | | | | | | | | | 40 | | | | | | | | | | | | | | | | | | | | | | | | | | | | | | | | | | | | | | | 51 | | | | | | | | | | | | | | | | | | | | | | | | | | | | | | | | | | | | | | | | | | SKTHVVLACVNK | | | | | | | | | | | | | | | | | | | | | | | | | | | | | | | | | | | | | | | | | | | | | | | | | | | | | | | | | | | | | | | | | | | | | | | | | | | | | | | | | | | | | | | | | | | | | | | | | | | | | | | | | | | | | | | | | | | | | | | | | | | | | | | | | | | | | | | | | | | | | | | | | | | | | | | | | | | | | | | | | | | | | | | | |  |  |  |  |  |  |  |  |  |  |  |  |  |  |  |  |  |  |  |  |  |  |  |  |  |  |  |  |
|  | | | | | | | 1139.6121 | | | | | | | | | | | | | | | | | | | | | | | | | 1139.611 | | | | | | | | | | | | | | | | | | | | | | | | | | | | | | | | 0 | | | | | | | | | | | | | | | | | | | | | | | | | | | | | -1 | | | | | | | | | | | | | | | | | | | | | | | | | | | | | | | | | | | | | 42 | | | | | | | | | | | | | | | | | | | | | | | | | | | | | | | | | | | | | | | 51 | | | | | | | | | | | | | | | | | | | | | | | | | | | | | | | | | | | | | | | | | | THVVLACVNK | | | | | | | | | | | | | | | | | | | | | | | | | | | | | | | | | | | | | | | | | | | | | | | | | | | | | | | | | | | | | | | | | | | | | | | | | | | | | | | | | | | | | | | | | | | | | | | | | | | | | | | | | | | | | | | | | | | | | | | | | | | | | | | | | | | | | | | | | | | | | | | | | | | | | | | | | | | | | | | | | | | | | | | | |  |  |  |  |  |  |  |  |  |  |  |  |  |  |  |  |  |  |  |  |  |  |  |  |  |  |  |  |
|  | | | | | | | 2160.9786 | | | | | | | | | | | | | | | | | | | | | | | | | 2160.977 | | | | | | | | | | | | | | | | | | | | | | | | | | | | | | | | 0 | | | | | | | | | | | | | | | | | | | | | | | | | | | | | -1 | | | | | | | | | | | | | | | | | | | | | | | | | | | | | | | | | | | | | 90 | | | | | | | | | | | | | | | | | | | | | | | | | | | | | | | | | | | | | | | 107 | | | | | | | | | | | | | | | | | | | | | | | | | | | | | | | | | | | | | | | | | | SECINYNYTYESPLPVGR | | | | | | | | | | | | | | | | | | | | | | | | | | | | | | | | | | | | | | | | | | | | | | | | | | | | | | | | | | | | | | | | | | | | | | | | | | | | | | | | | | | | | | | | | | | | | | | | | | | | | | | | | | | | | | | | | | | | | | | | | | | | | | | | | | | | | | | | | | | | | | | | | | | | | | | | | | | | | | | | | | | | | | | | |  |  |  |  |  |  |  |  |  |  |  |  |  |  |  |  |  |  |  |  |  |  |  |  |  |  |  |  |
|  | | | | | | | 2160.9786 | | | | | | | | | | | | | | | | | | | | | | | | | 2160.977 | | | | | | | | | | | | | | | | | | | | | | | | | | | | | | | | 0 | | | | | | | | | | | | | | | | | | | | | | | | | | | | | -1 | | | | | | | | | | | | | | | | | | | | | | | | | | | | | | | | | | | | | 90 | | | | | | | | | | | | | | | | | | | | | | | | | | | | | | | | | | | | | | | 107 | | | | | | | | | | | | | | | | | | | | | | | | | | | | | | | | | | | | | | | | | | SECINYNYTYESPLPVGR | | | | | | | | | | | | | | | | | | | | | | | | | | | | | | | | | | | | | | | | | | | | | | | | | | | | | | | | | | | | | | | | | | | | | | | | | | | | | | | | | | | | | | | | | | | | | | | | | | | | | | | | | | | | | | | | | | | | | | | | | | | | | | | | | | | | | | | | | | | | | | | | | | | | | | | | | | | | | | | | | | | | | | | | |  |  |  |  |  |  |  |  |  |  |  |  |  |  |  |  |  |  |  |  |  |  |  |  |  |  |  |  |
|  | | | | | | | 884.5331 | | | | | | | | | | | | | | | | | | | | | | | | | 884.5326 | | | | | | | | | | | | | | | | | | | | | | | | | | | | | | | | 0 | | | | | | | | | | | | | | | | | | | | | | | | | | | | | -1 | | | | | | | | | | | | | | | | | | | | | | | | | | | | | | | | | | | | | 108 | | | | | | | | | | | | | | | | | | | | | | | | | | | | | | | | | | | | | | | 115 | | | | | | | | | | | | | | | | | | | | | | | | | | | | | | | | | | | | | | | | | | LVVQLADK | | | | | | | | | | | | | | | | | | | | | | | | | | | | | | | | | | | | | | | | | | | | | | | | | | | | | | | | | | | | | | | | | | | | | | | | | | | | | | | | | | | | | | | | | | | | | | | | | | | | | | | | | | | | | | | | | | | | | | | | | | | | | | | | | | | | | | | | | | | | | | | | | | | | | | | | | | | | | | | | | | | | | | | | |  |  |  |  |  |  |  |  |  |  |  |  |  |  |  |  |  |  |  |  |  |  |  |  |  |  |  |  |
|  | | | | | | | 884.5331 | | | | | | | | | | | | | | | | | | | | | | | | | 884.5336 | | | | | | | | | | | | | | | | | | | | | | | | | | | | | | | | 0 | | | | | | | | | | | | | | | | | | | | | | | | | | | | | 1 | | | | | | | | | | | | | | | | | | | | | | | | | | | | | | | | | | | | | 108 | | | | | | | | | | | | | | | | | | | | | | | | | | | | | | | | | | | | | | | 115 | | | | | | | | | | | | | | | | | | | | | | | | | | | | | | | | | | | | | | | | | | LVVQLADK | | | | | | | | | | | | | | | | | | | | | | | | | | | | | | | | | | | | | | | | | | | | | | | | | | | | | | | | | | | | | | | | | | | | | | | | | | | | | | | | | | | | | | | | | | | | | | | | | | | | | | | | | | | | | | | | | | | | | | | | | | | | | | | | | | | | | | | | | | | | | | | | | | | | | | | | | | | | | | | | | | | | | | | | |  |  |  |  |  |  |  |  |  |  |  |  |  |  |  |  |  |  |  |  |  |  |  |  |  |  |  |  |
|  | | | | | | | 1197.5891 | | | | | | | | | | | | | | | | | | | | | | | | | 1197.588 | | | | | | | | | | | | | | | | | | | | | | | | | | | | | | | | 0 | | | | | | | | | | | | | | | | | | | | | | | | | | | | | -1 | | | | | | | | | | | | | | | | | | | | | | | | | | | | | | | | | | | | | 175 | | | | | | | | | | | | | | | | | | | | | | | | | | | | | | | | | | | | | | | 184 | | | | | | | | | | | | | | | | | | | | | | | | | | | | | | | | | | | | | | | | | | RFENFVGSSR | | | | | | | | | | | | | | | | | | | | | | | | | | | | | | | | | | | | | | | | | | | | | | | | | | | | | | | | | | | | | | | | | | | | | | | | | | | | | | | | | | | | | | | | | | | | | | | | | | | | | | | | | | | | | | | | | | | | | | | | | | | | | | | | | | | | | | | | | | | | | | | | | | | | | | | | | | | | | | | | | | | | | | | | |  |  |  |  |  |  |  |  |  |  |  |  |  |  |  |  |  |  |  |  |  |  |  |  |  |  |  |  |
|  | | | | | | | 1197.5891 | | | | | | | | | | | | | | | | | | | | | | | | | 1197.589 | | | | | | | | | | | | | | | | | | | | | | | | | | | | | | | | 0 | | | | | | | | | | | | | | | | | | | | | | | | | | | | | 0 | | | | | | | | | | | | | | | | | | | | | | | | | | | | | | | | | | | | | 175 | | | | | | | | | | | | | | | | | | | | | | | | | | | | | | | | | | | | | | | 184 | | | | | | | | | | | | | | | | | | | | | | | | | | | | | | | | | | | | | | | | | | RFENFVGSSR | | | | | | | | | | | | | | | | | | | | | | | | | | | | | | | | | | | | | | | | | | | | | | | | | | | | | | | | | | | | | | | | | | | | | | | | | | | | | | | | | | | | | | | | | | | | | | | | | | | | | | | | | | | | | | | | | | | | | | | | | | | | | | | | | | | | | | | | | | | | | | | | | | | | | | | | | | | | | | | | | | | | | | | | |  |  |  |  |  |  |  |  |  |  |  |  |  |  |  |  |  |  |  |  |  |  |  |  |  |  |  |  |
|  | | | | | | | 1639.8206 | | | | | | | | | | | | | | | | | | | | | | | | | 1639.819 | | | | | | | | | | | | | | | | | | | | | | | | | | | | | | | | 0 | | | | | | | | | | | | | | | | | | | | | | | | | | | | | -1 | | | | | | | | | | | | | | | | | | | | | | | | | | | | | | | | | | | | | 176 | | | | | | | | | | | | | | | | | | | | | | | | | | | | | | | | | | | | | | | 189 | | | | | | | | | | | | | | | | | | | | | | | | | | | | | | | | | | | | | | | | | | FENFVGSSREDLIK | | | | | | | | | | | | | | | | | | | | | | | | | | | | | | | | | | | | | | | | | | | | | | | | | | | | | | | | | | | | | | | | | | | | | | | | | | | | | | | | | | | | | | | | | | | | | | | | | | | | | | | | | | | | | | | | | | | | | | | | | | | | | | | | | | | | | | | | | | | | | | | | | | | | | | | | | | | | | | | | | | | | | | | | |  |  |  |  |  |  |  |  |  |  |  |  |  |  |  |  |  |  |  |  |  |  |  |  |  |  |  |  |
|  | | | | | | | 1356.7612 | | | | | | | | | | | | | | | | | | | | | | | | | 1356.759 | | | | | | | | | | | | | | | | | | | | | | | | | | | | | | | | 0 | | | | | | | | | | | | | | | | | | | | | | | | | | | | | -1 | | | | | | | | | | | | | | | | | | | | | | | | | | | | | | | | | | | | | 185 | | | | | | | | | | | | | | | | | | | | | | | | | | | | | | | | | | | | | | | 196 | | | | | | | | | | | | | | | | | | | | | | | | | | | | | | | | | | | | | | | | | | EDLIKDALIATR | | | | | | | | | | | | | | | | | | | | | | | | | | | | | | | | | | | | | | | | | | | | | | | | | | | | | | | | | | | | | | | | | | | | | | | | | | | | | | | | | | | | | | | | | | | | | | | | | | | | | | | | | | | | | | | | | | | | | | | | | | | | | | | | | | | | | | | | | | | | | | | | | | | | | | | | | | | | | | | | | | | | | | | | |  |  |  |  |  |  |  |  |  |  |  |  |  |  |  |  |  |  |  |  |  |  |  |  |  |  |  |  |
|  | | | | | | | 1356.7612 | | | | | | | | | | | | | | | | | | | | | | | | | 1356.761 | | | | | | | | | | | | | | | | | | | | | | | | | | | | | | | | 0 | | | | | | | | | | | | | | | | | | | | | | | | | | | | | 0 | | | | | | | | | | | | | | | | | | | | | | | | | | | | | | | | | | | | | 185 | | | | | | | | | | | | | | | | | | | | | | | | | | | | | | | | | | | | | | | 196 | | | | | | | | | | | | | | | | | | | | | | | | | | | | | | | | | | | | | | | | | | EDLIKDALIATR | | | | | | | | | | | | | | | | | | | | | | | | | | | | | | | | | | | | | | | | | | | | | | | | | | | | | | | | | | | | | | | | | | | | | | | | | | | | | | | | | | | | | | | | | | | | | | | | | | | | | | | | | | | | | | | | | | | | | | | | | | | | | | | | | | | | | | | | | | | | | | | | | | | | | | | | | | | | | | | | | | | | | | | | |  |  |  |  |  |  |  |  |  |  |  |  |  |  |  |  |  |  |  |  |  |  |  |  |  |  |  |  |
|  | | | | | | | 1356.7612 | | | | | | | | | | | | | | | | | | | | | | | | | 1356.762 | | | | | | | | | | | | | | | | | | | | | | | | | | | | | | | | 0 | | | | | | | | | | | | | | | | | | | | | | | | | | | | | 0 | | | | | | | | | | | | | | | | | | | | | | | | | | | | | | | | | | | | | 185 | | | | | | | | | | | | | | | | | | | | | | | | | | | | | | | | | | | | | | | 196 | | | | | | | | | | | | | | | | | | | | | | | | | | | | | | | | | | | | | | | | | | EDLIKDALIATR | | | | | | | | | | | | | | | | | | | | | | | | | | | | | | | | | | | | | | | | | | | | | | | | | | | | | | | | | | | | | | | | | | | | | | | | | | | | | | | | | | | | | | | | | | | | | | | | | | | | | | | | | | | | | | | | | | | | | | | | | | | | | | | | | | | | | | | | | | | | | | | | | | | | | | | | | | | | | | | | | | | | | | | | |  |  |  |  |  |  |  |  |  |  |  |  |  |  |  |  |  |  |  |  |  |  |  |  |  |  |  |  |
|  | | | | | | | 1356.7612 | | | | | | | | | | | | | | | | | | | | | | | | | 1356.762 | | | | | | | | | | | | | | | | | | | | | | | | | | | | | | | | 0 | | | | | | | | | | | | | | | | | | | | | | | | | | | | | 0 | | | | | | | | | | | | | | | | | | | | | | | | | | | | | | | | | | | | | 185 | | | | | | | | | | | | | | | | | | | | | | | | | | | | | | | | | | | | | | | 196 | | | | | | | | | | | | | | | | | | | | | | | | | | | | | | | | | | | | | | | | | | EDLIKDALIATR | | | | | | | | | | | | | | | | | | | | | | | | | | | | | | | | | | | | | | | | | | | | | | | | | | | | | | | | | | | | | | | | | | | | | | | | | | | | | | | | | | | | | | | | | | | | | | | | | | | | | | | | | | | | | | | | | | | | | | | | | | | | | | | | | | | | | | | | | | | | | | | | | | | | | | | | | | | | | | | | | | | | | | | | |  |  |  |  |  |  |  |  |  |  |  |  |  |  |  |  |  |  |  |  |  |  |  |  |  |  |  |  |
| Spot no. | | | | | | | Homologous protein | | | | | | | | | | | | | | | | | | Accession no. | | | | | | | | | | | | | | | | | | | | | | | | | | | | | Score | | | | | | | | | | | | | | | | | | | | | | | | | | | | | | | | | | | | | | | | | | | | | | | | | | | | | | | | | M.P. | | | | | | | | | | | | | | | | | | | | | | | | | | | | | | | | | | | | | Cov. (%) | | | | | | | | | | | | | | | | | | | | | | | | | | | | | | | | | | | | | | Blast score | | | | | | | | | | | | | | | | | | | | | | | | | | | | | | | | | | | | | | | | | | Mr (kDa) / pI | | | | | | | | | | | | | | | | | | | | | | | | | | | | | | | | | | | | | | | | | | | | | | | | | | | | | | | | | | | | | | | | | | | | | | | | | | | | | | | | | | | | | | | | | | | | | | Ratio | | | | | | | | | | | | | | | | | | | | | | | | | | | | | | | | | | | | | | | | | | | | | | | | | | | | | | | | | |  |  |  |  |  |  |  |  |  |  |  |  |  |  |  |  |  |  |  |  |  |  |  |  |  |  |  |  |
|  |  |  |  |  |  |  |  |  |  |  |  |  |  |  |  |  |  |  |  |  |  |  |  |  |  |  |  |  |  |  |  |  |  |  |  |  |  |  |  |  |  |  |  |  |  |  |  |  |  |  |  |  |  |  |  |  |  |  |  |  |  |  |  |  |  |  |  |  |  |  |  |  |  |  |  |  |  |  |  |  |  |  |  |  |  |  |  |  |  |  |  |  |  |  |  |  |  |  |  |  |  |  |  |  |  |  |  |  |  |  |  |  |  |  |  |  |  |  |  |  |  |  |  |  |  |  |  |  |  |  |  |  |  |  |  |  |  |  |  |  |  |  |  |  |  |  |  |  |  |  |  |  |  |  |  |  |  |  |  |  |  |  |  |  |  |  |  |  |  |  |  |  |  |  |  |  |  |  |  |  |  |  |  |  |  |  |  |  |  |  |  |  |  |  |  |  |  |  |  |  |  |  |  |  |  |  |  |  |  |  |  |  |  |  |  |  |  |  |  |  |  |  |  |  |  |  |  | Theo. | | | | | | | | | | | | | | | | | | | | | | | | | | | | | | | | | | | | | | | | | | | | | | | | | | | | | | | | | | | | | | Exp. | | | | | | | | | | | | | | | | | | | | | | | | | | | | | | | |  |  |  |  |  |  |  |  |  |  |  |  |  |  |  |  |  |  |  |  |  |  |  |  |  |  |  |  |  |  |  |  |  |  |  |  |  |  |  |  |  |  |  |  |  |  |  |  |  |  |  |  |  |  |  |  |  |  |  |  |  |  |  |  |  |  |  |  |  |  |  |  |  |  |  |  |  |  |  |  |  |  |  |  |  |  |
| 3b | | | | | | | lipoxygease L-4 | | | | | | | | | | | | | | | | | | Glyma13g42330.1 | | | | | | | | | | | | | | | | | | | | | | | | | | | | | 230 | | | | | | | | | | | | | | | | | | | | | | | | | | | | | | | | | | | | | | | | | | | | | | | | | | | | | | | | | 4 | | | | | | | | | | | | | | | | | | | | | | | | | | | | | | | | | | | | | 7 | | | | | | | | | | | | | | | | | | | | | | | | | | | | | | | | | | | | | | 1650 | | | | | | | | | | | | | | | | | | | | | | | | | | | | | | | | | | | | | | | | | | 96.8/5.76 | | | | | | | | | | | | | | | | | | | | | | | | | | | | | | | | | | | | | | | | | | | | | | | | | | | | | | | | | | | | | |  | | | | | | | | | | | | | | | | | | | | | | | | | | | | | | | |  | | | | | | | | | | | | | | | | | | | | | | | | | | | | | | | | | | | | | | | | | | | | | | | | | | | | | | | | | |  |  |  |  |  |  |  |  |  |  |  |  |  |  |  |  |  |  |  |  |  |  |  |  |  |  |  |  |
|  | | | | | | | Calc. Mass | | | | | | | | | | | | | | | | | | Observ. Mass | | | | | | | | | | | | | | | | | | | | | | | | | | | | | Da | | | | | | | | | | | | | | | | | | | | | | | | | | | | | | | | | | | | | | | | | | | | | | | | | | | | | | | | | ppm | | | | | | | | | | | | | | | | | | | | | | | | | | | | | | | | | | | | | start | | | | | | | | | | | | | | | | | | | | | | | | | | | | | | | | | | | | | | | | | end | | | | | | | | | | | | | | | | | | | | | | | | | | | | | | | | | | | | | | | | | | sequence | | | | | | | | | | | | | | | | | | | | | | | | | | | | | | | | | | | | | | | | | | | | | | | | | | | | | | | | | | | | | | | | | | | | | | | | | | | | | | | | | | | | | | | | | | | | | | | | | | | | | | | | | | | | | | | | | | | | | | | | | | | | | | | | | | | | | | | | | | | | | | | | | | | | |  |  |  |  |  |  |  |  |  |  |  |  |  |  |  |  |  |  |  |  |  |  |  |  |  |  |  |  |
|  | | | | | | | 2135.1303 | | | | | | | | | | | | | | | | | | 2135.127 | | | | | | | | | | | | | | | | | | | | | | | | | | | | | 0 | | | | | | | | | | | | | | | | | | | | | | | | | | | | | | | | | | | | | | | | | | | | | | | | | | | | | | | | | -1 | | | | | | | | | | | | | | | | | | | | | | | | | | | | | | | | | | | | | 156 | | | | | | | | | | | | | | | | | | | | | | | | | | | | | | | | | | | | | | | | | 174 | | | | | | | | | | | | | | | | | | | | | | | | | | | | | | | | | | | | | | | | | | IFFANNTYLPSETPAPLLK | | | | | | | | | | | | | | | | | | | | | | | | | | | | | | | | | | | | | | | | | | | | | | | | | | | | | | | | | | | | | | | | | | | | | | | | | | | | | | | | | | | | | | | | | | | | | | | | | | | | | | | | | | | | | | | | | | | | | | | | | | | | | | | | | | | | | | | | | | | | | | | | | | | | |  |  |  |  |  |  |  |  |  |  |  |  |  |  |  |  |  |  |  |  |  |  |  |  |  |  |  |  |
|  | | | | | | | 2061.8803 | | | | | | | | | | | | | | | | | | 2061.879 | | | | | | | | | | | | | | | | | | | | | | | | | | | | | 0 | | | | | | | | | | | | | | | | | | | | | | | | | | | | | | | | | | | | | | | | | | | | | | | | | | | | | | | | | 0 | | | | | | | | | | | | | | | | | | | | | | | | | | | | | | | | | | | | | 197 | | | | | | | | | | | | | | | | | | | | | | | | | | | | | | | | | | | | | | | | | 214 | | | | | | | | | | | | | | | | | | | | | | | | | | | | | | | | | | | | | | | | | | IYDYDVYNDLGNPDSGDK | | | | | | | | | | | | | | | | | | | | | | | | | | | | | | | | | | | | | | | | | | | | | | | | | | | | | | | | | | | | | | | | | | | | | | | | | | | | | | | | | | | | | | | | | | | | | | | | | | | | | | | | | | | | | | | | | | | | | | | | | | | | | | | | | | | | | | | | | | | | | | | | | | | | |  |  |  |  |  |  |  |  |  |  |  |  |  |  |  |  |  |  |  |  |  |  |  |  |  |  |  |  |
|  | | | | | | | 1099.555 | | | | | | | | | | | | | | | | | | 1099.555 | | | | | | | | | | | | | | | | | | | | | | | | | | | | | 0 | | | | | | | | | | | | | | | | | | | | | | | | | | | | | | | | | | | | | | | | | | | | | | | | | | | | | | | | | 0 | | | | | | | | | | | | | | | | | | | | | | | | | | | | | | | | | | | | | 264 | | | | | | | | | | | | | | | | | | | | | | | | | | | | | | | | | | | | | | | | | 273 | | | | | | | | | | | | | | | | | | | | | | | | | | | | | | | | | | | | | | | | | | SSDFLAYGIK | | | | | | | | | | | | | | | | | | | | | | | | | | | | | | | | | | | | | | | | | | | | | | | | | | | | | | | | | | | | | | | | | | | | | | | | | | | | | | | | | | | | | | | | | | | | | | | | | | | | | | | | | | | | | | | | | | | | | | | | | | | | | | | | | | | | | | | | | | | | | | | | | | | | |  |  |  |  |  |  |  |  |  |  |  |  |  |  |  |  |  |  |  |  |  |  |  |  |  |  |  |  |
|  | | | | | | | 1637.8665 | | | | | | | | | | | | | | | | | | 1637.863 | | | | | | | | | | | | | | | | | | | | | | | | | | | | | 0 | | | | | | | | | | | | | | | | | | | | | | | | | | | | | | | | | | | | | | | | | | | | | | | | | | | | | | | | | -2 | | | | | | | | | | | | | | | | | | | | | | | | | | | | | | | | | | | | | 305 | | | | | | | | | | | | | | | | | | | | | | | | | | | | | | | | | | | | | | | | | 319 | | | | | | | | | | | | | | | | | | | | | | | | | | | | | | | | | | | | | | | | | | LYEGGVTLPTNFLSK | | | | | | | | | | | | | | | | | | | | | | | | | | | | | | | | | | | | | | | | | | | | | | | | | | | | | | | | | | | | | | | | | | | | | | | | | | | | | | | | | | | | | | | | | | | | | | | | | | | | | | | | | | | | | | | | | | | | | | | | | | | | | | | | | | | | | | | | | | | | | | | | | | | | |  |  |  |  |  |  |  |  |  |  |  |  |  |  |  |  |  |  |  |  |  |  |  |  |  |  |  |  |
| Spot no. | | | | | | | | | | | Homologous protein | | | | | | | | | | | | | | | | | | | | | | | | | | | | | Accession no. | | | | | | | | | | | | | | | | | | | | | | | | | | | | | | | | | | Score | | | | | | | | | | | | | | | | | | | | | | | | | | | | | | | | | | M.P. | | | | | | | | | | | | | | | | | | | | | | | | | | | | | | | Cov. (%) | | | | | | | | | | | | | | | | | | | | | | | | | | | | | | | | | | | | | | | | | | Blast score | | | | | | | | | | | | | | | | | | | | | | | | | | | | | | | | | | | | | | | | Mr (kDa) / pI | | | | | | | | | | | | | | | | | | | | | | | | | | | | | | | | | | | | | | | | | | | | | | | | | | | | | | | | | | | | | | | | | | | | | | | | | | | | | | | | | | | | | | | | | Ratio | | | | | | | | | | | | | | | | | | | | | | | | | | | | | | | | | | | | | | | | | | | | | | | | | | | | | | | | | | | | | | | | | | | | | | | | | | | |  |  |  |  |  |  |  |  |  |  |  |  |  |  |  |  |  |  |  |  |  |  |
|  |  |  |  |  |  |  |  |  |  |  |  |  |  |  |  |  |  |  |  |  |  |  |  |  |  |  |  |  |  |  |  |  |  |  |  |  |  |  |  |  |  |  |  |  |  |  |  |  |  |  |  |  |  |  |  |  |  |  |  |  |  |  |  |  |  |  |  |  |  |  |  |  |  |  |  |  |  |  |  |  |  |  |  |  |  |  |  |  |  |  |  |  |  |  |  |  |  |  |  |  |  |  |  |  |  |  |  |  |  |  |  |  |  |  |  |  |  |  |  |  |  |  |  |  |  |  |  |  |  |  |  |  |  |  |  |  |  |  |  |  |  |  |  |  |  |  |  |  |  |  |  |  |  |  |  |  |  |  |  |  |  |  |  |  |  |  |  |  |  |  |  |  |  |  |  |  |  |  |  |  |  |  |  |  |  |  |  |  |  |  |  |  |  |  |  |  |  |  |  |  |  |  |  |  |  |  |  |  |  |  |  |  |  |  |  |  |  |  |  |  | Theo. | | | | | | | | | | | | | | | | | | | | | | | | | | | | | | | | | | | | | | | | | | | | | | | | | | | | | Exp. | | | | | | | | | | | | | | | | | | | | | | | | | | | | | | | | | | | |  |  |  |  |  |  |  |  |  |  |  |  |  |  |  |  |  |  |  |  |  |  |  |  |  |  |  |  |  |  |  |  |  |  |  |  |  |  |  |  |  |  |  |  |  |  |  |  |  |  |  |  |  |  |  |  |  |  |  |  |  |  |  |  |  |  |  |  |  |  |  |  |  |  |  |  |  |  |  |  |  |  |  |  |  |  |  |  |  |  |  |  |  |  |  |  |  |  |
| 3c | | | | | | | | | | | uncharacterized protein LOC100801737 | | | | | | | | | | | | | | | | | | | | | | | | | | | | | Glyma13g20780.1 | | | | | | | | | | | | | | | | | | | | | | | | | | | | | | | | | | 189 | | | | | | | | | | | | | | | | | | | | | | | | | | | | | | | | | | 3 | | | | | | | | | | | | | | | | | | | | | | | | | | | | | | | 13 | | | | | | | | | | | | | | | | | | | | | | | | | | | | | | | | | | | | | | | | | | 696 | | | | | | | | | | | | | | | | | | | | | | | | | | | | | | | | | | | | | | | | 38.3/5.29 | | | | | | | | | | | | | | | | | | | | | | | | | | | | | | | | | | | | | | | | | | | | | | | | | | | | |  | | | | | | | | | | | | | | | | | | | | | | | | | | | | | | | | | | | |  | | | | | | | | | | | | | | | | | | | | | | | | | | | | | | | | | | | | | | | | | | | | | | | | | | | | | | | | | | | | | | | | | | | | | | | | | | | |  |  |  |  |  |  |  |  |  |  |  |  |  |  |  |  |  |  |  |  |  |  |
|  | | | | | | | | | | | Calc. Mass | | | | | | | | | | | | | | | | | | | | | | | | | | | | | Observ. Mass | | | | | | | | | | | | | | | | | | | | | | | | | | | | | | | | | | Da | | | | | | | | | | | | | | | | | | | | | | | | | | | | | | | | | | ppm | | | | | | | | | | | | | | | | | | | | | | | | | | | | | | | start | | | | | | | | | | | | | | | | | | | | | | | | | | | | | | | | | | | | | | | | | | end | | | | | | | | | | | | | | | | | | | | | | | | | | | | | | | | | | | | | | | | sequence | | | | | | | | | | | | | | | | | | | | | | | | | | | | | | | | | | | | | | | | | | | | | | | | | | | | | | | | | | | | | | | | | | | | | | | | | | | | | | | | | | | | | | | | | | | | | | | | | | | | | | | | | | | | | | | | | | | | | | | | | | | | | | | | | | | | | | | | | | | | | | | | | | | | | | | | | | | | | | | | | | | | |  |  |  |  |  |  |  |  |  |  |  |  |  |  |  |  |  |  |  |  |  |  |
|  | | | | | | | | | | | 1409.6973 | | | | | | | | | | | | | | | | | | | | | | | | | | | | | 1409.697 | | | | | | | | | | | | | | | | | | | | | | | | | | | | | | | | | | 0 | | | | | | | | | | | | | | | | | | | | | | | | | | | | | | | | | | 0 | | | | | | | | | | | | | | | | | | | | | | | | | | | | | | | 130 | | | | | | | | | | | | | | | | | | | | | | | | | | | | | | | | | | | | | | | | | | 142 | | | | | | | | | | | | | | | | | | | | | | | | | | | | | | | | | | | | | | | | SLVANLSAANCYK | | | | | | | | | | | | | | | | | | | | | | | | | | | | | | | | | | | | | | | | | | | | | | | | | | | | | | | | | | | | | | | | | | | | | | | | | | | | | | | | | | | | | | | | | | | | | | | | | | | | | | | | | | | | | | | | | | | | | | | | | | | | | | | | | | | | | | | | | | | | | | | | | | | | | | | | | | | | | | | | | | | | |  |  |  |  |  |  |  |  |  |  |  |  |  |  |  |  |  |  |  |  |  |  |
|  | | | | | | | | | | | 1701.821 | | | | | | | | | | | | | | | | | | | | | | | | | | | | | 1701.814 | | | | | | | | | | | | | | | | | | | | | | | | | | | | | | | | | | 0 | | | | | | | | | | | | | | | | | | | | | | | | | | | | | | | | | | -4 | | | | | | | | | | | | | | | | | | | | | | | | | | | | | | | 229 | | | | | | | | | | | | | | | | | | | | | | | | | | | | | | | | | | | | | | | | | | 243 | | | | | | | | | | | | | | | | | | | | | | | | | | | | | | | | | | | | | | | | AQGWETDNVEEIALK | | | | | | | | | | | | | | | | | | | | | | | | | | | | | | | | | | | | | | | | | | | | | | | | | | | | | | | | | | | | | | | | | | | | | | | | | | | | | | | | | | | | | | | | | | | | | | | | | | | | | | | | | | | | | | | | | | | | | | | | | | | | | | | | | | | | | | | | | | | | | | | | | | | | | | | | | | | | | | | | | | | | |  |  |  |  |  |  |  |  |  |  |  |  |  |  |  |  |  |  |  |  |  |  |
|  | | | | | | | | | | | 1871.9299 | | | | | | | | | | | | | | | | | | | | | | | | | | | | | 1871.927 | | | | | | | | | | | | | | | | | | | | | | | | | | | | | | | | | | 0 | | | | | | | | | | | | | | | | | | | | | | | | | | | | | | | | | | -1 | | | | | | | | | | | | | | | | | | | | | | | | | | | | | | | 257 | | | | | | | | | | | | | | | | | | | | | | | | | | | | | | | | | | | | | | | | | | 274 | | | | | | | | | | | | | | | | | | | | | | | | | | | | | | | | | | | | | | | | ITVITQGADPVCVAEDGK | | | | | | | | | | | | | | | | | | | | | | | | | | | | | | | | | | | | | | | | | | | | | | | | | | | | | | | | | | | | | | | | | | | | | | | | | | | | | | | | | | | | | | | | | | | | | | | | | | | | | | | | | | | | | | | | | | | | | | | | | | | | | | | | | | | | | | | | | | | | | | | | | | | | | | | | | | | | | | | | | | | | |  |  |  |  |  |  |  |  |  |  |  |  |  |  |  |  |  |  |  |  |  |  |
| Spot no. | | | | | | | | | | | Homologous protein | | | | | | | | | | | | | | | | | | | | | | | | | | | | | Accession no. | | | | | | | | | | | | | | | | | | | | | | | | | | | | | | | | | | Score | | | | | | | | | | | | | | | | | | | | | | | | | | | | | | | | | | M.P. | | | | | | | | | | | | | | | | | | | | | | | | | | | | | | | | | | | | | Cov. (%) | | | | | | | | | | | | | | | | | | | | | | | | | | | | | | | | | | | | | | | Blast score | | | | | | | | | | | | | | | | | | | | | | | | | | | | | | | | | | | | | | | | | | Mr (kDa) / pI | | | | | | | | | | | | | | | | | | | | | | | | | | | | | | | | | | | | | | | | | | | | | | | | | | | | | | | | | | | | | | | | | | | | | | | | | | | | | | | Ratio | | | | | | | | | | | | | | | | | | | | | | | | | | | | | | | | | | | | | | | | | | | | | | | | | | | | | | | | | | | | | | | | | | | | | | | | | | | | | | | | |  |  |  |  |  |  |  |  |  |  |  |  |  |  |  |  |  |  |  |  |  |  |
|  |  |  |  |  |  |  |  |  |  |  |  |  |  |  |  |  |  |  |  |  |  |  |  |  |  |  |  |  |  |  |  |  |  |  |  |  |  |  |  |  |  |  |  |  |  |  |  |  |  |  |  |  |  |  |  |  |  |  |  |  |  |  |  |  |  |  |  |  |  |  |  |  |  |  |  |  |  |  |  |  |  |  |  |  |  |  |  |  |  |  |  |  |  |  |  |  |  |  |  |  |  |  |  |  |  |  |  |  |  |  |  |  |  |  |  |  |  |  |  |  |  |  |  |  |  |  |  |  |  |  |  |  |  |  |  |  |  |  |  |  |  |  |  |  |  |  |  |  |  |  |  |  |  |  |  |  |  |  |  |  |  |  |  |  |  |  |  |  |  |  |  |  |  |  |  |  |  |  |  |  |  |  |  |  |  |  |  |  |  |  |  |  |  |  |  |  |  |  |  |  |  |  |  |  |  |  |  |  |  |  |  |  |  |  |  |  |  |  |  |  |  |  |  |  |  | Theo. | | | | | | | | | | | | | | | | | | | | | | | | | | | | | | | | | | | | | | | | | | | | | | | | | | | | | | | | | Exp. | | | | | | | | | | | | | | | | | | | | | |  |  |  |  |  |  |  |  |  |  |  |  |  |  |  |  |  |  |  |  |  |  |  |  |  |  |  |  |  |  |  |  |  |  |  |  |  |  |  |  |  |  |  |  |  |  |  |  |  |  |  |  |  |  |  |  |  |  |  |  |  |  |  |  |  |  |  |  |  |  |  |  |  |  |  |  |  |  |  |  |  |  |  |  |  |  |  |  |  |  |  |  |  |  |  |  |  |  |  |  |  |  |  |
| 3d | | | | | | | | | | | unknown | | | | | | | | | | | | | | | | | | | | | | | | | | | | | Glyma10g34370.1 | | | | | | | | | | | | | | | | | | | | | | | | | | | | | | | | | | 174 | | | | | | | | | | | | | | | | | | | | | | | | | | | | | | | | | | 8 | | | | | | | | | | | | | | | | | | | | | | | | | | | | | | | | | | | | | 20 | | | | | | | | | | | | | | | | | | | | | | | | | | | | | | | | | | | | | | | 723 | | | | | | | | | | | | | | | | | | | | | | | | | | | | | | | | | | | | | | | | | | 42.6/5.52 | | | | | | | | | | | | | | | | | | | | | | | | | | | | | | | | | | | | | | | | | | | | | | | | | | | | | | | | |  | | | | | | | | | | | | | | | | | | | | | |  | | | | | | | | | | | | | | | | | | | | | | | | | | | | | | | | | | | | | | | | | | | | | | | | | | | | | | | | | | | | | | | | | | | | | | | | | | | | | | | | |  |  |  |  |  |  |  |  |  |  |  |  |  |  |  |  |  |  |  |  |  |  |
|  | | | | | | | | | | | Calc. Mass | | | | | | | | | | | | | | | | | | | | | | | | | | | | | Observ. Mass | | | | | | | | | | | | | | | | | | | | | | | | | | | | | | | | | | Da | | | | | | | | | | | | | | | | | | | | | | | | | | | | | | | | | | ppm | | | | | | | | | | | | | | | | | | | | | | | | | | | | | | | | | | | | | start | | | | | | | | | | | | | | | | | | | | | | | | | | | | | | | | | | | | | | | end | | | | | | | | | | | | | | | | | | | | | | | | | | | | | | | | | | | | | | | | | | sequence | | | | | | | | | | | | | | | | | | | | | | | | | | | | | | | | | | | | | | | | | | | | | | | | | | | | | | | | | | | | | | | | | | | | | | | | | | | | | | | | | | | | | | | | | | | | | | | | | | | | | | | | | | | | | | | | | | | | | | | | | | | | | | | | | | | | | | | | | | | | | | | | | | | | | | | | | | | | | | | |  |  |  |  |  |  |  |  |  |  |  |  |  |  |  |  |  |  |  |  |  |  |
|  | | | | | | | | | | | 1153.5516 | | | | | | | | | | | | | | | | | | | | | | | | | | | | | 1153.551 | | | | | | | | | | | | | | | | | | | | | | | | | | | | | | | | | | 0 | | | | | | | | | | | | | | | | | | | | | | | | | | | | | | | | | | -1 | | | | | | | | | | | | | | | | | | | | | | | | | | | | | | | | | | | | | 159 | | | | | | | | | | | | | | | | | | | | | | | | | | | | | | | | | | | | | | | 167 | | | | | | | | | | | | | | | | | | | | | | | | | | | | | | | | | | | | | | | | | | DFDAFDLRR | | | | | | | | | | | | | | | | | | | | | | | | | | | | | | | | | | | | | | | | | | | | | | | | | | | | | | | | | | | | | | | | | | | | | | | | | | | | | | | | | | | | | | | | | | | | | | | | | | | | | | | | | | | | | | | | | | | | | | | | | | | | | | | | | | | | | | | | | | | | | | | | | | | | | | | | | | | | | | | |  |  |  |  |  |  |  |  |  |  |  |  |  |  |  |  |  |  |  |  |  |  |
|  | | | | | | | | | | | 1959.9585 | | | | | | | | | | | | | | | | | | | | | | | | | | | | | 1959.956 | | | | | | | | | | | | | | | | | | | | | | | | | | | | | | | | | | 0 | | | | | | | | | | | | | | | | | | | | | | | | | | | | | | | | | | -1 | | | | | | | | | | | | | | | | | | | | | | | | | | | | | | | | | | | | | 179 | | | | | | | | | | | | | | | | | | | | | | | | | | | | | | | | | | | | | | | 197 | | | | | | | | | | | | | | | | | | | | | | | | | | | | | | | | | | | | | | | | | | AINSNGGVTYIHCTAGLGR | | | | | | | | | | | | | | | | | | | | | | | | | | | | | | | | | | | | | | | | | | | | | | | | | | | | | | | | | | | | | | | | | | | | | | | | | | | | | | | | | | | | | | | | | | | | | | | | | | | | | | | | | | | | | | | | | | | | | | | | | | | | | | | | | | | | | | | | | | | | | | | | | | | | | | | | | | | | | | | |  |  |  |  |  |  |  |  |  |  |  |  |  |  |  |  |  |  |  |  |  |  |
|  | | | | | | | | | | | 1175.6398 | | | | | | | | | | | | | | | | | | | | | | | | | | | | | 1175.64 | | | | | | | | | | | | | | | | | | | | | | | | | | | | | | | | | | 0 | | | | | | | | | | | | | | | | | | | | | | | | | | | | | | | | | | 0 | | | | | | | | | | | | | | | | | | | | | | | | | | | | | | | | | | | | | 236 | | | | | | | | | | | | | | | | | | | | | | | | | | | | | | | | | | | | | | | 247 | | | | | | | | | | | | | | | | | | | | | | | | | | | | | | | | | | | | | | | | | | SATADILTGLSK | | | | | | | | | | | | | | | | | | | | | | | | | | | | | | | | | | | | | | | | | | | | | | | | | | | | | | | | | | | | | | | | | | | | | | | | | | | | | | | | | | | | | | | | | | | | | | | | | | | | | | | | | | | | | | | | | | | | | | | | | | | | | | | | | | | | | | | | | | | | | | | | | | | | | | | | | | | | | | | |  |  |  |  |  |  |  |  |  |  |  |  |  |  |  |  |  |  |  |  |  |  |
|  | | | | | | | | | | | 1303.7347 | | | | | | | | | | | | | | | | | | | | | | | | | | | | | 1303.735 | | | | | | | | | | | | | | | | | | | | | | | | | | | | | | | | | | 0 | | | | | | | | | | | | | | | | | | | | | | | | | | | | | | | | | | 0 | | | | | | | | | | | | | | | | | | | | | | | | | | | | | | | | | | | | | 236 | | | | | | | | | | | | | | | | | | | | | | | | | | | | | | | | | | | | | | | 248 | | | | | | | | | | | | | | | | | | | | | | | | | | | | | | | | | | | | | | | | | | SATADILTGLSKK | | | | | | | | | | | | | | | | | | | | | | | | | | | | | | | | | | | | | | | | | | | | | | | | | | | | | | | | | | | | | | | | | | | | | | | | | | | | | | | | | | | | | | | | | | | | | | | | | | | | | | | | | | | | | | | | | | | | | | | | | | | | | | | | | | | | | | | | | | | | | | | | | | | | | | | | | | | | | | | |  |  |  |  |  |  |  |  |  |  |  |  |  |  |  |  |  |  |  |  |  |  |
|  | | | | | | | | | | | 960.4916 | | | | | | | | | | | | | | | | | | | | | | | | | | | | | 960.4926 | | | | | | | | | | | | | | | | | | | | | | | | | | | | | | | | | | 0 | | | | | | | | | | | | | | | | | | | | | | | | | | | | | | | | | | 1 | | | | | | | | | | | | | | | | | | | | | | | | | | | | | | | | | | | | | 275 | | | | | | | | | | | | | | | | | | | | | | | | | | | | | | | | | | | | | | | 282 | | | | | | | | | | | | | | | | | | | | | | | | | | | | | | | | | | | | | | | | | | IPLNFDDK | | | | | | | | | | | | | | | | | | | | | | | | | | | | | | | | | | | | | | | | | | | | | | | | | | | | | | | | | | | | | | | | | | | | | | | | | | | | | | | | | | | | | | | | | | | | | | | | | | | | | | | | | | | | | | | | | | | | | | | | | | | | | | | | | | | | | | | | | | | | | | | | | | | | | | | | | | | | | | | |  |  |  |  |  |  |  |  |  |  |  |  |  |  |  |  |  |  |  |  |  |  |
|  | | | | | | | | | | | 1239.6023 | | | | | | | | | | | | | | | | | | | | | | | | | | | | | 1239.601 | | | | | | | | | | | | | | | | | | | | | | | | | | | | | | | | | | 0 | | | | | | | | | | | | | | | | | | | | | | | | | | | | | | | | | | -1 | | | | | | | | | | | | | | | | | | | | | | | | | | | | | | | | | | | | | 291 | | | | | | | | | | | | | | | | | | | | | | | | | | | | | | | | | | | | | | | 300 | | | | | | | | | | | | | | | | | | | | | | | | | | | | | | | | | | | | | | | | | | ELPEGLYEYK | | | | | | | | | | | | | | | | | | | | | | | | | | | | | | | | | | | | | | | | | | | | | | | | | | | | | | | | | | | | | | | | | | | | | | | | | | | | | | | | | | | | | | | | | | | | | | | | | | | | | | | | | | | | | | | | | | | | | | | | | | | | | | | | | | | | | | | | | | | | | | | | | | | | | | | | | | | | | | | |  |  |  |  |  |  |  |  |  |  |  |  |  |  |  |  |  |  |  |  |  |  |
|  | | | | | | | | | | | 1857.9068 | | | | | | | | | | | | | | | | | | | | | | | | | | | | | 1857.905 | | | | | | | | | | | | | | | | | | | | | | | | | | | | | | | | | | 0 | | | | | | | | | | | | | | | | | | | | | | | | | | | | | | | | | | -1 | | | | | | | | | | | | | | | | | | | | | | | | | | | | | | | | | | | | | 344 | | | | | | | | | | | | | | | | | | | | | | | | | | | | | | | | | | | | | | | 359 | | | | | | | | | | | | | | | | | | | | | | | | | | | | | | | | | | | | | | | | | | RLTADDPDLTTDEQLR | | | | | | | | | | | | | | | | | | | | | | | | | | | | | | | | | | | | | | | | | | | | | | | | | | | | | | | | | | | | | | | | | | | | | | | | | | | | | | | | | | | | | | | | | | | | | | | | | | | | | | | | | | | | | | | | | | | | | | | | | | | | | | | | | | | | | | | | | | | | | | | | | | | | | | | | | | | | | | | |  |  |  |  |  |  |  |  |  |  |  |  |  |  |  |  |  |  |  |  |  |  |
|  | | | | | | | | | | | 1701.8057 | | | | | | | | | | | | | | | | | | | | | | | | | | | | | 1701.804 | | | | | | | | | | | | | | | | | | | | | | | | | | | | | | | | | | 0 | | | | | | | | | | | | | | | | | | | | | | | | | | | | | | | | | | -1 | | | | | | | | | | | | | | | | | | | | | | | | | | | | | | | | | | | | | 345 | | | | | | | | | | | | | | | | | | | | | | | | | | | | | | | | | | | | | | | 359 | | | | | | | | | | | | | | | | | | | | | | | | | | | | | | | | | | | | | | | | | | LTADDPDLTTDEQLR | | | | | | | | | | | | | | | | | | | | | | | | | | | | | | | | | | | | | | | | | | | | | | | | | | | | | | | | | | | | | | | | | | | | | | | | | | | | | | | | | | | | | | | | | | | | | | | | | | | | | | | | | | | | | | | | | | | | | | | | | | | | | | | | | | | | | | | | | | | | | | | | | | | | | | | | | | | | | | | |  |  |  |  |  |  |  |  |  |  |  |  |  |  |  |  |  |  |  |  |  |  |
| Spot no. | | | | | | | | | | | Homologous protein | | | | | | | | | | | | | | | | | | | | | | | | | | | | | Accession no. | | | | | | | | | | | | | | | | | | | | | | | | | | | | | | | | | | Score | | | | | | | | | | | | | | | | | | | | | | | | | | | | | | | | | | M.P. | | | | | | | | | | | | | | | | | | | | | | | | | | | | | | | | | | | | | Cov. (%) | | | | | | | | | | | | | | | | | | | | | | | | | | | | | | | | | | | | | | | Blast score | | | | | | | | | | | | | | | | | | | | | | | | | | | | | | | | | | | | | | | | | | Mr (kDa) / pI | | | | | | | | | | | | | | | | | | | | | | | | | | | | | | | | | | | | | | | | | | | | | | | | | | | | | | | | | | | | | | | | | | | | | | | | | | | | | | | | | | | | | | | | | | | | | | | | | | | | | | | | | | | | | | | | | | | | | | | | | | | | | | | | | | | | | | | Ratio | | | | | | | | | | | | | | | | | | |  |  |  |  |  |  |  |  |  |  |  |  |  |  |  |  |  |  |  |  |  |  |  |  |  |  |  |  |
|  |  |  |  |  |  |  |  |  |  |  |  |  |  |  |  |  |  |  |  |  |  |  |  |  |  |  |  |  |  |  |  |  |  |  |  |  |  |  |  |  |  |  |  |  |  |  |  |  |  |  |  |  |  |  |  |  |  |  |  |  |  |  |  |  |  |  |  |  |  |  |  |  |  |  |  |  |  |  |  |  |  |  |  |  |  |  |  |  |  |  |  |  |  |  |  |  |  |  |  |  |  |  |  |  |  |  |  |  |  |  |  |  |  |  |  |  |  |  |  |  |  |  |  |  |  |  |  |  |  |  |  |  |  |  |  |  |  |  |  |  |  |  |  |  |  |  |  |  |  |  |  |  |  |  |  |  |  |  |  |  |  |  |  |  |  |  |  |  |  |  |  |  |  |  |  |  |  |  |  |  |  |  |  |  |  |  |  |  |  |  |  |  |  |  |  |  |  |  |  |  |  |  |  |  |  |  |  |  |  |  |  |  |  |  |  |  |  |  |  |  |  |  |  |  |  | Theo. | | | | | | | | | | | | | | | | | | | | | | | | | | | | | | | | | | | | | | | | | | | | | | | | | | | | | | | | | Exp. | | | | | | | | | | | | | | | | | | | | | | | | | | | | | | | | | | | | | | | | | | | | | | | | | | | | | | | | | | | | | | | | | | | | | | | | | | | | | |  |  |  |  |  |  |  |  |  |  |  |  |  |  |  |  |  |  |  |  |  |  |  |  |  |  |  |  |  |  |  |  |  |  |  |  |  |  |  |  |  |  |  |  |  |  |  |
| 3e | | | | | | | | | | | unknown | | | | | | | | | | | | | | | | | | | | | | | | | | | | | Glyma02g46530.1 | | | | | | | | | | | | | | | | | | | | | | | | | | | | | | | | | | 146 | | | | | | | | | | | | | | | | | | | | | | | | | | | | | | | | | | 4 | | | | | | | | | | | | | | | | | | | | | | | | | | | | | | | | | | | | | 13 | | | | | | | | | | | | | | | | | | | | | | | | | | | | | | | | | | | | | | | 632 | | | | | | | | | | | | | | | | | | | | | | | | | | | | | | | | | | | | | | | | | | 44.4/6.16 | | | | | | | | | | | | | | | | | | | | | | | | | | | | | | | | | | | | | | | | | | | | | | | | | | | | | | | | |  | | | | | | | | | | | | | | | | | | | | | | | | | | | | | | | | | | | | | | | | | | | | | | | | | | | | | | | | | | | | | | | | | | | | | | | | | | | | | |  | | | | | | | | | | | | | | | | | | |  |  |  |  |  |  |  |  |  |  |  |  |  |  |  |  |  |  |  |  |  |  |  |  |  |  |  |  |
|  | | | | | | | | | | | Calc. Mass | | | | | | | | | | | | | | | | | | | | | | | | | | | | | Observ. Mass | | | | | | | | | | | | | | | | | | | | | | | | | | | | | | | | | | Da | | | | | | | | | | | | | | | | | | | | | | | | | | | | | | | | | | ppm | | | | | | | | | | | | | | | | | | | | | | | | | | | | | | | | | | | | | start | | | | | | | | | | | | | | | | | | | | | | | | | | | | | | | | | | | | | | | end | | | | | | | | | | | | | | | | | | | | | | | | | | | | | | | | | | | | | | | | | | sequence | | | | | | | | | | | | | | | | | | | | | | | | | | | | | | | | | | | | | | | | | | | | | | | | | | | | | | | | | | | | | | | | | | | | | | | | | | | | | | | | | | | | | | | | | | | | | | | | | | | | | | | | | | | | | | | | | | | | | | | | | | | | | | | | | | | | | | | | | | | | | | | | | | | | | | | | | |  |  |  |  |  |  |  |  |  |  |  |  |  |  |  |  |  |  |  |  |  |  |  |  |  |  |  |  |
|  | | | | | | | | | | | 1523.8532 | | | | | | | | | | | | | | | | | | | | | | | | | | | | | 1523.859 | | | | | | | | | | | | | | | | | | | | | | | | | | | | | | | | | | 0 | | | | | | | | | | | | | | | | | | | | | | | | | | | | | | | | | | 4 | | | | | | | | | | | | | | | | | | | | | | | | | | | | | | | | | | | | | 66 | | | | | | | | | | | | | | | | | | | | | | | | | | | | | | | | | | | | | | | 81 | | | | | | | | | | | | | | | | | | | | | | | | | | | | | | | | | | | | | | | | | | IKVVGIGGGGNNAVNR | | | | | | | | | | | | | | | | | | | | | | | | | | | | | | | | | | | | | | | | | | | | | | | | | | | | | | | | | | | | | | | | | | | | | | | | | | | | | | | | | | | | | | | | | | | | | | | | | | | | | | | | | | | | | | | | | | | | | | | | | | | | | | | | | | | | | | | | | | | | | | | | | | | | | | | | | |  |  |  |  |  |  |  |  |  |  |  |  |  |  |  |  |  |  |  |  |  |  |  |  |  |  |  |  |
|  | | | | | | | | | | | 1854.9072 | | | | | | | | | | | | | | | | | | | | | | | | | | | | | 1854.904 | | | | | | | | | | | | | | | | | | | | | | | | | | | | | | | | | | 0 | | | | | | | | | | | | | | | | | | | | | | | | | | | | | | | | | | -2 | | | | | | | | | | | | | | | | | | | | | | | | | | | | | | | | | | | | | 120 | | | | | | | | | | | | | | | | | | | | | | | | | | | | | | | | | | | | | | | 138 | | | | | | | | | | | | | | | | | | | | | | | | | | | | | | | | | | | | | | | | | | GLGTGGNPLLGEQAAEESR | | | | | | | | | | | | | | | | | | | | | | | | | | | | | | | | | | | | | | | | | | | | | | | | | | | | | | | | | | | | | | | | | | | | | | | | | | | | | | | | | | | | | | | | | | | | | | | | | | | | | | | | | | | | | | | | | | | | | | | | | | | | | | | | | | | | | | | | | | | | | | | | | | | | | | | | | |  |  |  |  |  |  |  |  |  |  |  |  |  |  |  |  |  |  |  |  |  |  |  |  |  |  |  |  |
|  | | | | | | | | | | | 1854.9072 | | | | | | | | | | | | | | | | | | | | | | | | | | | | | 1854.905 | | | | | | | | | | | | | | | | | | | | | | | | | | | | | | | | | | 0 | | | | | | | | | | | | | | | | | | | | | | | | | | | | | | | | | | -1 | | | | | | | | | | | | | | | | | | | | | | | | | | | | | | | | | | | | | 120 | | | | | | | | | | | | | | | | | | | | | | | | | | | | | | | | | | | | | | | 138 | | | | | | | | | | | | | | | | | | | | | | | | | | | | | | | | | | | | | | | | | | GLGTGGNPLLGEQAAEESR | | | | | | | | | | | | | | | | | | | | | | | | | | | | | | | | | | | | | | | | | | | | | | | | | | | | | | | | | | | | | | | | | | | | | | | | | | | | | | | | | | | | | | | | | | | | | | | | | | | | | | | | | | | | | | | | | | | | | | | | | | | | | | | | | | | | | | | | | | | | | | | | | | | | | | | | | |  |  |  |  |  |  |  |  |  |  |  |  |  |  |  |  |  |  |  |  |  |  |  |  |  |  |  |  |
|  | | | | | | | | | | | 1162.5982 | | | | | | | | | | | | | | | | | | | | | | | | | | | | | 1162.599 | | | | | | | | | | | | | | | | | | | | | | | | | | | | | | | | | | 0 | | | | | | | | | | | | | | | | | | | | | | | | | | | | | | | | | | 1 | | | | | | | | | | | | | | | | | | | | | | | | | | | | | | | | | | | | | 196 | | | | | | | | | | | | | | | | | | | | | | | | | | | | | | | | | | | | | | | 205 | | | | | | | | | | | | | | | | | | | | | | | | | | | | | | | | | | | | | | | | | | SLQAFEAIER | | | | | | | | | | | | | | | | | | | | | | | | | | | | | | | | | | | | | | | | | | | | | | | | | | | | | | | | | | | | | | | | | | | | | | | | | | | | | | | | | | | | | | | | | | | | | | | | | | | | | | | | | | | | | | | | | | | | | | | | | | | | | | | | | | | | | | | | | | | | | | | | | | | | | | | | | |  |  |  |  |  |  |  |  |  |  |  |  |  |  |  |  |  |  |  |  |  |  |  |  |  |  |  |  |
|  | | | | | | | | | | | 1367.7409 | | | | | | | | | | | | | | | | | | | | | | | | | | | | | 1367.738 | | | | | | | | | | | | | | | | | | | | | | | | | | | | | | | | | | 0 | | | | | | | | | | | | | | | | | | | | | | | | | | | | | | | | | | -2 | | | | | | | | | | | | | | | | | | | | | | | | | | | | | | | | | | | | | 209 | | | | | | | | | | | | | | | | | | | | | | | | | | | | | | | | | | | | | | | 220 | | | | | | | | | | | | | | | | | | | | | | | | | | | | | | | | | | | | | | | | | | NVDTLIVIPNDR | | | | | | | | | | | | | | | | | | | | | | | | | | | | | | | | | | | | | | | | | | | | | | | | | | | | | | | | | | | | | | | | | | | | | | | | | | | | | | | | | | | | | | | | | | | | | | | | | | | | | | | | | | | | | | | | | | | | | | | | | | | | | | | | | | | | | | | | | | | | | | | | | | | | | | | | | |  |  |  |  |  |  |  |  |  |  |  |  |  |  |  |  |  |  |  |  |  |  |  |  |  |  |  |  |
| Spot no. | | | | | | | | | | | Homologous protein | | | | | | | | | | | | | | | | | | | | | | | | | | | | | Accession no. | | | | | | | | | | | | | | | | | | | | | | | | | | | | | | | | | | Score | | | | | | | | | | | | | | | | | | | | | | | | | | | | | | | M.P. | | | | | | | | | | | | | | | | | | | | | | | | | | | | | | | | | | | | | | Cov. (%) | | | | | | | | | | | | | | | | | | | | | | | | | | | | | | | | | | | | | | | | Blast score | | | | | | | | | | | | | | | | | | | | | | | | | | | | | | | | | | | | | | | | | Mr (kDa) / pI | | | | | | | | | | | | | | | | | | | | | | | | | | | | | | | | | | | | | | | | | | | | | | | | | | | | | | | | | | | | | | | | | | | | | | | | | | | | | | | | | | | | | | | | | | | | | | | | | | | | | | | | | | | | | | | | | | | | | | | | | | | | | | | | | Ratio | | | | | | | | | | | | | | | | | | | | | | | | | | | | | | | | | |  |  |  |  |  |  |  |  |  |  |  |  |  |  |  |  |  |  |  |  |  |
|  |  |  |  |  |  |  |  |  |  |  |  |  |  |  |  |  |  |  |  |  |  |  |  |  |  |  |  |  |  |  |  |  |  |  |  |  |  |  |  |  |  |  |  |  |  |  |  |  |  |  |  |  |  |  |  |  |  |  |  |  |  |  |  |  |  |  |  |  |  |  |  |  |  |  |  |  |  |  |  |  |  |  |  |  |  |  |  |  |  |  |  |  |  |  |  |  |  |  |  |  |  |  |  |  |  |  |  |  |  |  |  |  |  |  |  |  |  |  |  |  |  |  |  |  |  |  |  |  |  |  |  |  |  |  |  |  |  |  |  |  |  |  |  |  |  |  |  |  |  |  |  |  |  |  |  |  |  |  |  |  |  |  |  |  |  |  |  |  |  |  |  |  |  |  |  |  |  |  |  |  |  |  |  |  |  |  |  |  |  |  |  |  |  |  |  |  |  |  |  |  |  |  |  |  |  |  |  |  |  |  |  |  |  |  |  |  |  |  |  |  |  |  |  | Theo. | | | | | | | | | | | | | | | | | | | | | | | | | | | | | | | | | | | | | | | | | | | | | | | | | | | | Exp. | | | | | | | | | | | | | | | | | | | | | | | | | | | | | | | | | | | | | | | | | | | | | | | | | | | | | | | | | | | | | | | | | | | | | | | | | | | | |  |  |  |  |  |  |  |  |  |  |  |  |  |  |  |  |  |  |  |  |  |  |  |  |  |  |  |  |  |  |  |  |  |  |  |  |  |  |  |  |  |  |  |  |  |  |  |  |  |  |  |  |  |  |  |
| 3f | | | | | | | | | | | Serine/threonine protein phosphatase | | | | | | | | | | | | | | | | | | | | | | | | | | | | | Glyma03g36510.1 | | | | | | | | | | | | | | | | | | | | | | | | | | | | | | | | | | 145 | | | | | | | | | | | | | | | | | | | | | | | | | | | | | | | 3 | | | | | | | | | | | | | | | | | | | | | | | | | | | | | | | | | | | | | | 13 | | | | | | | | | | | | | | | | | | | | | | | | | | | | | | | | | | | | | | | | 611 | | | | | | | | | | | | | | | | | | | | | | | | | | | | | | | | | | | | | | | | | 36.5/5.12 | | | | | | | | | | | | | | | | | | | | | | | | | | | | | | | | | | | | | | | | | | | | | | | | | | | |  | | | | | | | | | | | | | | | | | | | | | | | | | | | | | | | | | | | | | | | | | | | | | | | | | | | | | | | | | | | | | | | | | | | | | | | | | | | | |  | | | | | | | | | | | | | | | | | | | | | | | | | | | | | | | | | |  |  |  |  |  |  |  |  |  |  |  |  |  |  |  |  |  |  |  |  |  |
|  | | | | | | | | | | | Calc. Mass | | | | | | | | | | | | | | | | | | | | | | | | | | | | | Observ. Mass | | | | | | | | | | | | | | | | | | | | | | | | | | | | | | | | | | Da | | | | | | | | | | | | | | | | | | | | | | | | | | | | | | | ppm | | | | | | | | | | | | | | | | | | | | | | | | | | | | | | | | | | | | | | start | | | | | | | | | | | | | | | | | | | | | | | | | | | | | | | | | | | | | | | | end | | | | | | | | | | | | | | | | | | | | | | | | | | | | | | | | | | | | | | | | | sequence | | | | | | | | | | | | | | | | | | | | | | | | | | | | | | | | | | | | | | | | | | | | | | | | | | | | | | | | | | | | | | | | | | | | | | | | | | | | | | | | | | | | | | | | | | | | | | | | | | | | | | | | | | | | | | | | | | | | | | | | | | | | | | | | | | | | | | | | | | | | | | | | | | | | | | | | | | | | | | | | | | |  |  |  |  |  |  |  |  |  |  |  |  |  |  |  |  |  |  |  |  |  |
|  | | | | | | | | | | | 1669.8886 | | | | | | | | | | | | | | | | | | | | | | | | | | | | | 1669.888 | | | | | | | | | | | | | | | | | | | | | | | | | | | | | | | | | | 0 | | | | | | | | | | | | | | | | | | | | | | | | | | | | | | | -1 | | | | | | | | | | | | | | | | | | | | | | | | | | | | | | | | | | | | | | 39 | | | | | | | | | | | | | | | | | | | | | | | | | | | | | | | | | | | | | | | | 53 | | | | | | | | | | | | | | | | | | | | | | | | | | | | | | | | | | | | | | | | | AKEILTDESNVQPVK | | | | | | | | | | | | | | | | | | | | | | | | | | | | | | | | | | | | | | | | | | | | | | | | | | | | | | | | | | | | | | | | | | | | | | | | | | | | | | | | | | | | | | | | | | | | | | | | | | | | | | | | | | | | | | | | | | | | | | | | | | | | | | | | | | | | | | | | | | | | | | | | | | | | | | | | | | | | | | | | | | |  |  |  |  |  |  |  |  |  |  |  |  |  |  |  |  |  |  |  |  |  |
|  | | | | | | | | | | | 1880.771 | | | | | | | | | | | | | | | | | | | | | | | | | | | | | 1880.767 | | | | | | | | | | | | | | | | | | | | | | | | | | | | | | | | | | 0 | | | | | | | | | | | | | | | | | | | | | | | | | | | | | | | -2 | | | | | | | | | | | | | | | | | | | | | | | | | | | | | | | | | | | | | | 79 | | | | | | | | | | | | | | | | | | | | | | | | | | | | | | | | | | | | | | | | 93 | | | | | | | | | | | | | | | | | | | | | | | | | | | | | | | | | | | | | | | | | CPDTNYLFMGDYVDR | | | | | | | | | | | | | | | | | | | | | | | | | | | | | | | | | | | | | | | | | | | | | | | | | | | | | | | | | | | | | | | | | | | | | | | | | | | | | | | | | | | | | | | | | | | | | | | | | | | | | | | | | | | | | | | | | | | | | | | | | | | | | | | | | | | | | | | | | | | | | | | | | | | | | | | | | | | | | | | | | | |  |  |  |  |  |  |  |  |  |  |  |  |  |  |  |  |  |  |  |  |  |
|  | | | | | | | | | | | 1588.7708 | | | | | | | | | | | | | | | | | | | | | | | | | | | | | 1588.769 | | | | | | | | | | | | | | | | | | | | | | | | | | | | | | | | | | 0 | | | | | | | | | | | | | | | | | | | | | | | | | | | | | | | -1 | | | | | | | | | | | | | | | | | | | | | | | | | | | | | | | | | | | | | | 260 | | | | | | | | | | | | | | | | | | | | | | | | | | | | | | | | | | | | | | | | 272 | | | | | | | | | | | | | | | | | | | | | | | | | | | | | | | | | | | | | | | | | VVTIFSAPNYCYR | | | | | | | | | | | | | | | | | | | | | | | | | | | | | | | | | | | | | | | | | | | | | | | | | | | | | | | | | | | | | | | | | | | | | | | | | | | | | | | | | | | | | | | | | | | | | | | | | | | | | | | | | | | | | | | | | | | | | | | | | | | | | | | | | | | | | | | | | | | | | | | | | | | | | | | | | | | | | | | | | | |  |  |  |  |  |  |  |  |  |  |  |  |  |  |  |  |  |  |  |  |  |
| Spot no. | | | | | | | | | | | Homologous protein | | | | | | | | | | | | | | | | | | | | | | | | | | | | | Accession no. | | | | | | | | | | | | | | | | | | | | | | | | | | | | | | | | | | Score | | | | | | | | | | | | | | | | | | | | | | | | | | | | | | | M.P. | | | | | | | | | | | | | | | | | | | | | | | | | | | | | | | | | | | | | | Cov. (%) | | | | | | | | | | | | | | | | | | | | | | | | | | | | | | | | | | | | | | | | Blast score | | | | | | | | | | | | | | | | | | | | | | | | | | | | | | | | | | | | | | | | | Mr (kDa) / pI | | | | | | | | | | | | | | | | | | | | | | | | | | | | | | | | | | | | | | | | | | | | | | | | | | | | | | | | | | | | | | | | | | | | | | | | | | | | | | | | | | | | | | | | | | | | | | | | Ratio | | | | | | | | | | | | | | | | | | | | | | | | | | | | | | | | | | | | | | | | | | | | | | | | | | | | | | | | | | | | |  |  |  |  |  |  |  |  |  |  |  |  |  |  |  |  |  |  |  |  |  |  |  |  |  |  |  |
|  |  |  |  |  |  |  |  |  |  |  |  |  |  |  |  |  |  |  |  |  |  |  |  |  |  |  |  |  |  |  |  |  |  |  |  |  |  |  |  |  |  |  |  |  |  |  |  |  |  |  |  |  |  |  |  |  |  |  |  |  |  |  |  |  |  |  |  |  |  |  |  |  |  |  |  |  |  |  |  |  |  |  |  |  |  |  |  |  |  |  |  |  |  |  |  |  |  |  |  |  |  |  |  |  |  |  |  |  |  |  |  |  |  |  |  |  |  |  |  |  |  |  |  |  |  |  |  |  |  |  |  |  |  |  |  |  |  |  |  |  |  |  |  |  |  |  |  |  |  |  |  |  |  |  |  |  |  |  |  |  |  |  |  |  |  |  |  |  |  |  |  |  |  |  |  |  |  |  |  |  |  |  |  |  |  |  |  |  |  |  |  |  |  |  |  |  |  |  |  |  |  |  |  |  |  |  |  |  |  |  |  |  |  |  |  |  |  |  |  |  |  |  |  | Theo. | | | | | | | | | | | | | | | | | | | | | | | | | | | | | | | | | | | | | | | | | | | | | | | | | | | | Exp. | | | | | | | | | | | | | | | | | | | | | | | | | | | | | | | | | | | | | | | | | | | |  |  |  |  |  |  |  |  |  |  |  |  |  |  |  |  |  |  |  |  |  |  |  |  |  |  |  |  |  |  |  |  |  |  |  |  |  |  |  |  |  |  |  |  |  |  |  |  |  |  |  |  |  |  |  |  |  |  |  |  |  |  |  |  |  |  |  |  |  |  |  |  |  |  |  |  |  |  |  |  |  |  |  |  |  |  |  |  |
| 3g | | | | | | | | | | | Pectate lyase | | | | | | | | | | | | | | | | | | | | | | | | | | | | | Glyma11g37610.1 | | | | | | | | | | | | | | | | | | | | | | | | | | | | | | | | | | 139 | | | | | | | | | | | | | | | | | | | | | | | | | | | | | | | 3 | | | | | | | | | | | | | | | | | | | | | | | | | | | | | | | | | | | | | | 10 | | | | | | | | | | | | | | | | | | | | | | | | | | | | | | | | | | | | | | | | 691 | | | | | | | | | | | | | | | | | | | | | | | | | | | | | | | | | | | | | | | | | 45.1/8.91 | | | | | | | | | | | | | | | | | | | | | | | | | | | | | | | | | | | | | | | | | | | | | | | | | | | |  | | | | | | | | | | | | | | | | | | | | | | | | | | | | | | | | | | | | | | | | | | | |  | | | | | | | | | | | | | | | | | | | | | | | | | | | | | | | | | | | | | | | | | | | | | | | | | | | | | | | | | | | | |  |  |  |  |  |  |  |  |  |  |  |  |  |  |  |  |  |  |  |  |  |  |  |  |  |  |  |
|  | | | | | | | | | | | Calc. Mass | | | | | | | | | | | | | | | | | | | | | | | | | | | | | Observ. Mass | | | | | | | | | | | | | | | | | | | | | | | | | | | | | | | | | | Da | | | | | | | | | | | | | | | | | | | | | | | | | | | | | | | ppm | | | | | | | | | | | | | | | | | | | | | | | | | | | | | | | | | | | | | | start | | | | | | | | | | | | | | | | | | | | | | | | | | | | | | | | | | | | | | | | end | | | | | | | | | | | | | | | | | | | | | | | | | | | | | | | | | | | | | | | | | sequence | | | | | | | | | | | | | | | | | | | | | | | | | | | | | | | | | | | | | | | | | | | | | | | | | | | | | | | | | | | | | | | | | | | | | | | | | | | | | | | | | | | | | | | | | | | | | | | | | | | | | | | | | | | | | | | | | | | | | | | | | | | | | | | | | | | | | | | | | | | | | | | | | | | | | | | | | | | | |  |  |  |  |  |  |  |  |  |  |  |  |  |  |  |  |  |  |  |  |  |  |  |  |  |  |  |
|  | | | | | | | | | | | 1391.6868 | | | | | | | | | | | | | | | | | | | | | | | | | | | | | 1391.687 | | | | | | | | | | | | | | | | | | | | | | | | | | | | | | | | | | 0 | | | | | | | | | | | | | | | | | | | | | | | | | | | | | | | 0 | | | | | | | | | | | | | | | | | | | | | | | | | | | | | | | | | | | | | | 249 | | | | | | | | | | | | | | | | | | | | | | | | | | | | | | | | | | | | | | | | 260 | | | | | | | | | | | | | | | | | | | | | | | | | | | | | | | | | | | | | | | | | VMLLGHSDTFTR | | | | | | | | | | | | | | | | | | | | | | | | | | | | | | | | | | | | | | | | | | | | | | | | | | | | | | | | | | | | | | | | | | | | | | | | | | | | | | | | | | | | | | | | | | | | | | | | | | | | | | | | | | | | | | | | | | | | | | | | | | | | | | | | | | | | | | | | | | | | | | | | | | | | | | | | | | | | |  |  |  |  |  |  |  |  |  |  |  |  |  |  |  |  |  |  |  |  |  |  |  |  |  |  |  |
|  | | | | | | | | | | | 1806.8683 | | | | | | | | | | | | | | | | | | | | | | | | | | | | | 1806.868 | | | | | | | | | | | | | | | | | | | | | | | | | | | | | | | | | | 0 | | | | | | | | | | | | | | | | | | | | | | | | | | | | | | | 0 | | | | | | | | | | | | | | | | | | | | | | | | | | | | | | | | | | | | | | 301 | | | | | | | | | | | | | | | | | | | | | | | | | | | | | | | | | | | | | | | | 318 | | | | | | | | | | | | | | | | | | | | | | | | | | | | | | | | | | | | | | | | | MYAIGGSAAPTINSQGNR | | | | | | | | | | | | | | | | | | | | | | | | | | | | | | | | | | | | | | | | | | | | | | | | | | | | | | | | | | | | | | | | | | | | | | | | | | | | | | | | | | | | | | | | | | | | | | | | | | | | | | | | | | | | | | | | | | | | | | | | | | | | | | | | | | | | | | | | | | | | | | | | | | | | | | | | | | | | |  |  |  |  |  |  |  |  |  |  |  |  |  |  |  |  |  |  |  |  |  |  |  |  |  |  |  |
|  | | | | | | | | | | | 1822.8632 | | | | | | | | | | | | | | | | | | | | | | | | | | | | | 1822.86 | | | | | | | | | | | | | | | | | | | | | | | | | | | | | | | | | | 0 | | | | | | | | | | | | | | | | | | | | | | | | | | | | | | | -1 | | | | | | | | | | | | | | | | | | | | | | | | | | | | | | | | | | | | | | 301 | | | | | | | | | | | | | | | | | | | | | | | | | | | | | | | | | | | | | | | | 318 | | | | | | | | | | | | | | | | | | | | | | | | | | | | | | | | | | | | | | | | | MYAIGGSAAPTINSQGNR | | | | | | | | | | | | | | | | | | | | | | | | | | | | | | | | | | | | | | | | | | | | | | | | | | | | | | | | | | | | | | | | | | | | | | | | | | | | | | | | | | | | | | | | | | | | | | | | | | | | | | | | | | | | | | | | | | | | | | | | | | | | | | | | | | | | | | | | | | | | | | | | | | | | | | | | | | | | |  |  |  |  |  |  |  |  |  |  |  |  |  |  |  |  |  |  |  |  |  |  |  |  |  |  |  |
|  | | | | | | | | | | | 1622.8304 | | | | | | | | | | | | | | | | | | | | | | | | | | | | | 1622.83 | | | | | | | | | | | | | | | | | | | | | | | | | | | | | | | | | | 0 | | | | | | | | | | | | | | | | | | | | | | | | | | | | | | | 0 | | | | | | | | | | | | | | | | | | | | | | | | | | | | | | | | | | | | | | 319 | | | | | | | | | | | | | | | | | | | | | | | | | | | | | | | | | | | | | | | | 332 | | | | | | | | | | | | | | | | | | | | | | | | | | | | | | | | | | | | | | | | | FLAPNDNTFKEVTK | | | | | | | | | | | | | | | | | | | | | | | | | | | | | | | | | | | | | | | | | | | | | | | | | | | | | | | | | | | | | | | | | | | | | | | | | | | | | | | | | | | | | | | | | | | | | | | | | | | | | | | | | | | | | | | | | | | | | | | | | | | | | | | | | | | | | | | | | | | | | | | | | | | | | | | | | | | | |  |  |  |  |  |  |  |  |  |  |  |  |  |  |  |  |  |  |  |  |  |  |  |  |  |  |  |
| Spot no. | | | | | | | | | | | Homologous protein | | | | | | | | | | | | | | | | | | | | | | | | | | | | | Accession no. | | | | | | | | | | | | | | | | | | | | | | | | | | | | | | | | | | Score | | | | | | | | | | | | | | | | | | | | | | | | | | | | | | | M.P. | | | | | | | | | | | | | | | | | | | | | | | | | | | | | | | | | | | | | | Cov. (%) | | | | | | | | | | | | | | | | | | | | | | | | | | | | | | | | | | | | | | | | Blast score | | | | | | | | | | | | | | | | | | | | | | | | | | | | | | | | | | | | | | | | | Mr (kDa) / pI | | | | | | | | | | | | | | | | | | | | | | | | | | | | | | | | | | | | | | | | | | | | | | | | | | | | | | | | | | | | | | | | | | | | | | | | | | | | | | | Ratio | | | | | | | | | | | | | | | | | | | | | | | | | | | | | | | | | | | | | | | | | | | | | | | | | | | | | | | | | | | | | | | | | | | | | | | | | | | | | |  |  |  |  |  |  |  |  |  |  |  |  |  |  |  |  |  |  |  |  |  |  |  |  |  |  |  |
|  |  |  |  |  |  |  |  |  |  |  |  |  |  |  |  |  |  |  |  |  |  |  |  |  |  |  |  |  |  |  |  |  |  |  |  |  |  |  |  |  |  |  |  |  |  |  |  |  |  |  |  |  |  |  |  |  |  |  |  |  |  |  |  |  |  |  |  |  |  |  |  |  |  |  |  |  |  |  |  |  |  |  |  |  |  |  |  |  |  |  |  |  |  |  |  |  |  |  |  |  |  |  |  |  |  |  |  |  |  |  |  |  |  |  |  |  |  |  |  |  |  |  |  |  |  |  |  |  |  |  |  |  |  |  |  |  |  |  |  |  |  |  |  |  |  |  |  |  |  |  |  |  |  |  |  |  |  |  |  |  |  |  |  |  |  |  |  |  |  |  |  |  |  |  |  |  |  |  |  |  |  |  |  |  |  |  |  |  |  |  |  |  |  |  |  |  |  |  |  |  |  |  |  |  |  |  |  |  |  |  |  |  |  |  |  |  |  |  |  |  |  |  |  | Theo. | | | | | | | | | | | | | | | | | | | | | | | | | | | | | | | | | | | | | | | | | | | | | | | | Exp. | | | | | | | | | | | | | | | | | | | | | | | | | | | | | | |  |  |  |  |  |  |  |  |  |  |  |  |  |  |  |  |  |  |  |  |  |  |  |  |  |  |  |  |  |  |  |  |  |  |  |  |  |  |  |  |  |  |  |  |  |  |  |  |  |  |  |  |  |  |  |  |  |  |  |  |  |  |  |  |  |  |  |  |  |  |  |  |  |  |  |  |  |  |  |  |  |  |  |  |  |  |  |  |  |  |  |  |  |  |  |  |  |  |  |  |  |  |  |  |  |
| 3h | | | | | | | | | | | Unknown | | | | | | | | | | | | | | | | | | | | | | | | | | | | | Glyma05g31030.1 | | | | | | | | | | | | | | | | | | | | | | | | | | | | | | | | | | 133 | | | | | | | | | | | | | | | | | | | | | | | | | | | | | | | 4 | | | | | | | | | | | | | | | | | | | | | | | | | | | | | | | | | | | | | | 23 | | | | | | | | | | | | | | | | | | | | | | | | | | | | | | | | | | | | | | | | 309 | | | | | | | | | | | | | | | | | | | | | | | | | | | | | | | | | | | | | | | | | 25.0/4.98 | | | | | | | | | | | | | | | | | | | | | | | | | | | | | | | | | | | | | | | | | | | | | | | |  | | | | | | | | | | | | | | | | | | | | | | | | | | | | | | |  | | | | | | | | | | | | | | | | | | | | | | | | | | | | | | | | | | | | | | | | | | | | | | | | | | | | | | | | | | | | | | | | | | | | | | | | | | | | | |  |  |  |  |  |  |  |  |  |  |  |  |  |  |  |  |  |  |  |  |  |  |  |  |  |  |  |
|  | | | | | | | | | | | Calc. Mass | | | | | | | | | | | | | | | | | | | | | | | | | | | | | Observ. Mass | | | | | | | | | | | | | | | | | | | | | | | | | | | | | | | | | | Da | | | | | | | | | | | | | | | | | | | | | | | | | | | | | | | ppm | | | | | | | | | | | | | | | | | | | | | | | | | | | | | | | | | | | | | | start | | | | | | | | | | | | | | | | | | | | | | | | | | | | | | | | | | | | | | | | end | | | | | | | | | | | | | | | | | | | | | | | | | | | | | | | | | | | | | | | | | sequence | | | | | | | | | | | | | | | | | | | | | | | | | | | | | | | | | | | | | | | | | | | | | | | | | | | | | | | | | | | | | | | | | | | | | | | | | | | | | | | | | | | | | | | | | | | | | | | | | | | | | | | | | | | | | | | | | | | | | | | | | | | | | | | | | | | | | | | | | | | | | | | | | | | | | | | | | | | | |  |  |  |  |  |  |  |  |  |  |  |  |  |  |  |  |  |  |  |  |  |  |  |  |  |  |  |
|  | | | | | | | | | | | 1388.6969 | | | | | | | | | | | | | | | | | | | | | | | | | | | | | 1388.696 | | | | | | | | | | | | | | | | | | | | | | | | | | | | | | | | | | 0 | | | | | | | | | | | | | | | | | | | | | | | | | | | | | | | -1 | | | | | | | | | | | | | | | | | | | | | | | | | | | | | | | | | | | | | | 44 | | | | | | | | | | | | | | | | | | | | | | | | | | | | | | | | | | | | | | | | 55 | | | | | | | | | | | | | | | | | | | | | | | | | | | | | | | | | | | | | | | | | LKEMEEEAAALR | | | | | | | | | | | | | | | | | | | | | | | | | | | | | | | | | | | | | | | | | | | | | | | | | | | | | | | | | | | | | | | | | | | | | | | | | | | | | | | | | | | | | | | | | | | | | | | | | | | | | | | | | | | | | | | | | | | | | | | | | | | | | | | | | | | | | | | | | | | | | | | | | | | | | | | | | | | | |  |  |  |  |  |  |  |  |  |  |  |  |  |  |  |  |  |  |  |  |  |  |  |  |  |  |  |
|  | | | | | | | | | | | 1388.6969 | | | | | | | | | | | | | | | | | | | | | | | | | | | | | 1388.697 | | | | | | | | | | | | | | | | | | | | | | | | | | | | | | | | | | 0 | | | | | | | | | | | | | | | | | | | | | | | | | | | | | | | 0 | | | | | | | | | | | | | | | | | | | | | | | | | | | | | | | | | | | | | | 44 | | | | | | | | | | | | | | | | | | | | | | | | | | | | | | | | | | | | | | | | 55 | | | | | | | | | | | | | | | | | | | | | | | | | | | | | | | | | | | | | | | | | LKEMEEEAAALR | | | | | | | | | | | | | | | | | | | | | | | | | | | | | | | | | | | | | | | | | | | | | | | | | | | | | | | | | | | | | | | | | | | | | | | | | | | | | | | | | | | | | | | | | | | | | | | | | | | | | | | | | | | | | | | | | | | | | | | | | | | | | | | | | | | | | | | | | | | | | | | | | | | | | | | | | | | | |  |  |  |  |  |  |  |  |  |  |  |  |  |  |  |  |  |  |  |  |  |  |  |  |  |  |  |
|  | | | | | | | | | | | 1404.6918 | | | | | | | | | | | | | | | | | | | | | | | | | | | | | 1404.691 | | | | | | | | | | | | | | | | | | | | | | | | | | | | | | | | | | 0 | | | | | | | | | | | | | | | | | | | | | | | | | | | | | | | 0 | | | | | | | | | | | | | | | | | | | | | | | | | | | | | | | | | | | | | | 44 | | | | | | | | | | | | | | | | | | | | | | | | | | | | | | | | | | | | | | | | 55 | | | | | | | | | | | | | | | | | | | | | | | | | | | | | | | | | | | | | | | | | LKEMEEEAAALR | | | | | | | | | | | | | | | | | | | | | | | | | | | | | | | | | | | | | | | | | | | | | | | | | | | | | | | | | | | | | | | | | | | | | | | | | | | | | | | | | | | | | | | | | | | | | | | | | | | | | | | | | | | | | | | | | | | | | | | | | | | | | | | | | | | | | | | | | | | | | | | | | | | | | | | | | | | | |  |  |  |  |  |  |  |  |  |  |  |  |  |  |  |  |  |  |  |  |  |  |  |  |  |  |  |
|  | | | | | | | | | | | 2128.0396 | | | | | | | | | | | | | | | | | | | | | | | | | | | | | 2128.039 | | | | | | | | | | | | | | | | | | | | | | | | | | | | | | | | | | 0 | | | | | | | | | | | | | | | | | | | | | | | | | | | | | | | 0 | | | | | | | | | | | | | | | | | | | | | | | | | | | | | | | | | | | | | | 61 | | | | | | | | | | | | | | | | | | | | | | | | | | | | | | | | | | | | | | | | 81 | | | | | | | | | | | | | | | | | | | | | | | | | | | | | | | | | | | | | | | | | VDKEIGSVQDPANSAASQANK | | | | | | | | | | | | | | | | | | | | | | | | | | | | | | | | | | | | | | | | | | | | | | | | | | | | | | | | | | | | | | | | | | | | | | | | | | | | | | | | | | | | | | | | | | | | | | | | | | | | | | | | | | | | | | | | | | | | | | | | | | | | | | | | | | | | | | | | | | | | | | | | | | | | | | | | | | | | |  |  |  |  |  |  |  |  |  |  |  |  |  |  |  |  |  |  |  |  |  |  |  |  |  |  |  |
|  | | | | | | | | | | | 2473.1317 | | | | | | | | | | | | | | | | | | | | | | | | | | | | | 2473.13 | | | | | | | | | | | | | | | | | | | | | | | | | | | | | | | | | | 0 | | | | | | | | | | | | | | | | | | | | | | | | | | | | | | | -1 | | | | | | | | | | | | | | | | | | | | | | | | | | | | | | | | | | | | | | 64 | | | | | | | | | | | | | | | | | | | | | | | | | | | | | | | | | | | | | | | | 87 | | | | | | | | | | | | | | | | | | | | | | | | | | | | | | | | | | | | | | | | | EIGSVQDPANSAASQANKEEADSR | | | | | | | | | | | | | | | | | | | | | | | | | | | | | | | | | | | | | | | | | | | | | | | | | | | | | | | | | | | | | | | | | | | | | | | | | | | | | | | | | | | | | | | | | | | | | | | | | | | | | | | | | | | | | | | | | | | | | | | | | | | | | | | | | | | | | | | | | | | | | | | | | | | | | | | | | | | | |  |  |  |  |  |  |  |  |  |  |  |  |  |  |  |  |  |  |  |  |  |  |  |  |  |  |  |
|  | | | | | | | | | | | 1345.7606 | | | | | | | | | | | | | | | | | | | | | | | | | | | | | 1345.76 | | | | | | | | | | | | | | | | | | | | | | | | | | | | | | | | | | 0 | | | | | | | | | | | | | | | | | | | | | | | | | | | | | | | 0 | | | | | | | | | | | | | | | | | | | | | | | | | | | | | | | | | | | | | | 116 | | | | | | | | | | | | | | | | | | | | | | | | | | | | | | | | | | | | | | | | 127 | | | | | | | | | | | | | | | | | | | | | | | | | | | | | | | | | | | | | | | | | VTILTDKFGQPK | | | | | | | | | | | | | | | | | | | | | | | | | | | | | | | | | | | | | | | | | | | | | | | | | | | | | | | | | | | | | | | | | | | | | | | | | | | | | | | | | | | | | | | | | | | | | | | | | | | | | | | | | | | | | | | | | | | | | | | | | | | | | | | | | | | | | | | | | | | | | | | | | | | | | | | | | | | | |  |  |  |  |  |  |  |  |  |  |  |  |  |  |  |  |  |  |  |  |  |  |  |  |  |  |  |
| Spot no. | | | | | | | | | | | Homologous protein | | | | | | | | | | | | | | | | | | | | | | | | | | | | | Accession no. | | | | | | | | | | | | | | | | | | | | | | | | | | | | | | | | | | Score | | | | | | | | | | | | | | | | | | | | | | | | | | | | | | | M.P. | | | | | | | | | | | | | | | | | | | | | | | | | | | | | | | | | | | | | | Cov. (%) | | | | | | | | | | | | | | | | | | | | | | | | | | | | | | | | | | | | | | | | Blast score | | | | | | | | | | | | | | | | | | | | | | | | | | | | | | | | | | | | | | | | | Mr (kDa) / pI | | | | | | | | | | | | | | | | | | | | | | | | | | | | | | | | | | | | | | | | | | | | | | | | | | | | | | | | | | | | | | | | | | | | | | | | | | | | | | | Ratio | | | | | | | | | | | | | | | | | | | | | | | | | | | | | | | | | | | | | | | | | | | | | | | | | | | | | | | | | | | | | | | | | | | | | | | | | | | | | |  |  |  |  |  |  |  |  |  |  |  |  |  |  |  |  |  |  |  |  |  |  |  |  |  |  |  |
|  |  |  |  |  |  |  |  |  |  |  |  |  |  |  |  |  |  |  |  |  |  |  |  |  |  |  |  |  |  |  |  |  |  |  |  |  |  |  |  |  |  |  |  |  |  |  |  |  |  |  |  |  |  |  |  |  |  |  |  |  |  |  |  |  |  |  |  |  |  |  |  |  |  |  |  |  |  |  |  |  |  |  |  |  |  |  |  |  |  |  |  |  |  |  |  |  |  |  |  |  |  |  |  |  |  |  |  |  |  |  |  |  |  |  |  |  |  |  |  |  |  |  |  |  |  |  |  |  |  |  |  |  |  |  |  |  |  |  |  |  |  |  |  |  |  |  |  |  |  |  |  |  |  |  |  |  |  |  |  |  |  |  |  |  |  |  |  |  |  |  |  |  |  |  |  |  |  |  |  |  |  |  |  |  |  |  |  |  |  |  |  |  |  |  |  |  |  |  |  |  |  |  |  |  |  |  |  |  |  |  |  |  |  |  |  |  |  |  |  |  |  |  |  | Theo. | | | | | | | | | | | | | | | | | | | | | | | | | | | | | | | | | | | | | | | | | | | | | | | | | | | | | | | | Exp. | | | | | | | | | | | | | | | | | | | | | | |  |  |  |  |  |  |  |  |  |  |  |  |  |  |  |  |  |  |  |  |  |  |  |  |  |  |  |  |  |  |  |  |  |  |  |  |  |  |  |  |  |  |  |  |  |  |  |  |  |  |  |  |  |  |  |  |  |  |  |  |  |  |  |  |  |  |  |  |  |  |  |  |  |  |  |  |  |  |  |  |  |  |  |  |  |  |  |  |  |  |  |  |  |  |  |  |  |  |  |  |  |  |  |  |  |
| 3i | | | | | | | | | | | uncharacterized protein LOC100786604 | | | | | | | | | | | | | | | | | | | | | | | | | | | | | Glyma07g38630.1 | | | | | | | | | | | | | | | | | | | | | | | | | | | | | | | | | | 133 | | | | | | | | | | | | | | | | | | | | | | | | | | | | | | | 3 | | | | | | | | | | | | | | | | | | | | | | | | | | | | | | | | | | | | | | 9 | | | | | | | | | | | | | | | | | | | | | | | | | | | | | | | | | | | | | | | | 579 | | | | | | | | | | | | | | | | | | | | | | | | | | | | | | | | | | | | | | | | | 37.7/5.2 | | | | | | | | | | | | | | | | | | | | | | | | | | | | | | | | | | | | | | | | | | | | | | | | | | | | | | | |  | | | | | | | | | | | | | | | | | | | | | | |  | | | | | | | | | | | | | | | | | | | | | | | | | | | | | | | | | | | | | | | | | | | | | | | | | | | | | | | | | | | | | | | | | | | | | | | | | | | | | |  |  |  |  |  |  |  |  |  |  |  |  |  |  |  |  |  |  |  |  |  |  |  |  |  |  |  |
|  | | | | | | | | | | | Calc. Mass | | | | | | | | | | | | | | | | | | | | | | | | | | | | | Observ. Mass | | | | | | | | | | | | | | | | | | | | | | | | | | | | | | | | | | Da | | | | | | | | | | | | | | | | | | | | | | | | | | | | | | | ppm | | | | | | | | | | | | | | | | | | | | | | | | | | | | | | | | | | | | | | start | | | | | | | | | | | | | | | | | | | | | | | | | | | | | | | | | | | | | | | | end | | | | | | | | | | | | | | | | | | | | | | | | | | | | | | | | | | | | | | | | | sequence | | | | | | | | | | | | | | | | | | | | | | | | | | | | | | | | | | | | | | | | | | | | | | | | | | | | | | | | | | | | | | | | | | | | | | | | | | | | | | | | | | | | | | | | | | | | | | | | | | | | | | | | | | | | | | | | | | | | | | | | | | | | | | | | | | | | | | | | | | | | | | | | | | | | | | | | | | | | |  |  |  |  |  |  |  |  |  |  |  |  |  |  |  |  |  |  |  |  |  |  |  |  |  |  |  |
|  | | | | | | | | | | | 1870.9207 | | | | | | | | | | | | | | | | | | | | | | | | | | | | | 1870.919 | | | | | | | | | | | | | | | | | | | | | | | | | | | | | | | | | | 0 | | | | | | | | | | | | | | | | | | | | | | | | | | | | | | | -1 | | | | | | | | | | | | | | | | | | | | | | | | | | | | | | | | | | | | | | 34 | | | | | | | | | | | | | | | | | | | | | | | | | | | | | | | | | | | | | | | | 52 | | | | | | | | | | | | | | | | | | | | | | | | | | | | | | | | | | | | | | | | | MSNNSAVAPSADTVGPVVR | | | | | | | | | | | | | | | | | | | | | | | | | | | | | | | | | | | | | | | | | | | | | | | | | | | | | | | | | | | | | | | | | | | | | | | | | | | | | | | | | | | | | | | | | | | | | | | | | | | | | | | | | | | | | | | | | | | | | | | | | | | | | | | | | | | | | | | | | | | | | | | | | | | | | | | | | | | | |  |  |  |  |  |  |  |  |  |  |  |  |  |  |  |  |  |  |  |  |  |  |  |  |  |  |  |
|  | | | | | | | | | | | 1870.9207 | | | | | | | | | | | | | | | | | | | | | | | | | | | | | 1870.919 | | | | | | | | | | | | | | | | | | | | | | | | | | | | | | | | | | 0 | | | | | | | | | | | | | | | | | | | | | | | | | | | | | | | -1 | | | | | | | | | | | | | | | | | | | | | | | | | | | | | | | | | | | | | | 34 | | | | | | | | | | | | | | | | | | | | | | | | | | | | | | | | | | | | | | | | 52 | | | | | | | | | | | | | | | | | | | | | | | | | | | | | | | | | | | | | | | | | MSNNSAVAPSADTVGPVVR | | | | | | | | | | | | | | | | | | | | | | | | | | | | | | | | | | | | | | | | | | | | | | | | | | | | | | | | | | | | | | | | | | | | | | | | | | | | | | | | | | | | | | | | | | | | | | | | | | | | | | | | | | | | | | | | | | | | | | | | | | | | | | | | | | | | | | | | | | | | | | | | | | | | | | | | | | | | |  |  |  |  |  |  |  |  |  |  |  |  |  |  |  |  |  |  |  |  |  |  |  |  |  |  |  |
|  | | | | | | | | | | | 1886.9156 | | | | | | | | | | | | | | | | | | | | | | | | | | | | | 1886.913 | | | | | | | | | | | | | | | | | | | | | | | | | | | | | | | | | | 0 | | | | | | | | | | | | | | | | | | | | | | | | | | | | | | | -1 | | | | | | | | | | | | | | | | | | | | | | | | | | | | | | | | | | | | | | 34 | | | | | | | | | | | | | | | | | | | | | | | | | | | | | | | | | | | | | | | | 52 | | | | | | | | | | | | | | | | | | | | | | | | | | | | | | | | | | | | | | | | | MSNNSAVAPSADTVGPVVR | | | | | | | | | | | | | | | | | | | | | | | | | | | | | | | | | | | | | | | | | | | | | | | | | | | | | | | | | | | | | | | | | | | | | | | | | | | | | | | | | | | | | | | | | | | | | | | | | | | | | | | | | | | | | | | | | | | | | | | | | | | | | | | | | | | | | | | | | | | | | | | | | | | | | | | | | | | | |  |  |  |  |  |  |  |  |  |  |  |  |  |  |  |  |  |  |  |  |  |  |  |  |  |  |  |
|  | | | | | | | | | | | 2015.0106 | | | | | | | | | | | | | | | | | | | | | | | | | | | | | 2015.009 | | | | | | | | | | | | | | | | | | | | | | | | | | | | | | | | | | 0 | | | | | | | | | | | | | | | | | | | | | | | | | | | | | | | -1 | | | | | | | | | | | | | | | | | | | | | | | | | | | | | | | | | | | | | | 34 | | | | | | | | | | | | | | | | | | | | | | | | | | | | | | | | | | | | | | | | 53 | | | | | | | | | | | | | | | | | | | | | | | | | | | | | | | | | | | | | | | | | MSNNSAVAPSADTVGPVVRK | | | | | | | | | | | | | | | | | | | | | | | | | | | | | | | | | | | | | | | | | | | | | | | | | | | | | | | | | | | | | | | | | | | | | | | | | | | | | | | | | | | | | | | | | | | | | | | | | | | | | | | | | | | | | | | | | | | | | | | | | | | | | | | | | | | | | | | | | | | | | | | | | | | | | | | | | | | | |  |  |  |  |  |  |  |  |  |  |  |  |  |  |  |  |  |  |  |  |  |  |  |  |  |  |  |
|  | | | | | | | | | | | 1232.6554 | | | | | | | | | | | | | | | | | | | | | | | | | | | | | 1232.656 | | | | | | | | | | | | | | | | | | | | | | | | | | | | | | | | | | 0 | | | | | | | | | | | | | | | | | | | | | | | | | | | | | | | 0 | | | | | | | | | | | | | | | | | | | | | | | | | | | | | | | | | | | | | | 63 | | | | | | | | | | | | | | | | | | | | | | | | | | | | | | | | | | | | | | | | 73 | | | | | | | | | | | | | | | | | | | | | | | | | | | | | | | | | | | | | | | | | YGPNPIQVAFKBottom of Form | | | | | | | | | | | | | | | | | | | | | | | | | | | | | | | | | | | | | | | | | | | | | | | | | | | | | | | | | | | | | | | | | | | | | | | | | | | | | | | | | | | | | | | | | | | | | | | | | | | | | | | | | | | | | | | | | | | | | | | | | | | | | | | | | | | | | | | | | | | | | | | | | | | | | | | | | | | | |  |  |  |  |  |  |  |  |  |  |  |  |  |  |  |  |  |  |  |  |  |  |  |  |  |  |  |
| Spot no. | | | | Homologous protein | | | | | | | | | | | | | | | Accession no. | | | | | | | | | | | | | | | | | | | | | | | | | | | | | | Score | | | | | | | | | | | | | | | | | | | | | | | | | | | | | | | | | M.P. | | | | | | | | | | | | | | | | | | | | | | | | | | | | | | | | | | | Cov. (%) | | | | | | | | | | | | | | | | | | | | | | | | | | | | | | | | | | | | | | | | | | Blast score | | | | | | | | | | | | | | | | | | | | | | | | | | | | | | | | | | | | | | | | | | | | | Mr (kDa) / pI | | | | | | | | | | | | | | | | | | | | | | | | | | | | | | | | | | | | | | | | | | | | | | | | | | | | | | | | | | | | | | | | | | | | | | | | | | | | | | | | | | | | | | | | | | | | | | | | | | | | | | | | | | | | | | | | | | | | | | | | | | | | Ratio | | | | | | | | | | | | | | | | | | | | | | | | | | | | | | | | | | | | | | | | | | | | | | | | | |  |  |  |  |  |  |  |  |  |  |  |  |  |  |  |  |  |  |  |  |  |  |  |  |  |  |  |  |  |  |
|  |  |  |  |  |  |  |  |  |  |  |  |  |  |  |  |  |  |  |  |  |  |  |  |  |  |  |  |  |  |  |  |  |  |  |  |  |  |  |  |  |  |  |  |  |  |  |  |  |  |  |  |  |  |  |  |  |  |  |  |  |  |  |  |  |  |  |  |  |  |  |  |  |  |  |  |  |  |  |  |  |  |  |  |  |  |  |  |  |  |  |  |  |  |  |  |  |  |  |  |  |  |  |  |  |  |  |  |  |  |  |  |  |  |  |  |  |  |  |  |  |  |  |  |  |  |  |  |  |  |  |  |  |  |  |  |  |  |  |  |  |  |  |  |  |  |  |  |  |  |  |  |  |  |  |  |  |  |  |  |  |  |  |  |  |  |  |  |  |  |  |  |  |  |  |  |  |  |  |  |  |  |  |  |  |  |  |  |  |  |  |  |  |  |  |  |  |  |  |  |  |  |  |  | Theo. | | | | | | | | | | | | | | | | | | | | | | | | | | | | | | | | | | | | | | | | | | | | | | | | | | | | | | Exp. | | | | | | | | | | | | | | | | | | | | | | | | | | | | | | | | | | | | | | | | | | | | | | | | | | | | | | | | | | | | | | | | | | | | | |  |  |  |  |  |  |  |  |  |  |  |  |  |  |  |  |  |  |  |  |  |  |  |  |  |  |  |  |  |  |  |  |  |  |  |  |  |  |  |  |  |  |  |  |  |  |  |  |  |  |  |  |  |  |  |  |  |  |  |  |  |  |  |  |  |  |  |  |  |  |  |  |  |  |  |  |  |  |  |  |
| 3j | | | | Phosphoglycerate kinase | | | | | | | | | | | | | | | Glyma08g17600.1 | | | | | | | | | | | | | | | | | | | | | | | | | | | | | | 125 | | | | | | | | | | | | | | | | | | | | | | | | | | | | | | | | | 3 | | | | | | | | | | | | | | | | | | | | | | | | | | | | | | | | | | | 11 | | | | | | | | | | | | | | | | | | | | | | | | | | | | | | | | | | | | | | | | | | 735 | | | | | | | | | | | | | | | | | | | | | | | | | | | | | | | | | | | | | | | | | | | | | 42.6/6.28 | | | | | | | | | | | | | | | | | | | | | | | | | | | | | | | | | | | | | | | | | | | | | | | | | | | | | |  | | | | | | | | | | | | | | | | | | | | | | | | | | | | | | | | | | | | | | | | | | | | | | | | | | | | | | | | | | | | | | | | | | | | | |  | | | | | | | | | | | | | | | | | | | | | | | | | | | | | | | | | | | | | | | | | | | | | | | | | |  |  |  |  |  |  |  |  |  |  |  |  |  |  |  |  |  |  |  |  |  |  |  |  |  |  |  |  |  |  |
|  | | | | Calc. Mass | | | | | | | | | | | | | | | Observ. Mass | | | | | | | | | | | | | | | | | | | | | | | | | | | | | | Da | | | | | | | | | | | | | | | | | | | | | | | | | | | | | | | | | ppm | | | | | | | | | | | | | | | | | | | | | | | | | | | | | | | | | | | start | | | | | | | | | | | | | | | | | | | | | | | | | | | | | | | | | | | | | | | | | | end | | | | | | | | | | | | | | | | | | | | | | | | | | | | | | | | | | | | | | | | | | | | | sequence | | | | | | | | | | | | | | | | | | | | | | | | | | | | | | | | | | | | | | | | | | | | | | | | | | | | | | | | | | | | | | | | | | | | | | | | | | | | | | | | | | | | | | | | | | | | | | | | | | | | | | | | | | | | | | | | | | | | | | | | | | | | | | | | | | | | | | | | | | | | | | | | | | | | | | | | | | | | | | | | | | | | | | | | | | | | | |  |  |  |  |  |  |  |  |  |  |  |  |  |  |  |  |  |  |  |  |  |  |  |  |  |  |  |  |  |  |
|  | | | | 1403.7296 | | | | | | | | | | | | | | | 1403.728 | | | | | | | | | | | | | | | | | | | | | | | | | | | | | | 0 | | | | | | | | | | | | | | | | | | | | | | | | | | | | | | | | | -1 | | | | | | | | | | | | | | | | | | | | | | | | | | | | | | | | | | | 179 | | | | | | | | | | | | | | | | | | | | | | | | | | | | | | | | | | | | | | | | | | 191 | | | | | | | | | | | | | | | | | | | | | | | | | | | | | | | | | | | | | | | | | | | | | ELDYLVGAVSNPK | | | | | | | | | | | | | | | | | | | | | | | | | | | | | | | | | | | | | | | | | | | | | | | | | | | | | | | | | | | | | | | | | | | | | | | | | | | | | | | | | | | | | | | | | | | | | | | | | | | | | | | | | | | | | | | | | | | | | | | | | | | | | | | | | | | | | | | | | | | | | | | | | | | | | | | | | | | | | | | | | | | | | | | | | | | | | |  |  |  |  |  |  |  |  |  |  |  |  |  |  |  |  |  |  |  |  |  |  |  |  |  |  |  |  |  |  |
|  | | | | 1538.892 | | | | | | | | | | | | | | | 1538.89 | | | | | | | | | | | | | | | | | | | | | | | | | | | | | | 0 | | | | | | | | | | | | | | | | | | | | | | | | | | | | | | | | | -1 | | | | | | | | | | | | | | | | | | | | | | | | | | | | | | | | | | | 262 | | | | | | | | | | | | | | | | | | | | | | | | | | | | | | | | | | | | | | | | | | 276 | | | | | | | | | | | | | | | | | | | | | | | | | | | | | | | | | | | | | | | | | | | | | GVSLLLPTDVVIADK | | | | | | | | | | | | | | | | | | | | | | | | | | | | | | | | | | | | | | | | | | | | | | | | | | | | | | | | | | | | | | | | | | | | | | | | | | | | | | | | | | | | | | | | | | | | | | | | | | | | | | | | | | | | | | | | | | | | | | | | | | | | | | | | | | | | | | | | | | | | | | | | | | | | | | | | | | | | | | | | | | | | | | | | | | | | | |  |  |  |  |  |  |  |  |  |  |  |  |  |  |  |  |  |  |  |  |  |  |  |  |  |  |  |  |  |  |
|  | | | | 1572.8359 | | | | | | | | | | | | | | | 1572.835 | | | | | | | | | | | | | | | | | | | | | | | | | | | | | | 0 | | | | | | | | | | | | | | | | | | | | | | | | | | | | | | | | | 0 | | | | | | | | | | | | | | | | | | | | | | | | | | | | | | | | | | | 350 | | | | | | | | | | | | | | | | | | | | | | | | | | | | | | | | | | | | | | | | | | 366 | | | | | | | | | | | | | | | | | | | | | | | | | | | | | | | | | | | | | | | | | | | | | GVTTIIGGGDSVAAVEK | | | | | | | | | | | | | | | | | | | | | | | | | | | | | | | | | | | | | | | | | | | | | | | | | | | | | | | | | | | | | | | | | | | | | | | | | | | | | | | | | | | | | | | | | | | | | | | | | | | | | | | | | | | | | | | | | | | | | | | | | | | | | | | | | | | | | | | | | | | | | | | | | | | | | | | | | | | | | | | | | | | | | | | | | | | | | |  |  |  |  |  |  |  |  |  |  |  |  |  |  |  |  |  |  |  |  |  |  |  |  |  |  |  |  |  |  |
| Spot no. | Homologous protein | | | | | | | | | | | | | | Accession no. | | | | | | | | | | | | | | | | | | | | | | | | | | | | Score | | | | | | | | | | | | | | | | | | | | | | | | | | | | | | | | | | M.P. | | | | | | | | | | | | | | | | | | | | | | | | | | | | | | | | | | | | | | | | | | | | | | | | | | | | | | | | | | | | | | | | | | | | | | | | | | Cov. (%) | | | | | | | | | | | | | | | | | | | | | | | | | | | | | | | | | | | | | | | | | | | Blast score | | | | | | | | | | | | | | | | | | | | | | | | | | | | | | | | | | | | | | | | | | | Mr (kDa) / pI | | | | | | | | | | | | | | | | | | | | | | | | | | | | | | | | | | | | | | | | | | | | | | | | | | | | | | | | | | | | | | | | | | | | | | | | | | | | | | | | | | | | | | | | | | | | | | | | | | | | | | | | | | | | | | | | | | | | | | | | | | | | | | | | | | | | Ratio | | | | | | | | | | | | | | | | | | | | | | |  |  |  |  |  |  |  |  |  |  |  |  |  |  |  |  |
|  |  |  |  |  |  |  |  |  |  |  |  |  |  |  |  |  |  |  |  |  |  |  |  |  |  |  |  |  |  |  |  |  |  |  |  |  |  |  |  |  |  |  |  |  |  |  |  |  |  |  |  |  |  |  |  |  |  |  |  |  |  |  |  |  |  |  |  |  |  |  |  |  |  |  |  |  |  |  |  |  |  |  |  |  |  |  |  |  |  |  |  |  |  |  |  |  |  |  |  |  |  |  |  |  |  |  |  |  |  |  |  |  |  |  |  |  |  |  |  |  |  |  |  |  |  |  |  |  |  |  |  |  |  |  |  |  |  |  |  |  |  |  |  |  |  |  |  |  |  |  |  |  |  |  |  |  |  |  |  |  |  |  |  |  |  |  |  |  |  |  |  |  |  |  |  |  |  |  |  |  |  |  |  |  |  |  |  |  |  |  |  |  |  |  |  |  |  |  |  |  |  |  |  |  |  |  |  |  |  |  |  |  |  |  |  |  |  |  |  |  |  |  |  |  |  |  |  |  |  |  |  |  |  |  |  |  | Theo. | | | | | | | | | | | | | | | | | | | | | | | | | | | | | | | | | | | | | | | | | | | | | | | | | | | | | | | | | | | | | Exp. | | | | | | | | | | | | | | | | | | | | | | | | | | | | | | | | | | | | | | | | | | | | | | | | | | | | | | | | | | | | | | | | | | | | | | |  |  |  |  |  |  |  |  |  |  |  |  |  |  |  |  |  |  |  |  |  |  |  |  |  |  |  |  |  |  |  |  |  |  |  |  |  |  |  |
| 3k | uncharacterized protein LOC100807396 | | | | | | | | | | | | | | Glyma04g06250.1 | | | | | | | | | | | | | | | | | | | | | | | | | | | | 113 | | | | | | | | | | | | | | | | | | | | | | | | | | | | | | | | | | 3 | | | | | | | | | | | | | | | | | | | | | | | | | | | | | | | | | | | | | | | | | | | | | | | | | | | | | | | | | | | | | | | | | | | | | | | | | | 12 | | | | | | | | | | | | | | | | | | | | | | | | | | | | | | | | | | | | | | | | | | | 579 | | | | | | | | | | | | | | | | | | | | | | | | | | | | | | | | | | | | | | | | | | | 34.0/5.02 | | | | | | | | | | | | | | | | | | | | | | | | | | | | | | | | | | | | | | | | | | | | | | | | | | | | | | | | | | | | |  | | | | | | | | | | | | | | | | | | | | | | | | | | | | | | | | | | | | | | | | | | | | | | | | | | | | | | | | | | | | | | | | | | | | | | |  | | | | | | | | | | | | | | | | | | | | | | |  |  |  |  |  |  |  |  |  |  |  |  |  |  |  |  |
|  | Calc. Mass | | | | | | | | | | | | | | Observ. Mass | | | | | | | | | | | | | | | | | | | | | | | | | | | | Da | | | | | | | | | | | | | | | | | | | | | | | | | | | | | | | | | | ppm | | | | | | | | | | | | | | | | | | | | | | | | | | | | | | | | | | | | | | | | | | | | | | | | | | | | | | | | | | | | | | | | | | | | | | | | | | start | | | | | | | | | | | | | | | | | | | | | | | | | | | | | | | | | | | | | | | | | end | | | | | | | | | | | | | | | | | | | | | | | | | | | | | | | | | | | | | | | | | | sequence | | | | | | | | | | | | | | | | | | | | | | | | | | | | | | | | | | | | | | | | | | | | | | | | | | | | | | | | | | | | | | | | | | | | | | | | | | | | | | | | | | | | | | | | | | | | | | | | | | | | | | | | | | | | | | | | | | | | | | | | | | | | | | | | | | | | | | | | | | | | | | | | | | | | | | | | | | | | | |  |  |  |  |  |  |  |  |  |  |  |  |  |  |  |  |
|  | 1432.7158 | | | | | | | | | | | | | | 1432.711 | | | | | | | | | | | | | | | | | | | | | | | | | | | | 0 | | | | | | | | | | | | | | | | | | | | | | | | | | | | | | | | | | -3 | | | | | | | | | | | | | | | | | | | | | | | | | | | | | | | | | | | | | | | | | | | | | | | | | | | | | | | | | | | | | | | | | | | | | | | | | | 124 | | | | | | | | | | | | | | | | | | | | | | | | | | | | | | | | | | | | | | | | | 138 | | | | | | | | | | | | | | | | | | | | | | | | | | | | | | | | | | | | | | | | | | DAGSTASTAILVGDR | | | | | | | | | | | | | | | | | | | | | | | | | | | | | | | | | | | | | | | | | | | | | | | | | | | | | | | | | | | | | | | | | | | | | | | | | | | | | | | | | | | | | | | | | | | | | | | | | | | | | | | | | | | | | | | | | | | | | | | | | | | | | | | | | | | | | | | | | | | | | | | | | | | | | | | | | | | | | |  |  |  |  |  |  |  |  |  |  |  |  |  |  |  |  |
|  | 1432.7158 | | | | | | | | | | | | | | 1432.716 | | | | | | | | | | | | | | | | | | | | | | | | | | | | 0 | | | | | | | | | | | | | | | | | | | | | | | | | | | | | | | | | | 0 | | | | | | | | | | | | | | | | | | | | | | | | | | | | | | | | | | | | | | | | | | | | | | | | | | | | | | | | | | | | | | | | | | | | | | | | | | 124 | | | | | | | | | | | | | | | | | | | | | | | | | | | | | | | | | | | | | | | | | 138 | | | | | | | | | | | | | | | | | | | | | | | | | | | | | | | | | | | | | | | | | | DAGSTASTAILVGDR | | | | | | | | | | | | | | | | | | | | | | | | | | | | | | | | | | | | | | | | | | | | | | | | | | | | | | | | | | | | | | | | | | | | | | | | | | | | | | | | | | | | | | | | | | | | | | | | | | | | | | | | | | | | | | | | | | | | | | | | | | | | | | | | | | | | | | | | | | | | | | | | | | | | | | | | | | | | | |  |  |  |  |  |  |  |  |  |  |  |  |  |  |  |  |
|  | 1546.7515 | | | | | | | | | | | | | | 1546.75 | | | | | | | | | | | | | | | | | | | | | | | | | | | | 0 | | | | | | | | | | | | | | | | | | | | | | | | | | | | | | | | | | -1 | | | | | | | | | | | | | | | | | | | | | | | | | | | | | | | | | | | | | | | | | | | | | | | | | | | | | | | | | | | | | | | | | | | | | | | | | | 207 | | | | | | | | | | | | | | | | | | | | | | | | | | | | | | | | | | | | | | | | | 219 | | | | | | | | | | | | | | | | | | | | | | | | | | | | | | | | | | | | | | | | | | QYVVADPEIQEEK | | | | | | | | | | | | | | | | | | | | | | | | | | | | | | | | | | | | | | | | | | | | | | | | | | | | | | | | | | | | | | | | | | | | | | | | | | | | | | | | | | | | | | | | | | | | | | | | | | | | | | | | | | | | | | | | | | | | | | | | | | | | | | | | | | | | | | | | | | | | | | | | | | | | | | | | | | | | | |  |  |  |  |  |  |  |  |  |  |  |  |  |  |  |  |
|  | 1289.6398 | | | | | | | | | | | | | | 1289.64 | | | | | | | | | | | | | | | | | | | | | | | | | | | | 0 | | | | | | | | | | | | | | | | | | | | | | | | | | | | | | | | | | 0 | | | | | | | | | | | | | | | | | | | | | | | | | | | | | | | | | | | | | | | | | | | | | | | | | | | | | | | | | | | | | | | | | | | | | | | | | | 268 | | | | | | | | | | | | | | | | | | | | | | | | | | | | | | | | | | | | | | | | | 279 | | | | | | | | | | | | | | | | | | | | | | | | | | | | | | | | | | | | | | | | | | GSADNITCVVVR | | | | | | | | | | | | | | | | | | | | | | | | | | | | | | | | | | | | | | | | | | | | | | | | | | | | | | | | | | | | | | | | | | | | | | | | | | | | | | | | | | | | | | | | | | | | | | | | | | | | | | | | | | | | | | | | | | | | | | | | | | | | | | | | | | | | | | | | | | | | | | | | | | | | | | | | | | | | | |  |  |  |  |  |  |  |  |  |  |  |  |  |  |  |  |
| Spot no. | | | | Homologous protein | | | | | | | | | | | | | | | Accession no. | | | | | | | | | | | | | | | | | | | | | | | | | | | | | | Score | | | | | | | | | | | | | | | | | | | | | | | | | | | | | | | | | M.P. | | | | | | | | | | | | | | | | | | | | | | | | | | | | | | | | | | | Cov. (%) | | | | | | | | | | | | | | | | | | | | | | | | | | | | | | | | | | | | | | | | | | Blast score | | | | | | | | | | | | | | | | | | | | | | | | | | | | | | | | | | | | | | | | | | | | | Mr (kDa) / pI | | | | | | | | | | | | | | | | | | | | | | | | | | | | | | | | | | | | | | | | | | | | | | | | | | | | | | | | | | | | | | | | | | | | | | | | | | | | | | | | | | | | | | | | | | | | | | | | | | | | | | | | | | | | | | | | | | | | | | | | | | | | | | | | | | | | | | | | | | | | | | | | | | | | | | | | | Ratio | | | | | | | | | | | | | | | | | | | | |  |  |  |  |  |  |  |  |  |  |  |  |  |  |  |  |  |  |  |  |  |  |  |  |  |  |  |  |  |  |
|  |  |  |  |  |  |  |  |  |  |  |  |  |  |  |  |  |  |  |  |  |  |  |  |  |  |  |  |  |  |  |  |  |  |  |  |  |  |  |  |  |  |  |  |  |  |  |  |  |  |  |  |  |  |  |  |  |  |  |  |  |  |  |  |  |  |  |  |  |  |  |  |  |  |  |  |  |  |  |  |  |  |  |  |  |  |  |  |  |  |  |  |  |  |  |  |  |  |  |  |  |  |  |  |  |  |  |  |  |  |  |  |  |  |  |  |  |  |  |  |  |  |  |  |  |  |  |  |  |  |  |  |  |  |  |  |  |  |  |  |  |  |  |  |  |  |  |  |  |  |  |  |  |  |  |  |  |  |  |  |  |  |  |  |  |  |  |  |  |  |  |  |  |  |  |  |  |  |  |  |  |  |  |  |  |  |  |  |  |  |  |  |  |  |  |  |  |  |  |  |  |  |  |  | Theo. | | | | | | | | | | | | | | | | | | | | | | | | | | | | | | | | | | | | | | | | | | | | | | | | | | | | | | | | | | | | | | | | | | Exp. | | | | | | | | | | | | | | | | | | | | | | | | | | | | | | | | | | | | | | | | | | | | | | | | | | | | | | | | | | | | | | | | | | | | | | | | | | | | | | | | | | | | | | |  |  |  |  |  |  |  |  |  |  |  |  |  |  |  |  |  |  |  |  |  |  |  |  |  |  |  |  |  |  |  |  |  |  |  |  |  |  |  |  |  |  |  |  |  |  |  |  |  |  |  |
| 3l | | | | Photosystem II stability/assembly factor HCF136 | | | | | | | | | | | | | | | Glyma11g10710.1 | | | | | | | | | | | | | | | | | | | | | | | | | | | | | | 106 | | | | | | | | | | | | | | | | | | | | | | | | | | | | | | | | | 2 | | | | | | | | | | | | | | | | | | | | | | | | | | | | | | | | | | | 7 | | | | | | | | | | | | | | | | | | | | | | | | | | | | | | | | | | | | | | | | | | 578 | | | | | | | | | | | | | | | | | | | | | | | | | | | | | | | | | | | | | | | | | | | | | 42.4/5.74 | | | | | | | | | | | | | | | | | | | | | | | | | | | | | | | | | | | | | | | | | | | | | | | | | | | | | | | | | | | | | | | | | |  | | | | | | | | | | | | | | | | | | | | | | | | | | | | | | | | | | | | | | | | | | | | | | | | | | | | | | | | | | | | | | | | | | | | | | | | | | | | | | | | | | | | | | |  | | | | | | | | | | | | | | | | | | | | |  |  |  |  |  |  |  |  |  |  |  |  |  |  |  |  |  |  |  |  |  |  |  |  |  |  |  |  |  |  |
|  | | | | Calc. Mass | | | | | | | | | | | | | | | Observ. Mass | | | | | | | | | | | | | | | | | | | | | | | | | | | | | | Da | | | | | | | | | | | | | | | | | | | | | | | | | | | | | | | | | ppm | | | | | | | | | | | | | | | | | | | | | | | | | | | | | | | | | | | start | | | | | | | | | | | | | | | | | | | | | | | | | | | | | | | | | | | | | | | | | | end | | | | | | | | | | | | | | | | | | | | | | | | | | | | | | | | | | | | | | | | | | | | | sequence | | | | | | | | | | | | | | | | | | | | | | | | | | | | | | | | | | | | | | | | | | | | | | | | | | | | | | | | | | | | | | | | | | | | | | | | | | | | | | | | | | | | | | | | | | | | | | | | | | | | | | | | | | | | | | | | | | | | | | | | | | | | | | | | | | | | | | | | | | | | | | | | | | | | | | | | | | | | | | | | | | | | | | | | | | | | | |  |  |  |  |  |  |  |  |  |  |  |  |  |  |  |  |  |  |  |  |  |  |  |  |  |  |  |  |  |  |
|  | | | | 1541.6634 | | | | | | | | | | | | | | | 1541.662 | | | | | | | | | | | | | | | | | | | | | | | | | | | | | | 0 | | | | | | | | | | | | | | | | | | | | | | | | | | | | | | | | | -1 | | | | | | | | | | | | | | | | | | | | | | | | | | | | | | | | | | | 124 | | | | | | | | | | | | | | | | | | | | | | | | | | | | | | | | | | | | | | | | | | 136 | | | | | | | | | | | | | | | | | | | | | | | | | | | | | | | | | | | | | | | | | | | | | SIPSAEDEDFNYR | | | | | | | | | | | | | | | | | | | | | | | | | | | | | | | | | | | | | | | | | | | | | | | | | | | | | | | | | | | | | | | | | | | | | | | | | | | | | | | | | | | | | | | | | | | | | | | | | | | | | | | | | | | | | | | | | | | | | | | | | | | | | | | | | | | | | | | | | | | | | | | | | | | | | | | | | | | | | | | | | | | | | | | | | | | | | |  |  |  |  |  |  |  |  |  |  |  |  |  |  |  |  |  |  |  |  |  |  |  |  |  |  |  |  |  |  |
|  | | | | 1591.8205 | | | | | | | | | | | | | | | 1591.819 | | | | | | | | | | | | | | | | | | | | | | | | | | | | | | 0 | | | | | | | | | | | | | | | | | | | | | | | | | | | | | | | | | -1 | | | | | | | | | | | | | | | | | | | | | | | | | | | | | | | | | | | 346 | | | | | | | | | | | | | | | | | | | | | | | | | | | | | | | | | | | | | | | | | | 360 | | | | | | | | | | | | | | | | | | | | | | | | | | | | | | | | | | | | | | | | | | | | | DKAADNIAANLYSVK | | | | | | | | | | | | | | | | | | | | | | | | | | | | | | | | | | | | | | | | | | | | | | | | | | | | | | | | | | | | | | | | | | | | | | | | | | | | | | | | | | | | | | | | | | | | | | | | | | | | | | | | | | | | | | | | | | | | | | | | | | | | | | | | | | | | | | | | | | | | | | | | | | | | | | | | | | | | | | | | | | | | | | | | | | | | | |  |  |  |  |  |  |  |  |  |  |  |  |  |  |  |  |  |  |  |  |  |  |  |  |  |  |  |  |  |  |
|  | | | | 1591.8205 | | | | | | | | | | | | | | | 1591.821 | | | | | | | | | | | | | | | | | | | | | | | | | | | | | | 0 | | | | | | | | | | | | | | | | | | | | | | | | | | | | | | | | | 0 | | | | | | | | | | | | | | | | | | | | | | | | | | | | | | | | | | | 346 | | | | | | | | | | | | | | | | | | | | | | | | | | | | | | | | | | | | | | | | | | 360 | | | | | | | | | | | | | | | | | | | | | | | | | | | | | | | | | | | | | | | | | | | | | DKAADNIAANLYSVK | | | | | | | | | | | | | | | | | | | | | | | | | | | | | | | | | | | | | | | | | | | | | | | | | | | | | | | | | | | | | | | | | | | | | | | | | | | | | | | | | | | | | | | | | | | | | | | | | | | | | | | | | | | | | | | | | | | | | | | | | | | | | | | | | | | | | | | | | | | | | | | | | | | | | | | | | | | | | | | | | | | | | | | | | | | | | |  |  |  |  |  |  |  |  |  |  |  |  |  |  |  |  |  |  |  |  |  |  |  |  |  |  |  |  |  |  |
| Spot no. | | | | Homologous protein | | | | | | | | | | | | | | | Accession no. | | | | | | | | | | | | | | | | | | | | | | | | | | | | | | Score | | | | | | | | | | | | | | | | | | | | | | | | | | | | | | | | | M.P. | | | | | | | | | | | | | | | | | | | | | | | | | | | | | | | | | | | Cov. (%) | | | | | | | | | | | | | | | | | | | | | | | | | | | | | | | | | | | | | | | | | | Blast score | | | | | | | | | | | | | | | | | | | | | | | | | | | | | | | | | | | | | | | | | | | | | Mr (kDa) / pI | | | | | | | | | | | | | | | | | | | | | | | | | | | | | | | | | | | | | | | | | | | | | | | | | | | | | | | | | | | | | | | | | | | | | | | | | | | | | | | | | | | | | | | | | | | | | | | | | | | | | | | | | | | | | | | | | | | | | | | | | | | | | | | | | | | | | | | | | | | | | | | | | | | | | | | | | | | | | | Ratio | | | | | | | | | | | | | | | | | | | | | | | | | | | | | |  |  |  |  |  |  |  |  |  |  |  |  |  |  |  |  |
|  |  |  |  |  |  |  |  |  |  |  |  |  |  |  |  |  |  |  |  |  |  |  |  |  |  |  |  |  |  |  |  |  |  |  |  |  |  |  |  |  |  |  |  |  |  |  |  |  |  |  |  |  |  |  |  |  |  |  |  |  |  |  |  |  |  |  |  |  |  |  |  |  |  |  |  |  |  |  |  |  |  |  |  |  |  |  |  |  |  |  |  |  |  |  |  |  |  |  |  |  |  |  |  |  |  |  |  |  |  |  |  |  |  |  |  |  |  |  |  |  |  |  |  |  |  |  |  |  |  |  |  |  |  |  |  |  |  |  |  |  |  |  |  |  |  |  |  |  |  |  |  |  |  |  |  |  |  |  |  |  |  |  |  |  |  |  |  |  |  |  |  |  |  |  |  |  |  |  |  |  |  |  |  |  |  |  |  |  |  |  |  |  |  |  |  |  |  |  |  |  |  |  |  | Theo. | | | | | | | | | | | | | | | | | | | | | | | | | | | | | | | | | | | | | | | | | | | | | | | | | | | | | | | | | | | | | | | | | | Exp. | | | | | | | | | | | | | | | | | | | | | | | | | | | | | | | | | | | | | | | | | | | | | | | | | | | | | | | | | | | | | | | | | | | | | | | | | | | | | | | | | | | | | | | | | | | |  |  |  |  |  |  |  |  |  |  |  |  |  |  |  |  |  |  |  |  |  |  |  |  |  |  |  |  |  |  |  |  |  |  |  |  |  |  |  |  |  |  |  |  |  |  |
| 4a | | | | methionine synthase | | | | | | | | | | | | | | | Glyma16g04240.1 | | | | | | | | | | | | | | | | | | | | | | | | | | | | | | 122 | | | | | | | | | | | | | | | | | | | | | | | | | | | | | | | | | 2 | | | | | | | | | | | | | | | | | | | | | | | | | | | | | | | | | | | 4 | | | | | | | | | | | | | | | | | | | | | | | | | | | | | | | | | | | | | | | | | | 1491 | | | | | | | | | | | | | | | | | | | | | | | | | | | | | | | | | | | | | | | | | | | | | 84.6/5.93 | | | | | | | | | | | | | | | | | | | | | | | | | | | | | | | | | | | | | | | | | | | | | | | | | | | | | | | | | | | | | | | | | |  | | | | | | | | | | | | | | | | | | | | | | | | | | | | | | | | | | | | | | | | | | | | | | | | | | | | | | | | | | | | | | | | | | | | | | | | | | | | | | | | | | | | | | | | | | | |  | | | | | | | | | | | | | | | | | | | | | | | | | | | | | |  |  |  |  |  |  |  |  |  |  |  |  |  |  |  |  |
|  | | | | Calc. Mass | | | | | | | | | | | | | | | Observ. Mass | | | | | | | | | | | | | | | | | | | | | | | | | | | | | | Da | | | | | | | | | | | | | | | | | | | | | | | | | | | | | | | | | ppm | | | | | | | | | | | | | | | | | | | | | | | | | | | | | | | | | | | start | | | | | | | | | | | | | | | | | | | | | | | | | | | | | | | | | | | | | | | | | | end | | | | | | | | | | | | | | | | | | | | | | | | | | | | | | | | | | | | | | | | | | | | | sequence | | | | | | | | | | | | | | | | | | | | | | | | | | | | | | | | | | | | | | | | | | | | | | | | | | | | | | | | | | | | | | | | | | | | | | | | | | | | | | | | | | | | | | | | | | | | | | | | | | | | | | | | | | | | | | | | | | | | | | | | | | | | | | | | | | | | | | | | | | | | | | | | | | | | | | | | | | | | | | | | | | | | | | | | | | | | | | | | | | | | | | | | | | | |  |  |  |  |  |  |  |  |  |  |  |  |  |  |  |  |
|  | | | | 2317.1801 | | | | | | | | | | | | | | | 2317.181 | | | | | | | | | | | | | | | | | | | | | | | | | | | | | | 0 | | | | | | | | | | | | | | | | | | | | | | | | | | | | | | | | | 0 | | | | | | | | | | | | | | | | | | | | | | | | | | | | | | | | | | | 474 | | | | | | | | | | | | | | | | | | | | | | | | | | | | | | | | | | | | | | | | | | 493 | | | | | | | | | | | | | | | | | | | | | | | | | | | | | | | | | | | | | | | | | | | | | VVELQEELDIDVLVHGEPER | | | | | | | | | | | | | | | | | | | | | | | | | | | | | | | | | | | | | | | | | | | | | | | | | | | | | | | | | | | | | | | | | | | | | | | | | | | | | | | | | | | | | | | | | | | | | | | | | | | | | | | | | | | | | | | | | | | | | | | | | | | | | | | | | | | | | | | | | | | | | | | | | | | | | | | | | | | | | | | | | | | | | | | | | | | | | | | | | | | | | | | | | | | |  |  |  |  |  |  |  |  |  |  |  |  |  |  |  |  |
|  | | | | 1468.8249 | | | | | | | | | | | | | | | 1468.824 | | | | | | | | | | | | | | | | | | | | | | | | | | | | | | 0 | | | | | | | | | | | | | | | | | | | | | | | | | | | | | | | | | -1 | | | | | | | | | | | | | | | | | | | | | | | | | | | | | | | | | | | 597 | | | | | | | | | | | | | | | | | | | | | | | | | | | | | | | | | | | | | | | | | | 610 | | | | | | | | | | | | | | | | | | | | | | | | | | | | | | | | | | | | | | | | | | | | | AGITVIQIDEAALR | | | | | | | | | | | | | | | | | | | | | | | | | | | | | | | | | | | | | | | | | | | | | | | | | | | | | | | | | | | | | | | | | | | | | | | | | | | | | | | | | | | | | | | | | | | | | | | | | | | | | | | | | | | | | | | | | | | | | | | | | | | | | | | | | | | | | | | | | | | | | | | | | | | | | | | | | | | | | | | | | | | | | | | | | | | | | | | | | | | | | | | | | | | |  |  |  |  |  |  |  |  |  |  |  |  |  |  |  |  |
| Spot no. | | | | Homologous protein | | | | | | | | | | | | | | | Accession no. | | | | | | | | | | | | | | | | | | | | | | | | | | | | | | Score | | | | | | | | | | | | | | | | | | | | | | | | | | | | | | | | | M.P. | | | | | | | | | | | | | | | | | | | | | | | | | | | | | | | | | | | Cov. (%) | | | | | | | | | | | | | | | | | | | | | | | | | | | | | | | | | | | | | | | | | | Blast score | | | | | | | | | | | | | | | | | | | | | | | | | | | | | | | | | | | | | | | | | | | | | Mr (kDa) / pI | | | | | | | | | | | | | | | | | | | | | | | | | | | | | | | | | | | | | | | | | | | | | | | | | | | | | | | | | | | | | | | | | | | | | | | | | | | | | | | | | | | | | | | | | | | | | | | | | | | | | | | | | | | | | | | | | | | | | | | | | | | | | | | | | | | | | | | | | | | | | | | | | | | | | | | | | Ratio | | | | | | | | | | | | | | | | | | | | | | | | | | | | | | | | | | |  |  |  |  |  |  |  |  |  |  |  |  |  |  |  |  |
|  |  |  |  |  |  |  |  |  |  |  |  |  |  |  |  |  |  |  |  |  |  |  |  |  |  |  |  |  |  |  |  |  |  |  |  |  |  |  |  |  |  |  |  |  |  |  |  |  |  |  |  |  |  |  |  |  |  |  |  |  |  |  |  |  |  |  |  |  |  |  |  |  |  |  |  |  |  |  |  |  |  |  |  |  |  |  |  |  |  |  |  |  |  |  |  |  |  |  |  |  |  |  |  |  |  |  |  |  |  |  |  |  |  |  |  |  |  |  |  |  |  |  |  |  |  |  |  |  |  |  |  |  |  |  |  |  |  |  |  |  |  |  |  |  |  |  |  |  |  |  |  |  |  |  |  |  |  |  |  |  |  |  |  |  |  |  |  |  |  |  |  |  |  |  |  |  |  |  |  |  |  |  |  |  |  |  |  |  |  |  |  |  |  |  |  |  |  |  |  |  |  |  |  | Theo. | | | | | | | | | | | | | | | | | | | | | | | | | | | | | | | | | | | | | | | | | | | | | | | | | | | | | | | | | | | | | | | | | | Exp. | | | | | | | | | | | | | | | | | | | | | | | | | | | | | | | | | | | | | | | | | | | | | | | | | | | | | | | | | | | | | | | | | | | | | | | | | | | | | | | | | | | | | | |  |  |  |  |  |  |  |  |  |  |  |  |  |  |  |  |  |  |  |  |  |  |  |  |  |  |  |  |  |  |  |  |  |  |  |  |  |  |  |  |  |  |  |  |  |  |  |  |  |  |  |
| 4b | | | | Phosphoglycerate kinase | | | | | | | | | | | | | | | Glyma08g17600.1 | | | | | | | | | | | | | | | | | | | | | | | | | | | | | | 107 | | | | | | | | | | | | | | | | | | | | | | | | | | | | | | | | | 3 | | | | | | | | | | | | | | | | | | | | | | | | | | | | | | | | | | | 11 | | | | | | | | | | | | | | | | | | | | | | | | | | | | | | | | | | | | | | | | | | 735 | | | | | | | | | | | | | | | | | | | | | | | | | | | | | | | | | | | | | | | | | | | | | 42.6/6.28 | | | | | | | | | | | | | | | | | | | | | | | | | | | | | | | | | | | | | | | | | | | | | | | | | | | | | | | | | | | | | | | | | |  | | | | | | | | | | | | | | | | | | | | | | | | | | | | | | | | | | | | | | | | | | | | | | | | | | | | | | | | | | | | | | | | | | | | | | | | | | | | | | | | | | | | | | |  | | | | | | | | | | | | | | | | | | | | | | | | | | | | | | | | | | |  |  |  |  |  |  |  |  |  |  |  |  |  |  |  |  |
|  | | | | Calc. Mass | | | | | | | | | | | | | | | Observ. Mass | | | | | | | | | | | | | | | | | | | | | | | | | | | | | | Da | | | | | | | | | | | | | | | | | | | | | | | | | | | | | | | | | ppm | | | | | | | | | | | | | | | | | | | | | | | | | | | | | | | | | | | start | | | | | | | | | | | | | | | | | | | | | | | | | | | | | | | | | | | | | | | | | | end | | | | | | | | | | | | | | | | | | | | | | | | | | | | | | | | | | | | | | | | | | | | | sequence | | | | | | | | | | | | | | | | | | | | | | | | | | | | | | | | | | | | | | | | | | | | | | | | | | | | | | | | | | | | | | | | | | | | | | | | | | | | | | | | | | | | | | | | | | | | | | | | | | | | | | | | | | | | | | | | | | | | | | | | | | | | | | | | | | | | | | | | | | | | | | | | | | | | | | | | | | | | | | | | | | | | | | | | | | | | | | | | | | | | | | | | | | | |  |  |  |  |  |  |  |  |  |  |  |  |  |  |  |  |
|  | | | | 1403.7296 | | | | | | | | | | | | | | | 1403.729 | | | | | | | | | | | | | | | | | | | | | | | | | | | | | | 0 | | | | | | | | | | | | | | | | | | | | | | | | | | | | | | | | | -1 | | | | | | | | | | | | | | | | | | | | | | | | | | | | | | | | | | | 179 | | | | | | | | | | | | | | | | | | | | | | | | | | | | | | | | | | | | | | | | | | 191 | | | | | | | | | | | | | | | | | | | | | | | | | | | | | | | | | | | | | | | | | | | | | ELDYLVGAVSNPK | | | | | | | | | | | | | | | | | | | | | | | | | | | | | | | | | | | | | | | | | | | | | | | | | | | | | | | | | | | | | | | | | | | | | | | | | | | | | | | | | | | | | | | | | | | | | | | | | | | | | | | | | | | | | | | | | | | | | | | | | | | | | | | | | | | | | | | | | | | | | | | | | | | | | | | | | | | | | | | | | | | | | | | | | | | | | | | | | | | | | | | | | | | |  |  |  |  |  |  |  |  |  |  |  |  |  |  |  |  |
|  | | | | 1538.892 | | | | | | | | | | | | | | | 1538.89 | | | | | | | | | | | | | | | | | | | | | | | | | | | | | | 0 | | | | | | | | | | | | | | | | | | | | | | | | | | | | | | | | | -1 | | | | | | | | | | | | | | | | | | | | | | | | | | | | | | | | | | | 262 | | | | | | | | | | | | | | | | | | | | | | | | | | | | | | | | | | | | | | | | | | 276 | | | | | | | | | | | | | | | | | | | | | | | | | | | | | | | | | | | | | | | | | | | | | GVSLLLPTDVVIADK | | | | | | | | | | | | | | | | | | | | | | | | | | | | | | | | | | | | | | | | | | | | | | | | | | | | | | | | | | | | | | | | | | | | | | | | | | | | | | | | | | | | | | | | | | | | | | | | | | | | | | | | | | | | | | | | | | | | | | | | | | | | | | | | | | | | | | | | | | | | | | | | | | | | | | | | | | | | | | | | | | | | | | | | | | | | | | | | | | | | | | | | | | | |  |  |  |  |  |  |  |  |  |  |  |  |  |  |  |  |
|  | | | | 1572.8359 | | | | | | | | | | | | | | | 1572.836 | | | | | | | | | | | | | | | | | | | | | | | | | | | | | | 0 | | | | | | | | | | | | | | | | | | | | | | | | | | | | | | | | | 0 | | | | | | | | | | | | | | | | | | | | | | | | | | | | | | | | | | | 350 | | | | | | | | | | | | | | | | | | | | | | | | | | | | | | | | | | | | | | | | | | 366 | | | | | | | | | | | | | | | | | | | | | | | | | | | | | | | | | | | | | | | | | | | | | GVTTIIGGGDSVAAVEK | | | | | | | | | | | | | | | | | | | | | | | | | | | | | | | | | | | | | | | | | | | | | | | | | | | | | | | | | | | | | | | | | | | | | | | | | | | | | | | | | | | | | | | | | | | | | | | | | | | | | | | | | | | | | | | | | | | | | | | | | | | | | | | | | | | | | | | | | | | | | | | | | | | | | | | | | | | | | | | | | | | | | | | | | | | | | | | | | | | | | | | | | | | |  |  |  |  |  |  |  |  |  |  |  |  |  |  |  |  |
| Spot no. | | | | Homologous protein | | | | | | | | | | | | | | | | | Accession no. | | | | | | | | | | | | | | | | | | | | | | | | | | | | | | | | Score | | | | | | | | | | | | | | | | | | | | | | | | | | | | | | | | M.P. | | | | | | | | | | | | | | | | | | | | | | | | | | | | | | | | | | Cov. (%) | | | | | | | | | | | | | | | | | | | | | | | | | | | | | | | | | | | | | | | | Blast score | | | | | | | | | | | | | | | | | | | | | | | | | | | | | | | | | | | | | | | | | | | | | Mr (kDa) / pI | | | | | | | | | | | | | | | | | | | | | | | | | | | | | | | | | | | | | | | | | | | | | | | | | | | | | | | | | | | | | | | | | | | | | | | | | | | | | | | | | | | | | | | | | | | | | | | | | | | | | | | | | | | | | | | | | | | | | | | | | | | | | | | | | | | | | | | | | | | | | | | | | | | | | | | | | Ratio | | | | | | | | | | | | | | | | | | | | | | | | | | | | | | | | | | |  |  |  |  |  |  |  |  |  |  |  |  |  |  |  |  |
|  |  |  |  |  |  |  |  |  |  |  |  |  |  |  |  |  |  |  |  |  |  |  |  |  |  |  |  |  |  |  |  |  |  |  |  |  |  |  |  |  |  |  |  |  |  |  |  |  |  |  |  |  |  |  |  |  |  |  |  |  |  |  |  |  |  |  |  |  |  |  |  |  |  |  |  |  |  |  |  |  |  |  |  |  |  |  |  |  |  |  |  |  |  |  |  |  |  |  |  |  |  |  |  |  |  |  |  |  |  |  |  |  |  |  |  |  |  |  |  |  |  |  |  |  |  |  |  |  |  |  |  |  |  |  |  |  |  |  |  |  |  |  |  |  |  |  |  |  |  |  |  |  |  |  |  |  |  |  |  |  |  |  |  |  |  |  |  |  |  |  |  |  |  |  |  |  |  |  |  |  |  |  |  |  |  |  |  |  |  |  |  |  |  |  |  |  |  |  |  |  |  |  |  | Theo. | | | | | | | | | | | | | | | | | | | | | | | | | | | | | | | | | | | | | | | | | | | | | | | | | | | | | | | | | | | | | | | | | | Exp. | | | | | | | | | | | | | | | | | | | | | | | | | | | | | | | | | | | | | | | | | | | | | | | | | | | | | | | | | | | | | | | | | | | | | | | | | | | | | | | | | | | | | | |  |  |  |  |  |  |  |  |  |  |  |  |  |  |  |  |  |  |  |  |  |  |  |  |  |  |  |  |  |  |  |  |  |  |  |  |  |  |  |  |  |  |  |  |  |  |  |  |  |  |  |
| 5a | | | | Heat shock 70 kDa protein | | | | | | | | | | | | | | | | | Glyma17g08020.1 | | | | | | | | | | | | | | | | | | | | | | | | | | | | | | | | 2152 | | | | | | | | | | | | | | | | | | | | | | | | | | | | | | | | 25 | | | | | | | | | | | | | | | | | | | | | | | | | | | | | | | | | | 38 | | | | | | | | | | | | | | | | | | | | | | | | | | | | | | | | | | | | | | | | 1144 | | | | | | | | | | | | | | | | | | | | | | | | | | | | | | | | | | | | | | | | | | | | | 71.4/5.27 | | | | | | | | | | | | | | | | | | | | | | | | | | | | | | | | | | | | | | | | | | | | | | | | | | | | | | | | | | | | | | | | | |  | | | | | | | | | | | | | | | | | | | | | | | | | | | | | | | | | | | | | | | | | | | | | | | | | | | | | | | | | | | | | | | | | | | | | | | | | | | | | | | | | | | | | | |  | | | | | | | | | | | | | | | | | | | | | | | | | | | | | | | | | | |  |  |  |  |  |  |  |  |  |  |  |  |  |  |  |  |
|  | | | | Calc. Mass | | | | | | | | | | | | | | | | | Observ. Mass | | | | | | | | | | | | | | | | | | | | | | | | | | | | | | | | Da | | | | | | | | | | | | | | | | | | | | | | | | | | | | | | | | ppm | | | | | | | | | | | | | | | | | | | | | | | | | | | | | | | | | | start | | | | | | | | | | | | | | | | | | | | | | | | | | | | | | | | | | | | | | | | end | | | | | | | | | | | | | | | | | | | | | | | | | | | | | | | | | | | | | | | | | | | | | sequence | | | | | | | | | | | | | | | | | | | | | | | | | | | | | | | | | | | | | | | | | | | | | | | | | | | | | | | | | | | | | | | | | | | | | | | | | | | | | | | | | | | | | | | | | | | | | | | | | | | | | | | | | | | | | | | | | | | | | | | | | | | | | | | | | | | | | | | | | | | | | | | | | | | | | | | | | | | | | | | | | | | | | | | | | | | | | | | | | | | | | | | | | | | |  |  |  |  |  |  |  |  |  |  |  |  |  |  |  |  |
|  | | | | 1486.694 | | | | | | | | | | | | | | | | | 1486.692 | | | | | | | | | | | | | | | | | | | | | | | | | | | | | | | | 0 | | | | | | | | | | | | | | | | | | | | | | | | | | | | | | | | -1 | | | | | | | | | | | | | | | | | | | | | | | | | | | | | | | | | | 39 | | | | | | | | | | | | | | | | | | | | | | | | | | | | | | | | | | | | | | | | 51 | | | | | | | | | | | | | | | | | | | | | | | | | | | | | | | | | | | | | | | | | | | | | TTPSYVAFTDTER | | | | | | | | | | | | | | | | | | | | | | | | | | | | | | | | | | | | | | | | | | | | | | | | | | | | | | | | | | | | | | | | | | | | | | | | | | | | | | | | | | | | | | | | | | | | | | | | | | | | | | | | | | | | | | | | | | | | | | | | | | | | | | | | | | | | | | | | | | | | | | | | | | | | | | | | | | | | | | | | | | | | | | | | | | | | | | | | | | | | | | | | | | | |  |  |  |  |  |  |  |  |  |  |  |  |  |  |  |  |
|  | | | | 1486.694 | | | | | | | | | | | | | | | | | 1486.693 | | | | | | | | | | | | | | | | | | | | | | | | | | | | | | | | 0 | | | | | | | | | | | | | | | | | | | | | | | | | | | | | | | | -1 | | | | | | | | | | | | | | | | | | | | | | | | | | | | | | | | | | 39 | | | | | | | | | | | | | | | | | | | | | | | | | | | | | | | | | | | | | | | | 51 | | | | | | | | | | | | | | | | | | | | | | | | | | | | | | | | | | | | | | | | | | | | | TTPSYVAFTDTER | | | | | | | | | | | | | | | | | | | | | | | | | | | | | | | | | | | | | | | | | | | | | | | | | | | | | | | | | | | | | | | | | | | | | | | | | | | | | | | | | | | | | | | | | | | | | | | | | | | | | | | | | | | | | | | | | | | | | | | | | | | | | | | | | | | | | | | | | | | | | | | | | | | | | | | | | | | | | | | | | | | | | | | | | | | | | | | | | | | | | | | | | | | |  |  |  |  |  |  |  |  |  |  |  |  |  |  |  |  |
|  | | | | 1486.694 | | | | | | | | | | | | | | | | | 1486.693 | | | | | | | | | | | | | | | | | | | | | | | | | | | | | | | | 0 | | | | | | | | | | | | | | | | | | | | | | | | | | | | | | | | -1 | | | | | | | | | | | | | | | | | | | | | | | | | | | | | | | | | | 39 | | | | | | | | | | | | | | | | | | | | | | | | | | | | | | | | | | | | | | | | 51 | | | | | | | | | | | | | | | | | | | | | | | | | | | | | | | | | | | | | | | | | | | | | TTPSYVAFTDTER | | | | | | | | | | | | | | | | | | | | | | | | | | | | | | | | | | | | | | | | | | | | | | | | | | | | | | | | | | | | | | | | | | | | | | | | | | | | | | | | | | | | | | | | | | | | | | | | | | | | | | | | | | | | | | | | | | | | | | | | | | | | | | | | | | | | | | | | | | | | | | | | | | | | | | | | | | | | | | | | | | | | | | | | | | | | | | | | | | | | | | | | | | | |  |  |  |  |  |  |  |  |  |  |  |  |  |  |  |  |
|  | | | | 1486.694 | | | | | | | | | | | | | | | | | 1486.693 | | | | | | | | | | | | | | | | | | | | | | | | | | | | | | | | 0 | | | | | | | | | | | | | | | | | | | | | | | | | | | | | | | | -1 | | | | | | | | | | | | | | | | | | | | | | | | | | | | | | | | | | 39 | | | | | | | | | | | | | | | | | | | | | | | | | | | | | | | | | | | | | | | | 51 | | | | | | | | | | | | | | | | | | | | | | | | | | | | | | | | | | | | | | | | | | | | | TTPSYVAFTDTER | | | | | | | | | | | | | | | | | | | | | | | | | | | | | | | | | | | | | | | | | | | | | | | | | | | | | | | | | | | | | | | | | | | | | | | | | | | | | | | | | | | | | | | | | | | | | | | | | | | | | | | | | | | | | | | | | | | | | | | | | | | | | | | | | | | | | | | | | | | | | | | | | | | | | | | | | | | | | | | | | | | | | | | | | | | | | | | | | | | | | | | | | | | |  |  |  |  |  |  |  |  |  |  |  |  |  |  |  |  |
|  | | | | 1486.694 | | | | | | | | | | | | | | | | | 1486.693 | | | | | | | | | | | | | | | | | | | | | | | | | | | | | | | | 0 | | | | | | | | | | | | | | | | | | | | | | | | | | | | | | | | -1 | | | | | | | | | | | | | | | | | | | | | | | | | | | | | | | | | | 39 | | | | | | | | | | | | | | | | | | | | | | | | | | | | | | | | | | | | | | | | 51 | | | | | | | | | | | | | | | | | | | | | | | | | | | | | | | | | | | | | | | | | | | | | TTPSYVAFTDTER | | | | | | | | | | | | | | | | | | | | | | | | | | | | | | | | | | | | | | | | | | | | | | | | | | | | | | | | | | | | | | | | | | | | | | | | | | | | | | | | | | | | | | | | | | | | | | | | | | | | | | | | | | | | | | | | | | | | | | | | | | | | | | | | | | | | | | | | | | | | | | | | | | | | | | | | | | | | | | | | | | | | | | | | | | | | | | | | | | | | | | | | | | | |  |  |  |  |  |  |  |  |  |  |  |  |  |  |  |  |
|  | | | | 1486.694 | | | | | | | | | | | | | | | | | 1486.693 | | | | | | | | | | | | | | | | | | | | | | | | | | | | | | | | 0 | | | | | | | | | | | | | | | | | | | | | | | | | | | | | | | | -1 | | | | | | | | | | | | | | | | | | | | | | | | | | | | | | | | | | 39 | | | | | | | | | | | | | | | | | | | | | | | | | | | | | | | | | | | | | | | | 51 | | | | | | | | | | | | | | | | | | | | | | | | | | | | | | | | | | | | | | | | | | | | | TTPSYVAFTDTER | | | | | | | | | | | | | | | | | | | | | | | | | | | | | | | | | | | | | | | | | | | | | | | | | | | | | | | | | | | | | | | | | | | | | | | | | | | | | | | | | | | | | | | | | | | | | | | | | | | | | | | | | | | | | | | | | | | | | | | | | | | | | | | | | | | | | | | | | | | | | | | | | | | | | | | | | | | | | | | | | | | | | | | | | | | | | | | | | | | | | | | | | | | |  |  |  |  |  |  |  |  |  |  |  |  |  |  |  |  |
|  | | | | 1486.694 | | | | | | | | | | | | | | | | | 1486.693 | | | | | | | | | | | | | | | | | | | | | | | | | | | | | | | | 0 | | | | | | | | | | | | | | | | | | | | | | | | | | | | | | | | -1 | | | | | | | | | | | | | | | | | | | | | | | | | | | | | | | | | | 39 | | | | | | | | | | | | | | | | | | | | | | | | | | | | | | | | | | | | | | | | 51 | | | | | | | | | | | | | | | | | | | | | | | | | | | | | | | | | | | | | | | | | | | | | TTPSYVAFTDTER | | | | | | | | | | | | | | | | | | | | | | | | | | | | | | | | | | | | | | | | | | | | | | | | | | | | | | | | | | | | | | | | | | | | | | | | | | | | | | | | | | | | | | | | | | | | | | | | | | | | | | | | | | | | | | | | | | | | | | | | | | | | | | | | | | | | | | | | | | | | | | | | | | | | | | | | | | | | | | | | | | | | | | | | | | | | | | | | | | | | | | | | | | | |  |  |  |  |  |  |  |  |  |  |  |  |  |  |  |  |
|  | | | | 1486.694 | | | | | | | | | | | | | | | | | 1486.694 | | | | | | | | | | | | | | | | | | | | | | | | | | | | | | | | 0 | | | | | | | | | | | | | | | | | | | | | | | | | | | | | | | | 0 | | | | | | | | | | | | | | | | | | | | | | | | | | | | | | | | | | 39 | | | | | | | | | | | | | | | | | | | | | | | | | | | | | | | | | | | | | | | | 51 | | | | | | | | | | | | | | | | | | | | | | | | | | | | | | | | | | | | | | | | | | | | | TTPSYVAFTDTER | | | | | | | | | | | | | | | | | | | | | | | | | | | | | | | | | | | | | | | | | | | | | | | | | | | | | | | | | | | | | | | | | | | | | | | | | | | | | | | | | | | | | | | | | | | | | | | | | | | | | | | | | | | | | | | | | | | | | | | | | | | | | | | | | | | | | | | | | | | | | | | | | | | | | | | | | | | | | | | | | | | | | | | | | | | | | | | | | | | | | | | | | | | |  |  |  |  |  |  |  |  |  |  |  |  |  |  |  |  |
|  | | | | 1675.7988 | | | | | | | | | | | | | | | | | 1675.795 | | | | | | | | | | | | | | | | | | | | | | | | | | | | | | | | 0 | | | | | | | | | | | | | | | | | | | | | | | | | | | | | | | | -2 | | | | | | | | | | | | | | | | | | | | | | | | | | | | | | | | | | 59 | | | | | | | | | | | | | | | | | | | | | | | | | | | | | | | | | | | | | | | | 73 | | | | | | | | | | | | | | | | | | | | | | | | | | | | | | | | | | | | | | | | | | | | | NQVAMNPQNTVFDAK | | | | | | | | | | | | | | | | | | | | | | | | | | | | | | | | | | | | | | | | | | | | | | | | | | | | | | | | | | | | | | | | | | | | | | | | | | | | | | | | | | | | | | | | | | | | | | | | | | | | | | | | | | | | | | | | | | | | | | | | | | | | | | | | | | | | | | | | | | | | | | | | | | | | | | | | | | | | | | | | | | | | | | | | | | | | | | | | | | | | | | | | | | | |  |  |  |  |  |  |  |  |  |  |  |  |  |  |  |  |
|  | | | | 1675.7988 | | | | | | | | | | | | | | | | | 1675.797 | | | | | | | | | | | | | | | | | | | | | | | | | | | | | | | | 0 | | | | | | | | | | | | | | | | | | | | | | | | | | | | | | | | -1 | | | | | | | | | | | | | | | | | | | | | | | | | | | | | | | | | | 59 | | | | | | | | | | | | | | | | | | | | | | | | | | | | | | | | | | | | | | | | 73 | | | | | | | | | | | | | | | | | | | | | | | | | | | | | | | | | | | | | | | | | | | | | NQVAMNPQNTVFDAK | | | | | | | | | | | | | | | | | | | | | | | | | | | | | | | | | | | | | | | | | | | | | | | | | | | | | | | | | | | | | | | | | | | | | | | | | | | | | | | | | | | | | | | | | | | | | | | | | | | | | | | | | | | | | | | | | | | | | | | | | | | | | | | | | | | | | | | | | | | | | | | | | | | | | | | | | | | | | | | | | | | | | | | | | | | | | | | | | | | | | | | | | | | |  |  |  |  |  |  |  |  |  |  |  |  |  |  |  |  |
|  | | | | 1675.7988 | | | | | | | | | | | | | | | | | 1675.797 | | | | | | | | | | | | | | | | | | | | | | | | | | | | | | | | 0 | | | | | | | | | | | | | | | | | | | | | | | | | | | | | | | | -1 | | | | | | | | | | | | | | | | | | | | | | | | | | | | | | | | | | 59 | | | | | | | | | | | | | | | | | | | | | | | | | | | | | | | | | | | | | | | | 73 | | | | | | | | | | | | | | | | | | | | | | | | | | | | | | | | | | | | | | | | | | | | | NQVAMNPQNTVFDAK | | | | | | | | | | | | | | | | | | | | | | | | | | | | | | | | | | | | | | | | | | | | | | | | | | | | | | | | | | | | | | | | | | | | | | | | | | | | | | | | | | | | | | | | | | | | | | | | | | | | | | | | | | | | | | | | | | | | | | | | | | | | | | | | | | | | | | | | | | | | | | | | | | | | | | | | | | | | | | | | | | | | | | | | | | | | | | | | | | | | | | | | | | | |  |  |  |  |  |  |  |  |  |  |  |  |  |  |  |  |
|  | | | | 1675.7988 | | | | | | | | | | | | | | | | | 1675.799 | | | | | | | | | | | | | | | | | | | | | | | | | | | | | | | | 0 | | | | | | | | | | | | | | | | | | | | | | | | | | | | | | | | 0 | | | | | | | | | | | | | | | | | | | | | | | | | | | | | | | | | | 59 | | | | | | | | | | | | | | | | | | | | | | | | | | | | | | | | | | | | | | | | 73 | | | | | | | | | | | | | | | | | | | | | | | | | | | | | | | | | | | | | | | | | | | | | NQVAMNPQNTVFDAK | | | | | | | | | | | | | | | | | | | | | | | | | | | | | | | | | | | | | | | | | | | | | | | | | | | | | | | | | | | | | | | | | | | | | | | | | | | | | | | | | | | | | | | | | | | | | | | | | | | | | | | | | | | | | | | | | | | | | | | | | | | | | | | | | | | | | | | | | | | | | | | | | | | | | | | | | | | | | | | | | | | | | | | | | | | | | | | | | | | | | | | | | | | |  |  |  |  |  |  |  |  |  |  |  |  |  |  |  |  |
|  | | | | 1691.7937 | | | | | | | | | | | | | | | | | 1691.792 | | | | | | | | | | | | | | | | | | | | | | | | | | | | | | | | 0 | | | | | | | | | | | | | | | | | | | | | | | | | | | | | | | | -1 | | | | | | | | | | | | | | | | | | | | | | | | | | | | | | | | | | 59 | | | | | | | | | | | | | | | | | | | | | | | | | | | | | | | | | | | | | | | | 73 | | | | | | | | | | | | | | | | | | | | | | | | | | | | | | | | | | | | | | | | | | | | | NQVAMNPQNTVFDAK | | | | | | | | | | | | | | | | | | | | | | | | | | | | | | | | | | | | | | | | | | | | | | | | | | | | | | | | | | | | | | | | | | | | | | | | | | | | | | | | | | | | | | | | | | | | | | | | | | | | | | | | | | | | | | | | | | | | | | | | | | | | | | | | | | | | | | | | | | | | | | | | | | | | | | | | | | | | | | | | | | | | | | | | | | | | | | | | | | | | | | | | | | | |  |  |  |  |  |  |  |  |  |  |  |  |  |  |  |  |
|  | | | | 1691.7937 | | | | | | | | | | | | | | | | | 1691.793 | | | | | | | | | | | | | | | | | | | | | | | | | | | | | | | | 0 | | | | | | | | | | | | | | | | | | | | | | | | | | | | | | | | -1 | | | | | | | | | | | | | | | | | | | | | | | | | | | | | | | | | | 59 | | | | | | | | | | | | | | | | | | | | | | | | | | | | | | | | | | | | | | | | 73 | | | | | | | | | | | | | | | | | | | | | | | | | | | | | | | | | | | | | | | | | | | | | NQVAMNPQNTVFDAK | | | | | | | | | | | | | | | | | | | | | | | | | | | | | | | | | | | | | | | | | | | | | | | | | | | | | | | | | | | | | | | | | | | | | | | | | | | | | | | | | | | | | | | | | | | | | | | | | | | | | | | | | | | | | | | | | | | | | | | | | | | | | | | | | | | | | | | | | | | | | | | | | | | | | | | | | | | | | | | | | | | | | | | | | | | | | | | | | | | | | | | | | | | |  |  |  |  |  |  |  |  |  |  |  |  |  |  |  |  |
|  | | | | 1831.8999 | | | | | | | | | | | | | | | | | 1831.899 | | | | | | | | | | | | | | | | | | | | | | | | | | | | | | | | 0 | | | | | | | | | | | | | | | | | | | | | | | | | | | | | | | | 0 | | | | | | | | | | | | | | | | | | | | | | | | | | | | | | | | | | 59 | | | | | | | | | | | | | | | | | | | | | | | | | | | | | | | | | | | | | | | | 74 | | | | | | | | | | | | | | | | | | | | | | | | | | | | | | | | | | | | | | | | | | | | | NQVAMNPQNTVFDAKR | | | | | | | | | | | | | | | | | | | | | | | | | | | | | | | | | | | | | | | | | | | | | | | | | | | | | | | | | | | | | | | | | | | | | | | | | | | | | | | | | | | | | | | | | | | | | | | | | | | | | | | | | | | | | | | | | | | | | | | | | | | | | | | | | | | | | | | | | | | | | | | | | | | | | | | | | | | | | | | | | | | | | | | | | | | | | | | | | | | | | | | | | | | |  |  |  |  |  |  |  |  |  |  |  |  |  |  |  |  |
|  | | | | 1831.8999 | | | | | | | | | | | | | | | | | 1831.9 | | | | | | | | | | | | | | | | | | | | | | | | | | | | | | | | 0 | | | | | | | | | | | | | | | | | | | | | | | | | | | | | | | | 0 | | | | | | | | | | | | | | | | | | | | | | | | | | | | | | | | | | 59 | | | | | | | | | | | | | | | | | | | | | | | | | | | | | | | | | | | | | | | | 74 | | | | | | | | | | | | | | | | | | | | | | | | | | | | | | | | | | | | | | | | | | | | | NQVAMNPQNTVFDAKR | | | | | | | | | | | | | | | | | | | | | | | | | | | | | | | | | | | | | | | | | | | | | | | | | | | | | | | | | | | | | | | | | | | | | | | | | | | | | | | | | | | | | | | | | | | | | | | | | | | | | | | | | | | | | | | | | | | | | | | | | | | | | | | | | | | | | | | | | | | | | | | | | | | | | | | | | | | | | | | | | | | | | | | | | | | | | | | | | | | | | | | | | | | |  |  |  |  |  |  |  |  |  |  |  |  |  |  |  |  |
|  | | | | 1847.8948 | | | | | | | | | | | | | | | | | 1847.893 | | | | | | | | | | | | | | | | | | | | | | | | | | | | | | | | 0 | | | | | | | | | | | | | | | | | | | | | | | | | | | | | | | | -1 | | | | | | | | | | | | | | | | | | | | | | | | | | | | | | | | | | 59 | | | | | | | | | | | | | | | | | | | | | | | | | | | | | | | | | | | | | | | | 74 | | | | | | | | | | | | | | | | | | | | | | | | | | | | | | | | | | | | | | | | | | | | | NQVAMNPQNTVFDAKR | | | | | | | | | | | | | | | | | | | | | | | | | | | | | | | | | | | | | | | | | | | | | | | | | | | | | | | | | | | | | | | | | | | | | | | | | | | | | | | | | | | | | | | | | | | | | | | | | | | | | | | | | | | | | | | | | | | | | | | | | | | | | | | | | | | | | | | | | | | | | | | | | | | | | | | | | | | | | | | | | | | | | | | | | | | | | | | | | | | | | | | | | | | |  |  |  |  |  |  |  |  |  |  |  |  |  |  |  |  |
|  | | | | 1847.8948 | | | | | | | | | | | | | | | | | 1847.893 | | | | | | | | | | | | | | | | | | | | | | | | | | | | | | | | 0 | | | | | | | | | | | | | | | | | | | | | | | | | | | | | | | | -1 | | | | | | | | | | | | | | | | | | | | | | | | | | | | | | | | | | 59 | | | | | | | | | | | | | | | | | | | | | | | | | | | | | | | | | | | | | | | | 74 | | | | | | | | | | | | | | | | | | | | | | | | | | | | | | | | | | | | | | | | | | | | | NQVAMNPQNTVFDAKR | | | | | | | | | | | | | | | | | | | | | | | | | | | | | | | | | | | | | | | | | | | | | | | | | | | | | | | | | | | | | | | | | | | | | | | | | | | | | | | | | | | | | | | | | | | | | | | | | | | | | | | | | | | | | | | | | | | | | | | | | | | | | | | | | | | | | | | | | | | | | | | | | | | | | | | | | | | | | | | | | | | | | | | | | | | | | | | | | | | | | | | | | | | |  |  |  |  |  |  |  |  |  |  |  |  |  |  |  |  |
|  | | | | 1847.8948 | | | | | | | | | | | | | | | | | 1847.894 | | | | | | | | | | | | | | | | | | | | | | | | | | | | | | | | 0 | | | | | | | | | | | | | | | | | | | | | | | | | | | | | | | | 0 | | | | | | | | | | | | | | | | | | | | | | | | | | | | | | | | | | 59 | | | | | | | | | | | | | | | | | | | | | | | | | | | | | | | | | | | | | | | | 74 | | | | | | | | | | | | | | | | | | | | | | | | | | | | | | | | | | | | | | | | | | | | | NQVAMNPQNTVFDAKR | | | | | | | | | | | | | | | | | | | | | | | | | | | | | | | | | | | | | | | | | | | | | | | | | | | | | | | | | | | | | | | | | | | | | | | | | | | | | | | | | | | | | | | | | | | | | | | | | | | | | | | | | | | | | | | | | | | | | | | | | | | | | | | | | | | | | | | | | | | | | | | | | | | | | | | | | | | | | | | | | | | | | | | | | | | | | | | | | | | | | | | | | | | |  |  |  |  |  |  |  |  |  |  |  |  |  |  |  |  |
|  | | | | 1256.5343 | | | | | | | | | | | | | | | | | 1256.532 | | | | | | | | | | | | | | | | | | | | | | | | | | | | | | | | 0 | | | | | | | | | | | | | | | | | | | | | | | | | | | | | | | | -2 | | | | | | | | | | | | | | | | | | | | | | | | | | | | | | | | | | 80 | | | | | | | | | | | | | | | | | | | | | | | | | | | | | | | | | | | | | | | | 90 | | | | | | | | | | | | | | | | | | | | | | | | | | | | | | | | | | | | | | | | | | | | | FSDSSVQNDMK | | | | | | | | | | | | | | | | | | | | | | | | | | | | | | | | | | | | | | | | | | | | | | | | | | | | | | | | | | | | | | | | | | | | | | | | | | | | | | | | | | | | | | | | | | | | | | | | | | | | | | | | | | | | | | | | | | | | | | | | | | | | | | | | | | | | | | | | | | | | | | | | | | | | | | | | | | | | | | | | | | | | | | | | | | | | | | | | | | | | | | | | | | | |  |  |  |  |  |  |  |  |  |  |  |  |  |  |  |  |
|  | | | | 1685.9175 | | | | | | | | | | | | | | | | | 1685.915 | | | | | | | | | | | | | | | | | | | | | | | | | | | | | | | | 0 | | | | | | | | | | | | | | | | | | | | | | | | | | | | | | | | -2 | | | | | | | | | | | | | | | | | | | | | | | | | | | | | | | | | | 96 | | | | | | | | | | | | | | | | | | | | | | | | | | | | | | | | | | | | | | | | 111 | | | | | | | | | | | | | | | | | | | | | | | | | | | | | | | | | | | | | | | | | | | | | VVAGPGDKPMIVVNYK | | | | | | | | | | | | | | | | | | | | | | | | | | | | | | | | | | | | | | | | | | | | | | | | | | | | | | | | | | | | | | | | | | | | | | | | | | | | | | | | | | | | | | | | | | | | | | | | | | | | | | | | | | | | | | | | | | | | | | | | | | | | | | | | | | | | | | | | | | | | | | | | | | | | | | | | | | | | | | | | | | | | | | | | | | | | | | | | | | | | | | | | | | | |  |  |  |  |  |  |  |  |  |  |  |  |  |  |  |  |
|  | | | | 2129.1191 | | | | | | | | | | | | | | | | | 2129.116 | | | | | | | | | | | | | | | | | | | | | | | | | | | | | | | | 0 | | | | | | | | | | | | | | | | | | | | | | | | | | | | | | | | -2 | | | | | | | | | | | | | | | | | | | | | | | | | | | | | | | | | | 96 | | | | | | | | | | | | | | | | | | | | | | | | | | | | | | | | | | | | | | | | 115 | | | | | | | | | | | | | | | | | | | | | | | | | | | | | | | | | | | | | | | | | | | | | VVAGPGDKPMIVVNYKGEEK | | | | | | | | | | | | | | | | | | | | | | | | | | | | | | | | | | | | | | | | | | | | | | | | | | | | | | | | | | | | | | | | | | | | | | | | | | | | | | | | | | | | | | | | | | | | | | | | | | | | | | | | | | | | | | | | | | | | | | | | | | | | | | | | | | | | | | | | | | | | | | | | | | | | | | | | | | | | | | | | | | | | | | | | | | | | | | | | | | | | | | | | | | | |  |  |  |  |  |  |  |  |  |  |  |  |  |  |  |  |
|  | | | | 2129.1191 | | | | | | | | | | | | | | | | | 2129.118 | | | | | | | | | | | | | | | | | | | | | | | | | | | | | | | | 0 | | | | | | | | | | | | | | | | | | | | | | | | | | | | | | | | 0 | | | | | | | | | | | | | | | | | | | | | | | | | | | | | | | | | | 96 | | | | | | | | | | | | | | | | | | | | | | | | | | | | | | | | | | | | | | | | 115 | | | | | | | | | | | | | | | | | | | | | | | | | | | | | | | | | | | | | | | | | | | | | VVAGPGDKPMIVVNYKGEEK | | | | | | | | | | | | | | | | | | | | | | | | | | | | | | | | | | | | | | | | | | | | | | | | | | | | | | | | | | | | | | | | | | | | | | | | | | | | | | | | | | | | | | | | | | | | | | | | | | | | | | | | | | | | | | | | | | | | | | | | | | | | | | | | | | | | | | | | | | | | | | | | | | | | | | | | | | | | | | | | | | | | | | | | | | | | | | | | | | | | | | | | | | | |  |  |  |  |  |  |  |  |  |  |  |  |  |  |  |  |
|  | | | | 2129.1191 | | | | | | | | | | | | | | | | | 2129.119 | | | | | | | | | | | | | | | | | | | | | | | | | | | | | | | | 0 | | | | | | | | | | | | | | | | | | | | | | | | | | | | | | | | 0 | | | | | | | | | | | | | | | | | | | | | | | | | | | | | | | | | | 96 | | | | | | | | | | | | | | | | | | | | | | | | | | | | | | | | | | | | | | | | 115 | | | | | | | | | | | | | | | | | | | | | | | | | | | | | | | | | | | | | | | | | | | | | VVAGPGDKPMIVVNYKGEEK | | | | | | | | | | | | | | | | | | | | | | | | | | | | | | | | | | | | | | | | | | | | | | | | | | | | | | | | | | | | | | | | | | | | | | | | | | | | | | | | | | | | | | | | | | | | | | | | | | | | | | | | | | | | | | | | | | | | | | | | | | | | | | | | | | | | | | | | | | | | | | | | | | | | | | | | | | | | | | | | | | | | | | | | | | | | | | | | | | | | | | | | | | | |  |  |  |  |  |  |  |  |  |  |  |  |  |  |  |  |
|  | | | | 2129.1191 | | | | | | | | | | | | | | | | | 2129.119 | | | | | | | | | | | | | | | | | | | | | | | | | | | | | | | | 0 | | | | | | | | | | | | | | | | | | | | | | | | | | | | | | | | 0 | | | | | | | | | | | | | | | | | | | | | | | | | | | | | | | | | | 96 | | | | | | | | | | | | | | | | | | | | | | | | | | | | | | | | | | | | | | | | 115 | | | | | | | | | | | | | | | | | | | | | | | | | | | | | | | | | | | | | | | | | | | | | VVAGPGDKPMIVVNYKGEEK | | | | | | | | | | | | | | | | | | | | | | | | | | | | | | | | | | | | | | | | | | | | | | | | | | | | | | | | | | | | | | | | | | | | | | | | | | | | | | | | | | | | | | | | | | | | | | | | | | | | | | | | | | | | | | | | | | | | | | | | | | | | | | | | | | | | | | | | | | | | | | | | | | | | | | | | | | | | | | | | | | | | | | | | | | | | | | | | | | | | | | | | | | | |  |  |  |  |  |  |  |  |  |  |  |  |  |  |  |  |
|  | | | | 2129.1191 | | | | | | | | | | | | | | | | | 2129.12 | | | | | | | | | | | | | | | | | | | | | | | | | | | | | | | | 0 | | | | | | | | | | | | | | | | | | | | | | | | | | | | | | | | 0 | | | | | | | | | | | | | | | | | | | | | | | | | | | | | | | | | | 96 | | | | | | | | | | | | | | | | | | | | | | | | | | | | | | | | | | | | | | | | 115 | | | | | | | | | | | | | | | | | | | | | | | | | | | | | | | | | | | | | | | | | | | | | VVAGPGDKPMIVVNYKGEEK | | | | | | | | | | | | | | | | | | | | | | | | | | | | | | | | | | | | | | | | | | | | | | | | | | | | | | | | | | | | | | | | | | | | | | | | | | | | | | | | | | | | | | | | | | | | | | | | | | | | | | | | | | | | | | | | | | | | | | | | | | | | | | | | | | | | | | | | | | | | | | | | | | | | | | | | | | | | | | | | | | | | | | | | | | | | | | | | | | | | | | | | | | | |  |  |  |  |  |  |  |  |  |  |  |  |  |  |  |  |
|  | | | | 2145.114 | | | | | | | | | | | | | | | | | 2145.11 | | | | | | | | | | | | | | | | | | | | | | | | | | | | | | | | 0 | | | | | | | | | | | | | | | | | | | | | | | | | | | | | | | | -2 | | | | | | | | | | | | | | | | | | | | | | | | | | | | | | | | | | 96 | | | | | | | | | | | | | | | | | | | | | | | | | | | | | | | | | | | | | | | | 115 | | | | | | | | | | | | | | | | | | | | | | | | | | | | | | | | | | | | | | | | | | | | | VVAGPGDKPMIVVNYKGEEK | | | | | | | | | | | | | | | | | | | | | | | | | | | | | | | | | | | | | | | | | | | | | | | | | | | | | | | | | | | | | | | | | | | | | | | | | | | | | | | | | | | | | | | | | | | | | | | | | | | | | | | | | | | | | | | | | | | | | | | | | | | | | | | | | | | | | | | | | | | | | | | | | | | | | | | | | | | | | | | | | | | | | | | | | | | | | | | | | | | | | | | | | | | |  |  |  |  |  |  |  |  |  |  |  |  |  |  |  |  |
|  | | | | 2145.114 | | | | | | | | | | | | | | | | | 2145.113 | | | | | | | | | | | | | | | | | | | | | | | | | | | | | | | | 0 | | | | | | | | | | | | | | | | | | | | | | | | | | | | | | | | -1 | | | | | | | | | | | | | | | | | | | | | | | | | | | | | | | | | | 96 | | | | | | | | | | | | | | | | | | | | | | | | | | | | | | | | | | | | | | | | 115 | | | | | | | | | | | | | | | | | | | | | | | | | | | | | | | | | | | | | | | | | | | | | VVAGPGDKPMIVVNYKGEEK | | | | | | | | | | | | | | | | | | | | | | | | | | | | | | | | | | | | | | | | | | | | | | | | | | | | | | | | | | | | | | | | | | | | | | | | | | | | | | | | | | | | | | | | | | | | | | | | | | | | | | | | | | | | | | | | | | | | | | | | | | | | | | | | | | | | | | | | | | | | | | | | | | | | | | | | | | | | | | | | | | | | | | | | | | | | | | | | | | | | | | | | | | | |  |  |  |  |  |  |  |  |  |  |  |  |  |  |  |  |
|  | | | | 2145.114 | | | | | | | | | | | | | | | | | 2145.113 | | | | | | | | | | | | | | | | | | | | | | | | | | | | | | | | 0 | | | | | | | | | | | | | | | | | | | | | | | | | | | | | | | | -1 | | | | | | | | | | | | | | | | | | | | | | | | | | | | | | | | | | 96 | | | | | | | | | | | | | | | | | | | | | | | | | | | | | | | | | | | | | | | | 115 | | | | | | | | | | | | | | | | | | | | | | | | | | | | | | | | | | | | | | | | | | | | | VVAGPGDKPMIVVNYKGEEK | | | | | | | | | | | | | | | | | | | | | | | | | | | | | | | | | | | | | | | | | | | | | | | | | | | | | | | | | | | | | | | | | | | | | | | | | | | | | | | | | | | | | | | | | | | | | | | | | | | | | | | | | | | | | | | | | | | | | | | | | | | | | | | | | | | | | | | | | | | | | | | | | | | | | | | | | | | | | | | | | | | | | | | | | | | | | | | | | | | | | | | | | | | |  |  |  |  |  |  |  |  |  |  |  |  |  |  |  |  |
|  | | | | 1566.8327 | | | | | | | | | | | | | | | | | 1566.832 | | | | | | | | | | | | | | | | | | | | | | | | | | | | | | | | 0 | | | | | | | | | | | | | | | | | | | | | | | | | | | | | | | | 0 | | | | | | | | | | | | | | | | | | | | | | | | | | | | | | | | | | 116 | | | | | | | | | | | | | | | | | | | | | | | | | | | | | | | | | | | | | | | | 129 | | | | | | | | | | | | | | | | | | | | | | | | | | | | | | | | | | | | | | | | | | | | | KFSAEEISSMVLVK | | | | | | | | | | | | | | | | | | | | | | | | | | | | | | | | | | | | | | | | | | | | | | | | | | | | | | | | | | | | | | | | | | | | | | | | | | | | | | | | | | | | | | | | | | | | | | | | | | | | | | | | | | | | | | | | | | | | | | | | | | | | | | | | | | | | | | | | | | | | | | | | | | | | | | | | | | | | | | | | | | | | | | | | | | | | | | | | | | | | | | | | | | | |  |  |  |  |  |  |  |  |  |  |  |  |  |  |  |  |
|  | | | | 1582.8276 | | | | | | | | | | | | | | | | | 1582.825 | | | | | | | | | | | | | | | | | | | | | | | | | | | | | | | | 0 | | | | | | | | | | | | | | | | | | | | | | | | | | | | | | | | -2 | | | | | | | | | | | | | | | | | | | | | | | | | | | | | | | | | | 116 | | | | | | | | | | | | | | | | | | | | | | | | | | | | | | | | | | | | | | | | 129 | | | | | | | | | | | | | | | | | | | | | | | | | | | | | | | | | | | | | | | | | | | | | KFSAEEISSMVLVK | | | | | | | | | | | | | | | | | | | | | | | | | | | | | | | | | | | | | | | | | | | | | | | | | | | | | | | | | | | | | | | | | | | | | | | | | | | | | | | | | | | | | | | | | | | | | | | | | | | | | | | | | | | | | | | | | | | | | | | | | | | | | | | | | | | | | | | | | | | | | | | | | | | | | | | | | | | | | | | | | | | | | | | | | | | | | | | | | | | | | | | | | | | |  |  |  |  |  |  |  |  |  |  |  |  |  |  |  |  |
|  | | | | 1582.8276 | | | | | | | | | | | | | | | | | 1582.826 | | | | | | | | | | | | | | | | | | | | | | | | | | | | | | | | 0 | | | | | | | | | | | | | | | | | | | | | | | | | | | | | | | | -1 | | | | | | | | | | | | | | | | | | | | | | | | | | | | | | | | | | 116 | | | | | | | | | | | | | | | | | | | | | | | | | | | | | | | | | | | | | | | | 129 | | | | | | | | | | | | | | | | | | | | | | | | | | | | | | | | | | | | | | | | | | | | | KFSAEEISSMVLVK | | | | | | | | | | | | | | | | | | | | | | | | | | | | | | | | | | | | | | | | | | | | | | | | | | | | | | | | | | | | | | | | | | | | | | | | | | | | | | | | | | | | | | | | | | | | | | | | | | | | | | | | | | | | | | | | | | | | | | | | | | | | | | | | | | | | | | | | | | | | | | | | | | | | | | | | | | | | | | | | | | | | | | | | | | | | | | | | | | | | | | | | | | | |  |  |  |  |  |  |  |  |  |  |  |  |  |  |  |  |
|  | | | | 1556.8133 | | | | | | | | | | | | | | | | | 1556.813 | | | | | | | | | | | | | | | | | | | | | | | | | | | | | | | | 0 | | | | | | | | | | | | | | | | | | | | | | | | | | | | | | | | 0 | | | | | | | | | | | | | | | | | | | | | | | | | | | | | | | | | | 130 | | | | | | | | | | | | | | | | | | | | | | | | | | | | | | | | | | | | | | | | 143 | | | | | | | | | | | | | | | | | | | | | | | | | | | | | | | | | | | | | | | | | | | | | MREVAEAFLGHAVK | | | | | | | | | | | | | | | | | | | | | | | | | | | | | | | | | | | | | | | | | | | | | | | | | | | | | | | | | | | | | | | | | | | | | | | | | | | | | | | | | | | | | | | | | | | | | | | | | | | | | | | | | | | | | | | | | | | | | | | | | | | | | | | | | | | | | | | | | | | | | | | | | | | | | | | | | | | | | | | | | | | | | | | | | | | | | | | | | | | | | | | | | | | |  |  |  |  |  |  |  |  |  |  |  |  |  |  |  |  |
|  | | | | 1572.8082 | | | | | | | | | | | | | | | | | 1572.807 | | | | | | | | | | | | | | | | | | | | | | | | | | | | | | | | 0 | | | | | | | | | | | | | | | | | | | | | | | | | | | | | | | | -1 | | | | | | | | | | | | | | | | | | | | | | | | | | | | | | | | | | 130 | | | | | | | | | | | | | | | | | | | | | | | | | | | | | | | | | | | | | | | | 143 | | | | | | | | | | | | | | | | | | | | | | | | | | | | | | | | | | | | | | | | | | | | | MREVAEAFLGHAVK | | | | | | | | | | | | | | | | | | | | | | | | | | | | | | | | | | | | | | | | | | | | | | | | | | | | | | | | | | | | | | | | | | | | | | | | | | | | | | | | | | | | | | | | | | | | | | | | | | | | | | | | | | | | | | | | | | | | | | | | | | | | | | | | | | | | | | | | | | | | | | | | | | | | | | | | | | | | | | | | | | | | | | | | | | | | | | | | | | | | | | | | | | | |  |  |  |  |  |  |  |  |  |  |  |  |  |  |  |  |
|  | | | | 1572.8082 | | | | | | | | | | | | | | | | | 1572.809 | | | | | | | | | | | | | | | | | | | | | | | | | | | | | | | | 0 | | | | | | | | | | | | | | | | | | | | | | | | | | | | | | | | 0 | | | | | | | | | | | | | | | | | | | | | | | | | | | | | | | | | | 130 | | | | | | | | | | | | | | | | | | | | | | | | | | | | | | | | | | | | | | | | 143 | | | | | | | | | | | | | | | | | | | | | | | | | | | | | | | | | | | | | | | | | | | | | MREVAEAFLGHAVK | | | | | | | | | | | | | | | | | | | | | | | | | | | | | | | | | | | | | | | | | | | | | | | | | | | | | | | | | | | | | | | | | | | | | | | | | | | | | | | | | | | | | | | | | | | | | | | | | | | | | | | | | | | | | | | | | | | | | | | | | | | | | | | | | | | | | | | | | | | | | | | | | | | | | | | | | | | | | | | | | | | | | | | | | | | | | | | | | | | | | | | | | | | |  |  |  |  |  |  |  |  |  |  |  |  |  |  |  |  |
|  | | | | 1572.8082 | | | | | | | | | | | | | | | | | 1572.81 | | | | | | | | | | | | | | | | | | | | | | | | | | | | | | | | 0 | | | | | | | | | | | | | | | | | | | | | | | | | | | | | | | | 1 | | | | | | | | | | | | | | | | | | | | | | | | | | | | | | | | | | 130 | | | | | | | | | | | | | | | | | | | | | | | | | | | | | | | | | | | | | | | | 143 | | | | | | | | | | | | | | | | | | | | | | | | | | | | | | | | | | | | | | | | | | | | | MREVAEAFLGHAVK | | | | | | | | | | | | | | | | | | | | | | | | | | | | | | | | | | | | | | | | | | | | | | | | | | | | | | | | | | | | | | | | | | | | | | | | | | | | | | | | | | | | | | | | | | | | | | | | | | | | | | | | | | | | | | | | | | | | | | | | | | | | | | | | | | | | | | | | | | | | | | | | | | | | | | | | | | | | | | | | | | | | | | | | | | | | | | | | | | | | | | | | | | | |  |  |  |  |  |  |  |  |  |  |  |  |  |  |  |  |
|  | | | | 1572.8082 | | | | | | | | | | | | | | | | | 1572.811 | | | | | | | | | | | | | | | | | | | | | | | | | | | | | | | | 0 | | | | | | | | | | | | | | | | | | | | | | | | | | | | | | | | 2 | | | | | | | | | | | | | | | | | | | | | | | | | | | | | | | | | | 130 | | | | | | | | | | | | | | | | | | | | | | | | | | | | | | | | | | | | | | | | 143 | | | | | | | | | | | | | | | | | | | | | | | | | | | | | | | | | | | | | | | | | | | | | MREVAEAFLGHAVK | | | | | | | | | | | | | | | | | | | | | | | | | | | | | | | | | | | | | | | | | | | | | | | | | | | | | | | | | | | | | | | | | | | | | | | | | | | | | | | | | | | | | | | | | | | | | | | | | | | | | | | | | | | | | | | | | | | | | | | | | | | | | | | | | | | | | | | | | | | | | | | | | | | | | | | | | | | | | | | | | | | | | | | | | | | | | | | | | | | | | | | | | | | |  |  |  |  |  |  |  |  |  |  |  |  |  |  |  |  |
|  | | | | 1269.6717 | | | | | | | | | | | | | | | | | 1269.666 | | | | | | | | | | | | | | | | | | | | | | | | | | | | | | | | 0 | | | | | | | | | | | | | | | | | | | | | | | | | | | | | | | | -4 | | | | | | | | | | | | | | | | | | | | | | | | | | | | | | | | | | 132 | | | | | | | | | | | | | | | | | | | | | | | | | | | | | | | | | | | | | | | | 143 | | | | | | | | | | | | | | | | | | | | | | | | | | | | | | | | | | | | | | | | | | | | | EVAEAFLGHAVK | | | | | | | | | | | | | | | | | | | | | | | | | | | | | | | | | | | | | | | | | | | | | | | | | | | | | | | | | | | | | | | | | | | | | | | | | | | | | | | | | | | | | | | | | | | | | | | | | | | | | | | | | | | | | | | | | | | | | | | | | | | | | | | | | | | | | | | | | | | | | | | | | | | | | | | | | | | | | | | | | | | | | | | | | | | | | | | | | | | | | | | | | | | |  |  |  |  |  |  |  |  |  |  |  |  |  |  |  |  |
|  | | | | 1269.6717 | | | | | | | | | | | | | | | | | 1269.67 | | | | | | | | | | | | | | | | | | | | | | | | | | | | | | | | 0 | | | | | | | | | | | | | | | | | | | | | | | | | | | | | | | | -1 | | | | | | | | | | | | | | | | | | | | | | | | | | | | | | | | | | 132 | | | | | | | | | | | | | | | | | | | | | | | | | | | | | | | | | | | | | | | | 143 | | | | | | | | | | | | | | | | | | | | | | | | | | | | | | | | | | | | | | | | | | | | | EVAEAFLGHAVK | | | | | | | | | | | | | | | | | | | | | | | | | | | | | | | | | | | | | | | | | | | | | | | | | | | | | | | | | | | | | | | | | | | | | | | | | | | | | | | | | | | | | | | | | | | | | | | | | | | | | | | | | | | | | | | | | | | | | | | | | | | | | | | | | | | | | | | | | | | | | | | | | | | | | | | | | | | | | | | | | | | | | | | | | | | | | | | | | | | | | | | | | | | |  |  |  |  |  |  |  |  |  |  |  |  |  |  |  |  |
|  | | | | 1269.6717 | | | | | | | | | | | | | | | | | 1269.67 | | | | | | | | | | | | | | | | | | | | | | | | | | | | | | | | 0 | | | | | | | | | | | | | | | | | | | | | | | | | | | | | | | | -1 | | | | | | | | | | | | | | | | | | | | | | | | | | | | | | | | | | 132 | | | | | | | | | | | | | | | | | | | | | | | | | | | | | | | | | | | | | | | | 143 | | | | | | | | | | | | | | | | | | | | | | | | | | | | | | | | | | | | | | | | | | | | | EVAEAFLGHAVK | | | | | | | | | | | | | | | | | | | | | | | | | | | | | | | | | | | | | | | | | | | | | | | | | | | | | | | | | | | | | | | | | | | | | | | | | | | | | | | | | | | | | | | | | | | | | | | | | | | | | | | | | | | | | | | | | | | | | | | | | | | | | | | | | | | | | | | | | | | | | | | | | | | | | | | | | | | | | | | | | | | | | | | | | | | | | | | | | | | | | | | | | | | |  |  |  |  |  |  |  |  |  |  |  |  |  |  |  |  |
|  | | | | 1269.6717 | | | | | | | | | | | | | | | | | 1269.672 | | | | | | | | | | | | | | | | | | | | | | | | | | | | | | | | 0 | | | | | | | | | | | | | | | | | | | | | | | | | | | | | | | | 0 | | | | | | | | | | | | | | | | | | | | | | | | | | | | | | | | | | 132 | | | | | | | | | | | | | | | | | | | | | | | | | | | | | | | | | | | | | | | | 143 | | | | | | | | | | | | | | | | | | | | | | | | | | | | | | | | | | | | | | | | | | | | | EVAEAFLGHAVK | | | | | | | | | | | | | | | | | | | | | | | | | | | | | | | | | | | | | | | | | | | | | | | | | | | | | | | | | | | | | | | | | | | | | | | | | | | | | | | | | | | | | | | | | | | | | | | | | | | | | | | | | | | | | | | | | | | | | | | | | | | | | | | | | | | | | | | | | | | | | | | | | | | | | | | | | | | | | | | | | | | | | | | | | | | | | | | | | | | | | | | | | | | |  |  |  |  |  |  |  |  |  |  |  |  |  |  |  |  |
|  | | | | 1269.6717 | | | | | | | | | | | | | | | | | 1269.676 | | | | | | | | | | | | | | | | | | | | | | | | | | | | | | | | 0 | | | | | | | | | | | | | | | | | | | | | | | | | | | | | | | | 3 | | | | | | | | | | | | | | | | | | | | | | | | | | | | | | | | | | 132 | | | | | | | | | | | | | | | | | | | | | | | | | | | | | | | | | | | | | | | | 143 | | | | | | | | | | | | | | | | | | | | | | | | | | | | | | | | | | | | | | | | | | | | | EVAEAFLGHAVK | | | | | | | | | | | | | | | | | | | | | | | | | | | | | | | | | | | | | | | | | | | | | | | | | | | | | | | | | | | | | | | | | | | | | | | | | | | | | | | | | | | | | | | | | | | | | | | | | | | | | | | | | | | | | | | | | | | | | | | | | | | | | | | | | | | | | | | | | | | | | | | | | | | | | | | | | | | | | | | | | | | | | | | | | | | | | | | | | | | | | | | | | | | |  |  |  |  |  |  |  |  |  |  |  |  |  |  |  |  |
|  | | | | 1679.8267 | | | | | | | | | | | | | | | | | 1679.824 | | | | | | | | | | | | | | | | | | | | | | | | | | | | | | | | 0 | | | | | | | | | | | | | | | | | | | | | | | | | | | | | | | | -1 | | | | | | | | | | | | | | | | | | | | | | | | | | | | | | | | | | 144 | | | | | | | | | | | | | | | | | | | | | | | | | | | | | | | | | | | | | | | | 158 | | | | | | | | | | | | | | | | | | | | | | | | | | | | | | | | | | | | | | | | | | | | | NAVVTVPAYFNDSQR | | | | | | | | | | | | | | | | | | | | | | | | | | | | | | | | | | | | | | | | | | | | | | | | | | | | | | | | | | | | | | | | | | | | | | | | | | | | | | | | | | | | | | | | | | | | | | | | | | | | | | | | | | | | | | | | | | | | | | | | | | | | | | | | | | | | | | | | | | | | | | | | | | | | | | | | | | | | | | | | | | | | | | | | | | | | | | | | | | | | | | | | | | | |  |  |  |  |  |  |  |  |  |  |  |  |  |  |  |  |
|  | | | | 1679.8267 | | | | | | | | | | | | | | | | | 1679.825 | | | | | | | | | | | | | | | | | | | | | | | | | | | | | | | | 0 | | | | | | | | | | | | | | | | | | | | | | | | | | | | | | | | -1 | | | | | | | | | | | | | | | | | | | | | | | | | | | | | | | | | | 144 | | | | | | | | | | | | | | | | | | | | | | | | | | | | | | | | | | | | | | | | 158 | | | | | | | | | | | | | | | | | | | | | | | | | | | | | | | | | | | | | | | | | | | | | NAVVTVPAYFNDSQR | | | | | | | | | | | | | | | | | | | | | | | | | | | | | | | | | | | | | | | | | | | | | | | | | | | | | | | | | | | | | | | | | | | | | | | | | | | | | | | | | | | | | | | | | | | | | | | | | | | | | | | | | | | | | | | | | | | | | | | | | | | | | | | | | | | | | | | | | | | | | | | | | | | | | | | | | | | | | | | | | | | | | | | | | | | | | | | | | | | | | | | | | | | |  |  |  |  |  |  |  |  |  |  |  |  |  |  |  |  |
|  | | | | 1679.8267 | | | | | | | | | | | | | | | | | 1679.826 | | | | | | | | | | | | | | | | | | | | | | | | | | | | | | | | 0 | | | | | | | | | | | | | | | | | | | | | | | | | | | | | | | | -1 | | | | | | | | | | | | | | | | | | | | | | | | | | | | | | | | | | 144 | | | | | | | | | | | | | | | | | | | | | | | | | | | | | | | | | | | | | | | | 158 | | | | | | | | | | | | | | | | | | | | | | | | | | | | | | | | | | | | | | | | | | | | | NAVVTVPAYFNDSQR | | | | | | | | | | | | | | | | | | | | | | | | | | | | | | | | | | | | | | | | | | | | | | | | | | | | | | | | | | | | | | | | | | | | | | | | | | | | | | | | | | | | | | | | | | | | | | | | | | | | | | | | | | | | | | | | | | | | | | | | | | | | | | | | | | | | | | | | | | | | | | | | | | | | | | | | | | | | | | | | | | | | | | | | | | | | | | | | | | | | | | | | | | | |  |  |  |  |  |  |  |  |  |  |  |  |  |  |  |  |
|  | | | | 1679.8267 | | | | | | | | | | | | | | | | | 1679.827 | | | | | | | | | | | | | | | | | | | | | | | | | | | | | | | | 0 | | | | | | | | | | | | | | | | | | | | | | | | | | | | | | | | 0 | | | | | | | | | | | | | | | | | | | | | | | | | | | | | | | | | | 144 | | | | | | | | | | | | | | | | | | | | | | | | | | | | | | | | | | | | | | | | 158 | | | | | | | | | | | | | | | | | | | | | | | | | | | | | | | | | | | | | | | | | | | | | NAVVTVPAYFNDSQR | | | | | | | | | | | | | | | | | | | | | | | | | | | | | | | | | | | | | | | | | | | | | | | | | | | | | | | | | | | | | | | | | | | | | | | | | | | | | | | | | | | | | | | | | | | | | | | | | | | | | | | | | | | | | | | | | | | | | | | | | | | | | | | | | | | | | | | | | | | | | | | | | | | | | | | | | | | | | | | | | | | | | | | | | | | | | | | | | | | | | | | | | | | |  |  |  |  |  |  |  |  |  |  |  |  |  |  |  |  |
|  | | | | 1679.8267 | | | | | | | | | | | | | | | | | 1679.828 | | | | | | | | | | | | | | | | | | | | | | | | | | | | | | | | 0 | | | | | | | | | | | | | | | | | | | | | | | | | | | | | | | | 1 | | | | | | | | | | | | | | | | | | | | | | | | | | | | | | | | | | 144 | | | | | | | | | | | | | | | | | | | | | | | | | | | | | | | | | | | | | | | | 158 | | | | | | | | | | | | | | | | | | | | | | | | | | | | | | | | | | | | | | | | | | | | | NAVVTVPAYFNDSQR | | | | | | | | | | | | | | | | | | | | | | | | | | | | | | | | | | | | | | | | | | | | | | | | | | | | | | | | | | | | | | | | | | | | | | | | | | | | | | | | | | | | | | | | | | | | | | | | | | | | | | | | | | | | | | | | | | | | | | | | | | | | | | | | | | | | | | | | | | | | | | | | | | | | | | | | | | | | | | | | | | | | | | | | | | | | | | | | | | | | | | | | | | | |  |  |  |  |  |  |  |  |  |  |  |  |  |  |  |  |
|  | | | | 1184.6513 | | | | | | | | | | | | | | | | | 1184.65 | | | | | | | | | | | | | | | | | | | | | | | | | | | | | | | | 0 | | | | | | | | | | | | | | | | | | | | | | | | | | | | | | | | -1 | | | | | | | | | | | | | | | | | | | | | | | | | | | | | | | | | | 163 | | | | | | | | | | | | | | | | | | | | | | | | | | | | | | | | | | | | | | | | 174 | | | | | | | | | | | | | | | | | | | | | | | | | | | | | | | | | | | | | | | | | | | | | DAGAISGLNVLR | | | | | | | | | | | | | | | | | | | | | | | | | | | | | | | | | | | | | | | | | | | | | | | | | | | | | | | | | | | | | | | | | | | | | | | | | | | | | | | | | | | | | | | | | | | | | | | | | | | | | | | | | | | | | | | | | | | | | | | | | | | | | | | | | | | | | | | | | | | | | | | | | | | | | | | | | | | | | | | | | | | | | | | | | | | | | | | | | | | | | | | | | | | |  |  |  |  |  |  |  |  |  |  |  |  |  |  |  |  |
|  | | | | 1184.6513 | | | | | | | | | | | | | | | | | 1184.652 | | | | | | | | | | | | | | | | | | | | | | | | | | | | | | | | 0 | | | | | | | | | | | | | | | | | | | | | | | | | | | | | | | | 0 | | | | | | | | | | | | | | | | | | | | | | | | | | | | | | | | | | 163 | | | | | | | | | | | | | | | | | | | | | | | | | | | | | | | | | | | | | | | | 174 | | | | | | | | | | | | | | | | | | | | | | | | | | | | | | | | | | | | | | | | | | | | | DAGAISGLNVLR | | | | | | | | | | | | | | | | | | | | | | | | | | | | | | | | | | | | | | | | | | | | | | | | | | | | | | | | | | | | | | | | | | | | | | | | | | | | | | | | | | | | | | | | | | | | | | | | | | | | | | | | | | | | | | | | | | | | | | | | | | | | | | | | | | | | | | | | | | | | | | | | | | | | | | | | | | | | | | | | | | | | | | | | | | | | | | | | | | | | | | | | | | | |  |  |  |  |  |  |  |  |  |  |  |  |  |  |  |  |
|  | | | | 1184.6513 | | | | | | | | | | | | | | | | | 1184.652 | | | | | | | | | | | | | | | | | | | | | | | | | | | | | | | | 0 | | | | | | | | | | | | | | | | | | | | | | | | | | | | | | | | 0 | | | | | | | | | | | | | | | | | | | | | | | | | | | | | | | | | | 163 | | | | | | | | | | | | | | | | | | | | | | | | | | | | | | | | | | | | | | | | 174 | | | | | | | | | | | | | | | | | | | | | | | | | | | | | | | | | | | | | | | | | | | | | DAGAISGLNVLR | | | | | | | | | | | | | | | | | | | | | | | | | | | | | | | | | | | | | | | | | | | | | | | | | | | | | | | | | | | | | | | | | | | | | | | | | | | | | | | | | | | | | | | | | | | | | | | | | | | | | | | | | | | | | | | | | | | | | | | | | | | | | | | | | | | | | | | | | | | | | | | | | | | | | | | | | | | | | | | | | | | | | | | | | | | | | | | | | | | | | | | | | | | |  |  |  |  |  |  |  |  |  |  |  |  |  |  |  |  |
|  | | | | 1184.6513 | | | | | | | | | | | | | | | | | 1184.652 | | | | | | | | | | | | | | | | | | | | | | | | | | | | | | | | 0 | | | | | | | | | | | | | | | | | | | | | | | | | | | | | | | | 0 | | | | | | | | | | | | | | | | | | | | | | | | | | | | | | | | | | 163 | | | | | | | | | | | | | | | | | | | | | | | | | | | | | | | | | | | | | | | | 174 | | | | | | | | | | | | | | | | | | | | | | | | | | | | | | | | | | | | | | | | | | | | | DAGAISGLNVLR | | | | | | | | | | | | | | | | | | | | | | | | | | | | | | | | | | | | | | | | | | | | | | | | | | | | | | | | | | | | | | | | | | | | | | | | | | | | | | | | | | | | | | | | | | | | | | | | | | | | | | | | | | | | | | | | | | | | | | | | | | | | | | | | | | | | | | | | | | | | | | | | | | | | | | | | | | | | | | | | | | | | | | | | | | | | | | | | | | | | | | | | | | | |  |  |  |  |  |  |  |  |  |  |  |  |  |  |  |  |
|  | | | | 1184.6513 | | | | | | | | | | | | | | | | | 1184.652 | | | | | | | | | | | | | | | | | | | | | | | | | | | | | | | | 0 | | | | | | | | | | | | | | | | | | | | | | | | | | | | | | | | 1 | | | | | | | | | | | | | | | | | | | | | | | | | | | | | | | | | | 163 | | | | | | | | | | | | | | | | | | | | | | | | | | | | | | | | | | | | | | | | 174 | | | | | | | | | | | | | | | | | | | | | | | | | | | | | | | | | | | | | | | | | | | | | DAGAISGLNVLR | | | | | | | | | | | | | | | | | | | | | | | | | | | | | | | | | | | | | | | | | | | | | | | | | | | | | | | | | | | | | | | | | | | | | | | | | | | | | | | | | | | | | | | | | | | | | | | | | | | | | | | | | | | | | | | | | | | | | | | | | | | | | | | | | | | | | | | | | | | | | | | | | | | | | | | | | | | | | | | | | | | | | | | | | | | | | | | | | | | | | | | | | | | |  |  |  |  |  |  |  |  |  |  |  |  |  |  |  |  |
|  | | | | 1658.8879 | | | | | | | | | | | | | | | | | 1658.887 | | | | | | | | | | | | | | | | | | | | | | | | | | | | | | | | 0 | | | | | | | | | | | | | | | | | | | | | | | | | | | | | | | | 0 | | | | | | | | | | | | | | | | | | | | | | | | | | | | | | | | | | 175 | | | | | | | | | | | | | | | | | | | | | | | | | | | | | | | | | | | | | | | | 190 | | | | | | | | | | | | | | | | | | | | | | | | | | | | | | | | | | | | | | | | | | | | | IINEPTAAAIAYGLDK | | | | | | | | | | | | | | | | | | | | | | | | | | | | | | | | | | | | | | | | | | | | | | | | | | | | | | | | | | | | | | | | | | | | | | | | | | | | | | | | | | | | | | | | | | | | | | | | | | | | | | | | | | | | | | | | | | | | | | | | | | | | | | | | | | | | | | | | | | | | | | | | | | | | | | | | | | | | | | | | | | | | | | | | | | | | | | | | | | | | | | | | | | | |  |  |  |  |  |  |  |  |  |  |  |  |  |  |  |  |
|  | | | | 1658.8879 | | | | | | | | | | | | | | | | | 1658.888 | | | | | | | | | | | | | | | | | | | | | | | | | | | | | | | | 0 | | | | | | | | | | | | | | | | | | | | | | | | | | | | | | | | 0 | | | | | | | | | | | | | | | | | | | | | | | | | | | | | | | | | | 175 | | | | | | | | | | | | | | | | | | | | | | | | | | | | | | | | | | | | | | | | 190 | | | | | | | | | | | | | | | | | | | | | | | | | | | | | | | | | | | | | | | | | | | | | IINEPTAAAIAYGLDK | | | | | | | | | | | | | | | | | | | | | | | | | | | | | | | | | | | | | | | | | | | | | | | | | | | | | | | | | | | | | | | | | | | | | | | | | | | | | | | | | | | | | | | | | | | | | | | | | | | | | | | | | | | | | | | | | | | | | | | | | | | | | | | | | | | | | | | | | | | | | | | | | | | | | | | | | | | | | | | | | | | | | | | | | | | | | | | | | | | | | | | | | | | |  |  |  |  |  |  |  |  |  |  |  |  |  |  |  |  |
|  | | | | 1786.9828 | | | | | | | | | | | | | | | | | 1786.982 | | | | | | | | | | | | | | | | | | | | | | | | | | | | | | | | 0 | | | | | | | | | | | | | | | | | | | | | | | | | | | | | | | | -1 | | | | | | | | | | | | | | | | | | | | | | | | | | | | | | | | | | 175 | | | | | | | | | | | | | | | | | | | | | | | | | | | | | | | | | | | | | | | | 191 | | | | | | | | | | | | | | | | | | | | | | | | | | | | | | | | | | | | | | | | | | | | | IINEPTAAAIAYGLDKK | | | | | | | | | | | | | | | | | | | | | | | | | | | | | | | | | | | | | | | | | | | | | | | | | | | | | | | | | | | | | | | | | | | | | | | | | | | | | | | | | | | | | | | | | | | | | | | | | | | | | | | | | | | | | | | | | | | | | | | | | | | | | | | | | | | | | | | | | | | | | | | | | | | | | | | | | | | | | | | | | | | | | | | | | | | | | | | | | | | | | | | | | | | |  |  |  |  |  |  |  |  |  |  |  |  |  |  |  |  |
|  | | | | 1786.9828 | | | | | | | | | | | | | | | | | 1786.982 | | | | | | | | | | | | | | | | | | | | | | | | | | | | | | | | 0 | | | | | | | | | | | | | | | | | | | | | | | | | | | | | | | | -1 | | | | | | | | | | | | | | | | | | | | | | | | | | | | | | | | | | 175 | | | | | | | | | | | | | | | | | | | | | | | | | | | | | | | | | | | | | | | | 191 | | | | | | | | | | | | | | | | | | | | | | | | | | | | | | | | | | | | | | | | | | | | | IINEPTAAAIAYGLDKK | | | | | | | | | | | | | | | | | | | | | | | | | | | | | | | | | | | | | | | | | | | | | | | | | | | | | | | | | | | | | | | | | | | | | | | | | | | | | | | | | | | | | | | | | | | | | | | | | | | | | | | | | | | | | | | | | | | | | | | | | | | | | | | | | | | | | | | | | | | | | | | | | | | | | | | | | | | | | | | | | | | | | | | | | | | | | | | | | | | | | | | | | | | |  |  |  |  |  |  |  |  |  |  |  |  |  |  |  |  |
|  | | | | 1786.9828 | | | | | | | | | | | | | | | | | 1786.982 | | | | | | | | | | | | | | | | | | | | | | | | | | | | | | | | 0 | | | | | | | | | | | | | | | | | | | | | | | | | | | | | | | | -1 | | | | | | | | | | | | | | | | | | | | | | | | | | | | | | | | | | 175 | | | | | | | | | | | | | | | | | | | | | | | | | | | | | | | | | | | | | | | | 191 | | | | | | | | | | | | | | | | | | | | | | | | | | | | | | | | | | | | | | | | | | | | | IINEPTAAAIAYGLDKK | | | | | | | | | | | | | | | | | | | | | | | | | | | | | | | | | | | | | | | | | | | | | | | | | | | | | | | | | | | | | | | | | | | | | | | | | | | | | | | | | | | | | | | | | | | | | | | | | | | | | | | | | | | | | | | | | | | | | | | | | | | | | | | | | | | | | | | | | | | | | | | | | | | | | | | | | | | | | | | | | | | | | | | | | | | | | | | | | | | | | | | | | | | |  |  |  |  |  |  |  |  |  |  |  |  |  |  |  |  |
|  | | | | 1786.9828 | | | | | | | | | | | | | | | | | 1786.982 | | | | | | | | | | | | | | | | | | | | | | | | | | | | | | | | 0 | | | | | | | | | | | | | | | | | | | | | | | | | | | | | | | | 0 | | | | | | | | | | | | | | | | | | | | | | | | | | | | | | | | | | 175 | | | | | | | | | | | | | | | | | | | | | | | | | | | | | | | | | | | | | | | | 191 | | | | | | | | | | | | | | | | | | | | | | | | | | | | | | | | | | | | | | | | | | | | | IINEPTAAAIAYGLDKK | | | | | | | | | | | | | | | | | | | | | | | | | | | | | | | | | | | | | | | | | | | | | | | | | | | | | | | | | | | | | | | | | | | | | | | | | | | | | | | | | | | | | | | | | | | | | | | | | | | | | | | | | | | | | | | | | | | | | | | | | | | | | | | | | | | | | | | | | | | | | | | | | | | | | | | | | | | | | | | | | | | | | | | | | | | | | | | | | | | | | | | | | | | |  |  |  |  |  |  |  |  |  |  |  |  |  |  |  |  |
|  | | | | 1786.9828 | | | | | | | | | | | | | | | | | 1786.983 | | | | | | | | | | | | | | | | | | | | | | | | | | | | | | | | 0 | | | | | | | | | | | | | | | | | | | | | | | | | | | | | | | | 0 | | | | | | | | | | | | | | | | | | | | | | | | | | | | | | | | | | 175 | | | | | | | | | | | | | | | | | | | | | | | | | | | | | | | | | | | | | | | | 191 | | | | | | | | | | | | | | | | | | | | | | | | | | | | | | | | | | | | | | | | | | | | | IINEPTAAAIAYGLDKK | | | | | | | | | | | | | | | | | | | | | | | | | | | | | | | | | | | | | | | | | | | | | | | | | | | | | | | | | | | | | | | | | | | | | | | | | | | | | | | | | | | | | | | | | | | | | | | | | | | | | | | | | | | | | | | | | | | | | | | | | | | | | | | | | | | | | | | | | | | | | | | | | | | | | | | | | | | | | | | | | | | | | | | | | | | | | | | | | | | | | | | | | | | |  |  |  |  |  |  |  |  |  |  |  |  |  |  |  |  |
|  | | | | 1674.7234 | | | | | | | | | | | | | | | | | 1674.722 | | | | | | | | | | | | | | | | | | | | | | | | | | | | | | | | 0 | | | | | | | | | | | | | | | | | | | | | | | | | | | | | | | | -1 | | | | | | | | | | | | | | | | | | | | | | | | | | | | | | | | | | 226 | | | | | | | | | | | | | | | | | | | | | | | | | | | | | | | | | | | | | | | | 241 | | | | | | | | | | | | | | | | | | | | | | | | | | | | | | | | | | | | | | | | | | | | | ATAGDTHLGGEDFDNR | | | | | | | | | | | | | | | | | | | | | | | | | | | | | | | | | | | | | | | | | | | | | | | | | | | | | | | | | | | | | | | | | | | | | | | | | | | | | | | | | | | | | | | | | | | | | | | | | | | | | | | | | | | | | | | | | | | | | | | | | | | | | | | | | | | | | | | | | | | | | | | | | | | | | | | | | | | | | | | | | | | | | | | | | | | | | | | | | | | | | | | | | | | |  |  |  |  |  |  |  |  |  |  |  |  |  |  |  |  |
|  | | | | 1674.7234 | | | | | | | | | | | | | | | | | 1674.726 | | | | | | | | | | | | | | | | | | | | | | | | | | | | | | | | 0 | | | | | | | | | | | | | | | | | | | | | | | | | | | | | | | | 2 | | | | | | | | | | | | | | | | | | | | | | | | | | | | | | | | | | 226 | | | | | | | | | | | | | | | | | | | | | | | | | | | | | | | | | | | | | | | | 241 | | | | | | | | | | | | | | | | | | | | | | | | | | | | | | | | | | | | | | | | | | | | | ATAGDTHLGGEDFDNR | | | | | | | | | | | | | | | | | | | | | | | | | | | | | | | | | | | | | | | | | | | | | | | | | | | | | | | | | | | | | | | | | | | | | | | | | | | | | | | | | | | | | | | | | | | | | | | | | | | | | | | | | | | | | | | | | | | | | | | | | | | | | | | | | | | | | | | | | | | | | | | | | | | | | | | | | | | | | | | | | | | | | | | | | | | | | | | | | | | | | | | | | | | |  |  |  |  |  |  |  |  |  |  |  |  |  |  |  |  |
|  | | | | 1236.5961 | | | | | | | | | | | | | | | | | 1236.595 | | | | | | | | | | | | | | | | | | | | | | | | | | | | | | | | 0 | | | | | | | | | | | | | | | | | | | | | | | | | | | | | | | | -1 | | | | | | | | | | | | | | | | | | | | | | | | | | | | | | | | | | 242 | | | | | | | | | | | | | | | | | | | | | | | | | | | | | | | | | | | | | | | | 251 | | | | | | | | | | | | | | | | | | | | | | | | | | | | | | | | | | | | | | | | | | | | | MVNHFVSEFK | | | | | | | | | | | | | | | | | | | | | | | | | | | | | | | | | | | | | | | | | | | | | | | | | | | | | | | | | | | | | | | | | | | | | | | | | | | | | | | | | | | | | | | | | | | | | | | | | | | | | | | | | | | | | | | | | | | | | | | | | | | | | | | | | | | | | | | | | | | | | | | | | | | | | | | | | | | | | | | | | | | | | | | | | | | | | | | | | | | | | | | | | | | |  |  |  |  |  |  |  |  |  |  |  |  |  |  |  |  |
|  | | | | 1236.5961 | | | | | | | | | | | | | | | | | 1236.595 | | | | | | | | | | | | | | | | | | | | | | | | | | | | | | | | 0 | | | | | | | | | | | | | | | | | | | | | | | | | | | | | | | | -1 | | | | | | | | | | | | | | | | | | | | | | | | | | | | | | | | | | 242 | | | | | | | | | | | | | | | | | | | | | | | | | | | | | | | | | | | | | | | | 251 | | | | | | | | | | | | | | | | | | | | | | | | | | | | | | | | | | | | | | | | | | | | | MVNHFVSEFK | | | | | | | | | | | | | | | | | | | | | | | | | | | | | | | | | | | | | | | | | | | | | | | | | | | | | | | | | | | | | | | | | | | | | | | | | | | | | | | | | | | | | | | | | | | | | | | | | | | | | | | | | | | | | | | | | | | | | | | | | | | | | | | | | | | | | | | | | | | | | | | | | | | | | | | | | | | | | | | | | | | | | | | | | | | | | | | | | | | | | | | | | | | |  |  |  |  |  |  |  |  |  |  |  |  |  |  |  |  |
|  | | | | 1236.5961 | | | | | | | | | | | | | | | | | 1236.597 | | | | | | | | | | | | | | | | | | | | | | | | | | | | | | | | 0 | | | | | | | | | | | | | | | | | | | | | | | | | | | | | | | | 1 | | | | | | | | | | | | | | | | | | | | | | | | | | | | | | | | | | 242 | | | | | | | | | | | | | | | | | | | | | | | | | | | | | | | | | | | | | | | | 251 | | | | | | | | | | | | | | | | | | | | | | | | | | | | | | | | | | | | | | | | | | | | | MVNHFVSEFK | | | | | | | | | | | | | | | | | | | | | | | | | | | | | | | | | | | | | | | | | | | | | | | | | | | | | | | | | | | | | | | | | | | | | | | | | | | | | | | | | | | | | | | | | | | | | | | | | | | | | | | | | | | | | | | | | | | | | | | | | | | | | | | | | | | | | | | | | | | | | | | | | | | | | | | | | | | | | | | | | | | | | | | | | | | | | | | | | | | | | | | | | | | |  |  |  |  |  |  |  |  |  |  |  |  |  |  |  |  |
|  | | | | 1252.591 | | | | | | | | | | | | | | | | | 1252.589 | | | | | | | | | | | | | | | | | | | | | | | | | | | | | | | | 0 | | | | | | | | | | | | | | | | | | | | | | | | | | | | | | | | -2 | | | | | | | | | | | | | | | | | | | | | | | | | | | | | | | | | | 242 | | | | | | | | | | | | | | | | | | | | | | | | | | | | | | | | | | | | | | | | 251 | | | | | | | | | | | | | | | | | | | | | | | | | | | | | | | | | | | | | | | | | | | | | MVNHFVSEFK | | | | | | | | | | | | | | | | | | | | | | | | | | | | | | | | | | | | | | | | | | | | | | | | | | | | | | | | | | | | | | | | | | | | | | | | | | | | | | | | | | | | | | | | | | | | | | | | | | | | | | | | | | | | | | | | | | | | | | | | | | | | | | | | | | | | | | | | | | | | | | | | | | | | | | | | | | | | | | | | | | | | | | | | | | | | | | | | | | | | | | | | | | | |  |  |  |  |  |  |  |  |  |  |  |  |  |  |  |  |
|  | | | | 1252.591 | | | | | | | | | | | | | | | | | 1252.589 | | | | | | | | | | | | | | | | | | | | | | | | | | | | | | | | 0 | | | | | | | | | | | | | | | | | | | | | | | | | | | | | | | | -2 | | | | | | | | | | | | | | | | | | | | | | | | | | | | | | | | | | 242 | | | | | | | | | | | | | | | | | | | | | | | | | | | | | | | | | | | | | | | | 251 | | | | | | | | | | | | | | | | | | | | | | | | | | | | | | | | | | | | | | | | | | | | | MVNHFVSEFK | | | | | | | | | | | | | | | | | | | | | | | | | | | | | | | | | | | | | | | | | | | | | | | | | | | | | | | | | | | | | | | | | | | | | | | | | | | | | | | | | | | | | | | | | | | | | | | | | | | | | | | | | | | | | | | | | | | | | | | | | | | | | | | | | | | | | | | | | | | | | | | | | | | | | | | | | | | | | | | | | | | | | | | | | | | | | | | | | | | | | | | | | | | |  |  |  |  |  |  |  |  |  |  |  |  |  |  |  |  |
|  | | | | 1252.591 | | | | | | | | | | | | | | | | | 1252.59 | | | | | | | | | | | | | | | | | | | | | | | | | | | | | | | | 0 | | | | | | | | | | | | | | | | | | | | | | | | | | | | | | | | -1 | | | | | | | | | | | | | | | | | | | | | | | | | | | | | | | | | | 242 | | | | | | | | | | | | | | | | | | | | | | | | | | | | | | | | | | | | | | | | 251 | | | | | | | | | | | | | | | | | | | | | | | | | | | | | | | | | | | | | | | | | | | | | MVNHFVSEFK | | | | | | | | | | | | | | | | | | | | | | | | | | | | | | | | | | | | | | | | | | | | | | | | | | | | | | | | | | | | | | | | | | | | | | | | | | | | | | | | | | | | | | | | | | | | | | | | | | | | | | | | | | | | | | | | | | | | | | | | | | | | | | | | | | | | | | | | | | | | | | | | | | | | | | | | | | | | | | | | | | | | | | | | | | | | | | | | | | | | | | | | | | | |  |  |  |  |  |  |  |  |  |  |  |  |  |  |  |  |
|  | | | | 1252.591 | | | | | | | | | | | | | | | | | 1252.591 | | | | | | | | | | | | | | | | | | | | | | | | | | | | | | | | 0 | | | | | | | | | | | | | | | | | | | | | | | | | | | | | | | | 0 | | | | | | | | | | | | | | | | | | | | | | | | | | | | | | | | | | 242 | | | | | | | | | | | | | | | | | | | | | | | | | | | | | | | | | | | | | | | | 251 | | | | | | | | | | | | | | | | | | | | | | | | | | | | | | | | | | | | | | | | | | | | | MVNHFVSEFK | | | | | | | | | | | | | | | | | | | | | | | | | | | | | | | | | | | | | | | | | | | | | | | | | | | | | | | | | | | | | | | | | | | | | | | | | | | | | | | | | | | | | | | | | | | | | | | | | | | | | | | | | | | | | | | | | | | | | | | | | | | | | | | | | | | | | | | | | | | | | | | | | | | | | | | | | | | | | | | | | | | | | | | | | | | | | | | | | | | | | | | | | | | |  |  |  |  |  |  |  |  |  |  |  |  |  |  |  |  |
|  | | | | 1252.591 | | | | | | | | | | | | | | | | | 1252.591 | | | | | | | | | | | | | | | | | | | | | | | | | | | | | | | | 0 | | | | | | | | | | | | | | | | | | | | | | | | | | | | | | | | 0 | | | | | | | | | | | | | | | | | | | | | | | | | | | | | | | | | | 242 | | | | | | | | | | | | | | | | | | | | | | | | | | | | | | | | | | | | | | | | 251 | | | | | | | | | | | | | | | | | | | | | | | | | | | | | | | | | | | | | | | | | | | | | MVNHFVSEFK | | | | | | | | | | | | | | | | | | | | | | | | | | | | | | | | | | | | | | | | | | | | | | | | | | | | | | | | | | | | | | | | | | | | | | | | | | | | | | | | | | | | | | | | | | | | | | | | | | | | | | | | | | | | | | | | | | | | | | | | | | | | | | | | | | | | | | | | | | | | | | | | | | | | | | | | | | | | | | | | | | | | | | | | | | | | | | | | | | | | | | | | | | | |  |  |  |  |  |  |  |  |  |  |  |  |  |  |  |  |
|  | | | | 1252.591 | | | | | | | | | | | | | | | | | 1252.591 | | | | | | | | | | | | | | | | | | | | | | | | | | | | | | | | 0 | | | | | | | | | | | | | | | | | | | | | | | | | | | | | | | | 0 | | | | | | | | | | | | | | | | | | | | | | | | | | | | | | | | | | 242 | | | | | | | | | | | | | | | | | | | | | | | | | | | | | | | | | | | | | | | | 251 | | | | | | | | | | | | | | | | | | | | | | | | | | | | | | | | | | | | | | | | | | | | | MVNHFVSEFK | | | | | | | | | | | | | | | | | | | | | | | | | | | | | | | | | | | | | | | | | | | | | | | | | | | | | | | | | | | | | | | | | | | | | | | | | | | | | | | | | | | | | | | | | | | | | | | | | | | | | | | | | | | | | | | | | | | | | | | | | | | | | | | | | | | | | | | | | | | | | | | | | | | | | | | | | | | | | | | | | | | | | | | | | | | | | | | | | | | | | | | | | | | |  |  |  |  |  |  |  |  |  |  |  |  |  |  |  |  |
|  | | | | 1252.591 | | | | | | | | | | | | | | | | | 1252.592 | | | | | | | | | | | | | | | | | | | | | | | | | | | | | | | | 0 | | | | | | | | | | | | | | | | | | | | | | | | | | | | | | | | 1 | | | | | | | | | | | | | | | | | | | | | | | | | | | | | | | | | | 242 | | | | | | | | | | | | | | | | | | | | | | | | | | | | | | | | | | | | | | | | 251 | | | | | | | | | | | | | | | | | | | | | | | | | | | | | | | | | | | | | | | | | | | | | MVNHFVSEFK | | | | | | | | | | | | | | | | | | | | | | | | | | | | | | | | | | | | | | | | | | | | | | | | | | | | | | | | | | | | | | | | | | | | | | | | | | | | | | | | | | | | | | | | | | | | | | | | | | | | | | | | | | | | | | | | | | | | | | | | | | | | | | | | | | | | | | | | | | | | | | | | | | | | | | | | | | | | | | | | | | | | | | | | | | | | | | | | | | | | | | | | | | | |  |  |  |  |  |  |  |  |  |  |  |  |  |  |  |  |
|  | | | | 1252.591 | | | | | | | | | | | | | | | | | 1252.592 | | | | | | | | | | | | | | | | | | | | | | | | | | | | | | | | 0 | | | | | | | | | | | | | | | | | | | | | | | | | | | | | | | | 1 | | | | | | | | | | | | | | | | | | | | | | | | | | | | | | | | | | 242 | | | | | | | | | | | | | | | | | | | | | | | | | | | | | | | | | | | | | | | | 251 | | | | | | | | | | | | | | | | | | | | | | | | | | | | | | | | | | | | | | | | | | | | | MVNHFVSEFK | | | | | | | | | | | | | | | | | | | | | | | | | | | | | | | | | | | | | | | | | | | | | | | | | | | | | | | | | | | | | | | | | | | | | | | | | | | | | | | | | | | | | | | | | | | | | | | | | | | | | | | | | | | | | | | | | | | | | | | | | | | | | | | | | | | | | | | | | | | | | | | | | | | | | | | | | | | | | | | | | | | | | | | | | | | | | | | | | | | | | | | | | | | |  |  |  |  |  |  |  |  |  |  |  |  |  |  |  |  |
|  | | | | 1392.6972 | | | | | | | | | | | | | | | | | 1392.697 | | | | | | | | | | | | | | | | | | | | | | | | | | | | | | | | 0 | | | | | | | | | | | | | | | | | | | | | | | | | | | | | | | | 0 | | | | | | | | | | | | | | | | | | | | | | | | | | | | | | | | | | 242 | | | | | | | | | | | | | | | | | | | | | | | | | | | | | | | | | | | | | | | | 252 | | | | | | | | | | | | | | | | | | | | | | | | | | | | | | | | | | | | | | | | | | | | | MVNHFVSEFKR | | | | | | | | | | | | | | | | | | | | | | | | | | | | | | | | | | | | | | | | | | | | | | | | | | | | | | | | | | | | | | | | | | | | | | | | | | | | | | | | | | | | | | | | | | | | | | | | | | | | | | | | | | | | | | | | | | | | | | | | | | | | | | | | | | | | | | | | | | | | | | | | | | | | | | | | | | | | | | | | | | | | | | | | | | | | | | | | | | | | | | | | | | | |  |  |  |  |  |  |  |  |  |  |  |  |  |  |  |  |
|  | | | | 1408.6922 | | | | | | | | | | | | | | | | | 1408.69 | | | | | | | | | | | | | | | | | | | | | | | | | | | | | | | | 0 | | | | | | | | | | | | | | | | | | | | | | | | | | | | | | | | -2 | | | | | | | | | | | | | | | | | | | | | | | | | | | | | | | | | | 242 | | | | | | | | | | | | | | | | | | | | | | | | | | | | | | | | | | | | | | | | 252 | | | | | | | | | | | | | | | | | | | | | | | | | | | | | | | | | | | | | | | | | | | | | MVNHFVSEFKR | | | | | | | | | | | | | | | | | | | | | | | | | | | | | | | | | | | | | | | | | | | | | | | | | | | | | | | | | | | | | | | | | | | | | | | | | | | | | | | | | | | | | | | | | | | | | | | | | | | | | | | | | | | | | | | | | | | | | | | | | | | | | | | | | | | | | | | | | | | | | | | | | | | | | | | | | | | | | | | | | | | | | | | | | | | | | | | | | | | | | | | | | | | |  |  |  |  |  |  |  |  |  |  |  |  |  |  |  |  |
|  | | | | 1408.6922 | | | | | | | | | | | | | | | | | 1408.692 | | | | | | | | | | | | | | | | | | | | | | | | | | | | | | | | 0 | | | | | | | | | | | | | | | | | | | | | | | | | | | | | | | | 0 | | | | | | | | | | | | | | | | | | | | | | | | | | | | | | | | | | 242 | | | | | | | | | | | | | | | | | | | | | | | | | | | | | | | | | | | | | | | | 252 | | | | | | | | | | | | | | | | | | | | | | | | | | | | | | | | | | | | | | | | | | | | | MVNHFVSEFKR | | | | | | | | | | | | | | | | | | | | | | | | | | | | | | | | | | | | | | | | | | | | | | | | | | | | | | | | | | | | | | | | | | | | | | | | | | | | | | | | | | | | | | | | | | | | | | | | | | | | | | | | | | | | | | | | | | | | | | | | | | | | | | | | | | | | | | | | | | | | | | | | | | | | | | | | | | | | | | | | | | | | | | | | | | | | | | | | | | | | | | | | | | | |  |  |  |  |  |  |  |  |  |  |  |  |  |  |  |  |
|  | | | | 1408.6922 | | | | | | | | | | | | | | | | | 1408.693 | | | | | | | | | | | | | | | | | | | | | | | | | | | | | | | | 0 | | | | | | | | | | | | | | | | | | | | | | | | | | | | | | | | 0 | | | | | | | | | | | | | | | | | | | | | | | | | | | | | | | | | | 242 | | | | | | | | | | | | | | | | | | | | | | | | | | | | | | | | | | | | | | | | 252 | | | | | | | | | | | | | | | | | | | | | | | | | | | | | | | | | | | | | | | | | | | | | MVNHFVSEFKR | | | | | | | | | | | | | | | | | | | | | | | | | | | | | | | | | | | | | | | | | | | | | | | | | | | | | | | | | | | | | | | | | | | | | | | | | | | | | | | | | | | | | | | | | | | | | | | | | | | | | | | | | | | | | | | | | | | | | | | | | | | | | | | | | | | | | | | | | | | | | | | | | | | | | | | | | | | | | | | | | | | | | | | | | | | | | | | | | | | | | | | | | | | |  |  |  |  |  |  |  |  |  |  |  |  |  |  |  |  |
|  | | | | 1557.7068 | | | | | | | | | | | | | | | | | 1557.705 | | | | | | | | | | | | | | | | | | | | | | | | | | | | | | | | 0 | | | | | | | | | | | | | | | | | | | | | | | | | | | | | | | | -1 | | | | | | | | | | | | | | | | | | | | | | | | | | | | | | | | | | 305 | | | | | | | | | | | | | | | | | | | | | | | | | | | | | | | | | | | | | | | | 316 | | | | | | | | | | | | | | | | | | | | | | | | | | | | | | | | | | | | | | | | | | | | | ARFEEMNMDLFR | | | | | | | | | | | | | | | | | | | | | | | | | | | | | | | | | | | | | | | | | | | | | | | | | | | | | | | | | | | | | | | | | | | | | | | | | | | | | | | | | | | | | | | | | | | | | | | | | | | | | | | | | | | | | | | | | | | | | | | | | | | | | | | | | | | | | | | | | | | | | | | | | | | | | | | | | | | | | | | | | | | | | | | | | | | | | | | | | | | | | | | | | | | |  |  |  |  |  |  |  |  |  |  |  |  |  |  |  |  |
|  | | | | 1573.7017 | | | | | | | | | | | | | | | | | 1573.701 | | | | | | | | | | | | | | | | | | | | | | | | | | | | | | | | 0 | | | | | | | | | | | | | | | | | | | | | | | | | | | | | | | | -1 | | | | | | | | | | | | | | | | | | | | | | | | | | | | | | | | | | 305 | | | | | | | | | | | | | | | | | | | | | | | | | | | | | | | | | | | | | | | | 316 | | | | | | | | | | | | | | | | | | | | | | | | | | | | | | | | | | | | | | | | | | | | | ARFEEMNMDLFR | | | | | | | | | | | | | | | | | | | | | | | | | | | | | | | | | | | | | | | | | | | | | | | | | | | | | | | | | | | | | | | | | | | | | | | | | | | | | | | | | | | | | | | | | | | | | | | | | | | | | | | | | | | | | | | | | | | | | | | | | | | | | | | | | | | | | | | | | | | | | | | | | | | | | | | | | | | | | | | | | | | | | | | | | | | | | | | | | | | | | | | | | | | |  |  |  |  |  |  |  |  |  |  |  |  |  |  |  |  |
|  | | | | 1573.7017 | | | | | | | | | | | | | | | | | 1573.703 | | | | | | | | | | | | | | | | | | | | | | | | | | | | | | | | 0 | | | | | | | | | | | | | | | | | | | | | | | | | | | | | | | | 1 | | | | | | | | | | | | | | | | | | | | | | | | | | | | | | | | | | 305 | | | | | | | | | | | | | | | | | | | | | | | | | | | | | | | | | | | | | | | | 316 | | | | | | | | | | | | | | | | | | | | | | | | | | | | | | | | | | | | | | | | | | | | | ARFEEMNMDLFR | | | | | | | | | | | | | | | | | | | | | | | | | | | | | | | | | | | | | | | | | | | | | | | | | | | | | | | | | | | | | | | | | | | | | | | | | | | | | | | | | | | | | | | | | | | | | | | | | | | | | | | | | | | | | | | | | | | | | | | | | | | | | | | | | | | | | | | | | | | | | | | | | | | | | | | | | | | | | | | | | | | | | | | | | | | | | | | | | | | | | | | | | | | |  |  |  |  |  |  |  |  |  |  |  |  |  |  |  |  |
|  | | | | 1589.6966 | | | | | | | | | | | | | | | | | 1589.695 | | | | | | | | | | | | | | | | | | | | | | | | | | | | | | | | 0 | | | | | | | | | | | | | | | | | | | | | | | | | | | | | | | | -1 | | | | | | | | | | | | | | | | | | | | | | | | | | | | | | | | | | 305 | | | | | | | | | | | | | | | | | | | | | | | | | | | | | | | | | | | | | | | | 316 | | | | | | | | | | | | | | | | | | | | | | | | | | | | | | | | | | | | | | | | | | | | | ARFEEMNMDLFR | | | | | | | | | | | | | | | | | | | | | | | | | | | | | | | | | | | | | | | | | | | | | | | | | | | | | | | | | | | | | | | | | | | | | | | | | | | | | | | | | | | | | | | | | | | | | | | | | | | | | | | | | | | | | | | | | | | | | | | | | | | | | | | | | | | | | | | | | | | | | | | | | | | | | | | | | | | | | | | | | | | | | | | | | | | | | | | | | | | | | | | | | | | |  |  |  |  |  |  |  |  |  |  |  |  |  |  |  |  |
|  | | | | 1589.6966 | | | | | | | | | | | | | | | | | 1589.697 | | | | | | | | | | | | | | | | | | | | | | | | | | | | | | | | 0 | | | | | | | | | | | | | | | | | | | | | | | | | | | | | | | | 0 | | | | | | | | | | | | | | | | | | | | | | | | | | | | | | | | | | 305 | | | | | | | | | | | | | | | | | | | | | | | | | | | | | | | | | | | | | | | | 316 | | | | | | | | | | | | | | | | | | | | | | | | | | | | | | | | | | | | | | | | | | | | | ARFEEMNMDLFR | | | | | | | | | | | | | | | | | | | | | | | | | | | | | | | | | | | | | | | | | | | | | | | | | | | | | | | | | | | | | | | | | | | | | | | | | | | | | | | | | | | | | | | | | | | | | | | | | | | | | | | | | | | | | | | | | | | | | | | | | | | | | | | | | | | | | | | | | | | | | | | | | | | | | | | | | | | | | | | | | | | | | | | | | | | | | | | | | | | | | | | | | | | |  |  |  |  |  |  |  |  |  |  |  |  |  |  |  |  |
|  | | | | 1589.6966 | | | | | | | | | | | | | | | | | 1589.697 | | | | | | | | | | | | | | | | | | | | | | | | | | | | | | | | 0 | | | | | | | | | | | | | | | | | | | | | | | | | | | | | | | | 0 | | | | | | | | | | | | | | | | | | | | | | | | | | | | | | | | | | 305 | | | | | | | | | | | | | | | | | | | | | | | | | | | | | | | | | | | | | | | | 316 | | | | | | | | | | | | | | | | | | | | | | | | | | | | | | | | | | | | | | | | | | | | | ARFEEMNMDLFR | | | | | | | | | | | | | | | | | | | | | | | | | | | | | | | | | | | | | | | | | | | | | | | | | | | | | | | | | | | | | | | | | | | | | | | | | | | | | | | | | | | | | | | | | | | | | | | | | | | | | | | | | | | | | | | | | | | | | | | | | | | | | | | | | | | | | | | | | | | | | | | | | | | | | | | | | | | | | | | | | | | | | | | | | | | | | | | | | | | | | | | | | | | |  |  |  |  |  |  |  |  |  |  |  |  |  |  |  |  |
|  | | | | 1589.6966 | | | | | | | | | | | | | | | | | 1589.697 | | | | | | | | | | | | | | | | | | | | | | | | | | | | | | | | 0 | | | | | | | | | | | | | | | | | | | | | | | | | | | | | | | | 0 | | | | | | | | | | | | | | | | | | | | | | | | | | | | | | | | | | 305 | | | | | | | | | | | | | | | | | | | | | | | | | | | | | | | | | | | | | | | | 316 | | | | | | | | | | | | | | | | | | | | | | | | | | | | | | | | | | | | | | | | | | | | | ARFEEMNMDLFR | | | | | | | | | | | | | | | | | | | | | | | | | | | | | | | | | | | | | | | | | | | | | | | | | | | | | | | | | | | | | | | | | | | | | | | | | | | | | | | | | | | | | | | | | | | | | | | | | | | | | | | | | | | | | | | | | | | | | | | | | | | | | | | | | | | | | | | | | | | | | | | | | | | | | | | | | | | | | | | | | | | | | | | | | | | | | | | | | | | | | | | | | | | |  |  |  |  |  |  |  |  |  |  |  |  |  |  |  |  |
|  | | | | 1362.5584 | | | | | | | | | | | | | | | | | 1362.558 | | | | | | | | | | | | | | | | | | | | | | | | | | | | | | | | 0 | | | | | | | | | | | | | | | | | | | | | | | | | | | | | | | | 0 | | | | | | | | | | | | | | | | | | | | | | | | | | | | | | | | | | 307 | | | | | | | | | | | | | | | | | | | | | | | | | | | | | | | | | | | | | | | | 316 | | | | | | | | | | | | | | | | | | | | | | | | | | | | | | | | | | | | | | | | | | | | | FEEMNMDLFR | | | | | | | | | | | | | | | | | | | | | | | | | | | | | | | | | | | | | | | | | | | | | | | | | | | | | | | | | | | | | | | | | | | | | | | | | | | | | | | | | | | | | | | | | | | | | | | | | | | | | | | | | | | | | | | | | | | | | | | | | | | | | | | | | | | | | | | | | | | | | | | | | | | | | | | | | | | | | | | | | | | | | | | | | | | | | | | | | | | | | | | | | | | |  |  |  |  |  |  |  |  |  |  |  |  |  |  |  |  |
|  | | | | 1458.6635 | | | | | | | | | | | | | | | | | 1458.664 | | | | | | | | | | | | | | | | | | | | | | | | | | | | | | | | 0 | | | | | | | | | | | | | | | | | | | | | | | | | | | | | | | | 0 | | | | | | | | | | | | | | | | | | | | | | | | | | | | | | | | | | 307 | | | | | | | | | | | | | | | | | | | | | | | | | | | | | | | | | | | | | | | | 317 | | | | | | | | | | | | | | | | | | | | | | | | | | | | | | | | | | | | | | | | | | | | | FEEMNMDLFRK | | | | | | | | | | | | | | | | | | | | | | | | | | | | | | | | | | | | | | | | | | | | | | | | | | | | | | | | | | | | | | | | | | | | | | | | | | | | | | | | | | | | | | | | | | | | | | | | | | | | | | | | | | | | | | | | | | | | | | | | | | | | | | | | | | | | | | | | | | | | | | | | | | | | | | | | | | | | | | | | | | | | | | | | | | | | | | | | | | | | | | | | | | | |  |  |  |  |  |  |  |  |  |  |  |  |  |  |  |  |
|  | | | | 1474.6584 | | | | | | | | | | | | | | | | | 1474.66 | | | | | | | | | | | | | | | | | | | | | | | | | | | | | | | | 0 | | | | | | | | | | | | | | | | | | | | | | | | | | | | | | | | 1 | | | | | | | | | | | | | | | | | | | | | | | | | | | | | | | | | | 307 | | | | | | | | | | | | | | | | | | | | | | | | | | | | | | | | | | | | | | | | 317 | | | | | | | | | | | | | | | | | | | | | | | | | | | | | | | | | | | | | | | | | | | | | FEEMNMDLFRK | | | | | | | | | | | | | | | | | | | | | | | | | | | | | | | | | | | | | | | | | | | | | | | | | | | | | | | | | | | | | | | | | | | | | | | | | | | | | | | | | | | | | | | | | | | | | | | | | | | | | | | | | | | | | | | | | | | | | | | | | | | | | | | | | | | | | | | | | | | | | | | | | | | | | | | | | | | | | | | | | | | | | | | | | | | | | | | | | | | | | | | | | | | |  |  |  |  |  |  |  |  |  |  |  |  |  |  |  |  |
|  | | | | 1490.6534 | | | | | | | | | | | | | | | | | 1490.649 | | | | | | | | | | | | | | | | | | | | | | | | | | | | | | | | 0 | | | | | | | | | | | | | | | | | | | | | | | | | | | | | | | | -3 | | | | | | | | | | | | | | | | | | | | | | | | | | | | | | | | | | 307 | | | | | | | | | | | | | | | | | | | | | | | | | | | | | | | | | | | | | | | | 317 | | | | | | | | | | | | | | | | | | | | | | | | | | | | | | | | | | | | | | | | | | | | | FEEMNMDLFRK | | | | | | | | | | | | | | | | | | | | | | | | | | | | | | | | | | | | | | | | | | | | | | | | | | | | | | | | | | | | | | | | | | | | | | | | | | | | | | | | | | | | | | | | | | | | | | | | | | | | | | | | | | | | | | | | | | | | | | | | | | | | | | | | | | | | | | | | | | | | | | | | | | | | | | | | | | | | | | | | | | | | | | | | | | | | | | | | | | | | | | | | | | | |  |  |  |  |  |  |  |  |  |  |  |  |  |  |  |  |
|  | | | | 1822.9901 | | | | | | | | | | | | | | | | | 1822.987 | | | | | | | | | | | | | | | | | | | | | | | | | | | | | | | | 0 | | | | | | | | | | | | | | | | | | | | | | | | | | | | | | | | -1 | | | | | | | | | | | | | | | | | | | | | | | | | | | | | | | | | | 331 | | | | | | | | | | | | | | | | | | | | | | | | | | | | | | | | | | | | | | | | 347 | | | | | | | | | | | | | | | | | | | | | | | | | | | | | | | | | | | | | | | | | | | | | IDKSQVHEVVLVGGSTR | | | | | | | | | | | | | | | | | | | | | | | | | | | | | | | | | | | | | | | | | | | | | | | | | | | | | | | | | | | | | | | | | | | | | | | | | | | | | | | | | | | | | | | | | | | | | | | | | | | | | | | | | | | | | | | | | | | | | | | | | | | | | | | | | | | | | | | | | | | | | | | | | | | | | | | | | | | | | | | | | | | | | | | | | | | | | | | | | | | | | | | | | | | |  |  |  |  |  |  |  |  |  |  |  |  |  |  |  |  |
|  | | | | 1822.9901 | | | | | | | | | | | | | | | | | 1822.988 | | | | | | | | | | | | | | | | | | | | | | | | | | | | | | | | 0 | | | | | | | | | | | | | | | | | | | | | | | | | | | | | | | | -1 | | | | | | | | | | | | | | | | | | | | | | | | | | | | | | | | | | 331 | | | | | | | | | | | | | | | | | | | | | | | | | | | | | | | | | | | | | | | | 347 | | | | | | | | | | | | | | | | | | | | | | | | | | | | | | | | | | | | | | | | | | | | | IDKSQVHEVVLVGGSTR | | | | | | | | | | | | | | | | | | | | | | | | | | | | | | | | | | | | | | | | | | | | | | | | | | | | | | | | | | | | | | | | | | | | | | | | | | | | | | | | | | | | | | | | | | | | | | | | | | | | | | | | | | | | | | | | | | | | | | | | | | | | | | | | | | | | | | | | | | | | | | | | | | | | | | | | | | | | | | | | | | | | | | | | | | | | | | | | | | | | | | | | | | | |  |  |  |  |  |  |  |  |  |  |  |  |  |  |  |  |
|  | | | | 1822.9901 | | | | | | | | | | | | | | | | | 1822.989 | | | | | | | | | | | | | | | | | | | | | | | | | | | | | | | | 0 | | | | | | | | | | | | | | | | | | | | | | | | | | | | | | | | -1 | | | | | | | | | | | | | | | | | | | | | | | | | | | | | | | | | | 331 | | | | | | | | | | | | | | | | | | | | | | | | | | | | | | | | | | | | | | | | 347 | | | | | | | | | | | | | | | | | | | | | | | | | | | | | | | | | | | | | | | | | | | | | IDKSQVHEVVLVGGSTR | | | | | | | | | | | | | | | | | | | | | | | | | | | | | | | | | | | | | | | | | | | | | | | | | | | | | | | | | | | | | | | | | | | | | | | | | | | | | | | | | | | | | | | | | | | | | | | | | | | | | | | | | | | | | | | | | | | | | | | | | | | | | | | | | | | | | | | | | | | | | | | | | | | | | | | | | | | | | | | | | | | | | | | | | | | | | | | | | | | | | | | | | | | |  |  |  |  |  |  |  |  |  |  |  |  |  |  |  |  |
|  | | | | 1822.9901 | | | | | | | | | | | | | | | | | 1822.99 | | | | | | | | | | | | | | | | | | | | | | | | | | | | | | | | 0 | | | | | | | | | | | | | | | | | | | | | | | | | | | | | | | | 0 | | | | | | | | | | | | | | | | | | | | | | | | | | | | | | | | | | 331 | | | | | | | | | | | | | | | | | | | | | | | | | | | | | | | | | | | | | | | | 347 | | | | | | | | | | | | | | | | | | | | | | | | | | | | | | | | | | | | | | | | | | | | | IDKSQVHEVVLVGGSTR | | | | | | | | | | | | | | | | | | | | | | | | | | | | | | | | | | | | | | | | | | | | | | | | | | | | | | | | | | | | | | | | | | | | | | | | | | | | | | | | | | | | | | | | | | | | | | | | | | | | | | | | | | | | | | | | | | | | | | | | | | | | | | | | | | | | | | | | | | | | | | | | | | | | | | | | | | | | | | | | | | | | | | | | | | | | | | | | | | | | | | | | | | | |  |  |  |  |  |  |  |  |  |  |  |  |  |  |  |  |
|  | | | | 1822.9901 | | | | | | | | | | | | | | | | | 1822.99 | | | | | | | | | | | | | | | | | | | | | | | | | | | | | | | | 0 | | | | | | | | | | | | | | | | | | | | | | | | | | | | | | | | 0 | | | | | | | | | | | | | | | | | | | | | | | | | | | | | | | | | | 331 | | | | | | | | | | | | | | | | | | | | | | | | | | | | | | | | | | | | | | | | 347 | | | | | | | | | | | | | | | | | | | | | | | | | | | | | | | | | | | | | | | | | | | | | IDKSQVHEVVLVGGSTR | | | | | | | | | | | | | | | | | | | | | | | | | | | | | | | | | | | | | | | | | | | | | | | | | | | | | | | | | | | | | | | | | | | | | | | | | | | | | | | | | | | | | | | | | | | | | | | | | | | | | | | | | | | | | | | | | | | | | | | | | | | | | | | | | | | | | | | | | | | | | | | | | | | | | | | | | | | | | | | | | | | | | | | | | | | | | | | | | | | | | | | | | | | |  |  |  |  |  |  |  |  |  |  |  |  |  |  |  |  |
|  | | | | 1822.9901 | | | | | | | | | | | | | | | | | 1822.991 | | | | | | | | | | | | | | | | | | | | | | | | | | | | | | | | 0 | | | | | | | | | | | | | | | | | | | | | | | | | | | | | | | | 0 | | | | | | | | | | | | | | | | | | | | | | | | | | | | | | | | | | 331 | | | | | | | | | | | | | | | | | | | | | | | | | | | | | | | | | | | | | | | | 347 | | | | | | | | | | | | | | | | | | | | | | | | | | | | | | | | | | | | | | | | | | | | | IDKSQVHEVVLVGGSTR | | | | | | | | | | | | | | | | | | | | | | | | | | | | | | | | | | | | | | | | | | | | | | | | | | | | | | | | | | | | | | | | | | | | | | | | | | | | | | | | | | | | | | | | | | | | | | | | | | | | | | | | | | | | | | | | | | | | | | | | | | | | | | | | | | | | | | | | | | | | | | | | | | | | | | | | | | | | | | | | | | | | | | | | | | | | | | | | | | | | | | | | | | | |  |  |  |  |  |  |  |  |  |  |  |  |  |  |  |  |
|  | | | | 1822.9901 | | | | | | | | | | | | | | | | | 1822.991 | | | | | | | | | | | | | | | | | | | | | | | | | | | | | | | | 0 | | | | | | | | | | | | | | | | | | | | | | | | | | | | | | | | 0 | | | | | | | | | | | | | | | | | | | | | | | | | | | | | | | | | | 331 | | | | | | | | | | | | | | | | | | | | | | | | | | | | | | | | | | | | | | | | 347 | | | | | | | | | | | | | | | | | | | | | | | | | | | | | | | | | | | | | | | | | | | | | IDKSQVHEVVLVGGSTR | | | | | | | | | | | | | | | | | | | | | | | | | | | | | | | | | | | | | | | | | | | | | | | | | | | | | | | | | | | | | | | | | | | | | | | | | | | | | | | | | | | | | | | | | | | | | | | | | | | | | | | | | | | | | | | | | | | | | | | | | | | | | | | | | | | | | | | | | | | | | | | | | | | | | | | | | | | | | | | | | | | | | | | | | | | | | | | | | | | | | | | | | | | |  |  |  |  |  |  |  |  |  |  |  |  |  |  |  |  |
|  | | | | 1466.7842 | | | | | | | | | | | | | | | | | 1466.782 | | | | | | | | | | | | | | | | | | | | | | | | | | | | | | | | 0 | | | | | | | | | | | | | | | | | | | | | | | | | | | | | | | | -1 | | | | | | | | | | | | | | | | | | | | | | | | | | | | | | | | | | 334 | | | | | | | | | | | | | | | | | | | | | | | | | | | | | | | | | | | | | | | | 347 | | | | | | | | | | | | | | | | | | | | | | | | | | | | | | | | | | | | | | | | | | | | | SQVHEVVLVGGSTR | | | | | | | | | | | | | | | | | | | | | | | | | | | | | | | | | | | | | | | | | | | | | | | | | | | | | | | | | | | | | | | | | | | | | | | | | | | | | | | | | | | | | | | | | | | | | | | | | | | | | | | | | | | | | | | | | | | | | | | | | | | | | | | | | | | | | | | | | | | | | | | | | | | | | | | | | | | | | | | | | | | | | | | | | | | | | | | | | | | | | | | | | | | |  |  |  |  |  |  |  |  |  |  |  |  |  |  |  |  |
|  | | | | 1466.7842 | | | | | | | | | | | | | | | | | 1466.783 | | | | | | | | | | | | | | | | | | | | | | | | | | | | | | | | 0 | | | | | | | | | | | | | | | | | | | | | | | | | | | | | | | | -1 | | | | | | | | | | | | | | | | | | | | | | | | | | | | | | | | | | 334 | | | | | | | | | | | | | | | | | | | | | | | | | | | | | | | | | | | | | | | | 347 | | | | | | | | | | | | | | | | | | | | | | | | | | | | | | | | | | | | | | | | | | | | | SQVHEVVLVGGSTR | | | | | | | | | | | | | | | | | | | | | | | | | | | | | | | | | | | | | | | | | | | | | | | | | | | | | | | | | | | | | | | | | | | | | | | | | | | | | | | | | | | | | | | | | | | | | | | | | | | | | | | | | | | | | | | | | | | | | | | | | | | | | | | | | | | | | | | | | | | | | | | | | | | | | | | | | | | | | | | | | | | | | | | | | | | | | | | | | | | | | | | | | | | |  |  |  |  |  |  |  |  |  |  |  |  |  |  |  |  |
|  | | | | 1466.7842 | | | | | | | | | | | | | | | | | 1466.785 | | | | | | | | | | | | | | | | | | | | | | | | | | | | | | | | 0 | | | | | | | | | | | | | | | | | | | | | | | | | | | | | | | | 0 | | | | | | | | | | | | | | | | | | | | | | | | | | | | | | | | | | 334 | | | | | | | | | | | | | | | | | | | | | | | | | | | | | | | | | | | | | | | | 347 | | | | | | | | | | | | | | | | | | | | | | | | | | | | | | | | | | | | | | | | | | | | | SQVHEVVLVGGSTR | | | | | | | | | | | | | | | | | | | | | | | | | | | | | | | | | | | | | | | | | | | | | | | | | | | | | | | | | | | | | | | | | | | | | | | | | | | | | | | | | | | | | | | | | | | | | | | | | | | | | | | | | | | | | | | | | | | | | | | | | | | | | | | | | | | | | | | | | | | | | | | | | | | | | | | | | | | | | | | | | | | | | | | | | | | | | | | | | | | | | | | | | | | |  |  |  |  |  |  |  |  |  |  |  |  |  |  |  |  |
|  | | | | 1466.7842 | | | | | | | | | | | | | | | | | 1466.785 | | | | | | | | | | | | | | | | | | | | | | | | | | | | | | | | 0 | | | | | | | | | | | | | | | | | | | | | | | | | | | | | | | | 1 | | | | | | | | | | | | | | | | | | | | | | | | | | | | | | | | | | 334 | | | | | | | | | | | | | | | | | | | | | | | | | | | | | | | | | | | | | | | | 347 | | | | | | | | | | | | | | | | | | | | | | | | | | | | | | | | | | | | | | | | | | | | | SQVHEVVLVGGSTR | | | | | | | | | | | | | | | | | | | | | | | | | | | | | | | | | | | | | | | | | | | | | | | | | | | | | | | | | | | | | | | | | | | | | | | | | | | | | | | | | | | | | | | | | | | | | | | | | | | | | | | | | | | | | | | | | | | | | | | | | | | | | | | | | | | | | | | | | | | | | | | | | | | | | | | | | | | | | | | | | | | | | | | | | | | | | | | | | | | | | | | | | | | |  |  |  |  |  |  |  |  |  |  |  |  |  |  |  |  |
|  | | | | 1466.7842 | | | | | | | | | | | | | | | | | 1466.79 | | | | | | | | | | | | | | | | | | | | | | | | | | | | | | | | 0 | | | | | | | | | | | | | | | | | | | | | | | | | | | | | | | | 4 | | | | | | | | | | | | | | | | | | | | | | | | | | | | | | | | | | 334 | | | | | | | | | | | | | | | | | | | | | | | | | | | | | | | | | | | | | | | | 347 | | | | | | | | | | | | | | | | | | | | | | | | | | | | | | | | | | | | | | | | | | | | | SQVHEVVLVGGSTR | | | | | | | | | | | | | | | | | | | | | | | | | | | | | | | | | | | | | | | | | | | | | | | | | | | | | | | | | | | | | | | | | | | | | | | | | | | | | | | | | | | | | | | | | | | | | | | | | | | | | | | | | | | | | | | | | | | | | | | | | | | | | | | | | | | | | | | | | | | | | | | | | | | | | | | | | | | | | | | | | | | | | | | | | | | | | | | | | | | | | | | | | | | |  |  |  |  |  |  |  |  |  |  |  |  |  |  |  |  |
|  | | | | 1435.746 | | | | | | | | | | | | | | | | | 1435.745 | | | | | | | | | | | | | | | | | | | | | | | | | | | | | | | | 0 | | | | | | | | | | | | | | | | | | | | | | | | | | | | | | | | -1 | | | | | | | | | | | | | | | | | | | | | | | | | | | | | | | | | | 351 | | | | | | | | | | | | | | | | | | | | | | | | | | | | | | | | | | | | | | | | 362 | | | | | | | | | | | | | | | | | | | | | | | | | | | | | | | | | | | | | | | | | | | | | VQQLLQDFFNGK | | | | | | | | | | | | | | | | | | | | | | | | | | | | | | | | | | | | | | | | | | | | | | | | | | | | | | | | | | | | | | | | | | | | | | | | | | | | | | | | | | | | | | | | | | | | | | | | | | | | | | | | | | | | | | | | | | | | | | | | | | | | | | | | | | | | | | | | | | | | | | | | | | | | | | | | | | | | | | | | | | | | | | | | | | | | | | | | | | | | | | | | | | | |  |  |  |  |  |  |  |  |  |  |  |  |  |  |  |  |
|  | | | | 1435.746 | | | | | | | | | | | | | | | | | 1435.747 | | | | | | | | | | | | | | | | | | | | | | | | | | | | | | | | 0 | | | | | | | | | | | | | | | | | | | | | | | | | | | | | | | | 1 | | | | | | | | | | | | | | | | | | | | | | | | | | | | | | | | | | 351 | | | | | | | | | | | | | | | | | | | | | | | | | | | | | | | | | | | | | | | | 362 | | | | | | | | | | | | | | | | | | | | | | | | | | | | | | | | | | | | | | | | | | | | | VQQLLQDFFNGK | | | | | | | | | | | | | | | | | | | | | | | | | | | | | | | | | | | | | | | | | | | | | | | | | | | | | | | | | | | | | | | | | | | | | | | | | | | | | | | | | | | | | | | | | | | | | | | | | | | | | | | | | | | | | | | | | | | | | | | | | | | | | | | | | | | | | | | | | | | | | | | | | | | | | | | | | | | | | | | | | | | | | | | | | | | | | | | | | | | | | | | | | | | |  |  |  |  |  |  |  |  |  |  |  |  |  |  |  |  |
|  | | | | 1373.6033 | | | | | | | | | | | | | | | | | 1373.601 | | | | | | | | | | | | | | | | | | | | | | | | | | | | | | | | 0 | | | | | | | | | | | | | | | | | | | | | | | | | | | | | | | | -1 | | | | | | | | | | | | | | | | | | | | | | | | | | | | | | | | | | 545 | | | | | | | | | | | | | | | | | | | | | | | | | | | | | | | | | | | | | | | | 555 | | | | | | | | | | | | | | | | | | | | | | | | | | | | | | | | | | | | | | | | | | | | | NSLENYAYNMR | | | | | | | | | | | | | | | | | | | | | | | | | | | | | | | | | | | | | | | | | | | | | | | | | | | | | | | | | | | | | | | | | | | | | | | | | | | | | | | | | | | | | | | | | | | | | | | | | | | | | | | | | | | | | | | | | | | | | | | | | | | | | | | | | | | | | | | | | | | | | | | | | | | | | | | | | | | | | | | | | | | | | | | | | | | | | | | | | | | | | | | | | | | |  |  |  |  |  |  |  |  |  |  |  |  |  |  |  |  |
|  | | | | 1373.6033 | | | | | | | | | | | | | | | | | 1373.602 | | | | | | | | | | | | | | | | | | | | | | | | | | | | | | | | 0 | | | | | | | | | | | | | | | | | | | | | | | | | | | | | | | | -1 | | | | | | | | | | | | | | | | | | | | | | | | | | | | | | | | | | 545 | | | | | | | | | | | | | | | | | | | | | | | | | | | | | | | | | | | | | | | | 555 | | | | | | | | | | | | | | | | | | | | | | | | | | | | | | | | | | | | | | | | | | | | | NSLENYAYNMR | | | | | | | | | | | | | | | | | | | | | | | | | | | | | | | | | | | | | | | | | | | | | | | | | | | | | | | | | | | | | | | | | | | | | | | | | | | | | | | | | | | | | | | | | | | | | | | | | | | | | | | | | | | | | | | | | | | | | | | | | | | | | | | | | | | | | | | | | | | | | | | | | | | | | | | | | | | | | | | | | | | | | | | | | | | | | | | | | | | | | | | | | | | |  |  |  |  |  |  |  |  |  |  |  |  |  |  |  |  |
|  | | | | 1373.6033 | | | | | | | | | | | | | | | | | 1373.602 | | | | | | | | | | | | | | | | | | | | | | | | | | | | | | | | 0 | | | | | | | | | | | | | | | | | | | | | | | | | | | | | | | | -1 | | | | | | | | | | | | | | | | | | | | | | | | | | | | | | | | | | 545 | | | | | | | | | | | | | | | | | | | | | | | | | | | | | | | | | | | | | | | | 555 | | | | | | | | | | | | | | | | | | | | | | | | | | | | | | | | | | | | | | | | | | | | | NSLENYAYNMR | | | | | | | | | | | | | | | | | | | | | | | | | | | | | | | | | | | | | | | | | | | | | | | | | | | | | | | | | | | | | | | | | | | | | | | | | | | | | | | | | | | | | | | | | | | | | | | | | | | | | | | | | | | | | | | | | | | | | | | | | | | | | | | | | | | | | | | | | | | | | | | | | | | | | | | | | | | | | | | | | | | | | | | | | | | | | | | | | | | | | | | | | | | |  |  |  |  |  |  |  |  |  |  |  |  |  |  |  |  |
|  | | | | 1373.6033 | | | | | | | | | | | | | | | | | 1373.604 | | | | | | | | | | | | | | | | | | | | | | | | | | | | | | | | 0 | | | | | | | | | | | | | | | | | | | | | | | | | | | | | | | | 0 | | | | | | | | | | | | | | | | | | | | | | | | | | | | | | | | | | 545 | | | | | | | | | | | | | | | | | | | | | | | | | | | | | | | | | | | | | | | | 555 | | | | | | | | | | | | | | | | | | | | | | | | | | | | | | | | | | | | | | | | | | | | | NSLENYAYNMR | | | | | | | | | | | | | | | | | | | | | | | | | | | | | | | | | | | | | | | | | | | | | | | | | | | | | | | | | | | | | | | | | | | | | | | | | | | | | | | | | | | | | | | | | | | | | | | | | | | | | | | | | | | | | | | | | | | | | | | | | | | | | | | | | | | | | | | | | | | | | | | | | | | | | | | | | | | | | | | | | | | | | | | | | | | | | | | | | | | | | | | | | | | |  |  |  |  |  |  |  |  |  |  |  |  |  |  |  |  |
|  | | | | 1373.6033 | | | | | | | | | | | | | | | | | 1373.604 | | | | | | | | | | | | | | | | | | | | | | | | | | | | | | | | 0 | | | | | | | | | | | | | | | | | | | | | | | | | | | | | | | | 0 | | | | | | | | | | | | | | | | | | | | | | | | | | | | | | | | | | 545 | | | | | | | | | | | | | | | | | | | | | | | | | | | | | | | | | | | | | | | | 555 | | | | | | | | | | | | | | | | | | | | | | | | | | | | | | | | | | | | | | | | | | | | | NSLENYAYNMR | | | | | | | | | | | | | | | | | | | | | | | | | | | | | | | | | | | | | | | | | | | | | | | | | | | | | | | | | | | | | | | | | | | | | | | | | | | | | | | | | | | | | | | | | | | | | | | | | | | | | | | | | | | | | | | | | | | | | | | | | | | | | | | | | | | | | | | | | | | | | | | | | | | | | | | | | | | | | | | | | | | | | | | | | | | | | | | | | | | | | | | | | | | |  |  |  |  |  |  |  |  |  |  |  |  |  |  |  |  |
|  | | | | 1389.5983 | | | | | | | | | | | | | | | | | 1389.596 | | | | | | | | | | | | | | | | | | | | | | | | | | | | | | | | 0 | | | | | | | | | | | | | | | | | | | | | | | | | | | | | | | | -1 | | | | | | | | | | | | | | | | | | | | | | | | | | | | | | | | | | 545 | | | | | | | | | | | | | | | | | | | | | | | | | | | | | | | | | | | | | | | | 555 | | | | | | | | | | | | | | | | | | | | | | | | | | | | | | | | | | | | | | | | | | | | | NSLENYAYNMR | | | | | | | | | | | | | | | | | | | | | | | | | | | | | | | | | | | | | | | | | | | | | | | | | | | | | | | | | | | | | | | | | | | | | | | | | | | | | | | | | | | | | | | | | | | | | | | | | | | | | | | | | | | | | | | | | | | | | | | | | | | | | | | | | | | | | | | | | | | | | | | | | | | | | | | | | | | | | | | | | | | | | | | | | | | | | | | | | | | | | | | | | | | |  |  |  |  |  |  |  |  |  |  |  |  |  |  |  |  |
|  | | | | 1389.5983 | | | | | | | | | | | | | | | | | 1389.597 | | | | | | | | | | | | | | | | | | | | | | | | | | | | | | | | 0 | | | | | | | | | | | | | | | | | | | | | | | | | | | | | | | | -1 | | | | | | | | | | | | | | | | | | | | | | | | | | | | | | | | | | 545 | | | | | | | | | | | | | | | | | | | | | | | | | | | | | | | | | | | | | | | | 555 | | | | | | | | | | | | | | | | | | | | | | | | | | | | | | | | | | | | | | | | | | | | | NSLENYAYNMR | | | | | | | | | | | | | | | | | | | | | | | | | | | | | | | | | | | | | | | | | | | | | | | | | | | | | | | | | | | | | | | | | | | | | | | | | | | | | | | | | | | | | | | | | | | | | | | | | | | | | | | | | | | | | | | | | | | | | | | | | | | | | | | | | | | | | | | | | | | | | | | | | | | | | | | | | | | | | | | | | | | | | | | | | | | | | | | | | | | | | | | | | | | |  |  |  |  |  |  |  |  |  |  |  |  |  |  |  |  |
|  | | | | 1389.5983 | | | | | | | | | | | | | | | | | 1389.598 | | | | | | | | | | | | | | | | | | | | | | | | | | | | | | | | 0 | | | | | | | | | | | | | | | | | | | | | | | | | | | | | | | | 0 | | | | | | | | | | | | | | | | | | | | | | | | | | | | | | | | | | 545 | | | | | | | | | | | | | | | | | | | | | | | | | | | | | | | | | | | | | | | | 555 | | | | | | | | | | | | | | | | | | | | | | | | | | | | | | | | | | | | | | | | | | | | | NSLENYAYNMR | | | | | | | | | | | | | | | | | | | | | | | | | | | | | | | | | | | | | | | | | | | | | | | | | | | | | | | | | | | | | | | | | | | | | | | | | | | | | | | | | | | | | | | | | | | | | | | | | | | | | | | | | | | | | | | | | | | | | | | | | | | | | | | | | | | | | | | | | | | | | | | | | | | | | | | | | | | | | | | | | | | | | | | | | | | | | | | | | | | | | | | | | | | |  |  |  |  |  |  |  |  |  |  |  |  |  |  |  |  |
|  | | | | 1389.5983 | | | | | | | | | | | | | | | | | 1389.6 | | | | | | | | | | | | | | | | | | | | | | | | | | | | | | | | 0 | | | | | | | | | | | | | | | | | | | | | | | | | | | | | | | | 1 | | | | | | | | | | | | | | | | | | | | | | | | | | | | | | | | | | 545 | | | | | | | | | | | | | | | | | | | | | | | | | | | | | | | | | | | | | | | | 555 | | | | | | | | | | | | | | | | | | | | | | | | | | | | | | | | | | | | | | | | | | | | | NSLENYAYNMR | | | | | | | | | | | | | | | | | | | | | | | | | | | | | | | | | | | | | | | | | | | | | | | | | | | | | | | | | | | | | | | | | | | | | | | | | | | | | | | | | | | | | | | | | | | | | | | | | | | | | | | | | | | | | | | | | | | | | | | | | | | | | | | | | | | | | | | | | | | | | | | | | | | | | | | | | | | | | | | | | | | | | | | | | | | | | | | | | | | | | | | | | | | |  |  |  |  |  |  |  |  |  |  |  |  |  |  |  |  |
|  | | | | 1355.7119 | | | | | | | | | | | | | | | | | 1355.711 | | | | | | | | | | | | | | | | | | | | | | | | | | | | | | | | 0 | | | | | | | | | | | | | | | | | | | | | | | | | | | | | | | | -1 | | | | | | | | | | | | | | | | | | | | | | | | | | | | | | | | | | 603 | | | | | | | | | | | | | | | | | | | | | | | | | | | | | | | | | | | | | | | | 614 | | | | | | | | | | | | | | | | | | | | | | | | | | | | | | | | | | | | | | | | | | | | | ELEGICNPIIAK | | | | | | | | | | | | | | | | | | | | | | | | | | | | | | | | | | | | | | | | | | | | | | | | | | | | | | | | | | | | | | | | | | | | | | | | | | | | | | | | | | | | | | | | | | | | | | | | | | | | | | | | | | | | | | | | | | | | | | | | | | | | | | | | | | | | | | | | | | | | | | | | | | | | | | | | | | | | | | | | | | | | | | | | | | | | | | | | | | | | | | | | | | | |  |  |  |  |  |  |  |  |  |  |  |  |  |  |  |  |
|  | | | | 1355.7119 | | | | | | | | | | | | | | | | | 1355.711 | | | | | | | | | | | | | | | | | | | | | | | | | | | | | | | | 0 | | | | | | | | | | | | | | | | | | | | | | | | | | | | | | | | 0 | | | | | | | | | | | | | | | | | | | | | | | | | | | | | | | | | | 603 | | | | | | | | | | | | | | | | | | | | | | | | | | | | | | | | | | | | | | | | 614 | | | | | | | | | | | | | | | | | | | | | | | | | | | | | | | | | | | | | | | | | | | | | ELEGICNPIIAK | | | | | | | | | | | | | | | | | | | | | | | | | | | | | | | | | | | | | | | | | | | | | | | | | | | | | | | | | | | | | | | | | | | | | | | | | | | | | | | | | | | | | | | | | | | | | | | | | | | | | | | | | | | | | | | | | | | | | | | | | | | | | | | | | | | | | | | | | | | | | | | | | | | | | | | | | | | | | | | | | | | | | | | | | | | | | | | | | | | | | | | | | | | |  |  |  |  |  |  |  |  |  |  |  |  |  |  |  |  |
|  | | | | 2373.0552 | | | | | | | | | | | | | | | | | 2373.051 | | | | | | | | | | | | | | | | | | | | | | | | | | | | | | | | 0 | | | | | | | | | | | | | | | | | | | | | | | | | | | | | | | | -2 | | | | | | | | | | | | | | | | | | | | | | | | | | | | | | | | | | 615 | | | | | | | | | | | | | | | | | | | | | | | | | | | | | | | | | | | | | | | | 640 | | | | | | | | | | | | | | | | | | | | | | | | | | | | | | | | | | | | | | | | | | | | | MYQGAAGPGGDVPMGADMPAAGAGPK | | | | | | | | | | | | | | | | | | | | | | | | | | | | | | | | | | | | | | | | | | | | | | | | | | | | | | | | | | | | | | | | | | | | | | | | | | | | | | | | | | | | | | | | | | | | | | | | | | | | | | | | | | | | | | | | | | | | | | | | | | | | | | | | | | | | | | | | | | | | | | | | | | | | | | | | | | | | | | | | | | | | | | | | | | | | | | | | | | | | | | | | | | | |  |  |  |  |  |  |  |  |  |  |  |  |  |  |  |  |
|  | | | | 2389.0501 | | | | | | | | | | | | | | | | | 2389.043 | | | | | | | | | | | | | | | | | | | | | | | | | | | | | | | | 0 | | | | | | | | | | | | | | | | | | | | | | | | | | | | | | | | -3 | | | | | | | | | | | | | | | | | | | | | | | | | | | | | | | | | | 615 | | | | | | | | | | | | | | | | | | | | | | | | | | | | | | | | | | | | | | | | 640 | | | | | | | | | | | | | | | | | | | | | | | | | | | | | | | | | | | | | | | | | | | | | MYQGAAGPGGDVPMGADMPAAGAGPK | | | | | | | | | | | | | | | | | | | | | | | | | | | | | | | | | | | | | | | | | | | | | | | | | | | | | | | | | | | | | | | | | | | | | | | | | | | | | | | | | | | | | | | | | | | | | | | | | | | | | | | | | | | | | | | | | | | | | | | | | | | | | | | | | | | | | | | | | | | | | | | | | | | | | | | | | | | | | | | | | | | | | | | | | | | | | | | | | | | | | | | | | | | |  |  |  |  |  |  |  |  |  |  |  |  |  |  |  |  |
|  | | | | 2405.045 | | | | | | | | | | | | | | | | | 2405.04 | | | | | | | | | | | | | | | | | | | | | | | | | | | | | | | | 0 | | | | | | | | | | | | | | | | | | | | | | | | | | | | | | | | -2 | | | | | | | | | | | | | | | | | | | | | | | | | | | | | | | | | | 615 | | | | | | | | | | | | | | | | | | | | | | | | | | | | | | | | | | | | | | | | 640 | | | | | | | | | | | | | | | | | | | | | | | | | | | | | | | | | | | | | | | | | | | | | MYQGAAGPGGDVPMGADMPAAGAGPK | | | | | | | | | | | | | | | | | | | | | | | | | | | | | | | | | | | | | | | | | | | | | | | | | | | | | | | | | | | | | | | | | | | | | | | | | | | | | | | | | | | | | | | | | | | | | | | | | | | | | | | | | | | | | | | | | | | | | | | | | | | | | | | | | | | | | | | | | | | | | | | | | | | | | | | | | | | | | | | | | | | | | | | | | | | | | | | | | | | | | | | | | | | |  |  |  |  |  |  |  |  |  |  |  |  |  |  |  |  |
|  | | | | 2405.045 | | | | | | | | | | | | | | | | | 2405.041 | | | | | | | | | | | | | | | | | | | | | | | | | | | | | | | | 0 | | | | | | | | | | | | | | | | | | | | | | | | | | | | | | | | -1 | | | | | | | | | | | | | | | | | | | | | | | | | | | | | | | | | | 615 | | | | | | | | | | | | | | | | | | | | | | | | | | | | | | | | | | | | | | | | 640 | | | | | | | | | | | | | | | | | | | | | | | | | | | | | | | | | | | | | | | | | | | | | MYQGAAGPGGDVPMGADMPAAGAGPK | | | | | | | | | | | | | | | | | | | | | | | | | | | | | | | | | | | | | | | | | | | | | | | | | | | | | | | | | | | | | | | | | | | | | | | | | | | | | | | | | | | | | | | | | | | | | | | | | | | | | | | | | | | | | | | | | | | | | | | | | | | | | | | | | | | | | | | | | | | | | | | | | | | | | | | | | | | | | | | | | | | | | | | | | | | | | | | | | | | | | | | | | | | |  |  |  |  |  |  |  |  |  |  |  |  |  |  |  |  |
|  | | | | 2405.045 | | | | | | | | | | | | | | | | | 2405.042 | | | | | | | | | | | | | | | | | | | | | | | | | | | | | | | | 0 | | | | | | | | | | | | | | | | | | | | | | | | | | | | | | | | -1 | | | | | | | | | | | | | | | | | | | | | | | | | | | | | | | | | | 615 | | | | | | | | | | | | | | | | | | | | | | | | | | | | | | | | | | | | | | | | 640 | | | | | | | | | | | | | | | | | | | | | | | | | | | | | | | | | | | | | | | | | | | | | MYQGAAGPGGDVPMGADMPAAGAGPK | | | | | | | | | | | | | | | | | | | | | | | | | | | | | | | | | | | | | | | | | | | | | | | | | | | | | | | | | | | | | | | | | | | | | | | | | | | | | | | | | | | | | | | | | | | | | | | | | | | | | | | | | | | | | | | | | | | | | | | | | | | | | | | | | | | | | | | | | | | | | | | | | | | | | | | | | | | | | | | | | | | | | | | | | | | | | | | | | | | | | | | | | | | |  |  |  |  |  |  |  |  |  |  |  |  |  |  |  |  |
|  | | | | 2421.0399 | | | | | | | | | | | | | | | | | 2421.037 | | | | | | | | | | | | | | | | | | | | | | | | | | | | | | | | 0 | | | | | | | | | | | | | | | | | | | | | | | | | | | | | | | | -1 | | | | | | | | | | | | | | | | | | | | | | | | | | | | | | | | | | 615 | | | | | | | | | | | | | | | | | | | | | | | | | | | | | | | | | | | | | | | | 640 | | | | | | | | | | | | | | | | | | | | | | | | | | | | | | | | | | | | | | | | | | | | | MYQGAAGPGGDVPMGADMPAAGAGPK | | | | | | | | | | | | | | | | | | | | | | | | | | | | | | | | | | | | | | | | | | | | | | | | | | | | | | | | | | | | | | | | | | | | | | | | | | | | | | | | | | | | | | | | | | | | | | | | | | | | | | | | | | | | | | | | | | | | | | | | | | | | | | | | | | | | | | | | | | | | | | | | | | | | | | | | | | | | | | | | | | | | | | | | | | | | | | | | | | | | | | | | | | | |  |  |  |  |  |  |  |  |  |  |  |  |  |  |  |  |
|  | | | | 2421.0399 | | | | | | | | | | | | | | | | | 2421.037 | | | | | | | | | | | | | | | | | | | | | | | | | | | | | | | | 0 | | | | | | | | | | | | | | | | | | | | | | | | | | | | | | | | -1 | | | | | | | | | | | | | | | | | | | | | | | | | | | | | | | | | | 615 | | | | | | | | | | | | | | | | | | | | | | | | | | | | | | | | | | | | | | | | 640 | | | | | | | | | | | | | | | | | | | | | | | | | | | | | | | | | | | | | | | | | | | | | MYQGAAGPGGDVPMGADMPAAGAGPK | | | | | | | | | | | | | | | | | | | | | | | | | | | | | | | | | | | | | | | | | | | | | | | | | | | | | | | | | | | | | | | | | | | | | | | | | | | | | | | | | | | | | | | | | | | | | | | | | | | | | | | | | | | | | | | | | | | | | | | | | | | | | | | | | | | | | | | | | | | | | | | | | | | | | | | | | | | | | | | | | | | | | | | | | | | | | | | | | | | | | | | | | | | |  |  |  |  |  |  |  |  |  |  |  |  |  |  |  |  |
|  | | | | 2421.0399 | | | | | | | | | | | | | | | | | 2421.039 | | | | | | | | | | | | | | | | | | | | | | | | | | | | | | | | 0 | | | | | | | | | | | | | | | | | | | | | | | | | | | | | | | | 0 | | | | | | | | | | | | | | | | | | | | | | | | | | | | | | | | | | 615 | | | | | | | | | | | | | | | | | | | | | | | | | | | | | | | | | | | | | | | | 640 | | | | | | | | | | | | | | | | | | | | | | | | | | | | | | | | | | | | | | | | | | | | | MYQGAAGPGGDVPMGADMPAAGAGPK | | | | | | | | | | | | | | | | | | | | | | | | | | | | | | | | | | | | | | | | | | | | | | | | | | | | | | | | | | | | | | | | | | | | | | | | | | | | | | | | | | | | | | | | | | | | | | | | | | | | | | | | | | | | | | | | | | | | | | | | | | | | | | | | | | | | | | | | | | | | | | | | | | | | | | | | | | | | | | | | | | | | | | | | | | | | | | | | | | | | | | | | | | | |  |  |  |  |  |  |  |  |  |  |  |  |  |  |  |  |
| Spot no. | | | | Homologous protein | | | | | | | | | | | | | | | | | | | | Accession no. | | | | | | | | | | | | | | | | | | | | | | | | | | | | | | | | | | | | Score | | | | | | | | | | | | | | | | | | | | | | | | | | | | | M.P. | | | | | | | | | | | | | | | | | | | | | | | | | | | | | | | | | | | | Cov. (%) | | | | | | | | | | | | | | | | | | | | | | | | | | | | | | | | | | | | | | | | Blast score | | | | | | | | | | | | | | | | | | | | | | | | | | | | | | | | | | | | | | | | | | | | Mr (kDa) / pI | | | | | | | | | | | | | | | | | | | | | | | | | | | | | | | | | | | | | | | | | | | | | | | | | | | | | | | | | | | | | | | | | | | | | | | | | | | | | | | | | | | | | | | | | | | | | | | | | | | | | | | | | | | | | | | | | | | | | | | | | | | | | | | | | | | | | | | | | | | | | | | | | | | | | | | | | Ratio | | | | | | | | | | | | | | | | | | | | | | | | | | | | | |  |  |  |  |  |  |  |  |  |  |  |  |  |  |  |  |
|  |  |  |  |  |  |  |  |  |  |  |  |  |  |  |  |  |  |  |  |  |  |  |  |  |  |  |  |  |  |  |  |  |  |  |  |  |  |  |  |  |  |  |  |  |  |  |  |  |  |  |  |  |  |  |  |  |  |  |  |  |  |  |  |  |  |  |  |  |  |  |  |  |  |  |  |  |  |  |  |  |  |  |  |  |  |  |  |  |  |  |  |  |  |  |  |  |  |  |  |  |  |  |  |  |  |  |  |  |  |  |  |  |  |  |  |  |  |  |  |  |  |  |  |  |  |  |  |  |  |  |  |  |  |  |  |  |  |  |  |  |  |  |  |  |  |  |  |  |  |  |  |  |  |  |  |  |  |  |  |  |  |  |  |  |  |  |  |  |  |  |  |  |  |  |  |  |  |  |  |  |  |  |  |  |  |  |  |  |  |  |  |  |  |  |  |  |  |  |  |  |  |  |  |  |  |  |  |  | Theo. | | | | | | | | | | | | | | | | | | | | | | | | | | | | | | | | | | | | | | | | | | | | | | | | | | | | | | | | | | | | | | | | | | | | | | | | | | | | | | | Exp. | | | | | | | | | | | | | | | | | | | | | | | | | | | | | | | | | | | | | | | | | | | | | | | | | | | | | | | | | | | | | | | | | | | | | | | | | |  |  |  |  |  |  |  |  |  |  |  |  |  |  |  |  |  |  |  |  |  |  |  |  |  |  |  |  |  |  |  |  |  |  |  |  |  |  |  |  |  |  |  |  |  |  |
| 5b | | | | Phosphoglucomutase | | | | | | | | | | | | | | | | | | | | Glyma05g34790.1 | | | | | | | | | | | | | | | | | | | | | | | | | | | | | | | | | | | | 268 | | | | | | | | | | | | | | | | | | | | | | | | | | | | | 4 | | | | | | | | | | | | | | | | | | | | | | | | | | | | | | | | | | | | 9 | | | | | | | | | | | | | | | | | | | | | | | | | | | | | | | | | | | | | | | | 1057 | | | | | | | | | | | | | | | | | | | | | | | | | | | | | | | | | | | | | | | | | | | | 63.9/5.33 | | | | | | | | | | | | | | | | | | | | | | | | | | | | | | | | | | | | | | | | | | | | | | | | | | | | | | | | | | | | | | | | | | | | | | | | | | | | | | |  | | | | | | | | | | | | | | | | | | | | | | | | | | | | | | | | | | | | | | | | | | | | | | | | | | | | | | | | | | | | | | | | | | | | | | | | | |  | | | | | | | | | | | | | | | | | | | | | | | | | | | | | |  |  |  |  |  |  |  |  |  |  |  |  |  |  |  |  |
|  | | | | Calc. Mass | | | | | | | | | | | | | | | | | | | | Observ. Mass | | | | | | | | | | | | | | | | | | | | | | | | | | | | | | | | | | | | Da | | | | | | | | | | | | | | | | | | | | | | | | | | | | | ppm | | | | | | | | | | | | | | | | | | | | | | | | | | | | | | | | | | | | start | | | | | | | | | | | | | | | | | | | | | | | | | | | | | | | | | | | | | | | | end | | | | | | | | | | | | | | | | | | | | | | | | | | | | | | | | | | | | | | | | | | | | sequence | | | | | | | | | | | | | | | | | | | | | | | | | | | | | | | | | | | | | | | | | | | | | | | | | | | | | | | | | | | | | | | | | | | | | | | | | | | | | | | | | | | | | | | | | | | | | | | | | | | | | | | | | | | | | | | | | | | | | | | | | | | | | | | | | | | | | | | | | | | | | | | | | | | | | | | | | | | | | | | | | | | | | | | | | | | | | | | | | | | | | | |  |  |  |  |  |  |  |  |  |  |  |  |  |  |  |  |
|  | | | | 1788.9007 | | | | | | | | | | | | | | | | | | | | 1788.899 | | | | | | | | | | | | | | | | | | | | | | | | | | | | | | | | | | | | 0 | | | | | | | | | | | | | | | | | | | | | | | | | | | | | -1 | | | | | | | | | | | | | | | | | | | | | | | | | | | | | | | | | | | | 9 | | | | | | | | | | | | | | | | | | | | | | | | | | | | | | | | | | | | | | | | 25 | | | | | | | | | | | | | | | | | | | | | | | | | | | | | | | | | | | | | | | | | | | | VETTPFDGQKPGTSGLR | | | | | | | | | | | | | | | | | | | | | | | | | | | | | | | | | | | | | | | | | | | | | | | | | | | | | | | | | | | | | | | | | | | | | | | | | | | | | | | | | | | | | | | | | | | | | | | | | | | | | | | | | | | | | | | | | | | | | | | | | | | | | | | | | | | | | | | | | | | | | | | | | | | | | | | | | | | | | | | | | | | | | | | | | | | | | | | | | | | | | | |  |  |  |  |  |  |  |  |  |  |  |  |  |  |  |  |
|  | | | | 1788.9007 | | | | | | | | | | | | | | | | | | | | 1788.9 | | | | | | | | | | | | | | | | | | | | | | | | | | | | | | | | | | | | 0 | | | | | | | | | | | | | | | | | | | | | | | | | | | | | -1 | | | | | | | | | | | | | | | | | | | | | | | | | | | | | | | | | | | | 9 | | | | | | | | | | | | | | | | | | | | | | | | | | | | | | | | | | | | | | | | 25 | | | | | | | | | | | | | | | | | | | | | | | | | | | | | | | | | | | | | | | | | | | | VETTPFDGQKPGTSGLR | | | | | | | | | | | | | | | | | | | | | | | | | | | | | | | | | | | | | | | | | | | | | | | | | | | | | | | | | | | | | | | | | | | | | | | | | | | | | | | | | | | | | | | | | | | | | | | | | | | | | | | | | | | | | | | | | | | | | | | | | | | | | | | | | | | | | | | | | | | | | | | | | | | | | | | | | | | | | | | | | | | | | | | | | | | | | | | | | | | | | | |  |  |  |  |  |  |  |  |  |  |  |  |  |  |  |  |
|  | | | | 1691.7461 | | | | | | | | | | | | | | | | | | | | 1691.741 | | | | | | | | | | | | | | | | | | | | | | | | | | | | | | | | | | | | 0 | | | | | | | | | | | | | | | | | | | | | | | | | | | | | -3 | | | | | | | | | | | | | | | | | | | | | | | | | | | | | | | | | | | | 138 | | | | | | | | | | | | | | | | | | | | | | | | | | | | | | | | | | | | | | | | 153 | | | | | | | | | | | | | | | | | | | | | | | | | | | | | | | | | | | | | | | | | | | | YNMENGGPAPEGITDK | | | | | | | | | | | | | | | | | | | | | | | | | | | | | | | | | | | | | | | | | | | | | | | | | | | | | | | | | | | | | | | | | | | | | | | | | | | | | | | | | | | | | | | | | | | | | | | | | | | | | | | | | | | | | | | | | | | | | | | | | | | | | | | | | | | | | | | | | | | | | | | | | | | | | | | | | | | | | | | | | | | | | | | | | | | | | | | | | | | | | | |  |  |  |  |  |  |  |  |  |  |  |  |  |  |  |  |
|  | | | | 1691.7461 | | | | | | | | | | | | | | | | | | | | 1691.743 | | | | | | | | | | | | | | | | | | | | | | | | | | | | | | | | | | | | 0 | | | | | | | | | | | | | | | | | | | | | | | | | | | | | -2 | | | | | | | | | | | | | | | | | | | | | | | | | | | | | | | | | | | | 138 | | | | | | | | | | | | | | | | | | | | | | | | | | | | | | | | | | | | | | | | 153 | | | | | | | | | | | | | | | | | | | | | | | | | | | | | | | | | | | | | | | | | | | | YNMENGGPAPEGITDK | | | | | | | | | | | | | | | | | | | | | | | | | | | | | | | | | | | | | | | | | | | | | | | | | | | | | | | | | | | | | | | | | | | | | | | | | | | | | | | | | | | | | | | | | | | | | | | | | | | | | | | | | | | | | | | | | | | | | | | | | | | | | | | | | | | | | | | | | | | | | | | | | | | | | | | | | | | | | | | | | | | | | | | | | | | | | | | | | | | | | | |  |  |  |  |  |  |  |  |  |  |  |  |  |  |  |  |
|  | | | | 1288.6697 | | | | | | | | | | | | | | | | | | | | 1288.668 | | | | | | | | | | | | | | | | | | | | | | | | | | | | | | | | | | | | 0 | | | | | | | | | | | | | | | | | | | | | | | | | | | | | -1 | | | | | | | | | | | | | | | | | | | | | | | | | | | | | | | | | | | | 345 | | | | | | | | | | | | | | | | | | | | | | | | | | | | | | | | | | | | | | | | 357 | | | | | | | | | | | | | | | | | | | | | | | | | | | | | | | | | | | | | | | | | | | | SMPTSAALDVVAK | | | | | | | | | | | | | | | | | | | | | | | | | | | | | | | | | | | | | | | | | | | | | | | | | | | | | | | | | | | | | | | | | | | | | | | | | | | | | | | | | | | | | | | | | | | | | | | | | | | | | | | | | | | | | | | | | | | | | | | | | | | | | | | | | | | | | | | | | | | | | | | | | | | | | | | | | | | | | | | | | | | | | | | | | | | | | | | | | | | | | | |  |  |  |  |  |  |  |  |  |  |  |  |  |  |  |  |
|  | | | | 1288.6697 | | | | | | | | | | | | | | | | | | | | 1288.669 | | | | | | | | | | | | | | | | | | | | | | | | | | | | | | | | | | | | 0 | | | | | | | | | | | | | | | | | | | | | | | | | | | | | 0 | | | | | | | | | | | | | | | | | | | | | | | | | | | | | | | | | | | | 345 | | | | | | | | | | | | | | | | | | | | | | | | | | | | | | | | | | | | | | | | 357 | | | | | | | | | | | | | | | | | | | | | | | | | | | | | | | | | | | | | | | | | | | | SMPTSAALDVVAK | | | | | | | | | | | | | | | | | | | | | | | | | | | | | | | | | | | | | | | | | | | | | | | | | | | | | | | | | | | | | | | | | | | | | | | | | | | | | | | | | | | | | | | | | | | | | | | | | | | | | | | | | | | | | | | | | | | | | | | | | | | | | | | | | | | | | | | | | | | | | | | | | | | | | | | | | | | | | | | | | | | | | | | | | | | | | | | | | | | | | | |  |  |  |  |  |  |  |  |  |  |  |  |  |  |  |  |
|  | | | | 1304.6646 | | | | | | | | | | | | | | | | | | | | 1304.664 | | | | | | | | | | | | | | | | | | | | | | | | | | | | | | | | | | | | 0 | | | | | | | | | | | | | | | | | | | | | | | | | | | | | 0 | | | | | | | | | | | | | | | | | | | | | | | | | | | | | | | | | | | | 345 | | | | | | | | | | | | | | | | | | | | | | | | | | | | | | | | | | | | | | | | 357 | | | | | | | | | | | | | | | | | | | | | | | | | | | | | | | | | | | | | | | | | | | | SMPTSAALDVVAK | | | | | | | | | | | | | | | | | | | | | | | | | | | | | | | | | | | | | | | | | | | | | | | | | | | | | | | | | | | | | | | | | | | | | | | | | | | | | | | | | | | | | | | | | | | | | | | | | | | | | | | | | | | | | | | | | | | | | | | | | | | | | | | | | | | | | | | | | | | | | | | | | | | | | | | | | | | | | | | | | | | | | | | | | | | | | | | | | | | | | | |  |  |  |  |  |  |  |  |  |  |  |  |  |  |  |  |
|  | | | | 1304.6646 | | | | | | | | | | | | | | | | | | | | 1304.664 | | | | | | | | | | | | | | | | | | | | | | | | | | | | | | | | | | | | 0 | | | | | | | | | | | | | | | | | | | | | | | | | | | | | 0 | | | | | | | | | | | | | | | | | | | | | | | | | | | | | | | | | | | | 345 | | | | | | | | | | | | | | | | | | | | | | | | | | | | | | | | | | | | | | | | 357 | | | | | | | | | | | | | | | | | | | | | | | | | | | | | | | | | | | | | | | | | | | | SMPTSAALDVVAK | | | | | | | | | | | | | | | | | | | | | | | | | | | | | | | | | | | | | | | | | | | | | | | | | | | | | | | | | | | | | | | | | | | | | | | | | | | | | | | | | | | | | | | | | | | | | | | | | | | | | | | | | | | | | | | | | | | | | | | | | | | | | | | | | | | | | | | | | | | | | | | | | | | | | | | | | | | | | | | | | | | | | | | | | | | | | | | | | | | | | | |  |  |  |  |  |  |  |  |  |  |  |  |  |  |  |  |
|  | | | | 1511.7507 | | | | | | | | | | | | | | | | | | | | 1511.749 | | | | | | | | | | | | | | | | | | | | | | | | | | | | | | | | | | | | 0 | | | | | | | | | | | | | | | | | | | | | | | | | | | | | -1 | | | | | | | | | | | | | | | | | | | | | | | | | | | | | | | | | | | | 536 | | | | | | | | | | | | | | | | | | | | | | | | | | | | | | | | | | | | | | | | 547 | | | | | | | | | | | | | | | | | | | | | | | | | | | | | | | | | | | | | | | | | | | | LYIEQYEKDPSK | | | | | | | | | | | | | | | | | | | | | | | | | | | | | | | | | | | | | | | | | | | | | | | | | | | | | | | | | | | | | | | | | | | | | | | | | | | | | | | | | | | | | | | | | | | | | | | | | | | | | | | | | | | | | | | | | | | | | | | | | | | | | | | | | | | | | | | | | | | | | | | | | | | | | | | | | | | | | | | | | | | | | | | | | | | | | | | | | | | | | | |  |  |  |  |  |  |  |  |  |  |  |  |  |  |  |  |
|  | | | | 1511.7507 | | | | | | | | | | | | | | | | | | | | 1511.751 | | | | | | | | | | | | | | | | | | | | | | | | | | | | | | | | | | | | 0 | | | | | | | | | | | | | | | | | | | | | | | | | | | | | 0 | | | | | | | | | | | | | | | | | | | | | | | | | | | | | | | | | | | | 536 | | | | | | | | | | | | | | | | | | | | | | | | | | | | | | | | | | | | | | | | 547 | | | | | | | | | | | | | | | | | | | | | | | | | | | | | | | | | | | | | | | | | | | | LYIEQYEKDPSK | | | | | | | | | | | | | | | | | | | | | | | | | | | | | | | | | | | | | | | | | | | | | | | | | | | | | | | | | | | | | | | | | | | | | | | | | | | | | | | | | | | | | | | | | | | | | | | | | | | | | | | | | | | | | | | | | | | | | | | | | | | | | | | | | | | | | | | | | | | | | | | | | | | | | | | | | | | | | | | | | | | | | | | | | | | | | | | | | | | | | | |  |  |  |  |  |  |  |  |  |  |  |  |  |  |  |  |
| Spot no. | | | | Homologous protein | | | | | | | | | | | | | | | | | | | | Accession no. | | | | | | | | | | | | | | | | | | | | | | | | | | | | | | | | | | | | Score | | | | | | | | | | | | | | | | | | | | | | | | | | | | | M.P. | | | | | | | | | | | | | | | | | | | | | | | | | | | | | | | | | | | | Cov. (%) | | | | | | | | | | | | | | | | | | | | | | | | | | | | | | | | | | | | | | | | Blast score | | | | | | | | | | | | | | | | | | | | | | | | | | | | | | | | | | | | | | | | | | | | Mr (kDa) / pI | | | | | | | | | | | | | | | | | | | | | | | | | | | | | | | | | | | | | | | | | | | | | | | | | | | | | | | | | | | | | | | | | | | | | | | | | | | | | | | | | | | | | | | | | | | | | | | | | | | | | | | | | | | | | | | | | | | | | | | | | | | | | | | | | | | | | | | | | | | | | Ratio | | | | | | | | | | | | | | | | | | | | | | | | | | | | | | | | | | | | | | | | | |  |  |  |  |  |  |  |  |  |  |  |  |  |  |  |  |
|  |  |  |  |  |  |  |  |  |  |  |  |  |  |  |  |  |  |  |  |  |  |  |  |  |  |  |  |  |  |  |  |  |  |  |  |  |  |  |  |  |  |  |  |  |  |  |  |  |  |  |  |  |  |  |  |  |  |  |  |  |  |  |  |  |  |  |  |  |  |  |  |  |  |  |  |  |  |  |  |  |  |  |  |  |  |  |  |  |  |  |  |  |  |  |  |  |  |  |  |  |  |  |  |  |  |  |  |  |  |  |  |  |  |  |  |  |  |  |  |  |  |  |  |  |  |  |  |  |  |  |  |  |  |  |  |  |  |  |  |  |  |  |  |  |  |  |  |  |  |  |  |  |  |  |  |  |  |  |  |  |  |  |  |  |  |  |  |  |  |  |  |  |  |  |  |  |  |  |  |  |  |  |  |  |  |  |  |  |  |  |  |  |  |  |  |  |  |  |  |  |  |  |  |  |  |  |  |  | Theo. | | | | | | | | | | | | | | | | | | | | | | | | | | | | | | | | | | | | | | | | | | | | | | | | | | | | | | | | | | | | | | | | | | | | | Exp. | | | | | | | | | | | | | | | | | | | | | | | | | | | | | | | | | | | | | | | | | | | | | | | | | | | | | | | | | | | | | | | | | | | | | | | |  |  |  |  |  |  |  |  |  |  |  |  |  |  |  |  |  |  |  |  |  |  |  |  |  |  |  |  |  |  |  |  |  |  |  |  |  |  |  |  |  |  |  |  |  |  |  |  |  |  |  |  |  |  |  |  |  |  |
| 5c | | | | glyceraldehyde-3-phosphate dehydrogenase | | | | | | | | | | | | | | | | | | | | Glyma04g36860.1 | | | | | | | | | | | | | | | | | | | | | | | | | | | | | | | | | | | | 150 | | | | | | | | | | | | | | | | | | | | | | | | | | | | | 2 | | | | | | | | | | | | | | | | | | | | | | | | | | | | | | | | | | | | 12 | | | | | | | | | | | | | | | | | | | | | | | | | | | | | | | | | | | | | | | | 637 | | | | | | | | | | | | | | | | | | | | | | | | | | | | | | | | | | | | | | | | | | | | 37.1/6.72 | | | | | | | | | | | | | | | | | | | | | | | | | | | | | | | | | | | | | | | | | | | | | | | | | | | | | | | | | | | | | | | | | | | | |  | | | | | | | | | | | | | | | | | | | | | | | | | | | | | | | | | | | | | | | | | | | | | | | | | | | | | | | | | | | | | | | | | | | | | | | |  | | | | | | | | | | | | | | | | | | | | | | | | | | | | | | | | | | | | | | | | | |  |  |  |  |  |  |  |  |  |  |  |  |  |  |  |  |
|  | | | | Calc. Mass | | | | | | | | | | | | | | | | | | | | Observ. Mass | | | | | | | | | | | | | | | | | | | | | | | | | | | | | | | | | | | | Da | | | | | | | | | | | | | | | | | | | | | | | | | | | | | ppm | | | | | | | | | | | | | | | | | | | | | | | | | | | | | | | | | | | | start | | | | | | | | | | | | | | | | | | | | | | | | | | | | | | | | | | | | | | | | end | | | | | | | | | | | | | | | | | | | | | | | | | | | | | | | | | | | | | | | | | | | | sequence | | | | | | | | | | | | | | | | | | | | | | | | | | | | | | | | | | | | | | | | | | | | | | | | | | | | | | | | | | | | | | | | | | | | | | | | | | | | | | | | | | | | | | | | | | | | | | | | | | | | | | | | | | | | | | | | | | | | | | | | | | | | | | | | | | | | | | | | | | | | | | | | | | | | | | | | | | | | | | | | | | | | | | | | | | | | | | | | | | | | | | |  |  |  |  |  |  |  |  |  |  |  |  |  |  |  |  |
|  | | | | 2904.4029 | | | | | | | | | | | | | | | | | | | | 2904.4 | | | | | | | | | | | | | | | | | | | | | | | | | | | | | | | | | | | | 0 | | | | | | | | | | | | | | | | | | | | | | | | | | | | | -1 | | | | | | | | | | | | | | | | | | | | | | | | | | | | | | | | | | | | 83 | | | | | | | | | | | | | | | | | | | | | | | | | | | | | | | | | | | | | | | | 109 | | | | | | | | | | | | | | | | | | | | | | | | | | | | | | | | | | | | | | | | | | | | NPEEIPWGSTGADIIVESTGVFTDKDK | | | | | | | | | | | | | | | | | | | | | | | | | | | | | | | | | | | | | | | | | | | | | | | | | | | | | | | | | | | | | | | | | | | | | | | | | | | | | | | | | | | | | | | | | | | | | | | | | | | | | | | | | | | | | | | | | | | | | | | | | | | | | | | | | | | | | | | | | | | | | | | | | | | | | | | | | | | | | | | | | | | | | | | | | | | | | | | | | | | | | | |  |  |  |  |  |  |  |  |  |  |  |  |  |  |  |  |
|  | | | | 1497.8403 | | | | | | | | | | | | | | | | | | | | 1497.839 | | | | | | | | | | | | | | | | | | | | | | | | | | | | | | | | | | | | 0 | | | | | | | | | | | | | | | | | | | | | | | | | | | | | -1 | | | | | | | | | | | | | | | | | | | | | | | | | | | | | | | | | | | | 237 | | | | | | | | | | | | | | | | | | | | | | | | | | | | | | | | | | | | | | | | 250 | | | | | | | | | | | | | | | | | | | | | | | | | | | | | | | | | | | | | | | | | | | | VPTVDVSVVDLTVR | | | | | | | | | | | | | | | | | | | | | | | | | | | | | | | | | | | | | | | | | | | | | | | | | | | | | | | | | | | | | | | | | | | | | | | | | | | | | | | | | | | | | | | | | | | | | | | | | | | | | | | | | | | | | | | | | | | | | | | | | | | | | | | | | | | | | | | | | | | | | | | | | | | | | | | | | | | | | | | | | | | | | | | | | | | | | | | | | | | | | | |  |  |  |  |  |  |  |  |  |  |  |  |  |  |  |  |
| Spot no. | | | | Homologous protein | | | | | | | | | | | | | | | | | | | | Accession no. | | | | | | | | | | | | | | | | | | | | | | | | | | | | | | | | | | | | Score | | | | | | | | | | | | | | | | | | | | | | | | | | | | | M.P. | | | | | | | | | | | | | | | | | | | | | | | | | | | | | | | | | | | | Cov. (%) | | | | | | | | | | | | | | | | | | | | | | | | | | | | | | | | | | | | | | | | Blast score | | | | | | | | | | | | | | | | | | | | | | | | | | | | | | | | | | | | | | | | | | | | Mr (kDa) / pI | | | | | | | | | | | | | | | | | | | | | | | | | | | | | | | | | | | | | | | | | | | | | | | | | | | | | | | | | | | | | | | | | | | | | | | | | | | | | | | | | | | | | | | | | | | | | | | | | | | | | | | | | | | | | | | | | | | | | | | | | | | | | | | | | | | | | | | | | | | | | | | | | | | | Ratio | | | | | | | | | | | | | | | | | | | | | | | | | | | | | | | | | | |  |  |  |  |  |  |  |  |  |  |  |  |  |  |  |  |
|  |  |  |  |  |  |  |  |  |  |  |  |  |  |  |  |  |  |  |  |  |  |  |  |  |  |  |  |  |  |  |  |  |  |  |  |  |  |  |  |  |  |  |  |  |  |  |  |  |  |  |  |  |  |  |  |  |  |  |  |  |  |  |  |  |  |  |  |  |  |  |  |  |  |  |  |  |  |  |  |  |  |  |  |  |  |  |  |  |  |  |  |  |  |  |  |  |  |  |  |  |  |  |  |  |  |  |  |  |  |  |  |  |  |  |  |  |  |  |  |  |  |  |  |  |  |  |  |  |  |  |  |  |  |  |  |  |  |  |  |  |  |  |  |  |  |  |  |  |  |  |  |  |  |  |  |  |  |  |  |  |  |  |  |  |  |  |  |  |  |  |  |  |  |  |  |  |  |  |  |  |  |  |  |  |  |  |  |  |  |  |  |  |  |  |  |  |  |  |  |  |  |  |  |  |  |  |  |  | Theo. | | | | | | | | | | | | | | | | | | | | | | | | | | | | | | | | | | | | | | | | | Exp. | | | | | | | | | | | | | | | | | | | | | | | | | | | | | | | | | | | | | | | | | | | | | | | | | | | | | | | | | | | | | | | | | | | | | | | | | | | | | | | | | | | | | | | | | | | | | | | | | | | | | | | | | | |  |  |  |  |  |  |  |  |  |  |  |  |  |  |  |  |  |  |  |  |  |  |  |  |  |  |  |  |  |  |  |  |  |  |  |  |  |  |  |  |  |  |  |  |  |  |  |  |  |  |  |
| 5d | | | | uncharacterized protein LOC100819568 | | | | | | | | | | | | | | | | | | | | Glyma08g44590.1 | | | | | | | | | | | | | | | | | | | | | | | | | | | | | | | | | | | | 138 | | | | | | | | | | | | | | | | | | | | | | | | | | | | | 3 | | | | | | | | | | | | | | | | | | | | | | | | | | | | | | | | | | | | 5 | | | | | | | | | | | | | | | | | | | | | | | | | | | | | | | | | | | | | | | | 1167 | | | | | | | | | | | | | | | | | | | | | | | | | | | | | | | | | | | | | | | | | | | | 80.6/4.97 | | | | | | | | | | | | | | | | | | | | | | | | | | | | | | | | | | | | | | | | |  | | | | | | | | | | | | | | | | | | | | | | | | | | | | | | | | | | | | | | | | | | | | | | | | | | | | | | | | | | | | | | | | | | | | | | | | | | | | | | | | | | | | | | | | | | | | | | | | | | | | | | | | | | |  | | | | | | | | | | | | | | | | | | | | | | | | | | | | | | | | | | |  |  |  |  |  |  |  |  |  |  |  |  |  |  |  |  |
|  | | | | Calc. Mass | | | | | | | | | | | | | | | | | | | | Observ. Mass | | | | | | | | | | | | | | | | | | | | | | | | | | | | | | | | | | | | Da | | | | | | | | | | | | | | | | | | | | | | | | | | | | | ppm | | | | | | | | | | | | | | | | | | | | | | | | | | | | | | | | | | | | start | | | | | | | | | | | | | | | | | | | | | | | | | | | | | | | | | | | | | | | | end | | | | | | | | | | | | | | | | | | | | | | | | | | | | | | | | | | | | | | | | | | | | sequence | | | | | | | | | | | | | | | | | | | | | | | | | | | | | | | | | | | | | | | | | | | | | | | | | | | | | | | | | | | | | | | | | | | | | | | | | | | | | | | | | | | | | | | | | | | | | | | | | | | | | | | | | | | | | | | | | | | | | | | | | | | | | | | | | | | | | | | | | | | | | | | | | | | | | | | | | | | | | | | | | | | | | | | | | | | | | | | | | | | | | | |  |  |  |  |  |  |  |  |  |  |  |  |  |  |  |  |
|  | | | | 1274.6354 | | | | | | | | | | | | | | | | | | | | 1274.635 | | | | | | | | | | | | | | | | | | | | | | | | | | | | | | | | | | | | 0 | | | | | | | | | | | | | | | | | | | | | | | | | | | | | 0 | | | | | | | | | | | | | | | | | | | | | | | | | | | | | | | | | | | | 34 | | | | | | | | | | | | | | | | | | | | | | | | | | | | | | | | | | | | | | | | 45 | | | | | | | | | | | | | | | | | | | | | | | | | | | | | | | | | | | | | | | | | | | | ELISNASDALDK | | | | | | | | | | | | | | | | | | | | | | | | | | | | | | | | | | | | | | | | | | | | | | | | | | | | | | | | | | | | | | | | | | | | | | | | | | | | | | | | | | | | | | | | | | | | | | | | | | | | | | | | | | | | | | | | | | | | | | | | | | | | | | | | | | | | | | | | | | | | | | | | | | | | | | | | | | | | | | | | | | | | | | | | | | | | | | | | | | | | | | |  |  |  |  |  |  |  |  |  |  |  |  |  |  |  |  |
|  | | | | 1860.9145 | | | | | | | | | | | | | | | | | | | | 1860.909 | | | | | | | | | | | | | | | | | | | | | | | | | | | | | | | | | | | | 0 | | | | | | | | | | | | | | | | | | | | | | | | | | | | | -3 | | | | | | | | | | | | | | | | | | | | | | | | | | | | | | | | | | | | 271 | | | | | | | | | | | | | | | | | | | | | | | | | | | | | | | | | | | | | | | | 285 | | | | | | | | | | | | | | | | | | | | | | | | | | | | | | | | | | | | | | | | | | | | KPEEITKEEYSAFYK | | | | | | | | | | | | | | | | | | | | | | | | | | | | | | | | | | | | | | | | | | | | | | | | | | | | | | | | | | | | | | | | | | | | | | | | | | | | | | | | | | | | | | | | | | | | | | | | | | | | | | | | | | | | | | | | | | | | | | | | | | | | | | | | | | | | | | | | | | | | | | | | | | | | | | | | | | | | | | | | | | | | | | | | | | | | | | | | | | | | | | |  |  |  |  |  |  |  |  |  |  |  |  |  |  |  |  |
|  | | | | 1526.794 | | | | | | | | | | | | | | | | | | | | 1526.793 | | | | | | | | | | | | | | | | | | | | | | | | | | | | | | | | | | | | 0 | | | | | | | | | | | | | | | | | | | | | | | | | | | | | -1 | | | | | | | | | | | | | | | | | | | | | | | | | | | | | | | | | | | | 358 | | | | | | | | | | | | | | | | | | | | | | | | | | | | | | | | | | | | | | | | 371 | | | | | | | | | | | | | | | | | | | | | | | | | | | | | | | | | | | | | | | | | | | | GIVDSEDLPLNISR | | | | | | | | | | | | | | | | | | | | | | | | | | | | | | | | | | | | | | | | | | | | | | | | | | | | | | | | | | | | | | | | | | | | | | | | | | | | | | | | | | | | | | | | | | | | | | | | | | | | | | | | | | | | | | | | | | | | | | | | | | | | | | | | | | | | | | | | | | | | | | | | | | | | | | | | | | | | | | | | | | | | | | | | | | | | | | | | | | | | | | |  |  |  |  |  |  |  |  |  |  |  |  |  |  |  |  |
| Spot no. | | | | Homologous protein | | | | | | | | | | | | | | | | | | | | | | | | | Accession no. | | | | | | | | | | | | | | | | | | | | | | | | | | | | | | | | | | Score | | | | | | | | | | | | | | | | | | | | | | | | | | | | | | | | | M.P. | | | | | | | | | | | | | | | | | | | | | | | | | | | | | | | | | | | Cov. (%) | | | | | | | | | | | | | | | | | | | | | | | | | | | | | | | | | | | | | | | | | | | | Blast score | | | | | | | | | | | | | | | | | | | | | | | | | | | | | | | | | | | | | | | | | | Mr (kDa) / pI | | | | | | | | | | | | | | | | | | | | | | | | | | | | | | | | | | | | | | | | | | | | | | | | | | | | | | | | | | | | | | | | | | | | | | | | | | | | | | | | | | | | | | | | | | | | | | | | | | | | | | | | | | | | | | | | | | | | | | | | | | | | | | | | | | | | | | | | | | | | | | | | | Ratio | | | | | | | | | | | | | | | | | | | | | | | | | | | | | | | | | | | | | | |  |  |  |  |  |  |  |
|  |  |  |  |  |  |  |  |  |  |  |  |  |  |  |  |  |  |  |  |  |  |  |  |  |  |  |  |  |  |  |  |  |  |  |  |  |  |  |  |  |  |  |  |  |  |  |  |  |  |  |  |  |  |  |  |  |  |  |  |  |  |  |  |  |  |  |  |  |  |  |  |  |  |  |  |  |  |  |  |  |  |  |  |  |  |  |  |  |  |  |  |  |  |  |  |  |  |  |  |  |  |  |  |  |  |  |  |  |  |  |  |  |  |  |  |  |  |  |  |  |  |  |  |  |  |  |  |  |  |  |  |  |  |  |  |  |  |  |  |  |  |  |  |  |  |  |  |  |  |  |  |  |  |  |  |  |  |  |  |  |  |  |  |  |  |  |  |  |  |  |  |  |  |  |  |  |  |  |  |  |  |  |  |  |  |  |  |  |  |  |  |  |  |  |  |  |  |  |  |  |  |  |  |  |  |  |  |  |  |  |  |  |  |  |  |  | Theo. | | | | | | | | | | | | | | | | | | | | | | | | | | | | | | | | | | | | | | | | | Exp. | | | | | | | | | | | | | | | | | | | | | | | | | | | | | | | | | | | | | | | | | | | | | | | | | | | | | | | | | | | | | | | | | | | | | | | | | | | | | | | | | | | | | | | | | | | | | | | | | | | | | | | |  |  |  |  |  |  |  |  |  |  |  |  |  |  |  |  |  |  |  |  |  |  |  |  |  |  |  |  |  |  |  |  |  |  |  |  |  |  |  |  |  |  |  |  |  |  |
| 5e | | | | Alpha-L-arabinofuranosidase | | | | | | | | | | | | | | | | | | | | | | | | | Glyma03g27460.1 | | | | | | | | | | | | | | | | | | | | | | | | | | | | | | | | | | 135 | | | | | | | | | | | | | | | | | | | | | | | | | | | | | | | | | 2 | | | | | | | | | | | | | | | | | | | | | | | | | | | | | | | | | | | 4 | | | | | | | | | | | | | | | | | | | | | | | | | | | | | | | | | | | | | | | | | | | | 1144 | | | | | | | | | | | | | | | | | | | | | | | | | | | | | | | | | | | | | | | | | | 74.6/5.84 | | | | | | | | | | | | | | | | | | | | | | | | | | | | | | | | | | | | | | | | |  | | | | | | | | | | | | | | | | | | | | | | | | | | | | | | | | | | | | | | | | | | | | | | | | | | | | | | | | | | | | | | | | | | | | | | | | | | | | | | | | | | | | | | | | | | | | | | | | | | | | | | | |  | | | | | | | | | | | | | | | | | | | | | | | | | | | | | | | | | | | | | | |  |  |  |  |  |  |  |
|  | | | | Calc. Mass | | | | | | | | | | | | | | | | | | | | | | | | | Observ. Mass | | | | | | | | | | | | | | | | | | | | | | | | | | | | | | | | | | Da | | | | | | | | | | | | | | | | | | | | | | | | | | | | | | | | | ppm | | | | | | | | | | | | | | | | | | | | | | | | | | | | | | | | | | | start | | | | | | | | | | | | | | | | | | | | | | | | | | | | | | | | | | | | | | | | | | | | end | | | | | | | | | | | | | | | | | | | | | | | | | | | | | | | | | | | | | | | | | | sequence | | | | | | | | | | | | | | | | | | | | | | | | | | | | | | | | | | | | | | | | | | | | | | | | | | | | | | | | | | | | | | | | | | | | | | | | | | | | | | | | | | | | | | | | | | | | | | | | | | | | | | | | | | | | | | | | | | | | | | | | | | | | | | | | | | | | | | | | | | | | | | | | | | | | | | | | | | | | | | | | | | | | | | | | | | | | | | | | | | | | | | | |  |  |  |  |  |  |  |
|  | | | | 1170.5921 | | | | | | | | | | | | | | | | | | | | | | | | | 1170.591 | | | | | | | | | | | | | | | | | | | | | | | | | | | | | | | | | | 0 | | | | | | | | | | | | | | | | | | | | | | | | | | | | | | | | | -1 | | | | | | | | | | | | | | | | | | | | | | | | | | | | | | | | | | | 459 | | | | | | | | | | | | | | | | | | | | | | | | | | | | | | | | | | | | | | | | | | | | 469 | | | | | | | | | | | | | | | | | | | | | | | | | | | | | | | | | | | | | | | | | | AFVSEYAVTGK | | | | | | | | | | | | | | | | | | | | | | | | | | | | | | | | | | | | | | | | | | | | | | | | | | | | | | | | | | | | | | | | | | | | | | | | | | | | | | | | | | | | | | | | | | | | | | | | | | | | | | | | | | | | | | | | | | | | | | | | | | | | | | | | | | | | | | | | | | | | | | | | | | | | | | | | | | | | | | | | | | | | | | | | | | | | | | | | | | | | | | | |  |  |  |  |  |  |  |
|  | | | | 1966.9306 | | | | | | | | | | | | | | | | | | | | | | | | | 1966.927 | | | | | | | | | | | | | | | | | | | | | | | | | | | | | | | | | | 0 | | | | | | | | | | | | | | | | | | | | | | | | | | | | | | | | | -2 | | | | | | | | | | | | | | | | | | | | | | | | | | | | | | | | | | | 609 | | | | | | | | | | | | | | | | | | | | | | | | | | | | | | | | | | | | | | | | | | | | 626 | | | | | | | | | | | | | | | | | | | | | | | | | | | | | | | | | | | | | | | | | | TVLTSANVMDENSFSQPK | | | | | | | | | | | | | | | | | | | | | | | | | | | | | | | | | | | | | | | | | | | | | | | | | | | | | | | | | | | | | | | | | | | | | | | | | | | | | | | | | | | | | | | | | | | | | | | | | | | | | | | | | | | | | | | | | | | | | | | | | | | | | | | | | | | | | | | | | | | | | | | | | | | | | | | | | | | | | | | | | | | | | | | | | | | | | | | | | | | | | | | |  |  |  |  |  |  |  |
|  | | | | 1966.9306 | | | | | | | | | | | | | | | | | | | | | | | | | 1966.927 | | | | | | | | | | | | | | | | | | | | | | | | | | | | | | | | | | 0 | | | | | | | | | | | | | | | | | | | | | | | | | | | | | | | | | -2 | | | | | | | | | | | | | | | | | | | | | | | | | | | | | | | | | | | 609 | | | | | | | | | | | | | | | | | | | | | | | | | | | | | | | | | | | | | | | | | | | | 626 | | | | | | | | | | | | | | | | | | | | | | | | | | | | | | | | | | | | | | | | | | TVLTSANVMDENSFSQPK | | | | | | | | | | | | | | | | | | | | | | | | | | | | | | | | | | | | | | | | | | | | | | | | | | | | | | | | | | | | | | | | | | | | | | | | | | | | | | | | | | | | | | | | | | | | | | | | | | | | | | | | | | | | | | | | | | | | | | | | | | | | | | | | | | | | | | | | | | | | | | | | | | | | | | | | | | | | | | | | | | | | | | | | | | | | | | | | | | | | | | | |  |  |  |  |  |  |  |
|  | | | | 1982.9255 | | | | | | | | | | | | | | | | | | | | | | | | | 1982.924 | | | | | | | | | | | | | | | | | | | | | | | | | | | | | | | | | | 0 | | | | | | | | | | | | | | | | | | | | | | | | | | | | | | | | | -1 | | | | | | | | | | | | | | | | | | | | | | | | | | | | | | | | | | | 609 | | | | | | | | | | | | | | | | | | | | | | | | | | | | | | | | | | | | | | | | | | | | 626 | | | | | | | | | | | | | | | | | | | | | | | | | | | | | | | | | | | | | | | | | | TVLTSANVMDENSFSQPK | | | | | | | | | | | | | | | | | | | | | | | | | | | | | | | | | | | | | | | | | | | | | | | | | | | | | | | | | | | | | | | | | | | | | | | | | | | | | | | | | | | | | | | | | | | | | | | | | | | | | | | | | | | | | | | | | | | | | | | | | | | | | | | | | | | | | | | | | | | | | | | | | | | | | | | | | | | | | | | | | | | | | | | | | | | | | | | | | | | | | | | |  |  |  |  |  |  |  |
| Spot no. | | | | | | Homologous protein | | | | | | | | | | | | | | | | | | | | | | | | | | | Accession no. | | | | | | | | | | | | | | | | | | | | | | | | | | | | | | | | | | | Score | | | | | | | | | | | | | | | | | | | | | | | | | | | | | | M.P. | | | | | | | | | | | | | | | | | | | | | | | | | | | | | | | | | | | Cov. (%) | | | | | | | | | | | | | | | | | | | | | | | | | | | | | | | | | | | | | | | | | | Blast score | | | | | | | | | | | | | | | | | | | | | | | | | | | | | | | | | | | | | | | | | | Mr (kDa) / pI | | | | | | | | | | | | | | | | | | | | | | | | | | | | | | | | | | | | | | | | | | | | | | | | | | | | | | | | | | | | | | | | | | | | | | | | | | | | | | | | | | | | | | | | | | | | | | | | | | | | | | | | | | | | | | | | | | | | | | | | | | | | | | | | | | | | | | | | | | | | Ratio | | | | | | | | | | | | | | | | | | | | | | | | | | | | | | | | | | | | | | | | | | | |  |  |  |  |  |  |  |
|  |  |  |  |  |  |  |  |  |  |  |  |  |  |  |  |  |  |  |  |  |  |  |  |  |  |  |  |  |  |  |  |  |  |  |  |  |  |  |  |  |  |  |  |  |  |  |  |  |  |  |  |  |  |  |  |  |  |  |  |  |  |  |  |  |  |  |  |  |  |  |  |  |  |  |  |  |  |  |  |  |  |  |  |  |  |  |  |  |  |  |  |  |  |  |  |  |  |  |  |  |  |  |  |  |  |  |  |  |  |  |  |  |  |  |  |  |  |  |  |  |  |  |  |  |  |  |  |  |  |  |  |  |  |  |  |  |  |  |  |  |  |  |  |  |  |  |  |  |  |  |  |  |  |  |  |  |  |  |  |  |  |  |  |  |  |  |  |  |  |  |  |  |  |  |  |  |  |  |  |  |  |  |  |  |  |  |  |  |  |  |  |  |  |  |  |  |  |  |  |  |  |  |  |  |  |  |  |  |  |  |  |  |  |  |  |  | Theo. | | | | | | | | | | | | | | | | | | | | | | | | | | | | | | | | | | | | | | | | | | | | | | | | | | | | | | | | | | | | | | | | | | | | | | | Exp. | | | | | | | | | | | | | | | | | | | | | | | | | | | | | | | | | | | | | | | | | | | | | | | | | | | | | | | | | | | | | | | | | | | | |  |  |  |  |  |  |  |  |  |  |  |  |  |  |  |  |  |  |  |  |  |  |  |  |  |  |  |  |  |  |  |  |  |  |  |  |  |  |  |  |  |  |  |  |  |  |  |  |  |  |  |
| 5f | | | | | | RNA binding protein, putative | | | | | | | | | | | | | | | | | | | | | | | | | | | Glyma05g00330.1 | | | | | | | | | | | | | | | | | | | | | | | | | | | | | | | | | | | 120 | | | | | | | | | | | | | | | | | | | | | | | | | | | | | | 5 | | | | | | | | | | | | | | | | | | | | | | | | | | | | | | | | | | | 13 | | | | | | | | | | | | | | | | | | | | | | | | | | | | | | | | | | | | | | | | | | 446 | | | | | | | | | | | | | | | | | | | | | | | | | | | | | | | | | | | | | | | | | | 50.6/5.39 | | | | | | | | | | | | | | | | | | | | | | | | | | | | | | | | | | | | | | | | | | | | | | | | | | | | | | | | | | | | | | | | | | | | | | |  | | | | | | | | | | | | | | | | | | | | | | | | | | | | | | | | | | | | | | | | | | | | | | | | | | | | | | | | | | | | | | | | | | | | |  | | | | | | | | | | | | | | | | | | | | | | | | | | | | | | | | | | | | | | | | | | | |  |  |  |  |  |  |  |
|  | | | | | | Calc. Mass | | | | | | | | | | | | | | | | | | | | | | | | | | | Observ. Mass | | | | | | | | | | | | | | | | | | | | | | | | | | | | | | | | | | | Da | | | | | | | | | | | | | | | | | | | | | | | | | | | | | | ppm | | | | | | | | | | | | | | | | | | | | | | | | | | | | | | | | | | | start | | | | | | | | | | | | | | | | | | | | | | | | | | | | | | | | | | | | | | | | | | end | | | | | | | | | | | | | | | | | | | | | | | | | | | | | | | | | | | | | | | | | | sequence | | | | | | | | | | | | | | | | | | | | | | | | | | | | | | | | | | | | | | | | | | | | | | | | | | | | | | | | | | | | | | | | | | | | | | | | | | | | | | | | | | | | | | | | | | | | | | | | | | | | | | | | | | | | | | | | | | | | | | | | | | | | | | | | | | | | | | | | | | | | | | | | | | | | | | | | | | | | | | | | | | | | | | | | | | | | | | | | | | | | | | | |  |  |  |  |  |  |  |
|  | | | | | | 1262.5932 | | | | | | | | | | | | | | | | | | | | | | | | | | | 1262.592 | | | | | | | | | | | | | | | | | | | | | | | | | | | | | | | | | | | 0 | | | | | | | | | | | | | | | | | | | | | | | | | | | | | | -1 | | | | | | | | | | | | | | | | | | | | | | | | | | | | | | | | | | | 39 | | | | | | | | | | | | | | | | | | | | | | | | | | | | | | | | | | | | | | | | | | 48 | | | | | | | | | | | | | | | | | | | | | | | | | | | | | | | | | | | | | | | | | | FYQDSSFLTR | | | | | | | | | | | | | | | | | | | | | | | | | | | | | | | | | | | | | | | | | | | | | | | | | | | | | | | | | | | | | | | | | | | | | | | | | | | | | | | | | | | | | | | | | | | | | | | | | | | | | | | | | | | | | | | | | | | | | | | | | | | | | | | | | | | | | | | | | | | | | | | | | | | | | | | | | | | | | | | | | | | | | | | | | | | | | | | | | | | | | | | |  |  |  |  |  |  |  |
|  | | | | | | 1262.5932 | | | | | | | | | | | | | | | | | | | | | | | | | | | 1262.594 | | | | | | | | | | | | | | | | | | | | | | | | | | | | | | | | | | | 0 | | | | | | | | | | | | | | | | | | | | | | | | | | | | | | 1 | | | | | | | | | | | | | | | | | | | | | | | | | | | | | | | | | | | 39 | | | | | | | | | | | | | | | | | | | | | | | | | | | | | | | | | | | | | | | | | | 48 | | | | | | | | | | | | | | | | | | | | | | | | | | | | | | | | | | | | | | | | | | FYQDSSFLTR | | | | | | | | | | | | | | | | | | | | | | | | | | | | | | | | | | | | | | | | | | | | | | | | | | | | | | | | | | | | | | | | | | | | | | | | | | | | | | | | | | | | | | | | | | | | | | | | | | | | | | | | | | | | | | | | | | | | | | | | | | | | | | | | | | | | | | | | | | | | | | | | | | | | | | | | | | | | | | | | | | | | | | | | | | | | | | | | | | | | | | | |  |  |  |  |  |  |  |
|  | | | | | | 2090.979 | | | | | | | | | | | | | | | | | | | | | | | | | | | 2090.978 | | | | | | | | | | | | | | | | | | | | | | | | | | | | | | | | | | | 0 | | | | | | | | | | | | | | | | | | | | | | | | | | | | | | -1 | | | | | | | | | | | | | | | | | | | | | | | | | | | | | | | | | | | 49 | | | | | | | | | | | | | | | | | | | | | | | | | | | | | | | | | | | | | | | | | | 67 | | | | | | | | | | | | | | | | | | | | | | | | | | | | | | | | | | | | | | | | | | SDSNGVMTTVTTVQEIHEK | | | | | | | | | | | | | | | | | | | | | | | | | | | | | | | | | | | | | | | | | | | | | | | | | | | | | | | | | | | | | | | | | | | | | | | | | | | | | | | | | | | | | | | | | | | | | | | | | | | | | | | | | | | | | | | | | | | | | | | | | | | | | | | | | | | | | | | | | | | | | | | | | | | | | | | | | | | | | | | | | | | | | | | | | | | | | | | | | | | | | | | |  |  |  |  |  |  |  |
|  | | | | | | 1441.7242 | | | | | | | | | | | | | | | | | | | | | | | | | | | 1441.723 | | | | | | | | | | | | | | | | | | | | | | | | | | | | | | | | | | | 0 | | | | | | | | | | | | | | | | | | | | | | | | | | | | | | -1 | | | | | | | | | | | | | | | | | | | | | | | | | | | | | | | | | | | 111 | | | | | | | | | | | | | | | | | | | | | | | | | | | | | | | | | | | | | | | | | | 122 | | | | | | | | | | | | | | | | | | | | | | | | | | | | | | | | | | | | | | | | | | FSQTFFLAPQEK | | | | | | | | | | | | | | | | | | | | | | | | | | | | | | | | | | | | | | | | | | | | | | | | | | | | | | | | | | | | | | | | | | | | | | | | | | | | | | | | | | | | | | | | | | | | | | | | | | | | | | | | | | | | | | | | | | | | | | | | | | | | | | | | | | | | | | | | | | | | | | | | | | | | | | | | | | | | | | | | | | | | | | | | | | | | | | | | | | | | | | | |  |  |  |  |  |  |  |
|  | | | | | | 881.468 | | | | | | | | | | | | | | | | | | | | | | | | | | | 881.4686 | | | | | | | | | | | | | | | | | | | | | | | | | | | | | | | | | | | 0 | | | | | | | | | | | | | | | | | | | | | | | | | | | | | | 1 | | | | | | | | | | | | | | | | | | | | | | | | | | | | | | | | | | | 236 | | | | | | | | | | | | | | | | | | | | | | | | | | | | | | | | | | | | | | | | | | 243 | | | | | | | | | | | | | | | | | | | | | | | | | | | | | | | | | | | | | | | | | | SYAAIVMK | | | | | | | | | | | | | | | | | | | | | | | | | | | | | | | | | | | | | | | | | | | | | | | | | | | | | | | | | | | | | | | | | | | | | | | | | | | | | | | | | | | | | | | | | | | | | | | | | | | | | | | | | | | | | | | | | | | | | | | | | | | | | | | | | | | | | | | | | | | | | | | | | | | | | | | | | | | | | | | | | | | | | | | | | | | | | | | | | | | | | | | |  |  |  |  |  |  |  |
|  | | | | | | 1394.7055 | | | | | | | | | | | | | | | | | | | | | | | | | | | 1394.705 | | | | | | | | | | | | | | | | | | | | | | | | | | | | | | | | | | | 0 | | | | | | | | | | | | | | | | | | | | | | | | | | | | | | -1 | | | | | | | | | | | | | | | | | | | | | | | | | | | | | | | | | | | 244 | | | | | | | | | | | | | | | | | | | | | | | | | | | | | | | | | | | | | | | | | | 256 | | | | | | | | | | | | | | | | | | | | | | | | | | | | | | | | | | | | | | | | | | SHVASGHVYVPSR | | | | | | | | | | | | | | | | | | | | | | | | | | | | | | | | | | | | | | | | | | | | | | | | | | | | | | | | | | | | | | | | | | | | | | | | | | | | | | | | | | | | | | | | | | | | | | | | | | | | | | | | | | | | | | | | | | | | | | | | | | | | | | | | | | | | | | | | | | | | | | | | | | | | | | | | | | | | | | | | | | | | | | | | | | | | | | | | | | | | | | | |  |  |  |  |  |  |  |
|  | | | | | | 1394.7055 | | | | | | | | | | | | | | | | | | | | | | | | | | | 1394.707 | | | | | | | | | | | | | | | | | | | | | | | | | | | | | | | | | | | 0 | | | | | | | | | | | | | | | | | | | | | | | | | | | | | | 1 | | | | | | | | | | | | | | | | | | | | | | | | | | | | | | | | | | | 244 | | | | | | | | | | | | | | | | | | | | | | | | | | | | | | | | | | | | | | | | | | 256 | | | | | | | | | | | | | | | | | | | | | | | | | | | | | | | | | | | | | | | | | | SHVASGHVYVPSR | | | | | | | | | | | | | | | | | | | | | | | | | | | | | | | | | | | | | | | | | | | | | | | | | | | | | | | | | | | | | | | | | | | | | | | | | | | | | | | | | | | | | | | | | | | | | | | | | | | | | | | | | | | | | | | | | | | | | | | | | | | | | | | | | | | | | | | | | | | | | | | | | | | | | | | | | | | | | | | | | | | | | | | | | | | | | | | | | | | | | | | |  |  |  |  |  |  |  |
| Spot no. | | | | | | | | | | | | | Homologous protein | | | | | | | | | | | | | | | | | | | | | | | | | | | | Accession no. | | | | | | | | | | | | | | | | | | | | | | | | | | | | | | | | | | Score | | | | | | | | | | | | | | | | | | | | | | | | | | | | | | | | M.P. | | | | | | | | | | | | | | | | | | | | | | | | | | | | | | | | | | | | | Cov. (%) | | | | | | | | | | | | | | | | | | | | | | | | | | | | | | | | | | Blast score | | | | | | | | | | | | | | | | | | | | | | | | | | | | | | | | | | | | | | | Mr (kDa) / pI | | | | | | | | | | | | | | | | | | | | | | | | | | | | | | | | | | | | | | | | | | | | | | | | | | | | | | | | | | | | | | | | | | | | | | | | | | | | | | | | | | | | | | | | | | Ratio | | | | | | | | | | | | | | | | | | | | | | | | | | | | | | | | | | | | | | | | | | | | | | | | | | | | | | | | | | | | | | | | | | | | | | | | | | | | | | | | | | | | | | | | | | | | | |  |  |  |  |  |  |  |
|  |  |  |  |  |  |  |  |  |  |  |  |  |  |  |  |  |  |  |  |  |  |  |  |  |  |  |  |  |  |  |  |  |  |  |  |  |  |  |  |  |  |  |  |  |  |  |  |  |  |  |  |  |  |  |  |  |  |  |  |  |  |  |  |  |  |  |  |  |  |  |  |  |  |  |  |  |  |  |  |  |  |  |  |  |  |  |  |  |  |  |  |  |  |  |  |  |  |  |  |  |  |  |  |  |  |  |  |  |  |  |  |  |  |  |  |  |  |  |  |  |  |  |  |  |  |  |  |  |  |  |  |  |  |  |  |  |  |  |  |  |  |  |  |  |  |  |  |  |  |  |  |  |  |  |  |  |  |  |  |  |  |  |  |  |  |  |  |  |  |  |  |  |  |  |  |  |  |  |  |  |  |  |  |  |  |  |  |  |  |  |  |  |  |  |  |  |  |  |  |  |  |  |  |  |  |  |  |  |  |  |  |  |  |  |  |  | Theo. | | | | | | | | | | | | | | | | | | | | | | | | | | | | | | | | | Exp. | | | | | | | | | | | | | | | | | | | | | | | | | | | | | | | | | | | | | | | | | | | | | | | | | | | | | | | | |  |  |  |  |  |  |  |  |  |  |  |  |  |  |  |  |  |  |  |  |  |  |  |  |  |  |  |  |  |  |  |  |  |  |  |  |  |  |  |  |  |  |  |  |  |  |  |  |  |  |  |  |  |  |  |  |  |  |  |  |  |  |  |  |  |  |  |  |  |  |  |  |  |  |  |  |  |  |  |  |  |  |  |  |  |  |  |  |  |  |  |  |  |  |  |  |  |  |  |  |  |
| 1d | | | | | | | | | | | | | enolase | | | | | | | | | | | | | | | | | | | | | | | | | | | | Glyma03g34830.1 | | | | | | | | | | | | | | | | | | | | | | | | | | | | | | | | | | 198 | | | | | | | | | | | | | | | | | | | | | | | | | | | | | | | | 3 | | | | | | | | | | | | | | | | | | | | | | | | | | | | | | | | | | | | | 12 | | | | | | | | | | | | | | | | | | | | | | | | | | | | | | | | | | 863 | | | | | | | | | | | | | | | | | | | | | | | | | | | | | | | | | | | | | | | 48.1/5.49 | | | | | | | | | | | | | | | | | | | | | | | | | | | | | | | | |  | | | | | | | | | | | | | | | | | | | | | | | | | | | | | | | | | | | | | | | | | | | | | | | | | | | | | | | | |  | | | | | | | | | | | | | | | | | | | | | | | | | | | | | | | | | | | | | | | | | | | | | | | | | | | | | | | | | | | | | | | | | | | | | | | | | | | | | | | | | | | | | | | | | | | | | |  |  |  |  |  |  |  |
|  | | | | | | | | | | | | | Calc. Mass | | | | | | | | | | | | | | | | | | | | | | | | | | | | Observ. Mass | | | | | | | | | | | | | | | | | | | | | | | | | | | | | | | | | | Da | | | | | | | | | | | | | | | | | | | | | | | | | | | | | | | | ppm | | | | | | | | | | | | | | | | | | | | | | | | | | | | | | | | | | | | | start | | | | | | | | | | | | | | | | | | | | | | | | | | | | | | | | | | end | | | | | | | | | | | | | | | | | | | | | | | | | | | | | | | | | | | | | | | sequence | | | | | | | | | | | | | | | | | | | | | | | | | | | | | | | | | | | | | | | | | | | | | | | | | | | | | | | | | | | | | | | | | | | | | | | | | | | | | | | | | | | | | | | | | | | | | | | | | | | | | | | | | | | | | | | | | | | | | | | | | | | | | | | | | | | | | | | | | | | | | | | | | | | | | | | | | | | | | | | | | | | | | | | | | | | | | | | | | | | | | | | |  |  |  |  |  |  |  |
|  | | | | | | | | | | | | | 1937.8789 | | | | | | | | | | | | | | | | | | | | | | | | | | | | 1937.877 | | | | | | | | | | | | | | | | | | | | | | | | | | | | | | | | | | 0 | | | | | | | | | | | | | | | | | | | | | | | | | | | | | | | | -1 | | | | | | | | | | | | | | | | | | | | | | | | | | | | | | | | | | | | | 17 | | | | | | | | | | | | | | | | | | | | | | | | | | | | | | | 34 | | | | | | | | | | | | | | | | | | | | | | | | | | | | | | | | | | | | | | | | | | GNPTVEVDLTCSDGTFAR | | | | | | | | | | | | | | | | | | | | | | | | | | | | | | | | | | | | | | | | | | | | | | | | | | | | | | | | | | | | | | | | | | | | | | | | | | | | | | | | | | | | | | | | | | | | | | | | | | | | | | | | | | | | | | | | | | | | | | | | | | | | | | | | | | | | | | | | | | | | | | | | | | | | | | | | | | | | | | | | | | | | | | | | | | | | | | | | | | | | | | | |  |  |  |  |  |  |  |
|  | | | | | | | | | | | | | 1600.842 | | | | | | | | | | | | | | | | | | | | | | | | | | | | 1600.842 | | | | | | | | | | | | | | | | | | | | | | | | | | | | | | | | | | 0 | | | | | | | | | | | | | | | | | | | | | | | | | | | | | | | | 0 | | | | | | | | | | | | | | | | | | | | | | | | | | | | | | | | | | | | | 353 | | | | | | | | | | | | | | | | | | | | | | | | | | | | | | | 367 | | | | | | | | | | | | | | | | | | | | | | | | | | | | | | | | | | | | | | | | | | VNQIGSVTESIEAVR | | | | | | | | | | | | | | | | | | | | | | | | | | | | | | | | | | | | | | | | | | | | | | | | | | | | | | | | | | | | | | | | | | | | | | | | | | | | | | | | | | | | | | | | | | | | | | | | | | | | | | | | | | | | | | | | | | | | | | | | | | | | | | | | | | | | | | | | | | | | | | | | | | | | | | | | | | | | | | | | | | | | | | | | | | | | | | | | | | | | | | | |  |  |  |  |  |  |  |
|  | | | | | | | | | | | | | 2251.122 | | | | | | | | | | | | | | | | | | | | | | | | | | | | 2251.121 | | | | | | | | | | | | | | | | | | | | | | | | | | | | | | | | | | 0 | | | | | | | | | | | | | | | | | | | | | | | | | | | | | | | | 0 | | | | | | | | | | | | | | | | | | | | | | | | | | | | | | | | | | | | | 382 | | | | | | | | | | | | | | | | | | | | | | | | | | | | | | | 403 | | | | | | | | | | | | | | | | | | | | | | | | | | | | | | | | | | | | | | | | | | SGETEDTFIADLSVGLATGQIK | | | | | | | | | | | | | | | | | | | | | | | | | | | | | | | | | | | | | | | | | | | | | | | | | | | | | | | | | | | | | | | | | | | | | | | | | | | | | | | | | | | | | | | | | | | | | | | | | | | | | | | | | | | | | | | | | | | | | | | | | | | | | | | | | | | | | | | | | | | | | | | | | | | | | | | | | | | | | | | | | | | | | | | | | | | | | | | | | | | | | | | |  |  |  |  |  |  |  |
| Spot no. | | | Homologous protein | | | | | | | | | | | | | | | | | | Accession no. | | | | | | | | | | | | | | | | | | | | | | | | | | | | | Score | | | | | | | | | | | | | | | | | | | | | | | | | | | | | | | M.P. | | | | | | | | | | | | | | | | | | | | | | | | | | | | | | | | | | Cov. (%) | | | | | | | | | | | | | | | | | | | | | | | | | | | | | | | | | | | | | | | Blast score | | | | | | | | | | | | | | | | | | | | | | | | | | | | | | | | | | | | | | | | | | | | | Mr (kDa) / pI | | | | | | | | | | | | | | | | | | | | | | | | | | | | | | | | | | | | | | | | | | | | | | | | | | | | | | | | | | | | | | | | | | | | | | | | | | | | | | | | | | | | | | | | | | | | | | | | | | | | | | Ratio | | | | | | | | | | | | | | | | | | | | | | | | | | | | | | | | | | | | | | | | | | | | | | | | | | | | | | | | | | | | | | | | | | | | | | |  |  |  |  |  |  |  |  |  |  |  |  |  |  |  |  |  |  |  |  |  |  |  |  |  |  |  |  |  |  |  |  |  |  |  |  |
|  |  |  |  |  |  |  |  |  |  |  |  |  |  |  |  |  |  |  |  |  |  |  |  |  |  |  |  |  |  |  |  |  |  |  |  |  |  |  |  |  |  |  |  |  |  |  |  |  |  |  |  |  |  |  |  |  |  |  |  |  |  |  |  |  |  |  |  |  |  |  |  |  |  |  |  |  |  |  |  |  |  |  |  |  |  |  |  |  |  |  |  |  |  |  |  |  |  |  |  |  |  |  |  |  |  |  |  |  |  |  |  |  |  |  |  |  |  |  |  |  |  |  |  |  |  |  |  |  |  |  |  |  |  |  |  |  |  |  |  |  |  |  |  |  |  |  |  |  |  |  |  |  |  |  |  |  |  |  |  |  |  |  |  |  |  |  |  |  |  |  |  |  |  |  |  |  |  |  |  |  |  |  |  |  |  |  |  |  |  |  |  |  |  |  |  |  |  |  | Theo. | | | | | | | | | | | | | | | | | | | | | | | | | | | | | | | | | | | | | | | | | Exp. | | | | | | | | | | | | | | | | | | | | | | | | | | | | | | | | | | | | | | | | | | | | | | | | | | | | | | | | | | | | |  |  |  |  |  |  |  |  |  |  |  |  |  |  |  |  |  |  |  |  |  |  |  |  |  |  |  |  |  |  |  |  |  |  |  |  |  |  |  |  |  |  |  |  |  |  |  |  |  |  |  |  |  |  |  |  |  |  |  |  |  |  |  |  |  |  |  |  |  |  |  |  |  |  |  |  |  |  |  |  |  |  |  |  |  |  |  |  |  |  |  |  |  |  |  |  |  |  |  |  |  |  |  |  |  |  |  |
| 1e | | | uncharacterized protein LOC100305855 precursor | | | | | | | | | | | | | | | | | | Glyma01g10900.1 | | | | | | | | | | | | | | | | | | | | | | | | | | | | | 139 | | | | | | | | | | | | | | | | | | | | | | | | | | | | | | | 2 | | | | | | | | | | | | | | | | | | | | | | | | | | | | | | | | | | 10 | | | | | | | | | | | | | | | | | | | | | | | | | | | | | | | | | | | | | | | 385 | | | | | | | | | | | | | | | | | | | | | | | | | | | | | | | | | | | | | | | | | | | | | 22.9/4.85 | | | | | | | | | | | | | | | | | | | | | | | | | | | | | | | | | | | | | | | | |  | | | | | | | | | | | | | | | | | | | | | | | | | | | | | | | | | | | | | | | | | | | | | | | | | | | | | | | | | | | | |  | | | | | | | | | | | | | | | | | | | | | | | | | | | | | | | | | | | | | | | | | | | | | | | | | | | | | | | | | | | | | | | | | | | | | | |  |  |  |  |  |  |  |  |  |  |  |  |  |  |  |  |  |  |  |  |  |  |  |  |  |  |  |  |  |  |  |  |  |  |  |  |
|  | | | Calc. Mass | | | | | | | | | | | | | | | | | | Observ. Mass | | | | | | | | | | | | | | | | | | | | | | | | | | | | | Da | | | | | | | | | | | | | | | | | | | | | | | | | | | | | | | ppm | | | | | | | | | | | | | | | | | | | | | | | | | | | | | | | | | | start | | | | | | | | | | | | | | | | | | | | | | | | | | | | | | | | | | | | | | | end | | | | | | | | | | | | | | | | | | | | | | | | | | | | | | | | | | | | | | | | | | | | | sequence | | | | | | | | | | | | | | | | | | | | | | | | | | | | | | | | | | | | | | | | | | | | | | | | | | | | | | | | | | | | | | | | | | | | | | | | | | | | | | | | | | | | | | | | | | | | | | | | | | | | | | | | | | | | | | | | | | | | | | | | | | | | | | | | | | | | | | | | | | | | | | | | | | | | | | | | | | | | | | | | | | | | | | | | | | | | |  |  |  |  |  |  |  |  |  |  |  |  |  |  |  |  |  |  |  |  |  |  |  |  |  |  |  |  |  |  |  |  |  |  |  |  |
|  | | | 1447.6766 | | | | | | | | | | | | | | | | | | 1447.676 | | | | | | | | | | | | | | | | | | | | | | | | | | | | | 0 | | | | | | | | | | | | | | | | | | | | | | | | | | | | | | | 0 | | | | | | | | | | | | | | | | | | | | | | | | | | | | | | | | | | 156 | | | | | | | | | | | | | | | | | | | | | | | | | | | | | | | | | | | | | | | 167 | | | | | | | | | | | | | | | | | | | | | | | | | | | | | | | | | | | | | | | | | | | | | LVFCPQQAEDNK | | | | | | | | | | | | | | | | | | | | | | | | | | | | | | | | | | | | | | | | | | | | | | | | | | | | | | | | | | | | | | | | | | | | | | | | | | | | | | | | | | | | | | | | | | | | | | | | | | | | | | | | | | | | | | | | | | | | | | | | | | | | | | | | | | | | | | | | | | | | | | | | | | | | | | | | | | | | | | | | | | | | | | | | | | | | |  |  |  |  |  |  |  |  |  |  |  |  |  |  |  |  |  |  |  |  |  |  |  |  |  |  |  |  |  |  |  |  |  |  |  |  |
|  | | | 1199.7026 | | | | | | | | | | | | | | | | | | 1199.702 | | | | | | | | | | | | | | | | | | | | | | | | | | | | | 0 | | | | | | | | | | | | | | | | | | | | | | | | | | | | | | | -1 | | | | | | | | | | | | | | | | | | | | | | | | | | | | | | | | | | 188 | | | | | | | | | | | | | | | | | | | | | | | | | | | | | | | | | | | | | | | 197 | | | | | | | | | | | | | | | | | | | | | | | | | | | | | | | | | | | | | | | | | | | | | NKPLVVQFQK | | | | | | | | | | | | | | | | | | | | | | | | | | | | | | | | | | | | | | | | | | | | | | | | | | | | | | | | | | | | | | | | | | | | | | | | | | | | | | | | | | | | | | | | | | | | | | | | | | | | | | | | | | | | | | | | | | | | | | | | | | | | | | | | | | | | | | | | | | | | | | | | | | | | | | | | | | | | | | | | | | | | | | | | | | | | |  |  |  |  |  |  |  |  |  |  |  |  |  |  |  |  |  |  |  |  |  |  |  |  |  |  |  |  |  |  |  |  |  |  |  |  |
|  | | | 1199.7026 | | | | | | | | | | | | | | | | | | 1199.702 | | | | | | | | | | | | | | | | | | | | | | | | | | | | | 0 | | | | | | | | | | | | | | | | | | | | | | | | | | | | | | | -1 | | | | | | | | | | | | | | | | | | | | | | | | | | | | | | | | | | 188 | | | | | | | | | | | | | | | | | | | | | | | | | | | | | | | | | | | | | | | 197 | | | | | | | | | | | | | | | | | | | | | | | | | | | | | | | | | | | | | | | | | | | | | NKPLVVQFQK | | | | | | | | | | | | | | | | | | | | | | | | | | | | | | | | | | | | | | | | | | | | | | | | | | | | | | | | | | | | | | | | | | | | | | | | | | | | | | | | | | | | | | | | | | | | | | | | | | | | | | | | | | | | | | | | | | | | | | | | | | | | | | | | | | | | | | | | | | | | | | | | | | | | | | | | | | | | | | | | | | | | | | | | | | | | |  |  |  |  |  |  |  |  |  |  |  |  |  |  |  |  |  |  |  |  |  |  |  |  |  |  |  |  |  |  |  |  |  |  |  |  |
|  | | | 1199.7026 | | | | | | | | | | | | | | | | | | 1199.702 | | | | | | | | | | | | | | | | | | | | | | | | | | | | | 0 | | | | | | | | | | | | | | | | | | | | | | | | | | | | | | | 0 | | | | | | | | | | | | | | | | | | | | | | | | | | | | | | | | | | 188 | | | | | | | | | | | | | | | | | | | | | | | | | | | | | | | | | | | | | | | 197 | | | | | | | | | | | | | | | | | | | | | | | | | | | | | | | | | | | | | | | | | | | | | NKPLVVQFQK | | | | | | | | | | | | | | | | | | | | | | | | | | | | | | | | | | | | | | | | | | | | | | | | | | | | | | | | | | | | | | | | | | | | | | | | | | | | | | | | | | | | | | | | | | | | | | | | | | | | | | | | | | | | | | | | | | | | | | | | | | | | | | | | | | | | | | | | | | | | | | | | | | | | | | | | | | | | | | | | | | | | | | | | | | | | |  |  |  |  |  |  |  |  |  |  |  |  |  |  |  |  |  |  |  |  |  |  |  |  |  |  |  |  |  |  |  |  |  |  |  |  |
|  | | | 1199.7026 | | | | | | | | | | | | | | | | | | 1199.702 | | | | | | | | | | | | | | | | | | | | | | | | | | | | | 0 | | | | | | | | | | | | | | | | | | | | | | | | | | | | | | | 0 | | | | | | | | | | | | | | | | | | | | | | | | | | | | | | | | | | 188 | | | | | | | | | | | | | | | | | | | | | | | | | | | | | | | | | | | | | | | 197 | | | | | | | | | | | | | | | | | | | | | | | | | | | | | | | | | | | | | | | | | | | | | NKPLVVQFQK | | | | | | | | | | | | | | | | | | | | | | | | | | | | | | | | | | | | | | | | | | | | | | | | | | | | | | | | | | | | | | | | | | | | | | | | | | | | | | | | | | | | | | | | | | | | | | | | | | | | | | | | | | | | | | | | | | | | | | | | | | | | | | | | | | | | | | | | | | | | | | | | | | | | | | | | | | | | | | | | | | | | | | | | | | | | |  |  |  |  |  |  |  |  |  |  |  |  |  |  |  |  |  |  |  |  |  |  |  |  |  |  |  |  |  |  |  |  |  |  |  |  |
|  | | | 1199.7026 | | | | | | | | | | | | | | | | | | 1199.703 | | | | | | | | | | | | | | | | | | | | | | | | | | | | | 0 | | | | | | | | | | | | | | | | | | | | | | | | | | | | | | | 0 | | | | | | | | | | | | | | | | | | | | | | | | | | | | | | | | | | 188 | | | | | | | | | | | | | | | | | | | | | | | | | | | | | | | | | | | | | | | 197 | | | | | | | | | | | | | | | | | | | | | | | | | | | | | | | | | | | | | | | | | | | | | NKPLVVQFQK | | | | | | | | | | | | | | | | | | | | | | | | | | | | | | | | | | | | | | | | | | | | | | | | | | | | | | | | | | | | | | | | | | | | | | | | | | | | | | | | | | | | | | | | | | | | | | | | | | | | | | | | | | | | | | | | | | | | | | | | | | | | | | | | | | | | | | | | | | | | | | | | | | | | | | | | | | | | | | | | | | | | | | | | | | | | |  |  |  |  |  |  |  |  |  |  |  |  |  |  |  |  |  |  |  |  |  |  |  |  |  |  |  |  |  |  |  |  |  |  |  |  |
|  | | | 1199.7026 | | | | | | | | | | | | | | | | | | 1199.703 | | | | | | | | | | | | | | | | | | | | | | | | | | | | | 0 | | | | | | | | | | | | | | | | | | | | | | | | | | | | | | | 0 | | | | | | | | | | | | | | | | | | | | | | | | | | | | | | | | | | 188 | | | | | | | | | | | | | | | | | | | | | | | | | | | | | | | | | | | | | | | 197 | | | | | | | | | | | | | | | | | | | | | | | | | | | | | | | | | | | | | | | | | | | | | NKPLVVQFQK | | | | | | | | | | | | | | | | | | | | | | | | | | | | | | | | | | | | | | | | | | | | | | | | | | | | | | | | | | | | | | | | | | | | | | | | | | | | | | | | | | | | | | | | | | | | | | | | | | | | | | | | | | | | | | | | | | | | | | | | | | | | | | | | | | | | | | | | | | | | | | | | | | | | | | | | | | | | | | | | | | | | | | | | | | | | |  |  |  |  |  |  |  |  |  |  |  |  |  |  |  |  |  |  |  |  |  |  |  |  |  |  |  |  |  |  |  |  |  |  |  |  |
|  | | | 1199.7026 | | | | | | | | | | | | | | | | | | 1199.703 | | | | | | | | | | | | | | | | | | | | | | | | | | | | | 0 | | | | | | | | | | | | | | | | | | | | | | | | | | | | | | | 0 | | | | | | | | | | | | | | | | | | | | | | | | | | | | | | | | | | 188 | | | | | | | | | | | | | | | | | | | | | | | | | | | | | | | | | | | | | | | 197 | | | | | | | | | | | | | | | | | | | | | | | | | | | | | | | | | | | | | | | | | | | | | NKPLVVQFQK | | | | | | | | | | | | | | | | | | | | | | | | | | | | | | | | | | | | | | | | | | | | | | | | | | | | | | | | | | | | | | | | | | | | | | | | | | | | | | | | | | | | | | | | | | | | | | | | | | | | | | | | | | | | | | | | | | | | | | | | | | | | | | | | | | | | | | | | | | | | | | | | | | | | | | | | | | | | | | | | | | | | | | | | | | | | |  |  |  |  |  |  |  |  |  |  |  |  |  |  |  |  |  |  |  |  |  |  |  |  |  |  |  |  |  |  |  |  |  |  |  |  |
|  | | | 1199.7026 | | | | | | | | | | | | | | | | | | 1199.703 | | | | | | | | | | | | | | | | | | | | | | | | | | | | | 0 | | | | | | | | | | | | | | | | | | | | | | | | | | | | | | | 0 | | | | | | | | | | | | | | | | | | | | | | | | | | | | | | | | | | 188 | | | | | | | | | | | | | | | | | | | | | | | | | | | | | | | | | | | | | | | 197 | | | | | | | | | | | | | | | | | | | | | | | | | | | | | | | | | | | | | | | | | | | | | NKPLVVQFQK | | | | | | | | | | | | | | | | | | | | | | | | | | | | | | | | | | | | | | | | | | | | | | | | | | | | | | | | | | | | | | | | | | | | | | | | | | | | | | | | | | | | | | | | | | | | | | | | | | | | | | | | | | | | | | | | | | | | | | | | | | | | | | | | | | | | | | | | | | | | | | | | | | | | | | | | | | | | | | | | | | | | | | | | | | | | |  |  |  |  |  |  |  |  |  |  |  |  |  |  |  |  |  |  |  |  |  |  |  |  |  |  |  |  |  |  |  |  |  |  |  |  |
|  | | | 1199.7026 | | | | | | | | | | | | | | | | | | 1199.703 | | | | | | | | | | | | | | | | | | | | | | | | | | | | | 0 | | | | | | | | | | | | | | | | | | | | | | | | | | | | | | | 1 | | | | | | | | | | | | | | | | | | | | | | | | | | | | | | | | | | 188 | | | | | | | | | | | | | | | | | | | | | | | | | | | | | | | | | | | | | | | 197 | | | | | | | | | | | | | | | | | | | | | | | | | | | | | | | | | | | | | | | | | | | | | NKPLVVQFQK | | | | | | | | | | | | | | | | | | | | | | | | | | | | | | | | | | | | | | | | | | | | | | | | | | | | | | | | | | | | | | | | | | | | | | | | | | | | | | | | | | | | | | | | | | | | | | | | | | | | | | | | | | | | | | | | | | | | | | | | | | | | | | | | | | | | | | | | | | | | | | | | | | | | | | | | | | | | | | | | | | | | | | | | | | | | |  |  |  |  |  |  |  |  |  |  |  |  |  |  |  |  |  |  |  |  |  |  |  |  |  |  |  |  |  |  |  |  |  |  |  |  |
| Spot no. | | | Homologous protein | | | | | | | | | | | | | | | | | | Accession no. | | | | | | | | | | | | | | | | | | | | | | | | | | | | | Score | | | | | | | | | | | | | | | | | | | | | | | | | | | | | | | M.P. | | | | | | | | | | | | | | | | | | | | | | | | | | | | | | | | | | Cov. (%) | | | | | | | | | | | | | | | | | | | | | | | | | | | | | | | | | | | | | | | Blast score | | | | | | | | | | | | | | | | | | | | | | | | | | | | | | | | | | | | | | | | | | | | | Mr (kDa) / pI | | | | | | | | | | | | | | | | | | | | | | | | | | | | | | | | | | | | | | | | | | | | | | | | | | | | | | | | | | | | | | | | | | | | | | | | | | | | | | | | | | | | | | | | | | | | | | | | | | | | | | | | | | | | | | | | | | Ratio | | | | | | | | | | | | | | | | | | | | | | | | | | | | | | | | | | | | | | | | | | | | | | | | | | | | | | | | | | | | | | | | | | |  |  |  |  |  |  |  |  |  |  |  |  |  |  |  |  |  |  |  |  |  |  |  |  |  |  |  |  |
|  |  |  |  |  |  |  |  |  |  |  |  |  |  |  |  |  |  |  |  |  |  |  |  |  |  |  |  |  |  |  |  |  |  |  |  |  |  |  |  |  |  |  |  |  |  |  |  |  |  |  |  |  |  |  |  |  |  |  |  |  |  |  |  |  |  |  |  |  |  |  |  |  |  |  |  |  |  |  |  |  |  |  |  |  |  |  |  |  |  |  |  |  |  |  |  |  |  |  |  |  |  |  |  |  |  |  |  |  |  |  |  |  |  |  |  |  |  |  |  |  |  |  |  |  |  |  |  |  |  |  |  |  |  |  |  |  |  |  |  |  |  |  |  |  |  |  |  |  |  |  |  |  |  |  |  |  |  |  |  |  |  |  |  |  |  |  |  |  |  |  |  |  |  |  |  |  |  |  |  |  |  |  |  |  |  |  |  |  |  |  |  |  |  |  |  |  |  |  | Theo. | | | | | | | | | | | | | | | | | | | | | | | | | | | | | | | | | | | | | | | | | | | | | | | | | | | | | | | | | | | | | | | | | | | | | | | | | | | Exp. | | | | | | | | | | | | | | | | | | | | | | | | | | | | | | | | | | | | | | |  |  |  |  |  |  |  |  |  |  |  |  |  |  |  |  |  |  |  |  |  |  |  |  |  |  |  |  |  |  |  |  |  |  |  |  |  |  |  |  |  |  |  |  |  |  |  |  |  |  |  |  |  |  |  |  |  |  |  |  |  |  |  |  |  |  |  |  |  |  |  |  |  |  |  |  |  |  |  |  |  |  |  |  |  |  |  |  |  |  |  |  |  |  |  |
| 1f | | | uncharacterized protein LOC100527911 | | | | | | | | | | | | | | | | | | Glyma02g11540.1 | | | | | | | | | | | | | | | | | | | | | | | | | | | | | 129 | | | | | | | | | | | | | | | | | | | | | | | | | | | | | | | 7 | | | | | | | | | | | | | | | | | | | | | | | | | | | | | | | | | | 37 | | | | | | | | | | | | | | | | | | | | | | | | | | | | | | | | | | | | | | | 276 | | | | | | | | | | | | | | | | | | | | | | | | | | | | | | | | | | | | | | | | | | | | | 18.1/10.21 | | | | | | | | | | | | | | | | | | | | | | | | | | | | | | | | | | | | | | | | | | | | | | | | | | | | | | | | | | | | | | | | | | | | | | | | | | |  | | | | | | | | | | | | | | | | | | | | | | | | | | | | | | | | | | | | | | |  | | | | | | | | | | | | | | | | | | | | | | | | | | | | | | | | | | | | | | | | | | | | | | | | | | | | | | | | | | | | | | | | | | |  |  |  |  |  |  |  |  |  |  |  |  |  |  |  |  |  |  |  |  |  |  |  |  |  |  |  |  |
|  | | | Calc. Mass | | | | | | | | | | | | | | | | | | Observ. Mass | | | | | | | | | | | | | | | | | | | | | | | | | | | | | Da | | | | | | | | | | | | | | | | | | | | | | | | | | | | | | | ppm | | | | | | | | | | | | | | | | | | | | | | | | | | | | | | | | | | start | | | | | | | | | | | | | | | | | | | | | | | | | | | | | | | | | | | | | | | end | | | | | | | | | | | | | | | | | | | | | | | | | | | | | | | | | | | | | | | | | | | | | sequence | | | | | | | | | | | | | | | | | | | | | | | | | | | | | | | | | | | | | | | | | | | | | | | | | | | | | | | | | | | | | | | | | | | | | | | | | | | | | | | | | | | | | | | | | | | | | | | | | | | | | | | | | | | | | | | | | | | | | | | | | | | | | | | | | | | | | | | | | | | | | | | | | | | | | | | | | | | | | | | | | | | | | | | | | | | | |  |  |  |  |  |  |  |  |  |  |  |  |  |  |  |  |  |  |  |  |  |  |  |  |  |  |  |  |  |  |  |  |  |  |  |  |
|  | | | 1011.5059 | | | | | | | | | | | | | | | | | | 1011.507 | | | | | | | | | | | | | | | | | | | | | | | | | | | | | 0 | | | | | | | | | | | | | | | | | | | | | | | | | | | | | | | 1 | | | | | | | | | | | | | | | | | | | | | | | | | | | | | | | | | | 28 | | | | | | | | | | | | | | | | | | | | | | | | | | | | | | | | | | | | | | | 36 | | | | | | | | | | | | | | | | | | | | | | | | | | | | | | | | | | | | | | | | | | | | | TAVAVTYCK | | | | | | | | | | | | | | | | | | | | | | | | | | | | | | | | | | | | | | | | | | | | | | | | | | | | | | | | | | | | | | | | | | | | | | | | | | | | | | | | | | | | | | | | | | | | | | | | | | | | | | | | | | | | | | | | | | | | | | | | | | | | | | | | | | | | | | | | | | | | | | | | | | | | | | | | | | | | | | | | | | | | | | | | | | | | |  |  |  |  |  |  |  |  |  |  |  |  |  |  |  |  |  |  |  |  |  |  |  |  |  |  |  |  |  |  |  |  |  |  |  |  |
|  | | | 1750.9287 | | | | | | | | | | | | | | | | | | 1750.927 | | | | | | | | | | | | | | | | | | | | | | | | | | | | | 0 | | | | | | | | | | | | | | | | | | | | | | | | | | | | | | | -1 | | | | | | | | | | | | | | | | | | | | | | | | | | | | | | | | | | 44 | | | | | | | | | | | | | | | | | | | | | | | | | | | | | | | | | | | | | | | 58 | | | | | | | | | | | | | | | | | | | | | | | | | | | | | | | | | | | | | | | | | | | | | INGCPIELVEPEILR | | | | | | | | | | | | | | | | | | | | | | | | | | | | | | | | | | | | | | | | | | | | | | | | | | | | | | | | | | | | | | | | | | | | | | | | | | | | | | | | | | | | | | | | | | | | | | | | | | | | | | | | | | | | | | | | | | | | | | | | | | | | | | | | | | | | | | | | | | | | | | | | | | | | | | | | | | | | | | | | | | | | | | | | | | | | |  |  |  |  |  |  |  |  |  |  |  |  |  |  |  |  |  |  |  |  |  |  |  |  |  |  |  |  |  |  |  |  |  |  |  |  |
|  | | | 1750.9287 | | | | | | | | | | | | | | | | | | 1750.929 | | | | | | | | | | | | | | | | | | | | | | | | | | | | | 0 | | | | | | | | | | | | | | | | | | | | | | | | | | | | | | | 0 | | | | | | | | | | | | | | | | | | | | | | | | | | | | | | | | | | 44 | | | | | | | | | | | | | | | | | | | | | | | | | | | | | | | | | | | | | | | 58 | | | | | | | | | | | | | | | | | | | | | | | | | | | | | | | | | | | | | | | | | | | | | INGCPIELVEPEILR | | | | | | | | | | | | | | | | | | | | | | | | | | | | | | | | | | | | | | | | | | | | | | | | | | | | | | | | | | | | | | | | | | | | | | | | | | | | | | | | | | | | | | | | | | | | | | | | | | | | | | | | | | | | | | | | | | | | | | | | | | | | | | | | | | | | | | | | | | | | | | | | | | | | | | | | | | | | | | | | | | | | | | | | | | | | |  |  |  |  |  |  |  |  |  |  |  |  |  |  |  |  |  |  |  |  |  |  |  |  |  |  |  |  |  |  |  |  |  |  |  |  |
|  | | | 1750.9287 | | | | | | | | | | | | | | | | | | 1750.932 | | | | | | | | | | | | | | | | | | | | | | | | | | | | | 0 | | | | | | | | | | | | | | | | | | | | | | | | | | | | | | | 2 | | | | | | | | | | | | | | | | | | | | | | | | | | | | | | | | | | 44 | | | | | | | | | | | | | | | | | | | | | | | | | | | | | | | | | | | | | | | 58 | | | | | | | | | | | | | | | | | | | | | | | | | | | | | | | | | | | | | | | | | | | | | INGCPIELVEPEILR | | | | | | | | | | | | | | | | | | | | | | | | | | | | | | | | | | | | | | | | | | | | | | | | | | | | | | | | | | | | | | | | | | | | | | | | | | | | | | | | | | | | | | | | | | | | | | | | | | | | | | | | | | | | | | | | | | | | | | | | | | | | | | | | | | | | | | | | | | | | | | | | | | | | | | | | | | | | | | | | | | | | | | | | | | | | |  |  |  |  |  |  |  |  |  |  |  |  |  |  |  |  |  |  |  |  |  |  |  |  |  |  |  |  |  |  |  |  |  |  |  |  |
|  | | | 1374.8275 | | | | | | | | | | | | | | | | | | 1374.823 | | | | | | | | | | | | | | | | | | | | | | | | | | | | | 0 | | | | | | | | | | | | | | | | | | | | | | | | | | | | | | | -4 | | | | | | | | | | | | | | | | | | | | | | | | | | | | | | | | | | 59 | | | | | | | | | | | | | | | | | | | | | | | | | | | | | | | | | | | | | | | 70 | | | | | | | | | | | | | | | | | | | | | | | | | | | | | | | | | | | | | | | | | | | | | FKAFEPILLLGK | | | | | | | | | | | | | | | | | | | | | | | | | | | | | | | | | | | | | | | | | | | | | | | | | | | | | | | | | | | | | | | | | | | | | | | | | | | | | | | | | | | | | | | | | | | | | | | | | | | | | | | | | | | | | | | | | | | | | | | | | | | | | | | | | | | | | | | | | | | | | | | | | | | | | | | | | | | | | | | | | | | | | | | | | | | | |  |  |  |  |  |  |  |  |  |  |  |  |  |  |  |  |  |  |  |  |  |  |  |  |  |  |  |  |  |  |  |  |  |  |  |  |
|  | | | 1099.6641 | | | | | | | | | | | | | | | | | | 1099.663 | | | | | | | | | | | | | | | | | | | | | | | | | | | | | 0 | | | | | | | | | | | | | | | | | | | | | | | | | | | | | | | -1 | | | | | | | | | | | | | | | | | | | | | | | | | | | | | | | | | | 61 | | | | | | | | | | | | | | | | | | | | | | | | | | | | | | | | | | | | | | | 70 | | | | | | | | | | | | | | | | | | | | | | | | | | | | | | | | | | | | | | | | | | | | | AFEPILLLGK | | | | | | | | | | | | | | | | | | | | | | | | | | | | | | | | | | | | | | | | | | | | | | | | | | | | | | | | | | | | | | | | | | | | | | | | | | | | | | | | | | | | | | | | | | | | | | | | | | | | | | | | | | | | | | | | | | | | | | | | | | | | | | | | | | | | | | | | | | | | | | | | | | | | | | | | | | | | | | | | | | | | | | | | | | | | |  |  |  |  |  |  |  |  |  |  |  |  |  |  |  |  |  |  |  |  |  |  |  |  |  |  |  |  |  |  |  |  |  |  |  |  |
|  | | | 1099.6641 | | | | | | | | | | | | | | | | | | 1099.664 | | | | | | | | | | | | | | | | | | | | | | | | | | | | | 0 | | | | | | | | | | | | | | | | | | | | | | | | | | | | | | | 0 | | | | | | | | | | | | | | | | | | | | | | | | | | | | | | | | | | 61 | | | | | | | | | | | | | | | | | | | | | | | | | | | | | | | | | | | | | | | 70 | | | | | | | | | | | | | | | | | | | | | | | | | | | | | | | | | | | | | | | | | | | | | AFEPILLLGK | | | | | | | | | | | | | | | | | | | | | | | | | | | | | | | | | | | | | | | | | | | | | | | | | | | | | | | | | | | | | | | | | | | | | | | | | | | | | | | | | | | | | | | | | | | | | | | | | | | | | | | | | | | | | | | | | | | | | | | | | | | | | | | | | | | | | | | | | | | | | | | | | | | | | | | | | | | | | | | | | | | | | | | | | | | | |  |  |  |  |  |  |  |  |  |  |  |  |  |  |  |  |  |  |  |  |  |  |  |  |  |  |  |  |  |  |  |  |  |  |  |  |
|  | | | 1099.6641 | | | | | | | | | | | | | | | | | | 1099.665 | | | | | | | | | | | | | | | | | | | | | | | | | | | | | 0 | | | | | | | | | | | | | | | | | | | | | | | | | | | | | | | 1 | | | | | | | | | | | | | | | | | | | | | | | | | | | | | | | | | | 61 | | | | | | | | | | | | | | | | | | | | | | | | | | | | | | | | | | | | | | | 70 | | | | | | | | | | | | | | | | | | | | | | | | | | | | | | | | | | | | | | | | | | | | | AFEPILLLGK | | | | | | | | | | | | | | | | | | | | | | | | | | | | | | | | | | | | | | | | | | | | | | | | | | | | | | | | | | | | | | | | | | | | | | | | | | | | | | | | | | | | | | | | | | | | | | | | | | | | | | | | | | | | | | | | | | | | | | | | | | | | | | | | | | | | | | | | | | | | | | | | | | | | | | | | | | | | | | | | | | | | | | | | | | | | |  |  |  |  |  |  |  |  |  |  |  |  |  |  |  |  |  |  |  |  |  |  |  |  |  |  |  |  |  |  |  |  |  |  |  |  |
|  | | | 1342.7972 | | | | | | | | | | | | | | | | | | 1342.797 | | | | | | | | | | | | | | | | | | | | | | | | | | | | | 0 | | | | | | | | | | | | | | | | | | | | | | | | | | | | | | | 0 | | | | | | | | | | | | | | | | | | | | | | | | | | | | | | | | | | 61 | | | | | | | | | | | | | | | | | | | | | | | | | | | | | | | | | | | | | | | 72 | | | | | | | | | | | | | | | | | | | | | | | | | | | | | | | | | | | | | | | | | | | | | AFEPILLLGKSR | | | | | | | | | | | | | | | | | | | | | | | | | | | | | | | | | | | | | | | | | | | | | | | | | | | | | | | | | | | | | | | | | | | | | | | | | | | | | | | | | | | | | | | | | | | | | | | | | | | | | | | | | | | | | | | | | | | | | | | | | | | | | | | | | | | | | | | | | | | | | | | | | | | | | | | | | | | | | | | | | | | | | | | | | | | | |  |  |  |  |  |  |  |  |  |  |  |  |  |  |  |  |  |  |  |  |  |  |  |  |  |  |  |  |  |  |  |  |  |  |  |  |
|  | | | 1485.8052 | | | | | | | | | | | | | | | | | | 1485.805 | | | | | | | | | | | | | | | | | | | | | | | | | | | | | 0 | | | | | | | | | | | | | | | | | | | | | | | | | | | | | | | 0 | | | | | | | | | | | | | | | | | | | | | | | | | | | | | | | | | | 82 | | | | | | | | | | | | | | | | | | | | | | | | | | | | | | | | | | | | | | | 95 | | | | | | | | | | | | | | | | | | | | | | | | | | | | | | | | | | | | | | | | | | | | | VKGGGHTSQIYAIR | | | | | | | | | | | | | | | | | | | | | | | | | | | | | | | | | | | | | | | | | | | | | | | | | | | | | | | | | | | | | | | | | | | | | | | | | | | | | | | | | | | | | | | | | | | | | | | | | | | | | | | | | | | | | | | | | | | | | | | | | | | | | | | | | | | | | | | | | | | | | | | | | | | | | | | | | | | | | | | | | | | | | | | | | | | | |  |  |  |  |  |  |  |  |  |  |  |  |  |  |  |  |  |  |  |  |  |  |  |  |  |  |  |  |  |  |  |  |  |  |  |  |
|  | | | 1485.8052 | | | | | | | | | | | | | | | | | | 1485.807 | | | | | | | | | | | | | | | | | | | | | | | | | | | | | 0 | | | | | | | | | | | | | | | | | | | | | | | | | | | | | | | 1 | | | | | | | | | | | | | | | | | | | | | | | | | | | | | | | | | | 82 | | | | | | | | | | | | | | | | | | | | | | | | | | | | | | | | | | | | | | | 95 | | | | | | | | | | | | | | | | | | | | | | | | | | | | | | | | | | | | | | | | | | | | | VKGGGHTSQIYAIR | | | | | | | | | | | | | | | | | | | | | | | | | | | | | | | | | | | | | | | | | | | | | | | | | | | | | | | | | | | | | | | | | | | | | | | | | | | | | | | | | | | | | | | | | | | | | | | | | | | | | | | | | | | | | | | | | | | | | | | | | | | | | | | | | | | | | | | | | | | | | | | | | | | | | | | | | | | | | | | | | | | | | | | | | | | | |  |  |  |  |  |  |  |  |  |  |  |  |  |  |  |  |  |  |  |  |  |  |  |  |  |  |  |  |  |  |  |  |  |  |  |  |
|  | | | 938.5225 | | | | | | | | | | | | | | | | | | 938.5218 | | | | | | | | | | | | | | | | | | | | | | | | | | | | | 0 | | | | | | | | | | | | | | | | | | | | | | | | | | | | | | | -1 | | | | | | | | | | | | | | | | | | | | | | | | | | | | | | | | | | 101 | | | | | | | | | | | | | | | | | | | | | | | | | | | | | | | | | | | | | | | 108 | | | | | | | | | | | | | | | | | | | | | | | | | | | | | | | | | | | | | | | | | | | | | ALVAFYQK | | | | | | | | | | | | | | | | | | | | | | | | | | | | | | | | | | | | | | | | | | | | | | | | | | | | | | | | | | | | | | | | | | | | | | | | | | | | | | | | | | | | | | | | | | | | | | | | | | | | | | | | | | | | | | | | | | | | | | | | | | | | | | | | | | | | | | | | | | | | | | | | | | | | | | | | | | | | | | | | | | | | | | | | | | | | |  |  |  |  |  |  |  |  |  |  |  |  |  |  |  |  |  |  |  |  |  |  |  |  |  |  |  |  |  |  |  |  |  |  |  |  |
|  | | | 938.5225 | | | | | | | | | | | | | | | | | | 938.5221 | | | | | | | | | | | | | | | | | | | | | | | | | | | | | 0 | | | | | | | | | | | | | | | | | | | | | | | | | | | | | | | 0 | | | | | | | | | | | | | | | | | | | | | | | | | | | | | | | | | | 101 | | | | | | | | | | | | | | | | | | | | | | | | | | | | | | | | | | | | | | | 108 | | | | | | | | | | | | | | | | | | | | | | | | | | | | | | | | | | | | | | | | | | | | | ALVAFYQK | | | | | | | | | | | | | | | | | | | | | | | | | | | | | | | | | | | | | | | | | | | | | | | | | | | | | | | | | | | | | | | | | | | | | | | | | | | | | | | | | | | | | | | | | | | | | | | | | | | | | | | | | | | | | | | | | | | | | | | | | | | | | | | | | | | | | | | | | | | | | | | | | | | | | | | | | | | | | | | | | | | | | | | | | | | | |  |  |  |  |  |  |  |  |  |  |  |  |  |  |  |  |  |  |  |  |  |  |  |  |  |  |  |  |  |  |  |  |  |  |  |  |
|  | | | 938.5225 | | | | | | | | | | | | | | | | | | 938.5226 | | | | | | | | | | | | | | | | | | | | | | | | | | | | | 0 | | | | | | | | | | | | | | | | | | | | | | | | | | | | | | | 0 | | | | | | | | | | | | | | | | | | | | | | | | | | | | | | | | | | 101 | | | | | | | | | | | | | | | | | | | | | | | | | | | | | | | | | | | | | | | 108 | | | | | | | | | | | | | | | | | | | | | | | | | | | | | | | | | | | | | | | | | | | | | ALVAFYQK | | | | | | | | | | | | | | | | | | | | | | | | | | | | | | | | | | | | | | | | | | | | | | | | | | | | | | | | | | | | | | | | | | | | | | | | | | | | | | | | | | | | | | | | | | | | | | | | | | | | | | | | | | | | | | | | | | | | | | | | | | | | | | | | | | | | | | | | | | | | | | | | | | | | | | | | | | | | | | | | | | | | | | | | | | | | |  |  |  |  |  |  |  |  |  |  |  |  |  |  |  |  |  |  |  |  |  |  |  |  |  |  |  |  |  |  |  |  |  |  |  |  |
| Spot no. | | | Homologous protein | | | | | | | | | | | | | | | | | | Accession no. | | | | | | | | | | | | | | | | | | | | | | | | | | | | | Score | | | | | | | | | | | | | | | | | | | | | | | | | | | | | | | M.P. | | | | | | | | | | | | | | | | | | | | | | | | | | | | | | | | | | Cov. (%) | | | | | | | | | | | | | | | | | | | | | | | | | | | | | | | | | | | | | | | Blast score | | | | | | | | | | | | | | | | | | | | | | | | | | | | | | | | | | | | | | | | | | | | | Mr (kDa) / pI | | | | | | | | | | | | | | | | | | | | | | | | | | | | | | | | | | | | | | | | | | | | | | | | | | | | | | | | | | | | | | | | | | | | | | | | | | | | | | | | | | | | | | | | | | | | | | | | | | | | | | | Ratio | | | | | | | | | | | | | | | | | | | | | | | | | | | | | | | | | | | | | | | | | | | | | | | | | | | | | | | | | | | | | | | | | | | | | | | | | | | | | |  |  |  |  |  |  |  |  |  |  |  |  |  |  |  |  |  |  |  |  |  |  |  |  |  |  |  |  |
|  |  |  |  |  |  |  |  |  |  |  |  |  |  |  |  |  |  |  |  |  |  |  |  |  |  |  |  |  |  |  |  |  |  |  |  |  |  |  |  |  |  |  |  |  |  |  |  |  |  |  |  |  |  |  |  |  |  |  |  |  |  |  |  |  |  |  |  |  |  |  |  |  |  |  |  |  |  |  |  |  |  |  |  |  |  |  |  |  |  |  |  |  |  |  |  |  |  |  |  |  |  |  |  |  |  |  |  |  |  |  |  |  |  |  |  |  |  |  |  |  |  |  |  |  |  |  |  |  |  |  |  |  |  |  |  |  |  |  |  |  |  |  |  |  |  |  |  |  |  |  |  |  |  |  |  |  |  |  |  |  |  |  |  |  |  |  |  |  |  |  |  |  |  |  |  |  |  |  |  |  |  |  |  |  |  |  |  |  |  |  |  |  |  |  |  |  |  |  | Theo. | | | | | | | | | | | | | | | | | | | | | | | | | | | | | | | | | | | | | | | | | | | Exp. | | | | | | | | | | | | | | | | | | | | | | | | | | | | | | | | | | | | | | | | | | | | | | | | | | | | | | | | | | | |  |  |  |  |  |  |  |  |  |  |  |  |  |  |  |  |  |  |  |  |  |  |  |  |  |  |  |  |  |  |  |  |  |  |  |  |  |  |  |  |  |  |  |  |  |  |  |  |  |  |  |  |  |  |  |  |  |  |  |  |  |  |  |  |  |  |  |  |  |  |  |  |  |  |  |  |  |  |  |  |  |  |  |  |  |  |  |  |  |  |  |  |  |  |  |  |  |  |  |  |  |  |  |  |  |  |
| 1g | | | glyceraldehyde-3-phosphate dehydrogenase | | | | | | | | | | | | | | | | | | Glyma04g36860.1 | | | | | | | | | | | | | | | | | | | | | | | | | | | | | 120 | | | | | | | | | | | | | | | | | | | | | | | | | | | | | | | 2 | | | | | | | | | | | | | | | | | | | | | | | | | | | | | | | | | | 13 | | | | | | | | | | | | | | | | | | | | | | | | | | | | | | | | | | | | | | | 637 | | | | | | | | | | | | | | | | | | | | | | | | | | | | | | | | | | | | | | | | | | | | | 37.1/6.72 | | | | | | | | | | | | | | | | | | | | | | | | | | | | | | | | | | | | | | | | | | |  | | | | | | | | | | | | | | | | | | | | | | | | | | | | | | | | | | | | | | | | | | | | | | | | | | | | | | | | | | | |  | | | | | | | | | | | | | | | | | | | | | | | | | | | | | | | | | | | | | | | | | | | | | | | | | | | | | | | | | | | | | | | | | | | | | | | | | | | | | |  |  |  |  |  |  |  |  |  |  |  |  |  |  |  |  |  |  |  |  |  |  |  |  |  |  |  |  |
|  | | | Calc. Mass | | | | | | | | | | | | | | | | | | Observ. Mass | | | | | | | | | | | | | | | | | | | | | | | | | | | | | Da | | | | | | | | | | | | | | | | | | | | | | | | | | | | | | | ppm | | | | | | | | | | | | | | | | | | | | | | | | | | | | | | | | | | start | | | | | | | | | | | | | | | | | | | | | | | | | | | | | | | | | | | | | | | end | | | | | | | | | | | | | | | | | | | | | | | | | | | | | | | | | | | | | | | | | | | | | sequence | | | | | | | | | | | | | | | | | | | | | | | | | | | | | | | | | | | | | | | | | | | | | | | | | | | | | | | | | | | | | | | | | | | | | | | | | | | | | | | | | | | | | | | | | | | | | | | | | | | | | | | | | | | | | | | | | | | | | | | | | | | | | | | | | | | | | | | | | | | | | | | | | | | | | | | | | | | | | | | | | | | | | | | | | | | | | | | | | | | | |  |  |  |  |  |  |  |  |  |  |  |  |  |  |  |  |  |  |  |  |  |  |  |  |  |  |  |  |
|  | | | 2904.4029 | | | | | | | | | | | | | | | | | | 2904.403 | | | | | | | | | | | | | | | | | | | | | | | | | | | | | 0 | | | | | | | | | | | | | | | | | | | | | | | | | | | | | | | 0 | | | | | | | | | | | | | | | | | | | | | | | | | | | | | | | | | | 83 | | | | | | | | | | | | | | | | | | | | | | | | | | | | | | | | | | | | | | | 109 | | | | | | | | | | | | | | | | | | | | | | | | | | | | | | | | | | | | | | | | | | | | | NPEEIPWGSTGADIIVESTGVFTDKDK | | | | | | | | | | | | | | | | | | | | | | | | | | | | | | | | | | | | | | | | | | | | | | | | | | | | | | | | | | | | | | | | | | | | | | | | | | | | | | | | | | | | | | | | | | | | | | | | | | | | | | | | | | | | | | | | | | | | | | | | | | | | | | | | | | | | | | | | | | | | | | | | | | | | | | | | | | | | | | | | | | | | | | | | | | | | | | | | | | | | |  |  |  |  |  |  |  |  |  |  |  |  |  |  |  |  |  |  |  |  |  |  |  |  |  |  |  |  |
|  | | | 2143.991 | | | | | | | | | | | | | | | | | | 2143.985 | | | | | | | | | | | | | | | | | | | | | | | | | | | | | 0 | | | | | | | | | | | | | | | | | | | | | | | | | | | | | | | -3 | | | | | | | | | | | | | | | | | | | | | | | | | | | | | | | | | | 274 | | | | | | | | | | | | | | | | | | | | | | | | | | | | | | | | | | | | | | | 293 | | | | | | | | | | | | | | | | | | | | | | | | | | | | | | | | | | | | | | | | | | | | | GILGYTEDDVVSTDFVGDSR | | | | | | | | | | | | | | | | | | | | | | | | | | | | | | | | | | | | | | | | | | | | | | | | | | | | | | | | | | | | | | | | | | | | | | | | | | | | | | | | | | | | | | | | | | | | | | | | | | | | | | | | | | | | | | | | | | | | | | | | | | | | | | | | | | | | | | | | | | | | | | | | | | | | | | | | | | | | | | | | | | | | | | | | | | | | | | | | | | | | |  |  |  |  |  |  |  |  |  |  |  |  |  |  |  |  |  |  |  |  |  |  |  |  |  |  |  |  |
| Spot no. | | | Homologous protein | | | | | | | | | | | | | | | | | | Accession no. | | | | | | | | | | | | | | | | | | | | | | | | | | | | | Score | | | | | | | | | | | | | | | | | | | | | | | | | | | | | | | | | | | M.P. | | | | | | | | | | | | | | | | | | | | | | | | | | | | | | | | | | | Cov. (%) | | | | | | | | | | | | | | | | | | | | | | | | | | | | | | | | | | | | | Blast score | | | | | | | | | | | | | | | | | | | | | | | | | | | | | | | | | | | | | | | | | | | | | Mr (kDa) / pI | | | | | | | | | | | | | | | | | | | | | | | | | | | | | | | | | | | | | | | | | | | | | | | | | | | | | | | | | | | | | | | | | | | | | | | | | | | | | | | | | | | | | | | | | | | | | | | | | | | | | | | | | | | | | | | | | | | | | | | | | | | | | | | | | | Ratio | | | | | | | | | | | | | | | | | | | | | | | | | | | | | | | | | | | | | | | |  |  |  |  |  |  |  |  |  |  |  |  |  |  |  |  |  |  |  |  |  |  |  |  |  |  |  |  |  |  |  |  |  |  |  |  |
|  |  |  |  |  |  |  |  |  |  |  |  |  |  |  |  |  |  |  |  |  |  |  |  |  |  |  |  |  |  |  |  |  |  |  |  |  |  |  |  |  |  |  |  |  |  |  |  |  |  |  |  |  |  |  |  |  |  |  |  |  |  |  |  |  |  |  |  |  |  |  |  |  |  |  |  |  |  |  |  |  |  |  |  |  |  |  |  |  |  |  |  |  |  |  |  |  |  |  |  |  |  |  |  |  |  |  |  |  |  |  |  |  |  |  |  |  |  |  |  |  |  |  |  |  |  |  |  |  |  |  |  |  |  |  |  |  |  |  |  |  |  |  |  |  |  |  |  |  |  |  |  |  |  |  |  |  |  |  |  |  |  |  |  |  |  |  |  |  |  |  |  |  |  |  |  |  |  |  |  |  |  |  |  |  |  |  |  |  |  |  |  |  |  |  |  |  |  |  |  |  |  | Theo. | | | | | | | | | | | | | | | | | | | | | | | | | | | | | | | | | | | | | | | | Exp. | | | | | | | | | | | | | | | | | | | | | | | | | | | | | | | | | | | | | | | | | | | | | | | | | | | | | | | | | | | | | | | | | | | | | | | | | | | | | | | | | | | | | | | | | |  |  |  |  |  |  |  |  |  |  |  |  |  |  |  |  |  |  |  |  |  |  |  |  |  |  |  |  |  |  |  |  |  |  |  |  |  |  |  |  |  |  |  |  |  |  |  |  |  |  |  |  |  |  |  |  |  |  |  |  |  |  |  |  |  |  |  |  |  |  |  |  |  |  |  |  |
| 1h | | | stem 31 kDa glycoprotein | | | | | | | | | | | | | | | | | | Glyma08g21410.1 | | | | | | | | | | | | | | | | | | | | | | | | | | | | | 100 | | | | | | | | | | | | | | | | | | | | | | | | | | | | | | | | | | | 1 | | | | | | | | | | | | | | | | | | | | | | | | | | | | | | | | | | | 5 | | | | | | | | | | | | | | | | | | | | | | | | | | | | | | | | | | | | | 500 | | | | | | | | | | | | | | | | | | | | | | | | | | | | | | | | | | | | | | | | | | | | | 29.7/6.71 | | | | | | | | | | | | | | | | | | | | | | | | | | | | | | | | | | | | | | | |  | | | | | | | | | | | | | | | | | | | | | | | | | | | | | | | | | | | | | | | | | | | | | | | | | | | | | | | | | | | | | | | | | | | | | | | | | | | | | | | | | | | | | | | | | |  | | | | | | | | | | | | | | | | | | | | | | | | | | | | | | | | | | | | | | | |  |  |  |  |  |  |  |  |  |  |  |  |  |  |  |  |  |  |  |  |  |  |  |  |  |  |  |  |  |  |  |  |  |  |  |  |
|  | | | Calc. Mass | | | | | | | | | | | | | | | | | | Observ. Mass | | | | | | | | | | | | | | | | | | | | | | | | | | | | | Da | | | | | | | | | | | | | | | | | | | | | | | | | | | | | | | | | | | ppm | | | | | | | | | | | | | | | | | | | | | | | | | | | | | | | | | | | start | | | | | | | | | | | | | | | | | | | | | | | | | | | | | | | | | | | | | end | | | | | | | | | | | | | | | | | | | | | | | | | | | | | | | | | | | | | | | | | | | | | sequence | | | | | | | | | | | | | | | | | | | | | | | | | | | | | | | | | | | | | | | | | | | | | | | | | | | | | | | | | | | | | | | | | | | | | | | | | | | | | | | | | | | | | | | | | | | | | | | | | | | | | | | | | | | | | | | | | | | | | | | | | | | | | | | | | | | | | | | | | | | | | | | | | | | | | | | | | | | | | | | | | | | | | | | | | |  |  |  |  |  |  |  |  |  |  |  |  |  |  |  |  |  |  |  |  |  |  |  |  |  |  |  |  |  |  |  |  |  |  |  |  |
|  | | | 1559.7368 | | | | | | | | | | | | | | | | | | 1559.736 | | | | | | | | | | | | | | | | | | | | | | | | | | | | | 0 | | | | | | | | | | | | | | | | | | | | | | | | | | | | | | | | | | | -1 | | | | | | | | | | | | | | | | | | | | | | | | | | | | | | | | | | | 84 | | | | | | | | | | | | | | | | | | | | | | | | | | | | | | | | | | | | | 96 | | | | | | | | | | | | | | | | | | | | | | | | | | | | | | | | | | | | | | | | | | | | | TVNQQAFFYASER | | | | | | | | | | | | | | | | | | | | | | | | | | | | | | | | | | | | | | | | | | | | | | | | | | | | | | | | | | | | | | | | | | | | | | | | | | | | | | | | | | | | | | | | | | | | | | | | | | | | | | | | | | | | | | | | | | | | | | | | | | | | | | | | | | | | | | | | | | | | | | | | | | | | | | | | | | | | | | | | | | | | | | | | | |  |  |  |  |  |  |  |  |  |  |  |  |  |  |  |  |  |  |  |  |  |  |  |  |  |  |  |  |  |  |  |  |  |  |  |  |
|  | | | 1559.7368 | | | | | | | | | | | | | | | | | | 1559.736 | | | | | | | | | | | | | | | | | | | | | | | | | | | | | 0 | | | | | | | | | | | | | | | | | | | | | | | | | | | | | | | | | | | -1 | | | | | | | | | | | | | | | | | | | | | | | | | | | | | | | | | | | 84 | | | | | | | | | | | | | | | | | | | | | | | | | | | | | | | | | | | | | 96 | | | | | | | | | | | | | | | | | | | | | | | | | | | | | | | | | | | | | | | | | | | | | TVNQQAFFYASER | | | | | | | | | | | | | | | | | | | | | | | | | | | | | | | | | | | | | | | | | | | | | | | | | | | | | | | | | | | | | | | | | | | | | | | | | | | | | | | | | | | | | | | | | | | | | | | | | | | | | | | | | | | | | | | | | | | | | | | | | | | | | | | | | | | | | | | | | | | | | | | | | | | | | | | | | | | | | | | | | | | | | | | | | |  |  |  |  |  |  |  |  |  |  |  |  |  |  |  |  |  |  |  |  |  |  |  |  |  |  |  |  |  |  |  |  |  |  |  |  |
| Spot no. | | | Homologous protein | | | | | | | | | | | | | | | | | | Accession no. | | | | | | | | | | | | | | | | | | | | | | | | | | | | | Score | | | | | | | | | | | | | | | | | | | | | | | | | | | | | | | | | | | M.P. | | | | | | | | | | | | | | | | | | | | | | | | | | | | | | | | | | | Cov. (%) | | | | | | | | | | | | | | | | | | | | | | | | | | | | | | | | | | | | | Blast score | | | | | | | | | | | | | | | | | | | | | | | | | | | | | | | | | | | | | | | | | | | | | Mr (kDa) / pI | | | | | | | | | | | | | | | | | | | | | | | | | | | | | | | | | | | | | | | | | | | | | | | | | | | | | | | | | | | | | | | | | | | | | | | | | | | | | | | | | | | | | | | | | | | | | | | | | | | | | | | | | | | | | | | | | | | | | | | | | | | | | | | | | | | | Ratio | | | | | | | | | | | | | | | | | | | | | | | | | | | | | | | | | | | | | |  |  |  |  |  |  |  |  |  |  |  |  |  |  |  |  |  |  |  |  |  |  |  |  |  |  |  |  |  |  |  |  |  |  |  |  |
|  |  |  |  |  |  |  |  |  |  |  |  |  |  |  |  |  |  |  |  |  |  |  |  |  |  |  |  |  |  |  |  |  |  |  |  |  |  |  |  |  |  |  |  |  |  |  |  |  |  |  |  |  |  |  |  |  |  |  |  |  |  |  |  |  |  |  |  |  |  |  |  |  |  |  |  |  |  |  |  |  |  |  |  |  |  |  |  |  |  |  |  |  |  |  |  |  |  |  |  |  |  |  |  |  |  |  |  |  |  |  |  |  |  |  |  |  |  |  |  |  |  |  |  |  |  |  |  |  |  |  |  |  |  |  |  |  |  |  |  |  |  |  |  |  |  |  |  |  |  |  |  |  |  |  |  |  |  |  |  |  |  |  |  |  |  |  |  |  |  |  |  |  |  |  |  |  |  |  |  |  |  |  |  |  |  |  |  |  |  |  |  |  |  |  |  |  |  |  |  |  |  | Theo. | | | | | | | | | | | | | | | | | | | | | | | | | | | | | | | | | | | | | | | | | | | | | | Exp. | | | | | | | | | | | | | | | | | | | | | | | | | | | | | | | | | | | | | | | | | | | | | | | | | | | | | | | | | | | | | | | | | | | | | | | | | | | | | | | | | | | | | |  |  |  |  |  |  |  |  |  |  |  |  |  |  |  |  |  |  |  |  |  |  |  |  |  |  |  |  |  |  |  |  |  |  |  |  |  |  |  |  |  |  |  |  |  |  |  |  |  |  |  |  |  |  |  |  |  |  |  |  |  |  |  |  |  |  |  |  |  |  |  |  |  |  |
| 2a | | | uncharacterized protein LOC100527731 | | | | | | | | | | | | | | | | | | Glyma07g37270.1 | | | | | | | | | | | | | | | | | | | | | | | | | | | | | 212 | | | | | | | | | | | | | | | | | | | | | | | | | | | | | | | | | | | 6 | | | | | | | | | | | | | | | | | | | | | | | | | | | | | | | | | | | 40 | | | | | | | | | | | | | | | | | | | | | | | | | | | | | | | | | | | | | 317 | | | | | | | | | | | | | | | | | | | | | | | | | | | | | | | | | | | | | | | | | | | | | 17.0/4.72 | | | | | | | | | | | | | | | | | | | | | | | | | | | | | | | | | | | | | | | | | | | | | |  | | | | | | | | | | | | | | | | | | | | | | | | | | | | | | | | | | | | | | | | | | | | | | | | | | | | | | | | | | | | | | | | | | | | | | | | | | | | | | | | | | | | | |  | | | | | | | | | | | | | | | | | | | | | | | | | | | | | | | | | | | | | |  |  |  |  |  |  |  |  |  |  |  |  |  |  |  |  |  |  |  |  |  |  |  |  |  |  |  |  |  |  |  |  |  |  |  |  |
|  | | | Calc. Mass | | | | | | | | | | | | | | | | | | Observ. Mass | | | | | | | | | | | | | | | | | | | | | | | | | | | | | Da | | | | | | | | | | | | | | | | | | | | | | | | | | | | | | | | | | | ppm | | | | | | | | | | | | | | | | | | | | | | | | | | | | | | | | | | | start | | | | | | | | | | | | | | | | | | | | | | | | | | | | | | | | | | | | | end | | | | | | | | | | | | | | | | | | | | | | | | | | | | | | | | | | | | | | | | | | | | | sequence | | | | | | | | | | | | | | | | | | | | | | | | | | | | | | | | | | | | | | | | | | | | | | | | | | | | | | | | | | | | | | | | | | | | | | | | | | | | | | | | | | | | | | | | | | | | | | | | | | | | | | | | | | | | | | | | | | | | | | | | | | | | | | | | | | | | | | | | | | | | | | | | | | | | | | | | | | | | | | | | | | | | | | | | | |  |  |  |  |  |  |  |  |  |  |  |  |  |  |  |  |  |  |  |  |  |  |  |  |  |  |  |  |  |  |  |  |  |  |  |  |
|  | | | 2172.0627 | | | | | | | | | | | | | | | | | | 2172.063 | | | | | | | | | | | | | | | | | | | | | | | | | | | | | 0 | | | | | | | | | | | | | | | | | | | | | | | | | | | | | | | | | | | 0 | | | | | | | | | | | | | | | | | | | | | | | | | | | | | | | | | | | 2 | | | | | | | | | | | | | | | | | | | | | | | | | | | | | | | | | | | | | 21 | | | | | | | | | | | | | | | | | | | | | | | | | | | | | | | | | | | | | | | | | | | | | GVFTFEDETTSPVAPATLYK | | | | | | | | | | | | | | | | | | | | | | | | | | | | | | | | | | | | | | | | | | | | | | | | | | | | | | | | | | | | | | | | | | | | | | | | | | | | | | | | | | | | | | | | | | | | | | | | | | | | | | | | | | | | | | | | | | | | | | | | | | | | | | | | | | | | | | | | | | | | | | | | | | | | | | | | | | | | | | | | | | | | | | | | | |  |  |  |  |  |  |  |  |  |  |  |  |  |  |  |  |  |  |  |  |  |  |  |  |  |  |  |  |  |  |  |  |  |  |  |  |
|  | | | 1254.682 | | | | | | | | | | | | | | | | | | 1254.682 | | | | | | | | | | | | | | | | | | | | | | | | | | | | | 0 | | | | | | | | | | | | | | | | | | | | | | | | | | | | | | | | | | | 0 | | | | | | | | | | | | | | | | | | | | | | | | | | | | | | | | | | | 22 | | | | | | | | | | | | | | | | | | | | | | | | | | | | | | | | | | | | | 33 | | | | | | | | | | | | | | | | | | | | | | | | | | | | | | | | | | | | | | | | | | | | | ALVTDADNVIPK | | | | | | | | | | | | | | | | | | | | | | | | | | | | | | | | | | | | | | | | | | | | | | | | | | | | | | | | | | | | | | | | | | | | | | | | | | | | | | | | | | | | | | | | | | | | | | | | | | | | | | | | | | | | | | | | | | | | | | | | | | | | | | | | | | | | | | | | | | | | | | | | | | | | | | | | | | | | | | | | | | | | | | | | | |  |  |  |  |  |  |  |  |  |  |  |  |  |  |  |  |  |  |  |  |  |  |  |  |  |  |  |  |  |  |  |  |  |  |  |  |
|  | | | 1254.682 | | | | | | | | | | | | | | | | | | 1254.682 | | | | | | | | | | | | | | | | | | | | | | | | | | | | | 0 | | | | | | | | | | | | | | | | | | | | | | | | | | | | | | | | | | | 0 | | | | | | | | | | | | | | | | | | | | | | | | | | | | | | | | | | | 22 | | | | | | | | | | | | | | | | | | | | | | | | | | | | | | | | | | | | | 33 | | | | | | | | | | | | | | | | | | | | | | | | | | | | | | | | | | | | | | | | | | | | | ALVTDADNVIPK | | | | | | | | | | | | | | | | | | | | | | | | | | | | | | | | | | | | | | | | | | | | | | | | | | | | | | | | | | | | | | | | | | | | | | | | | | | | | | | | | | | | | | | | | | | | | | | | | | | | | | | | | | | | | | | | | | | | | | | | | | | | | | | | | | | | | | | | | | | | | | | | | | | | | | | | | | | | | | | | | | | | | | | | | |  |  |  |  |  |  |  |  |  |  |  |  |  |  |  |  |  |  |  |  |  |  |  |  |  |  |  |  |  |  |  |  |  |  |  |  |
|  | | | 1254.682 | | | | | | | | | | | | | | | | | | 1254.682 | | | | | | | | | | | | | | | | | | | | | | | | | | | | | 0 | | | | | | | | | | | | | | | | | | | | | | | | | | | | | | | | | | | 0 | | | | | | | | | | | | | | | | | | | | | | | | | | | | | | | | | | | 22 | | | | | | | | | | | | | | | | | | | | | | | | | | | | | | | | | | | | | 33 | | | | | | | | | | | | | | | | | | | | | | | | | | | | | | | | | | | | | | | | | | | | | ALVTDADNVIPK | | | | | | | | | | | | | | | | | | | | | | | | | | | | | | | | | | | | | | | | | | | | | | | | | | | | | | | | | | | | | | | | | | | | | | | | | | | | | | | | | | | | | | | | | | | | | | | | | | | | | | | | | | | | | | | | | | | | | | | | | | | | | | | | | | | | | | | | | | | | | | | | | | | | | | | | | | | | | | | | | | | | | | | | | |  |  |  |  |  |  |  |  |  |  |  |  |  |  |  |  |  |  |  |  |  |  |  |  |  |  |  |  |  |  |  |  |  |  |  |  |
|  | | | 1914.0211 | | | | | | | | | | | | | | | | | | 1914.021 | | | | | | | | | | | | | | | | | | | | | | | | | | | | | 0 | | | | | | | | | | | | | | | | | | | | | | | | | | | | | | | | | | | 0 | | | | | | | | | | | | | | | | | | | | | | | | | | | | | | | | | | | 22 | | | | | | | | | | | | | | | | | | | | | | | | | | | | | | | | | | | | | 39 | | | | | | | | | | | | | | | | | | | | | | | | | | | | | | | | | | | | | | | | | | | | | ALVTDADNVIPKAVDAFR | | | | | | | | | | | | | | | | | | | | | | | | | | | | | | | | | | | | | | | | | | | | | | | | | | | | | | | | | | | | | | | | | | | | | | | | | | | | | | | | | | | | | | | | | | | | | | | | | | | | | | | | | | | | | | | | | | | | | | | | | | | | | | | | | | | | | | | | | | | | | | | | | | | | | | | | | | | | | | | | | | | | | | | | | |  |  |  |  |  |  |  |  |  |  |  |  |  |  |  |  |  |  |  |  |  |  |  |  |  |  |  |  |  |  |  |  |  |  |  |  |
|  | | | 1279.666 | | | | | | | | | | | | | | | | | | 1279.666 | | | | | | | | | | | | | | | | | | | | | | | | | | | | | 0 | | | | | | | | | | | | | | | | | | | | | | | | | | | | | | | | | | | 0 | | | | | | | | | | | | | | | | | | | | | | | | | | | | | | | | | | | 55 | | | | | | | | | | | | | | | | | | | | | | | | | | | | | | | | | | | | | 65 | | | | | | | | | | | | | | | | | | | | | | | | | | | | | | | | | | | | | | | | | | | | | KITFLEDGETK | | | | | | | | | | | | | | | | | | | | | | | | | | | | | | | | | | | | | | | | | | | | | | | | | | | | | | | | | | | | | | | | | | | | | | | | | | | | | | | | | | | | | | | | | | | | | | | | | | | | | | | | | | | | | | | | | | | | | | | | | | | | | | | | | | | | | | | | | | | | | | | | | | | | | | | | | | | | | | | | | | | | | | | | | |  |  |  |  |  |  |  |  |  |  |  |  |  |  |  |  |  |  |  |  |  |  |  |  |  |  |  |  |  |  |  |  |  |  |  |  |
|  | | | 1279.666 | | | | | | | | | | | | | | | | | | 1279.668 | | | | | | | | | | | | | | | | | | | | | | | | | | | | | 0 | | | | | | | | | | | | | | | | | | | | | | | | | | | | | | | | | | | 1 | | | | | | | | | | | | | | | | | | | | | | | | | | | | | | | | | | | 55 | | | | | | | | | | | | | | | | | | | | | | | | | | | | | | | | | | | | | 65 | | | | | | | | | | | | | | | | | | | | | | | | | | | | | | | | | | | | | | | | | | | | | KITFLEDGETK | | | | | | | | | | | | | | | | | | | | | | | | | | | | | | | | | | | | | | | | | | | | | | | | | | | | | | | | | | | | | | | | | | | | | | | | | | | | | | | | | | | | | | | | | | | | | | | | | | | | | | | | | | | | | | | | | | | | | | | | | | | | | | | | | | | | | | | | | | | | | | | | | | | | | | | | | | | | | | | | | | | | | | | | | |  |  |  |  |  |  |  |  |  |  |  |  |  |  |  |  |  |  |  |  |  |  |  |  |  |  |  |  |  |  |  |  |  |  |  |  |
|  | | | 1151.571 | | | | | | | | | | | | | | | | | | 1151.57 | | | | | | | | | | | | | | | | | | | | | | | | | | | | | 0 | | | | | | | | | | | | | | | | | | | | | | | | | | | | | | | | | | | -1 | | | | | | | | | | | | | | | | | | | | | | | | | | | | | | | | | | | 56 | | | | | | | | | | | | | | | | | | | | | | | | | | | | | | | | | | | | | 65 | | | | | | | | | | | | | | | | | | | | | | | | | | | | | | | | | | | | | | | | | | | | | ITFLEDGETK | | | | | | | | | | | | | | | | | | | | | | | | | | | | | | | | | | | | | | | | | | | | | | | | | | | | | | | | | | | | | | | | | | | | | | | | | | | | | | | | | | | | | | | | | | | | | | | | | | | | | | | | | | | | | | | | | | | | | | | | | | | | | | | | | | | | | | | | | | | | | | | | | | | | | | | | | | | | | | | | | | | | | | | | | |  |  |  |  |  |  |  |  |  |  |  |  |  |  |  |  |  |  |  |  |  |  |  |  |  |  |  |  |  |  |  |  |  |  |  |  |
|  | | | 1413.794 | | | | | | | | | | | | | | | | | | 1413.794 | | | | | | | | | | | | | | | | | | | | | | | | | | | | | 0 | | | | | | | | | | | | | | | | | | | | | | | | | | | | | | | | | | | 0 | | | | | | | | | | | | | | | | | | | | | | | | | | | | | | | | | | | 104 | | | | | | | | | | | | | | | | | | | | | | | | | | | | | | | | | | | | | 119 | | | | | | | | | | | | | | | | | | | | | | | | | | | | | | | | | | | | | | | | | | | | | LAAGANGGSAGKLTVK | | | | | | | | | | | | | | | | | | | | | | | | | | | | | | | | | | | | | | | | | | | | | | | | | | | | | | | | | | | | | | | | | | | | | | | | | | | | | | | | | | | | | | | | | | | | | | | | | | | | | | | | | | | | | | | | | | | | | | | | | | | | | | | | | | | | | | | | | | | | | | | | | | | | | | | | | | | | | | | | | | | | | | | | | |  |  |  |  |  |  |  |  |  |  |  |  |  |  |  |  |  |  |  |  |  |  |  |  |  |  |  |  |  |  |  |  |  |  |  |  |
| Spot no. | | | | | | | Homologous protein | | | | | | | | | | | | | | | | | | Accession no. | | | | | | | | | | | | | | | | | | | | | | | | | | | | | | | | Score | | | | | | | | | | | | | | | | | | | | | | | | | | | | | | M.P. | | | | | | | | | | | | | | | | | | | | | | | | | | | | | | | | | | | Cov. (%) | | | | | | | | | | | | | | | | | | | | | | | | | | | | | | | | | | | | Blast score | | | | | | | | | | | | | | | | | | | | | | | | | | | | | | | | | | | | | | | | | | | | Mr (kDa) / pI | | | | | | | | | | | | | | | | | | | | | | | | | | | | | | | | | | | | | | | | | | | | | | | | | | | | | | | | | | | | | | | | | | | | | | | | | | | | | | | | | | | | | | | | | | | | | | | | | | | | | | | | | | | | | | | | | | | | Ratio | | | | | | | | | | | | | | | | | | | | | | | | | | | | | | | | | | | | | | | | | | | | | | | | | | | | | | | | | | | | | |  |  |  |  |  |  |  |  |  |  |  |  |  |  |  |  |  |  |  |  |  |  |  |  |  |  |  |  |
|  |  |  |  |  |  |  |  |  |  |  |  |  |  |  |  |  |  |  |  |  |  |  |  |  |  |  |  |  |  |  |  |  |  |  |  |  |  |  |  |  |  |  |  |  |  |  |  |  |  |  |  |  |  |  |  |  |  |  |  |  |  |  |  |  |  |  |  |  |  |  |  |  |  |  |  |  |  |  |  |  |  |  |  |  |  |  |  |  |  |  |  |  |  |  |  |  |  |  |  |  |  |  |  |  |  |  |  |  |  |  |  |  |  |  |  |  |  |  |  |  |  |  |  |  |  |  |  |  |  |  |  |  |  |  |  |  |  |  |  |  |  |  |  |  |  |  |  |  |  |  |  |  |  |  |  |  |  |  |  |  |  |  |  |  |  |  |  |  |  |  |  |  |  |  |  |  |  |  |  |  |  |  |  |  |  |  |  |  |  |  |  |  |  |  |  |  |  |  |  |  |  | Theo. | | | | | | | | | | | | | | | | | | | | | | | | | | | | | | | | | | | | | | | | | | Exp. | | | | | | | | | | | | | | | | | | | | | | | | | | | | | | | | | | | | | | | | | | | | | | | | | | | | | | | | | | | | | | | | | | | | | | | | | |  |  |  |  |  |  |  |  |  |  |  |  |  |  |  |  |  |  |  |  |  |  |  |  |  |  |  |  |  |  |  |  |  |  |  |  |  |  |  |  |  |  |  |  |  |  |  |  |  |  |  |  |  |  |  |  |  |  |  |  |  |  |  |  |  |  |  |  |  |  |  |  |  |  |  |  |  |  |  |  |  |  |  |  |  |  |  |  |  |  |
| 2b | | | | | | | Phosphoglycerate kinase | | | | | | | | | | | | | | | | | | Glyma08g17600.1 | | | | | | | | | | | | | | | | | | | | | | | | | | | | | | | | 156 | | | | | | | | | | | | | | | | | | | | | | | | | | | | | | 3 | | | | | | | | | | | | | | | | | | | | | | | | | | | | | | | | | | | 14 | | | | | | | | | | | | | | | | | | | | | | | | | | | | | | | | | | | | 735 | | | | | | | | | | | | | | | | | | | | | | | | | | | | | | | | | | | | | | | | | | | | 42.6/6.28 | | | | | | | | | | | | | | | | | | | | | | | | | | | | | | | | | | | | | | | | | |  | | | | | | | | | | | | | | | | | | | | | | | | | | | | | | | | | | | | | | | | | | | | | | | | | | | | | | | | | | | | | | | | | | | | | | | | | |  | | | | | | | | | | | | | | | | | | | | | | | | | | | | | | | | | | | | | | | | | | | | | | | | | | | | | | | | | | | | | |  |  |  |  |  |  |  |  |  |  |  |  |  |  |  |  |  |  |  |  |  |  |  |  |  |  |  |  |
|  | | | | | | | Calc. Mass | | | | | | | | | | | | | | | | | | Observ. Mass | | | | | | | | | | | | | | | | | | | | | | | | | | | | | | | | Da | | | | | | | | | | | | | | | | | | | | | | | | | | | | | | ppm | | | | | | | | | | | | | | | | | | | | | | | | | | | | | | | | | | | start | | | | | | | | | | | | | | | | | | | | | | | | | | | | | | | | | | | | end | | | | | | | | | | | | | | | | | | | | | | | | | | | | | | | | | | | | | | | | | | | | sequence | | | | | | | | | | | | | | | | | | | | | | | | | | | | | | | | | | | | | | | | | | | | | | | | | | | | | | | | | | | | | | | | | | | | | | | | | | | | | | | | | | | | | | | | | | | | | | | | | | | | | | | | | | | | | | | | | | | | | | | | | | | | | | | | | | | | | | | | | | | | | | | | | | | | | | | | | | | | | | | | | | | | | | | | | | | | | | | | | |  |  |  |  |  |  |  |  |  |  |  |  |  |  |  |  |  |  |  |  |  |  |  |  |  |  |  |  |
|  | | | | | | | 2040.9964 | | | | | | | | | | | | | | | | | | 2040.989 | | | | | | | | | | | | | | | | | | | | | | | | | | | | | | | | 0 | | | | | | | | | | | | | | | | | | | | | | | | | | | | | | -3 | | | | | | | | | | | | | | | | | | | | | | | | | | | | | | | | | | | 24 | | | | | | | | | | | | | | | | | | | | | | | | | | | | | | | | | | | | 41 | | | | | | | | | | | | | | | | | | | | | | | | | | | | | | | | | | | | | | | | | | | | VDLNVPLDDNLNITDDTR | | | | | | | | | | | | | | | | | | | | | | | | | | | | | | | | | | | | | | | | | | | | | | | | | | | | | | | | | | | | | | | | | | | | | | | | | | | | | | | | | | | | | | | | | | | | | | | | | | | | | | | | | | | | | | | | | | | | | | | | | | | | | | | | | | | | | | | | | | | | | | | | | | | | | | | | | | | | | | | | | | | | | | | | | | | | | | | | | |  |  |  |  |  |  |  |  |  |  |  |  |  |  |  |  |  |  |  |  |  |  |  |  |  |  |  |  |
|  | | | | | | | 2637.3749 | | | | | | | | | | | | | | | | | | 2637.38 | | | | | | | | | | | | | | | | | | | | | | | | | | | | | | | | 0 | | | | | | | | | | | | | | | | | | | | | | | | | | | | | | 2 | | | | | | | | | | | | | | | | | | | | | | | | | | | | | | | | | | | 233 | | | | | | | | | | | | | | | | | | | | | | | | | | | | | | | | | | | | 257 | | | | | | | | | | | | | | | | | | | | | | | | | | | | | | | | | | | | | | | | | | | | AQGYSVGSSLVEEDKLSLATTLLEK | | | | | | | | | | | | | | | | | | | | | | | | | | | | | | | | | | | | | | | | | | | | | | | | | | | | | | | | | | | | | | | | | | | | | | | | | | | | | | | | | | | | | | | | | | | | | | | | | | | | | | | | | | | | | | | | | | | | | | | | | | | | | | | | | | | | | | | | | | | | | | | | | | | | | | | | | | | | | | | | | | | | | | | | | | | | | | | | | |  |  |  |  |  |  |  |  |  |  |  |  |  |  |  |  |  |  |  |  |  |  |  |  |  |  |  |  |
|  | | | | | | | 1572.8359 | | | | | | | | | | | | | | | | | | 1572.833 | | | | | | | | | | | | | | | | | | | | | | | | | | | | | | | | 0 | | | | | | | | | | | | | | | | | | | | | | | | | | | | | | -2 | | | | | | | | | | | | | | | | | | | | | | | | | | | | | | | | | | | 350 | | | | | | | | | | | | | | | | | | | | | | | | | | | | | | | | | | | | 366 | | | | | | | | | | | | | | | | | | | | | | | | | | | | | | | | | | | | | | | | | | | | GVTTIIGGGDSVAAVEK | | | | | | | | | | | | | | | | | | | | | | | | | | | | | | | | | | | | | | | | | | | | | | | | | | | | | | | | | | | | | | | | | | | | | | | | | | | | | | | | | | | | | | | | | | | | | | | | | | | | | | | | | | | | | | | | | | | | | | | | | | | | | | | | | | | | | | | | | | | | | | | | | | | | | | | | | | | | | | | | | | | | | | | | | | | | | | | | | |  |  |  |  |  |  |  |  |  |  |  |  |  |  |  |  |  |  |  |  |  |  |  |  |  |  |  |  |
| Spot no. | | | | | | | Homologous protein | | | | | | | | | | | | | | | | | | Accession no. | | | | | | | | | | | | | | | | | | | | | | | | | | | | | | | | Score | | | | | | | | | | | | | | | | | | | | | | | | | | | | | | M.P. | | | | | | | | | | | | | | | | | | | | | | | | | | | | | | | | | | | Cov. (%) | | | | | | | | | | | | | | | | | | | | | | | | | | | | | | | | | | | | Blast score | | | | | | | | | | | | | | | | | | | | | | | | | | | | | | | | | | | | | | | | | | | | Mr (kDa) / pI | | | | | | | | | | | | | | | | | | | | | | | | | | | | | | | | | | | | | | | | | | | | | | | | | | | | | | | | | | | | | | | | | | | | | | | | | | | | | | | | | | | | | | | | | | | | | | | | | | | | | | | | | | | | | | | | | | | | | | | | | | | | | | | | | | | | Ratio | | | | | | | | | | | | | | | | | | | | | | | | | | | | | | | | | | | | | | | | | | | | | |  |  |  |  |  |  |  |  |  |  |  |  |  |  |  |  |  |  |  |  |  |  |  |  |  |  |  |  |
|  |  |  |  |  |  |  |  |  |  |  |  |  |  |  |  |  |  |  |  |  |  |  |  |  |  |  |  |  |  |  |  |  |  |  |  |  |  |  |  |  |  |  |  |  |  |  |  |  |  |  |  |  |  |  |  |  |  |  |  |  |  |  |  |  |  |  |  |  |  |  |  |  |  |  |  |  |  |  |  |  |  |  |  |  |  |  |  |  |  |  |  |  |  |  |  |  |  |  |  |  |  |  |  |  |  |  |  |  |  |  |  |  |  |  |  |  |  |  |  |  |  |  |  |  |  |  |  |  |  |  |  |  |  |  |  |  |  |  |  |  |  |  |  |  |  |  |  |  |  |  |  |  |  |  |  |  |  |  |  |  |  |  |  |  |  |  |  |  |  |  |  |  |  |  |  |  |  |  |  |  |  |  |  |  |  |  |  |  |  |  |  |  |  |  |  |  |  |  |  |  |  | Theo. | | | | | | | | | | | | | | | | | | | | | | | | | | | | | | | | | | | | | | | | Exp. | | | | | | | | | | | | | | | | | | | | | | | | | | | | | | | | | | | | | | | | | | | | | | | | | | | | | | | | | | | | | | | | | | | | | | | | | | | | | | | | | | | | | | | | | | | |  |  |  |  |  |  |  |  |  |  |  |  |  |  |  |  |  |  |  |  |  |  |  |  |  |  |  |  |  |  |  |  |  |  |  |  |  |  |  |  |  |  |  |  |  |  |  |  |  |  |  |  |  |  |  |  |  |  |  |  |  |  |  |  |  |  |  |  |  |  |  |  |  |  |
| 2c | | | | | | | enolase | | | | | | | | | | | | | | | | | | Glyma03g34830.1 | | | | | | | | | | | | | | | | | | | | | | | | | | | | | | | | 153 | | | | | | | | | | | | | | | | | | | | | | | | | | | | | | 1 | | | | | | | | | | | | | | | | | | | | | | | | | | | | | | | | | | | 4 | | | | | | | | | | | | | | | | | | | | | | | | | | | | | | | | | | | | 863 | | | | | | | | | | | | | | | | | | | | | | | | | | | | | | | | | | | | | | | | | | | | 48.1/5.49 | | | | | | | | | | | | | | | | | | | | | | | | | | | | | | | | | | | | | | | |  | | | | | | | | | | | | | | | | | | | | | | | | | | | | | | | | | | | | | | | | | | | | | | | | | | | | | | | | | | | | | | | | | | | | | | | | | | | | | | | | | | | | | | | | | | | |  | | | | | | | | | | | | | | | | | | | | | | | | | | | | | | | | | | | | | | | | | | | | | |  |  |  |  |  |  |  |  |  |  |  |  |  |  |  |  |  |  |  |  |  |  |  |  |  |  |  |  |
|  | | | | | | | Calc. Mass | | | | | | | | | | | | | | | | | | Observ. Mass | | | | | | | | | | | | | | | | | | | | | | | | | | | | | | | | Da | | | | | | | | | | | | | | | | | | | | | | | | | | | | | | ppm | | | | | | | | | | | | | | | | | | | | | | | | | | | | | | | | | | | start | | | | | | | | | | | | | | | | | | | | | | | | | | | | | | | | | | | | end | | | | | | | | | | | | | | | | | | | | | | | | | | | | | | | | | | | | | | | | | | | | sequence | | | | | | | | | | | | | | | | | | | | | | | | | | | | | | | | | | | | | | | | | | | | | | | | | | | | | | | | | | | | | | | | | | | | | | | | | | | | | | | | | | | | | | | | | | | | | | | | | | | | | | | | | | | | | | | | | | | | | | | | | | | | | | | | | | | | | | | | | | | | | | | | | | | | | | | | | | | | | | | | | | | | | | | | | | | | | | | | | |  |  |  |  |  |  |  |  |  |  |  |  |  |  |  |  |  |  |  |  |  |  |  |  |  |  |  |  |
|  | | | | | | | 2251.122 | | | | | | | | | | | | | | | | | | 2251.119 | | | | | | | | | | | | | | | | | | | | | | | | | | | | | | | | 0 | | | | | | | | | | | | | | | | | | | | | | | | | | | | | | -1 | | | | | | | | | | | | | | | | | | | | | | | | | | | | | | | | | | | 382 | | | | | | | | | | | | | | | | | | | | | | | | | | | | | | | | | | | | 403 | | | | | | | | | | | | | | | | | | | | | | | | | | | | | | | | | | | | | | | | | | | | SGETEDTFIADLSVGLATGQIK | | | | | | | | | | | | | | | | | | | | | | | | | | | | | | | | | | | | | | | | | | | | | | | | | | | | | | | | | | | | | | | | | | | | | | | | | | | | | | | | | | | | | | | | | | | | | | | | | | | | | | | | | | | | | | | | | | | | | | | | | | | | | | | | | | | | | | | | | | | | | | | | | | | | | | | | | | | | | | | | | | | | | | | | | | | | | | | | | |  |  |  |  |  |  |  |  |  |  |  |  |  |  |  |  |  |  |  |  |  |  |  |  |  |  |  |  |
| Spot no. | | | | | | | Homologous protein | | | | | | | | | | | | | | | | | | | | | | | | | Accession no. | | | | | | | | | | | | | | | | | | | | | | | | | | | | | | | | Score | | | | | | | | | | | | | | | | | | | | | | | | | | | | | M.P. | | | | | | | | | | | | | | | | | | | | | | | | | | | | | | | | | | | | | Cov. (%) | | | | | | | | | | | | | | | | | | | | | | | | | | | | | | | | | | | | | | | Blast score | | | | | | | | | | | | | | | | | | | | | | | | | | | | | | | | | | | | | | | | | | Mr (kDa) / pI | | | | | | | | | | | | | | | | | | | | | | | | | | | | | | | | | | | | | | | | | | | | | | | | | | | | | | | | | | | | | | | | | | | | | | | | | | | | | | | | | | | | | | | | | | | | | | | | | | | | | | | | | | | | | | | | | | | | | | | | | | | | | | Ratio | | | | | | | | | | | | | | | | | | | | | | | | | | | | | | | | | | | | | | | | | | |  |  |  |  |  |  |  |  |  |  |  |  |  |  |  |  |  |  |  |  |  |  |  |  |  |  |  |  |
|  |  |  |  |  |  |  |  |  |  |  |  |  |  |  |  |  |  |  |  |  |  |  |  |  |  |  |  |  |  |  |  |  |  |  |  |  |  |  |  |  |  |  |  |  |  |  |  |  |  |  |  |  |  |  |  |  |  |  |  |  |  |  |  |  |  |  |  |  |  |  |  |  |  |  |  |  |  |  |  |  |  |  |  |  |  |  |  |  |  |  |  |  |  |  |  |  |  |  |  |  |  |  |  |  |  |  |  |  |  |  |  |  |  |  |  |  |  |  |  |  |  |  |  |  |  |  |  |  |  |  |  |  |  |  |  |  |  |  |  |  |  |  |  |  |  |  |  |  |  |  |  |  |  |  |  |  |  |  |  |  |  |  |  |  |  |  |  |  |  |  |  |  |  |  |  |  |  |  |  |  |  |  |  |  |  |  |  |  |  |  |  |  |  |  |  |  |  |  |  |  |  |  |  |  |  |  |  |  |  |  | Theo. | | | | | | | | | | | | | | | | | | | | | | | | | | | | | | | | | | | | | | | | | | | | | | | | | | | | | | | | | | | | | | | Exp. | | | | | | | | | | | | | | | | | | | | | | | | | | | | | | | | | | | | | | | | | | | | | | | | | | | | | | | | | | | | | | |  |  |  |  |  |  |  |  |  |  |  |  |  |  |  |  |  |  |  |  |  |  |  |  |  |  |  |  |  |  |  |  |  |  |  |  |  |  |  |  |  |  |  |  |  |  |  |  |  |  |  |  |  |  |  |  |  |  |  |  |  |  |  |  |  |  |  |  |  |  |  |
| 2d | | | | | | | uncharacterized protein LOC100499665 | | | | | | | | | | | | | | | | | | | | | | | | | Glyma03g40110.1 | | | | | | | | | | | | | | | | | | | | | | | | | | | | | | | | 147 | | | | | | | | | | | | | | | | | | | | | | | | | | | | | 3 | | | | | | | | | | | | | | | | | | | | | | | | | | | | | | | | | | | | | 22 | | | | | | | | | | | | | | | | | | | | | | | | | | | | | | | | | | | | | | | 243 | | | | | | | | | | | | | | | | | | | | | | | | | | | | | | | | | | | | | | | | | | 16.6/10.87 | | | | | | | | | | | | | | | | | | | | | | | | | | | | | | | | | | | | | | | | | | | | | | | | | | | | | | | | | | | | | | |  | | | | | | | | | | | | | | | | | | | | | | | | | | | | | | | | | | | | | | | | | | | | | | | | | | | | | | | | | | | | | | |  | | | | | | | | | | | | | | | | | | | | | | | | | | | | | | | | | | | | | | | | | | |  |  |  |  |  |  |  |  |  |  |  |  |  |  |  |  |  |  |  |  |  |  |  |  |  |  |  |  |
|  | | | | | | | Calc. Mass | | | | | | | | | | | | | | | | | | | | | | | | | Observ. Mass | | | | | | | | | | | | | | | | | | | | | | | | | | | | | | | | Da | | | | | | | | | | | | | | | | | | | | | | | | | | | | | ppm | | | | | | | | | | | | | | | | | | | | | | | | | | | | | | | | | | | | | start | | | | | | | | | | | | | | | | | | | | | | | | | | | | | | | | | | | | | | | end | | | | | | | | | | | | | | | | | | | | | | | | | | | | | | | | | | | | | | | | | | sequence | | | | | | | | | | | | | | | | | | | | | | | | | | | | | | | | | | | | | | | | | | | | | | | | | | | | | | | | | | | | | | | | | | | | | | | | | | | | | | | | | | | | | | | | | | | | | | | | | | | | | | | | | | | | | | | | | | | | | | | | | | | | | | | | | | | | | | | | | | | | | | | | | | | | | | | | | | | | | | | | | | | | | | | | |  |  |  |  |  |  |  |  |  |  |  |  |  |  |  |  |  |  |  |  |  |  |  |  |  |  |  |  |
|  | | | | | | | 1880.8574 | | | | | | | | | | | | | | | | | | | | | | | | | 1880.853 | | | | | | | | | | | | | | | | | | | | | | | | | | | | | | | | 0 | | | | | | | | | | | | | | | | | | | | | | | | | | | | | -2 | | | | | | | | | | | | | | | | | | | | | | | | | | | | | | | | | | | | | 66 | | | | | | | | | | | | | | | | | | | | | | | | | | | | | | | | | | | | | | | 83 | | | | | | | | | | | | | | | | | | | | | | | | | | | | | | | | | | | | | | | | | | DESSPYAAMLAAQDVAAR | | | | | | | | | | | | | | | | | | | | | | | | | | | | | | | | | | | | | | | | | | | | | | | | | | | | | | | | | | | | | | | | | | | | | | | | | | | | | | | | | | | | | | | | | | | | | | | | | | | | | | | | | | | | | | | | | | | | | | | | | | | | | | | | | | | | | | | | | | | | | | | | | | | | | | | | | | | | | | | | | | | | | | | | |  |  |  |  |  |  |  |  |  |  |  |  |  |  |  |  |  |  |  |  |  |  |  |  |  |  |  |  |
|  | | | | | | | 1880.8574 | | | | | | | | | | | | | | | | | | | | | | | | | 1880.856 | | | | | | | | | | | | | | | | | | | | | | | | | | | | | | | | 0 | | | | | | | | | | | | | | | | | | | | | | | | | | | | | -1 | | | | | | | | | | | | | | | | | | | | | | | | | | | | | | | | | | | | | 66 | | | | | | | | | | | | | | | | | | | | | | | | | | | | | | | | | | | | | | | 83 | | | | | | | | | | | | | | | | | | | | | | | | | | | | | | | | | | | | | | | | | | DESSPYAAMLAAQDVAAR | | | | | | | | | | | | | | | | | | | | | | | | | | | | | | | | | | | | | | | | | | | | | | | | | | | | | | | | | | | | | | | | | | | | | | | | | | | | | | | | | | | | | | | | | | | | | | | | | | | | | | | | | | | | | | | | | | | | | | | | | | | | | | | | | | | | | | | | | | | | | | | | | | | | | | | | | | | | | | | | | | | | | | | | |  |  |  |  |  |  |  |  |  |  |  |  |  |  |  |  |  |  |  |  |  |  |  |  |  |  |  |  |
|  | | | | | | | 1754.9163 | | | | | | | | | | | | | | | | | | | | | | | | | 1754.916 | | | | | | | | | | | | | | | | | | | | | | | | | | | | | | | | 0 | | | | | | | | | | | | | | | | | | | | | | | | | | | | | 0 | | | | | | | | | | | | | | | | | | | | | | | | | | | | | | | | | | | | | 125 | | | | | | | | | | | | | | | | | | | | | | | | | | | | | | | | | | | | | | | 140 | | | | | | | | | | | | | | | | | | | | | | | | | | | | | | | | | | | | | | | | | | IGRIEDVTPIPSDSTR | | | | | | | | | | | | | | | | | | | | | | | | | | | | | | | | | | | | | | | | | | | | | | | | | | | | | | | | | | | | | | | | | | | | | | | | | | | | | | | | | | | | | | | | | | | | | | | | | | | | | | | | | | | | | | | | | | | | | | | | | | | | | | | | | | | | | | | | | | | | | | | | | | | | | | | | | | | | | | | | | | | | | | | | |  |  |  |  |  |  |  |  |  |  |  |  |  |  |  |  |  |  |  |  |  |  |  |  |  |  |  |  |
|  | | | | | | | 1428.7096 | | | | | | | | | | | | | | | | | | | | | | | | | 1428.704 | | | | | | | | | | | | | | | | | | | | | | | | | | | | | | | | 0 | | | | | | | | | | | | | | | | | | | | | | | | | | | | | -4 | | | | | | | | | | | | | | | | | | | | | | | | | | | | | | | | | | | | | 128 | | | | | | | | | | | | | | | | | | | | | | | | | | | | | | | | | | | | | | | 140 | | | | | | | | | | | | | | | | | | | | | | | | | | | | | | | | | | | | | | | | | | IEDVTPIPSDSTR | | | | | | | | | | | | | | | | | | | | | | | | | | | | | | | | | | | | | | | | | | | | | | | | | | | | | | | | | | | | | | | | | | | | | | | | | | | | | | | | | | | | | | | | | | | | | | | | | | | | | | | | | | | | | | | | | | | | | | | | | | | | | | | | | | | | | | | | | | | | | | | | | | | | | | | | | | | | | | | | | | | | | | | | |  |  |  |  |  |  |  |  |  |  |  |  |  |  |  |  |  |  |  |  |  |  |  |  |  |  |  |  |
|  | | | | | | | 1428.7096 | | | | | | | | | | | | | | | | | | | | | | | | | 1428.707 | | | | | | | | | | | | | | | | | | | | | | | | | | | | | | | | 0 | | | | | | | | | | | | | | | | | | | | | | | | | | | | | -2 | | | | | | | | | | | | | | | | | | | | | | | | | | | | | | | | | | | | | 128 | | | | | | | | | | | | | | | | | | | | | | | | | | | | | | | | | | | | | | | 140 | | | | | | | | | | | | | | | | | | | | | | | | | | | | | | | | | | | | | | | | | | IEDVTPIPSDSTR | | | | | | | | | | | | | | | | | | | | | | | | | | | | | | | | | | | | | | | | | | | | | | | | | | | | | | | | | | | | | | | | | | | | | | | | | | | | | | | | | | | | | | | | | | | | | | | | | | | | | | | | | | | | | | | | | | | | | | | | | | | | | | | | | | | | | | | | | | | | | | | | | | | | | | | | | | | | | | | | | | | | | | | | |  |  |  |  |  |  |  |  |  |  |  |  |  |  |  |  |  |  |  |  |  |  |  |  |  |  |  |  |
| Spot no. | | | | | | | Homologous protein | | | | | | | | | | | | | | | | | | | | | | | | | Accession no. | | | | | | | | | | | | | | | | | | | | | | | | | | | | | | | | Score | | | | | | | | | | | | | | | | | | | | | | | | | | | | | M.P. | | | | | | | | | | | | | | | | | | | | | | | | | | | | | | | | | | | | | Cov. (%) | | | | | | | | | | | | | | | | | | | | | | | | | | | | | | | | | | | | | | | Blast score | | | | | | | | | | | | | | | | | | | | | | | | | | | | | | | | | | | | | | | | | | Mr (kDa) / pI | | | | | | | | | | | | | | | | | | | | | | | | | | | | | | | | | | | | | | | | | | | | | | | | | | | | | | | | | | | | | | | | | | | | | | | | | | | | | | | | | | | | | | | | | | | | | | | | | | | | | | Ratio | | | | | | | | | | | | | | | | | | | | | | | | | | | | | | | | | | | | | | | | | | | | | | | | | | | | | | | | | | | | | | | | | | |  |  |  |  |  |  |  |  |  |  |  |  |  |  |  |  |  |  |  |  |  |  |  |  |  |  |  |  |
|  |  |  |  |  |  |  |  |  |  |  |  |  |  |  |  |  |  |  |  |  |  |  |  |  |  |  |  |  |  |  |  |  |  |  |  |  |  |  |  |  |  |  |  |  |  |  |  |  |  |  |  |  |  |  |  |  |  |  |  |  |  |  |  |  |  |  |  |  |  |  |  |  |  |  |  |  |  |  |  |  |  |  |  |  |  |  |  |  |  |  |  |  |  |  |  |  |  |  |  |  |  |  |  |  |  |  |  |  |  |  |  |  |  |  |  |  |  |  |  |  |  |  |  |  |  |  |  |  |  |  |  |  |  |  |  |  |  |  |  |  |  |  |  |  |  |  |  |  |  |  |  |  |  |  |  |  |  |  |  |  |  |  |  |  |  |  |  |  |  |  |  |  |  |  |  |  |  |  |  |  |  |  |  |  |  |  |  |  |  |  |  |  |  |  |  |  |  |  |  |  |  |  |  |  |  |  |  |  |  |  | Theo. | | | | | | | | | | | | | | | | | | | | | | | | | | | | | | | | | | | | | | | | | | | | | | | | | | | | | | | | | | | | | | | Exp. | | | | | | | | | | | | | | | | | | | | | | | | | | | | | | | | | | | | | | |  |  |  |  |  |  |  |  |  |  |  |  |  |  |  |  |  |  |  |  |  |  |  |  |  |  |  |  |  |  |  |  |  |  |  |  |  |  |  |  |  |  |  |  |  |  |  |  |  |  |  |  |  |  |  |  |  |  |  |  |  |  |  |  |  |  |  |  |  |  |  |  |  |  |  |  |  |  |  |  |  |  |  |  |  |  |  |  |  |  |  |  |  |  |  |
| 3a | | | | | | | unknown | | | | | | | | | | | | | | | | | | | | | | | | | Glyma09g29030.1 | | | | | | | | | | | | | | | | | | | | | | | | | | | | | | | | 765 | | | | | | | | | | | | | | | | | | | | | | | | | | | | | 10 | | | | | | | | | | | | | | | | | | | | | | | | | | | | | | | | | | | | | 33 | | | | | | | | | | | | | | | | | | | | | | | | | | | | | | | | | | | | | | | 468 | | | | | | | | | | | | | | | | | | | | | | | | | | | | | | | | | | | | | | | | | | 31.4/5.07 | | | | | | | | | | | | | | | | | | | | | | | | | | | | | | | | | | | | | | | | | | | | | | | | | | | | | | | | | | | | | | |  | | | | | | | | | | | | | | | | | | | | | | | | | | | | | | | | | | | | | | |  | | | | | | | | | | | | | | | | | | | | | | | | | | | | | | | | | | | | | | | | | | | | | | | | | | | | | | | | | | | | | | | | | | |  |  |  |  |  |  |  |  |  |  |  |  |  |  |  |  |  |  |  |  |  |  |  |  |  |  |  |  |
|  | | | | | | | Calc. Mass | | | | | | | | | | | | | | | | | | | | | | | | | Observ. Mass | | | | | | | | | | | | | | | | | | | | | | | | | | | | | | | | Da | | | | | | | | | | | | | | | | | | | | | | | | | | | | | ppm | | | | | | | | | | | | | | | | | | | | | | | | | | | | | | | | | | | | | start | | | | | | | | | | | | | | | | | | | | | | | | | | | | | | | | | | | | | | | end | | | | | | | | | | | | | | | | | | | | | | | | | | | | | | | | | | | | | | | | | | sequence | | | | | | | | | | | | | | | | | | | | | | | | | | | | | | | | | | | | | | | | | | | | | | | | | | | | | | | | | | | | | | | | | | | | | | | | | | | | | | | | | | | | | | | | | | | | | | | | | | | | | | | | | | | | | | | | | | | | | | | | | | | | | | | | | | | | | | | | | | | | | | | | | | | | | | | | | | | | | | | | | | | | | | | | |  |  |  |  |  |  |  |  |  |  |  |  |  |  |  |  |  |  |  |  |  |  |  |  |  |  |  |  |
|  | | | | | | | 1709.7645 | | | | | | | | | | | | | | | | | | | | | | | | | 1709.761 | | | | | | | | | | | | | | | | | | | | | | | | | | | | | | | | 0 | | | | | | | | | | | | | | | | | | | | | | | | | | | | | -2 | | | | | | | | | | | | | | | | | | | | | | | | | | | | | | | | | | | | | 4 | | | | | | | | | | | | | | | | | | | | | | | | | | | | | | | | | | | | | | | 18 | | | | | | | | | | | | | | | | | | | | | | | | | | | | | | | | | | | | | | | | | | NQYDTDVTTWSPAGR | | | | | | | | | | | | | | | | | | | | | | | | | | | | | | | | | | | | | | | | | | | | | | | | | | | | | | | | | | | | | | | | | | | | | | | | | | | | | | | | | | | | | | | | | | | | | | | | | | | | | | | | | | | | | | | | | | | | | | | | | | | | | | | | | | | | | | | | | | | | | | | | | | | | | | | | | | | | | | | | | | | | | | | | |  |  |  |  |  |  |  |  |  |  |  |  |  |  |  |  |  |  |  |  |  |  |  |  |  |  |  |  |
|  | | | | | | | 1709.7645 | | | | | | | | | | | | | | | | | | | | | | | | | 1709.762 | | | | | | | | | | | | | | | | | | | | | | | | | | | | | | | | 0 | | | | | | | | | | | | | | | | | | | | | | | | | | | | | -1 | | | | | | | | | | | | | | | | | | | | | | | | | | | | | | | | | | | | | 4 | | | | | | | | | | | | | | | | | | | | | | | | | | | | | | | | | | | | | | | 18 | | | | | | | | | | | | | | | | | | | | | | | | | | | | | | | | | | | | | | | | | | NQYDTDVTTWSPAGR | | | | | | | | | | | | | | | | | | | | | | | | | | | | | | | | | | | | | | | | | | | | | | | | | | | | | | | | | | | | | | | | | | | | | | | | | | | | | | | | | | | | | | | | | | | | | | | | | | | | | | | | | | | | | | | | | | | | | | | | | | | | | | | | | | | | | | | | | | | | | | | | | | | | | | | | | | | | | | | | | | | | | | | | |  |  |  |  |  |  |  |  |  |  |  |  |  |  |  |  |  |  |  |  |  |  |  |  |  |  |  |  |
|  | | | | | | | 1709.7645 | | | | | | | | | | | | | | | | | | | | | | | | | 1709.763 | | | | | | | | | | | | | | | | | | | | | | | | | | | | | | | | 0 | | | | | | | | | | | | | | | | | | | | | | | | | | | | | -1 | | | | | | | | | | | | | | | | | | | | | | | | | | | | | | | | | | | | | 4 | | | | | | | | | | | | | | | | | | | | | | | | | | | | | | | | | | | | | | | 18 | | | | | | | | | | | | | | | | | | | | | | | | | | | | | | | | | | | | | | | | | | NQYDTDVTTWSPAGR | | | | | | | | | | | | | | | | | | | | | | | | | | | | | | | | | | | | | | | | | | | | | | | | | | | | | | | | | | | | | | | | | | | | | | | | | | | | | | | | | | | | | | | | | | | | | | | | | | | | | | | | | | | | | | | | | | | | | | | | | | | | | | | | | | | | | | | | | | | | | | | | | | | | | | | | | | | | | | | | | | | | | | | | |  |  |  |  |  |  |  |  |  |  |  |  |  |  |  |  |  |  |  |  |  |  |  |  |  |  |  |  |
|  | | | | | | | 1709.7645 | | | | | | | | | | | | | | | | | | | | | | | | | 1709.763 | | | | | | | | | | | | | | | | | | | | | | | | | | | | | | | | 0 | | | | | | | | | | | | | | | | | | | | | | | | | | | | | -1 | | | | | | | | | | | | | | | | | | | | | | | | | | | | | | | | | | | | | 4 | | | | | | | | | | | | | | | | | | | | | | | | | | | | | | | | | | | | | | | 18 | | | | | | | | | | | | | | | | | | | | | | | | | | | | | | | | | | | | | | | | | | NQYDTDVTTWSPAGR | | | | | | | | | | | | | | | | | | | | | | | | | | | | | | | | | | | | | | | | | | | | | | | | | | | | | | | | | | | | | | | | | | | | | | | | | | | | | | | | | | | | | | | | | | | | | | | | | | | | | | | | | | | | | | | | | | | | | | | | | | | | | | | | | | | | | | | | | | | | | | | | | | | | | | | | | | | | | | | | | | | | | | | | |  |  |  |  |  |  |  |  |  |  |  |  |  |  |  |  |  |  |  |  |  |  |  |  |  |  |  |  |
|  | | | | | | | 1709.7645 | | | | | | | | | | | | | | | | | | | | | | | | | 1709.763 | | | | | | | | | | | | | | | | | | | | | | | | | | | | | | | | 0 | | | | | | | | | | | | | | | | | | | | | | | | | | | | | -1 | | | | | | | | | | | | | | | | | | | | | | | | | | | | | | | | | | | | | 4 | | | | | | | | | | | | | | | | | | | | | | | | | | | | | | | | | | | | | | | 18 | | | | | | | | | | | | | | | | | | | | | | | | | | | | | | | | | | | | | | | | | | NQYDTDVTTWSPAGR | | | | | | | | | | | | | | | | | | | | | | | | | | | | | | | | | | | | | | | | | | | | | | | | | | | | | | | | | | | | | | | | | | | | | | | | | | | | | | | | | | | | | | | | | | | | | | | | | | | | | | | | | | | | | | | | | | | | | | | | | | | | | | | | | | | | | | | | | | | | | | | | | | | | | | | | | | | | | | | | | | | | | | | | |  |  |  |  |  |  |  |  |  |  |  |  |  |  |  |  |  |  |  |  |  |  |  |  |  |  |  |  |
|  | | | | | | | 1709.7645 | | | | | | | | | | | | | | | | | | | | | | | | | 1709.763 | | | | | | | | | | | | | | | | | | | | | | | | | | | | | | | | 0 | | | | | | | | | | | | | | | | | | | | | | | | | | | | | -1 | | | | | | | | | | | | | | | | | | | | | | | | | | | | | | | | | | | | | 4 | | | | | | | | | | | | | | | | | | | | | | | | | | | | | | | | | | | | | | | 18 | | | | | | | | | | | | | | | | | | | | | | | | | | | | | | | | | | | | | | | | | | NQYDTDVTTWSPAGR | | | | | | | | | | | | | | | | | | | | | | | | | | | | | | | | | | | | | | | | | | | | | | | | | | | | | | | | | | | | | | | | | | | | | | | | | | | | | | | | | | | | | | | | | | | | | | | | | | | | | | | | | | | | | | | | | | | | | | | | | | | | | | | | | | | | | | | | | | | | | | | | | | | | | | | | | | | | | | | | | | | | | | | | |  |  |  |  |  |  |  |  |  |  |  |  |  |  |  |  |  |  |  |  |  |  |  |  |  |  |  |  |
|  | | | | | | | 1709.7645 | | | | | | | | | | | | | | | | | | | | | | | | | 1709.763 | | | | | | | | | | | | | | | | | | | | | | | | | | | | | | | | 0 | | | | | | | | | | | | | | | | | | | | | | | | | | | | | -1 | | | | | | | | | | | | | | | | | | | | | | | | | | | | | | | | | | | | | 4 | | | | | | | | | | | | | | | | | | | | | | | | | | | | | | | | | | | | | | | 18 | | | | | | | | | | | | | | | | | | | | | | | | | | | | | | | | | | | | | | | | | | NQYDTDVTTWSPAGR | | | | | | | | | | | | | | | | | | | | | | | | | | | | | | | | | | | | | | | | | | | | | | | | | | | | | | | | | | | | | | | | | | | | | | | | | | | | | | | | | | | | | | | | | | | | | | | | | | | | | | | | | | | | | | | | | | | | | | | | | | | | | | | | | | | | | | | | | | | | | | | | | | | | | | | | | | | | | | | | | | | | | | | | |  |  |  |  |  |  |  |  |  |  |  |  |  |  |  |  |  |  |  |  |  |  |  |  |  |  |  |  |
|  | | | | | | | 1426.7166 | | | | | | | | | | | | | | | | | | | | | | | | | 1426.716 | | | | | | | | | | | | | | | | | | | | | | | | | | | | | | | | 0 | | | | | | | | | | | | | | | | | | | | | | | | | | | | | 0 | | | | | | | | | | | | | | | | | | | | | | | | | | | | | | | | | | | | | 19 | | | | | | | | | | | | | | | | | | | | | | | | | | | | | | | | | | | | | | | 30 | | | | | | | | | | | | | | | | | | | | | | | | | | | | | | | | | | | | | | | | | | LFQVEYAMEAVK | | | | | | | | | | | | | | | | | | | | | | | | | | | | | | | | | | | | | | | | | | | | | | | | | | | | | | | | | | | | | | | | | | | | | | | | | | | | | | | | | | | | | | | | | | | | | | | | | | | | | | | | | | | | | | | | | | | | | | | | | | | | | | | | | | | | | | | | | | | | | | | | | | | | | | | | | | | | | | | | | | | | | | | | |  |  |  |  |  |  |  |  |  |  |  |  |  |  |  |  |  |  |  |  |  |  |  |  |  |  |  |  |
|  | | | | | | | 1442.7115 | | | | | | | | | | | | | | | | | | | | | | | | | 1442.711 | | | | | | | | | | | | | | | | | | | | | | | | | | | | | | | | 0 | | | | | | | | | | | | | | | | | | | | | | | | | | | | | -1 | | | | | | | | | | | | | | | | | | | | | | | | | | | | | | | | | | | | | 19 | | | | | | | | | | | | | | | | | | | | | | | | | | | | | | | | | | | | | | | 30 | | | | | | | | | | | | | | | | | | | | | | | | | | | | | | | | | | | | | | | | | | LFQVEYAMEAVK | | | | | | | | | | | | | | | | | | | | | | | | | | | | | | | | | | | | | | | | | | | | | | | | | | | | | | | | | | | | | | | | | | | | | | | | | | | | | | | | | | | | | | | | | | | | | | | | | | | | | | | | | | | | | | | | | | | | | | | | | | | | | | | | | | | | | | | | | | | | | | | | | | | | | | | | | | | | | | | | | | | | | | | | |  |  |  |  |  |  |  |  |  |  |  |  |  |  |  |  |  |  |  |  |  |  |  |  |  |  |  |  |
|  | | | | | | | 1442.7115 | | | | | | | | | | | | | | | | | | | | | | | | | 1442.711 | | | | | | | | | | | | | | | | | | | | | | | | | | | | | | | | 0 | | | | | | | | | | | | | | | | | | | | | | | | | | | | | -1 | | | | | | | | | | | | | | | | | | | | | | | | | | | | | | | | | | | | | 19 | | | | | | | | | | | | | | | | | | | | | | | | | | | | | | | | | | | | | | | 30 | | | | | | | | | | | | | | | | | | | | | | | | | | | | | | | | | | | | | | | | | | LFQVEYAMEAVK | | | | | | | | | | | | | | | | | | | | | | | | | | | | | | | | | | | | | | | | | | | | | | | | | | | | | | | | | | | | | | | | | | | | | | | | | | | | | | | | | | | | | | | | | | | | | | | | | | | | | | | | | | | | | | | | | | | | | | | | | | | | | | | | | | | | | | | | | | | | | | | | | | | | | | | | | | | | | | | | | | | | | | | | |  |  |  |  |  |  |  |  |  |  |  |  |  |  |  |  |  |  |  |  |  |  |  |  |  |  |  |  |
|  | | | | | | | 1442.7115 | | | | | | | | | | | | | | | | | | | | | | | | | 1442.711 | | | | | | | | | | | | | | | | | | | | | | | | | | | | | | | | 0 | | | | | | | | | | | | | | | | | | | | | | | | | | | | | 0 | | | | | | | | | | | | | | | | | | | | | | | | | | | | | | | | | | | | | 19 | | | | | | | | | | | | | | | | | | | | | | | | | | | | | | | | | | | | | | | 30 | | | | | | | | | | | | | | | | | | | | | | | | | | | | | | | | | | | | | | | | | | LFQVEYAMEAVK | | | | | | | | | | | | | | | | | | | | | | | | | | | | | | | | | | | | | | | | | | | | | | | | | | | | | | | | | | | | | | | | | | | | | | | | | | | | | | | | | | | | | | | | | | | | | | | | | | | | | | | | | | | | | | | | | | | | | | | | | | | | | | | | | | | | | | | | | | | | | | | | | | | | | | | | | | | | | | | | | | | | | | | | |  |  |  |  |  |  |  |  |  |  |  |  |  |  |  |  |  |  |  |  |  |  |  |  |  |  |  |  |
|  | | | | | | | 1442.7115 | | | | | | | | | | | | | | | | | | | | | | | | | 1442.711 | | | | | | | | | | | | | | | | | | | | | | | | | | | | | | | | 0 | | | | | | | | | | | | | | | | | | | | | | | | | | | | | 0 | | | | | | | | | | | | | | | | | | | | | | | | | | | | | | | | | | | | | 19 | | | | | | | | | | | | | | | | | | | | | | | | | | | | | | | | | | | | | | | 30 | | | | | | | | | | | | | | | | | | | | | | | | | | | | | | | | | | | | | | | | | | LFQVEYAMEAVK | | | | | | | | | | | | | | | | | | | | | | | | | | | | | | | | | | | | | | | | | | | | | | | | | | | | | | | | | | | | | | | | | | | | | | | | | | | | | | | | | | | | | | | | | | | | | | | | | | | | | | | | | | | | | | | | | | | | | | | | | | | | | | | | | | | | | | | | | | | | | | | | | | | | | | | | | | | | | | | | | | | | | | | | |  |  |  |  |  |  |  |  |  |  |  |  |  |  |  |  |  |  |  |  |  |  |  |  |  |  |  |  |
|  | | | | | | | 871.4875 | | | | | | | | | | | | | | | | | | | | | | | | | 871.4876 | | | | | | | | | | | | | | | | | | | | | | | | | | | | | | | | 0 | | | | | | | | | | | | | | | | | | | | | | | | | | | | | 0 | | | | | | | | | | | | | | | | | | | | | | | | | | | | | | | | | | | | | 31 | | | | | | | | | | | | | | | | | | | | | | | | | | | | | | | | | | | | | | | 39 | | | | | | | | | | | | | | | | | | | | | | | | | | | | | | | | | | | | | | | | | | QGSAAIGLR | | | | | | | | | | | | | | | | | | | | | | | | | | | | | | | | | | | | | | | | | | | | | | | | | | | | | | | | | | | | | | | | | | | | | | | | | | | | | | | | | | | | | | | | | | | | | | | | | | | | | | | | | | | | | | | | | | | | | | | | | | | | | | | | | | | | | | | | | | | | | | | | | | | | | | | | | | | | | | | | | | | | | | | | |  |  |  |  |  |  |  |  |  |  |  |  |  |  |  |  |  |  |  |  |  |  |  |  |  |  |  |  |
|  | | | | | | | 1354.7391 | | | | | | | | | | | | | | | | | | | | | | | | | 1354.738 | | | | | | | | | | | | | | | | | | | | | | | | | | | | | | | | 0 | | | | | | | | | | | | | | | | | | | | | | | | | | | | | -1 | | | | | | | | | | | | | | | | | | | | | | | | | | | | | | | | | | | | | 40 | | | | | | | | | | | | | | | | | | | | | | | | | | | | | | | | | | | | | | | 51 | | | | | | | | | | | | | | | | | | | | | | | | | | | | | | | | | | | | | | | | | | SKTHVVLACVNK | | | | | | | | | | | | | | | | | | | | | | | | | | | | | | | | | | | | | | | | | | | | | | | | | | | | | | | | | | | | | | | | | | | | | | | | | | | | | | | | | | | | | | | | | | | | | | | | | | | | | | | | | | | | | | | | | | | | | | | | | | | | | | | | | | | | | | | | | | | | | | | | | | | | | | | | | | | | | | | | | | | | | | | | |  |  |  |  |  |  |  |  |  |  |  |  |  |  |  |  |  |  |  |  |  |  |  |  |  |  |  |  |
|  | | | | | | | 1354.7391 | | | | | | | | | | | | | | | | | | | | | | | | | 1354.738 | | | | | | | | | | | | | | | | | | | | | | | | | | | | | | | | 0 | | | | | | | | | | | | | | | | | | | | | | | | | | | | | -1 | | | | | | | | | | | | | | | | | | | | | | | | | | | | | | | | | | | | | 40 | | | | | | | | | | | | | | | | | | | | | | | | | | | | | | | | | | | | | | | 51 | | | | | | | | | | | | | | | | | | | | | | | | | | | | | | | | | | | | | | | | | | SKTHVVLACVNK | | | | | | | | | | | | | | | | | | | | | | | | | | | | | | | | | | | | | | | | | | | | | | | | | | | | | | | | | | | | | | | | | | | | | | | | | | | | | | | | | | | | | | | | | | | | | | | | | | | | | | | | | | | | | | | | | | | | | | | | | | | | | | | | | | | | | | | | | | | | | | | | | | | | | | | | | | | | | | | | | | | | | | | | |  |  |  |  |  |  |  |  |  |  |  |  |  |  |  |  |  |  |  |  |  |  |  |  |  |  |  |  |
|  | | | | | | | 1354.7391 | | | | | | | | | | | | | | | | | | | | | | | | | 1354.738 | | | | | | | | | | | | | | | | | | | | | | | | | | | | | | | | 0 | | | | | | | | | | | | | | | | | | | | | | | | | | | | | -1 | | | | | | | | | | | | | | | | | | | | | | | | | | | | | | | | | | | | | 40 | | | | | | | | | | | | | | | | | | | | | | | | | | | | | | | | | | | | | | | 51 | | | | | | | | | | | | | | | | | | | | | | | | | | | | | | | | | | | | | | | | | | SKTHVVLACVNK | | | | | | | | | | | | | | | | | | | | | | | | | | | | | | | | | | | | | | | | | | | | | | | | | | | | | | | | | | | | | | | | | | | | | | | | | | | | | | | | | | | | | | | | | | | | | | | | | | | | | | | | | | | | | | | | | | | | | | | | | | | | | | | | | | | | | | | | | | | | | | | | | | | | | | | | | | | | | | | | | | | | | | | | |  |  |  |  |  |  |  |  |  |  |  |  |  |  |  |  |  |  |  |  |  |  |  |  |  |  |  |  |
|  | | | | | | | 1354.7391 | | | | | | | | | | | | | | | | | | | | | | | | | 1354.738 | | | | | | | | | | | | | | | | | | | | | | | | | | | | | | | | 0 | | | | | | | | | | | | | | | | | | | | | | | | | | | | | -1 | | | | | | | | | | | | | | | | | | | | | | | | | | | | | | | | | | | | | 40 | | | | | | | | | | | | | | | | | | | | | | | | | | | | | | | | | | | | | | | 51 | | | | | | | | | | | | | | | | | | | | | | | | | | | | | | | | | | | | | | | | | | SKTHVVLACVNK | | | | | | | | | | | | | | | | | | | | | | | | | | | | | | | | | | | | | | | | | | | | | | | | | | | | | | | | | | | | | | | | | | | | | | | | | | | | | | | | | | | | | | | | | | | | | | | | | | | | | | | | | | | | | | | | | | | | | | | | | | | | | | | | | | | | | | | | | | | | | | | | | | | | | | | | | | | | | | | | | | | | | | | | |  |  |  |  |  |  |  |  |  |  |  |  |  |  |  |  |  |  |  |  |  |  |  |  |  |  |  |  |
|  | | | | | | | 1354.7391 | | | | | | | | | | | | | | | | | | | | | | | | | 1354.739 | | | | | | | | | | | | | | | | | | | | | | | | | | | | | | | | 0 | | | | | | | | | | | | | | | | | | | | | | | | | | | | | 0 | | | | | | | | | | | | | | | | | | | | | | | | | | | | | | | | | | | | | 40 | | | | | | | | | | | | | | | | | | | | | | | | | | | | | | | | | | | | | | | 51 | | | | | | | | | | | | | | | | | | | | | | | | | | | | | | | | | | | | | | | | | | SKTHVVLACVNK | | | | | | | | | | | | | | | | | | | | | | | | | | | | | | | | | | | | | | | | | | | | | | | | | | | | | | | | | | | | | | | | | | | | | | | | | | | | | | | | | | | | | | | | | | | | | | | | | | | | | | | | | | | | | | | | | | | | | | | | | | | | | | | | | | | | | | | | | | | | | | | | | | | | | | | | | | | | | | | | | | | | | | | | |  |  |  |  |  |  |  |  |  |  |  |  |  |  |  |  |  |  |  |  |  |  |  |  |  |  |  |  |
|  | | | | | | | 1354.7391 | | | | | | | | | | | | | | | | | | | | | | | | | 1354.739 | | | | | | | | | | | | | | | | | | | | | | | | | | | | | | | | 0 | | | | | | | | | | | | | | | | | | | | | | | | | | | | | 0 | | | | | | | | | | | | | | | | | | | | | | | | | | | | | | | | | | | | | 40 | | | | | | | | | | | | | | | | | | | | | | | | | | | | | | | | | | | | | | | 51 | | | | | | | | | | | | | | | | | | | | | | | | | | | | | | | | | | | | | | | | | | SKTHVVLACVNK | | | | | | | | | | | | | | | | | | | | | | | | | | | | | | | | | | | | | | | | | | | | | | | | | | | | | | | | | | | | | | | | | | | | | | | | | | | | | | | | | | | | | | | | | | | | | | | | | | | | | | | | | | | | | | | | | | | | | | | | | | | | | | | | | | | | | | | | | | | | | | | | | | | | | | | | | | | | | | | | | | | | | | | | |  |  |  |  |  |  |  |  |  |  |  |  |  |  |  |  |  |  |  |  |  |  |  |  |  |  |  |  |
|  | | | | | | | 1354.7391 | | | | | | | | | | | | | | | | | | | | | | | | | 1354.739 | | | | | | | | | | | | | | | | | | | | | | | | | | | | | | | | 0 | | | | | | | | | | | | | | | | | | | | | | | | | | | | | 0 | | | | | | | | | | | | | | | | | | | | | | | | | | | | | | | | | | | | | 40 | | | | | | | | | | | | | | | | | | | | | | | | | | | | | | | | | | | | | | | 51 | | | | | | | | | | | | | | | | | | | | | | | | | | | | | | | | | | | | | | | | | | SKTHVVLACVNK | | | | | | | | | | | | | | | | | | | | | | | | | | | | | | | | | | | | | | | | | | | | | | | | | | | | | | | | | | | | | | | | | | | | | | | | | | | | | | | | | | | | | | | | | | | | | | | | | | | | | | | | | | | | | | | | | | | | | | | | | | | | | | | | | | | | | | | | | | | | | | | | | | | | | | | | | | | | | | | | | | | | | | | | |  |  |  |  |  |  |  |  |  |  |  |  |  |  |  |  |  |  |  |  |  |  |  |  |  |  |  |  |
|  | | | | | | | 1354.7391 | | | | | | | | | | | | | | | | | | | | | | | | | 1354.74 | | | | | | | | | | | | | | | | | | | | | | | | | | | | | | | | 0 | | | | | | | | | | | | | | | | | | | | | | | | | | | | | 0 | | | | | | | | | | | | | | | | | | | | | | | | | | | | | | | | | | | | | 40 | | | | | | | | | | | | | | | | | | | | | | | | | | | | | | | | | | | | | | | 51 | | | | | | | | | | | | | | | | | | | | | | | | | | | | | | | | | | | | | | | | | | SKTHVVLACVNK | | | | | | | | | | | | | | | | | | | | | | | | | | | | | | | | | | | | | | | | | | | | | | | | | | | | | | | | | | | | | | | | | | | | | | | | | | | | | | | | | | | | | | | | | | | | | | | | | | | | | | | | | | | | | | | | | | | | | | | | | | | | | | | | | | | | | | | | | | | | | | | | | | | | | | | | | | | | | | | | | | | | | | | | |  |  |  |  |  |  |  |  |  |  |  |  |  |  |  |  |  |  |  |  |  |  |  |  |  |  |  |  |
|  | | | | | | | 1354.7391 | | | | | | | | | | | | | | | | | | | | | | | | | 1354.741 | | | | | | | | | | | | | | | | | | | | | | | | | | | | | | | | 0 | | | | | | | | | | | | | | | | | | | | | | | | | | | | | 1 | | | | | | | | | | | | | | | | | | | | | | | | | | | | | | | | | | | | | 40 | | | | | | | | | | | | | | | | | | | | | | | | | | | | | | | | | | | | | | | 51 | | | | | | | | | | | | | | | | | | | | | | | | | | | | | | | | | | | | | | | | | | SKTHVVLACVNK | | | | | | | | | | | | | | | | | | | | | | | | | | | | | | | | | | | | | | | | | | | | | | | | | | | | | | | | | | | | | | | | | | | | | | | | | | | | | | | | | | | | | | | | | | | | | | | | | | | | | | | | | | | | | | | | | | | | | | | | | | | | | | | | | | | | | | | | | | | | | | | | | | | | | | | | | | | | | | | | | | | | | | | | |  |  |  |  |  |  |  |  |  |  |  |  |  |  |  |  |  |  |  |  |  |  |  |  |  |  |  |  |
|  | | | | | | | 1139.6121 | | | | | | | | | | | | | | | | | | | | | | | | | 1139.611 | | | | | | | | | | | | | | | | | | | | | | | | | | | | | | | | 0 | | | | | | | | | | | | | | | | | | | | | | | | | | | | | -1 | | | | | | | | | | | | | | | | | | | | | | | | | | | | | | | | | | | | | 42 | | | | | | | | | | | | | | | | | | | | | | | | | | | | | | | | | | | | | | | 51 | | | | | | | | | | | | | | | | | | | | | | | | | | | | | | | | | | | | | | | | | | THVVLACVNK | | | | | | | | | | | | | | | | | | | | | | | | | | | | | | | | | | | | | | | | | | | | | | | | | | | | | | | | | | | | | | | | | | | | | | | | | | | | | | | | | | | | | | | | | | | | | | | | | | | | | | | | | | | | | | | | | | | | | | | | | | | | | | | | | | | | | | | | | | | | | | | | | | | | | | | | | | | | | | | | | | | | | | | | |  |  |  |  |  |  |  |  |  |  |  |  |  |  |  |  |  |  |  |  |  |  |  |  |  |  |  |  |
[truncated: 3,440,798 more chars]
