# Supplementary material for: Proteomic and Biochemical Analyses of the Cotyledon and Root of Flooding-Stressed Soybean Plants
Source: PLoS One. 2013 Jun 14;8(6):e65301. doi: 10.1371/journal.pone.0065301 (PMC3683008; doi:10.1371/journal.pone.0065301)
Supplement: Table S4 — Peak lists for proteins identified in the cotyledon of flooding-stressed soybean plants. (DOCX) [file pone.0065301.s010.docx]

| Table S4 Peak list of the identified proteins from cotyledon of soybean under flooding stress | | | | | | | | | | | | | | | |
| --- | --- | --- | --- | --- | --- | --- | --- | --- | --- | --- | --- | --- | --- | --- | --- |
| Spot no. | Homologous protein | Accession no. | Score | | M.P. | Cov. (%) | | Blast score | | Mr (kDa) / pI | | | | Ratio | |
|  |  |  |  |  |  |  |  |  |  | Theo. | | | Exp. |  |  |
| 1 | Os05g0553000 | Glyma10g41330.1 | 317 | | 5 | 12 | | 879 | | 60.1/5.8 | | |  |  | |
|  | Calc. Mass | Observ. Mass | Da | | ppm | start | | end | | sequence | | | | | |
|  | 1617.9202 | 1617.9193 | 0 | | -1 | 122 | | 136 | | LVLEVAQHLGEGVVRTop of Form | | | | | |
|  | 1277.6286 | 1277.629 | 0 | | 0 | 137 | | 148 | | TIAMDATEGVVR | | | | | |
|  | 1408.8038 | 1408.8028 | 0 | | -1 | 152 | | 165 | | VLNTGSPITVPVGR | | | | | |
|  | 1408.8038 | 1408.8032 | 0 | | 0 | 152 | | 165 | | VLNTGSPITVPVGR | | | | | |
|  | 1398.762 | 1398.7612 | 0 | | -1 | 311 | | 323 | | VGLTGLTVAEHFR | | | | | |
|  | 1491.7681 | 1491.7634 | 0 | | -3 | 340 | | 353 | | FTQANSEVSALLGR | | | | | |
|  | 1491.7681 | 1491.767 | 0 | | -1 | 340 | | 353 | | FTQANSEVSALLGR | | | | | |
| Spot no. | Homologous protein | Accession no. | Score | | M.P. | Cov. (%) | | Blast score | | Mr (kDa) / pI | | | | Ratio | |
|  |  |  |  |  |  |  |  |  |  | Theo. | | | Exp. |  |  |
| 2a | Os05g0553000 | Glyma10g41330.1 | 191 | | 3 | 7 | | 879 | | 60.1/5.8 | | |  |  | |
|  | Calc. Mass | Observ. Mass | Da | | ppm | start | | end | | sequence | | | | | |
|  | 1617.9202 | 1617.9217 | 0 | | 1 | 122 | | 136 | | LVLEVAQHLGEGVVRTop of Form | | | | | |
|  | 1408.8038 | 1408.8032 | 0 | | 0 | 152 | | 165 | | VLNTGSPITVPVGR | | | | | |
|  | 1491.7681 | 1491.7668 | 0 | | -1 | 340 | | 353 | | FTQANSEVSALLGR | | | | | |
|  | 1491.7681 | 1491.7682 | 0 | | 0 | 340 | | 353 | | FTQANSEVSALLGR | | | | | |
| Spot no. | Homologous protein | Accession no. | Score | M.P. | | | Cov. (%) | | Blast score | | Mr (kDa) / pI | | | | Ratio |
|  |  |  |  |  |  |  |  |  |  |  | Theo. | Exp. | | |  |
| 2b | uncharacterized protein LOC100306653 | Glyma13g23980.1 | 110 | 5 | | | 30 | | 264 | | 14.4/5.73 |  | | |  |
|  | Calc. Mass | Observ. Mass | Da | ppm | | | start | | end | | sequence | | | | |
|  | 1260.6462 | 1260.6456 | 0 | 0 | | | 41 | | 52 | | DAISANSIPAFR | | | | |
|  | 1388.7412 | 1388.7409 | 0 | 0 | | | 41 | | 53 | | DAISANSIPAFRK | | | | |
|  | 1388.7412 | 1388.7418 | 0 | 0 | | | 41 | | 53 | | DAISANSIPAFRK | | | | |
|  | 1355.6867 | 1355.6857 | 0 | -1 | | | 53 | | 65 | | KGSIPGVTSDHMK | | | | |
|  | 1355.6867 | 1355.6869 | 0 | 0 | | | 53 | | 65 | | KGSIPGVTSDHMK | | | | |
|  | 1456.815 | 1456.8139 | 0 | -1 | | | 70 | | 82 | | LGVPHPLQQNLNK | | | | |
|  | 1456.815 | 1456.814 | 0 | -1 | | | 70 | | 82 | | LGVPHPLQQNLNK | | | | |
|  | 1456.815 | 1456.8144 | 0 | 0 | | | 70 | | 82 | | LGVPHPLQQNLNK | | | | |
|  | 1456.815 | 1456.8145 | 0 | 0 | | | 70 | | 82 | | LGVPHPLQQNLNK | | | | |
|  | 1456.815 | 1456.8148 | 0 | 0 | | | 70 | | 82 | | LGVPHPLQQNLNK | | | | |
|  | 1456.815 | 1456.8148 | 0 | 0 | | | 70 | | 82 | | LGVPHPLQQNLNK | | | | |
|  | 1456.815 | 1456.8151 | 0 | 0 | | | 70 | | 82 | | LGVPHPLQQNLNK | | | | |
|  | 1456.815 | 1456.8154 | 0 | 0 | | | 70 | | 82 | | LGVPHPLQQNLNK | | | | |
|  | 1699.9369 | 1699.9357 | 0 | -1 | | | 70 | | 84 | | LGVPHPLQQNLNKDK | | | | |
|  | 1699.9369 | 1699.9366 | 0 | 0 | | | 70 | | 84 | | LGVPHPLQQNLNKDK | | | | |
|  | 1699.9369 | 1699.9373 | 0 | 0 | | | 70 | | 84 | | LGVPHPLQQNLNKDK | | | | |

| Spot no. | Homologous protein | Accession no. | Score | M.P. | Cov. (%) | | Blast score | | Mr (kDa) / pI | | Ratio |
| --- | --- | --- | --- | --- | --- | --- | --- | --- | --- | --- | --- |
|  |  |  |  |  |  |  |  |  | Theo. | Exp. |  |
| 2c | glycinin G2 precursor | Glyma03g32020.1 | 103 | 2 | 4 | | 779 | | 55.1/5.46 |  |  |
|  | Calc. Mass | Observ. Mass | Da | ppm | start | | end | | sequence | | |
|  | 993.5243 | 993.5254 | 0 | 1 | 347 | 355 | | LSAQYGSLR | | | |
|  | 1449.6412 | 1449.6406 | 0 | 0 | 414 | 425 | | SQSDNFEYVSFK | | | |
|  | 1449.6412 | 1449.6406 | 0 | 0 | 414 | 425 | | SQSDNFEYVSFK | | | |
|  | 1449.6412 | 1449.6408 | 0 | 0 | 414 | 425 | | SQSDNFEYVSFK | | | |

| Spot no. | Homologous protein | | | | | | Accession no. | | | | | Score | | | | | | | M.P. | | | | | | Cov. (%) | | | | | | | | | | Blast score | | | | | | | | | | | | Mr (kDa) / pI | | | | | | | | | | | | | | | | | | | Ratio | | | | | | | | | |  |
| --- | --- | --- | --- | --- | --- | --- | --- | --- | --- | --- | --- | --- | --- | --- | --- | --- | --- | --- | --- | --- | --- | --- | --- | --- | --- | --- | --- | --- | --- | --- | --- | --- | --- | --- | --- | --- | --- | --- | --- | --- | --- | --- | --- | --- | --- | --- | --- | --- | --- | --- | --- | --- | --- | --- | --- | --- | --- | --- | --- | --- | --- | --- | --- | --- | --- | --- | --- | --- | --- | --- | --- | --- | --- | --- | --- | --- |
|  |  |  |  |  |  |  |  |  |  |  |  |  |  |  |  |  |  |  |  |  |  |  |  |  |  |  |  |  |  |  |  |  |  |  |  |  |  |  |  |  |  |  |  |  |  |  | Theo. | | | | | | | | | Exp. | | | | | | | | | |  | | | | | | | | | |  |
| 4a | seed biotinylated protein 68 kDa isoform | | | | | | Glyma13g36780.1 | | | | | 154 | | | | | | | 8 | | | | | | 18 | | | | | | | | | | 1033 | | | | | | | | | | | | 68.2/6.18 | | | | | | | | |  | | | | | | | | | |  | | | | | | | | | |  |
|  | Calc. Mass | | | | | | Observ. Mass | | | | | Da | | | | | | | ppm | | | | | | start | | | | | | | | | | end | | | | | | | | | | | | sequence | | | | | | | | | | | | | | | | | | | | | | | | | | | | |  |
|  | 1511.7191 | | | | | | 1511.7184 | | | | | 0 | | | | | | | 0 | | | | | | 28 | | | | | | | | | | 40 | | | | | | | | | | | | MATHFEHLAEQAK | | | | | | | | | | | | | | | | | | | | | | | | | | | | |  |
|  | 1511.7191 | | | | | | 1511.7187 | | | | | 0 | | | | | | | 0 | | | | | | 28 | | | | | | | | | | 40 | | | | | | | | | | | | MATHFEHLAEQAK | | | | | | | | | | | | | | | | | | | | | | | | | | | | |  |
|  | 1511.7191 | | | | | | 1511.7187 | | | | | 0 | | | | | | | 0 | | | | | | 28 | | | | | | | | | | 40 | | | | | | | | | | | | MATHFEHLAEQAK | | | | | | | | | | | | | | | | | | | | | | | | | | | | |  |
|  | 1511.7191 | | | | | | 1511.7187 | | | | | 0 | | | | | | | 0 | | | | | | 28 | | | | | | | | | | 40 | | | | | | | | | | | | MATHFEHLAEQAK | | | | | | | | | | | | | | | | | | | | | | | | | | | | |  |
|  | 1511.7191 | | | | | | 1511.7199 | | | | | 0 | | | | | | | 1 | | | | | | 28 | | | | | | | | | | 40 | | | | | | | | | | | | MATHFEHLAEQAK | | | | | | | | | | | | | | | | | | | | | | | | | | | | |  |
|  | 1511.7191 | | | | | | 1511.7199 | | | | | 0 | | | | | | | 1 | | | | | | 28 | | | | | | | | | | 40 | | | | | | | | | | | | MATHFEHLAEQAK | | | | | | | | | | | | | | | | | | | | | | | | | | | | |  |
|  | 1511.7191 | | | | | | 1511.7218 | | | | | 0 | | | | | | | 2 | | | | | | 28 | | | | | | | | | | 40 | | | | | | | | | | | | MATHFEHLAEQAK | | | | | | | | | | | | | | | | | | | | | | | | | | | | |  |
|  | 1511.7191 | | | | | | 1511.7228 | | | | | 0 | | | | | | | 2 | | | | | | 28 | | | | | | | | | | 40 | | | | | | | | | | | | MATHFEHLAEQAK | | | | | | | | | | | | | | | | | | | | | | | | | | | | |  |
|  | 1527.714 | | | | | | 1527.712 | | | | | 0 | | | | | | | -1 | | | | | | 28 | | | | | | | | | | 40 | | | | | | | | | | | | MATHFEHLAEQAK | | | | | | | | | | | | | | | | | | | | | | | | | | | | |  |
|  | 1527.714 | | | | | | 1527.7132 | | | | | 0 | | | | | | | -1 | | | | | | 28 | | | | | | | | | | 40 | | | | | | | | | | | | MATHFEHLAEQAK | | | | | | | | | | | | | | | | | | | | | | | | | | | | |  |
|  | 1527.714 | | | | | | 1527.7135 | | | | | 0 | | | | | | | 0 | | | | | | 28 | | | | | | | | | | 40 | | | | | | | | | | | | MATHFEHLAEQAK | | | | | | | | | | | | | | | | | | | | | | | | | | | | |  |
|  | 1527.714 | | | | | | 1527.7136 | | | | | 0 | | | | | | | 0 | | | | | | 28 | | | | | | | | | | 40 | | | | | | | | | | | | MATHFEHLAEQAK | | | | | | | | | | | | | | | | | | | | | | | | | | | | |  |
|  | 1527.714 | | | | | | 1527.718 | | | | | 0 | | | | | | | 3 | | | | | | 28 | | | | | | | | | | 40 | | | | | | | | | | | | MATHFEHLAEQAK | | | | | | | | | | | | | | | | | | | | | | | | | | | | |  |
|  | 1570.7587 | | | | | | 1570.7539 | | | | | 0 | | | | | | | -3 | | | | | | 49 | | | | | | | | | | 63 | | | | | | | | | | | | DTPQGSIEALQAGER | | | | | | | | | | | | | | | | | | | | | | | | | | | | |  |
|  | 1235.6431 | | | | | | 1235.6422 | | | | | 0 | | | | | | | -1 | | | | | | 190 | | | | | | | | | | 200 | | | | | | | | | | | | TTAVITCTLEK | | | | | | | | | | | | | | | | | | | | | | | | | | | | |  |
|  | 1723.8741 | | | | | | 1723.8732 | | | | | 0 | | | | | | | 0 | | | | | | 280 | | | | | | | | | | 296 | | | | | | | | | | | | GQQGYAVTKDTISSAAK | | | | | | | | | | | | | | | | | | | | | | | | | | | | |  |
|  | 1723.8741 | | | | | | 1723.8742 | | | | | 0 | | | | | | | 0 | | | | | | 280 | | | | | | | | | | 296 | | | | | | | | | | | | GQQGYAVTKDTISSAAK | | | | | | | | | | | | | | | | | | | | | | | | | | | | |  |
|  | 1507.7532 | | | | | | 1507.7527 | | | | | 0 | | | | | | | 0 | | | | | | 385 | | | | | | | | | | 400 | | | | | | | | | | | | AAAHVVEGAAGYAGHK | | | | | | | | | | | | | | | | | | | | | | | | | | | | |  |
|  | 1566.7889 | | | | | | 1566.7834 | | | | | 0 | | | | | | | -3 | | | | | | 414 | | | | | | | | | | 429 | | | | | | | | | | | | GLAASAGETAKEYTAK | | | | | | | | | | | | | | | | | | | | | | | | | | | | |  |
|  | 1566.7889 | | | | | | 1566.7858 | | | | | 0 | | | | | | | -2 | | | | | | 414 | | | | | | | | | | 429 | | | | | | | | | | | | GLAASAGETAKEYTAK | | | | | | | | | | | | | | | | | | | | | | | | | | | | |  |
|  | 2323.1404 | | | | | | 2323.1389 | | | | | 0 | | | | | | | -1 | | | | | | 442 | | | | | | | | | | 462 | | | | | | | | | | | | KPSQPQEAEERPSEGIGETVR | | | | | | | | | | | | | | | | | | | | | | | | | | | | |  |
|  | 2323.1404 | | | | | | 2323.1395 | | | | | 0 | | | | | | | 0 | | | | | | 442 | | | | | | | | | | 462 | | | | | | | | | | | | KPSQPQEAEERPSEGIGETVR | | | | | | | | | | | | | | | | | | | | | | | | | | | | |  |
|  | 2323.1404 | | | | | | 2323.1401 | | | | | 0 | | | | | | | 0 | | | | | | 442 | | | | | | | | | | 462 | | | | | | | | | | | | KPSQPQEAEERPSEGIGETVR | | | | | | | | | | | | | | | | | | | | | | | | | | | | |  |
|  | 2323.1404 | | | | | | 2323.1404 | | | | | 0 | | | | | | | 0 | | | | | | 442 | | | | | | | | | | 462 | | | | | | | | | | | | KPSQPQEAEERPSEGIGETVR | | | | | | | | | | | | | | | | | | | | | | | | | | | | |  |
|  | 1317.714 | | | | | | 1317.7153 | | | | | 0 | | | | | | | 1 | | | | | | 527 | | | | | | | | | | 539 | | | | | | | | | | | | SLTSIGEKLGDAK | | | | | | | | | | | | | | | | | | | | | | | | | | | | |  |
| Spot no. | Homologous protein | | | | | | Accession no. | | | | | Score | | | | | | | M.P. | | | | | | Cov. (%) | | | | | | | | Blast score | | | | | | | | | | | | | | | Mr (kDa) / pI | | | | | | | | | | | | | | | | | | | | | | | Ratio | | | | |  |
|  |  |  |  |  |  |  |  |  |  |  |  |  |  |  |  |  |  |  |  |  |  |  |  |  |  |  |  |  |  |  |  |  |  |  |  |  |  |  |  |  |  |  |  |  |  |  |  | Theo. | | | | | | | | Exp. | | | | | | | | | | | | | | |  |  |  |  |  |  |
| 4b | Os05g0553000 | | | | | | Glyma10g41330.1 | | | | | 146 | | | | | | | 3 | | | | | | 7 | | | | | | | | 879 | | | | | | | | | | | | | | | 60.1/5.8 | | | | | | | |  | | | | | | | | | | | | | | |  | | | | |  |
|  | Calc. Mass | | | | | | Observ. Mass | | | | | Da | | | | | | | ppm | | | | | | start | | | | | | | | end | | | | | | | | | | | | | | | sequence | | | | | | | | | | | | | | | | | | | | | | | | | | | |  |
|  | 1617.9202 | | | | | | 1617.9211 | | | | | 0 | | | | | | | 1 | | | | | | 122 | | | | | | | | 136 | | | | | | | | | | | | | | | LVLEVAQHLGEGVVR | | | | | | | | | | | | | | | | | | | | | | | | | | | |  |
|  | 1398.762 | | | | | | 1398.7612 | | | | | 0 | | | | | | | -1 | | | | | | 311 | | | | | | | | 323 | | | | | | | | | | | | | | | VGLTGLTVAEHFR | | | | | | | | | | | | | | | | | | | | | | | | | | | |  |
|  | 1491.7681 | | | | | | 1491.7688 | | | | | 0 | | | | | | | 0 | | | | | | 340 | | | | | | | | 353 | | | | | | | | | | | | | | | FTQANSEVSALLGR | | | | | | | | | | | | | | | | | | | | | | | | | | | |  |
| Spot no. | | Homologous protein | | | | | | Accession no. | | | | | Score | | | | | | M.P. | | | | | | | Cov. (%) | | | | | | | | | Blast score | | | | | | | | | | Mr (kDa) / pI | | | | | | | | | | | | | | | | | | | | | | | | | | Ratio | | | | |  |
|  |  |  |  |  |  |  |  |  |  |  |  |  |  |  |  |  |  |  |  |  |  |  |  |  |  |  |  |  |  |  |  |  |  |  |  |  |  |  |  |  |  |  |  |  | Theo. | | | | | | | | | | | Exp. | | | | | | | | | | | | | | |  |  |  |  |  |  |
| 4c | | lipoxygease L-4 | | | | | | Glyma13g42330.1 | | | | | 134 | | | | | | 8 | | | | | | | 13 | | | | | | | | | 1650 | | | | | | | | | | 96.8/5.76 | | | | | | | | | | |  | | | | | | | | | | | | | | |  | | | | |  |
|  | | Calc. Mass | | | | | | Observ. Mass | | | | | Da | | | | | | ppm | | | | | | start | | | | | | | | | end | | | | | | | | | | sequence | | | | | | | | | | | | | | | | | | | | | | | | | | | | | | | |  |
|  | | 1348.6122 | | | | | | 1348.6102 | | | | | 0 | | | | | | -1 | | | | | | 115 | | | | | | | | | 124 | | | | | | | | | | NFMQNEFYLK | | | | | | | | | | | | | | | | | | | | | | | | | | | | | | | |  |
|  | | 2061.8803 | | | | | | 2061.8814 | | | | | 0 | | | | | | 1 | | | | | | 197 | | | | | | | | | 214 | | | | | | | | | | IYDYDVYNDLGNPDSGDK | | | | | | | | | | | | | | | | | | | | | | | | | | | | | | | |  |
|  | | 1890.9476 | | | | | | 1890.9448 | | | | | 0 | | | | | | -1 | | | | | | 240 | | | | | | | | | 255 | | | | | | | | | | KDPNSEKPSDFVYLPR | | | | | | | | | | | | | | | | | | | | | | | | | | | | | | | |  |
|  | | 1637.8665 | | | | | | 1637.8634 | | | | | 0 | | | | | | -2 | | | | | | 305 | | | | | | | | | 319 | | | | | | | | | | LYEGGVTLPTNFLSK | | | | | | | | | | | | | | | | | | | | | | | | | | | | | | | |  |
|  | | 849.5688 | | | | | | 849.5688 | | | | | 0 | | | | | | 0 | | | | | | 320 | | | | | | | | | 327 | | | | | | | | | | IAPIPVIK | | | | | | | | | | | | | | | | | | | | | | | | | | | | | | | |  |
|  | | 1704.8141 | | | | | | 1704.814 | | | | | 0 | | | | | | 0 | | | | | | 384 | | | | | | | | | 398 | | | | | | | | | | LDTQAYGDHTCIIAK | | | | | | | | | | | | | | | | | | | | | | | | | | | | | | | |  |
|  | | 2089.0804 | | | | | | 2089.0753 | | | | | 0 | | | | | | -2 | | | | | | 399 | | | | | | | | | 417 | | | | | | | | | | EHLEPNLGGLTVEQAIQNK | | | | | | | | | | | | | | | | | | | | | | | | | | | | | | | |  |
|  | | 1141.6343 | | | | | | 1141.6338 | | | | | 0 | | | | | | 0 | | | | | | 559 | | | | | | | | | 569 | | | | | | | | | | ALVNADGIIEK | | | | | | | | | | | | | | | | | | | | | | | | | | | | | | | |  |
| Spot no. | Homologous protein | | | | | | Accession no. | | | | | Score | | | | | | | M.P. | | | | | | Cov. (%) | | | | | | | | | | | Blast score | | | | | | | | | Mr (kDa) / pI | | | | | | | | | | | | | | | | | | | | | | | Ratio | | | | | | | |  |
|  |  |  |  |  |  |  |  |  |  |  |  |  |  |  |  |  |  |  |  |  |  |  |  |  |  |  |  |  |  |  |  |  |  |  |  |  |  |  |  |  |  |  |  |  | Theo. | | | | | | | | | | Exp. | | | | | | | | | | | | |  |  |  |  |  |  |  |  |  |
| 6a | Unknown | | | | | | Glyma06g38160.1 | | | | | 321 | | | | | | | 10 | | | | | | 20 | | | | | | | | | | | 763 | | | | | | | | | 43.5/9.12 | | | | | | | | | |  | | | | | | | | | | | | |  | | | | | | | |  |
|  | Calc. Mass | | | | | | Observ. Mass | | | | | Da | | | | | | | ppm | | | | | | start | | | | | | | | | | | end | | | | | | | | | sequence | | | | | | | | | | | | | | | | | | | | | | | | | | | | | | |  |
|  | 1729.9938 | | | | | | 1729.9902 | | | | | 0 | | | | | | | -2 | | | | | | 86 | | | | | | | | | | | 104 | | | | | | | | | KGSVIITGASSGLGLATAK | | | | | | | | | | | | | | | | | | | | | | | | | | | | | | |  |
|  | 1729.9938 | | | | | | 1729.9918 | | | | | 0 | | | | | | | -1 | | | | | | 86 | | | | | | | | | | | 104 | | | | | | | | | KGSVIITGASSGLGLATAK | | | | | | | | | | | | | | | | | | | | | | | | | | | | | | |  |
|  | 1729.9938 | | | | | | 1729.9918 | | | | | 0 | | | | | | | -1 | | | | | | 86 | | | | | | | | | | | 104 | | | | | | | | | KGSVIITGASSGLGLATAK | | | | | | | | | | | | | | | | | | | | | | | | | | | | | | |  |
|  | 1601.8988 | | | | | | 1601.8978 | | | | | 0 | | | | | | | -1 | | | | | | 87 | | | | | | | | | | | 104 | | | | | | | | | GSVIITGASSGLGLATAK | | | | | | | | | | | | | | | | | | | | | | | | | | | | | | |  |
|  | 942.5386 | | | | | | 942.5346 | | | | | 0 | | | | | | | -4 | | | | | | 206 | | | | | | | | | | | 213 | | | | | | | | | LLLDDLNK | | | | | | | | | | | | | | | | | | | | | | | | | | | | | | |  |
|  | 942.5386 | | | | | | 942.538 | | | | | 0 | | | | | | | -1 | | | | | | 206 | | | | | | | | | | | 213 | | | | | | | | | LLLDDLNK | | | | | | | | | | | | | | | | | | | | | | | | | | | | | | |  |
|  | 942.5386 | | | | | | 942.538 | | | | | 0 | | | | | | | -1 | | | | | | 206 | | | | | | | | | | | 213 | | | | | | | | | LLLDDLNK | | | | | | | | | | | | | | | | | | | | | | | | | | | | | | |  |
|  | 942.5386 | | | | | | 942.5388 | | | | | 0 | | | | | | | 0 | | | | | | 206 | | | | | | | | | | | 213 | | | | | | | | | LLLDDLNK | | | | | | | | | | | | | | | | | | | | | | | | | | | | | | |  |
|  | 1619.8406 | | | | | | 1619.8406 | | | | | 0 | | | | | | | 0 | | | | | | 206 | | | | | | | | | | | 219 | | | | | | | | | LLLDDLNKSDYPSK | | | | | | | | | | | | | | | | | | | | | | | | | | | | | | |  |
|  | 1564.6949 | | | | | | 1564.6936 | | | | | 0 | | | | | | | -1 | | | | | | 279 | | | | | | | | | | | 290 | | | | | | | | | VCNMLTMQEFHR | | | | | | | | | | | | | | | | | | | | | | | | | | | | | | |  |
|  | 1580.6898 | | | | | | 1580.684 | | | | | 0 | | | | | | | -4 | | | | | | 279 | | | | | | | | | | | 290 | | | | | | | | | VCNMLTMQEFHR | | | | | | | | | | | | | | | | | | | | | | | | | | | | | | |  |
|  | 1580.6898 | | | | | | 1580.6872 | | | | | 0 | | | | | | | -2 | | | | | | 279 | | | | | | | | | | | 290 | | | | | | | | | VCNMLTMQEFHR | | | | | | | | | | | | | | | | | | | | | | | | | | | | | | |  |
|  | 1580.6898 | | | | | | 1580.6932 | | | | | 0 | | | | | | | 2 | | | | | | 279 | | | | | | | | | | | 290 | | | | | | | | | VCNMLTMQEFHR | | | | | | | | | | | | | | | | | | | | | | | | | | | | | | |  |
|  | 1596.6847 | | | | | | 1596.6829 | | | | | 0 | | | | | | | -1 | | | | | | 279 | | | | | | | | | | | 290 | | | | | | | | | VCNMLTMQEFHR | | | | | | | | | | | | | | | | | | | | | | | | | | | | | | |  |
|  | 1596.6847 | | | | | | 1596.6838 | | | | | 0 | | | | | | | -1 | | | | | | 279 | | | | | | | | | | | 290 | | | | | | | | | VCNMLTMQEFHR | | | | | | | | | | | | | | | | | | | | | | | | | | | | | | |  |
|  | 1736.7909 | | | | | | 1736.7898 | | | | | 0 | | | | | | | -1 | | | | | | 279 | | | | | | | | | | | 291 | | | | | | | | | VCNMLTMQEFHRR | | | | | | | | | | | | | | | | | | | | | | | | | | | | | | |  |
|  | 910.5025 | | | | | | 910.5026 | | | | | 0 | | | | | | | 0 | | | | | | 316 | | | | | | | | | | | 322 | | | | | | | | | EHIPLFR | | | | | | | | | | | | | | | | | | | | | | | | | | | | | | |  |
|  | 910.5025 | | | | | | 910.5026 | | | | | 0 | | | | | | | 0 | | | | | | 316 | | | | | | | | | | | 322 | | | | | | | | | EHIPLFR | | | | | | | | | | | | | | | | | | | | | | | | | | | | | | |  |
|  | 910.5025 | | | | | | 910.5032 | | | | | 0 | | | | | | | 1 | | | | | | 316 | | | | | | | | | | | 322 | | | | | | | | | EHIPLFR | | | | | | | | | | | | | | | | | | | | | | | | | | | | | | |  |
|  | 988.5746 | | | | | | 988.5736 | | | | | 0 | | | | | | | -1 | | | | | | 323 | | | | | | | | | | | 330 | | | | | | | | | LLFPPFQK | | | | | | | | | | | | | | | | | | | | | | | | | | | | | | |  |
|  | 988.5746 | | | | | | 988.574 | | | | | 0 | | | | | | | -1 | | | | | | 323 | | | | | | | | | | | 330 | | | | | | | | | LLFPPFQK | | | | | | | | | | | | | | | | | | | | | | | | | | | | | | |  |
|  | 988.5746 | | | | | | 988.5747 | | | | | 0 | | | | | | | 0 | | | | | | 323 | | | | | | | | | | | 330 | | | | | | | | | LLFPPFQK | | | | | | | | | | | | | | | | | | | | | | | | | | | | | | |  |
|  | 1256.6976 | | | | | | 1256.6966 | | | | | 0 | | | | | | | -1 | | | | | | 346 | | | | | | | | | | | 357 | | | | | | | | | LAQVVSDPSLTK | | | | | | | | | | | | | | | | | | | | | | | | | | | | | | |  |
|  | 1256.6976 | | | | | | 1256.6968 | | | | | 0 | | | | | | | -1 | | | | | | 346 | | | | | | | | | | | 357 | | | | | | | | | LAQVVSDPSLTK | | | | | | | | | | | | | | | | | | | | | | | | | | | | | | |  |
|  | 1256.6976 | | | | | | 1256.6968 | | | | | 0 | | | | | | | -1 | | | | | | 346 | | | | | | | | | | | 357 | | | | | | | | | LAQVVSDPSLTK | | | | | | | | | | | | | | | | | | | | | | | | | | | | | | |  |
|  | 1256.6976 | | | | | | 1256.697 | | | | | 0 | | | | | | | 0 | | | | | | 346 | | | | | | | | | | | 357 | | | | | | | | | LAQVVSDPSLTK | | | | | | | | | | | | | | | | | | | | | | | | | | | | | | |  |
|  | 1017.5495 | | | | | | 1017.5484 | | | | | 0 | | | | | | | -1 | | | | | | 387 | | | | | | | | | | | 394 | | | | | | | | | KVWEISEK | | | | | | | | | | | | | | | | | | | | | | | | | | | | | | |  |
| Spot no. | | | Homologous protein | | | | | | Accession no. | | | | | | Score | | | | | M.P. | | | | | | | Cov. (%) | | | | | | | | | | Blast score | | | | | | | | | Mr (kDa) / pI | | | | | | | | | | | | | | | | | | | Ratio | | | | | | | | | | |  |
|  |  |  |  |  |  |  |  |  |  |  |  |  |  |  |  |  |  |  |  |  |  |  |  |  |  |  |  |  |  |  |  |  |  |  |  |  |  |  |  |  |  |  |  |  |  | Theo. | | | | | | | | | | | Exp. | | | | | | | |  |  |  |  |  |  |  |  |  |  |  |  |
| 6b | | | Os05g0553000 | | | | | | Glyma10g41330.1 | | | | | | 136 | | | | | 3 | | | | | | | 7 | | | | | | | | | | 879 | | | | | | | | | 60.1/5.8 | | | | | | | | | | |  | | | | | | | |  | | | | | | | | | | |  |
|  | | | Calc. Mass | | | | | | Observ. Mass | | | | | | Da | | | | | ppm | | | | | | | start | | | | | | | | | | end | | | | | | | | | sequence | | | | | | | | | | | | | | | | | | | | | | | | | | | | | |  |
|  | | | 1408.8038 | | | | | | 1408.8032 | | | | | | 0 | | | | | 0 | | | | | | | 152 | | | | | | | | | | 165 | | | | | | | | | VLNTGSPITVPVGR | | | | | | | | | | | | | | | | | | | | | | | | | | | | | |  |
|  | | | 1398.762 | | | | | | 1398.7618 | | | | | | 0 | | | | | 0 | | | | | | | 311 | | | | | | | | | | 323 | | | | | | | | | VGLTGLTVAEHFR | | | | | | | | | | | | | | | | | | | | | | | | | | | | | |  |
|  | | | 1491.7681 | | | | | | 1491.7664 | | | | | | 0 | | | | | -1 | | | | | | | 340 | | | | | | | | | | 353 | | | | | | | | | FTQANSEVSALLGR | | | | | | | | | | | | | | | | | | | | | | | | | | | | | |  |
| Spot no. | | | Homologous protein | | | | | | Accession no. | | | | | Score | | | | | | M.P. | | | | | | | Cov. (%) | | | | | | | | | Blast score | | | | | | | | | Mr (kDa) / pI | | | | | | | | | | | | | | | | | | | | | | Ratio | | | | | | | | |  |
|  |  |  |  |  |  |  |  |  |  |  |  |  |  |  |  |  |  |  |  |  |  |  |  |  |  |  |  |  |  |  |  |  |  |  |  |  |  |  |  |  |  |  |  |  | Theo. | | | | | | | | | | | | | Exp. | | | | | | | | |  |  |  |  |  |  |  |  |  |  |
| 7 | | | peroxisomal fatty acid beta-oxidation multifunctional protein | | | | | | Glyma07g37570.1 | | | | | 267 | | | | | | 13 | | | | | | | 13 | | | | | | | | | 1364 | | | | | | | | | 84.2/9.01 | | | | | | | | | | | | |  | | | | | | | | |  | | | | | | | | |  |
|  | | | Calc. Mass | | | | | | Observ. Mass | | | | | Da | | | | | | ppm | | | | | | | start | | | | | | | | | end | | | | | | | | | sequence | | | | | | | | | | | | | | | | | | | | | | | | | | | | | | |  |
|  | | | 1613.8196 | | | | | | 1613.8186 | | | | | 0 | | | | | | -1 | | | | | | | 334 | | | | | | | | | 349 | | | | | | | | | GTSKVPGVTDCGLAPR | | | | | | | | | | | | | | | | | | | | | | | | | | | | | | |  |
|  | | | 1613.8196 | | | | | | 1613.8228 | | | | | 0 | | | | | | 2 | | | | | | | 334 | | | | | | | | | 349 | | | | | | | | | GTSKVPGVTDCGLAPR | | | | | | | | | | | | | | | | | | | | | | | | | | | | | | |  |
|  | | | 1240.6234 | | | | | | 1240.6222 | | | | | 0 | | | | | | -1 | | | | | | | 338 | | | | | | | | | | 349 | | | | | | | | | VPGVTDCGLAPR | | | | | | | | | | | | | | | | | | | | | | | | | | | | | |  |
|  | | | 1240.6234 | | | | | | 1240.6224 | | | | | 0 | | | | | | -1 | | | | | | | 338 | | | | | | | | | | 349 | | | | | | | | | VPGVTDCGLAPR | | | | | | | | | | | | | | | | | | | | | | | | | | | | | |  |
|  | | | 1240.6234 | | | | | | 1240.6226 | | | | | 0 | | | | | | -1 | | | | | | | 338 | | | | | | | | | | 349 | | | | | | | | | VPGVTDCGLAPR | | | | | | | | | | | | | | | | | | | | | | | | | | | | | |  |
|  | | | 1240.6234 | | | | | | 1240.6238 | | | | | 0 | | | | | | 0 | | | | | | | 338 | | | | | | | | | | 349 | | | | | | | | | VPGVTDCGLAPR | | | | | | | | | | | | | | | | | | | | | | | | | | | | | |  |
|  | | | 1717.8934 | | | | | | 1717.8907 | | | | | 0 | | | | | | -2 | | | | | | | 524 | | | | | | | | | | 539 | | | | | | | | | KTPVVVGNCTGFAVNR | | | | | | | | | | | | | | | | | | | | | | | | | | | | | |  |
|  | | | 1589.7984 | | | | | | 1589.797 | | | | | 0 | | | | | | -1 | | | | | | | 525 | | | | | | | | | | 539 | | | | | | | | | TPVVVGNCTGFAVNR | | | | | | | | | | | | | | | | | | | | | | | | | | | | | |  |
|  | | | 1589.7984 | | | | | | 1589.7974 | | | | | 0 | | | | | | -1 | | | | | | | 525 | | | | | | | | | | 539 | | | | | | | | | TPVVVGNCTGFAVNR | | | | | | | | | | | | | | | | | | | | | | | | | | | | | |  |
|  | | | 1443.7391 | | | | | | 1443.7358 | | | | | 0 | | | | | | -2 | | | | | | | 604 | | | | | | | | | | 615 | | | | | | | | | SMLIPLLQEDNR | | | | | | | | | | | | | | | | | | | | | | | | | | | | | |  |
|  | | | 1147.555 | | | | | | 1147.5542 | | | | | 0 | | | | | | -1 | | | | | | | 622 | | | | | | | | | | 630 | | | | | | | | | KGFYLYDDK | | | | | | | | | | | | | | | | | | | | | | | | | | | | | |  |
|  | | | 1019.46 | | | | | | 1019.4612 | | | | | 0 | | | | | | 1 | | | | | | | 623 | | | | | | | | | | 630 | | | | | | | | | GFYLYDDK | | | | | | | | | | | | | | | | | | | | | | | | | | | | | |  |
|  | | | 1175.5611 | | | | | | 1175.5566 | | | | | 0 | | | | | | -4 | | | | | | | 623 | | | | | | | | | | 631 | | | | | | | | | GFYLYDDKR | | | | | | | | | | | | | | | | | | | | | | | | | | | | | |  |
|  | | | 1340.7299 | | | | | | 1340.7298 | | | | | 0 | | | | | | 0 | | | | | | | 750 | | | | | | | | | | 762 | | | | | | | | | GIPLSASLEQQAK | | | | | | | | | | | | | | | | | | | | | | | | | | | | | |  |
|  | | | 1340.7299 | | | | | | 1340.7322 | | | | | 0 | | | | | | 2 | | | | | | | 750 | | | | | | | | | | 762 | | | | | | | | | GIPLSASLEQQAK | | | | | | | | | | | | | | | | | | | | | | | | | | | | | |  |
| Spot no. | | | Homologous protein | | | | | | Accession no. | | | | | | Score | | | | | | | | M.P. | | | | | | | Cov. (%) | | | | | | | | | | | | | Blast score | | | | | | | | | | | Mr (kDa) / pI | | | | | | | | | | | | | | | | | | | Ratio | | |  |
|  |  |  |  |  |  |  |  |  |  |  |  |  |  |  |  |  |  |  |  |  |  |  |  |  |  |  |  |  |  |  |  |  |  |  |  |  |  |  |  |  |  |  |  |  |  |  |  |  |  |  |  |  |  | Theo. | | | | | Exp. | | | | | | | | | | | | | |  |  |  |  |
| 8 | | | beta-conglycinin alpha prime subunit | | | | | | Glyma10g39150.1 | | | | | | 1274 | | | | | | | | 15 | | | | | | | 25 | | | | | | | | | | | | | 893 | | | | | | | | | | | 72.7/5.5 | | | | |  | | | | | | | | | | | | | |  | | |  |
|  | | | Calc. Mass | | | | | | Observ. Mass | | | | | | Da | | | | | | | | ppm | | | | | | | start | | | | | | | | | | | | | end | | | | | | | | | | | sequence | | | | | | | | | | | | | | | | | | | | | |  |
|  | | | 1231.6098 | | | | | | 1231.6094 | | | | | | 0 | | | | | | | | 0 | | | | | | | 208 | | | | | | | | | | | | | 217 | | | | | | | | | | | NKNPFHFNSK | | | | | | | | | | | | | | | | | | | | | |  |
|  | | | 1231.6098 | | | | | | 1231.6096 | | | | | | 0 | | | | | | | | 0 | | | | | | | 208 | | | | | | | | | | | | | 217 | | | | | | | | | | | NKNPFHFNSK | | | | | | | | | | | | | | | | | | | | | |  |
|  | | | 1231.6098 | | | | | | 1231.6099 | | | | | | 0 | | | | | | | | 0 | | | | | | | 208 | | | | | | | | | | | | | 217 | | | | | | | | | | | NKNPFHFNSK | | | | | | | | | | | | | | | | | | | | | |  |
|  | | | 1231.6098 | | | | | | 1231.6099 | | | | | | 0 | | | | | | | | 0 | | | | | | | 208 | | | | | | | | | | | | | 217 | | | | | | | | | | | NKNPFHFNSK | | | | | | | | | | | | | | | | | | | | | |  |
|  | | | 1231.6098 | | | | | | 1231.6099 | | | | | | 0 | | | | | | | | 0 | | | | | | | 208 | | | | | | | | | | | | | 217 | | | | | | | | | | | NKNPFHFNSK | | | | | | | | | | | | | | | | | | | | | |  |
|  | | | 1231.6098 | | | | | | 1231.6105 | | | | | | 0 | | | | | | | | 1 | | | | | | | 208 | | | | | | | | | | | | | 217 | | | | | | | | | | | NKNPFHFNSK | | | | | | | | | | | | | | | | | | | | | |  |
|  | | | 1231.6098 | | | | | | 1231.6111 | | | | | | 0 | | | | | | | | 1 | | | | | | | 208 | | | | | | | | | | | | | 217 | | | | | | | | | | | NKNPFHFNSK | | | | | | | | | | | | | | | | | | | | | |  |
|  | | | 782.4327 | | | | | | 782.4317 | | | | | | 0 | | | | | | | | -1 | | | | | | | 219 | | | | | | | | | | | | | 224 | | | | | | | | | | | FQTLFK | | | | | | | | | | | | | | | | | | | | | |  |
|  | | | 1337.6211 | | | | | | 1337.6206 | | | | | | 0 | | | | | | | | 0 | | | | | | | 291 | | | | | | | | | | | | | 302 | | | | | | | | | | | DSYNLQSGDALR | | | | | | | | | | | | | | | | | | | | | |  |
|  | | | 1337.6211 | | | | | | 1337.6206 | | | | | | 0 | | | | | | | | 0 | | | | | | | 291 | | | | | | | | | | | | | 302 | | | | | | | | | | | DSYNLQSGDALR | | | | | | | | | | | | | | | | | | | | | |  |
|  | | | 1337.6211 | | | | | | 1337.6208 | | | | | | 0 | | | | | | | | 0 | | | | | | | 291 | | | | | | | | | | | | | 302 | | | | | | | | | | | DSYNLQSGDALR | | | | | | | | | | | | | | | | | | | | | |  |
|  | | | 1337.6211 | | | | | | 1337.6212 | | | | | | 0 | | | | | | | | 0 | | | | | | | 291 | | | | | | | | | | | | | 302 | | | | | | | | | | | DSYNLQSGDALR | | | | | | | | | | | | | | | | | | | | | |  |
|  | | | 1337.6211 | | | | | | 1337.6218 | | | | | | 0 | | | | | | | | 1 | | | | | | | 291 | | | | | | | | | | | | | 302 | | | | | | | | | | | DSYNLQSGDALR | | | | | | | | | | | | | | | | | | | | | |  |
|  | | | 2136.0124 | | | | | | 2136.0094 | | | | | | 0 | | | | | | | | -1 | | | | | | | 303 | | | | | | | | | | | | | 321 | | | | | | | | | | | VPAGTTYYVVNPDNDENLR | | | | | | | | | | | | | | | | | | | | | |  |
|  | | | 2136.0124 | | | | | | 2136.0094 | | | | | | 0 | | | | | | | | -1 | | | | | | | 303 | | | | | | | | | | | | | 321 | | | | | | | | | | | VPAGTTYYVVNPDNDENLR | | | | | | | | | | | | | | | | | | | | | |  |
|  | | | 2136.0124 | | | | | | 2136.0094 | | | | | | 0 | | | | | | | | -1 | | | | | | | 303 | | | | | | | | | | | | | 321 | | | | | | | | | | | VPAGTTYYVVNPDNDENLR | | | | | | | | | | | | | | | | | | | | | |  |
|  | | | 2136.0124 | | | | | | 2136.0094 | | | | | | 0 | | | | | | | | -1 | | | | | | | 303 | | | | | | | | | | | | | 321 | | | | | | | | | | | VPAGTTYYVVNPDNDENLR | | | | | | | | | | | | | | | | | | | | | |  |
|  | | | 2136.0124 | | | | | | 2136.0094 | | | | | | 0 | | | | | | | | -1 | | | | | | | 303 | | | | | | | | | | | | | 321 | | | | | | | | | | | VPAGTTYYVVNPDNDENLR | | | | | | | | | | | | | | | | | | | | | |  |
|  | | | 2136.0124 | | | | | | 2136.01 | | | | | | 0 | | | | | | | | -1 | | | | | | | 303 | | | | | | | | | | | | | 321 | | | | | | | | | | | VPAGTTYYVVNPDNDENLR | | | | | | | | | | | | | | | | | | | | | |  |
|  | | | 2136.0124 | | | | | | 2136.01 | | | | | | 0 | | | | | | | | -1 | | | | | | | 303 | | | | | | | | | | | | | 321 | | | | | | | | | | | VPAGTTYYVVNPDNDENLR | | | | | | | | | | | | | | | | | | | | | |  |
|  | | | 2136.0124 | | | | | | 2136.0103 | | | | | | 0 | | | | | | | | -1 | | | | | | | 303 | | | | | | | | | | | | | 321 | | | | | | | | | | | VPAGTTYYVVNPDNDENLR | | | | | | | | | | | | | | | | | | | | | |  |
|  | | | 2136.0124 | | | | | | 2136.0114 | | | | | | 0 | | | | | | | | 0 | | | | | | | 303 | | | | | | | | | | | | | 321 | | | | | | | | | | | VPAGTTYYVVNPDNDENLR | | | | | | | | | | | | | | | | | | | | | |  |
|  | | | 2136.0124 | | | | | | 2136.0114 | | | | | | 0 | | | | | | | | 0 | | | | | | | 303 | | | | | | | | | | | | | 321 | | | | | | | | | | | VPAGTTYYVVNPDNDENLR | | | | | | | | | | | | | | | | | | | | | |  |
|  | | | 2136.0124 | | | | | | 2136.0134 | | | | | | 0 | | | | | | | | 1 | | | | | | | 303 | | | | | | | | | | | | | 321 | | | | | | | | | | | VPAGTTYYVVNPDNDENLR | | | | | | | | | | | | | | | | | | | | | |  |
|  | | | 1408.8224 | | | | | | 1408.8211 | | | | | | 0 | | | | | | | | -1 | | | | | | | 322 | | | | | | | | | | | | | 334 | | | | | | | | | | | MITLAIPVNKPGR | | | | | | | | | | | | | | | | | | | | | |  |
|  | | | 1408.8224 | | | | | | 1408.8212 | | | | | | 0 | | | | | | | | -1 | | | | | | | 322 | | | | | | | | | | | | | 334 | | | | | | | | | | | MITLAIPVNKPGR | | | | | | | | | | | | | | | | | | | | | |  |
|  | | | 1408.8224 | | | | | | 1408.8212 | | | | | | 0 | | | | | | | | -1 | | | | | | | 322 | | | | | | | | | | | | | 334 | | | | | | | | | | | MITLAIPVNKPGR | | | | | | | | | | | | | | | | | | | | | |  |
|  | | | 1408.8224 | | | | | | 1408.8217 | | | | | | 0 | | | | | | | | -1 | | | | | | | 322 | | | | | | | | | | | | | 334 | | | | | | | | | | | MITLAIPVNKPGR | | | | | | | | | | | | | | | | | | | | | |  |
|  | | | 1408.8224 | | | | | | 1408.822 | | | | | | 0 | | | | | | | | 0 | | | | | | | 322 | | | | | | | | | | | | | 334 | | | | | | | | | | | MITLAIPVNKPGR | | | | | | | | | | | | | | | | | | | | | |  |
|  | | | 1408.8224 | | | | | | 1408.8258 | | | | | | 0 | | | | | | | | 2 | | | | | | | 322 | | | | | | | | | | | | | 334 | | | | | | | | | | | MITLAIPVNKPGR | | | | | | | | | | | | | | | | | | | | | |  |
|  | | | 1424.8173 | | | | | | 1424.8162 | | | | | | 0 | | | | | | | | -1 | | | | | | | 322 | | | | | | | | | | | | | 334 | | | | | | | | | | | MITLAIPVNKPGR | | | | | | | | | | | | | | | | | | | | | |  |
|  | | | 1424.8173 | | | | | | 1424.8165 | | | | | | 0 | | | | | | | | -1 | | | | | | | 322 | | | | | | | | | | | | | 334 | | | | | | | | | | | MITLAIPVNKPGR | | | | | | | | | | | | | | | | | | | | | |  |
|  | | | 1424.8173 | | | | | | 1424.8171 | | | | | | 0 | | | | | | | | 0 | | | | | | | 322 | | | | | | | | | | | | | 334 | | | | | | | | | | | MITLAIPVNKPGR | | | | | | | | | | | | | | | | | | | | | |  |
|  | | | 1424.8173 | | | | | | 1424.8174 | | | | | | 0 | | | | | | | | 0 | | | | | | | 322 | | | | | | | | | | | | | 334 | | | | | | | | | | | MITLAIPVNKPGR | | | | | | | | | | | | | | | | | | | | | |  |
|  | | | 1152.5662 | | | | | | 1152.5637 | | | | | | 0 | | | | | | | | -2 | | | | | | | 356 | | | | | | | | | | | | | 365 | | | | | | | | | | | NILEASYDTK | | | | | | | | | | | | | | | | | | | | | |  |
|  | | | 1152.5662 | | | | | | 1152.5654 | | | | | | 0 | | | | | | | | -1 | | | | | | | 356 | | | | | | | | | | | | | 365 | | | | | | | | | | | NILEASYDTK | | | | | | | | | | | | | | | | | | | | | |  |
|  | | | 1152.5662 | | | | | | 1152.5656 | | | | | | 0 | | | | | | | | -1 | | | | | | | 356 | | | | | | | | | | | | | 365 | | | | | | | | | | | NILEASYDTK | | | | | | | | | | | | | | | | | | | | | |  |
|  | | | 1760.8442 | | | | | | 1760.8444 | | | | | | 0 | | | | | | | | 0 | | | | | | | 372 | | | | | | | | | | | | | 386 | | | | | | | | | | | VLFGREEGQQQGEER | | | | | | | | | | | | | | | | | | | | | |  |
|  | | | 1760.8442 | | | | | | 1760.8459 | | | | | | 0 | | | | | | | | 1 | | | | | | | 372 | | | | | | | | | | | | | 386 | | | | | | | | | | | VLFGREEGQQQGEER | | | | | | | | | | | | | | | | | | | | | |  |
|  | | | 1371.7973 | | | | | | 1371.797 | | | | | | 0 | | | | | | | | 0 | | | | | | | 387 | | | | | | | | | | | | | 398 | | | | | | | | | | | LQESVIVEISKK | | | | | | | | | | | | | | | | | | | | | |  |
|  | | | 1371.7973 | | | | | | 1371.7978 | | | | | | 0 | | | | | | | | 0 | | | | | | | 387 | | | | | | | | | | | | | 398 | | | | | | | | | | | LQESVIVEISKK | | | | | | | | | | | | | | | | | | | | | |  |
|  | | | 1371.7973 | | | | | | 1371.7999 | | | | | | 0 | | | | | | | | 2 | | | | | | | 387 | | | | | | | | | | | | | 398 | | | | | | | | | | | LQESVIVEISKK | | | | | | | | | | | | | | | | | | | | | |  |
|  | | | 1533.8151 | | | | | | 1533.8126 | | | | | | 0 | | | | | | | | -2 | | | | | | | 413 | | | | | | | | | | | | | 425 | | | | | | | | | | | KTISSEDKPFNLR | | | | | | | | | | | | | | | | | | | | | |  |
|  | | | 1533.8151 | | | | | | 1533.8146 | | | | | | 0 | | | | | | | | 0 | | | | | | | 413 | | | | | | | | | | | | | 425 | | | | | | | | | | | KTISSEDKPFNLR | | | | | | | | | | | | | | | | | | | | | |  |
|  | | | 1533.8151 | | | | | | 1533.8148 | | | | | | 0 | | | | | | | | 0 | | | | | | | 413 | | | | | | | | | | | | | 425 | | | | | | | | | | | KTISSEDKPFNLR | | | | | | | | | | | | | | | | | | | | | |  |
|  | | | 1533.8151 | | | | | | 1533.8149 | | | | | | 0 | | | | | | | | 0 | | | | | | | 413 | | | | | | | | | | | | | 425 | | | | | | | | | | | KTISSEDKPFNLR | | | | | | | | | | | | | | | | | | | | | |  |
|  | | | 1533.8151 | | | | | | 1533.8149 | | | | | | 0 | | | | | | | | 0 | | | | | | | 413 | | | | | | | | | | | | | 425 | | | | | | | | | | | KTISSEDKPFNLR | | | | | | | | | | | | | | | | | | | | | |  |
|  | | | 1533.8151 | | | | | | 1533.8158 | | | | | | 0 | | | | | | | | 0 | | | | | | | 413 | | | | | | | | | | | | | 425 | | | | | | | | | | | KTISSEDKPFNLR | | | | | | | | | | | | | | | | | | | | | |  |
|  | | | 1533.8151 | | | | | | 1533.8158 | | | | | | 0 | | | | | | | | 0 | | | | | | | 413 | | | | | | | | | | | | | 425 | | | | | | | | | | | KTISSEDKPFNLR | | | | | | | | | | | | | | | | | | | | | |  |
|  | | | 1533.8151 | | | | | | 1533.8164 | | | | | | 0 | | | | | | | | 1 | | | | | | | 413 | | | | | | | | | | | | | 425 | | | | | | | | | | | KTISSEDKPFNLR | | | | | | | | | | | | | | | | | | | | | |  |
|  | | | 1405.7201 | | | | | | 1405.7182 | | | | | | 0 | | | | | | | | -1 | | | | | | | 414 | | | | | | | | | | | | | 425 | | | | | | | | | | | TISSEDKPFNLR | | | | | | | | | | | | | | | | | | | | | |  |
|  | | | 1405.7201 | | | | | | 1405.7186 | | | | | | 0 | | | | | | | | -1 | | | | | | | 414 | | | | | | | | | | | | | 425 | | | | | | | | | | | TISSEDKPFNLR | | | | | | | | | | | | | | | | | | | | | |  |
|  | | | 1405.7201 | | | | | | 1405.7186 | | | | | | 0 | | | | | | | | -1 | | | | | | | 414 | | | | | | | | | | | | | 425 | | | | | | | | | | | TISSEDKPFNLR | | | | | | | | | | | | | | | | | | | | | |  |
|  | | | 1405.7201 | | | | | | 1405.7186 | | | | | | 0 | | | | | | | | -1 | | | | | | | 414 | | | | | | | | | | | | | 425 | | | | | | | | | | | TISSEDKPFNLR | | | | | | | | | | | | | | | | | | | | | |  |
|  | | | 1405.7201 | | | | | | 1405.7192 | | | | | | 0 | | | | | | | | -1 | | | | | | | 414 | | | | | | | | | | | | | 425 | | | | | | | | | | | TISSEDKPFNLR | | | | | | | | | | | | | | | | | | | | | |  |
|  | | | 1405.7201 | | | | | | 1405.7197 | | | | | | 0 | | | | | | | | 0 | | | | | | | 414 | | | | | | | | | | | | | 425 | | | | | | | | | | | TISSEDKPFNLR | | | | | | | | | | | | | | | | | | | | | |  |
|  | | | 1405.7201 | | | | | | 1405.7203 | | | | | | 0 | | | | | | | | 0 | | | | | | | 414 | | | | | | | | | | | | | 425 | | | | | | | | | | | TISSEDKPFNLR | | | | | | | | | | | | | | | | | | | | | |  |
|  | | | 1405.7201 | | | | | | 1405.7228 | | | | | | 0 | | | | | | | | 2 | | | | | | | 414 | | | | | | | | | | | | | 425 | | | | | | | | | | | TISSEDKPFNLR | | | | | | | | | | | | | | | | | | | | | |  |
|  | | | 975.5277 | | | | | | 975.5276 | | | | | | 0 | | | | | | | | 0 | | | | | | | 438 | | | | | | | | | | | | | 445 | | | | | | | | | | | LFEITPEK | | | | | | | | | | | | | | | | | | | | | |  |
|  | | | 975.5277 | | | | | | 975.5276 | | | | | | 0 | | | | | | | | 0 | | | | | | | 438 | | | | | | | | | | | | | 445 | | | | | | | | | | | LFEITPEK | | | | | | | | | | | | | | | | | | | | | |  |
|  | | | 975.5277 | | | | | | 975.5276 | | | | | | 0 | | | | | | | | 0 | | | | | | | 438 | | | | | | | | | | | | | 445 | | | | | | | | | | | LFEITPEK | | | | | | | | | | | | | | | | | | | | | |  |
|  | | | 1583.8671 | | | | | | 1583.8642 | | | | | | 0 | | | | | | | | -2 | | | | | | | 438 | | | | | | | | | | | | | 450 | | | | | | | | | | | LFEITPEKNPQLR | | | | | | | | | | | | | | | | | | | | | |  |
|  | | | 1583.8671 | | | | | | 1583.866 | | | | | | 0 | | | | | | | | -1 | | | | | | | 438 | | | | | | | | | | | | | 450 | | | | | | | | | | | LFEITPEKNPQLR | | | | | | | | | | | | | | | | | | | | | |  |
|  | | | 1583.8671 | | | | | | 1583.8666 | | | | | | 0 | | | | | | | | 0 | | | | | | | 438 | | | | | | | | | | | | | 450 | | | | | | | | | | | LFEITPEKNPQLR | | | | | | | | | | | | | | | | | | | | | |  |
|  | | | 1583.8671 | | | | | | 1583.8666 | | | | | | 0 | | | | | | | | 0 | | | | | | | 438 | | | | | | | | | | | | | 450 | | | | | | | | | | | LFEITPEKNPQLR | | | | | | | | | | | | | | | | | | | | | |  |
|  | | | 1583.8671 | | | | | | 1583.8666 | | | | | | 0 | | | | | | | | 0 | | | | | | | 438 | | | | | | | | | | | | | 450 | | | | | | | | | | | LFEITPEKNPQLR | | | | | | | | | | | | | | | | | | | | | |  |
|  | | | 1583.8671 | | | | | | 1583.8681 | | | | | | 0 | | | | | | | | 1 | | | | | | | 438 | | | | | | | | | | | | | 450 | | | | | | | | | | | LFEITPEKNPQLR | | | | | | | | | | | | | | | | | | | | | |  |
|  | | | 1583.8671 | | | | | | 1583.8738 | | | | | | 0 | | | | | | | | 4 | | | | | | | 438 | | | | | | | | | | | | | 450 | | | | | | | | | | | LFEITPEKNPQLR | | | | | | | | | | | | | | | | | | | | | |  |
|  | | | 1382.679 | | | | | | 1382.6794 | | | | | | 0 | | | | | | | | 0 | | | | | | | 500 | | | | | | | | | | | | | 510 | | | | | | | | | | | QQQEEQPLEVR | | | | | | | | | | | | | | | | | | | | | |  |
|  | | | 843.4702 | | | | | | 843.4687 | | | | | | 0 | | | | | | | | -2 | | | | | | | 581 | | | | | | | | | | | | | 587 | | | | | | | | | | | DIENLIK | | | | | | | | | | | | | | | | | | | | | |  |
|  | | | 843.4702 | | | | | | 843.4688 | | | | | | 0 | | | | | | | | -2 | | | | | | | 581 | | | | | | | | | | | | | 587 | | | | | | | | | | | DIENLIK | | | | | | | | | | | | | | | | | | | | | |  |
|  | | | 843.4702 | | | | | | 843.4692 | | | | | | 0 | | | | | | | | -1 | | | | | | | 581 | | | | | | | | | | | | | 587 | | | | | | | | | | | DIENLIK | | | | | | | | | | | | | | | | | | | | | |  |
|  | | | 843.4702 | | | | | | 843.4696 | | | | | | 0 | | | | | | | | -1 | | | | | | | 581 | | | | | | | | | | | | | 587 | | | | | | | | | | | DIENLIK | | | | | | | | | | | | | | | | | | | | | |  |
|  | | | 843.4702 | | | | | | 843.4698 | | | | | | 0 | | | | | | | | 0 | | | | | | | 581 | | | | | | | | | | | | | 587 | | | | | | | | | | | DIENLIK | | | | | | | | | | | | | | | | | | | | | |  |
|  | | | 843.4702 | | | | | | 843.4701 | | | | | | 0 | | | | | | | | 0 | | | | | | | 581 | | | | | | | | | | | | | 587 | | | | | | | | | | | DIENLIK | | | | | | | | | | | | | | | | | | | | | |  |
|  | | | 843.4702 | | | | | | 843.4702 | | | | | | 0 | | | | | | | | 0 | | | | | | | 581 | | | | | | | | | | | | | 587 | | | | | | | | | | | DIENLIK | | | | | | | | | | | | | | | | | | | | | |  |
|  | | | 843.4702 | | | | | | 843.4702 | | | | | | 0 | | | | | | | | 0 | | | | | | | 581 | | | | | | | | | | | | | 587 | | | | | | | | | | | DIENLIK | | | | | | | | | | | | | | | | | | | | | |  |
|  | | | 843.4702 | | | | | | 843.4706 | | | | | | 0 | | | | | | | | 1 | | | | | | | 581 | | | | | | | | | | | | | 587 | | | | | | | | | | | DIENLIK | | | | | | | | | | | | | | | | | | | | | |  |
|  | | | 1740.7955 | | | | | | 1740.7926 | | | | | | 0 | | | | | | | | -2 | | | | | | | 588 | | | | | | | | | | | | | 602 | | | | | | | | | | | SQSESYFVDAQPQQK | | | | | | | | | | | | | | | | | | | | | |  |
|  | | | 1740.7955 | | | | | | 1740.7932 | | | | | | 0 | | | | | | | | -1 | | | | | | | 588 | | | | | | | | | | | | | 602 | | | | | | | | | | | SQSESYFVDAQPQQK | | | | | | | | | | | | | | | | | | | | | |  |
|  | | | 1740.7955 | | | | | | 1740.7936 | | | | | | 0 | | | | | | | | -1 | | | | | | | 588 | | | | | | | | | | | | | 602 | | | | | | | | | | | SQSESYFVDAQPQQK | | | | | | | | | | | | | | | | | | | | | |  |
|  | | | 1740.7955 | | | | | | 1740.7936 | | | | | | 0 | | | | | | | | -1 | | | | | | | 588 | | | | | | | | | | | | | 602 | | | | | | | | | | | SQSESYFVDAQPQQK | | | | | | | | | | | | | | | | | | | | | |  |
|  | | | 1740.7955 | | | | | | 1740.7954 | | | | | | 0 | | | | | | | | 0 | | | | | | | 588 | | | | | | | | | | | | | 602 | | | | | | | | | | | SQSESYFVDAQPQQK | | | | | | | | | | | | | | | | | | | | | |  |
| Spot no. | | | Homologous protein | | | | | | Accession no. | | | | | | Score | | | | | | M.P. | | | | | | | Cov. (%) | | | | | | | | | | | | | | | Blast score | | | | | | | | | | | Mr (kDa) / pI | | | | | | | | | | | | | | | | | Ratio | | | | |  |
|  |  |  |  |  |  |  |  |  |  |  |  |  |  |  |  |  |  |  |  |  |  |  |  |  |  |  |  |  |  |  |  |  |  |  |  |  |  |  |  |  |  |  |  |  |  |  |  |  |  |  |  |  |  | Theo. | | | | | | | | | | Exp. | | | | | | |  |  |  |  |  |  |
| 9 | | | beta-conglycinin alpha subunit | | | | | | Glyma20g28650.1 | | | | | | 284 | | | | | | 12 | | | | | | | 18 | | | | | | | | | | | | | | | 783 | | | | | | | | | | | 70.8/5.12 | | | | | | | | | |  | | | | | | |  | | | | |  |
|  | | | Calc. Mass | | | | | | Observ. Mass | | | | | | Da | | | | | | ppm | | | | | | | start | | | | | | | | | | | | | | | end | | | | | | | | | | | sequence | | | | | | | | | | | | | | | | | | | | | |  |
|  | | | 1292.6626 | | | | | | 1292.6566 | | | | | | 0 | | | | | | -5 | | | | | | | 192 | | | | | | | | | | | | | | | 202 | | | | | | | | | | | NKNPFLFGSNR | | | | | | | | | | | | | | | | | | | | | |  |
|  | | | 1292.6626 | | | | | | 1292.6616 | | | | | | 0 | | | | | | -1 | | | | | | | 192 | | | | | | | | | | | | | | | 202 | | | | | | | | | | | NKNPFLFGSNR | | | | | | | | | | | | | | | | | | | | | |  |
|  | | | 1292.6626 | | | | | | 1292.6635 | | | | | | 0 | | | | | | 1 | | | | | | | 192 | | | | | | | | | | | | | | | 202 | | | | | | | | | | | NKNPFLFGSNR | | | | | | | | | | | | | | | | | | | | | |  |
|  | | | 1050.5247 | | | | | | 1050.5246 | | | | | | 0 | | | | | | 0 | | | | | | | 194 | | | | | | | | | | | | | | | 202 | | | | | | | | | | | NPFLFGSNR | | | | | | | | | | | | | | | | | | | | | |  |
|  | | | 783.4167 | | | | | | 783.4167 | | | | | | 0 | | | | | | 0 | | | | | | | 203 | | | | | | | | | | | | | | | 208 | | | | | | | | | | | FETLFK | | | | | | | | | | | | | | | | | | | | | |  |
|  | | | 2151.0233 | | | | | | 2151.0208 | | | | | | 0 | | | | | | -1 | | | | | | | 287 | | | | | | | | | | | | | | | 305 | | | | | | | | | | | VPSGTTYYVVNPDNNENLR | | | | | | | | | | | | | | | | | | | | | |  |
|  | | | 1390.866 | | | | | | 1390.8606 | | | | | | 0 | | | | | | -4 | | | | | | | 306 | | | | | | | | | | | | | | | 318 | | | | | | | | | | | LITLAIPVNKPGR | | | | | | | | | | | | | | | | | | | | | |  |
|  | | | 1390.866 | | | | | | 1390.8608 | | | | | | 0 | | | | | | -4 | | | | | | | 306 | | | | | | | | | | | | | | | 318 | | | | | | | | | | | LITLAIPVNKPGR | | | | | | | | | | | | | | | | | | | | | |  |
|  | | | 1390.866 | | | | | | 1390.8644 | | | | | | 0 | | | | | | -1 | | | | | | | 306 | | | | | | | | | | | | | | | 318 | | | | | | | | | | | LITLAIPVNKPGR | | | | | | | | | | | | | | | | | | | | | |  |
|  | | | 1390.866 | | | | | | 1390.8644 | | | | | | 0 | | | | | | -1 | | | | | | | 306 | | | | | | | | | | | | | | | 318 | | | | | | | | | | | LITLAIPVNKPGR | | | | | | | | | | | | | | | | | | | | | |  |
|  | | | 1390.866 | | | | | | 1390.8658 | | | | | | 0 | | | | | | 0 | | | | | | | 306 | | | | | | | | | | | | | | | 318 | | | | | | | | | | | LITLAIPVNKPGR | | | | | | | | | | | | | | | | | | | | | |  |
|  | | | 1152.5662 | | | | | | 1152.5648 | | | | | | 0 | | | | | | -1 | | | | | | | 340 | | | | | | | | | | | | | | | 349 | | | | | | | | | | | NILEASYDTK | | | | | | | | | | | | | | | | | | | | | |  |
|  | | | 1243.7023 | | | | | | 1243.7008 | | | | | | 0 | | | | | | -1 | | | | | | | 371 | | | | | | | | | | | | | | | 381 | | | | | | | | | | | LQESVIVEISK | | | | | | | | | | | | | | | | | | | | | |  |
|  | | | 1533.8151 | | | | | | 1533.814 | | | | | | 0 | | | | | | -1 | | | | | | | 397 | | | | | | | | | | | | | | | 409 | | | | | | | | | | | KTISSEDKPFNLR | | | | | | | | | | | | | | | | | | | | | |  |
|  | | | 1533.8151 | | | | | | 1533.8152 | | | | | | 0 | | | | | | 0 | | | | | | | 397 | | | | | | | | | | | | | | | 409 | | | | | | | | | | | KTISSEDKPFNLR | | | | | | | | | | | | | | | | | | | | | |  |
|  | | | 1533.8151 | | | | | | 1533.8155 | | | | | | 0 | | | | | | 0 | | | | | | | 397 | | | | | | | | | | 409 | | | | | | | | | | | KTISSEDKPFNLR | | | | | | | | | | | | | | | | | | | | | | | | | | |  |
|  | | | 1533.8151 | | | | | | 1533.8164 | | | | | | 0 | | | | | | 1 | | | | | | | 397 | | | | | | | | | | 409 | | | | | | | | | | | KTISSEDKPFNLR | | | | | | | | | | | | | | | | | | | | | | | | | | |  |
|  | | | 1405.7201 | | | | | | 1405.7194 | | | | | | 0 | | | | | | 0 | | | | | | | 398 | | | | | | | | | | 409 | | | | | | | | | | | TISSEDKPFNLR | | | | | | | | | | | | | | | | | | | | | | | | | | |  |
|  | | | 1405.7201 | | | | | | 1405.7196 | | | | | | 0 | | | | | | 0 | | | | | | | 398 | | | | | | | | | | 409 | | | | | | | | | | | TISSEDKPFNLR | | | | | | | | | | | | | | | | | | | | | | | | | | |  |
|  | | | 1405.7201 | | | | | | 1405.7203 | | | | | | 0 | | | | | | 0 | | | | | | | 398 | | | | | | | | | | 409 | | | | | | | | | | | TISSEDKPFNLR | | | | | | | | | | | | | | | | | | | | | | | | | | |  |
|  | | | 1405.7201 | | | | | | 1405.7209 | | | | | | 0 | | | | | | 1 | | | | | | | 398 | | | | | | | | | | 409 | | | | | | | | | | | TISSEDKPFNLR | | | | | | | | | | | | | | | | | | | | | | | | | | |  |
|  | | | 1405.7201 | | | | | | 1405.7212 | | | | | | 0 | | | | | | 1 | | | | | | | 398 | | | | | | | | | | 409 | | | | | | | | | | | TISSEDKPFNLR | | | | | | | | | | | | | | | | | | | | | | | | | | |  |
|  | | | 1405.7201 | | | | | | 1405.7218 | | | | | | 0 | | | | | | 1 | | | | | | | 398 | | | | | | | | | | 409 | | | | | | | | | | | TISSEDKPFNLR | | | | | | | | | | | | | | | | | | | | | | | | | | |  |
|  | | | 1009.512 | | | | | | 1009.5114 | | | | | | 0 | | | | | | -1 | | | | | | | 422 | | | | | | | | | | 429 | | | | | | | | | | | FFEITPEK | | | | | | | | | | | | | | | | | | | | | | | | | | |  |
|  | | | 1009.512 | | | | | | 1009.5128 | | | | | | 0 | | | | | | 1 | | | | | | | 422 | | | | | | | | | | 429 | | | | | | | | | | | FFEITPEK | | | | | | | | | | | | | | | | | | | | | | | | | | |  |
|  | | | 1009.512 | | | | | | 1009.5136 | | | | | | 0 | | | | | | 2 | | | | | | | 422 | | | | | | | | | | 429 | | | | | | | | | | | FFEITPEK | | | | | | | | | | | | | | | | | | | | | | | | | | |  |
|  | | | 1617.8515 | | | | | | 1617.849 | | | | | | 0 | | | | | | -1 | | | | | | | 422 | | | | | | | | | | 434 | | | | | | | | | | | FFEITPEKNPQLR | | | | | | | | | | | | | | | | | | | | | | | | | | |  |
|  | | | 1617.8515 | | | | | | 1617.8521 | | | | | | 0 | | | | | | 0 | | | | | | | 422 | | | | | | | | | | 434 | | | | | | | | | | | FFEITPEKNPQLR | | | | | | | | | | | | | | | | | | | | | | | | | | |  |
|  | | | 2024.9399 | | | | | | 2024.938 | | | | | | 0 | | | | | | -1 | | | | | | | 479 | | | | | | | | | | 494 | | | | | | | | | | | EQQQEQQQEEQPLEVR | | | | | | | | | | | | | | | | | | | | | | | | | | |  |
| Spot no. | | | Homologous protein | | | | | | Accession no. | | | | | | Score | | | | | | M.P. | | | | | | | Cov. (%) | | | | | | | | | | Blast score | | | | | | | | | | | Mr (kDa) / pI | | | | | | | | | | | | | | | | | | | | | | Ratio | | | | |  |
|  |  |  |  |  |  |  |  |  |  |  |  |  |  |  |  |  |  |  |  |  |  |  |  |  |  |  |  |  |  |  |  |  |  |  |  |  |  |  |  |  |  |  |  |  |  |  |  |  | Theo. | | | | | | | | | | | Exp. | | | | | | | | | | |  |  |  |  |  |  |
| 10a | | | Chain A, Crystal Structure Of Glycinin A3b4 Subunit Homohexamer | | | | | | Glyma13g18450.1 | | | | | | 729 | | | | | | 7 | | | | | | | 23 | | | | | | | | | | 628 | | | | | | | | | | | 47.0/5.27 | | | | | | | | | | |  | | | | | | | | | | |  | | | | |  |
|  | | | Calc. Mass | | | | | | Observ. Mass | | | | | | Da | | | | | | ppm | | | | | | | start | | | | | | | | | | end | | | | | | | | | | | sequence | | | | | | | | | | | | | | | | | | | | | | | | | | |  |
|  | | | 2082.9541 | | | | | | 2082.9554 | | | | | | 0 | | | | | | 1 | | | | | | | 2 | | | | | | | | | | 18 | | | | | | | | | | | FNECQLNNLNALEPDHR | | | | | | | | | | | | | | | | | | | | | | | | | | |  |
|  | | | 2728.2915 | | | | | | 2728.2868 | | | | | | 0 | | | | | | -2 | | | | | | | 158 | | | | | | | | | | 180 | | | | | | | | | | | VFYLAGNPDIEHPETMQQQQQQK | | | | | | | | | | | | | | | | | | | | | | | | | | |  |
|  | | | 2744.2864 | | | | | | 2744.2837 | | | | | | 0 | | | | | | -1 | | | | | | | 158 | | | | | | | | | | 180 | | | | | | | | | | | VFYLAGNPDIEHPETMQQQQQQK | | | | | | | | | | | | | | | | | | | | | | | | | | |  |
|  | | | 2744.2864 | | | | | | 2744.2837 | | | | | | 0 | | | | | | -1 | | | | | | | 158 | | | | | | | | | | 180 | | | | | | | | | | | VFYLAGNPDIEHPETMQQQQQQK | | | | | | | | | | | | | | | | | | | | | | | | | | |  |
|  | | | 2287.0829 | | | | | | 2287.0795 | | | | | | 0 | | | | | | -1 | | | | | | | 186 | | | | | | | | | | 206 | | | | | | | | | | | KQGQHQQQEEEGGSVLSGFSK | | | | | | | | | | | | | | | | | | | | | | | | | | |  |
|  | | | 2287.0829 | | | | | | 2287.0813 | | | | | | 0 | | | | | | -1 | | | | | | | 186 | | | | | | | | | | 206 | | | | | | | | | | | KQGQHQQQEEEGGSVLSGFSK | | | | | | | | | | | | | | | | | | | | | | | | | | |  |
|  | | | 2287.0829 | | | | | | 2287.0819 | | | | | | 0 | | | | | | 0 | | | | | | | 186 | | | | | | | | | | 206 | | | | | | | | | | | KQGQHQQQEEEGGSVLSGFSK | | | | | | | | | | | | | | | | | | | | | | | | | | |  |
|  | | | 2287.0829 | | | | | | 2287.0819 | | | | | | 0 | | | | | | 0 | | | | | | | 186 | | | | | | | | | | 206 | | | | | | | | | | | KQGQHQQQEEEGGSVLSGFSK | | | | | | | | | | | | | | | | | | | | | | | | | | |  |
|  | | | 2287.0829 | | | | | | 2287.0821 | | | | | | 0 | | | | | | 0 | | | | | | | 186 | | | | | | | | | | 206 | | | | | | | | | | | KQGQHQQQEEEGGSVLSGFSK | | | | | | | | | | | | | | | | | | | | | | | | | | |  |
|  | | | 2287.0829 | | | | | | 2287.0821 | | | | | | 0 | | | | | | 0 | | | | | | | 186 | | | | | | | | | | 206 | | | | | | | | | | | KQGQHQQQEEEGGSVLSGFSK | | | | | | | | | | | | | | | | | | | | | | | | | | |  |
|  | | | 2287.0829 | | | | | | 2287.0825 | | | | | | 0 | | | | | | 0 | | | | | | | 186 | | | | | | | | | | 206 | | | | | | | | | | | KQGQHQQQEEEGGSVLSGFSK | | | | | | | | | | | | | | | | | | | | | | | | | | |  |
|  | | | 2287.0829 | | | | | | 2287.0837 | | | | | | 0 | | | | | | 0 | | | | | | | 186 | | | | | | | | | | 206 | | | | | | | | | | | KQGQHQQQEEEGGSVLSGFSK | | | | | | | | | | | | | | | | | | | | | | | | | | |  |
|  | | | 2158.9879 | | | | | | 2158.9846 | | | | | | 0 | | | | | | -2 | | | | | | | 187 | | | | | | | | | | 206 | | | | | | | | | | | QGQHQQQEEEGGSVLSGFSK | | | | | | | | | | | | | | | | | | | | | | | | | | |  |
|  | | | 2158.9879 | | | | | | 2158.9849 | | | | | | 0 | | | | | | -1 | | | | | | | 187 | | | | | | | | | | 206 | | | | | | | | | | | QGQHQQQEEEGGSVLSGFSK | | | | | | | | | | | | | | | | | | | | | | | | | | |  |
|  | | | 2158.9879 | | | | | | 2158.9854 | | | | | | 0 | | | | | | -1 | | | | | | | 187 | | | | | | | | | | 206 | | | | | | | | | | | QGQHQQQEEEGGSVLSGFSK | | | | | | | | | | | | | | | | | | | | | | | | | | |  |
|  | | | 2158.9879 | | | | | | 2158.9861 | | | | | | 0 | | | | | | -1 | | | | | | | 187 | | | | | | | | | | 206 | | | | | | | | | | | QGQHQQQEEEGGSVLSGFSK | | | | | | | | | | | | | | | | | | | | | | | | | | |  |
|  | | | 1850.8435 | | | | | | 1850.8352 | | | | | | 0 | | | | | | -4 | | | | | | | 207 | | | | | | | | | | 222 | | | | | | | | | | | HFLAQSFNTNEDTAEK | | | | | | | | | | | | | | | | | | | | | | | | | | |  |
|  | | | 1850.8435 | | | | | | 1850.8376 | | | | | | 0 | | | | | | -3 | | | | | | | 207 | | | | | | | | | | 222 | | | | | | | | | | | HFLAQSFNTNEDTAEK | | | | | | | | | | | | | | | | | | | | | | | | | | |  |
|  | | | 1850.8435 | | | | | | 1850.8396 | | | | | | 0 | | | | | | -2 | | | | | | | 207 | | | | | | | | | | 222 | | | | | | | | | | | HFLAQSFNTNEDTAEK | | | | | | | | | | | | | | | | | | | | | | | | | | |  |
|  | | | 1850.8435 | | | | | | 1850.8399 | | | | | | 0 | | | | | | -2 | | | | | | | 207 | | | | | | | | | | 222 | | | | | | | | | | | HFLAQSFNTNEDTAEK | | | | | | | | | | | | | | | | | | | | | | | | | | |  |
|  | | | 1850.8435 | | | | | | 1850.8404 | | | | | | 0 | | | | | | -2 | | | | | | | 207 | | | | | | | | | | 222 | | | | | | | | | | | HFLAQSFNTNEDTAEK | | | | | | | | | | | | | | | | | | | | | | | | | | |  |
|  | | | 1850.8435 | | | | | | 1850.8424 | | | | | | 0 | | | | | | -1 | | | | | | | 207 | | | | | | | | | | 222 | | | | | | | | | | | HFLAQSFNTNEDTAEK | | | | | | | | | | | | | | | | | | | | | | | | | | |  |
|  | | | 1850.8435 | | | | | | 1850.8432 | | | | | | 0 | | | | | | 0 | | | | | | | 207 | | | | | | | | | | 222 | | | | | | | | | | | HFLAQSFNTNEDTAEK | | | | | | | | | | | | | | | | | | | | | | | | | | |  |
|  | | | 1850.8435 | | | | | | 1850.8436 | | | | | | 0 | | | | | | 0 | | | | | | | 207 | | | | | | | | | | 222 | | | | | | | | | | | HFLAQSFNTNEDTAEK | | | | | | | | | | | | | | | | | | | | | | | | | | |  |
|  | | | 1850.8435 | | | | | | 1850.8441 | | | | | | 0 | | | | | | 0 | | | | | | | 207 | | | | | | | | | | 222 | | | | | | | | | | | HFLAQSFNTNEDTAEK | | | | | | | | | | | | | | | | | | | | | | | | | | |  |
|  | | | 1850.8435 | | | | | | 1850.8468 | | | | | | 0 | | | | | | 2 | | | | | | | 207 | | | | | | | | | | 222 | | | | | | | | | | | HFLAQSFNTNEDTAEK | | | | | | | | | | | | | | | | | | | | | | | | | | |  |
|  | | | 2120.0287 | | | | | | 2120.0239 | | | | | | 0 | | | | | | -2 | | | | | | | 207 | | | | | | | | | | 224 | | | | | | | | | | | HFLAQSFNTNEDTAEKLR | | | | | | | | | | | | | | | | | | | | | | | | | | |  |
|  | | | 2120.0287 | | | | | | 2120.0277 | | | | | | 0 | | | | | | 0 | | | | | | | 207 | | | | | | | | | | 224 | | | | | | | | | | | HFLAQSFNTNEDTAEKLR | | | | | | | | | | | | | | | | | | | | | | | | | | |  |
|  | | | 2120.0287 | | | | | | 2120.0278 | | | | | | 0 | | | | | | 0 | | | | | | | 207 | | | | | | | | | | 224 | | | | | | | | | | | HFLAQSFNTNEDTAEKLR | | | | | | | | | | | | | | | | | | | | | | | | | | |  |
|  | | | 2120.0287 | | | | | | 2120.0289 | | | | | | 0 | | | | | | 0 | | | | | | | 207 | | | | | | | | | | 224 | | | | | | | | | | | HFLAQSFNTNEDTAEKLR | | | | | | | | | | | | | | | | | | | | | | | | | | |  |
|  | | | 1653.9665 | | | | | | 1653.9616 | | | | | | 0 | | | | | | -3 | | | | | | | 231 | | | | | | | | | | 246 | | | | | | | | | | | KQIVTVEGGLSVISPK | | | | | | | | | | | | | | | | | | | | | | | | | | |  |
| Spot no. | | | | | | Homologous protein | | | | Accession no. | | | | | | Score | | | | | | M.P. | | | | | | | | Cov. (%) | | | | | | | | | Blast score | | | | | | | | | | | Mr (kDa) / pI | | | | | | | | | | | | | | | | | | | | | | Ratio | | | | |
|  |  |  |  |  |  |  |  |  |  |  |  |  |  |  |  |  |  |  |  |  |  |  |  |  |  |  |  |  |  |  |  |  |  |  |  |  |  |  |  |  |  |  |  |  |  |  |  |  |  | Theo. | | | | | | | | | | | Exp. | | | | | | | | | | |  |  |  |  |  |
| 10b | | | | | | Os05g0553000 | | | | Glyma10g41330.1 | | | | | | 108 | | | | | | 3 | | | | | | | | 7 | | | | | | | | | 879 | | | | | | | | | | | 60.1/5.8 | | | | | | | | | | |  | | | | | | | | | | |  | | | | |
|  | | | | | | Calc. Mass | | | | Observ. Mass | | | | | | Da | | | | | | ppm | | | | | | | | start | | | | | | | | | | | | | end | | | | | | | | | | | sequence | | | | | | | | | | | | | | | | | | | | | | |
|  | | | | | | 1617.9202 | | | | 1617.9208 | | | | | | 0 | | | | | | 0 | | | | | | | | 122 | | | | | | | | | | | | | 136 | | | | | | | | | | | LVLEVAQHLGEGVVR | | | | | | | | | | | | | | | | | | | | | | |
|  | | | | | | 1398.762 | | | | 1398.7603 | | | | | | 0 | | | | | | -1 | | | | | | | | 311 | | | | | | | | | | | | | 323 | | | | | | | | | | | VGLTGLTVAEHFR | | | | | | | | | | | | | | | | | | | | | | |
|  | | | | | | 1491.7681 | | | | 1491.7638 | | | | | | 0 | | | | | | -3 | | | | | | | | 340 | | | | | | | | | | | | | 353 | | | | | | | | | | | FTQANSEVSALLGR | | | | | | | | | | | | | | | | | | | | | | |
| Spot no. | | | Homologous protein | | | | | | Accession no. | | | | | | Score | | | | | | M.P. | | | | | | | | Cov. (%) | | | | | | | | | Blast score | | | | | | | | | | | Mr (kDa) / pI | | | | | | | | | | | | | | | | | | | | | | Ratio | | | | |  |
|  |  |  |  |  |  |  |  |  |  |  |  |  |  |  |  |  |  |  |  |  |  |  |  |  |  |  |  |  |  |  |  |  |  |  |  |  |  |  |  |  |  |  |  |  |  |  |  |  | Theo. | | | | | | | | | | | Exp. | | | | | | | | | | |  |  |  |  |  |  |
| 12a | | | beta-conglycinin alpha subunit | | | | | | Glyma20g28650.1 | | | | | | 155 | | | | | | 8 | | | | | | | | 14 | | | | | | | | | 783 | | | | | | | | | | | 70.8/5.12 | | | | | | | | | | |  | | | | | | | | | | |  | | | | |  |
|  | | | Calc. Mass | | | | | | Observ. Mass | | | | | | Da | | | | | | ppm | | | | | | | | start | | | | | | | | | | | end | | | | | | | | | | | sequence | | | | | | | | | | | | | | | | | | | | | | | | |  |
|  | | | 1377.7306 | | | | | | 1377.7309 | | | | | | 0 | | | | | | 0 | | | | | | | | 152 | | | | | | | | | | | 162 | | | | | | | | | | | QFPFPRPPHQK | | | | | | | | | | | | | | | | | | | | | | | | |  |
|  | | | 1050.5247 | | | | | | 1050.5238 | | | | | | 0 | | | | | | -1 | | | | | | | | 194 | | | | | | | | | | | 202 | | | | | | | | | | | NPFLFGSNR | | | | | | | | | | | | | | | | | | | | | | | | |  |
|  | | | 1050.5247 | | | | | | 1050.5246 | | | | | | 0 | | | | | | 0 | | | | | | | | 194 | | | | | | | | | | | 202 | | | | | | | | | | | NPFLFGSNR | | | | | | | | | | | | | | | | | | | | | | | | |  |
|  | | | 1388.716 | | | | | | 1388.7148 | | | | | | 0 | | | | | | -1 | | | | | | | | 224 | | | | | | | | | | | 234 | | | | | | | | | | | SPQLQNLRDYR | | | | | | | | | | | | | | | | | | | | | | | | |  |
|  | | | 2151.0233 | | | | | | 2151.0214 | | | | | | 0 | | | | | | -1 | | | | | | | | 287 | | | | | | | | | | | 305 | | | | | | | | | | | VPSGTTYYVVNPDNNENLR | | | | | | | | | | | | | | | | | | | | | | | | |  |
|  | | | 2151.0233 | | | | | | 2151.0217 | | | | | | 0 | | | | | | -1 | | | | | | | | 287 | | | | | | | | | | | 305 | | | | | | | | | | | VPSGTTYYVVNPDNNENLR | | | | | | | | | | | | | | | | | | | | | | | | |  |
|  | | | 2151.0233 | | | | | | 2151.0234 | | | | | | 0 | | | | | | 0 | | | | | | | | 287 | | | | | | | | | | | 305 | | | | | | | | | | | VPSGTTYYVVNPDNNENLR | | | | | | | | | | | | | | | | | | | | | | | | |  |
|  | | | 1390.866 | | | | | | 1390.8644 | | | | | | 0 | | | | | | -1 | | | | | | | | 306 | | | | | | | | | | | 318 | | | | | | | | | | | LITLAIPVNKPGR | | | | | | | | | | | | | | | | | | | | | | | | |  |
|  | | | 1390.866 | | | | | | 1390.867 | | | | | | 0 | | | | | | 1 | | | | | | | | 306 | | | | | | | | | | | 318 | | | | | | | | | | | LITLAIPVNKPGR | | | | | | | | | | | | | | | | | | | | | | | | |  |
|  | | | 1533.8151 | | | | | | 1533.8134 | | | | | | 0 | | | | | | -1 | | | | | | | | 397 | | | | | | | | | | | 409 | | | | | | | | | | | KTISSEDKPFNLR | | | | | | | | | | | | | | | | | | | | | | | | |  |
|  | | | 1533.8151 | | | | | | 1533.8149 | | | | | | 0 | | | | | | 0 | | | | | | | | 397 | | | | | | | | | | | 409 | | | | | | | | | | | KTISSEDKPFNLR | | | | | | | | | | | | | | | | | | | | | | | | |  |
|  | | | 1533.8151 | | | | | | 1533.8158 | | | | | | 0 | | | | | | 0 | | | | | | | | 397 | | | | | | | | | | | 409 | | | | | | | | | | | KTISSEDKPFNLR | | | | | | | | | | | | | | | | | | | | | | | | |  |
|  | | | 1533.8151 | | | | | | 1533.8158 | | | | | | 0 | | | | | | 0 | | | | | | | | 397 | | | | | | | | | | | 409 | | | | | | | | | | | KTISSEDKPFNLR | | | | | | | | | | | | | | | | | | | | | | | | |  |
|  | | | 1533.8151 | | | | | | 1533.8167 | | | | | | 0 | | | | | | 1 | | | | | | | | 397 | | | | | | | | | | | 409 | | | | | | | | | | | KTISSEDKPFNLR | | | | | | | | | | | | | | | | | | | | | | | | |  |
|  | | | 1405.7201 | | | | | | 1405.7186 | | | | | | 0 | | | | | | -1 | | | | | | | | 398 | | | | | | | | | | | 409 | | | | | | | | | | | TISSEDKPFNLR | | | | | | | | | | | | | | | | | | | | | | | | |  |
|  | | | 1405.7201 | | | | | | 1405.7192 | | | | | | 0 | | | | | | -1 | | | | | | | | 398 | | | | | | | | | | | 409 | | | | | | | | | | | TISSEDKPFNLR | | | | | | | | | | | | | | | | | | | | | | | | |  |
|  | | | 1405.7201 | | | | | | 1405.7197 | | | | | | 0 | | | | | | 0 | | | | | | | | 398 | | | | | | | | | | | 409 | | | | | | | | | | | TISSEDKPFNLR | | | | | | | | | | | | | | | | | | | | | | | | |  |
|  | | | 1405.7201 | | | | | | 1405.7206 | | | | | | 0 | | | | | | 0 | | | | | | | | 398 | | | | | | | | | | | 409 | | | | | | | | | | | TISSEDKPFNLR | | | | | | | | | | | | | | | | | | | | | | | | |  |
|  | | | 1617.8515 | | | | | | 1617.8524 | | | | | | 0 | | | | | | 1 | | | | | | | | 422 | | | | | | | | | | | 434 | | | | | | | | | | | FFEITPEKNPQLR | | | | | | | | | | | | | | | | | | | | | | | | |  |
| Spot no. | | | Homologous protein | | | | | | Accession no. | | | | | | Score | | | | | | M.P. | | | | | | | | | Cov. (%) | | | | | | | | | | Blast score | | | | | | | | | | | Mr (kDa) / pI | | | | | | | | | | | | | | | | | | | | Ratio | | | | |  |
|  |  |  |  |  |  |  |  |  |  |  |  |  |  |  |  |  |  |  |  |  |  |  |  |  |  |  |  |  |  |  |  |  |  |  |  |  |  |  |  |  |  |  |  |  |  |  |  |  |  |  | Theo. | | | | | | | | | | | | | Exp. | | | | | | |  |  |  |  |  |  |
| 12b | | | Os05g0553000 | | | | | | Glyma10g41330.1 | | | | | | 118 | | | | | | 3 | | | | | | | | | 6 | | | | | | | | | | 879 | | | | | | | | | | | 60.1/5.8 | | | | | | | | | | | | |  | | | | | | |  | | | | |  |
|  | | | Calc. Mass | | | | | | Observ. Mass | | | | | | Da | | | | | | ppm | | | | | | | | | start | | | | | | | | | | end | | | | | | | | | | | sequence | | | | | | | | | | | | | | | | | | | | | | | | |  |
|  | | | 1408.8038 | | | | | | 1408.8024 | | | | | | 0 | | | | | | -1 | | | | | | | | | 152 | | | | | | | | | | 165 | | | | | | | | | | | VLNTGSPITVPVGR | | | | | | | | | | | | | | | | | | | | | | | | |  |
|  | | | 974.5549 | | | | | | 974.5556 | | | | | | 0 | | | | | | 1 | | | | | | | | | 229 | | | | | | | | | | 239 | | | | | | | | | | | IGLFGGAGVGK | | | | | | | | | | | | | | | | | | | | | | | | |  |
|  | | | 1491.7681 | | | | | | 1491.7662 | | | | | | 0 | | | | | | -1 | | | | | | | | | 340 | | | | | | | | | | 353 | | | | | | | | | | | FTQANSEVSALLGR | | | | | | | | | | | | | | | | | | | | | | | | |  |
| Spot no. | | | Homologous protein | | | | | | Accession no. | | | | | | Score | | | | | | M.P. | | | | | | | | | Cov. (%) | | | | | | | | | | Blast score | | | | | | | | | | | Mr (kDa) / pI | | | | | | | | | | | | | | | | | | | | | | | Ratio | |  |
|  |  |  |  |  |  |  |  |  |  |  |  |  |  |  |  |  |  |  |  |  |  |  |  |  |  |  |  |  |  |  |  |  |  |  |  |  |  |  |  |  |  |  |  |  |  |  |  |  |  |  | Theo. | | | | | | | | | | | | | Exp. | | | | | | | | | |  |  |  |
| 12c | | | Chain A, Crystal Structure Of Glycinin A3b4 Subunit Homohexamer | | | | | | Glyma13g18450.1 | | | | | | 100 | | | | | | 4 | | | | | | | | | 9 | | | | | | | | | | 628 | | | | | | | | | | | 47.0/5.27 | | | | | | | | | | | | |  | | | | | | | | | |  | |  |
|  | | | Calc. Mass | | | | | | Observ. Mass | | | | | | Da | | | | | | ppm | | | | | | | | | start | | | | | | | | | | end | | | | | | | | | | | sequence | | | | | | | | | | | | | | | | | | | | | | | | |  |
|  | | | 2287.0829 | | | | | | 2287.0825 | | | | | | 0 | | | | | | 0 | | | | | | | | | 186 | | | | | | | | | | 206 | | | | | | | | | | | KQGQHQQQEEEGGSVLSGFSK | | | | | | | | | | | | | | | | | | | | | | | | |  |
|  | | | 2158.9879 | | | | | | 2158.9885 | | | | | | 0 | | | | | | 0 | | | | | | | | | 187 | | | | | | | | | | 206 | | | | | | | | | | | QGQHQQQEEEGGSVLSGFSK | | | | | | | | | | | | | | | | | | | | | | | | |  |
|  | | | 1850.8435 | | | | | | 1850.8408 | | | | | | 0 | | | | | | -1 | | | | | | | | | 207 | | | | | | | | | | 222 | | | | | | | | | | | HFLAQSFNTNEDTAEK | | | | | | | | | | | | | | | | | | | | | | | | |  |
|  | | | 1850.8435 | | | | | | 1850.8438 | | | | | | 0 | | | | | | 0 | | | | | | | | | 207 | | | | | | | | | | 222 | | | | | | | | | | | HFLAQSFNTNEDTAEK | | | | | | | | | | | | | | | | | | | | | | | | |  |
|  | | | 2120.0287 | | | | | | 2120.035 | | | | | | 0 | | | | | | 3 | | | | | | | | | 207 | | | | | | | | | | 224 | | | | | | | | | | | HFLAQSFNTNEDTAEKLR | | | | | | | | | | | | | | | | | | | | | | | | |  |
| Spot no. | | | | Homologous protein | | | | | | Accession no. | | | | | | | Score | | | | | | M.P. | | | | | | | | Cov. (%) | | | | | | | | | | | | Blast score | | | | | | | | | | | Mr (kDa) / pI | | | | | | | | | | | | | | | | | | | | Ratio | |  |
|  |  |  |  |  |  |  |  |  |  |  |  |  |  |  |  |  |  |  |  |  |  |  |  |  |  |  |  |  |  |  |  |  |  |  |  |  |  |  |  |  |  |  |  |  |  |  |  |  |  |  |  |  |  | Theo. | | | | | | | | | | Exp. | | | | | | | | | |  |  |  |
| 13 | | | | Os05g0553000 | | | | | | Glyma10g41330.1 | | | | | | | 124 | | | | | | 3 | | | | | | | | 7 | | | | | | | | | | | | 879 | | | | | | | | | | | 60.1/5.8 | | | | | | | | | |  | | | | | | | | | |  | |  |
|  | | | | Calc. Mass | | | | | | Observ. Mass | | | | | | | Da | | | | | | ppm | | | | | | | | start | | | | | | | | | | | | end | | | | | | | | | | | sequence | | | | | | | | | | | | | | | | | | | | | |  |
|  | | | | 1408.8038 | | | | | | 1408.8014 | | | | | | | 0 | | | | | | -2 | | | | | | | | 152 | | | | | | | | | | | | 165 | | | | | | | | | | | VLNTGSPITVPVGR | | | | | | | | | | | | | | | | | | | | | |  |
|  | | | | 1398.762 | | | | | | 1398.7627 | | | | | | | 0 | | | | | | 1 | | | | | | | | 311 | | | | | | | | | | | | 323 | | | | | | | | | | | VGLTGLTVAEHFR | | | | | | | | | | | | | | | | | | | | | |  |
|  | | | | 1491.7681 | | | | | | 1491.7666 | | | | | | | 0 | | | | | | -1 | | | | | | | | 340 | | | | | | | | | | | | 353 | | | | | | | | | | | FTQANSEVSALLGR | | | | | | | | | | | | | | | | | | | | | |  |
|  | | | | 1491.7681 | | | | | | 1491.7668 | | | | | | | 0 | | | | | | -1 | | | | | | | | 340 | | | | | | | | | | | | 353 | | | | | | | | | | | FTQANSEVSALLGR | | | | | | | | | | | | | | | | | | | | | |  |
| Spot no. | | | | Homologous protein | | | | | | Accession no. | | | | | | | Score | | | | | | M.P. | | | | | | | | Cov. (%) | | | | | | | | | | | | Blast score | | | | | | | | | | | Mr (kDa) / pI | | | | | | | | | | | | | | | | | Ratio | | | | |  |
|  |  |  |  |  |  |  |  |  |  |  |  |  |  |  |  |  |  |  |  |  |  |  |  |  |  |  |  |  |  |  |  |  |  |  |  |  |  |  |  |  |  |  |  |  |  |  |  |  |  |  |  |  |  | Theo. | | | | | | | | | | Exp. | | | | | | |  |  |  |  |  |  |
| 16a | | | | Sucrose-binding protein | | | | | | Glyma10g03390.1 | | | | | | | 551 | | | | | | 9 | | | | | | | | 23 | | | | | | | | | | | | 723 | | | | | | | | | | | 58.6/6.08 | | | | | | | | | |  | | | | | | |  | | | | |  |
|  | | | | Calc. Mass | | | | | | Observ. Mass | | | | | | | Da | | | | | | ppm | | | | | | | | start | | | | | | | | | | | | end | | | | | | | | | | | sequence | | | | | | | | | | | | | | | | | | | | | |  |
|  | | | | 1805.7877 | | | | | | 1805.7816 | | | | | | | 0 | | | | | | -3 | | | | | | | | 30 | | | | | | | | | | | | 44 | | | | | | | | | | | ETEVEEEDPELVTCK | | | | | | | | | | | | | | | | | | | | | |  |
|  | | | | 1805.7877 | | | | | | 1805.7847 | | | | | | | 0 | | | | | | -2 | | | | | | | | 30 | | | | | | | | | | | | 44 | | | | | | | | | | | ETEVEEEDPELVTCK | | | | | | | | | | | | | | | | | | | | | |  |
|  | | | | 1805.7877 | | | | | | 1805.785 | | | | | | | 0 | | | | | | -1 | | | | | | | | 30 | | | | | | | | | | | | 44 | | | | | | | | | | | ETEVEEEDPELVTCK | | | | | | | | | | | | | | | | | | | | | |  |
|  | | | | 1805.7877 | | | | | | 1805.7859 | | | | | | | 0 | | | | | | -1 | | | | | | | | 30 | | | | | | | | | | | | 44 | | | | | | | | | | | ETEVEEEDPELVTCK | | | | | | | | | | | | | | | | | | | | | |  |
|  | | | | 1805.7877 | | | | | | 1805.7866 | | | | | | | 0 | | | | | | -1 | | | | | | | | 30 | | | | | | | | | | | | 44 | | | | | | | | | | | ETEVEEEDPELVTCK | | | | | | | | | | | | | | | | | | | | | |  |
|  | | | | 1303.7248 | | | | | | 1303.7248 | | | | | | | 0 | | | | | | 0 | | | | | | | | 139 | | | | | | | | | | | | 149 | | | | | | | | | | | SKLLQGIENFR | | | | | | | | | | | | | | | | | | | | | |  |
|  | | | | 1360.7086 | | | | | | 1360.7074 | | | | | | | 0 | | | | | | -1 | | | | | | | | 179 | | | | | | | | | | | | 191 | | | | | | | | | | | AVLGLVSESETEK | | | | | | | | | | | | | | | | | | | | | |  |
|  | | | | 1360.7086 | | | | | | 1360.7074 | | | | | | | 0 | | | | | | -1 | | | | | | | | 179 | | | | | | | | | | | | 191 | | | | | | | | | | | AVLGLVSESETEK | | | | | | | | | | | | | | | | | | | | | |  |
|  | | | | 1360.7086 | | | | | | 1360.7076 | | | | | | | 0 | | | | | | -1 | | | | | | | | 179 | | | | | | | | | | | | 191 | | | | | | | | | | | AVLGLVSESETEK | | | | | | | | | | | | | | | | | | | | | |  |
|  | | | | 1141.5193 | | | | | | 1141.519 | | | | | | | 0 | | | | | | 0 | | | | | | | | 236 | | | | | | | | | | | | 245 | | | | | | | | | | | FEEFFGPGGR | | | | | | | | | | | | | | | | | | | | | |  |
|  | | | | 1723.8529 | | | | | | 1723.851 | | | | | | | 0 | | | | | | -1 | | | | | | | | 273 | | | | | | | | | | | | 287 | | | | | | | | | | | LFDQQNEGSIFAISR | | | | | | | | | | | | | | | | | | | | | |  |
|  | | | | 1723.8529 | | | | | | 1723.8514 | | | | | | | 0 | | | | | | -1 | | | | | | | | 273 | | | | | | | | | | | | 287 | | | | | | | | | | | LFDQQNEGSIFAISR | | | | | | | | | | | | | | | | | | | | | |  |
|  | | | | 1886.901 | | | | | | 1886.8962 | | | | | | | 0 | | | | | | -3 | | | | | | | | 328 | | | | | | | | | | | | 343 | | | | | | | | | | | LTEVGPDDDEKSWLQR | | | | | | | | | | | | | | | | | | | | | |  |
|  | | | | 1886.901 | | | | | | 1886.8962 | | | | | | | 0 | | | | | | -3 | | | | | | | | 328 | | | | | | | | | | | | 343 | | | | | | | | | | | LTEVGPDDDEKSWLQR | | | | | | | | | | | | | | | | | | | | | |  |
|  | | | | 1886.901 | | | | | | 1886.8996 | | | | | | | 0 | | | | | | -1 | | | | | | | | 328 | | | | | | | | | | | | 343 | | | | | | | | | | | LTEVGPDDDEKSWLQR | | | | | | | | | | | | | | | | | | | | | |  |
|  | | | | 855.5178 | | | | | | 855.5176 | | | | | | | 0 | | | | | | 0 | | | | | | | | 370 | | | | | | | | | | | | 377 | | | | | | | | | | | IALVIDGR | | | | | | | | | | | | | | | | | | | | | |  |
|  | | | | 855.5178 | | | | | | 855.5186 | | | | | | | 0 | | | | | | 1 | | | | | | | | 370 | | | | | | | | | | | | 377 | | | | | | | | | | | IALVIDGR | | | | | | | | | | | | | | | | | | | | | |  |
|  | | | | 1273.6514 | | | | | | 1273.6512 | | | | | | | 0 | | | | | | 0 | | | | | | | | 455 | | | | | | | | | | | | 466 | | | | | | | | | | | DNIVSSLDNVAK | | | | | | | | | | | | | | | | | | | | | |  |
|  | | | | 1273.6514 | | | | | | 1273.6514 | | | | | | | 0 | | | | | | 0 | | | | | | | | 455 | | | | | | | | | | | | 466 | | | | | | | | | | | DNIVSSLDNVAK | | | | | | | | | | | | | | | | | | | | | |  |
|  | | | | 1273.6514 | | | | | | 1273.6518 | | | | | | | 0 | | | | | | 0 | | | | | | | | 455 | | | | | | | | | | | | 466 | | | | | | | | | | | DNIVSSLDNVAK | | | | | | | | | | | | | | | | | | | | | |  |
|  | | | | 2002.9095 | | | | | | 2002.9054 | | | | | | | 0 | | | | | | -2 | | | | | | | | 467 | | | | | | | | | | | | 483 | | | | | | | | | | | ELAFNYPSEMVNGVFDR | | | | | | | | | | | | | | | | | | | | | |  |
| Spot no. | | | | | Homologous protein | | | | | | Accession no. | | | | | | | Score | | | | | | M.P. | | | | | | | | Cov. (%) | | | | | | | | | Blast score | | | | | | | | | | | Mr (kDa) / pI | | | | | | | | | | | | | | | | | Ratio | | | | | | | |
|  |  |  |  |  |  |  |  |  |  |  |  |  |  |  |  |  |  |  |  |  |  |  |  |  |  |  |  |  |  |  |  |  |  |  |  |  |  |  |  |  |  |  |  |  |  |  |  |  |  |  |  | Theo. | | | | | | | | | | Exp. | | | | | | |  |  |  |  |  |  |  |  |
| 16b | | | | | Lipoxygenase | | | | | | Glyma13g42340.1 | | | | | | | 130 | | | | | | 4 | | | | | | | | 6 | | | | | | | | | 1481 | | | | | | | | | | | 93.2/6.52 | | | | | | | | | |  | | | | | | |  | | | | | | | |
|  | | | | | Calc. Mass | | | | | | Observ. Mass | | | | | | | Da | | | | | | ppm | | | | | | | | start | | | | | | | | | | end | | | | | | | | | | | sequence | | | | | | | | | | | | | | | | | | | | | | | |
|  | | | | | 1301.7191 | | | | | | 1301.7176 | | | | | | | 0 | | | | | | -1 | | | | | | | | 23 | | | | | | | | | | 34 | | | | | | | | | | | NVLDINSITSVK | | | | | | | | | | | | | | | | | | | | | | | |
|  | | | | | 2061.8803 | | | | | | 2061.8814 | | | | | | | 0 | | | | | | 1 | | | | | | | | 200 | | | | | | | | | | 217 | | | | | | | | | | | IYDYDVYNDLGNPDSGDK | | | | | | | | | | | | | | | | | | | | | | | |
|  | | | | | 1083.5601 | | | | | | 1083.5638 | | | | | | | 0 | | | | | | 3 | | | | | | | | 267 | | | | | | | | | | 276 | | | | | | | | | | | SSDFLAFGIK | | | | | | | | | | | | | | | | | | | | | | | |
|  | | | | | 1637.8665 | | | | | | 1637.8658 | | | | | | | 0 | | | | | | 0 | | | | | | | | 308 | | | | | | | | | | 322 | | | | | | | | | | | LYEGGVTLPTNFLSK | | | | | | | | | | | | | | | | | | | | | | | |
| Spot no. | | | | | Homologous protein | | | | | | Accession no. | | | | | | | Score | | | | | | M.P. | | | | | | | | Cov. (%) | | | | | | | | | Blast score | | | | | | | | | | | Mr (kDa) / pI | | | | | | | | | | | | | | | | | | Ratio | | | | |  |  |
|  |  |  |  |  |  |  |  |  |  |  |  |  |  |  |  |  |  |  |  |  |  |  |  |  |  |  |  |  |  |  |  |  |  |  |  |  |  |  |  |  |  |  |  |  |  |  |  |  |  |  |  | Theo. | | | | | | | | | | | Exp. | | | | | | |  |  |  |  |  |  |  |
| 17a | | | | | lipoxygenase L-3 | | | | | | Glyma15g03030.1 | | | | | | | 175 | | | | | | 12 | | | | | | | | 13 | | | | | | | | | 1717 | | | | | | | | | | | 97.3/6.12 | | | | | | | | | | |  | | | | | | |  | | | | |  |  |
|  | | | | | Calc. Mass | | | | | | Observ. Mass | | | | | | | Da | | | | | | ppm | | | | | | | | start | | | | | | | | | | end | | | | | | | | | | | sequence | | | | | | | | | | | | | | | | | | | | | |  |  |
|  | | | | | 1145.6656 | | | | | | 1145.6656 | | | | | | | 0 | | | | | | 0 | | | | | | | | 57 | | | | | | | | | | 67 | | | | | | | | | | | SVSLQLISATK | | | | | | | | | | | | | | | | | | | | | |  |  |
|  | | | | | 1701.9261 | | | | | | 1701.925 | | | | | | | 0 | | | | | | -1 | | | | | | | | 57 | | | | | | | | | | 73 | | | | | | | | | | | SVSLQLISATKADANGK | | | | | | | | | | | | | | | | | | | | | |  |  |
|  | | | | | 1357.6738 | | | | | | 1357.6729 | | | | | | | 0 | | | | | | -1 | | | | | | | | 179 | | | | | | | | | | 188 | | | | | | | | | | | YREEELHNLR | | | | | | | | | | | | | | | | | | | | | |  |  |
|  | | | | | 865.5637 | | | | | | 865.5618 | | | | | | | 0 | | | | | | -2 | | | | | | | | 325 | | | | | | | | | | 332 | | | | | | | | | | | ISPLPVLK | | | | | | | | | | | | | | | | | | | | | |  |  |
|  | | | | | 865.5637 | | | | | | 865.5638 | | | | | | | 0 | | | | | | 0 | | | | | | | | 325 | | | | | | | | | | 332 | | | | | | | | | | | ISPLPVLK | | | | | | | | | | | | | | | | | | | | | |  |  |
|  | | | | | 1426.7456 | | | | | | 1426.7443 | | | | | | | 0 | | | | | | -1 | | | | | | | | 337 | | | | | | | | | | 349 | | | | | | | | | | | TDGEQALKFPPPK | | | | | | | | | | | | | | | | | | | | | |  |  |
|  | | | | | 1426.7456 | | | | | | 1426.7456 | | | | | | | 0 | | | | | | 0 | | | | | | | | 337 | | | | | | | | | | 349 | | | | | | | | | | | TDGEQALKFPPPK | | | | | | | | | | | | | | | | | | | | | |  |  |
|  | | | | | 1905.9432 | | | | | | 1905.9409 | | | | | | | 0 | | | | | | -1 | | | | | | | | 387 | | | | | | | | | | 403 | | | | | | | | | | | SKLDSQVYGDHTSQITK | | | | | | | | | | | | | | | | | | | | | |  |  |
|  | | | | | 1905.9432 | | | | | | 1905.9425 | | | | | | | 0 | | | | | | 0 | | | | | | | | 387 | | | | | | | | | | 403 | | | | | | | | | | | SKLDSQVYGDHTSQITK | | | | | | | | | | | | | | | | | | | | | |  |  |
|  | | | | | 1905.9432 | | | | | | 1905.9427 | | | | | | | 0 | | | | | | 0 | | | | | | | | 387 | | | | | | | | | | 403 | | | | | | | | | | | SKLDSQVYGDHTSQITK | | | | | | | | | | | | | | | | | | | | | |  |  |
|  | | | | | 1905.9432 | | | | | | 1905.943 | | | | | | | 0 | | | | | | 0 | | | | | | | | 387 | | | | | | | | | | 403 | | | | | | | | | | | SKLDSQVYGDHTSQITK | | | | | | | | | | | | | | | | | | | | | |  |  |
|  | | | | | 1690.8162 | | | | | | 1690.8172 | | | | | | | 0 | | | | | | 1 | | | | | | | | 389 | | | | | | | | | | 403 | | | | | | | | | | | LDSQVYGDHTSQITK | | | | | | | | | | | | | | | | | | | | | |  |  |
|  | | | | | 1103.5393 | | | | | | 1103.5386 | | | | | | | 0 | | | | | | -1 | | | | | | | | 553 | | | | | | | | | | 562 | | | | | | | | | | | DTMNINGLAR | | | | | | | | | | | | | | | | | | | | | |  |  |
|  | | | | | 1103.5393 | | | | | | 1103.539 | | | | | | | 0 | | | | | | 0 | | | | | | | | 553 | | | | | | | | | | 562 | | | | | | | | | | | DTMNINGLAR | | | | | | | | | | | | | | | | | | | | | |  |  |
|  | | | | | 1694.7981 | | | | | | 1694.7976 | | | | | | | 0 | | | | | | 0 | | | | | | | | 607 | | | | | | | | | | 621 | | | | | | | | | | | RGMAIEDPSCPHGIR | | | | | | | | | | | | | | | | | | | | | |  |  |
|  | | | | | 1694.7981 | | | | | | 1694.7985 | | | | | | | 0 | | | | | | 0 | | | | | | | | 607 | | | | | | | | | | 621 | | | | | | | | | | | RGMAIEDPSCPHGIR | | | | | | | | | | | | | | | | | | | | | |  |  |
|  | | | | | 1538.697 | | | | | | 1538.6926 | | | | | | | 0 | | | | | | -3 | | | | | | | | 608 | | | | | | | | | | 621 | | | | | | | | | | | GMAIEDPSCPHGIR | | | | | | | | | | | | | | | | | | | | | |  |  |
|  | | | | | 1538.697 | | | | | | 1538.6968 | | | | | | | 0 | | | | | | 0 | | | | | | | | 608 | | | | | | | | | | 621 | | | | | | | | | | | GMAIEDPSCPHGIR | | | | | | | | | | | | | | | | | | | | | |  |  |
|  | | | | | 1961.8789 | | | | | | 1961.8786 | | | | | | | 0 | | | | | | 0 | | | | | | | | 654 | | | | | | | | | | 669 | | | | | | | | | | | SDDTLREDPELQACWK | | | | | | | | | | | | | | | | | | | | | |  |  |
|  | | | | | 1700.7715 | | | | | | 1700.7706 | | | | | | | 0 | | | | | | -1 | | | | | | | | 733 | | | | | | | | | | 746 | | | | | | | | | | | FMPEKGSAEYEELR | | | | | | | | | | | | | | | | | | | | | |  |  |

| Spot no. | | Homologous protein | | | | Accession no. | | | | | | | | Score | | | | | | | | | | | M.P. | | | | | | | | | | | | Cov. (%) | | | | | | | | | | | Blast score | | | | | | | | | | | | Mr (kDa) / pI | | | | | | | | | | | | | | | | | | | | | Ratio | | | | | | | | | | |  |  |  |  |
| --- | --- | --- | --- | --- | --- | --- | --- | --- | --- | --- | --- | --- | --- | --- | --- | --- | --- | --- | --- | --- | --- | --- | --- | --- | --- | --- | --- | --- | --- | --- | --- | --- | --- | --- | --- | --- | --- | --- | --- | --- | --- | --- | --- | --- | --- | --- | --- | --- | --- | --- | --- | --- | --- | --- | --- | --- | --- | --- | --- | --- | --- | --- | --- | --- | --- | --- | --- | --- | --- | --- | --- | --- | --- | --- | --- | --- | --- | --- | --- | --- | --- | --- | --- | --- | --- | --- | --- | --- | --- | --- | --- | --- | --- | --- | --- |
|  |  |  |  |  |  |  |  |  |  |  |  |  |  |  |  |  |  |  |  |  |  |  |  |  |  |  |  |  |  |  |  |  |  |  |  |  |  |  |  |  |  |  |  |  |  |  |  |  |  |  |  |  |  |  |  |  |  |  |  | Theo. | | | | | | | | | | Exp. | | | | | | | | | | |  |  |  |  |  |  |  |  |  |  |  |  |  |  |  |
| 17b | | lipoxygenase-2 | | | | Glyma13g42310.1 | | | | | | | | 135 | | | | | | | | | | | 7 | | | | | | | | | | | | 11 | | | | | | | | | | | 1720 | | | | | | | | | | | | 97.7/6.21 | | | | | | | | | |  | | | | | | | | | | |  | | | | | | | | | | |  |  |  |  |
|  | | Calc. Mass | | | | Observ. Mass | | | | | | | | Da | | | | | | | | | | | ppm | | | | | | | | | | | | start | | | | | | | | | | | end | | | | | | | | | | | | sequence | | | | | | | | | | | | | | | | | | | | | | | | | | | | | | | |  |  |  |  |
|  | | 1710.0039 | | | | 1710.001 | | | | | | | | 0 | | | | | | | | | | | -2 | | | | | | | | | | | | 69 | | | | | | | | | | | 85 | | | | | | | | | | | | SVALQLISATKPLANGK | | | | | | | | | | | | | | | | | | | | | | | | | | | | | | | |  |  |  |  |
|  | | 1541.8049 | | | | 1541.8042 | | | | | | | | 0 | | | | | | | | | | | 0 | | | | | | | | | | | | 141 | | | | | | | | | | | 154 | | | | | | | | | | | | SLTLEDVPNQGTIR | | | | | | | | | | | | | | | | | | | | | | | | | | | | | | | |  |  |  |  |
|  | | 1099.555 | | | | 1099.555 | | | | | | | | 0 | | | | | | | | | | | 0 | | | | | | | | | | | | 280 | | | | | | | | | | | 289 | | | | | | | | | | | | SSDFLAYGIK | | | | | | | | | | | | | | | | | | | | | | | | | | | | | | | |  |  |  |  |
|  | | 1343.669 | | | | 1343.6676 | | | | | | | | 0 | | | | | | | | | | | -1 | | | | | | | | | | | | 378 | | | | | | | | | | | 389 | | | | | | | | | | | | EMVAGVNPCVIR | | | | | | | | | | | | | | | | | | | | | | | | | | | | | | | |  |  |  |  |
|  | | 1551.7417 | | | | 1551.7404 | | | | | | | | 0 | | | | | | | | | | | -1 | | | | | | | | | | | | 398 | | | | | | | | | | | 411 | | | | | | | | | | | | SNLDPTIYGEQTSK | | | | | | | | | | | | | | | | | | | | | | | | | | | | | | | |  |  |  |  |
|  | | 1551.7417 | | | | 1551.7408 | | | | | | | | 0 | | | | | | | | | | | -1 | | | | | | | | | | | | 398 | | | | | | | | | | | 411 | | | | | | | | | | | | SNLDPTIYGEQTSK | | | | | | | | | | | | | | | | | | | | | | | | | | | | | | | |  |  |  |  |
|  | | 1416.7837 | | | | 1416.7834 | | | | | | | | 0 | | | | | | | | | | | 0 | | | | | | | | | | | | 617 | | | | | | | | | | | 630 | | | | | | | | | | | | GVAIKDPSAPHGLR | | | | | | | | | | | | | | | | | | | | | | | | | | | | | | | |  |  |  |  |
|  | | 1416.7837 | | | | 1416.784 | | | | | | | | 0 | | | | | | | | | | | 0 | | | | | | | | | | | | 617 | | | | | | | | | | | 630 | | | | | | | | | | | | GVAIKDPSAPHGLR | | | | | | | | | | | | | | | | | | | | | | | | | | | | | | | |  |  |  |  |
|  | | 1761.8858 | | | | 1761.8884 | | | | | | | | 0 | | | | | | | | | | | 1 | | | | | | | | | | | | 742 | | | | | | | | | | | 756 | | | | | | | | | | | | LLPEKGTPEYEEMVK | | | | | | | | | | | | | | | | | | | | | | | | | | | | | | | |  |  |  |  |
| Spot no. | | Homologous protein | | | | | | | | Accession no. | | | | | | | Score | | | | | | | | | | | | M.P. | | | | | | | | | | | | Cov. (%) | | | | | | | | | | | | Blast score | | | | | | | | | | | | | Mr (kDa) / pI | | | | | | | | | | | | | | | | Ratio | | | | | | | | | | |  |  |  |
|  |  |  |  |  |  |  |  |  |  |  |  |  |  |  |  |  |  |  |  |  |  |  |  |  |  |  |  |  |  |  |  |  |  |  |  |  |  |  |  |  |  |  |  |  |  |  |  |  |  |  |  |  |  |  |  |  |  |  |  |  |  |  |  |  |  | Theo. | | | | | | Exp. | | | | | | | | | |  |  |  |  |  |  |  |  |  |  |  |  |  |  |
| 18a | | beta-conglycinin alpha subunit | | | | | | | | Glyma20g28650.1 | | | | | | | 297 | | | | | | | | | | | | 14 | | | | | | | | | | | | 19 | | | | | | | | | | | | 783 | | | | | | | | | | | | | 70.8/5.12 | | | | | |  | | | | | | | | | |  | | | | | | | | | | |  |  |  |
|  | | Calc. Mass | | | | | | | | Observ. Mass | | | | | | | Da | | | | | | | | | | | | ppm | | | | | | | | | | | | start | | | | | | | | | | | end | | | | | | | | | | sequence | | | | | | | | | | | | | | | | | | | | | | | | | | | | | | |  |  |  |
|  | | 1292.6626 | | | | | | | | 1292.6616 | | | | | | | 0 | | | | | | | | | | | | -1 | | | | | | | | | | | | 192 | | | | | | | | | | | 202 | | | | | | | | | | NKNPFLFGSNR | | | | | | | | | | | | | | | | | | | | | | | | | | | | | | |  |  |  |
|  | | 1292.6626 | | | | | | | | 1292.6616 | | | | | | | 0 | | | | | | | | | | | | -1 | | | | | | | | | | | | 192 | | | | | | | | | | | 202 | | | | | | | | | | NKNPFLFGSNR | | | | | | | | | | | | | | | | | | | | | | | | | | | | | | |  |  |  |
|  | | 1292.6626 | | | | | | | | 1292.6629 | | | | | | | 0 | | | | | | | | | | | | 0 | | | | | | | | | | | | 192 | | | | | | | | | | | 202 | | | | | | | | | | NKNPFLFGSNR | | | | | | | | | | | | | | | | | | | | | | | | | | | | | | |  |  |  |
|  | | 1292.6626 | | | | | | | | 1292.6629 | | | | | | | 0 | | | | | | | | | | | | 0 | | | | | | | | | | | | 192 | | | | | | | | | | | 202 | | | | | | | | | | NKNPFLFGSNR | | | | | | | | | | | | | | | | | | | | | | | | | | | | | | |  |  |  |
|  | | 1050.5247 | | | | | | | | 1050.524 | | | | | | | 0 | | | | | | | | | | | | -1 | | | | | | | | | | | | 194 | | | | | | | | | | | 202 | | | | | | | | | | NPFLFGSNR | | | | | | | | | | | | | | | | | | | | | | | | | | | | | | |  |  |  |
|  | | 1050.5247 | | | | | | | | 1050.5257 | | | | | | | 0 | | | | | | | | | | | | 1 | | | | | | | | | | | | 194 | | | | | | | | | | | 202 | | | | | | | | | | NPFLFGSNR | | | | | | | | | | | | | | | | | | | | | | | | | | | | | | |  |  |  |
|  | | 783.4167 | | | | | | | | 783.4161 | | | | | | | 0 | | | | | | | | | | | | -1 | | | | | | | | | | | | 203 | | | | | | | | | | | 208 | | | | | | | | | | FETLFK | | | | | | | | | | | | | | | | | | | | | | | | | | | | | | |  |  |  |
|  | | 1401.7041 | | | | | | | | 1401.7048 | | | | | | | 0 | | | | | | | | | | | | 0 | | | | | | | | | | | | 203 | | | | | | | | | | | 213 | | | | | | | | | | FETLFKNQYGR | | | | | | | | | | | | | | | | | | | | | | | | | | | | | | |  |  |  |
|  | | 1388.716 | | | | | | | | 1388.7148 | | | | | | | 0 | | | | | | | | | | | | -1 | | | | | | | | | | | | 224 | | | | | | | | | | | | | | 234 | | | | | | | | | SPQLQNLRDYR | | | | | | | | | | | | | | | | | | | | | | | | | | | | |  |  |  |
|  | | 1388.716 | | | | | | | | 1388.7152 | | | | | | | 0 | | | | | | | | | | | | -1 | | | | | | | | | | | | 224 | | | | | | | | | | | | | | 234 | | | | | | | | | SPQLQNLRDYR | | | | | | | | | | | | | | | | | | | | | | | | | | | | |  |  |  |
|  | | 1388.716 | | | | | | | | 1388.7157 | | | | | | | 0 | | | | | | | | | | | | 0 | | | | | | | | | | | | 224 | | | | | | | | | | | | | | 234 | | | | | | | | | SPQLQNLRDYR | | | | | | | | | | | | | | | | | | | | | | | | | | | | |  |  |  |
|  | | 2151.0233 | | | | | | | | 2151.0194 | | | | | | | 0 | | | | | | | | | | | | -2 | | | | | | | | | | | | 287 | | | | | | | | | | | | | | 305 | | | | | | | | | VPSGTTYYVVNPDNNENLR | | | | | | | | | | | | | | | | | | | | | | | | | | | | |  |  |  |
|  | | 2151.0233 | | | | | | | | 2151.0194 | | | | | | | 0 | | | | | | | | | | | | -2 | | | | | | | | | | | | 287 | | | | | | | | | | | | | | 305 | | | | | | | | | VPSGTTYYVVNPDNNENLR | | | | | | | | | | | | | | | | | | | | | | | | | | | | |  |  |  |
|  | | 2151.0233 | | | | | | | | 2151.0217 | | | | | | | 0 | | | | | | | | | | | | -1 | | | | | | | | | | | | 287 | | | | | | | | | | | | | | 305 | | | | | | | | | VPSGTTYYVVNPDNNENLR | | | | | | | | | | | | | | | | | | | | | | | | | | | | |  |  |  |
|  | | 1390.866 | | | | | | | | 1390.8608 | | | | | | | 0 | | | | | | | | | | | | -4 | | | | | | | | | | | | 306 | | | | | | | | | | | | | | 318 | | | | | | | | | LITLAIPVNKPGR | | | | | | | | | | | | | | | | | | | | | | | | | | | | |  |  |  |
|  | | 1390.866 | | | | | | | | 1390.8628 | | | | | | | 0 | | | | | | | | | | | | -2 | | | | | | | | | | | | 306 | | | | | | | | | | | | | | 318 | | | | | | | | | LITLAIPVNKPGR | | | | | | | | | | | | | | | | | | | | | | | | | | | | |  |  |  |
|  | | 1390.866 | | | | | | | | 1390.8658 | | | | | | | 0 | | | | | | | | | | | | 0 | | | | | | | | | | | | 306 | | | | | | | | | | | | | | 318 | | | | | | | | | LITLAIPVNKPGR | | | | | | | | | | | | | | | | | | | | | | | | | | | | |  |  |  |
|  | | 1152.5662 | | | | | | | | 1152.567 | | | | | | | 0 | | | | | | | | | | | | 1 | | | | | | | | | | | | 340 | | | | | | | | | | | | | | 349 | | | | | | | | | NILEASYDTK | | | | | | | | | | | | | | | | | | | | | | | | | | | | |  |  |  |
|  | | 1533.8151 | | | | | | | | 1533.813 | | | | | | | 0 | | | | | | | | | | | | -1 | | | | | | | | | | | | 397 | | | | | | | | | | | | | | 409 | | | | | | | | | KTISSEDKPFNLR | | | | | | | | | | | | | | | | | | | | | | | | | | | | |  |  |  |
|  | | 1533.8151 | | | | | | | | 1533.814 | | | | | | | 0 | | | | | | | | | | | | -1 | | | | | | | | | | | | 397 | | | | | | | | | | | | | | 409 | | | | | | | | | KTISSEDKPFNLR | | | | | | | | | | | | | | | | | | | | | | | | | | | | |  |  |  |
|  | | 1533.8151 | | | | | | | | 1533.8143 | | | | | | | 0 | | | | | | | | | | | | -1 | | | | | | | | | | | | 397 | | | | | | | | | | | | | | 409 | | | | | | | | | KTISSEDKPFNLR | | | | | | | | | | | | | | | | | | | | | | | | | | | | |  |  |  |
|  | | 1533.8151 | | | | | | | | 1533.8149 | | | | | | | 0 | | | | | | | | | | | | 0 | | | | | | | | | | | | 397 | | | | | | | | | | | | | | 409 | | | | | | | | | KTISSEDKPFNLR | | | | | | | | | | | | | | | | | | | | | | | | | | | | |  |  |  |
|  | | 1533.8151 | | | | | | | | 1533.8152 | | | | | | | 0 | | | | | | | | | | | | 0 | | | | | | | | | | | | 397 | | | | | | | | | | | | | | 409 | | | | | | | | | KTISSEDKPFNLR | | | | | | | | | | | | | | | | | | | | | | | | | | | | |  |  |  |
|  | | 1533.8151 | | | | | | | | 1533.8155 | | | | | | | 0 | | | | | | | | | | | | 0 | | | | | | | | | | | | 397 | | | | | | | | | | | | | | 409 | | | | | | | | | KTISSEDKPFNLR | | | | | | | | | | | | | | | | | | | | | | | | | | | | |  |  |  |
|  | | 1405.7201 | | | | | | | | 1405.7167 | | | | | | | 0 | | | | | | | | | | | | -2 | | | | | | | | | | | | 398 | | | | | | | | | | | | | | 409 | | | | | | | | | TISSEDKPFNLR | | | | | | | | | | | | | | | | | | | | | | | | | | | | |  |  |  |
|  | | 1405.7201 | | | | | | | | 1405.7192 | | | | | | | 0 | | | | | | | | | | | | -1 | | | | | | | | | | | | 398 | | | | | | | | | | | | | | 409 | | | | | | | | | TISSEDKPFNLR | | | | | | | | | | | | | | | | | | | | | | | | | | | | |  |  |  |
|  | | 1405.7201 | | | | | | | | 1405.7196 | | | | | | | 0 | | | | | | | | | | | | 0 | | | | | | | | | | | | 398 | | | | | | | | | | | | | | 409 | | | | | | | | | TISSEDKPFNLR | | | | | | | | | | | | | | | | | | | | | | | | | | | | |  |  |  |
|  | | 1405.7201 | | | | | | | | 1405.7197 | | | | | | | 0 | | | | | | | | | | | | 0 | | | | | | | | | | | | 398 | | | | | | | | | | | | | | 409 | | | | | | | | | TISSEDKPFNLR | | | | | | | | | | | | | | | | | | | | | | | | | | | | |  |  |  |
|  | | 1405.7201 | | | | | | | | 1405.72 | | | | | | | 0 | | | | | | | | | | | | 0 | | | | | | | | | | | | 398 | | | | | | | | | | | | | | 409 | | | | | | | | | TISSEDKPFNLR | | | | | | | | | | | | | | | | | | | | | | | | | | | | |  |  |  |
|  | | 1307.7125 | | | | | | | | 1307.7121 | | | | | | | 0 | | | | | | | | | | | | 0 | | | | | | | | | | | | 419 | | | | | | | | | | | | | | 429 | | | | | | | | | LGKFFEITPEK | | | | | | | | | | | | | | | | | | | | | | | | | | | | |  |  |  |
|  | | 1009.512 | | | | | | | | 1009.5126 | | | | | | | 0 | | | | | | | | | | | | 1 | | | | | | | | | | | | 422 | | | | | | | | | | | | | | 429 | | | | | | | | | FFEITPEK | | | | | | | | | | | | | | | | | | | | | | | | | | | | |  |  |  |
|  | | 1617.8515 | | | | | | | | 1617.8494 | | | | | | | 0 | | | | | | | | | | | | -1 | | | | | | | | | | | | 422 | | | | | | | | | | | | | | 434 | | | | | | | | | FFEITPEKNPQLR | | | | | | | | | | | | | | | | | | | | | | | | | | | | |  |  |  |
|  | | 1617.8515 | | | | | | | | 1617.8506 | | | | | | | 0 | | | | | | | | | | | | -1 | | | | | | | | | | | | 422 | | | | | | | | | | | | | | 434 | | | | | | | | | FFEITPEKNPQLR | | | | | | | | | | | | | | | | | | | | | | | | | | | | |  |  |  |
|  | | 1617.8515 | | | | | | | | 1617.8506 | | | | | | | 0 | | | | | | | | | | | | -1 | | | | | | | | | | | | 422 | | | | | | | | | | | | | | 434 | | | | | | | | | FFEITPEKNPQLR | | | | | | | | | | | | | | | | | | | | | | | | | | | | |  |  |  |
|  | | 1617.8515 | | | | | | | | 1617.853 | | | | | | | 0 | | | | | | | | | | | | 1 | | | | | | | | | | | | 422 | | | | | | | | | | | | | | 434 | | | | | | | | | FFEITPEKNPQLR | | | | | | | | | | | | | | | | | | | | | | | | | | | | |  |  |  |
|  | | 1580.7583 | | | | | | | | 1580.7565 | | | | | | | 0 | | | | | | | | | | | | -1 | | | | | | | | | | | | 572 | | | | | | | | | | | | | | 584 | | | | | | | | | NQRESYFVDAQPK | | | | | | | | | | | | | | | | | | | | | | | | | | | | |  |  |  |
| Spot no. | | | Homologous protein | | | | | | | | Accession no. | | | | | | | Score | | | | | | | | | | | | M.P. | | | | | | | | | | | Cov. (%) | | | | | | | | | | Blast score | | | | | | | | | | | | | | | Mr (kDa) / pI | | | | | | | | | | | | | | | | | | | | | Ratio | | | | | | | | |
|  |  |  |  |  |  |  |  |  |  |  |  |  |  |  |  |  |  |  |  |  |  |  |  |  |  |  |  |  |  |  |  |  |  |  |  |  |  |  |  |  |  |  |  |  |  |  |  |  |  |  |  |  |  |  |  |  |  |  |  |  |  |  |  |  |  | Theo. | | | | | | | | Exp. | | | | | | | | | | | | |  |  |  |  |  |  |  |  |  |
| 18b | | | peroxisomal 3-ketoacyl-CoA thiolase | | | | | | | | Glyma10g24590.1 | | | | | | | 111 | | | | | | | | | | | | 3 | | | | | | | | | | | 11 | | | | | | | | | | 790 | | | | | | | | | | | | | | | 49.4/7.95 | | | | | | | |  | | | | | | | | | | | | |  | | | | | | | | |
|  | | | Calc. Mass | | | | | | | | Observ. Mass | | | | | | | Da | | | | | | | | | | | | ppm | | | | | | | | | | | start | | | | | | | | | | end | | | | | | | | | | | | | | | sequence | | | | | | | | | | | | | | | | | | | | | | | | | | | | | |
|  | | | 1275.7075 | | | | | | | | 1275.7068 | | | | | | | 0 | | | | | | | | | | | | 0 | | | | | | | | | | | 234 | | | | | | | | | | 244 | | | | | | | | | | | | | | | FKDEIVPVTTK | | | | | | | | | | | | | | | | | | | | | | | | | | | | | |
|  | | | 1275.7075 | | | | | | | | 1275.7078 | | | | | | | 0 | | | | | | | | | | | | 0 | | | | | | | | | | | 234 | | | | | | | | | | 244 | | | | | | | | | | | | | | | FKDEIVPVTTK | | | | | | | | | | | | | | | | | | | | | | | | | | | | | |
|  | | | 2209.0533 | | | | | | | | 2209.0534 | | | | | | | 0 | | | | | | | | | | | | 0 | | | | | | | | | | | 283 | | | | | | | | | | 305 | | | | | | | | | | | | | | | DGSTTAGNSSQVTDGASAVLLMK | | | | | | | | | | | | | | | | | | | | | | | | | | | | | |
|  | | | 1891.9687 | | | | | | | | 1891.9696 | | | | | | | 0 | | | | | | | | | | | | 0 | | | | | | | | | | | 382 | | | | | | | | | | 401 | | | | | | | | | | | | | | | INVNGGAMAIGHPLGSTGAR | | | | | | | | | | | | | | | | | | | | | | | | | | | | | |
|  | | | 1907.9636 | | | | | | | | 1907.9632 | | | | | | | 0 | | | | | | | | | | | | 0 | | | | | | | | | | | 382 | | | | | | | | | | 401 | | | | | | | | | | | | | | | INVNGGAMAIGHPLGSTGAR | | | | | | | | | | | | | | | | | | | | | | | | | | | | | |
| Spot no. | | | Homologous protein | | | | | | | | | Accession no. | | | | | | | Score | | | | | | | | | | | M.P. | | | | | | | | | | | Cov. (%) | | | | | | | | | Blast score | | | | | | | | | | | | | Mr (kDa) / pI | | | | | | | | | | | | | | | | | | | | | | | Ratio | | | | | | | | | |
|  |  |  |  |  |  |  |  |  |  |  |  |  |  |  |  |  |  |  |  |  |  |  |  |  |  |  |  |  |  |  |  |  |  |  |  |  |  |  |  |  |  |  |  |  |  |  |  |  |  |  |  |  |  |  |  |  |  |  |  |  |  |  | Theo. | | | | | | | | | | Exp. | | | | | | | | | | | | |  |  |  |  |  |  |  |  |  |  |
| 19 | | | lipoxygease L-4 | | | | | | | | | Glyma13g42330.1 | | | | | | | 120 | | | | | | | | | | | 4 | | | | | | | | | | | 7 | | | | | | | | | 1650 | | | | | | | | | | | | | 96.8/5.76 | | | | | | | | | |  | | | | | | | | | | | | |  | | | | | | | | | |
|  | | | Calc. Mass | | | | | | | | | Observ. Mass | | | | | | | Da | | | | | | | | | | | ppm | | | | | | | | | | | start | | | | | | | | | end | | | | | | | | | | | | | sequence | | | | | | | | | | | | | | | | | | | | | | | | | | | | | | | | |
|  | | | 2061.8803 | | | | | | | | | 2061.8814 | | | | | | | 0 | | | | | | | | | | | 1 | | | | | | | | | | | 197 | | | | | | | | | 214 | | | | | | | | | | | | | IYDYDVYNDLGNPDSGDK | | | | | | | | | | | | | | | | | | | | | | | | | | | | | | | | |
|  | | | 1637.8665 | | | | | | | | | 1637.8618 | | | | | | | 0 | | | | | | | | | | | -3 | | | | | | | | | | | 305 | | | | | | | | | 319 | | | | | | | | | | | | | LYEGGVTLPTNFLSK | | | | | | | | | | | | | | | | | | | | | | | | | | | | | | | | |
|  | | | 2089.0804 | | | | | | | | | 2089.0774 | | | | | | | 0 | | | | | | | | | | | -1 | | | | | | | | | | | 399 | | | | | | | | | 417 | | | | | | | | | | | | | EHLEPNLGGLTVEQAIQNK | | | | | | | | | | | | | | | | | | | | | | | | | | | | | | | | |
|  | | | 1141.6343 | | | | | | | | | 1141.6338 | | | | | | | 0 | | | | | | | | | | | 0 | | | | | | | | | | | 559 | | | | | | | | | 569 | | | | | | | | | | | | | ALVNADGIIEK | | | | | | | | | | | | | | | | | | | | | | | | | | | | | | | | |
| Spot no. | | | Homologous protein | | | | | | | | | Accession no. | | | | | | | Score | | | | | | | | | | M.P. | | | | | | | | | | | Cov. (%) | | | | | | | | | Blast score | | | | | | | | | | | | Mr (kDa) / pI | | | | | | | | | | | | | | | | | | | | | | Ratio | | | | | | | | | | | | |
|  |  |  |  |  |  |  |  |  |  |  |  |  |  |  |  |  |  |  |  |  |  |  |  |  |  |  |  |  |  |  |  |  |  |  |  |  |  |  |  |  |  |  |  |  |  |  |  |  |  |  |  |  |  |  |  |  |  |  |  |  | Theo. | | | | | | | | | | Exp. | | | | | | | | | | | |  |  |  |  |  |  |  |  |  |  |  |  |  |
| 20a | | | Phosphoglycerate kinase | | | | | | | | | Glyma08g17600.1 | | | | | | | 139 | | | | | | | | | | 4 | | | | | | | | | | | 15 | | | | | | | | | 735 | | | | | | | | | | | | 42.6/6.28 | | | | | | | | | |  | | | | | | | | | | | |  | | | | | | | | | | | | |
|  | | | Calc. Mass | | | | | | | | | Observ. Mass | | | | | | | Da | | | | | | | | | | ppm | | | | | | | | | | start | | | | | | | | | | end | | | | | | | | | | | | sequence | | | | | | | | | | | | | | | | | | | | | | | | | | | | | | | | | | |
|  | | | 2040.9964 | | | | | | | | | 2040.9934 | | | | | | | 0 | | | | | | | | | | -1 | | | | | | | | | | 24 | | | | | | | | | | 41 | | | | | | | | | | | | VDLNVPLDDNLNITDDTR | | | | | | | | | | | | | | | | | | | | | | | | | | | | | | | | | | |
|  | | | 1932.9694 | | | | | | | | | 1932.9718 | | | | | | | 0 | | | | | | | | | | 1 | | | | | | | | | | 138 | | | | | | | | | | 155 | | | | | | | | | | | | LASLADLYVNDAFGTAHR | | | | | | | | | | | | | | | | | | | | | | | | | | | | | | | | | | |
|  | | | 1403.7296 | | | | | | | | | 1403.725 | | | | | | | 0 | | | | | | | | | | -3 | | | | | | | | | | 179 | | | | | | | | | | 191 | | | | | | | | | | | | ELDYLVGAVSNPK | | | | | | | | | | | | | | | | | | | | | | | | | | | | | | | | | | |
|  | | | 1538.892 | | | | | | | | | 1538.89 | | | | | | | 0 | | | | | | | | | | -1 | | | | | | | | | | 262 | | | | | | | | | | 276 | | | | | | | | | | | | GVSLLLPTDVVIADK | | | | | | | | | | | | | | | | | | | | | | | | | | | | | | | | | | |
| Spot no. | | | | Homologous protein | | | | | | | | | Accession no. | | | | | | | Score | | | | | | | | | | | M.P. | | | | | | | | | | | | Cov. (%) | | | | | | | | | | | | | Blast score | | | | | | | | | | | Mr (kDa) / pI | | | | | | | | | | | | | | | | | | | | | Ratio | | | | | | | |
|  |  |  |  |  |  |  |  |  |  |  |  |  |  |  |  |  |  |  |  |  |  |  |  |  |  |  |  |  |  |  |  |  |  |  |  |  |  |  |  |  |  |  |  |  |  |  |  |  |  |  |  |  |  |  |  |  |  |  |  |  |  |  |  |  |  |  | Theo. | | | | | | | | | Exp. | | | | | | | | | | | |  |  |  |  |  |  |  |  |
| 20b | | | | lipoxygease L-4 | | | | | | | | | Glyma13g42330.1 | | | | | | | 110 | | | | | | | | | | | 4 | | | | | | | | | | | | 6 | | | | | | | | | | | | | 1650 | | | | | | | | | | | 96.8/5.76 | | | | | | | | |  | | | | | | | | | | | |  | | | | | | | |
|  | | | | Calc. Mass | | | | | | | | | Observ. Mass | | | | | | | Da | | | | | | | | | | | ppm | | | | | | | | | | | | start | | | | | | | | | | | | | end | | | | | | | | | | | sequence | | | | | | | | | | | | | | | | | | | | | | | | | | | | |
|  | | | | 2061.8803 | | | | | | | | | 2061.8734 | | | | | | | 0 | | | | | | | | | | | -3 | | | | | | | | | | | | 197 | | | | | | | | | | | | | 214 | | | | | | | | | | | IYDYDVYNDLGNPDSGDK | | | | | | | | | | | | | | | | | | | | | | | | | | | | |
|  | | | | 1637.8665 | | | | | | | | | 1637.863 | | | | | | | 0 | | | | | | | | | | | -2 | | | | | | | | | | | | 305 | | | | | | | | | | | | | 319 | | | | | | | | | | | LYEGGVTLPTNFLSK | | | | | | | | | | | | | | | | | | | | | | | | | | | | |
|  | | | | 1704.8141 | | | | | | | | | 1704.814 | | | | | | | 0 | | | | | | | | | | | 0 | | | | | | | | | | | | 384 | | | | | | | | | | | | | 398 | | | | | | | | | | | LDTQAYGDHTCIIAK | | | | | | | | | | | | | | | | | | | | | | | | | | | | |
|  | | | | 1141.6343 | | | | | | | | | 1141.6332 | | | | | | | 0 | | | | | | | | | | | -1 | | | | | | | | | | | | 559 | | | | | | | | | | | | | 569 | | | | | | | | | | | ALVNADGIIEK | | | | | | | | | | | | | | | | | | | | | | | | | | | | |
| Spot no. | | | | Homologous protein | | | | | | | | | Accession no. | | | | | | | Score | | | | | | | | | | | | M.P. | | | | | | | | | | | Cov. (%) | | | | | | | | | | | | | Blast score | | | | | | | | | | | Mr (kDa) / pI | | | | | | | | | | | | | | | | | | | | | Ratio | | | | | | | |
|  |  |  |  |  |  |  |  |  |  |  |  |  |  |  |  |  |  |  |  |  |  |  |  |  |  |  |  |  |  |  |  |  |  |  |  |  |  |  |  |  |  |  |  |  |  |  |  |  |  |  |  |  |  |  |  |  |  |  |  |  |  |  |  |  |  |  | Theo. | | | | | | | | | | Exp. | | | | | | | | | | |  |  |  |  |  |  |  |  |
| 21 | | | | Chain A, Crystal Structure Of Glycinin A3b4 Subunit Homohexamer | | | | | | | | | Glyma13g18450.1 | | | | | | | 130 | | | | | | | | | | | | 4 | | | | | | | | | | | 16 | | | | | | | | | | | | | 628 | | | | | | | | | | | 47.0/5.27 | | | | | | | | | |  | | | | | | | | | | |  | | | | | | | |
|  | | | | | Calc. Mass | | Observ. Mass | | | | | | | | Da | | | | | | | | | | | ppm | | | | | | | | | | | | start | | | | | | | | | end | | | | | | | | | | | | sequence | | | | | | | | | | | | | | | | | | | | | | | | | | | | | | | | | | | | |
|  | | | | | 2082.9541 | | 2082.9508 | | | | | | | | 0 | | | | | | | | | | | -2 | | | | | | | | | | | | 2 | | | | | | | | | 18 | | | | | | | | | | | | FNECQLNNLNALEPDHR | | | | | | | | | | | | | | | | | | | | | | | | | | | | | | | | | | | | |
|  | | | | | 2082.9541 | | 2082.952 | | | | | | | | 0 | | | | | | | | | | | -1 | | | | | | | | | | | | 2 | | | | | | | | | 18 | | | | | | | | | | | | FNECQLNNLNALEPDHR | | | | | | | | | | | | | | | | | | | | | | | | | | | | | | | | | | | | |
|  | | | | | 2082.9541 | | 2082.952 | | | | | | | | 0 | | | | | | | | | | | -1 | | | | | | | | | | | | 2 | | | | | | | | | 18 | | | | | | | | | | | | FNECQLNNLNALEPDHR | | | | | | | | | | | | | | | | | | | | | | | | | | | | | | | | | | | | |
|  | | | | | 2082.9541 | | 2082.9544 | | | | | | | | 0 | | | | | | | | | | | 0 | | | | | | | | | | | | 2 | | | | | | | | | 18 | | | | | | | | | | | | FNECQLNNLNALEPDHR | | | | | | | | | | | | | | | | | | | | | | | | | | | | | | | | | | | | |
|  | | | | | 2954.408 | | 2954.4016 | | | | | | | | 0 | | | | | | | | | | | -2 | | | | | | | | | | | | 19 | | | | | | | | | 45 | | | | | | | | | | | | VESEGGLIETWNSQHPELQCAGVTVSK | | | | | | | | | | | | | | | | | | | | | | | | | | | | | | | | | | | | |
|  | | | | | 2954.408 | | 2954.4037 | | | | | | | | 0 | | | | | | | | | | | -1 | | | | | | | | | | | | 19 | | | | | | | | | 45 | | | | | | | | | | | | VESEGGLIETWNSQHPELQCAGVTVSK | | | | | | | | | | | | | | | | | | | | | | | | | | | | | | | | | | | | |
|  | | | | | 3110.5091 | | 3110.5057 | | | | | | | | 0 | | | | | | | | | | | -1 | | | | | | | | | | | | 19 | | | | | | | | | 46 | | | | | | | | | | | | VESEGGLIETWNSQHPELQCAGVTVSKR | | | | | | | | | | | | | | | | | | | | | | | | | | | | | | | | | | | | |
|  | | | | | 2356.2249 | | 2356.2214 | | | | | | | | 0 | | | | | | | | | | | -2 | | | | | | | | | | | | 51 | | | | | | | | | 71 | | | | | | | | | | | | NGLHLPSYSPYPQMIIVVQGK | | | | | | | | | | | | | | | | | | | | | | | | | | | | | | | | | | | | |
|  | | | | | 2356.2249 | | 2356.2238 | | | | | | | | 0 | | | | | | | | | | | 0 | | | | | | | | | | | | 51 | | | | | | | | | 71 | | | | | | | | | | | | NGLHLPSYSPYPQMIIVVQGK | | | | | | | | | | | | | | | | | | | | | | | | | | | | | | | | | | | | |
| Spot no. | | Homologous protein | | | | | | | Accession no. | | | | | | | | | | | | | Score | | | | | | | | | | | | | M.P. | | | | | | | | | | Cov. (%) | | | | | | | | | | | | | Blast score | | | | | | | | | | | Mr (kDa) / pI | | | | | | | | | | | | | | | | | | | | | | | Ratio | | | |
|  |  |  |  |  |  |  |  |  |  |  |  |  |  |  |  |  |  |  |  |  |  |  |  |  |  |  |  |  |  |  |  |  |  |  |  |  |  |  |  |  |  |  |  |  |  |  |  |  |  |  |  |  |  |  |  |  |  |  |  |  |  |  |  |  |  |  |  |  | Theo. | | | | | | | | | | | | Exp. | | | | | | | | | | |  |  |  |  |
| 22 | | glycinin G2 precursor | | | | | | | Glyma03g32020.1 | | | | | | | | | | | | | 320 | | | | | | | | | | | | | 2 | | | | | | | | | | 4 | | | | | | | | | | | | | 799 | | | | | | | | | | | 55.1/5.46 | | | | | | | | | | | |  | | | | | | | | | | |  | | | |
|  | | Calc. Mass | | | | | | | Observ. Mass | | | | | | | | | | | | | Da | | | | | | | | | | | | | ppm | | | | | | | | | | start | | | | | | | | | | | | | end | | | | | | | | | | | sequence | | | | | | | | | | | | | | | | | | | | | | | | | | |
|  | | 993.5243 | | | | | | | 993.5244 | | | | | | | | | | | | | 0 | | | | | | | | | | | | | 0 | | | | | | | | | | 347 | | | | | | | | | | | | | 355 | | | | | | | | | | | LSAQYGSLR | | | | | | | | | | | | | | | | | | | | | | | | | | |
|  | | 993.5243 | | | | | | | 993.5246 | | | | | | | | | | | | | 0 | | | | | | | | | | | | | 0 | | | | | | | | | | 347 | | | | | | | | | | | | | 355 | | | | | | | | | | | LSAQYGSLR | | | | | | | | | | | | | | | | | | | | | | | | | | |
|  | | 993.5243 | | | | | | | 993.5256 | | | | | | | | | | | | | 0 | | | | | | | | | | | | | 1 | | | | | | | | | | 347 | | | | | | | | | | | | | 355 | | | | | | | | | | | LSAQYGSLR | | | | | | | | | | | | | | | | | | | | | | | | | | |
|  | | 993.5243 | | | | | | | 993.5266 | | | | | | | | | | | | | 0 | | | | | | | | | | | | | 2 | | | | | | | | | | 347 | | | | | | | | | | | | | 355 | | | | | | | | | | | LSAQYGSLR | | | | | | | | | | | | | | | | | | | | | | | | | | |
|  | | 1449.6412 | | | | | | | 1449.6402 | | | | | | | | | | | | | 0 | | | | | | | | | | | | | -1 | | | | | | | | | | 414 | | | | | | | | | | | | | 425 | | | | | | | | | | | SQSDNFEYVSFK | | | | | | | | | | | | | | | | | | | | | | | | | | |
|  | | 1449.6412 | | | | | | | 1449.6404 | | | | | | | | | | | | | 0 | | | | | | | | | | | | | -1 | | | | | | | | | | 414 | | | | | | | | | | | | | 425 | | | | | | | | | | | SQSDNFEYVSFK | | | | | | | | | | | | | | | | | | | | | | | | | | |
|  | | 1449.6412 | | | | | | | 1449.6404 | | | | | | | | | | | | | 0 | | | | | | | | | | | | | -1 | | | | | | | | | | 414 | | | | | | | | | | | | | 425 | | | | | | | | | | | SQSDNFEYVSFK | | | | | | | | | | | | | | | | | | | | | | | | | | |
|  | | 1449.6412 | | | | | | | 1449.6404 | | | | | | | | | | | | | 0 | | | | | | | | | | | | | -1 | | | | | | | | | | 414 | | | | | | | | | | | | | 425 | | | | | | | | | | | SQSDNFEYVSFK | | | | | | | | | | | | | | | | | | | | | | | | | | |
|  | | 1449.6412 | | | | | | | 1449.6406 | | | | | | | | | | | | | 0 | | | | | | | | | | | | | 0 | | | | | | | | | | 414 | | | | | | | | | | | | | 425 | | | | | | | | | | | SQSDNFEYVSFK | | | | | | | | | | | | | | | | | | | | | | | | | | |
|  | | 1449.6412 | | | | | | | 1449.6406 | | | | | | | | | | | | | 0 | | | | | | | | | | | | | 0 | | | | | | | | | | 414 | | | | | | | | | | | | | 425 | | | | | | | | | | | SQSDNFEYVSFK | | | | | | | | | | | | | | | | | | | | | | | | | | |
|  | | 1449.6412 | | | | | | | 1449.642 | | | | | | | | | | | | | 0 | | | | | | | | | | | | | 1 | | | | | | | | | | 414 | | | | | | | | | | | | | 425 | | | | | | | | | | | SQSDNFEYVSFK | | | | | | | | | | | | | | | | | | | | | | | | | | |
|  | | 1449.6412 | | | | | | | 1449.643 | | | | | | | | | | | | | 0 | | | | | | | | | | | | | 1 | | | | | | | | | | 414 | | | | | | | | | | | | | 425 | | | | | | | | | | | SQSDNFEYVSFK | | | | | | | | | | | | | | | | | | | | | | | | | | |
|  | | 1449.6412 | | | | | | | 1449.6448 | | | | | | | | | | | | | 0 | | | | | | | | | | | | | 2 | | | | | | | | | | 414 | | | | | | | | | | | | | 425 | | | | | | | | | | | SQSDNFEYVSFK | | | | | | | | | | | | | | | | | | | | | | | | | | |
| Spot no. | | Homologous protein | | | | | | | Accession no. | | | | | | | | | | | | | | | | Score | | | | | | | | | | | | M.P. | | | | | | | | | | Cov. (%) | | | | | | | | | | | Blast score | | | | | | | | | | | Mr (kDa) / pI | | | | | | | | | | | | | | | | | | | | | | Ratio | | | | |
|  |  |  |  |  |  |  |  |  |  |  |  |  |  |  |  |  |  |  |  |  |  |  |  |  |  |  |  |  |  |  |  |  |  |  |  |  |  |  |  |  |  |  |  |  |  |  |  |  |  |  |  |  |  |  |  |  |  |  |  |  |  |  |  |  |  |  |  |  | Theo. | | | | | | | | | | | Exp. | | | | | | | | | | |  |  |  |  |  |
| 24 | | glycinin G2 precursor | | | | | | | Glyma03g32020.1 | | | | | | | | | | | | | | | | 101 | | | | | | | | | | | | 3 | | | | | | | | | | 6 | | | | | | | | | | | 799 | | | | | | | | | | | 55.1/5.46 | | | | | | | | | | |  | | | | | | | | | | |  | | | | |
|  | | Calc. Mass | | | | | | | Observ. Mass | | | | | | | | | | | | | | Da | | | | | | | | | | | | | ppm | | | | | | | | | | start | | | | | | | | | | | | end | | | | | | | | | | | sequence | | | | | | | | | | | | | | | | | | | | | | | | | | |
|  | | 1585.7776 | | | | | | | 1585.7756 | | | | | | | | | | | | | | 0 | | | | | | | | | | | | | -1 | | | | | | | | | | 179 | | | | | | | | | | | | 191 | | | | | | | | | | | FYLAGNQEQEFLK | | | | | | | | | | | | | | | | | | | | | | | | | | |
|  | | 1585.7776 | | | | | | | 1585.7762 | | | | | | | | | | | | | | 0 | | | | | | | | | | | | | -1 | | | | | | | | | | 179 | | | | | | | | | | | | 191 | | | | | | | | | | | FYLAGNQEQEFLK | | | | | | | | | | | | | | | | | | | | | | | | | | |
|  | | 993.5243 | | | | | | | 993.524 | | | | | | | | | | | | | | 0 | | | | | | | | | | | | | 0 | | | | | | | | | | 347 | | | | | | | | | | | | 355 | | | | | | | | | | | LSAQYGSLR | | | | | | | | | | | | | | | | | | | | | | | | | | |
|  | | 1449.6412 | | | | | | | 1449.6374 | | | | | | | | | | | | | | 0 | | | | | | | | | | | | | -3 | | | | | | | | | | 414 | | | | | | | | | | | | 425 | | | | | | | | | | | SQSDNFEYVSFK | | | | | | | | | | | | | | | | | | | | | | | | | | |
|  | | 1449.6412 | | | | | | | 1449.64 | | | | | | | | | | | | | | 0 | | | | | | | | | | | | | -1 | | | | | | | | | | 414 | | | | | | | | | | | | 425 | | | | | | | | | | | SQSDNFEYVSFK | | | | | | | | | | | | | | | | | | | | | | | | | | |
| Spot no. | | Homologous protein | | | | | | | | Accession no. | | | | | | | | | | | | | | Score | | | | | | | | | | M.P. | | | | | | | | | | | | | Cov. (%) | | | | | | | | | | | Blast score | | | | | | | | | | | Mr (kDa) / pI | | | | | | | | | | | | | | | | | | | | | Ratio | | | | | |
|  |  |  |  |  |  |  |  |  |  |  |  |  |  |  |  |  |  |  |  |  |  |  |  |  |  |  |  |  |  |  |  |  |  |  |  |  |  |  |  |  |  |  |  |  |  |  |  |  |  |  |  |  |  |  |  |  |  |  |  |  |  |  |  |  |  |  |  |  | Theo. | | | | | | | | | | Exp. | | | | | | | | | | |  |  |  |  |  |  |
| 25a | | Beta-conglycinin, beta chain | | | | | | | | Glyma20g28640.1 | | | | | | | | | | | | | | 1205 | | | | | | | | | | 22 | | | | | | | | | | | | | 45 | | | | | | | | | | | 801 | | | | | | | | | | | 50.7/5.88 | | | | | | | | | |  | | | | | | | | | | |  | | | | | |
|  | | Calc. Mass | | | | | | | | Observ. Mass | | | | | | | | | | | | | | Da | | | | | | | | | | ppm | | | | | | | | | | | | | start | | | | | | | | | | | end | | | | | | | | | | | sequence | | | | | | | | | | | | | | | | | | | | | | | | | | |
|  | | 1550.7477 | | | | | | | | 1550.7472 | | | | | | | | | | | | | | 0 | | | | | | | | | | 0 | | | | | | | | | | | | | 26 | | | | | | | | | | | 37 | | | | | | | | | | | VREDENNPFYLR | | | | | | | | | | | | | | | | | | | | | | | | | | |
|  | | 1727.7863 | | | | | | | | 1727.7844 | | | | | | | | | | | | | | 0 | | | | | | | | | | -1 | | | | | | | | | | | | | 38 | | | | | | | | | | | 52 | | | | | | | | | | | SSNSFQTLFENQNGR | | | | | | | | | | | | | | | | | | | | | | | | | | |
|  | | 1727.7863 | | | | | | | | 1727.7846 | | | | | | | | | | | | | | 0 | | | | | | | | | | -1 | | | | | | | | | | | | | 38 | | | | | | | | | | | 52 | | | | | | | | | | | SSNSFQTLFENQNGR | | | | | | | | | | | | | | | | | | | | | | | | | | |
|  | | 1727.7863 | | | | | | | | 1727.7852 | | | | | | | | | | | | | | 0 | | | | | | | | | | -1 | | | | | | | | | | | | | 38 | | | | | | | | | | | 52 | | | | | | | | | | | SSNSFQTLFENQNGR | | | | | | | | | | | | | | | | | | | | | | | | | | |
|  | | 1727.7863 | | | | | | | | 1727.7862 | | | | | | | | | | | | | | 0 | | | | | | | | | | 0 | | | | | | | | | | | | | 38 | | | | | | | | | | | 52 | | | | | | | | | | | SSNSFQTLFENQNGR | | | | | | | | | | | | | | | | | | | | | | | | | | |
|  | | 1727.7863 | | | | | | | | 1727.7866 | | | | | | | | | | | | | | 0 | | | | | | | | | | 0 | | | | | | | | | | | | | 38 | | | | | | | | | | | 52 | | | | | | | | | | | SSNSFQTLFENQNGR | | | | | | | | | | | | | | | | | | | | | | | | | | |
|  | | 1111.6098 | | | | | | | | 1111.6086 | | | | | | | | | | | | | | 0 | | | | | | | | | | -1 | | | | | | | | | | | | | 62 | | | | | | | | | | | 70 | | | | | | | | | | | RSPQLENLR | | | | | | | | | | | | | | | | | | | | | | | | | | |
|  | | 955.5087 | | | | | | | | 955.5078 | | | | | | | | | | | | | | 0 | | | | | | | | | | -1 | | | | | | | | | | | | | 63 | | | | | | | | | | | 70 | | | | | | | | | | | SPQLENLR | | | | | | | | | | | | | | | | | | | | | | | | | | |
|  | | 1389.7 | | | | | | | | 1389.6982 | | | | | | | | | | | | | | 0 | | | | | | | | | | -1 | | | | | | | | | | | | | 63 | | | | | | | | | | | 73 | | | | | | | | | | | SPQLENLRDYR | | | | | | | | | | | | | | | | | | | | | | | | | | |
|  | | 1389.7 | | | | | | | | 1389.6986 | | | | | | | | | | | | | | 0 | | | | | | | | | | -1 | | | | | | | | | | | | | 63 | | | | | | | | | | | 73 | | | | | | | | | | | SPQLENLRDYR | | | | | | | | | | | | | | | | | | | | | | | | | | |
|  | | 1389.7 | | | | | | | | 1389.6988 | | | | | | | | | | | | | | 0 | | | | | | | | | | -1 | | | | | | | | | | | | | 63 | | | | | | | | | | | 73 | | | | | | | | | | | SPQLENLRDYR | | | | | | | | | | | | | | | | | | | | | | | | | | |
|  | | 1389.7 | | | | | | | | 1389.6997 | | | | | | | | | | | | | | 0 | | | | | | | | | | 0 | | | | | | | | | | | | | 63 | | | | | | | | | | | 73 | | | | | | | | | | | SPQLENLRDYR | | | | | | | | | | | | | | | | | | | | | | | | | | |
|  | | 1389.7 | | | | | | | | 1389.7006 | | | | | | | | | | | | | | 0 | | | | | | | | | | 0 | | | | | | | | | | | | | 63 | | | | | | | | | | | 73 | | | | | | | | | | | SPQLENLRDYR | | | | | | | | | | | | | | | | | | | | | | | | | | |
|  | | 1242.6568 | | | | | | | | 1242.6554 | | | | | | | | | | | | | | 0 | | | | | | | | | | -1 | | | | | | | | | | | | | 103 | | | | | | | | | | | 113 | | | | | | | | | | | AILTLVNNDDR | | | | | | | | | | | | | | | | | | | | | | | | | | |
|  | | 1242.6568 | | | | | | | | 1242.6556 | | | | | | | | | | | | | | 0 | | | | | | | | | | -1 | | | | | | | | | | | | | 103 | | | | | | | | | | | 113 | | | | | | | | | | | AILTLVNNDDR | | | | | | | | | | | | | | | | | | | | | | | | | | |
|  | | 1242.6568 | | | | | | | | 1242.6564 | | | | | | | | | | | | | | 0 | | | | | | | | | | 0 | | | | | | | | | | | | | 103 | | | | | | | | | | | 113 | | | | | | | | | | | AILTLVNNDDR | | | | | | | | | | | | | | | | | | | | | | | | | | |
|  | | 2596.263 | | | | | | | | 2596.2621 | | | | | | | | | | | | | | 0 | | | | | | | | | | 0 | | | | | | | | | | | | | 103 | | | | | | | | | | | 125 | | | | | | | | | | | AILTLVNNDDRDSYNLHPGDAQR | | | | | | | | | | | | | | | | | | | | | | | | | | |
|  | | 2596.263 | | | | | | | | 2596.2628 | | | | | | | | | | | | | | 0 | | | | | | | | | | 0 | | | | | | | | | | | | | 103 | | | | | | | | | | | 125 | | | | | | | | | | | AILTLVNNDDRDSYNLHPGDAQR | | | | | | | | | | | | | | | | | | | | | | | | | | |
|  | | 2180.1014 | | | | | | | | 2180.0997 | | | | | | | | | | | | | | 0 | | | | | | | | | | -1 | | | | | | | | | | | | | 126 | | | | | | | | | | | 144 | | | | | | | | | | | IPAGTTYYLVNPHDHQNLK | | | | | | | | | | | | | | | | | | | | | | | | | | |
|  | | 2180.1014 | | | | | | | | 2180.0997 | | | | | | | | | | | | | | 0 | | | | | | | | | | -1 | | | | | | | | | | | | | 126 | | | | | | | | | | | 144 | | | | | | | | | | | IPAGTTYYLVNPHDHQNLK | | | | | | | | | | | | | | | | | | | | | | | | | | |
|  | | 2180.1014 | | | | | | | | 2180.0998 | | | | | | | | | | | | | | 0 | | | | | | | | | | -1 | | | | | | | | | | | | | 126 | | | | | | | | | | | 144 | | | | | | | | | | | IPAGTTYYLVNPHDHQNLK | | | | | | | | | | | | | | | | | | | | | | | | | | |
|  | | 2180.1014 | | | | | | | | 2180.1004 | | | | | | | | | | | | | | 0 | | | | | | | | | | 0 | | | | | | | | | | | | | 126 | | | | | | | | | | | 144 | | | | | | | | | | | IPAGTTYYLVNPHDHQNLK | | | | | | | | | | | | | | | | | | | | | | | | | | |
|  | | 2180.1014 | | | | | | | | 2180.1007 | | | | | | | | | | | | | | 0 | | | | | | | | | | 0 | | | | | | | | | | | | | 126 | | | | | | | | | | | 144 | | | | | | | | | | | IPAGTTYYLVNPHDHQNLK | | | | | | | | | | | | | | | | | | | | | | | | | | |
|  | | 2180.1014 | | | | | | | | 2180.1016 | | | | | | | | | | | | | | 0 | | | | | | | | | | 0 | | | | | | | | | | | | | 126 | | | | | | | | | | | 144 | | | | | | | | | | | IPAGTTYYLVNPHDHQNLK | | | | | | | | | | | | | | | | | | | | | | | | | | |
|  | | 1200.5986 | | | | | | | | 1200.5986 | | | | | | | | | | | | | | 0 | | | | | | | | | | 0 | | | | | | | | | | | | | 195 | | | | | | | | | | | 204 | | | | | | | | | | | VLLGEEEEQR | | | | | | | | | | | | | | | | | | | | | | | | | | |
|  | | 1228.6663 | | | | | | | | 1228.6644 | | | | | | | | | | | | | | 0 | | | | | | | | | | -2 | | | | | | | | | | | | | 205 | | | | | | | | | | | 215 | | | | | | | | | | | QQEGVIVELSK | | | | | | | | | | | | | | | | | | | | | | | | | | |
|  | | 1754.9526 | | | | | | | | 1754.9484 | | | | | | | | | | | | | | 0 | | | | | | | | | | -2 | | | | | | | | | | | | | 205 | | | | | | | | | | | 219 | | | | | | | | | | | QQEGVIVELSKEQIR | | | | | | | | | | | | | | | | | | | | | | | | | | |
|  | | 1534.7627 | | | | | | | | 1534.7608 | | | | | | | | | | | | | | 0 | | | | | | | | | | -1 | | | | | | | | | | | | | 231 | | | | | | | | | | | 243 | | | | | | | | | | | KTISSEDEPFNLR | | | | | | | | | | | | | | | | | | | | | | | | | | |
|  | | 1534.7627 | | | | | | | | 1534.7608 | | | | | | | | | | | | | | 0 | | | | | | | | | | -1 | | | | | | | | | | | | | 231 | | | | | | | | | | | 243 | | | | | | | | | | | KTISSEDEPFNLR | | | | | | | | | | | | | | | | | | | | | | | | | | |
|  | | 1534.7627 | | | | | | | | 1534.761 | | | | | | | | | | | | | | 0 | | | | | | | | | | -1 | | | | | | | | | | | | | 231 | | | | | | | | | | | 243 | | | | | | | | | | | KTISSEDEPFNLR | | | | | | | | | | | | | | | | | | | | | | | | | | |
|  | | 1534.7627 | | | | | | | | 1534.7623 | | | | | | | | | | | | | | 0 | | | | | | | | | | 0 | | | | | | | | | | | | | 231 | | | | | | | | | | | 243 | | | | | | | | | | | KTISSEDEPFNLR | | | | | | | | | | | | | | | | | | | | | | | | | | |
|  | | 1534.7627 | | | | | | | | 1534.7623 | | | | | | | | | | | | | | 0 | | | | | | | | | | 0 | | | | | | | | | | | | | 231 | | | | | | | | | | | 243 | | | | | | | | | | | KTISSEDEPFNLR | | | | | | | | | | | | | | | | | | | | | | | | | | |
|  | | 1534.7627 | | | | | | | | 1534.7626 | | | | | | | | | | | | | | 0 | | | | | | | | | | 0 | | | | | | | | | | | | | 231 | | | | | | | | | | | 243 | | | | | | | | | | | KTISSEDEPFNLR | | | | | | | | | | | | | | | | | | | | | | | | | | |
|  | | 1534.7627 | | | | | | | | 1534.7629 | | | | | | | | | | | | | | 0 | | | | | | | | | | 0 | | | | | | | | | | | | | 231 | | | | | | | | | | | 243 | | | | | | | | | | | KTISSEDEPFNLR | | | | | | | | | | | | | | | | | | | | | | | | | | |
|  | | 1534.7627 | | | | | | | | 1534.7638 | | | | | | | | | | | | | | 0 | | | | | | | | | | 1 | | | | | | | | | | | | | 231 | | | | | | | | | | | 243 | | | | | | | | | | | KTISSEDEPFNLR | | | | | | | | | | | | | | | | | | | | | | | | | | |
|  | | 1534.7627 | | | | | | | | 1534.7638 | | | | | | | | | | | | | | 0 | | | | | | | | | | 1 | | | | | | | | | | | | | 231 | | | | | | | | | | | 243 | | | | | | | | | | | KTISSEDEPFNLR | | | | | | | | | | | | | | | | | | | | | | | | | | |
|  | | 1406.6678 | | | | | | | | 1406.6656 | | | | | | | | | | | | | | 0 | | | | | | | | | | -1 | | | | | | | | | | | | | 232 | | | | | | | | | | | 243 | | | | | | | | | | | TISSEDEPFNLR | | | | | | | | | | | | | | | | | | | | | | | | | | |
|  | | 1406.6678 | | | | | | | | 1406.6664 | | | | | | | | | | | | | | 0 | | | | | | | | | | -1 | | | | | | | | | | | | | 232 | | | | | | | | | | | 243 | | | | | | | | | | | TISSEDEPFNLR | | | | | | | | | | | | | | | | | | | | | | | | | | |
|  | | 1406.6678 | | | | | | | | 1406.6664 | | | | | | | | | | | | | | 0 | | | | | | | | | | -1 | | | | | | | | | | | | | 232 | | | | | | | | | | | 243 | | | | | | | | | | | TISSEDEPFNLR | | | | | | | | | | | | | | | | | | | | | | | | | | |
|  | | 1406.6678 | | | | | | | | 1406.6666 | | | | | | | | | | | | | | 0 | | | | | | | | | | -1 | | | | | | | | | | | | | 232 | | | | | | | | | | | 243 | | | | | | | | | | | TISSEDEPFNLR | | | | | | | | | | | | | | | | | | | | | | | | | | |
|  | | 1406.6678 | | | | | | | | 1406.6676 | | | | | | | | | | | | | | 0 | | | | | | | | | | 0 | | | | | | | | | | | | | 232 | | | | | | | | | | | 243 | | | | | | | | | | | TISSEDEPFNLR | | | | | | | | | | | | | | | | | | | | | | | | | | |
|  | | 1395.6895 | | | | | | | | 1395.6877 | | | | | | | | | | | | | | 0 | | | | | | | | | | -1 | | | | | | | | | | | | | 244 | | | | | | | | | | | 255 | | | | | | | | | | | SRNPIYSNNFGK | | | | | | | | | | | | | | | | | | | | | | | | | | |
|  | | 1395.6895 | | | | | | | | 1395.6878 | | | | | | | | | | | | | | 0 | | | | | | | | | | -1 | | | | | | | | | | | | | 244 | | | | | | | | | | | 255 | | | | | | | | | | | SRNPIYSNNFGK | | | | | | | | | | | | | | | | | | | | | | | | | | |
|  | | 1395.6895 | | | | | | | | 1395.6886 | | | | | | | | | | | | | | 0 | | | | | | | | | | -1 | | | | | | | | | | | | | 244 | | | | | | | | | | | 255 | | | | | | | | | | | SRNPIYSNNFGK | | | | | | | | | | | | | | | | | | | | | | | | | | |
|  | | 1152.5564 | | | | | | | | 1152.5546 | | | | | | | | | | | | | | 0 | | | | | | | | | | -1 | | | | | | | | | | | | | 246 | | | | | | | | | | | 255 | | | | | | | | | | | NPIYSNNFGK | | | | | | | | | | | | | | | | | | | | | | | | | | |
|  | | 1009.512 | | | | | | | | 1009.5097 | | | | | | | | | | | | | | 0 | | | | | | | | | | -2 | | | | | | | | | | | | | 256 | | | | | | | | | | | 263 | | | | | | | | | | | FFEITPEK | | | | | | | | | | | | | | | | | | | | | | | | | | |
|  | | 1009.512 | | | | | | | | 1009.5097 | | | | | | | | | | | | | | 0 | | | | | | | | | | -2 | | | | | | | | | | | | | 256 | | | | | | | | | | | 263 | | | | | | | | | | | FFEITPEK | | | | | | | | | | | | | | | | | | | | | | | | | | |
|  | | 1009.512 | | | | | | | | 1009.5106 | | | | | | | | | | | | | | 0 | | | | | | | | | | -1 | | | | | | | | | | | | | 256 | | | | | | | | | | | 263 | | | | | | | | | | | FFEITPEK | | | | | | | | | | | | | | | | | | | | | | | | | | |
|  | | 1009.512 | | | | | | | | 1009.512 | | | | | | | | | | | | | | 0 | | | | | | | | | | 0 | | | | | | | | | | | | | 256 | | | | | | | | | | | 263 | | | | | | | | | | | FFEITPEK | | | | | | | | | | | | | | | | | | | | | | | | | | |
|  | | 1009.512 | | | | | | | | 1009.5124 | | | | | | | | | | | | | | 0 | | | | | | | | | | 0 | | | | | | | | | | | | | 256 | | | | | | | | | | | 263 | | | | | | | | | | | FFEITPEK | | | | | | | | | | | | | | | | | | | | | | | | | | |
|  | | 1009.512 | | | | | | | | 1009.5126 | | | | | | | | | | | | | | 0 | | | | | | | | | | 1 | | | | | | | | | | | | | 256 | | | | | | | | | | | 263 | | | | | | | | | | | FFEITPEK | | | | | | | | | | | | | | | | | | | | | | | | | | |
|  | | 1617.8515 | | | | | | | | 1617.8504 | | | | | | | | | | | | | | 0 | | | | | | | | | | -1 | | | | | | | | | | | | | 256 | | | | | | | | | | | 268 | | | | | | | | | | | FFEITPEKNPQLR | | | | | | | | | | | | | | | | | | | | | | | | | | |
|  | | 1617.8515 | | | | | | | | 1617.8504 | | | | | | | | | | | | | | 0 | | | | | | | | | | -1 | | | | | | | | | | | | | 256 | | | | | | | | | | | 268 | | | | | | | | | | | FFEITPEKNPQLR | | | | | | | | | | | | | | | | | | | | | | | | | | |
|  | | 1617.8515 | | | | | | | | 1617.8506 | | | | | | | | | | | | | | 0 | | | | | | | | | | -1 | | | | | | | | | | | | | 256 | | | | | | | | | | | 268 | | | | | | | | | | | FFEITPEKNPQLR | | | | | | | | | | | | | | | | | | | | | | | | | | |
|  | | 1617.8515 | | | | | | | | 1617.8508 | | | | | | | | | | | | | | 0 | | | | | | | | | | 0 | | | | | | | | | | | | | 256 | | | | | | | | | | | 268 | | | | | | | | | | | FFEITPEKNPQLR | | | | | | | | | | | | | | | | | | | | | | | | | | |
|  | | 1617.8515 | | | | | | | | 1617.8509 | | | | | | | | | | | | | | 0 | | | | | | | | | | 0 | | | | | | | | | | | | | 256 | | | | | | | | | | | 268 | | | | | | | | | | | FFEITPEKNPQLR | | | | | | | | | | | | | | | | | | | | | | | | | | |
|  | | 1617.8515 | | | | | | | | 1617.8512 | | | | | | | | | | | | | | 0 | | | | | | | | | | 0 | | | | | | | | | | | | | 256 | | | | | | | | | | | 268 | | | | | | | | | | | FFEITPEKNPQLR | | | | | | | | | | | | | | | | | | | | | | | | | | |
|  | | 1511.7579 | | | | | | | | 1511.7577 | | | | | | | | | | | | | | 0 | | | | | | | | | | 0 | | | | | | | | | | | | | 318 | | | | | | | | | | | 329 | | | | | | | | | | | QKQEEEPLEVQR | | | | | | | | | | | | | | | | | | | | | | | | | | |
|  | | 1360.7099 | | | | | | | | 1360.7076 | | | | | | | | | | | | | | 0 | | | | | | | | | | -2 | | | | | | | | | | | | | 371 | | | | | | | | | | | 382 | | | | | | | | | | | NFLAGEKDNVVR | | | | | | | | | | | | | | | | | | | | | | | | | | |
|  | | 1360.7099 | | | | | | | | 1360.7084 | | | | | | | | | | | | | | 0 | | | | | | | | | | -1 | | | | | | | | | | | | | 371 | | | | | | | | | | | 382 | | | | | | | | | | | NFLAGEKDNVVR | | | | | | | | | | | | | | | | | | | | | | | | | | |
|  | | 1360.7099 | | | | | | | | 1360.7084 | | | | | | | | | | | | | | 0 | | | | | | | | | | -1 | | | | | | | | | | | | | 371 | | | | | | | | | | | 382 | | | | | | | | | | | NFLAGEKDNVVR | | | | | | | | | | | | | | | | | | | | | | | | | | |
|  | | 1360.7099 | | | | | | | | 1360.7088 | | | | | | | | | | | | | | 0 | | | | | | | | | | -1 | | | | | | | | | | | | | 371 | | | | | | | | | | | 382 | | | | | | | | | | | NFLAGEKDNVVR | | | | | | | | | | | | | | | | | | | | | | | | | | |
|  | | 1360.7099 | | | | | | | | 1360.7095 | | | | | | | | | | | | | | 0 | | | | | | | | | | 0 | | | | | | | | | | | | | 371 | | | | | | | | | | | 382 | | | | | | | | | | | NFLAGEKDNVVR | | | | | | | | | | | | | | | | | | | | | | | | | | |
|  | | 1360.7099 | | | | | | | | 1360.7098 | | | | | | | | | | | | | | 0 | | | | | | | | | | 0 | | | | | | | | | | | | | 371 | | | | | | | | | | | 382 | | | | | | | | | | | NFLAGEKDNVVR | | | | | | | | | | | | | | | | | | | | | | | | | | |
|  | | 1360.7099 | | | | | | | | 1360.7101 | | | | | | | | | | | | | | 0 | | | | | | | | | | 0 | | | | | | | | | | | | | 371 | | | | | | | | | | | 382 | | | | | | | | | | | NFLAGEKDNVVR | | | | | | | | | | | | | | | | | | | | | | | | | | |
|  | | 1360.7099 | | | | | | | | 1360.7107 | | | | | | | | | | | | | | 0 | | | | | | | | | | 1 | | | | | | | | | | | | | 371 | | | | | | | | | | | 382 | | | | | | | | | | | NFLAGEKDNVVR | | | | | | | | | | | | | | | | | | | | | | | | | | |
|  | | 1360.7099 | | | | | | | | 1360.7107 | | | | | | | | | | | | | | 0 | | | | | | | | | | 1 | | | | | | | | | | | | | 371 | | | | | | | | | | | 382 | | | | | | | | | | | NFLAGEKDNVVR | | | | | | | | | | | | | | | | | | | | | | | | | | |
|  | | 1772.8693 | | | | | | | | 1772.8666 | | | | | | | | | | | | | | 0 | | | | | | | | | | -2 | | | | | | | | | | | | | 387 | | | | | | | | | | | 402 | | | | | | | | | | | QVQELAFPGSAQDVER | | | | | | | | | | | | | | | | | | | | | | | | | | |
|  | | 1772.8693 | | | | | | | | 1772.8674 | | | | | | | | | | | | | | 0 | | | | | | | | | | -1 | | | | | | | | | | | | | 387 | | | | | | | | | | | 402 | | | | | | | | | | | QVQELAFPGSAQDVER | | | | | | | | | | | | | | | | | | | | | | | | | | |
|  | | 1772.8693 | | | | | | | | 1772.8674 | | | | | | | | | | | | | | 0 | | | | | | | | | | -1 | | | | | | | | | | | | | 387 | | | | | | | | | | | 402 | | | | | | | | | | | QVQELAFPGSAQDVER | | | | | | | | | | | | | | | | | | | | | | | | | | |
|  | | 1772.8693 | | | | | | | | 1772.8678 | | | | | | | | | | | | | | 0 | | | | | | | | | | -1 | | | | | | | | | | | | | 387 | | | | | | | | | | | 402 | | | | | | | | | | | QVQELAFPGSAQDVER | | | | | | | | | | | | | | | | | | | | | | | | | | |
|  | | 1772.8693 | | | | | | | | 1772.868 | | | | | | | | | | | | | | 0 | | | | | | | | | | -1 | | | | | | | | | | | | | 387 | | | | | | | | | | | 402 | | | | | | | | | | | QVQELAFPGSAQDVER | | | | | | | | | | | | | | | | | | | | | | | | | | |
|  | | 1772.8693 | | | | | | | | 1772.8696 | | | | | | | | | | | | | | 0 | | | | | | | | | | 0 | | | | | | | | | | | | | 387 | | | | | | | | | | | 402 | | | | | | | | | | | QVQELAFPGSAQDVER | | | | | | | | | | | | | | | | | | | | | | | | | | |
|  | | 1438.6729 | | | | | | | | 1438.6716 | | | | | | | | | | | | | | 0 | | | | | | | | | | -1 | | | | | | | | | | | | | 409 | | | | | | | | | | | 420 | | | | | | | | | | | ESYFVDAQPQQK | | | | | | | | | | | | | | | | | | | | | | | | | | |
|  | | 1968.9065 | | | | | | | | 1968.9018 | | | | | | | | | | | | | | 0 | | | | | | | | | | -2 | | | | | | | | | | | | | 409 | | | | | | | | | | | 425 | | | | | | | | | | | ESYFVDAQPQQKEEGSK | | | | | | | | | | | | | | | | | | | | | | | | | | |
|  | | 1968.9065 | | | | | | | | 1968.9079 | | | | | | | | | | | | | | 0 | | | | | | | | | | 1 | | | | | | | | | | | | | 409 | | | | | | | | | | | 425 | | | | | | | | | | | ESYFVDAQPQQKEEGSK | | | | | | | | | | | | | | | | | | | | | | | | | | |
| Spot no. | | Homologous protein | | | | | | | | Accession no. | | | | | | | Score | | | | | | | | | | | M.P. | | | | | | | | | | | | | | | Cov. (%) | | | | | | | | | | | | Blast score | | | | | | | | | | | Mr (kDa) / pI | | | | | | | | | | | | | | | | | | | | Ratio | | | | | | | | | |
|  |  |  |  |  |  |  |  |  |  |  |  |  |  |  |  |  |  |  |  |  |  |  |  |  |  |  |  |  |  |  |  |  |  |  |  |  |  |  |  |  |  |  |  |  |  |  |  |  |  |  |  |  |  |  |  |  |  |  |  |  |  |  |  |  |  | Theo. | | | | | | | | | | Exp. | | | | | | | | | |  |  |  |  |  |  |  |  |  |  |
| 25b | | Sucrose-binding protein | | | | | | | | Glyma10g03390.1 | | | | | | | 264 | | | | | | | | | | | 8 | | | | | | | | | | | | | | | 19 | | | | | | | | | | | | 741 | | | | | | | | | | | 58.6/6.08 | | | | | | | | | |  | | | | | | | | |  | | | | | | | | | | |
|  | | Calc. Mass | | | | | | | | Observ. Mass | | | | | | | Da | | | | | | | | | | | | ppm | | | | | | | | | | | | | | | | start | | | | | | | | | | | | | | end | | | | | | | | | | | sequence | | | | | | | | | | | | | | | | | | | | | | | | |  |
|  | | 1303.7248 | | | | | | | | 1303.7228 | | | | | | | 0 | | | | | | | | | | | | -2 | | | | | | | | | | | | | | | | 139 | | | | | | | | | | | | | | 149 | | | | | | | | | | | SKLLQGIENFR | | | | | | | | | | | | | | | | | | | | | | | | |  |
|  | | 1360.7086 | | | | | | | | 1360.7076 | | | | | | | 0 | | | | | | | | | | | | -1 | | | | | | | | | | | | | | | | 179 | | | | | | | | | | | | | | 191 | | | | | | | | | | | AVLGLVSESETEK | | | | | | | | | | | | | | | | | | | | | | | | |  |
|  | | 1360.7086 | | | | | | | | 1360.7078 | | | | | | | 0 | | | | | | | | | | | | -1 | | | | | | | | | | | | | | | | 179 | | | | | | | | | | | | | | 191 | | | | | | | | | | | AVLGLVSESETEK | | | | | | | | | | | | | | | | | | | | | | | | |  |
|  | | 1360.7086 | | | | | | | | 1360.7084 | | | | | | | 0 | | | | | | | | | | | | 0 | | | | | | | | | | | | | | | | 179 | | | | | | | | | | | | | | 191 | | | | | | | | | | | AVLGLVSESETEK | | | | | | | | | | | | | | | | | | | | | | | | |  |
|  | | 1360.7086 | | | | | | | | 1360.7084 | | | | | | | 0 | | | | | | | | | | | | 0 | | | | | | | | | | | | | | | | 179 | | | | | | | | | | | | | | 191 | | | | | | | | | | | AVLGLVSESETEK | | | | | | | | | | | | | | | | | | | | | | | | |  |
|  | | 1141.5193 | | | | | | | | 1141.518 | | | | | | | 0 | | | | | | | | | | | | -1 | | | | | | | | | | | | | | | | 236 | | | | | | | | | | | | | | 245 | | | | | | | | | | | FEEFFGPGGR | | | | | | | | | | | | | | | | | | | | | | | | |  |
|  | | 1723.8529 | | | | | | | | 1723.8508 | | | | | | | 0 | | | | | | | | | | | | -1 | | | | | | | | | | | | | | | | 273 | | | | | | | | | | | | | | 287 | | | | | | | | | | | LFDQQNEGSIFAISR | | | | | | | | | | | | | | | | | | | | | | | | |  |
|  | | 1886.901 | | | | | | | | 1886.8996 | | | | | | | 0 | | | | | | | | | | | | -1 | | | | | | | | | | | | | | | | 328 | | | | | | | | | | | | | | 343 | | | | | | | | | | | LTEVGPDDDEKSWLQR | | | | | | | | | | | | | | | | | | | | | | | | |  |
|  | | 855.5178 | | | | | | | | 855.5182 | | | | | | | 0 | | | | | | | | | | | | 1 | | | | | | | | | | | | | | | | 370 | | | | | | | | | | | | | | 377 | | | | | | | | | | | IALVIDGR | | | | | | | | | | | | | | | | | | | | | | | | |  |
|  | | 1508.6976 | | | | | | | | 1508.6971 | | | | | | | 0 | | | | | | | | | | | | 0 | | | | | | | | | | | | | | | | 378 | | | | | | | | | | | | | | 390 | | | | | | | | | | | GHLQISCPHMSSR | | | | | | | | | | | | | | | | | | | | | | | | |  |
|  | | 1273.6514 | | | | | | | | 1273.6504 | | | | | | | 0 | | | | | | | | | | | | -1 | | | | | | | | | | | | | | | | 455 | | | | | | | | | | | | | | 466 | | | | | | | | | | | DNIVSSLDNVAK | | | | | | | | | | | | | | | | | | | | | | | | |  |
|  | | 1273.6514 | | | | | | | | 1273.6518 | | | | | | | 0 | | | | | | | | | | | | 0 | | | | | | | | | | | | | | | | 455 | | | | | | | | | | | | | | 466 | | | | | | | | | | | DNIVSSLDNVAK | | | | | | | | | | | | | | | | | | | | | | | | |  |
