# Supplementary material for: Proteomic and Biochemical Analyses of the Cotyledon and Root of Flooding-Stressed Soybean Plants
Source: PLoS One. 2013 Jun 14;8(6):e65301. doi: 10.1371/journal.pone.0065301 (PMC3683008; doi:10.1371/journal.pone.0065301)
Supplement: Table S5 — Common proteins to both cotyledon and root of flooding-stressed soybean plants. (DOCX) [file pone.0065301.s011.docx]

| Table S5 Common proteins between cotyledon and root of soybean under flooding stress. | | | | | | | | |
| --- | --- | --- | --- | --- | --- | --- | --- | --- |
| Homologous protein | Spot No | Accession No | Score | Cov(%) | M.P. | Blast score |  |  |
|  |  |  |  |  |  |  | Theo.  Mr (kDa) / pI | F.C. |
| Heat shock 70 kDa protein | C15 | Glyma17g08020.1  P26413 | 130 | 24 | 13 (6) | 1144 | 71.2/5.3 | 6.7 |
| Heat shock 70 kDa protein | R5 | Glyma17g08020.1  P26413 | 2152 | 38 | 25 (13) | 1144 | 71.2/5.3 | 1.4 |
